# Allosteric inhibition of SHP2 uncovers aberrant TLR7 trafficking in aggravating psoriasis

**DOI:** 10.15252/emmm.202114455

**Published:** 2021-12-22

**Authors:** Yuyu Zhu, Zhigui Wu, Wei Yan, Fenli Shao, Bowen Ke, Xian Jiang, Jian Gao, Wenjie Guo, Yuping Lai, Hongyue Ma, Dijun Chen, Qiang Xu, Yang Sun

**Affiliations:** ^1^ State Key Laboratory of Pharmaceutical Biotechnology Department of Biotechnology and Pharmaceutical Sciences School of Life Sciences Nanjing University Nanjing China; ^2^ College of Pharmacy Nanjing University of Chinese Medicine Nanjing China; ^3^ Department of Dermatology and Venereology West China Hospital Sichuan University Chengdu China; ^4^ Laboratory of Anesthesia and Critical Care Medicine Department of Anesthesiology Translational Neuroscience Center, West China Hospital and State Key Laboratory of Biotherapy Sichuan University Chengdu Sichuan China; ^5^ Shanghai Key Laboratory of Regulatory Biology School of Life Sciences East China Normal University Shanghai China; ^6^ Chemistry and Biomedicine Innovation Center (ChemBIC) Nanjing University Nanjing China

**Keywords:** psoriasis, PTPN11, scRNA‐seq, therapeutic target, TLR7, Immunology, Skin

## Abstract

Psoriasis is a complex chronic inflammatory skin disease with unclear molecular mechanisms. We found that the Src homology‐2 domain‐containing protein tyrosine phosphatase‐2 (SHP2) was highly expressed in both psoriatic patients and imiquimod (IMQ)‐induced psoriasis‐like mice. Also, the SHP2 allosteric inhibitor SHP099 reduced pro‐inflammatory cytokine expression in PBMCs taken from psoriatic patients. Consistently, SHP099 significantly ameliorated IMQ‐triggered skin inflammation in mice. Single‐cell RNA sequencing of murine skin demonstrated that SHP2 inhibition impaired skin inflammation in myeloid cells, especially macrophages. Furthermore, IMQ‐induced psoriasis‐like skin inflammation was significantly alleviated in myeloid cells (monocytes, mature macrophages, and granulocytes)—but not dendritic cells conditional SHP2 knockout mice. Mechanistically, SHP2 promoted the trafficking of toll‐like receptor 7 (TLR7) from the Golgi to the endosome in macrophages by dephosphorylating TLR7 at Tyr1024, boosting the ubiquitination of TLR7 and NF‐*κ*B‐mediated skin inflammation. Importantly, *Tlr7* point‐mutant knock‐in mice showed an attenuated psoriasis‐like phenotype compared to wild‐type littermates following IMQ treatment. Collectively, our findings identify SHP2 as a novel regulator of psoriasis and suggest that SHP2 inhibition may be a promising therapeutic approach for psoriatic patients.

## Graphical abstract

From the clinical level of psoriasis patients, the whole animal level, and the cellular level, this study reveals that SHP2 promotes TLR7 trafficking to endosomes and activates the downstream NF‐κB pathway through dephosphorylation of TLR7, thus exacerbating the pathogenesis of psoriasis and providing a potential target for the development of therapeutic drugs for psoriasis.

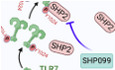

**The paper explained**

**Problem**

Psoriasis is a common and complex chronic inflammatory skin disease that is characterized by epidermal hyperplasia, aberrant differentiation of keratinocytes, exaggerated angiogenesis, and dermal infiltration of inflammatory cells. It affects 2–3% of the global population. However, the exact molecular mechanisms remain elusive.

**Results**

We unravel a novel regulation by SHP2 in TLR7‐mediated NF‐*κ*B signaling activation in macrophages. Such positive regulation of SHP2, through dephosphorylating TLR7 at Tyr1024, promotes the trafficking of TLR7 to the endosome, and maintains excessive activation of TLR7/NF‐*κ*B signaling. Together, our studies identify SHP2 as a novel driver for the pathogenesis of psoriasis and suggest that SHP2 inhibition as a promising therapeutic approach for the treatment of autoimmune diseases, such as psoriasis.

**Impact**

In biology, we identify SHP2 as a novel accelerator of psoriasis progression via enhancing TLR7 trafficking. In addition, this is the first report that TLR7 can be phosphorylated at Tyr1024 in the skin lesions of psoriatic patients and its dephosphorylation by SHP2 aggravates the development of psoriasis. In therapeutics, allosteric inhibition of SHP2 ameliorates murine psoriasis‐like skin inflammation model, suggesting SHP2 is a promising therapeutic target for patients with psoriasis. Our findings further highlight the critical role of phosphatase SHP2 in autoimmune diseases.

## Introduction

Psoriasis is a common and complex chronic inflammatory skin disease that is characterized by epidermal hyperplasia, aberrant differentiation of keratinocytes, exaggerated angiogenesis, and dermal infiltration of inflammatory cells (Perera *et al*, 2012; Boehncke & Schön, 2015; Lebwohl, 2018). It affects 2–3% of the global population (Crow, 2012). The imiquimod (IMQ)‐induced mouse model largely mimics the psoriasis phenotype in humans (van der Fits *et al*, 2009) and has been widely used for the pre‐clinical study of psoriasis. Recent studies using the IMQ‐induced mouse model have identified the involvement of the IL‐23/IL‐17 axis in psoriasis (Boehncke & Schön, 2015; Kopp *et al*, 2015; Burkett & Kuchroo, 2016; Lebwohl, 2019). Activated macrophages and dendritic cells (DCs) generate IL‐23, TNF‐*α*, IL‐6, and IL‐1*β* to stimulate and polarize helper T (Th) cells to transition into Th1, Th17, and Th22 cell subsets, which, respectively, produce IFN‐*γ*, IL‐17A/F, and IL‐22. These pro‐inflammatory cytokines then promote the proliferation and activation of keratinocyte cells, which in turn produce IL‐23 and aggravate the inflammation and psoriasis progression. In psoriatic patients, the major TNF‐*α*‐producing cells are the slan^+^ macrophages (Brunner *et al*, 2013). The reduction in these cells during psoriasis remission underlines their critical role in psoriasis development, maintenance, or both (Brunner *et al*, 2013), emphasizing an important role for macrophages in the development of psoriasis. However, the cellular and molecular mechanisms of psoriasis underlying its development remain unclear.

The Src homology‐2 domain‐containing protein tyrosine phosphatase‐2 (SHP2) is a ubiquitously expressed cytoplasmic tyrosine phosphatase containing two SH2 domains and one catalytic protein tyrosine phosphatase (PTP) domain (Feng *et al*, 1993). It is encoded by *PTPN11* and has critical functions in cell proliferation, differentiation, and survival (Qu, 2002). SHP2 is inactive in the basal state, but when combined with a phosphotyrosine (pY) protein, it is activated as a phosphatase to elicit downstream signaling (Barford & Neel, 1998). SHP2 missense mutations account for approximately 50% of the cases of Noonan syndrome (Keilhack *et al*, 2005), a common autosomal dominant disorder characterized by multiple, variably penetrant defects. Intriguingly, a 55‐year‐old woman with many cardinal physical characteristics of Noonan syndrome also suffered simultaneously from annular pustular psoriasis (Catharino *et al*, 2016), suggesting a potential role of SHP2 in psoriasis; however, this possibility has not been investigated either *in vitro* or *in vivo*.

Other possible effectors of psoriasis are the toll‐like receptors (TLRs), which are essential sensors of a variety of viral and microbial infections and endogenously derived damage‐associated molecular patterns (DAMPs) (Bryant *et al*, 2015). TLRs recruit adaptor proteins and activate transcription factors to produce inflammatory cytokines that promote the initiation of adaptive immune responses (Newman *et al*, 2016). The TLR family consists of 10 members in humans and 13 in mice, which are expressed on the cell surface and intracellularly (Barbalat *et al*, 2011). The intracellular TLRs, including TLR3, TLR7, TLR8, and TLR9 in humans, recognize nucleic acids (Blasius & Beutler, 2010). TLR7 recognizes single‐stranded RNA and traffics it to endosomes (Wang *et al*, 2020). Imiquimod (IMQ) is a TLR7 ligand that has been used for the topical treatment of genital and perianal warts caused by the human papillomavirus (Beutner & Tyring, 1997). Interestingly, topical IMQ treatment of patients with actinic keratoses and superficial basal cell carcinomas can exacerbate even previously well‐controlled psoriasis (Gilliet *et al*, 2004). Such IMQ‐exacerbated psoriasis occurs both in the topically treated areas and at distant, previously unaffected skin sites (Wu *et al*, 2004). These findings suggest, but have not yet confirmed, that TLR7 activation may promote psoriasis.

In this study, we performed single‐cell RNA sequencing (scRNA‐seq), a powerful and unbiased method of examining the pathological processes of human disease (Shalek & Benson, 2017), to profile the different transcriptomics of the skin microenvironment at a single‐cell level with the SHP2 allosteric inhibitor SHP099. Unsupervised analyses revealed that SHP099 impaired skin inflammation in myeloid cells (especially macrophages). Ablation of SHP2 in macrophages weakened NF‐*κ*B activation, subsequently resulting in amelioration of IMQ‐induced psoriasis‐like skin inflammation in mice. Mechanistically, SHP2 dephosphorylated TLR7 at Tyr1024 to promote its ubiquitination in macrophages and trafficking to the endosome. Overall, our findings identify SHP2 as a critical regulator of psoriasis and as a potential therapeutic target for the treatment of psoriasis‐related skin diseases.

## Results

### SHP2 expression increased in both human psoriatic patients and IMQ‐induced psoriasis‐like mice

**Figure 1:**
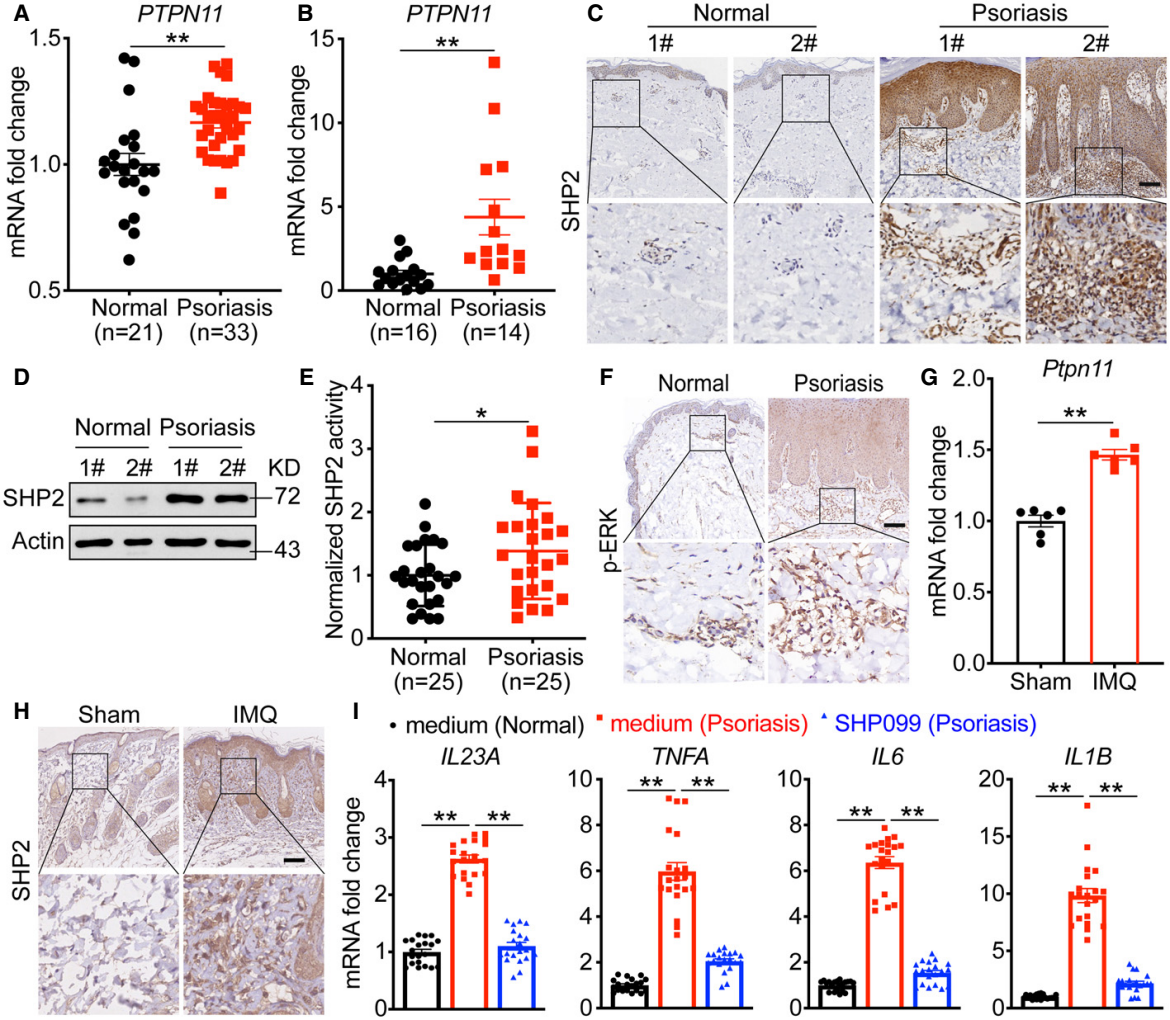
SHP2 expression increased in both human psoriatic patients and IMQ‐induced psoriasis‐like mice Expression of *PTPN11* (gene encoding SHP2) in skin lesions in psoriatic patients compared with skin from healthy donors based on microarray data (No. GSE14905).Expression of *PTPN11* in human PBMCs of psoriatic patients (*n* = 14) and normal controls (*n* = 16).Representative SHP2 staining in skin sections of psoriatic patients (*n* = 13) and normal controls (*n* = 5). Scale bars: 200 μm.Western blot analysis of PBMCs lysates derived from psoriatic patients and normal controls.The catalytic activity of SHP2 was measured in human PBMCs lysates derived from psoriatic patients (*n* = 25) and normal controls (*n* = 25).Representative p‐ERK staining of skin sections of psoriatic patients and normal controls. Scale bars: 200 μm.Quantitative PCR analysis of *Ptpn11* mRNA levels in the IMQ‐treated or non‐treated dorsal back of C57BL/6 mice at day 5 (*n* = 6/group). Data were normalized to *Gapdh* expression.Representative histological sections of IMQ‐treated or non‐treated dorsal back of C57BL/6 mice at day 5. Scale bar: 100 μm.Quantitative PCR analysis of mRNA levels in human PBMCs derived from psoriatic patients (*n* = 20) and normal healthy controls (*n* = 20). Data information: Data are represented as mean ± SEM. *P* values are determined by two‐tailed Mann–Whitney *U* test (A and B) or two‐tailed unpaired Student’s *t*‐test (E, G, and I). *P < 0.05, **P < 0.01. Source data are available online for this figure.

To assess whether SHP2 may play a functional role in psoriasis, we first analyzed the expression and activity of SHP2 in human samples collected from healthy donors (normal controls) and psoriatic patients. At the mRNA level, SHP2 (encoded by the *PTPN11* gene) was significantly higher in the skin lesions (Fig 1A) and human peripheral blood mononuclear cells (PBMCs) (Fig 1B) of psoriatic patients than those of normal controls. At the protein level, skin biopsies (Fig 1C) and PBMCs (Fig 1D) from psoriatic patients also showed a considerable increase in SHP2 compared to normal controls. Consistently, enzymatic assays revealed increased SHP2 activity in PBMCs from psoriatic patients versus normal controls (Fig 1E). Additionally, the p‐ERK levels, which were shown to be positively regulated by SHP2, were also higher in the skin lesions of psoriatic patients than those of normal controls (Fig 1F). Similarly, SHP2 expression was increased in the dorsal skin of the IMQ‐induced psoriasis‐like murine model, both at the mRNA and protein levels (Fig 1G and H). Collectively, data from human specimens and murine models indicate an increase in SHP2 functioning in psoriasis.

Next, we treated the PBMCs derived from human donors with SHP099, a potent allosteric inhibitor of SHP2 (Chen *et al*, 2016). As expected, when compared to normal control donors, the PBMCs obtained from psoriatic patients expressed significantly higher levels of psoriasis‐related cytokines; however, when these PBMCs were treated with 10 μM SHP099, these cytokines decreased remarkably, to a level comparable to that in the normal controls (Fig 1I). Furthermore, SHP099 also markedly reduced IMQ‐induced levels of psoriasis‐related cytokines in human PBMCs from healthy donors (Appendix Fig S1), without affecting their basal levels (Appendix Fig S2). Taken together, our data demonstrated that SHP2 expression increased in both human psoriatic patients and IMQ‐induced psoriasis‐like mice.

### SHP2 inhibitor SHP099 attenuated the psoriasis‐like phenotype in the IMQ‐induced murine model

**Figure 2:**
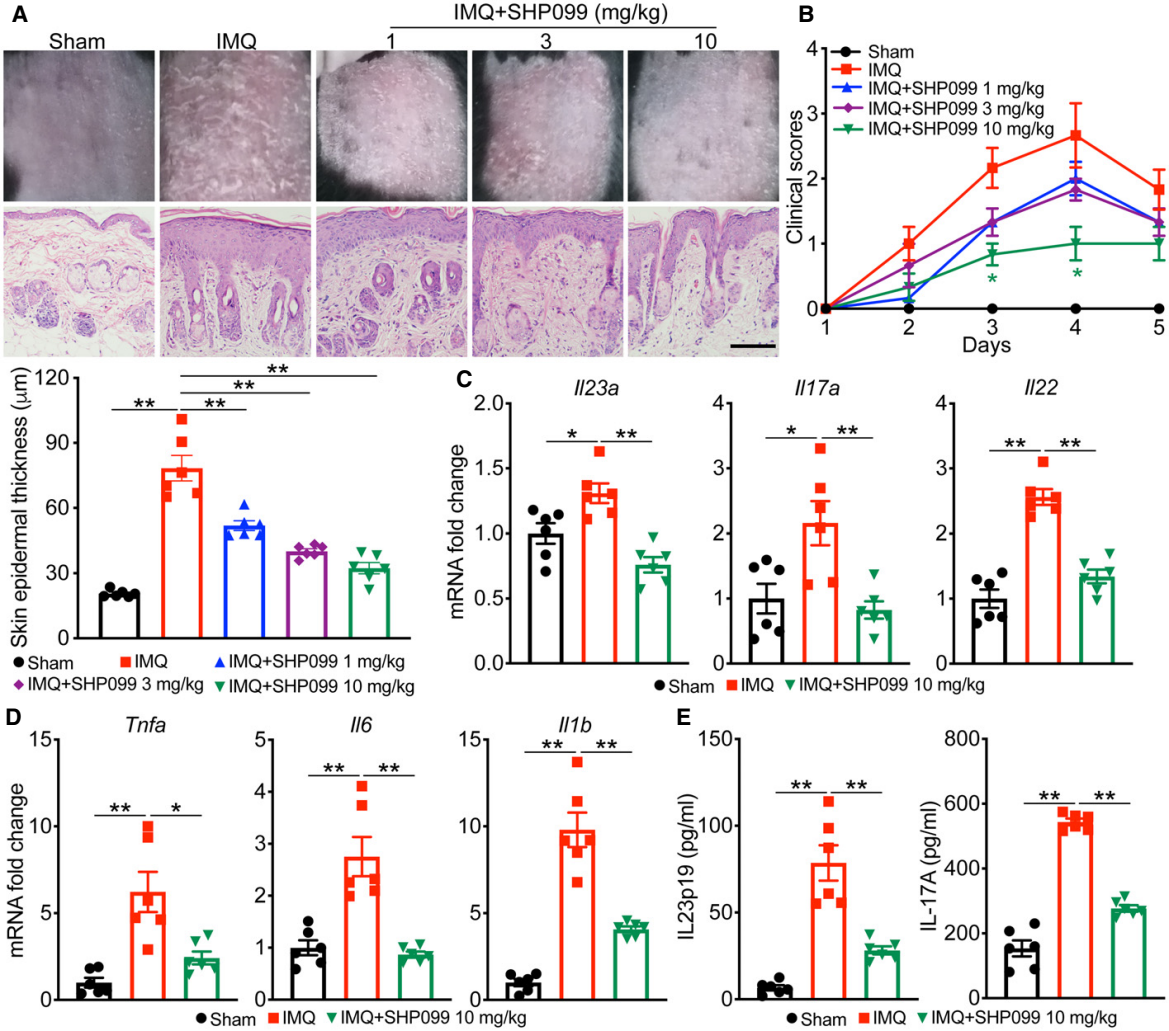
SHP2 inhibitor SHP099 attenuated the psoriasis‐like phenotype in the IMQ‐induced murine model C57BL/6 mice (*n* = 6/group) were treated with indicated dose of SHP099 or vehicle for 4 days. APhenotypic presentation (*top*), H&E staining (*middle*), and statistical data (*bottom*) (mean ± SEM) of dorsal skin. Scale bar: 100 μm.BClinical scores plotted with mean ± SEM. *Denotes statistical significance when compared with the IMQ group.C, DQuantitative PCR analysis of mRNA encoding IL‐23/IL‐17A axis cytokines (C) and other psoriasis‐related cytokines (D) in the dorsal skin of C57BL/6 mice treated with indicated dose of SHP099 or vehicle for 4 days. Results were normalized to *Gapdh* expression.EELISA quantification of protein levels of cytokines in mouse serum. Data information: Data are represented as mean ± SEM. *P* values are determined by two‐tailed unpaired Student’s *t*‐test (A, C–E) or Tukey multiple‐comparison test (B). *P < 0.05, **P < 0.01. Source data are available online for this figure.

To determine whether SHP2 activity affects psoriasis pathogenesis, we treated the murine psoriatic model with SHP099. SHP099 significantly suppressed IMQ‐induced swelling, epidermal acanthosis, keratinocytes proliferation, and dermal inflammatory cell infiltration (Fig 2A), accompanied by a drastic decrease in the clinical scores (Fig 2B). SHP099 did not affect the skin condition of normal mice (Appendix Fig S3).

**Figure EV1:**
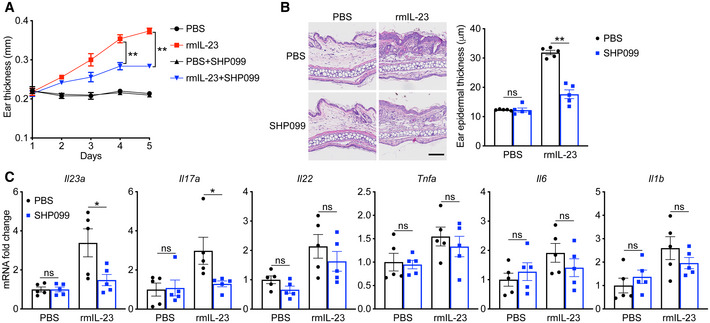
SHP099 attenuated the psoriasis‐like phenotype in the IL‐23‐induced murine model C57BL/6 mice (*n* = 5/group) were subjected to IL‐23‐induced psoriasis‐like skin inflammation and were treated with 10 mg/kg SHP099 or PBS for 4 days. A, BEar thickness (A), H&E staining, and statistic results (B) of ear skin from SHP099 treated with or without C57BL/6 mice injected intradermally with rmIL‐23 or PBS for 4 days. Ear thickness was measured daily.CQuantitative PCR analysis of mRNA encoding IL‐23/IL‐17A axis cytokines and other psoriasis‐related cytokines in the ear skin. Results were normalized to *Gapdh* expression. Data information: Data are represented as mean ± SEM. *P* values are determined by Tukey multiple‐comparison test (A–C). *P < 0.05, **P < 0.01, ns, not significant. Source data are available online for this figure.

Consistent with the ameliorated psoriasis‐like phenotype, the mRNA levels of genes related to the IL‐23/IL‐17 axis, such as *Il23a*, *Il17a*, and *Il22* (Fig 2C), and the expression of *Tnfa*, *Il6*, and *Il1b* was decreased in the skin (Fig 2D). The IL‐23 and IL‐17A serum levels were also significantly reduced by SHP099 (Fig 2E). SHP099 had a moderate effect on the IL‐23‐induced psoriasis‐like mouse model, although not as strong as on the IMQ model. Total ear thickness clearly decreased, but psoriasis‐related inflammatory cytokines were only slightly affected (Fig EV1). Taken together, these data demonstrated that SHP099 ameliorated the IMQ‐induced and IL‐23‐induced psoriasis progression in mice.

### scRNA‐seq analysis revealed that SHP099 reprogrammed IMQ‐induced inflammation in mouse skin

**Figure 3:**
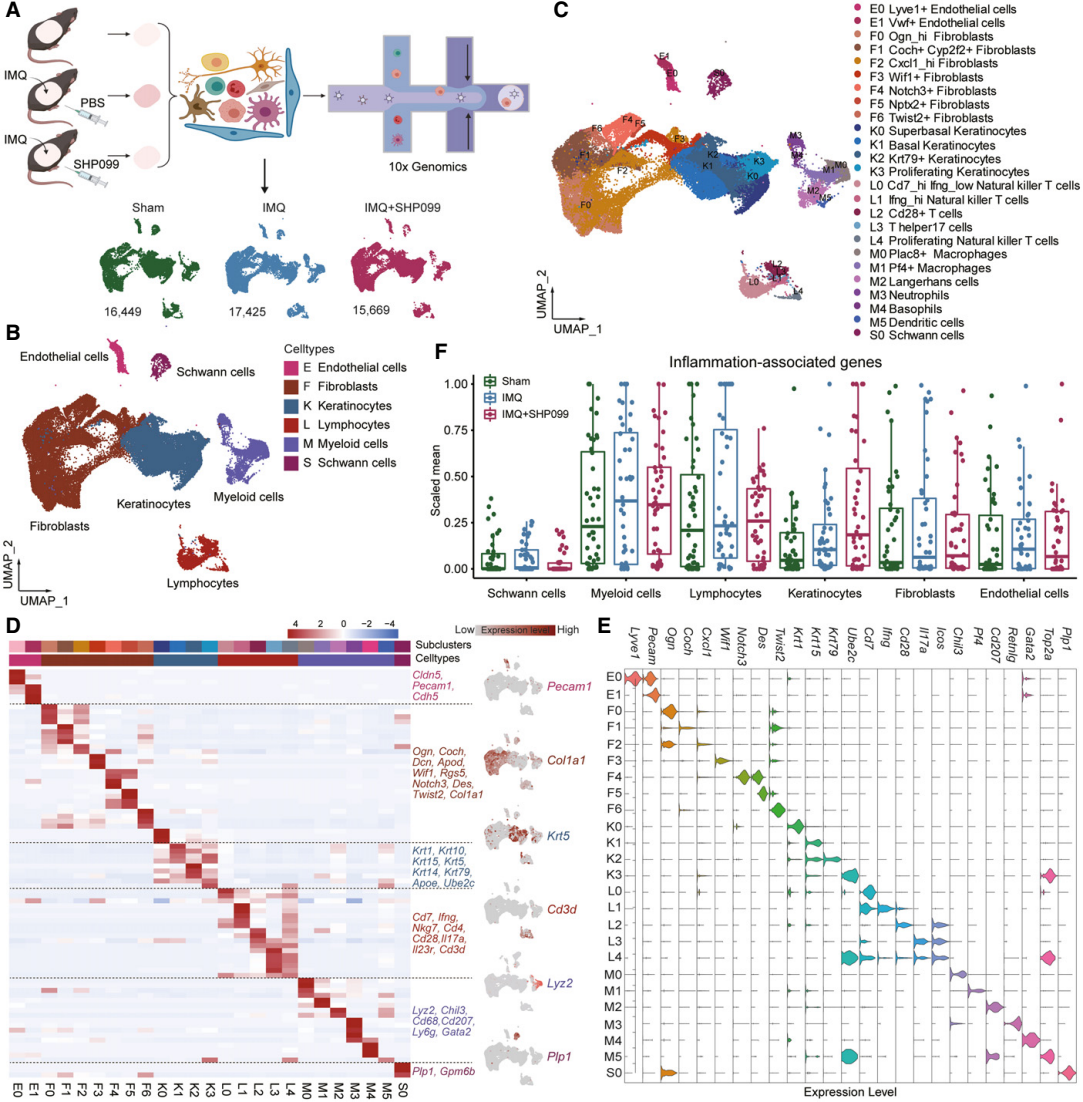
scRNA‐seq analysis revealed that SHP099 reprogrammed IMQ‐induced inflammation in mouse skin Workflow for single‐cell profiling of dorsal skin from the sham, IMQ, and IMQ+SHP099 three groups. The number of cells used in the integration was shown for each sample group (*bottom*). Each group consists of two samples, including two mice per sample in the sham group and three mice in the model and SHP099 administration groups.UMAP visualization of all cells. Clusters are colored and labeled according to their inferred cell type identities.UMAP plot of cells colored by cell subsets.Heatmap displayed the top 20 differentially expressed genes for each cluster (*left*). Selected genes for each cluster are highlighted (*middle*). Feature plots of expression distribution for cluster‐specific marker genes (*right*).Relative expression of selected cluster‐specific genes in each cluster. High‐density bar charts showed the distribution of the normalized expression levels of genes.Violin plot showed the relative expression of 47 inflammation‐associated genes based on sample groups and cell clusters. Source data are available online for this figure.

To clarify the mechanism by which the small‐molecule allosteric inhibitor SHP099 improves psoriasis, we used scRNA‐seq to better understand the pathological processes and determine transcriptomic changes. We dissociated dorsal skin from untreated (Sham) mice, IMQ‐induced (IMQ) mice, and IMQ‐induced mice treated with 10 mg/kg SHP099 (IMQ+SHP099) in a single‐cell suspension and compared unsorted cells from the three groups using scRNA‐seq analysis (Fig 3A). A total of 49,543 cells met the preprocessing threshold (Appendix Fig S4) and were used for downstream analysis. Uniform manifold approximation and projection (UMAP) plotting revealed 6 clusters, including endothelial cells, fibroblasts, keratinocytes, lymphocytes, myeloid cells, and Schwann cells (Fig 3B), which were further separated into 25 subpopulations for more detailed profiling of rare subsets (Fig 3C). Cluster identities were determined by the expression of unique marker genes using heatmaps (Fig 3D and Appendix Fig S5), bar charts (Fig 3E), and feature plots (Appendix Fig S6), revealing successful capture of major cell subsets. Consistent with Fig 2, the inflammation‐associated genes significantly increased in IMQ‐induced mouse skin compared to sham controls, and treatment with SHP099 reduced the expression of inflammation‐associated genes (Fig 3F). Obviously, SHP099 treatment normalized myeloid cells and endothelial cells in psoriasis‐like mouse skin lesions. Overall, these results indicated that the identified cell types and differentially expressed marker genes corresponded to most major known cell types, suggesting that SHP099 improves the IMQ‐induced psoriasis‐like phenotype by inhibiting the expression of inflammation‐associated genes.

### SHP099 regulated genes associated with inflammation in myeloid cells in mouse skin

**Figure 4:**
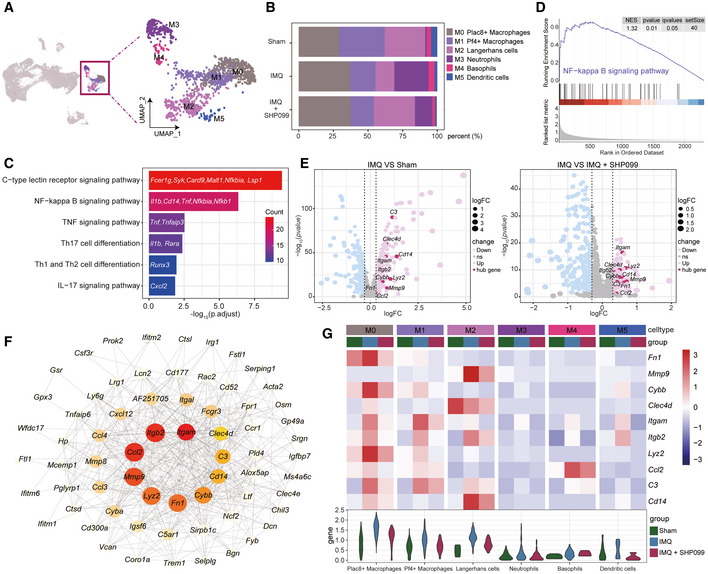
SHP099 regulated genes associated with inflammation in myeloid cells in mouse skin UMAP plot of myeloid cells subclusters.Bar plots showing the percentage (%) of cell types in each sample group.Representative KEGG pathways enriched in upregulated genes in myeloid cells.Gene set enrichment analysis (GSEA) of the NF‐*κ*B signaling pathway.Volcano plots of DEGs that were upregulated (red) or downregulated (blue) in myeloid cells. Examples of DEGs were labeled.Gene networks of DEGs that were upregulated in the IMQ group compared with the sham and SHP099 groups.Expression patterns of the top 10 hub genes in (F). Heatmap (*top*) displays the average expression in the three groups and in different myeloid cell subsets. Boxplots (*bottom*) show the corresponding expression distribution.

To further explore the biological significances of transcriptional changes in the myeloid cells cluster, we first separated the myeloid cells into six clusters: *Plac8* ^+^ macrophages, *Pf4* ^+^ macrophages, Langerhans cells, neutrophils, basophils, and proliferating dendritic cells (Fig 4A). Among these subclusters, macrophages accounted for the largest proportion (Fig 4B). We performed pathway enrichment analysis using upregulated genes in the myeloid cells and found that biological pathways, such as C‐type lectin receptor signaling, NF‐*κ*B signaling, and TNF signaling pathways, were highly enriched (Fig 4C and D; Appendix Figs S7 and S8). To identify IMQ response genes, we examined the overlap of upregulated genes using differential expressed genes (DEGs) analysis of IMQ versus normal and IMQ versus SHP099 in myeloid cells (Fig 4E). We mapped these upregulated genes (*n* = 108) into functional protein association networks from the STRING database (Szklarczyk *et al*, 2019), and identified *Itgam*, *Ccl2*, *Itgb2*, *Mmp9*, *Fn1*, *Lyz2*, *Cybb*, *Cd14*, *C3*, and *Clec4d* as the top 10 hub genes (Fig 4F and Appendix Fig S9). Interestingly, these genes were mainly expressed in macrophages and Langerhans cells (Fig 4G), implying that they may function as master regulators in a cell type‐specific manner.

### SHP2 deficiency in myeloid cells alleviated the psoriasis‐like phenotype in the IMQ‐induced murine model

**Figure 5:**
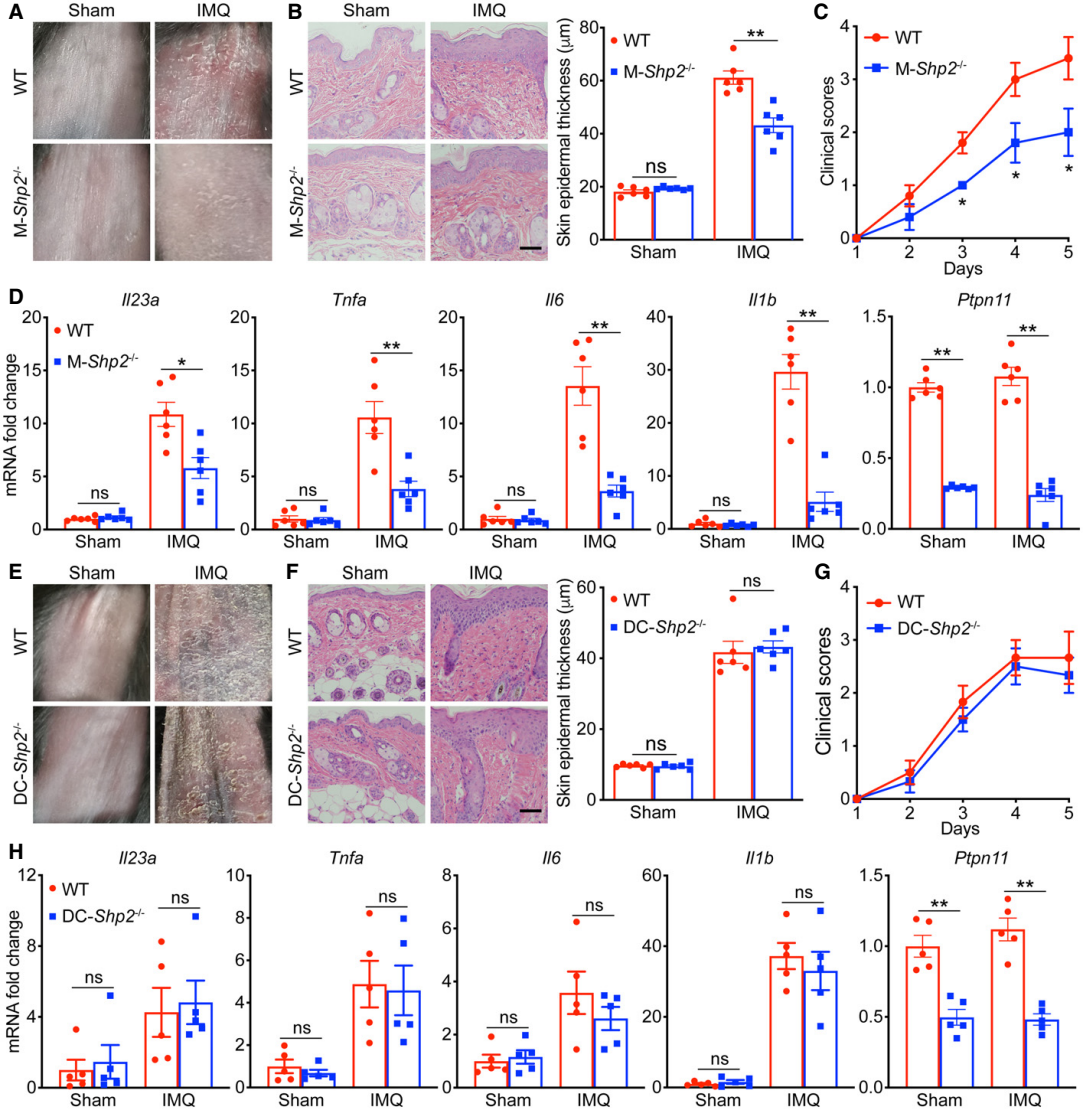
SHP2 deficiency in myeloid cells alleviated the psoriasis‐like phenotype in the IMQ‐induced murine model A–HRepresentative images (A and E), histological sections of dorsal back (B and F), and clinical scores (C and G) and quantitative PCR analysis of mRNA levels in dorsal skin (D and H) from wild‐type (*n* = 6) and M‐*Shp2* ^−/−^ mice or DC‐*Shp2* ^−/−^ mice (*n* = 6) treated with IMQ for 4 days. Scale bar: 100 μm. Left: H&E (hematoxylin and eosin) staining data; right: statistical data (mean ± SEM). Results were normalized to *Gapdh* expression. Data information: Data are represented as mean ± SEM. *P* values were calculated by Tukey multiple‐comparison test (B, D, F, H) or Bonferroni multiple‐comparison test (C, G). *P < 0.05, **P < 0.01, ns, not significant. Source data are available online for this figure.

To determine the causal role of SHP2 in psoriasis, we generated myeloid cell lineages (monocytes, mature macrophages, and granulocytes)‐specific SHP2 conditional knockout mice, called M‐*Shp2* ^−/−^ mice (*Lyz2*‐cre:*Shp2* ^fl/fl^ mice), and confirmed a pretty efficiency of *Shp2* deletion in bone marrow‐derived macrophages (BMDMs), but not in bone marrow‐derived dendritic cells (BMDCs), as shown in Appendix Fig S10A. We treated the animals’ dorsal skin with IMQ, and after 4 days of treatment, the wild‐type mice exhibited swelling, epidermal acanthosis, and greatly increased proliferation of keratinocytes, dermal inflammatory cell infiltration, and inflammatory cytokines. These pathological changes were remarkably ameliorated in M‐*Shp2* ^−/−^ littermates (Fig 5A–D). Although *Lyz2*‐cre is also expressed in neutrophils, given the high knockdown efficiency of *Shp2* in macrophages and our group demonstration that SHP2 deletion in neutrophils alleviates psoriasis‐like skin inflammation in mice (preprint: Ding *et al*, 2021), our work focuses on the role of SHP2 in macrophages. To further examine the effect of SHP2 deletion on myeloid cells on psoriasis, we generated dendritic cells‐specific SHP2 conditional knockout mice, called DC‐*Shp2* ^−/−^ mice (*Itgax*‐cre:*Shp2* ^fl/fl^ mice), and examined the effect of *Shp2* deletion in DCs and macrophages, as shown in Appendix Fig S10B–E. Upon IMQ challenge, psoriasis‐like pathological changes were not significantly different in DC‐*Shp2* ^−/−^ littermates (Fig 5E–H), suggesting that DC‐derived SHP2 did not affect psoriasis progression. A similar decrease in IMQ‐activated inflammatory cytokines (e.g., *Il23a*, *Tnfa*, *Il6*, and *Il1b*) was also observed in the BMDMs (Appendix Fig S11A) and peritoneal macrophages (PMs; Appendix Fig S11B) of the M‐*Shp2* ^−/−^ mice. Notably, such a decrease in the pro‐inflammatory cytokines in M‐*Shp2* ^−/−^ mice was recapitulated by SHP2‐deficient THP‐1 cells compared to the control cells (Appendix Fig S11C). Also, when stimulated with IL‐36, peritoneal macrophages derived from M‐*Shp2* ^−/−^ mice also produced fewer psoriasis‐related cytokines than those derived from the wild‐type littermates (Appendix Fig S11D).

Additionally, there was a substantial increase in CD68^+^ macrophages (Appendix Fig S12A), neutrophils (Appendix Fig S13A), and DCs (Appendix Fig S13B) infiltration in the skin lesions of psoriatic patients compared to normal controls, consistent with Fig 3. Noticeably, there is a clear co‐localization of CD68 and SHP2 occurred in the psoriatic dermis (Appendix Fig S12B). A similar result was observed in the IMQ‐induced psoriasis‐like murine model (Appendix Fig S12C), suggesting that SHP2 was highly expressed in the infiltrated macrophages in the psoriatic skin. To ground our observation in a spatial sense, we performed spatial transcriptome sequencing analysis in normal skin and psoriasis patient lesion skin using the section‐specific barcodes. We obtained two skin sections from normal control and one lesion skin from psoriasis patient (Appendix Fig S14A and B), and we focused on cluster 1 and cluster 3 located in the dermis (Appendix Fig S14C). *PTPN11* was most strongly correlated with macrophages in lesional psoriatic skin (Appendix Fig S14D). And the expression of CD68, which was co‐expressed with *PTPN11*, was also upregulated in the skin lesions of psoriatic (Appendix Fig S14E and F). There are many kinds of cells inside each spot, and the differences between the cells in the spots in Cluster 1 and Cluster 3 of normal control and psoriatic patient are obvious. Normal control had only a few macrophages, whereas psoriatic patient had a large infiltration of macrophages (Appendix Fig S14G), which is consistent with the results of Appendix Fig S12A. Strong cellular interactions, especially between macrophages and fibroblasts, were present in Cluster 1 and Cluster 3 of psoriatic patient (Appendix Fig S14H). The above demonstrates that *PTPN11* in macrophages plays an important role in psoriasis. Taken together, our data demonstrated that SHP2 deficiency in myeloid cells promoted resistance to IMQ‐induced psoriasis in mice.

### SHP2 deficiency in macrophages mitigated psoriasis by suppressing NF‐*κ*B activation

**Figure 6:**
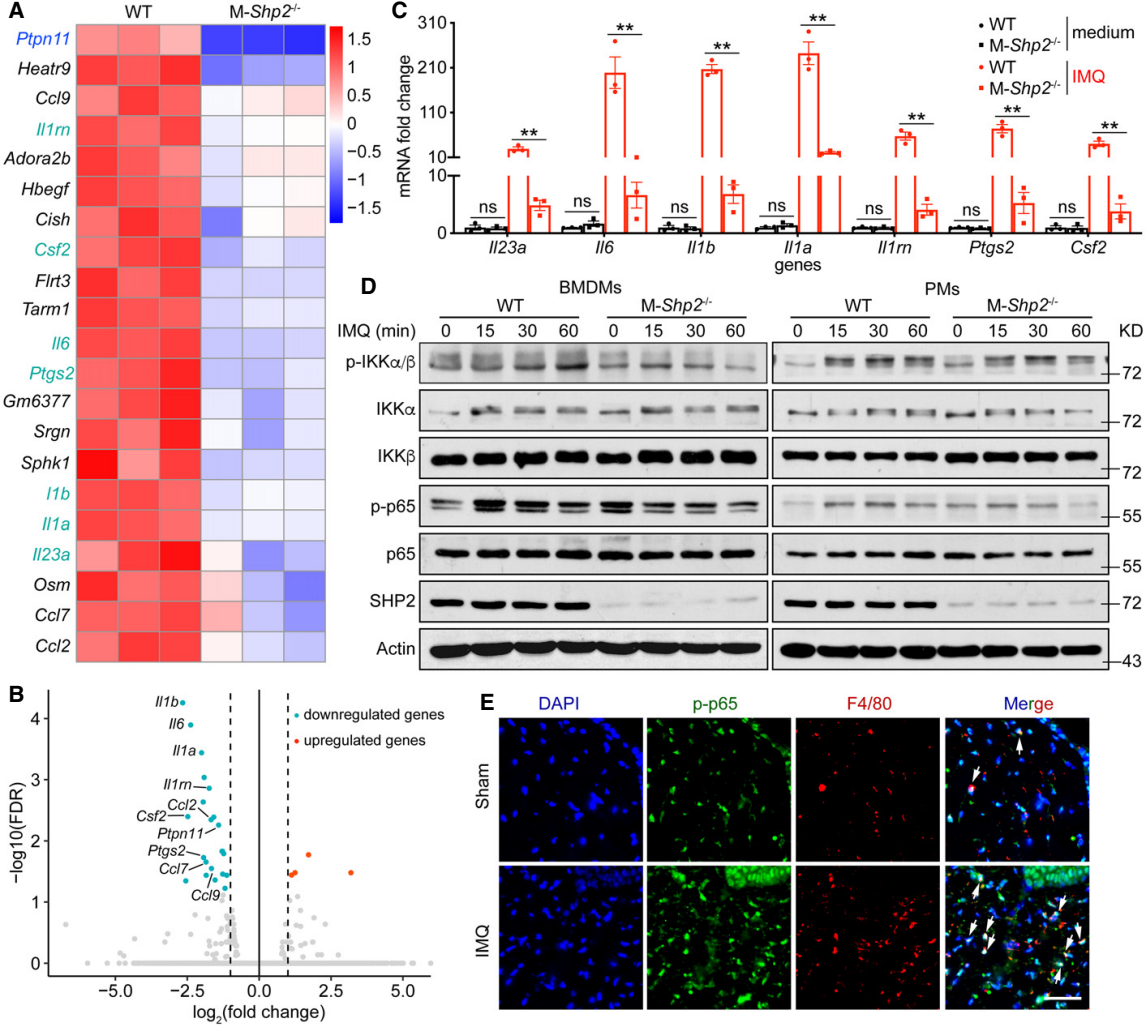
SHP2 deficiency in macrophages mitigated psoriasis by suppressing NF‐*κ*B activation Heat map showing mRNA expression in peritoneal macrophages derived from wild‐type and M‐*Shp2* ^−/−^ mice after IMQ treatment based on RNA sequencing (*n* = 3/group). Colors represent high (red) and low (blue) intensity. Genes labeled in green indicate they are in the NF‐*κ*B signaling.Volcano plot image of upregulated (in red) and downregulated (in green) genes (M‐*Shp2* ^−/−^ mice compared to wild‐type mice) in peritoneal macrophages from wild‐type and M‐*Shp2* ^−/−^ mice after IMQ treatment (*n* = 3/group).Expression levels of representative psoriasis‐related genes decreased in the IMQ model of M‐*Shp2* ^−/−^ mice compared to wild‐type mice (*n* = 3/group).BMDMs and PMs derived from wild‐type and M‐*Shp2* ^−/−^ mice were stimulated by IMQ (10 µg/ml) for indicated times. Whole cell lysates were subjected to Western blotting.Representative p‐p65 staining of skin sections from mouse. Scale bar: 100 μm. Data information: Data are represented as mean ± SEM. *P* values are determined by Tukey multiple‐comparison test (C). **P < 0.01, ns, not significant. Source data are available online for this figure.

**Figure EV2:**
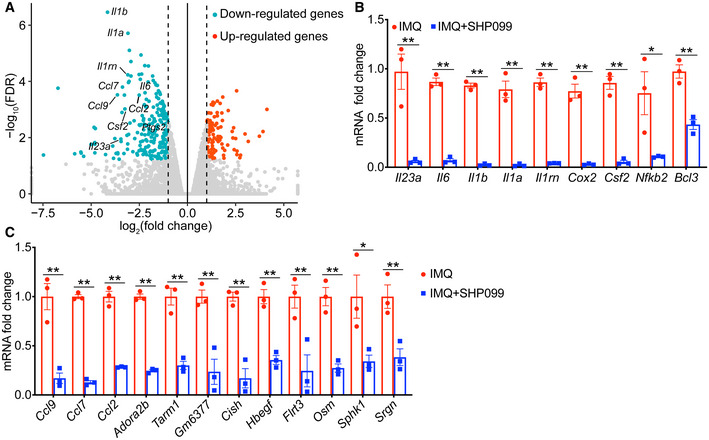
SHP2‐allosteric inhibitor SHP099 prevented psoriasis by downregulating NF‐*κ*B activation AVolcano plot image of upregulated genes (red) and downregulated genes (green) (treated with SHP099 compared to untreated with SHP099) from peritoneal macrophages derived from C57BL/6 mice untreated or pretreated with SHP099 (10 µM) for 2 h and then stimulated by IMQ (10 µg/ml) for 4 h (*n* = 3/group).B, CPeritoneal macrophages derived from C57BL/6 mice were untreated or pretreated with SHP099 (10 µM) for 2 h and then stimulated by IMQ (10 µg/ml) for 4 h. Expression levels of indicated genes decreased in the SHP099 group compared to medium control. Data information: Data are represented as mean ± SEM. *P* values are determined by Tukey multiple‐comparison test (B, C). *P < 0.05, **P < 0.01. Source data are available online for this figure.

RNA sequencing of peritoneal macrophages isolated from wild‐type and M‐*Shp2* ^−/−^ mice and then treated with IMQ for 4 h *in vitro* revealed cytokines enrichment related to NF‐*κ*B signaling (Fig 6A and B). The decrease in several genes in the NF‐*κ*B signaling in macrophages from M‐*Shp2* ^−/−^ mice was confirmed by quantitative PCR (qPCR; Fig 6C). Comparisons between IMQ‐stimulated BMDMs and PMs from wild‐type versus M‐*Shp2* ^−/−^ mice also demonstrated significantly lower phosphorylation levels of IKK*α*/*β* and its downstream p65 in macrophages from M‐*Shp2* ^−/−^ mice, specifically after 15–60 min of IMQ stimulation (Fig 6D). However, after IMQ stimulation, p‐p38 and p‐IRF3 levels were not differentially expressed in the IMQ‐stimulated PMs from M‐*Shp2* ^−/−^ mice and wild‐type mice (Appendix Fig S15). In parallel, analysis of the skin lesions collected from IMQ‐induced mice (Fig 6E) and psoriatic patients (Appendix Fig S16) also revealed significantly higher levels of p‐p65 protein in the infiltrated cells. Despite the small difference in proportion of p65^+^ within the CD68^+^ cells between the normal and psoriasis, both the infiltration of macrophages and the expression of p‐p65 were significantly higher in the lesions of psoriatic patients than in healthy individuals, which contributes to the progression of psoriasis. RNA sequencing of peritoneal macrophages confirmed a significant reduction in the mRNA expression of NF‐*κ*B signaling‐related cytokines in response to IMQ stimulation *in vitro* due to SHP099 (Fig EV2). This result was consistent with Fig 4. Collectively, these data suggested that the loss of SHP2 in macrophages ameliorated the disease severity by attenuating NF‐*κ*B activation. Furthermore, we performed scRNA‐seq on unsorted cells from normal skin and lesional psoriatic skin. After performing unsupervised clustering and a UMAP plot analyses (Appendix Fig S17A), cluster identities were determined by the expression of established markers (Appendix Fig S17B), which indicates successful capture of major skin cell subsets. Heatmap showed that *PTPN11* was positively more correlated with the NF‐*κ*B pathway in macrophages than dendritic cells from psoriasis lesions (Appendix Fig S17C), suggesting that *PTPN11* exacerbates psoriasis mainly in macrophages by activating the NF‐*κ*B pathway.

### SHP2 interacted with TLR7 and promoted TLR7 trafficking to endosomes

**Figure 7:**
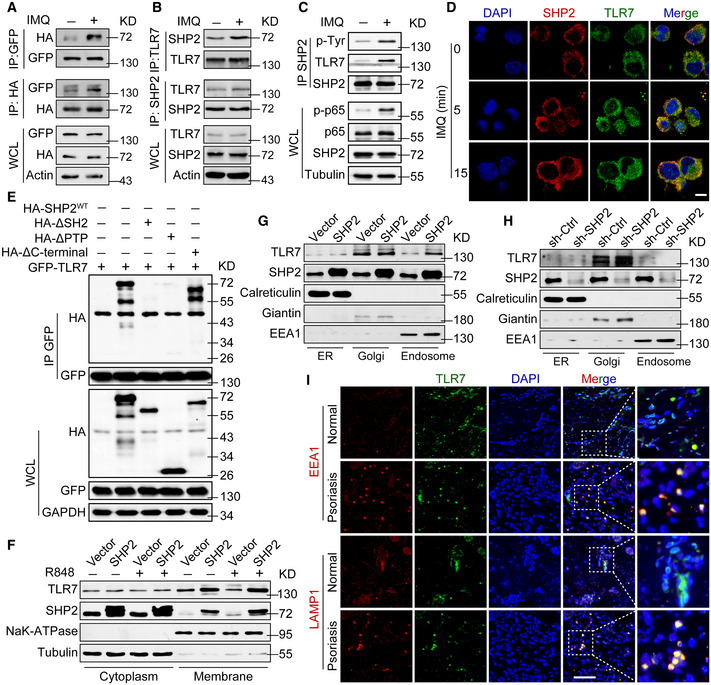
SHP2 interacted with TLR7 and promoted TLR7 trafficking to endosomes AHEK293T cells were transfected with plasmids expressing HA‐tagged SHP2 and GFP‐tagged TLR7. After 48 h, cells were left unstimulated or stimulated by IMQ (10 µg/ml) for 30 min. Cell lysates were immunoprecipitated by anti‐HA or anti‐GFP and probed by indicated antibodies.BPMA‐differentiated THP‐1 cells were unstimulated or stimulated by IMQ (10 µg/ml) for 30 min. Cell lysates were immunoprecipitated by anti‐TLR7 or anti‐SHP2 and probed by indicated antibodies.CBMDMs derived from wild‐type mice were stimulated by IMQ (10 µg/ml) for 30 min. Cell lysates were immunoprecipitated by anti‐SHP2 and probed by indicated antibodies.DRepresentative images of confocal microscopy performed with PMA‐differentiated THP‐1 cells treated with IMQ (10 µg/ml) for indicated durations. Scale bar: 20 µm.EImmunoblotting of HEK293T cells co‐transfected for 48 h with GFP‐TLR7, plus HA‐SHP2, HA‐SHP2 mutant vectors, followed by immunoprecipitation with anti‐GFP beads.FPMA‐differentiated THP‐1 cells were infected with vector or SHP2 lentivirus and then treated with or without R848 (10 µg/ml). Subcellular fractionation was performed, and cytoplasmic and cell membrane proteins were probed by respective antibodies.G, HImmunoblot analysis of TLR7 and SHP2 in the isolated ER, Golgi, and endosome from PMA‐differentiated THP‐1 cells with either SHP2 overexpressed (G) and SHP2 deficiency (H).ISkin sections from psoriatic patients and healthy controls were immune‐stained for TLR7 together with a marker of the early endosome (EEA1) or late endosome/lysosome (LAMP1) prior to analysis by confocal microscopy, showing TLR7 localization in different organelles. Scale bar: 50 μm. Source data are available online for this figure.

To further determine the molecular mechanism underlying SHP2‐modulated psoriasis, we identified SHP2‐interacting proteins. Specifically, we overexpressed HA‐tagged SHP2 in THP‐1 cells, immunoprecipitated SHP2 with HA antibody, and performed mass spectrometry. Of the mass spectrometry‐identified SHP2‐interacting proteins, we focused on TLR7 because it has been reported to participate in the development of psoriasis (Kim *et al*, 2018). Next, we performed co‐immunoprecipitation (co‐IP), which confirmed that TLR7 indeed interacted with SHP2 in HEK293T cells overexpressing HA‐tagged SHP2 and GFP‐tagged TLR7 (Fig 7A). The endogenous interaction between TLR7 and SHP2 was augmented in THP‐1 cells (Fig 7B) and BMDMs (Fig 7C) after IMQ stimulation. Agreeing with the co‐IP results, confocal microscopy revealed a significant increase in the co‐localization of TLR7 and SHP2 in THP‐1 cells following IMQ treatment (Fig 7D).

SHP2 possesses two SH2 domains at the N terminus, a PTP domain and a phosphotyrosine‐containing tail. To determine the structural basis of its scaffolding function, we generated a series of SHP2 mutants, with the PTP, SH2, or C‐terminal tail domain deleted. Deletion of the PTP or SH2 domain almost completely abolished the association of SHP2 and TLR7 (Fig 7E), suggesting that the PTP and SH2 domains of SHP2 interact with TLR7.

Next, we interrogated the molecular effect of SHP2 interaction with TLR7. Resiquimod (R848) is a TLR7/8 agonist that induces consistent effects with IMQ (Appendix Fig S18). Analysis of isolated cytoplasm and cell membranes confirmed the increase in TLR7 on the cell membranes when SHP2 was overexpressed (Fig 7F). Simultaneously, we observed an accumulation of TLR7 on the cell membrane, which was more obvious in cells overexpressing SHP2 and eliminated when SHP2 was knocked down (Appendix Fig S19). Collectively, these data suggested that SHP2 promotes TLR7 localization to the cell membrane.

To obtain further details on the TLR7 membrane localization, we isolated the endoplasmic reticulum (ER), Golgi, endosomes, and plasma membranes from THP‐1 cells. SHP2 overexpression caused an increased level of TLR7 only in the endosomes but not in the ER or Golgi (Fig 7G). Conversely, in SHP2‐deficient cells, TLR7 decreased in the endosomes (Fig 7H). Detection of subcellular localization of TLR7 in the human skin revealed comparable levels of TLR7 in the ER and Golgi (marked by calreticulin and giantin, respectively) between psoriatic patients and normal controls (Fig 7I and Appendix Fig S20). By contrast, TLR7 was significantly increased in the endosomes of the skin from psoriatic patients, where it was co‐located with EEA1 and LAMP1 (Fig 7I). SHP099 also significantly decreased TLR7 in the endosomes, where it co‐localized with EEA1 and LAMP1 (Appendix Fig S21). Therefore, the data in Fig 7 indicated that SHP2 promotes the trafficking of TLR7 to the endosomes, particularly in the context of psoriasis.

### SHP2 enhanced activation of TLR7/NF‐*κ*B signaling in a phosphatase‐dependent manner

**Figure 8:**
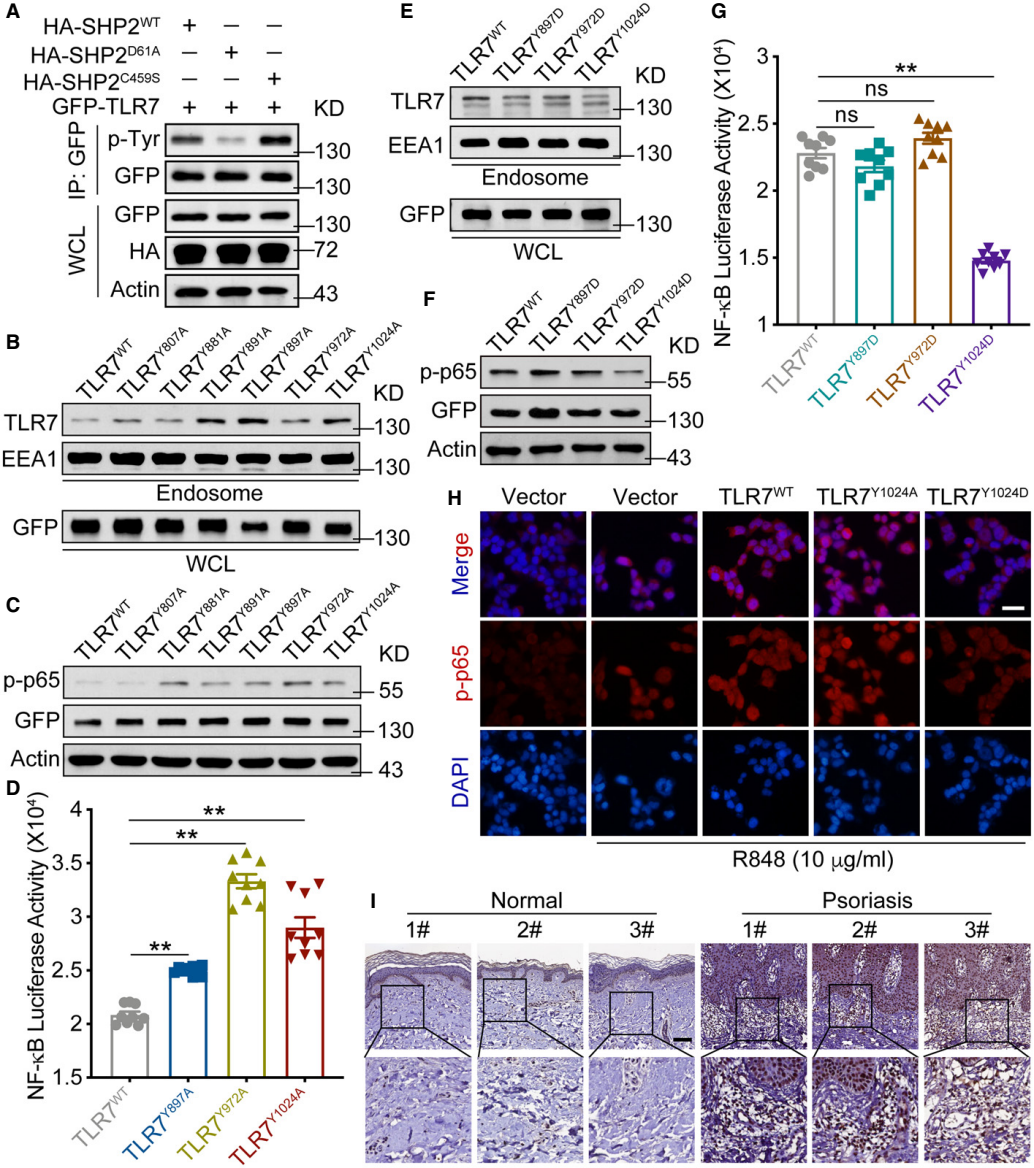
SHP2 enhanced activation of TLR7/NF‐*κ*B signaling in a phosphatase‐dependent manner AImmunoblotting of HEK293T cells co‐transfected for 48 h with GFP‐TLR7, plus HA‐tagged SHP2 or SHP2 mutant vectors, followed by immunoprecipitation with anti‐GFP.B, EImmunoblotting of TLR7 in the endosome isolated from HEK293T cells that were transfected with GFP‐TLR7 or GFP‐TLR7 mutant vectors.C, FImmunoblotting of p‐p65 in lysates of HEK293T cells transfected with GFP‐TLR7 and TLR7 mutant vectors and then treated with IMQ (10 µg/ml) for 30 min.D, GHEK293T cells transfected with NF‐*κ*B‐luciferase reporter and indicated TLR7 mutant vectors and then treated with IMQ (10 µg/ml) for 24 h were harvested for luciferase assay.HImmunofluorescence staining of HEK293T cells transfected with GFP‐TLR7 and TLR7 mutant vectors, prior to treatment of R848 (10 µg/ml) for 30 min and labeled with specific antibodies. Scale bar: 50 μm.IRepresentative p‐TLR7 Y1024 staining of skin sections from human. Scale bar: 100 μm. Data information: Data are represented as mean ± SEM. *P* values are determined by two‐tailed unpaired Student’s *t*‐test (D, G). **P < 0.01, ns, not significant. Source data are available online for this figure.

Given that SHP2 is a tyrosine phosphatase, we queried whether SHP2 dephosphorylated tyrosine residues on TLR7. In HEK293T cells co‐transfected with GFP‐TLR7 and HA‐SHP2 (wild‐type, WT) vectors and then stimulated by IMQ, we detected phosphor‐tyrosine (p‐Tyr) in TLR7 pulled down by anti‐GFP (Fig 8A, left lane). When the WT SHP2 was replaced with a gain‐of‐function mutant (SHP2^D61A^), the p‐Tyr in TLR7 was inhibited (Fig 8A, middle lane); however, the loss‐of‐function mutant SHP2^C459S^ intensified the p‐Tyr in TLR7 (Fig 8A, right lane).

TLR7 has two predicted tyrosine residues (Y807 and Y881) in the linker region (http://www.phosphosite.org/) and four tyrosine residues (Y891, Y897, Y972, and Y1024) in the TIR domain. We thus constructed mutant plasmids of TLR7 by replacing tyrosine with alanine and used them to transfect HEK293T cells with these plasmids. Western blots of endosomal proteins from HEK293T cells transfected with GFP‐TLR7 or mutant vectors revealed that several mutations, including Y891A, Y897A, Y972A, and Y1024A, increased TLR7 expression relative to cells expressing wild‐type TLR7 (Fig 8B). Immunoblotting assays showed that compared to wild‐type TLR7, Y897A, Y972A, and Y1024A mutants enhanced TLR7 responses with p‐p65 as a proxy (Fig 8C). NF‐*κ*B activation, assessed using a luciferase assay, also increased in the HEK293T cells overexpressing Y897A, Y972A, and Y1024A mutants of TLR7 (Fig 8D).

To further explore the effects of TLR7 phosphorylation on its downstream signaling, we constructed another set of mutant plasmids by replacing these tyrosine residues with aspartic acid to mimic lasting phosphorylation. Compared to HEK293T cells transfected with wild‐type TLR7 vector, those transfected with respective GFP‐TLR7 mutants expressed lower levels of TLR7, which was most obvious with Y1024D (Fig 8E). Regarding p65 phosphorylation and NF‐*κ*B activation, only Y1024D mutation caused marked attenuation (Fig 8F–H).

To detect the phosphorylation of TLR7 at Y1024, we used an antibody specifically against phosphor‐TLR7 Y1024 and found that the level of Y1024 phosphorylation significantly increased in skin biopsies from psoriatic patients compared to normal controls (Fig 8I), suggesting that TLR7 phosphorylation at Y1024 plays a role in psoriasis development.

**Figure EV3:**
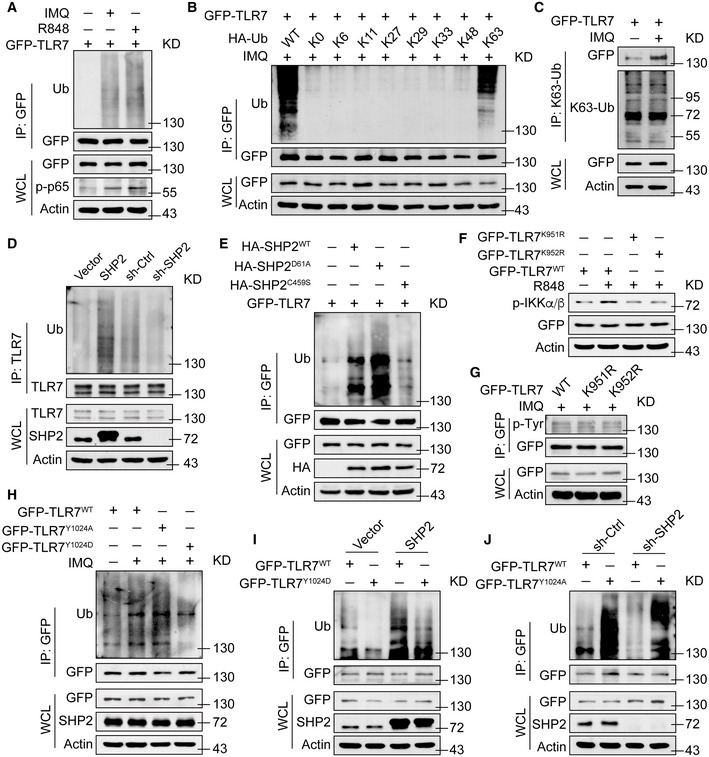
SHP2 mediated the ubiquitination of TLR7 via dephosphorylating its Y1024 Immunoblotting of TLR7 ubiquitination in HEK293T cells transfected with GFP‐TLR7 for 48 h, then treated with IMQ (10 µg/ml) or R848 (10 µg/ml) for 1 h.Immunoblotting of TLR7 ubiquitination in HEK293T cells co‐transfected with GFP‐TLR7, along with HA‐Ub or mutant Ubs and treated with IMQ (10 µg/ml) for 1 h.Immunoblotting of HEK293T cells transfected with GFP‐TLR7 and treated with or without IMQ (10 µg/ml) for 30 min.Immunoblotting of TLR7 ubiquitination in THP1 cells with vector, SHP2, shRNA‐Control, or shRNA‐SHP2 lentivirus was treated with IMQ (10 µg/ml) for 1 h.HEK293T cells co‐transfected with GFP‐TLR7 and HA‐SHP2 and HA‐SHP2 mutant vectors for 48 h, and then infected with IMQ (10 µg/ml) for 1 h.Immunoblotting of HEK293T cells transfected with GFP‐TLR7 and TLR7 mutant vectors and treated with or without R848 (10 µg/ml) for 30 min.Immunoblotting of HEK293T cells transfected with GFP‐TLR7 and TLR7 mutant vectors and treated with IMQ (10 µg/ml) for 30 min.Immunoblotting of HEK293T cells transfected with GFP‐TLR7 and TLR7 mutant vectors and treated with or without IMQ (10 µg/ml).Immunoblotting of TLR7 ubiquitination in HEK293T cells with vector and SHP2 lentivirus transfected with GFP‐TLR7 and GFP‐TLR7^Y1024D^ plasmids and infected with IMQ (10 µg/ml) for 1 h.Immunoblotting of HEK293T cells with shRNA‐Control or shRNA‐SHP2 lentivirus transfected with GFP‐TLR7 and GFP‐TLR7^Y1024A^ plasmids and infected with IMQ (10 µg/ml) for 1 h. Source data are available online for this figure.

We next explored why the phosphorylation of TLR7 affected downstream NF‐*κ*B activation. As shown in Fig 7, SHP2 promotes the trafficking of TLR7 from the Golgi to endosomes, and ubiquitination plays a significant role in this trafficking. Therefore, we first demonstrated that TLR7 was ubiquitinated following stimulation by TLR7 agonists (e.g., IMQ and R848; Fig EV3A). To investigate which form of the polyubiquitin chain of TLR7 was regulated by SHP2, we co‐transfected GFP‐TLR7 plasmid and HA‐Ub or mutant Ubs and then carried out immunoprecipitation with anti‐GFP (Fig EV3B), or only transfected GFP‐TLR7 plasmid and then used an antibody to K63‐linked ubiquitin (Fig EV3C). The data showed that TLR7 polyubiquitination was K63 linked. Importantly, SHP2 also positively regulated TLR7 ubiquitination, since the ubiquitination level of TLR7 increased when SHP2 was overexpressed (Fig EV3D), especially in an active form (Fig EV3E). Next, we substituted two corresponding lysine residues in TLR7 (K951R and K952R) (Chiang *et al*, 2012), both of which reduced the phosphorylation levels of IKK*α*/*β* (Fig EV3F), without affecting TLR7 phosphorylation (Fig EV3G). We then evaluated the effect of SHP2 on TLR7 ubiquitination, specifically through Y1024. In cells transfected with the TLR7^Y1024A^ mutant mimicking dephosphorylation, the ubiquitination of TLR7 increased, as with the effect of IMQ. However, in cells expressing the phospho‐mimicking TLR7^Y1024D^, TLR7 was less ubiquitinated (Fig EV3H). Furthermore, ubiquitination of wild‐type TLR7 was increased by SHP2 overexpression to a higher level than when cells were transfected with TLR7^Y1024D^ (Fig EV3I). In line with these results, SHP2 knockdown using shRNA decreased the ubiquitination of wild‐type TLR7, without affecting that of TLR7^Y1024A^ (Fig EV3H). Similarly, for SHP2‐deficient HEK293T cells transfected with TLR7^Y1024A^, TLR7 ubiquitination was almost unchanged (Fig EV3J). Collectively, these lines of evidence suggest that SHP2 regulates the function of TLR7, specifically through dephosphorylation of TLR7, promoting its ubiquitination and trafficking to endosomes.

### The *Tlr7*‐Y1025D mutant alleviated the psoriasis‐like phenotype in the IMQ‐induced murine model

**Figure 9:**
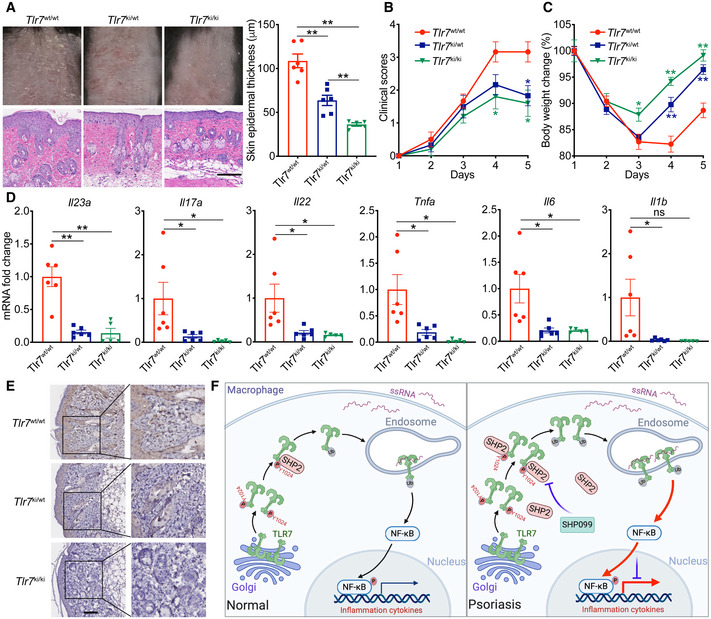
The *Tlr7*‐Y1025D mutant alleviated the psoriasis‐like phenotype in the IMQ‐induced murine model *Tlr7* ^wt/wt^ female mice (*n* = 6) and *Tlr7*‐Y1025D mutant *Tlr7* ^ki/wt^ female mice (*n* = 6) and *Tlr7* ^ki/ki^ female mice (*n* = 5) were treated with indicated dose of IMQ for 4 days. Phenotypic presentation (*top*) and H&E staining (*bottom*) of dorsal skin. Scale bar: 100 μm. Left: H&E staining data; right: statistical data (mean ± SEM).Clinical scores plotted with mean ± SEM. *Denotes statistical significance when compared with the *Tlr7* ^wt/wt^ group.Body weight change plotted with mean ± SEM. *Denotes statistical significance when compared with the *Tlr7* ^wt/wt^ group.Quantitative PCR analysis of mRNA encoding IL‐23/IL‐17A axis cytokines and other psoriasis‐related cytokines in the dorsal skin. Results were normalized to *Gapdh* expression.Representative p‐p65 staining of the dorsal skin. Scale bars: 100 μm.The graphic illustration of the mechanism of SHP2 accelerating the development of psoriasis. In macrophages of normal skin, when stimulating by TLR7 agonist ssRNA (e.g., IMQ, R848), TLR7 is phosphorylated and SHP2 is recruited to dephosphorylate TLR7, then TLR7 is ubiquitinated, and next activates downstream NF‐*κ*B signaling. However, in infiltrated macrophages of psoriatic skin, SHP2 is overexpressed. Hyper‐dephosphorylation of TLR7 at Tyr1024 by SHP2 leads to hyper‐ubiquitination of TLR7 and overactivation of NF‐*κ*B activation, resulting in unrestrained skin inflammation. Created in BioRender.com. Data information: Data are represented as mean ± SEM. *P* values are determined by two‐tailed Mann–Whitney *U* test (A, D) or Tukey multiple‐comparison test (B, C). *P < 0.05, **P < 0.01, ns, not significant. Source data are available online for this figure.

**Figure EV4:**
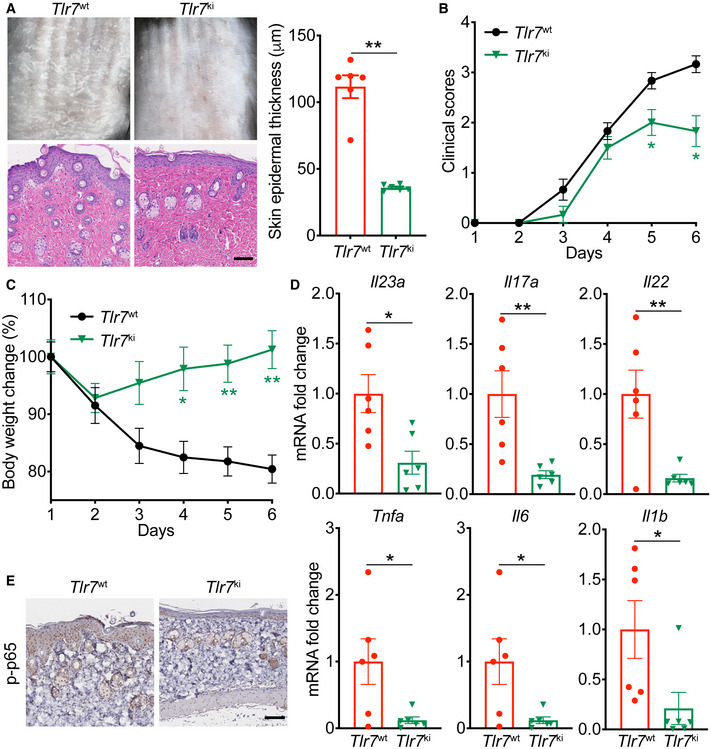
The IMQ‐induced psoriasis‐like phenotype was improved in *Tlr7* ^ki^ mice *Tlr7* ^wt^ male mice (*n* = 6) and *Tlr7*‐Y1025D mutant *Tlr7* ^ki^ male mice (*n* = 6) were treated with indicated dose of IMQ for 5 days. Phenotypic presentation (*top*) and H&E staining (*bottom*) of dorsal skin. Scale bar: 100 μm. Left: H&E staining data; right: statistical data (mean ± SEM).Clinical scores plotted with mean ± SEM. * Denotes statistical significance when compared with the *Tlr7* ^wt^ group.Body weight change plotted with mean ± SEM. * Denotes statistical significance when compared with the *Tlr7* ^wt^ group.Quantitative PCR analysis of mRNA encoding IL‐23/IL‐17A axis cytokines and other psoriasis‐related cytokines in the dorsal skin. Results were normalized to *Gapdh* expression.Representative p‐p65 staining of the dorsal skin. Scale bars: 100 μm. Data information: Data are represented as mean ± SEM. *P*‐values are determined by two‐tailed unpaired Student’s *t*‐test (A, D) or Bonferroni multiple‐comparison test (B, C). *P < 0.05, **P < 0.01. Source data are available online for this figure.

**Figure EV5:**
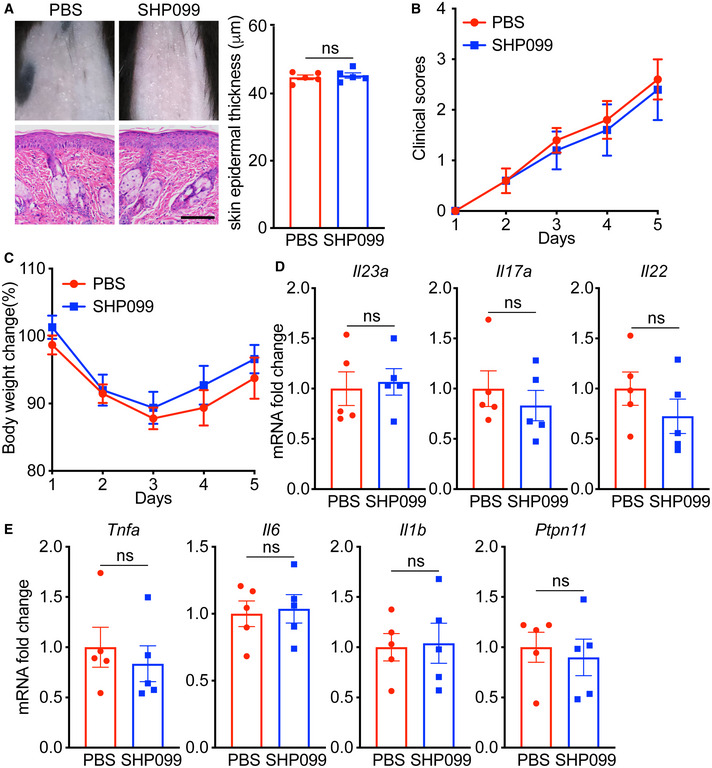
SHP099 provided no benefit in the *Tlr7*‐Y1025D mice treated with IMQ *Tlr7*‐Y1025D mutant *Tlr7* ^ki^ male mice (*n* = 5/group) were treated with 10 mg/kg SHP099 or vehicle for 4 days. APhenotypic presentation (*top*) and H&E staining (*bottom*) of dorsal skin. Scale bar: 100 μm. Left: H&E staining data; right: statistical data (mean ± SEM).BClinical scores plotted with mean ± SEM.CBody weight change plotted with mean ± SEM.D, EQuantitative PCR analysis of mRNA encoding IL‐23/IL‐17A axis cytokines (D) and other psoriasis‐related cytokines (E) in the dorsal skin. Results were normalized to *Gapdh* expression. Data information: Data are represented as mean ± SEM. *P* values are determined by two‐tailed Student’s *t*‐test (A, D, E) or Bonferroni multiple‐comparison test (B, C). ns, not significant. Source data are available online for this figure.

To further confirm the function of phosphor‐TLR7 Y1024, given that human *TLR7*‐Y1024 corresponds to mouse *Tlr7*‐Y1025, we generated *Tlr7*‐Y1025D site‐mutant mice and treated their dorsal skins with IMQ. The psoriasis‐like phenotype, including the skin’s epidermal thickness and infiltration by inflammatory cells (Fig 9A), clinical score (Fig 9B), and body weight (Fig 9C), significantly improved in the *Tlr7*‐Y1025D mutant female mice (*Tlr7* ^ki/wt^ mice and *Tlr7* ^ki/ki^ mice) compared with the wild‐type mice. Also, the expression of IL‐23/IL‐17 axis‐related genes, such as *Il23a*, *Il17a*, and *Il22*, and other pro‐inflammatory cytokines, such as *Tnfa*, *Il6*, and *Il1b*, in the skin decreased markedly in the IMQ‐treated *Tlr7*‐Y1025D mutant mice (Fig 9D). Consistent with Fig 8, *Tlr7*‐Y1025D mutant mouse, skin lesions also revealed a significantly lower level of p‐p65 protein in the infiltrated cells (Fig 9E). The IMQ‐induced psoriasis‐like phenotype was also ameliorated in the *Tlr7* ^ki^ male mice (Fig EV4). In the *Tlr7* ^ki^ male mouse, IL23‐induced psoriasis‐like skin inflammation was improved to some extent. Total ear thickness decreased in *Tlr7* ^ki^ mice, but psoriasis‐related inflammatory cytokines were marginally affected (Appendix Fig 22). Moreover, SHP099 provided no benefit for the TLR7‐Y1025D mice treated with IMQ (Fig EV5). Collectively, these results demonstrated that phosphor‐TLR7 Y1024 plays an important role in the progression of psoriasis.

Collectively, our data demonstrated a positive regulation of psoriasis by SHP2. By dephosphorylating TLR7 at Tyr1024 and inducing TLR7 ubiquitination, SHP2 drove the trafficking of TLR7 to the endosomes, which sustained the activation of downstream TLR7/NF‐*κ*B signaling, thereby promoting the development of psoriasis (Fig 9F).

## Discussion

Accumulating evidence has revealed that SHP2 is closely related to autoimmune disease (Wang *et al*, 2016). In this study, scRNA‐seq of skin tissues demonstrated that the SHP2 allosteric inhibitor SHP099 impaired skin inflammation in myeloid cells, including macrophages, dendritic cells, and neutrophils. We found that SHP2 expression greatly increased in the infiltrated macrophages of psoriatic skin. Consequently, SHP2‐mediated TLR7/NF‐*κ*B activation was augmented, leading to further increases in the expressions of psoriasis‐related inflammatory cytokines, including IL‐23A, TNF‐*α*, IL‐6, and IL‐1*β*, thus accelerating psoriasis‐like skin inflammation. Dendritic cells play an important role in both the initiation (Wang & Bai, 2020) and developmental stages (Ganguly *et al*, 2009; Brunner *et al*, 2013; Riol‐Blanco *et al*, 2014) of psoriasis; however, the conditional knockdown of SHP2 in dendritic cells did not ameliorate psoriasis‐like skin inflammation in mice, or in γδTh17 cells (Kadekar *et al*, 2020). Interestingly, SHP2 deficiency in myeloid cell lineages (monocytes, mature macrophages, and granulocytes) or the administration of an allosteric inhibitor of SHP2 ameliorated psoriasis‐like disease in mice, suggesting that SHP2 promotes psoriasis progression with cell specificity. Oxidative stress, granule composition, and neutrophil extracellular traps of neutrophils are associated with the initial and maintenance phases of psoriasis (Lin *et al*, 2011; Katayama, 2018; Chiang *et al*, 2019; Jiang *et al*, 2019; Wang & Jin, 2020; Wójcik *et al*, 2020), and the role of SHP2 in these phenomena needs to be further investigated. Together, our results demonstrated that SHP2 is a critical regulator of psoriatic development and suggested SHP2 as a potential therapeutic target for the treatment of psoriasis.

Both the innate and adaptive immune systems have been considered important drivers of psoriasis. Recently, the IL‐23/IL‐17 axis has been shown to play a pivotal role in psoriasis (Boehncke & Schön, 2015; Kopp *et al*, 2015; Burkett & Kuchroo, 2016; Lebwohl, 2019), and several negative regulators of this axis have been identified, including adaptor proteins and microRNAs. Mechanisms mediated by these regulators in T cells, macrophages, and keratinocytes have been shown to contribute to the development of psoriasis (Boisson *et al*, 2013; Yan *et al*, 2015; Srivastava *et al*, 2017; Bambouskova *et al*, 2018; Wang *et al*, 2018; Wu *et al*, 2018; Xu *et al*, 2018). ACT1 is a critical adaptor protein in the IL‐17 signaling pathway. A missense single‐nucleotide polymorphism (rs33980500; SNP‐D10N) in the coding region of ACT1 was suggested to increase the compensatory Th17 cells, with excessive secretion of IL‐22 and IL‐17 being the main cause of psoriasis susceptibility in ACT1‐D10N patients (Boisson *et al*, 2013). Another negative regulator is microRNA (miR)‐146a, a deficiency of which contributes to IL‐17‐driven inflammation in keratinocytes, early disease onset, and aggravated skin inflammation in IMQ‐induced models (Srivastava *et al*, 2017). However, several other molecules, for example, microRNA‐210 (Wu *et al*, 2018) and I*κ*B*ζ* (Bambouskova *et al*, 2018), IL‐25 (Xu *et al*, 2018), Card14 (Wang *et al*, 2018), and NF‐*κ*B (Yan *et al*, 2015) have been identified as positive regulators of psoriasis by regulating various processes, including Th17 cell differentiation, IL‐17‐driven inflammation, and the proliferation of keratinocytes (Bambouskova *et al*, 2018, 3; Wang *et al*, 2018, 14; Wu *et al*, 2018; Xu *et al*, 2018; Yan *et al*, 2015). Also, antimicrobial peptide LL37 has been shown to enable keratinocytes to produce IFN‐*β*, which promotes the maturation of dendritic cells and contributes to the pathogenesis of psoriasis (Zhang *et al*, 2016). The extensive findings from these previous studies strongly point to a central role of NF‐*κ*B activation and IL‐23/IL‐17 signaling in psoriasis. In this study, our data showed that, in the infiltrated macrophages of psoriatic skin, SHP2 was strongly induced and acted as a positive regulator of psoriasis. Via dephosphorylation of TLR7 specifically at Tyr1024, SHP2 triggered a sequela of molecular events, including (i) ubiquitination of TLR7, (ii) the trafficking of TLR7 to the endosomes, and (iii) excessive activation of TLR7/NF‐*κ*B signaling, fueling the uncontrolled inflammation. Ablation or inhibition of SHP2 in macrophages reduced the production of pro‐inflammatory cytokines and prevented the development of psoriasis‐like skin inflammation in both the IMQ‐induced murine model and psoriatic patients. Taken together, our findings connecting SHP2 and TLR7 highlighted the importance of SHP2 as a positive regulator of inflammation in psoriasis and probably in other autoimmune diseases.

TLRs are essential players in innate and adaptive immunity. Intracellular TLRs, such as TLR3, TLR7, TLR8, and TLR9, are intrinsically capable of recognizing nucleic acids and inducing immune responses (Petes *et al*, 2017). In particular, TLR7 recognizes ssRNA and acts via MyD88 to activate two major signaling pathways, including NF‐*κ*B and IFN regulatory factors (IRFs), thus triggering the transcription of genes that encode pro‐inflammatory cytokines and the production of type I IFNs (Hu *et al*, 2016). Previous studies have shown that TLR7 activation leads to upregulation of pro‐inflammatory cytokines, accelerating the development of psoriasis (Kim *et al*, 2018). TLR7 overexpression, as a result of TLR8 deficiency in DCs, leads to augmented NF‐*κ*B activation in response to TLR7 ligands and thereby aggravates the development of spontaneous autoimmunity (Demaria *et al*, 2010, 8). Interestingly, *Datura metel* L.—a traditional Chinese medicine known to suppress the progression of IMQ‐induced psoriasis in a the mouse model—can suppress TLR7 and TLR8 expression and inhibit the activity of the TLR7/8‐MyD88‐NF‐*κ*B‐NLRP3 inflammasome pathway (Yang *et al*, 2019). Our data demonstrated that SHP2 can promote the localization of TLR7 to the endosome. SHP2 overexpression or overactivation (e.g., as observed in IMQ or psoriasis) maintains excessive activation of TLR7/NF‐*κ*B signaling and accelerates psoriasis‐like skin inflammation. Mechanistically, we found that SHP2 drove TLR7 translocation in a phosphatase‐dependent manner, specifically by dephosphorylating TLR7 at Tyr1024. Although several TLRs including TLR2, TLR3, TLR4, TLR5, TLR8, and TLR9 can be tyrosine phosphorylated upon stimulation in the cytoplasmic TIR domain (Chattopadhyay & Sen, 2014), it is not known whether and how TLR7 can be (de)phosphorylated in the tyrosine residues. While a previous study showed that SHP2 inhibited TLR3‐activated but not TLR2‐, TLR7‐, and TLR9‐activated pro‐inflammatory IL‐6 and TNF‐*α* production (An *et al*, 2006), there seemed to be a discrepancy between the previous study and our data, which may have related to the experimental system. We used peritoneal macrophages from conditional knockout mice, whereas the previous study took peritoneal macrophages from wild‐type mice and then transfected them with SHP2 siRNA, but the difference was still marked. In this study, we first proved that TLR7 can be phosphorylated at Tyr1024 in the skin lesions of psoriatic patients and that its dephosphorylation by SHP2 accelerates TLR7 ubiquitination and promotes TLR7 activation of NF‐*κ*B signaling, contributing to the aggravation of psoriasis. In psoriatic skin, TLR7 also can be phosphorylated at Tyr1024 in epidermal cells, where TLR7 may be involved in the expansion of inflammation in psoriasis, exacerbating psoriasis by activating the NF‐*κ*B pathway to upregulate the expression of multiple inflammatory factors. It may also bind to antimicrobial peptide LL37 and others to trigger the inflammatory response of immune cells. Or it may be involved in the initiation of psoriasis, which requires extensive experiments to confirm. A previous study demonstrated that TLR9 was also involved in psoriasis pathogenesis (Tanaka *et al*, 2018). Considering that TLR9 is localized to the endosome, SHP2 may also be involved in TLR9‐mediated signaling, but much more experimental proof is needed.

SHP2 is a ubiquitously expressed tyrosine phosphatase encoded by *PTPN11*. It was the first cloned phosphatase containing the SH2 domain (Feng *et al*, 1993). SHP2, as an intracellular signaling molecule responding to various cytokines, growth factors, and other extracellular stimulators, is ubiquitously expressed in various cells of the body and participates in various aspects of cellular physiology including cell proliferation, activation, migration, and differentiation (Tajan *et al*, 2015). We have previously demonstrated that, following treatment of NLRP3 inflammasome stimulators, SHP2 translocates to mitochondria where it interacts with mitochondrial endomembrane protein adenine nucleotide translocase 1 (ANT1) and dephosphorylates ANT1 to maintain mitochondrial homeostasis. This serves as a mechanism to negatively regulate the activation of NLRP3 inflammasomes (Guo *et al*, 2017), leading to SHP2 being regarded as an anti‐inflammatory factor. Other studies have suggested that SHP2 can also act as a negative regulator of inflammation, for example, SHP2 has been identified as a detrimental factor in inflammatory bowel disease (IBD), which disrupts the macrophage response to interleukin 10 (Xiao *et al*, 2019). However, a study performed in the context of systemic lupus erythematosus (SLE) showed that SHP2 increased in PBMCs isolated from patients with the SLE compared to the normal controls. A SHP2 inhibitor (11a‐1) reversed the symptoms of SLE‐associated organ damage, suggesting that SHP2 is a positive regulator of the development of systemic lupus erythematosus (Wang *et al*, 2016). In the current study, we found that SHP2 levels in the PBMCs and skin lesions of psoriatic patients were significantly higher than those in normal controls. Using myeloid cell lineages (monocytes, mature macrophages, and granulocytes)‐specific conditionally SHP2 knockout mice (M‐*Shp2* ^−/−^ mice) or a SHP2‐allosteric inhibitor (SHP099), we demonstrated that SHP2 inhibition attenuated the IMQ‐induced psoriasis‐like phenotype, suggesting that SHP2 is a positive regulator of psoriatic disease progression. Overall, the pro‐ or anti‐inflammatory role of SHP2 in inflammatory diseases is context dependent, involving an intricate balance between various immune cells and numerous chemokines and cytokines. Interestingly, SHP2 deficiency in macrophages attenuates the IMQ‐induced psoriasis‐like phenotype, whereas its deficiency in DCs does not. SHP2 may exert mainly antifungal, rather than anti‐inflammatory, effects in DCs (Deng *et al*, 2015).

In summary, we revealed a novel regulation by SHP2 of TLR7‐mediated NF‐*κ*B signaling activation in macrophages. Such positive regulation by SHP2, through dephosphorylation of TLR7 at Tyr1024, promotes the trafficking of TLR7 to the endosome and maintains excessive activation of TLR7/NF‐*κ*B signaling. Our research has therefore identified SHP2 as a novel driver of psoriasis pathogenesis and suggested SHP2 inhibition as a promising therapeutic approach for the treatment of autoimmune diseases, such as psoriasis.

## Materials and Methods

### Mice

C57BL/6 mice were purchased from GemPharmatech Co. Ltd (Nanjing, China). The myeloid cell lineages (monocytes, mature macrophages, and granulocytes)‐specific SHP2 knockout mice (M‐*Shp2* ^−/−^ mice) were generated by crossing *Shp2* floxed mice with *Lyz2*‐cre transgenic mice (Guo *et al*, 2017). The dendritic cell‐specific SHP2 knockout mice (DC‐*Shp2* ^−/−^ mice) were generated by crossing *Shp2* floxed mice with *Itgax*‐cre transgenic mice (Caton *et al*, 2007; Durai & Murphy, 2016), which were purchased from Jackson Laboratory (JAX: 008068). We purchased B6.129S1‐*Tlr7^tm1Flv^*/J mice (*Tlr7* ^mut/wt^ mice, TLR7 knockout mice) from Jackson Laboratory (JAX: 008380). We generated *Tlr7*‐Y1025D point mutation mice via the CRISPR/Cas9 system. First, one sgRNA targeting the Exon5 of the *Tlr7* gene was constructed and transcribed *in vitro*. The donor vector with the *Tlr7*‐Y1025D fragment was designed and constructed in vitro, and Cas9 mRNA, sgRNA, and the donor were then co‐injected into zygotes. Thereafter, the zygotes were transferred into the oviducts of pseudopregnant ICR females at 0.5 dpc. F0 mice were birthed after 19–21 days of transplantation, and all the offspring of ICR females (F0 mice) were identified using PCR and sequencing of tail DNA. Positive F0 mice, which had copies of the point mutation of Y1025D of the *Tlr7* gene, were genotyped by the methods. Finally, F0 mice were crossed with C57BL/6 mice to produce heterozygous mice. These mice were bred and maintained in specific pathogen‐free conditions at GemPharmatech Co. Ltd (Nanjing, China) and the Experimental Animal Center at Nanjing University. All mice were of the C57BL/6 background strain, and we used age‐ and sex‐matched mice 6‐ to 10‐week‐old. Mice had unlimited access to standard laboratory chow and water under a light–dark cycle of 12 h each in a room at a temperature of 22°C. All animal experiments were carried out according to the NIH Guide for the Care and Use of Laboratory Animals (National Academies Press, 2011) and were approved by the Experimental Animal Care and Use Committee of Nanjing University (IACUC‐2006024). All efforts were made to minimize the number of animals used and their suffering.

### Human specimens

All human studies were approved by the Ethics Institutional Review Board of West China Hospital, Sichuan University. Normal human skin was obtained from patients at West China Hospital who were undergoing elective surgeries in which skin was discarded routinely as part of the procedures. Psoriatic skin biopsies and blood were obtained from psoriatic patients, who were recruited by the Department of Dermatology and Venereology at West China Hospital under protocol 2019‐R‐513. PBMCs were isolated from the peripheral blood of psoriatic patients and normal healthy donors. Informed consent from patients was obtained and the experiments conformed to the principles set out in the WMA Declaration of Helsinki and the Department of Health and Human Services Belmont Report. Appendix Table S1 shows the information of healthy donors and psoriatic patients.

### SHP2 enzyme activity assay

The enzyme activity of SHP2 in PBMCs was measured using the surrogate substrate DiFMUP in a fluorescence assay. The phosphatase reactions were performed in a 96‐well black plate (Corning) at room temperature. The final reaction volume was 100 μl, including 60 mM HEPES, pH 7.2, 75 mM NaCl, 75 mM KCl, 1 mM EDTA, 0.05% P‐20, and 5 mM DTT. The PBMCs of normal controls and psoriatic patients were lysed in Western and IP lysis buffer (Beyotime) supplemented with protease (MCE). Proteins were quantified using the Bradford assay (HyClone‐Pierce) in two groups. Proteins (1 µg) were incubated with PHPS1 (a PTP inhibitor; Sigma‐Aldrich) at 25°C for 30 min, and the surrogate substrate DiFMUP (Invitrogen) was added and incubated at 25°C for 30 min. The reaction was quenched by adding bpV (phen) (Enzo Life Sciences). The fluorescence signal was measured using a microplate reader (Envision) with excitation and emission wavelengths of 340 and 450 nm, respectively. The other group was the same, but without PHPS1. Finally, the subtraction of these two groups indicated the enzyme activity of SHP2 in PBMCs.

### IMQ‐induced psoriasis‐like mouse model

Eight‐ to ten‐week‐old C57BL/6 wild‐type or transgenic mice were used to establishing the IMQ‐induced psoriasis‐like model. The mice were treated with a daily topical dose of 62.5 mg of IMQ cream (5%, Aldara; 3 M Pharmaceuticals) on the shaved dorsal skin for 4 consecutive days. Mice were sacrificed 1 day after the final treatment. The severity of disease was measured using the clinical Psoriasis Area and Severity Index, which was scored on a scale from 0 to 4: 0, none; 1, slight; 2, moderate; 3, marked; and 4, very marked (Shao *et al*, 2016). For each mouse, skin lesions were taken for hematoxylin and eosin (H&E) staining, immunocytochemistry, immunofluorescence, and qPCR analysis. Serum was also collected for cytokine assays with an ELISA kit.

### IL‐23‐induced psoriasis‐like mouse model

An IL‐23‐mediated mouse model of psoriasis was created as previously described (Rizzo *et al*, 2011; Zhu *et al*, 2017; Wu *et al*, 2018; Xu *et al*, 2018). Generally, mouse ears were injected intradermally with 1 μg recombinant mouse IL‐23 (IL‐23; R&D Systems, #1887‐ML‐010) dissolved in 25 μl PBS/0.1% BSA into one ear and 25 μl PBS/0.1% BSA into the contralateral ear for 4 days. Ear thickness was measured daily.

### Histological analysis

Harvested mouse dorsal skin and human skin tissues were flushed with PBS, fixed with 4% formaldehyde overnight, and embedded in paraffin. Sections (5 μm thick) were stained with hematoxylin and eosin (H&E) according to standard procedures.

For immunohistochemistry, the human and mouse skin paraffin sections were deparaffinized, rehydrated, and antibody retrieved with sodium citrate, blocked, then stained with anti‐SHP2 (Santa Cruz, catalog sc‐7384), anti‐ERK (Cell Signal Technology, catalog 4695), anti‐CD68 (Cell Signal Technology, catalog 76437), anti‐p‐p65 (Cell Signal Technology, catalog 3033), and anti‐Ki67 (Abcam, catalog ab15580) were used at 1:100 overnight at 4°C. After three rinses of 1×PBS, the slides were detected using the Real Envision Detection kit (GeneTech) according to the manufacturer’s instructions.

For immunofluorescence, paraffin‐embedded human and mouse skin sections were deparaffinized and rehydrated, antigen retrieved with sodium citrate, blocked with 5% goat serum, and incubated with primary Abs overnight at 4°C. Anti‐SHP2 (Santa Cruz, catalog sc‐7384), anti‐CD68 (Cell Signal Technology, catalog 76437), anti‐p‐p65 (Cell Signal Technology, catalog 3033), anti‐TLR7 (Novus, catalog NBP2‐24906), anti‐EEA1 (Santa Cruz, catalog sc‐137130), anti‐LAMP1 (Santa Cruz, catalog sc‐20011), anti‐Rab11a (Santa Cruz, catalog sc‐166912), anti‐LAT1 (Santa Cruz, catalog sc‐53550), anti‐Calreticulin (Abcam, catalog ab92516), anti‐Giantin (Abcam, catalog ab80864), and anti‐TLR7 (Santa Cruz, catalog sc‐57463) were used in 1:100. After three rinses of 1× PBST, sections were treated with Alexa Fluor 633‐conjugated donkey anti‐goat IgG (H + L) cross‐adsorbed secondary Ab (1:500; Invitrogen, catalog A‐21082), Alexa Fluor 488‐conjugated goat anti‐rabbit IgG (H + L) cross‐adsorbed secondary Ab (1:500; Invitrogen, catalog A‐11034), Alexa Fluor 488‐conjugated goat anti‐mouse IgG (H + L) cross‐adsorbed secondary Ab (1:500; Invitrogen, catalog A‐32723), Alexa Fluor 546‐conjugated goat anti‐mouse IgG (H + L) cross‐adsorbed secondary Ab (1:500; Invitrogen, catalog A‐11003), and Alexa Fluor 594‐conjugated goat anti‐rabbit IgG (H + L) cross‐adsorbed secondary Ab (1:500; Invitrogen, catalog A‐11037) at room temperature for 2 h in the dark, and the nuclei were stained with DAPI. All the cells were imaged by an inverted confocal microscope (Carl Zeiss).

### Immunocytochemistry

PMA (Sigma‐Aldrich)‐differentiated THP‐1 cells or PMs were incubated in 24‐well culture dishes at a density of 1 × 10^5^ cells per well. Cells were washed by PBS and then fixed by 4% paraformaldehyde for 10 min at room temperature. Following three rinses in 1×PBS, cells were permeabilized using 0.5% Triton X‐100 (Beyotime) for 30 min at 4°C. After blocking cells with 5% BSA for 1 h, cells were cultured with primary Abs overnight at 4°C. Anti‐TLR7 (Novus, catalog NBP2‐24906), anti‐SHP2 (Santa Cruz, catalog sc‐7384), anti‐EEA1 (Santa Cruz, catalog sc‐137130), anti‐LAMP1 (Santa Cruz, catalog sc‐20011) and anti‐EEA1 (R&D systems, catalog AF‐8047) were used at 1:100. After three rinses of 1×PBST, coverslips were treated with Alexa Fluor 633‐conjugated donkey anti‐goat IgG (H + L) cross‐adsorbed secondary Ab (1:500; Invitrogen, catalog A‐21082), Alexa Fluor 488‐conjugated goat anti‐rabbit IgG (H + L) cross‐adsorbed secondary Ab (1:500; Invitrogen, catalog A‐11034), Alexa Fluor 488‐conjugated goat anti‐mouse IgG (H + L) cross‐adsorbed secondary Ab (1:500; Invitrogen, catalog A‐32723), Alexa Fluor 546‐conjugated goat anti‐mouse IgG (H + L) cross‐adsorbed secondary Ab (1:500; Invitrogen, catalog A‐11003), and Alexa Fluor 594‐conjugated goat anti‐rabbit IgG (H + L) cross‐adsorbed secondary Ab (1:500; Invitrogen, catalog A‐11037) at room temperature for 2 h in the dark, and the nuclei were stained with DAPI. All the cells were imaged by an inverted confocal microscope (Carl Zeiss).

### Single cell dissociation from mouse and human skin

Three groups of mice were sacrificed, and the dorsal skin was cut quickly, then placed in a cell culture dish containing cold DPBS, cut into mungbean size, washed three times with cold DPBS, and transferred to a C tube (Miltenyi). Collagenase I (Sigma), collagenase II (Sigma), and Dispase^®^ (Sigma) were added, and the corresponding program was selected to start digestion. After the procedure ended, the cells were observed under a microscope with a hemocytometer. All the liquid and tissue were poured into a 15 ml centrifuge tube, the C tube washed with DMEM, then transferred to the same 15 ml tube, centrifuged at 4°C, 350 g for 7 min, and the supernatant aspirated with a Pasteur tube. Trypsin (1–2 ml) was added, and the cell pellet was blown evenly, then placed at room temperature for 3–5 min. Five milliliter DMEM containing 10% FBS was added after sieving through a 70 µm sieve (Miltenyi) in a new 15 ml tube, all the tissue and liquid were transferred to the sieve, and 5 ml DMEM was added to rinse the screen. The 15 ml tube was centrifuged at 4°C, 350 *g*, for 7 min, and the supernatant removed. If the pellet contained any red, 1 ml of 1× cleavage reagent was added to it, resuspended, and mixed, then placed on ice for 2–3 min. It was supplemented with 5 ml DMEM containing with 10% FBS to stop cleavage; centrifuged at 4°C, 350 *g*, for 7 min; washed with 7 ml DMEM containing 10% FBS; and resuspended according to the size of the pellet.

Human skin sections were enzymatically digested with Whole Skin Dissociation kit (Miltenyi Biotec) according to manufacturer’s instruction. After dissociation, supernatant was removed and the pelleted cells were subsequently lysed red blood cells.

### cDNA library construction and single‐cell RNA‐seq

A single skin cell was captured in the 10× Genomics Chromium Single Cell 3′ Solution, and RNA‐seq libraries were prepared following the manufacturer’s protocol (10× Genomics). The libraries were subjected to high‐throughput sequencing on the Novaseq6000 platform, and 150‐bp paired‐end reads were generated. The scRNA‐seq data from mice skin and human skin have been deposited in the GEO database under accession number GSE165021.

### Process and quality control of the single‐cell RNA‐seq data

Single‐cell RNA‐seq data for each experiment were processed with cellranger count (10×Genomics CellRanger (v3.1.0)) based on the mouse reference genome GRCm38 (mm10). Digital gene expression matrices were analyzed in R (v3.6.0), using the Seurat (v3.2.0) package (Butler *et al*, 2018). Cells were filtered by the number of UMIs (< 100,000 UMIs), the number of genes (< 6,500 genes), and percentage of mitochondrial genes (“percent.mt” lower than 10%), yielding a total of 16,499 cells for Sham mouse, 17,425 cells for IMQ‐induced mouse, and 15,669 cells for IMQ‐induced and treated with SHP099 mouse, respectively (Supplemental Data). Normalization was performed with the *SCTransform* (Hafemeister & Satija, 2019) function with regression of percentage of mitochondrial genes. For integration, 3,000 shared highly variable genes were identified using the *SelectIntegrationFeatures* function. Integration anchors were identified based on these genes using the *FindIntegrationAnchors* (Stuart *et al*, 2019) function with an “SCT” normalization method. The data were then integrated using the *IntegrateData* function. Principal component analysis (PCA) and uniform manifold approximation and projection (UMAP) dimension reduction with the top 30 principal components were performed. A nearest‐neighbor graph using the 30 dimensions of the PCA reduction was calculated using *FindNeighbors*, followed by clustering using *FindClusters* with a resolution of 0.8. Candidate marker genes for each cell cluster were identified by the *FindAllMarkers* function. For each cluster of cells, group‐specific differentially expressed genes were identified using the Wilcoxon rank sum test as implemented in *FindAllMarkers*.

### Annotating cell clusters

For each cell cluster identified as above, a cell type was assigned to it by using a combination of differentially expressed genes and known gene signatures. The *FeaturePlot* function showed that the cluster‐specific marker genes *Pecam1*, *Col1a1*, *Krt5*, *Cd3d*, *Lyz2*, and *Plp1* were significantly enriched in endothelial cells, fibroblasts, keratinocytes, lymphocytes, myeloid cells, and Schwann cells, respectively. The high‐density bar charts of cluster‐specific genes were estimated using *VlnPlot*, with the following parameters: *pt.size* = 0, *stack* = T.

### Analysis of inflammation‐associated genes

We constructed a set of inflammation‐associated genes: *Ifng*, *Ifngr1*, *Ifngr2*, *Ifnb1*, *Il10*, *Il12a*, *Il12b*, *Il12rb1*, *Il12rb2*, *Il17a*, *Il17f*, *Il17ra*, *Il18*, *Il18r1*, *Il18rap*, *Il1a*, *Il1b*, *Il2*, *Il21*, *Il22*, *Il23a*, *Il23r*, *Il2rg*, *Il4*, *Il4r*, *Il6*, *Il26*, *Tyk2*, *Jak2*, *Jun*, *Mapk14*, *Nfkb1*, *Rorc*, *S100a8*, *S100a9*, *Stat1*, *Stat3*, *Stat6*, *Tgfb1*, *Tgfb2*, *Tgfb3*, *Tnf*, *Tnfsf15*, *Mmp9*, *Ccl2*, *Ccl20*, and *Ccr4*. To compare the expression patterns of these inflammation‐associated genes for the six cell types in the three groups (Fig 3F), the mean expression levels for each gene were scaled to relative values ranging from 0 to 1. Scaled normalization data were visualized using *ggboxplot*.

### Enrichment analysis of KEGG pathways

Enrichment analysis of KEGG pathways was performed using the R package clusterProfiler (3.11.1) (Yu *et al*, 2012) with parameters of *PValueCutoff* = 0.05, *PAdjustMethod* = BH, *QValueCutoff* = 0.05, *MingsSize* = 10, and *MaxGSSize* = 500.

### Spatial transcriptomics (ST)

We obtained two skin sections from normal control and one lesion skin from psoriasis patient. All samples were then processed for full ST experiment as per manufacturer’s instructions (10x Genomics) being cut in a pre‐cooled cryostat at 10 μm thickness onto 6.5 × 6.5 mm capture areas, and RNA extracted using the RNeasy Mini kit (QIAGEN), followed by RIN calculation (RIN ≥ 7) using the Pico Reagent kit (Agilent). Tissue optimization experiments were then performed, using fluorescence microscopy imaging, and the infiltration time with the largest fluorescence signal and the smallest signal diffusion was optimal according to the imaging results. After permeabilization, reverse transcription and second strand synthesis are performed on slides, followed by cDNA amplification and quality control, and the pooled libraries are sequenced on Nova‐seq 6000 (Illumina). The spatial transcriptome data from human skin have been deposited to the GEO database with accession number GSE165021.

### Human scRNA‐seq and ST analysis

scRNA‐seq data were aligned to the hg38 genome and processed with CellRanger (version 6.0.2). Then, the matrix was imported into the Seurat package (version 4.0.0) for subsequent analysis. In our cases, we filtered cells with parameters (nFeature_RNA > 200 & nFeature_RNA < 6,000 & nCount_RNA < 25,000 & percent.mito < 10), and finally, 23,648 total cells were remaining (normal1: 14,693, normal2: 8,275, Psoriasis1: 10,529, and Psoriasis2: 4,067). Totally 3,000 highly variable genes were generated for performing principal component analysis (PCA) reduction dimension. To analyze datasets from the same time point, we performed harmony among datasets. Harmony uses PCA to embed the transcriptome expression profile into low‐dimensional space, and then uses an iterative process to remove the specific effects of the dataset. Finally, single‐cell clustering was visualized by t‐distributed stochastic neighbor embedding (t‐SNE), utilizing previous computed harmony embeddings 1–30, and a resolution parameter of 0.3 was used to find clusters. In downstream analysis, cor function was used for calculating correlation coefficient between *PTPN11* and each gene from NF‐*κ*B pathways in four groups (Macrophages in Normal, Macrophages in Psoriasis, Dendritic cells in Normal, and Dendritic cells in Psoriasis), respectively.

Raw ST data from ST and Visium samples were processed using spaceranger (version 1.2.0) and analyzed by Seurat package (version 4.0.3) in R in addition to custom scripts. For each sample, spots were filtered for maximum detected gene count of 50,000 so that Psoriasis spots were filtered to 2,971 and the Normal spots all were retained. Normalization across spots was performed with the SCTransform function with regression of replicate and number of genes per spot. Dimensionality reduction and clustering were performed with principal component analysis (PCA) with the first 30 PCs. For merging data, the two samples were merged with the merge Seurat function and re‐normalized with SCTransform (regressing replicate and number of genes per spot) prior to PCA and UMAP on the first 30 PCs. Between the two samples with overlapping clusters, we employed canonical correlation analysis (CCA) to align the ST data by Anchors found by FindIntegrationAnchors function. Six clusters were found by performing FindClusters with 0.1 resolution. Cluster1 and Cluster3 were considered as corium regions for the high expression of inflammatory gene. SPOTlight (Elosua‐Bayes *et al*, 2021) was employed to integrate single‐cell RNA sequencing and ST datasets by NMF regression and find the optimal weighted combinations of cell types to explain a spot's cellular composition. Spots in Cluster1 and Cluster3 were selected to show cellular composition by function spatial_scatterpie. Cell–cell interactions determined by the spatial disposition of cells are shown with igraph package (version 1.2.6.9181).

### Quantitative PCR (qPCR)

Total RNA was extracted from the skin tissues of the mice (BMDMs, PMs, or THP‐1 cells) using TRIzol™ (TaKaRa) as described by the manufacturer. Single‐stranded cDNA was synthesized from 1 μg of total RNA by reverse transcription. Real‐time PCR was performed with AceQ Universal SYBR qPCR Master Mix (Vazyme Biotech Co, Ltd, China) on a CFX 100 (Bio‐Rad, Hercules, CA) cycler using the primers listed in Appendix Table S2. The amplification program was as follows: 95°C for 2.5 min, 44 cycles at 95°C for 15 s, and 60°C for 30 s. Dissociation curves were analyzed at the end of the amplification. The level of *Gapdh* or *ACTIN* RNA expression was used to normalize the data.

### RNA‐seq analysis

Peritoneal macrophages from wild‐type and M‐*Shp2* ^−/−^ mice (8–10 weeks old) were stimulated with IMQ 4 h later, then PMs were used for total RNA isolation with TRIzol™ (TaKaRa), and subjected to RNA‐seq analysis. RNA sequencing libraries were generated using NEBNext^®^ Ultra™ RNA Library Prep kit for Illumina^®^ (NEB, USA) following the manufacturer’s recommendations. Raw data (raw reads) in fastq format were firstly processed with in‐house Perl scripts. The index of the reference genome was built using Hisat2 v.2.0.4 and paired‐end clean reads were aligned to the reference genome using Hisat2 v.2.0.4. HTSeq v.0.9.1 was used to count the numbers of reads mapped to each gene. Differential expression analysis was performed using the DESeq R package. The *P* values were adjusted using the Benjamini & Hochberg method. Corrected *P* values of 0.005 and a log 2 (fold change) of 1 were set as the cut‐offs for significantly differential expression. The GEO accession number for the RNA‐seq reported in this study is GSE147535.

### Isolation of peritoneal macrophages

Mice were intraperitoneally injected with 1 ml starch broth per mouse. After 3 days, peritoneal cells were harvested by lavaging the peritoneal cavity with 5 ml PBS. Floating cells were removed by DMEM washing and adherent peritoneal macrophages were cultured in DMEM containing 10% FBS and 1× penicillin/streptomycin at 37°C overnight, followed by treatment with different stimuli according to the experimental designs.

### Generation of bone marrow‐derived macrophages

Mice were sacrificed by cervical dislocation, and their femurs and tibias were removed. Bone marrow cells were isolated by flushing with DMEM. Red blood cells were lysed using NH_4_Cl. Bone marrow cells were cultured in DMEM containing 10% FBS supplemented with M‐CSF (10 ng/ml) for 5–7 days. Two days later, non‐adherent cells were removed, the media were freshly replenished, and cultured for 2 more days. The BMDMs were used on days 5–7.

### Immunoprecipitation

First, HEK293T cells were transfected with various epitope‐tagged vectors. After 48‐h incubation, we extracted protein using lysis buffer supplemented with protease and phosphatase inhibitors. Each sample was incubated with the indicated antibodies overnight at 4°C and then incubated with magnetic beads (Millipore) at room temperature for 1 h. Proteins not immobilized on beads were removed by washing five times with cold lysis buffer. Next, the precipitates were boiled for 10 min in an SDS loading buffer. The presence or absence of the target protein was evaluated using Western blots with the indicated antibodies. Anti GFP‐tag (1:1,000; Santa Cruz, catalog sc‐9996), anti‐HA‐tag (Cell Signal Technology, catalog 3724), anti‐TLR7 (Santa Cruz, catalog sc‐57463), anti‐TLR7 (Novus, catalog NBP2‐24906), anti‐SHP2 (Santa Cruz, catalog sc‐7384), and anti‐K63‐Ub (Cell Signal Technology, catalog 5621) were used.

### Western blotting

Cells or skin samples were lysed in radioimmunoprecipitation assay (RIPA) buffer supplemented with protease and phosphatase inhibitor (MCE). Proteins were quantified by the Bradford assay (HyClone‐Pierce). The proteins were then separated by SDS‐polyacrylamide gel electrophoresis (PAGE) and electrophoretically transferred onto polyvinylidene difluoride membranes. The membranes were probed with antibodies overnight at 4°C, and then incubated with a horseradish peroxidase‐coupled secondary antibody. Detection was performed using a LumiGLO chemiluminescent substrate system. Anti‐SHP2 (1:1,000; Santa Cruz, catalog sc‐7384), anti‐p‐IKK*α*/*β* (1:1,000; Cell Signal Technology, catalog 2697), anti‐IKK*α* (1:1,000; Cell Signal Technology, catalog 11930), anti‐IKK*β* (1:1,000; Cell Signal Technology, catalog 8943), anti‐p‐p65 (1:1,000; Cell Signal Technology, catalog 3033), anti‐p65 (1:1,000; Cell Signal Technology, catalog 8243), p‐p38 (1:1,000; Cell Signal Technology, catalog 4511), p38 (1:1,000; Cell Signal Technology, catalog 8690), p‐IRF3 (1:1,000; Cell Signal Technology, catalog 29047), IRF3 (1:1,000; Cell Signal Technology, catalog 4302), anti‐TLR7 (1:1,000; Abcam, catalog ab113524), anti‐TLR7 (1:1,000; Santa Cruz, catalog sc‐57463), anti‐TLR7 (1:1,000; Novus, catalog NBP2‐24906), anti‐NaK‐ATPase (1:1,000; Cell Signal Technology, catalog 23565), anti‐Calreticulin (1:1,000; Abcam, catalog ab92516), anti‐Giantin (1:1,000; Abcam, catalog ab80864), anti‐EEA1 (1:1,000; Cell Signal Technology, catalog 3288), anti‐GFP‐tag (1:1,000; Santa Cruz, catalog sc‐9996) and anti‐HA‐tag (1:1,000; Cell Signal Technology, catalog 3724), anti‐p‐Tyr (1:1,000; Santa Cruz, catalog sc‐7020), anti‐Ub (1:1,000; Santa Cruz, catalog sc‐8017), anti‐K63‐linkage‐specific polyubiquitin (1:1,000; Cell Signal Technology, catalog 5621S), anti‐*β*‐actin (1:2,000, Abmart, catalog M20011), and anti‐*β*‐tubulin (1:2,000, Abmart, catalog M20005) were used.

### Isolation of membrane, ER, Golgi, and endosome protein

#### Membrane

Membrane Protein Extraction kit (Thermo Scientific) was used to isolate membrane protein according to the manufacturer’s instructions. Briefly, 5 × 10^6^ cells were collected and washed in cold PBS. After centrifugation, permeabilization buffer was added to the cell pellet and centrifuged. Solubilization buffer was added to the pellet and incubated at 4°C for 30 min. After centrifugation, supernatant containing solubilized membrane and membrane‐associated proteins was transferred to a fresh tube.

#### ER

ER Enrichment kit (Invent Biotechnologies) was used to isolate ER according to the manufacturer’s instructions. Briefly, 3 × 10^7^ cells were collected and added buffer A. Then, the cell suspension was transferred to a filter cartridge and was centrifuged twice. Next, all supernatant was transferred to a fresh tube and centrifuged. After centrifugation, supernatant was transferred to a fresh tube and added buffer B, and centrifuged. The pellet cold buffer A was resuspended and vortexed vigorously. Buffer C was added and vortexed briefly. Supernatant was transferred to a fresh tube and added buffer D. After centrifugation, the pellet was dissolved in buffer WA‐009.

#### Golgi

Golgi Apparatus Enrichment Kit (Invent Biotechnologies) was used to isolate Golgi according to the manufacturer’s instructions. Briefly, 3 × 10^7^ cells were collected and washed in cold PBS, then transferred to a filter cartridge. After centrifugation, transferred supernatant to a fresh tube and added equal buffer B and vortexed briefly. After centrifugation, the pellet was resuspended in cold buffer A and centrifuged. Supernatant was transferred to a fresh tube and added cold buffer C and vortexed vigorously. After centrifugation, the pellet was dissolved in buffer WA‐009.

#### Endosome

Endosome Isolation and Cell Fractionation kit (Invent Biotechnologies) was used to isolate endosomes according to the manufacturer’s instructions. Briefly, 3 × 10^7^ cells were collected and added buffer A. Then, the cell suspension was transferred to a filter cartridge and centrifuged. The pellet was resuspended and centrifuged, next the supernatant was transferred to a fresh tube and added buffer B, and was incubated at 4°C overnight. After centrifugation, the pellet was dissolved in buffer WA‐009.

### Luciferase assay

1 × 10^5^ HEK293T cells were seeded in the wells of a 24‐well plate overnight, and each well was co‐transfected with 500 ng of different TLR7 mutant vectors, 100 ng of the firefly luciferase reporter plasmid pGL3 containing NF‐*κ*B promoter together with 0.5 ng of β‐gal‐promoter‐dependent Renilla luciferase reporter. After 24 h transfection, the cells were lysed. Firefly and Renilla luciferase activities were measured with the Dual‐Luciferase^®^ Reporter System (Promega).

### Generation of phospho‐TLR7 Y1024 antibody

Rabbit polyclonal antibody was raised against TLR7 phosphorylated at Y1024 (p‐TLR7 Y1024) in collaboration with ABclonal Biotech (Wuhan, China). In brief, 1,018–1,028aa NPQAHP(Y‐p)FWQC was coupled to KLH and used to immunized three experimental Japanese rabbits. After the rabbits were sacrificed, the affinity‐purified antibody was finally provided. The control peptide NPQAHPYFWQC was used for antibody purification and detection.

### ELISA

ELISA assays were used to detect the presence of various cytokines in mouse serum isolated from IMQ‐induced murine model animals. ELISA kits for mouse cytokines IL‐23A and IL‐17A were purchased from eBioscience. ELISA detections were all performed according to the manufacturer’s instructions.

### Statistics

Statistical analysis was performed using GraphPad Prism 9.0. Data are presented as the mean ± SEM. We assessed data for normal distribution and similar variance between groups. Unpaired two‐tailed Student’s *t*‐test, Mann–Whitney *U* test, Bonferroni or Tukey multiple‐comparison test, and Fisher’s exact test were used to assess statistical significance (*P < 0.05, **P < 0.01, ns = not significant). Error bars depict SEM. Exact *P* values are listed in Appendix Table S3.

## Author contributions

YS conceived this project and designed the study. YZ, WY, and FS performed the experiments. YZ, ZW, and DC analyzed the data. QX, BK, XJ, JG, WG, YL, and HM gave methodological support and conceptual advice. YS and YZ wrote the manuscript. All authors discussed the results and commented on the manuscript.

## Conflict of interest

Y. Sun and Q. Xu have a patent pending on use of SHP2 inhibitors in psoriasis (202010109716.0). The other authors declare no conflict of interest.

## Supporting information



Appendix

Expanded View Figures PDF

Source Data for Expanded View

Source Data for Figure 1

Source Data for Figure 2

Source Data for Figure 3

Source Data for Figure 5

Source Data for Figure 6

Source Data for Figure 7

Source Data for Figure 8

Source Data for Figure 9

## Data Availability

The scRNA‐seq data and spatial transcriptome data have been deposited to the GEO database with accession number GSE165021 http://www.ncbi.nlm.nih.gov/geo/query/acc.cgi?acc=GSE165021. The accession number for the RNA‐seq reported in this study in GEO is GSE147535 http://www.ncbi.nlm.nih.gov/geo/query/acc.cgi?acc=GSE147535.
