# Supplementary material for: Allosteric inhibition of SHP2 uncovers aberrant TLR7 trafficking in aggravating psoriasis
Source: EMBO Mol Med. 2021 Dec 22;14(3):e14455. doi: 10.15252/emmm.202114455 (PMC8899919; doi:10.15252/emmm.202114455)
Supplement: Supplementary file 1 — Appendix [file EMMM-14-e14455-s007.pdf]

Appendix Figures for

Allosteric inhibition of SHP2 uncovers aberrant TLR7 trafficking in aggravating psoriasis

Yuyu Zhu[1,2#], Zhigui Wu[1#], Wei Yan[3#], Fenli Shao[1#], Bowen Ke[4], Xian Jiang[3], Jian Gao[1], Wenjie Guo[1], Yuping Lai[5], Hongyue Ma[2], Dijun Chen[1], Qiang Xu[1] and Yang Sun[1,6*]

[1]State Key Laboratory of Pharmaceutical Biotechnology, Department of Biotechnology and Pharmaceutical Sciences, School of Life Sciences, Nanjing University, 163 Xianlin Avenue, Nanjing 210023, China; [2]College of Pharmacy, Nanjing University of Chinese Medicine, 138 Xianlin Avenue, Nanjing 210023, China; [3]Department of Dermatology and Venereology, West China Hospital, Sichuan University, Chengdu, Sichuan, China; [4]Laboratory of Anesthesia and Critical Care Medicine, Department of Anesthesiology, Translational Neuroscience Center, West China Hospital and State Key Laboratory of Biotherapy, Sichuan University, Chengdu, Sichuan 610041, China; [5]Shanghai Key Laboratory of Regulatory Biology, School of Life Sciences, East China Normal University, Shanghai 200241, China; [6]Chemistry and Biomedicine Innovation Center (ChemBIC), Nanjing University, Nanjing 210023, China.

[#]These authors contributed equally to this work.

Running title: SHP2 aggravates psoriasis via TLR7

[*] Corresponding to

Yang Sun, Ph.D., Professor

School of Life Sciences, Nanjing University, Nanjing 210023 China

Tel/Fax: +86-25-89687620;

Email: yangsun@nju.edu.cn (Y. Sun)

Appendix Fig S1 SHP2 inhibitor reduced the psoriasis-related cytokines levels in human peripheral blood mononuclear cells
Appendix Fig S2 SHP099 did not affect the expression of inflammatory factors in normal PBMCs
Appendix Fig S3 SHP099 did not affect the skin condition of normal mice
Appendix Fig S4 scRNA-seq quality control plots
Appendix Fig S5 Distinct gene signatures of mouse skin cell clusters
Appendix Fig S6 Expression of selected cluster-specific genes for all clusters
Appendix Fig S7 Gene set enrichment analysis of myeloid cells
Appendix Fig S8 Pathway enrichment analysis in M0 and M1
Appendix Fig S9 Topological structure value of hub genes
Appendix Fig S10 Deletion efficiency of SHP2 in myeloid cell lineages- and DC-knockout mice
Appendix Fig S11 SHP2-deficient macrophages produced less psoriasis-related cytokines with IMQ stimulation
Appendix Fig S12 SHP2 highly expressed in the infiltrated macrophages in both psoriatic skin and IMQ-induced murine skin
Appendix Fig S13 SHP2 highly expressed in neutrophils and DCs of skin lesion tissues of psoriatic patients
Appendix Fig S14 Spatial transcriptome sequencing analysis revealed that *PTPN11* was most strongly correlated with macrophages in lesional psoriatic skin
Appendix Fig S15 SHP2 did not affect the IRF and MAPK signaling in macrophages in response to IMQ stimulation
Appendix Fig S16 p-p65 highly expressed in macrophages of skin lesion tissues of IMQ-induced mice and psoriatic patients
Appendix Fig S17 scRNA-seq analysis revealed that *PTPN11* positively correlated with the NF-kappa B pathway in macrophages of psoriasis lesions
Appendix Fig S18 SHP2 deficiency reduced the psoriasis-related cytokines levels in R848-induced macrophages
Appendix Fig S19 SHP2 promoted TLR7 localization to the cell membrane
Appendix Fig S20 Cellular localization of TLR7 in psoriatic skin
Appendix Fig S21 The localization of TLR7 in the endosome was decreased in SHP099-treated group
Appendix Fig S22 IL-23-induced psoriasis-like phenotype was alleviated in $Tlr7^{ki}$ mice
Appendix Table S1 Information for healthy donors and psoriatic patients
Appendix Table S2 Primers for quantitative PCR analysis
Appendix Table S3 Exact *p* values for significant comparisons

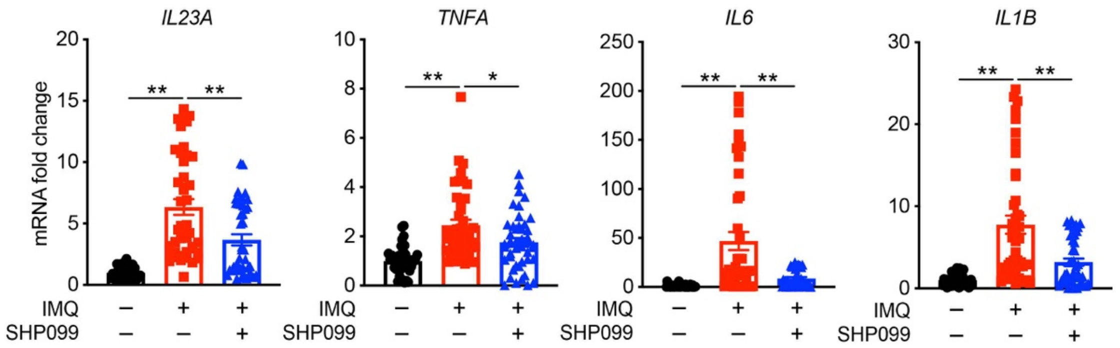

Appendix Fig S1. SHP2 inhibitor reduced the psoriasis-related cytokines levels in human peripheral blood mononuclear cells.

Quantitative PCR analysis of mRNA levels of human peripheral blood mononuclear cells derived from healthy donors (*n*=43) were untreated or pre-treated with SHP099 (10 µM) for 2 h and then stimulated by IMQ (10 µg/ml) for 6 h. The mRNA levels were normalized relative to *β*-actin.

Data information: Data are represented mean ± SEM. *P* values are determined by two-tailed unpaired Student's *t*-test. **P*<0.05, ***P*<0.01.

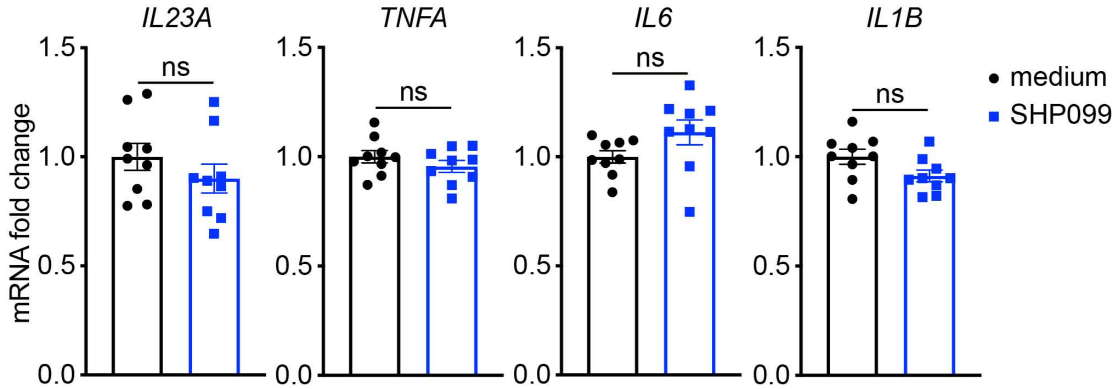

Appendix Fig S2. SHP099 did not affect the expression of inflammatory factors in normal PBMCs.

Quantitative PCR analysis of mRNA levels in human PBMCs derived from normal healthy controls (*n*=9) were treated with or without SHP099 (10 µM).

Data information: Data are represented mean ± SEM. *P* values are determined by two-tailed unpaired Student's *t* test. ns, no significant.

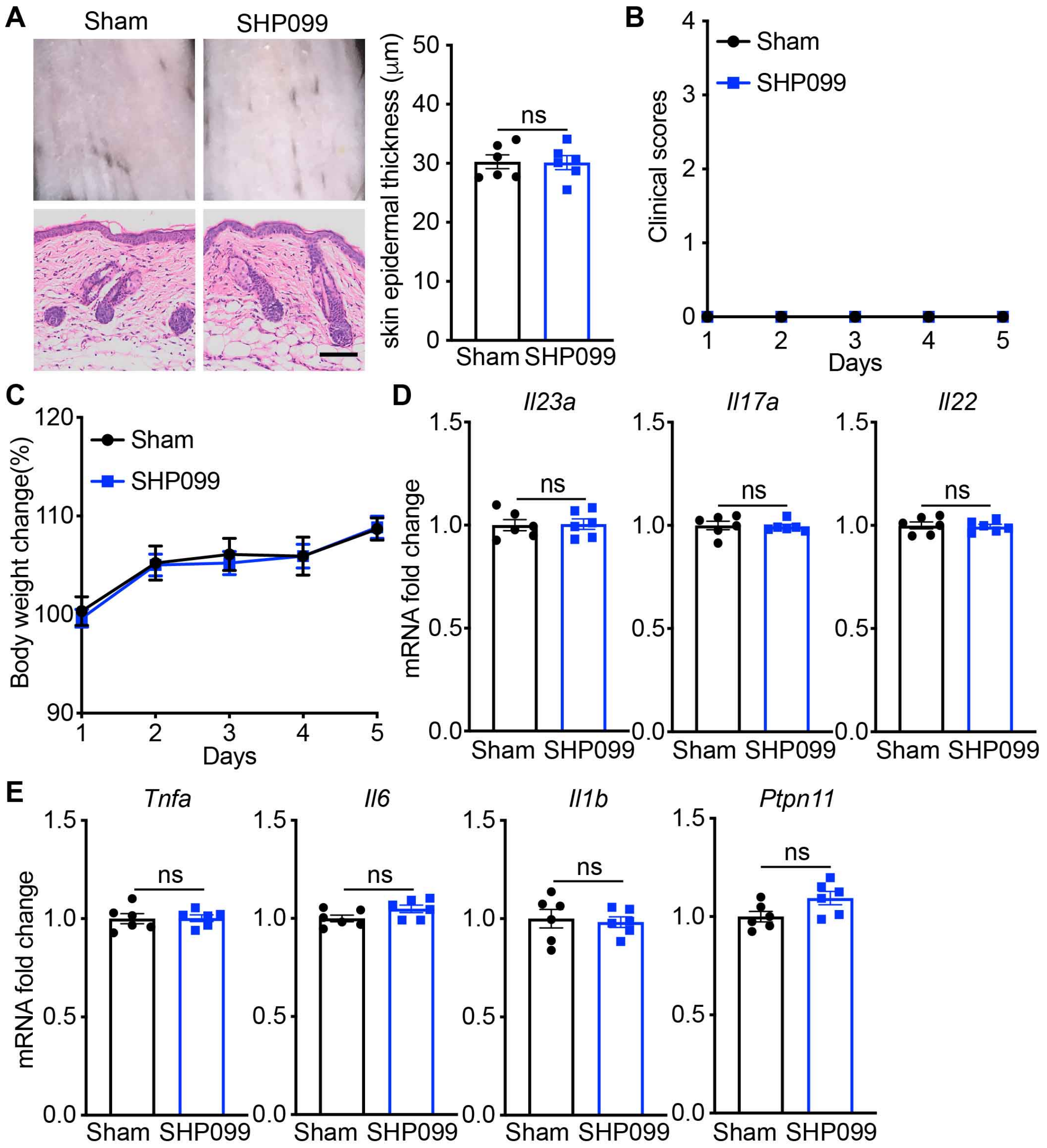

Appendix Fig S3. SHP099 did not affect the skin condition of normal mice.

C57BL/6 mice (*n*=6/group) were treated with 10 mg/kg SHP099 or vehicle for 4 days.

A Phenotypic presentation (*top*) and H&E staining (*bottom*) of dorsal skin. Scale bar: 100 μm. Left: H&E staining data; right: statistical data (mean ± SEM).

B Clinical scores plotted with mean ± SEM.

C Body weight change plotted with mean ± SEM.

D, E Quantitative PCR analysis of mRNA encoding IL-23/IL-17A axis cytokines (D) and other psoriasis-related cytokines (E) in the dorsal skin. Results were normalized to *Gapdh* expression.

Data information: Data are represented mean ± SEM. *P* values are determined by two-tailed unpaired Student's *t* test (A–E). ns, no significant.

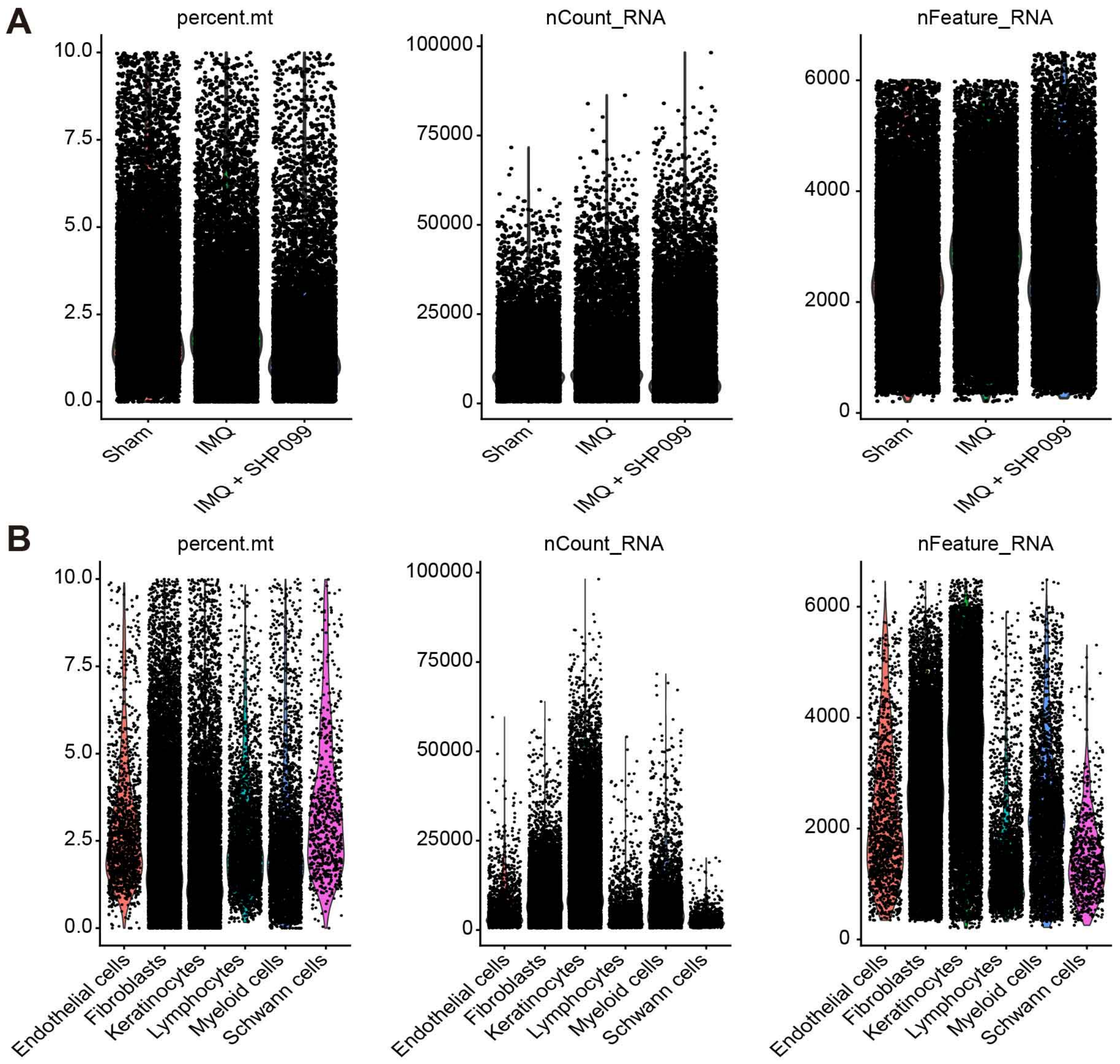

Appendix Fig S4. scRNA-seq quality control plots.

Violin plots showing the percent mitochondrial content (percent.mt), the number of UMIs (nCount_RNA) and the number of genes (nFeature_RNA) per group (A) and per cluster (B).

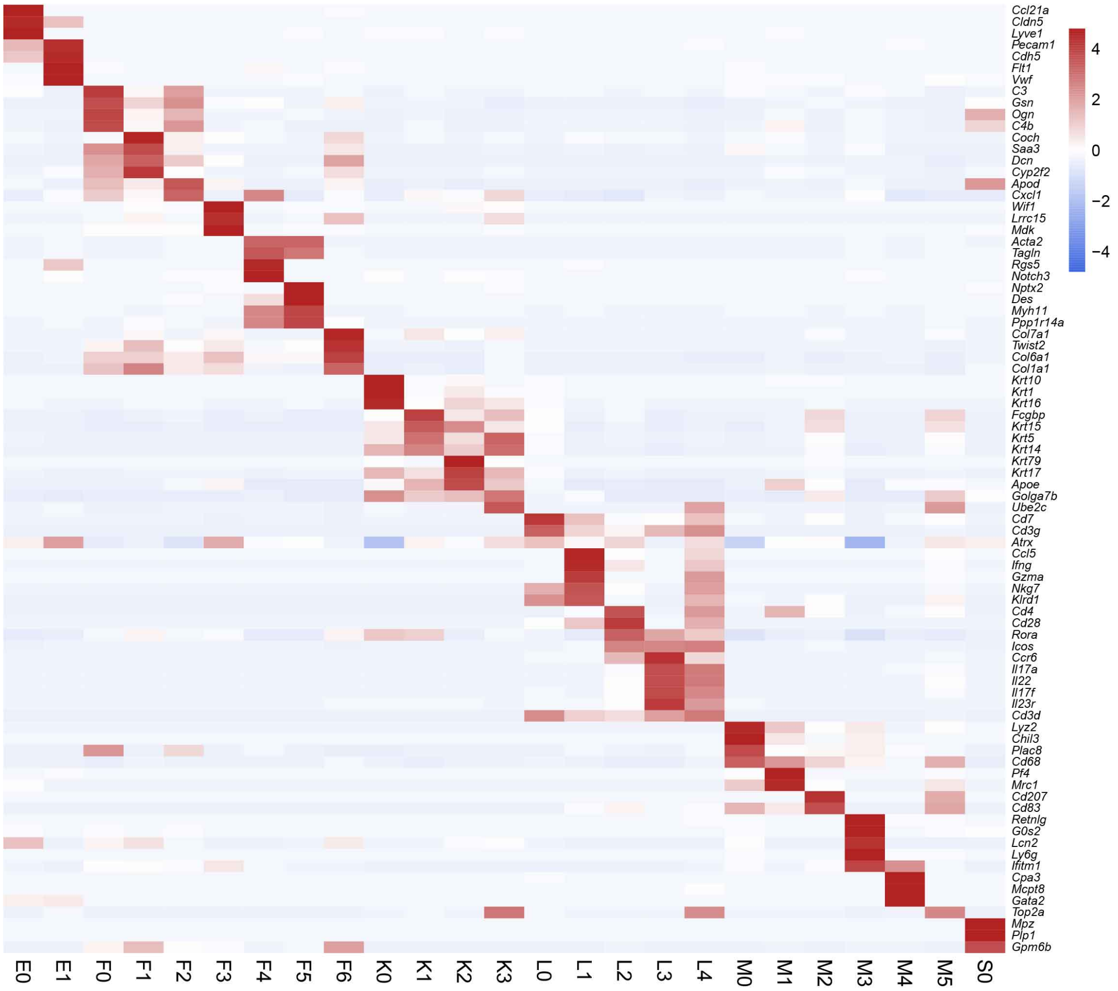

Appendix Fig S5. Distinct gene signatures of mouse skin cell clusters.
Heatmap of the top genes marking mouse skin cell clusters (from Fig. 3 D).

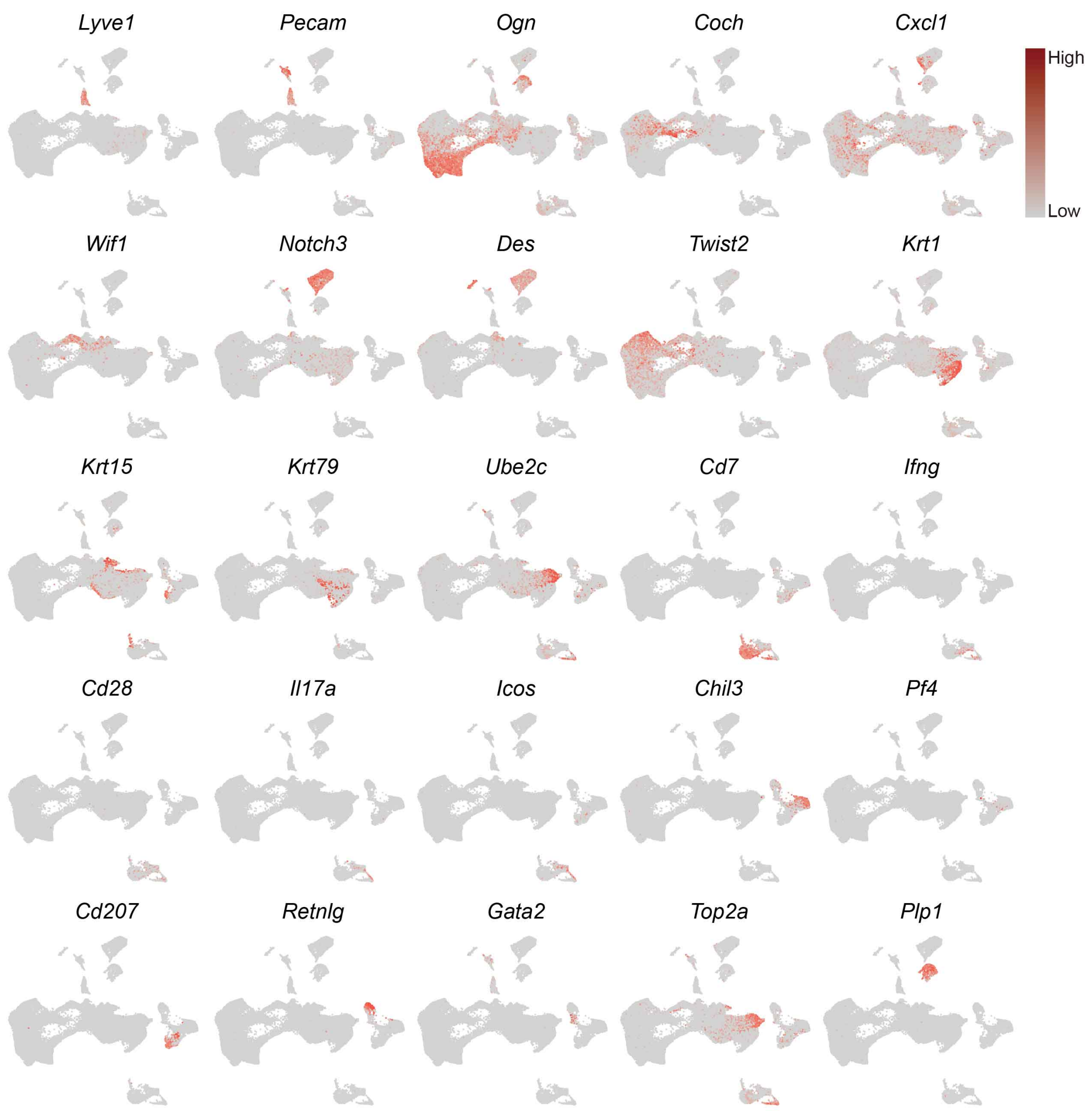

Appendix Fig S6. Expression of selected cluster-specific genes for all clusters.

Feature plots of expression distribution for selected cluster-specific genes. Expression levels are color-coded and overlaid onto UMAP plot (from Fig. 3 E).

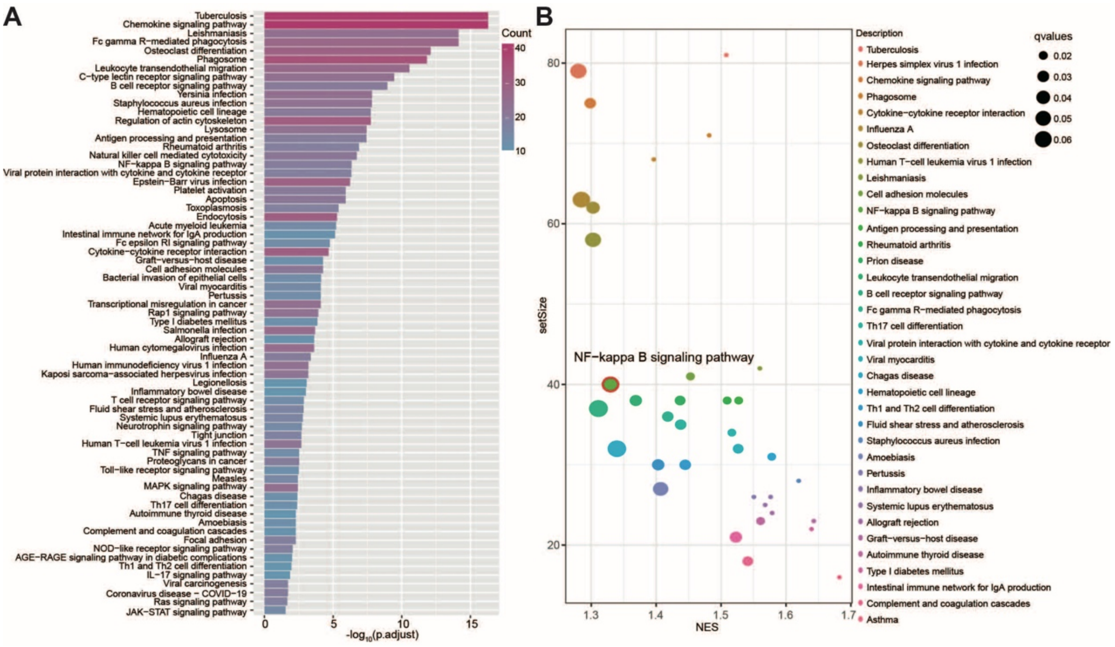

Appendix Fig S7. Gene set enrichment analysis of myeloid cells.

A KEGG pathway analysis of myeloid cells. Pathways ranked by adjusted *P* value were plotted in the visualization.

B Identification of signaling family networks dominated by myeloid cells.

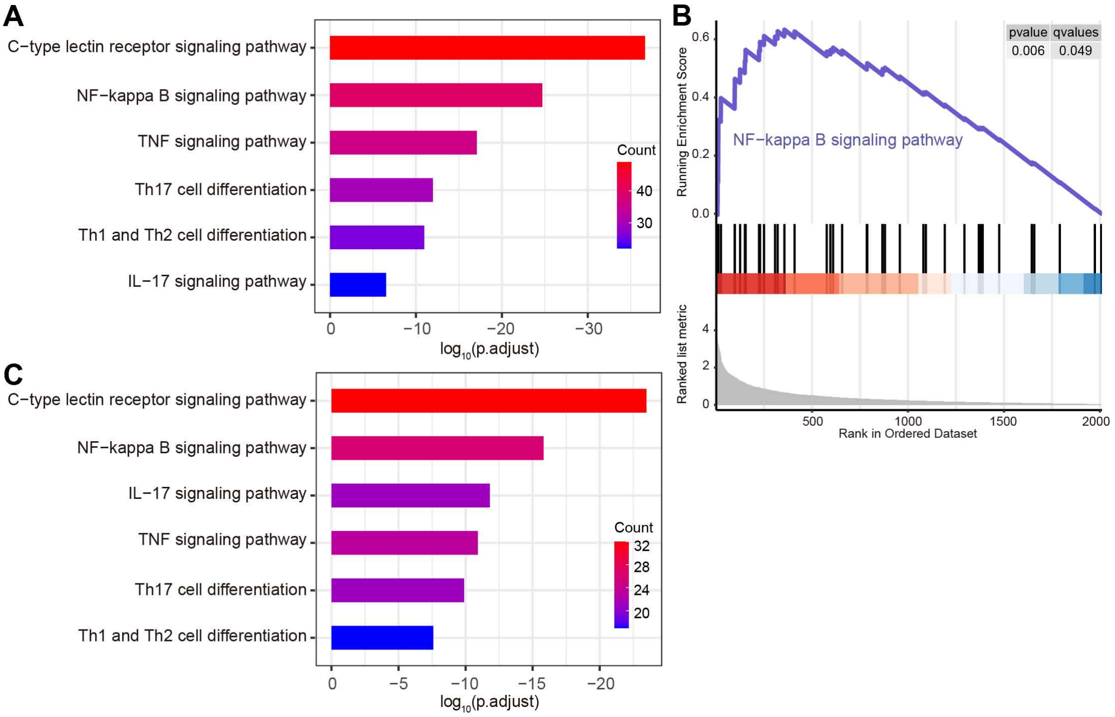

Appendix Fig S8. Pathway enrichment analysis in M0 and M1.

A, C Representative KEGG pathways enriched in upregulated genes in M0 or M1.

B Gene set enrichment analysis (GSEA) of the NF-kappa B signaling pathway in M0.

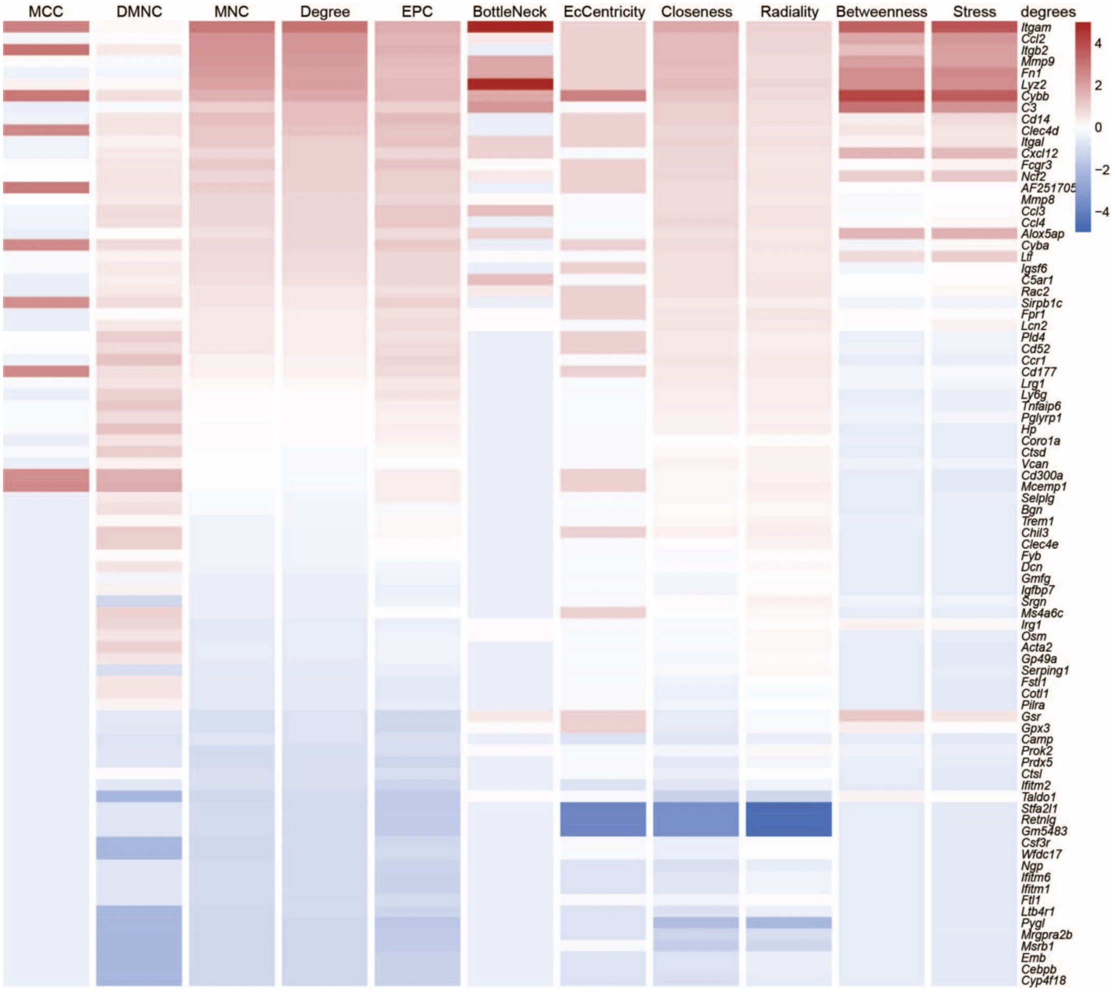

Appendix Fig S9. Topological structure value of hub genes.

Maximal Clique Centrality (MCC), Density of Maximum Neighborhood Component (DMNC), Maximum Neighborhood Component (MNC), Degree, Edge Percolated Component (EPC), congestionTopological structure values of (BN), Eccentricity, Closeness, Radiality, Betweenness and Stress were showed the size of these topological structure values by using Pheatmap.

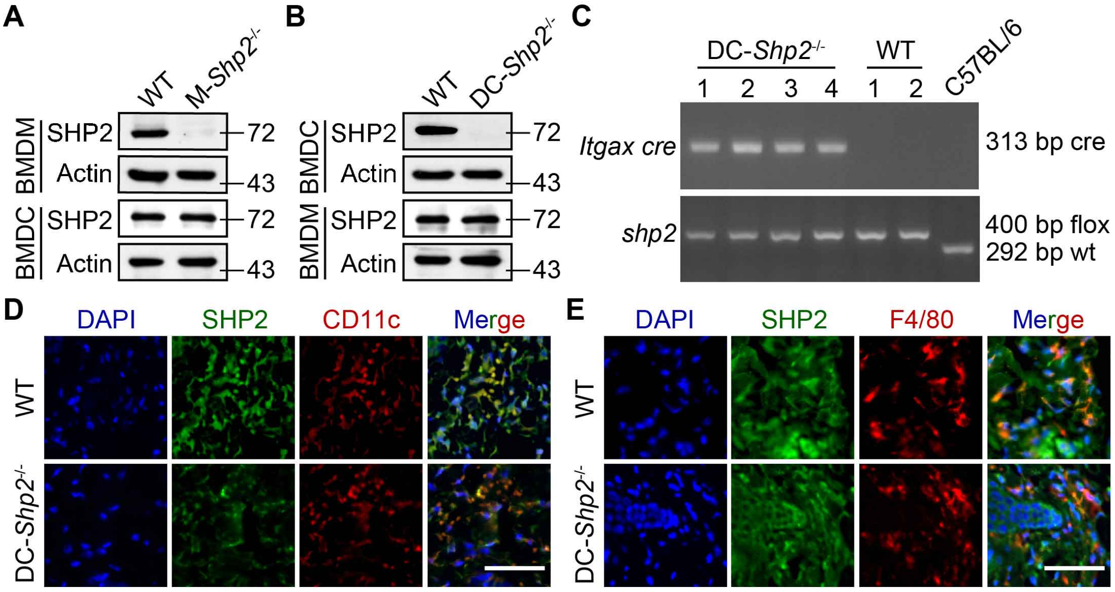

Appendix Fig S10. Deletion efficiency of SHP2 in myeloid cell lineages- and DC-knockout mice.

A, B Cell lysates from BMDMs and BMDCs generated from wild-type mice and M-*Shp2*$^{-/-}$ mice (A) or DC-*Shp2*$^{-/-}$ mice (B) were probed by anti-SHP2 and anti-Actin.

C SHP2 mRNA expression level in BMDCs of wild-type and DC-*Shp2*$^{-/-}$ mice.

D, E Immunofluorescence staining of skin sections from wild-type mice and DC-*Shp2*$^{-/-}$ mice with SHP2 and CD11c (D) or F4/80 (E), respectively showing SHP2 expression in dendritic cells or macrophages. Scale bar: 50 µm.

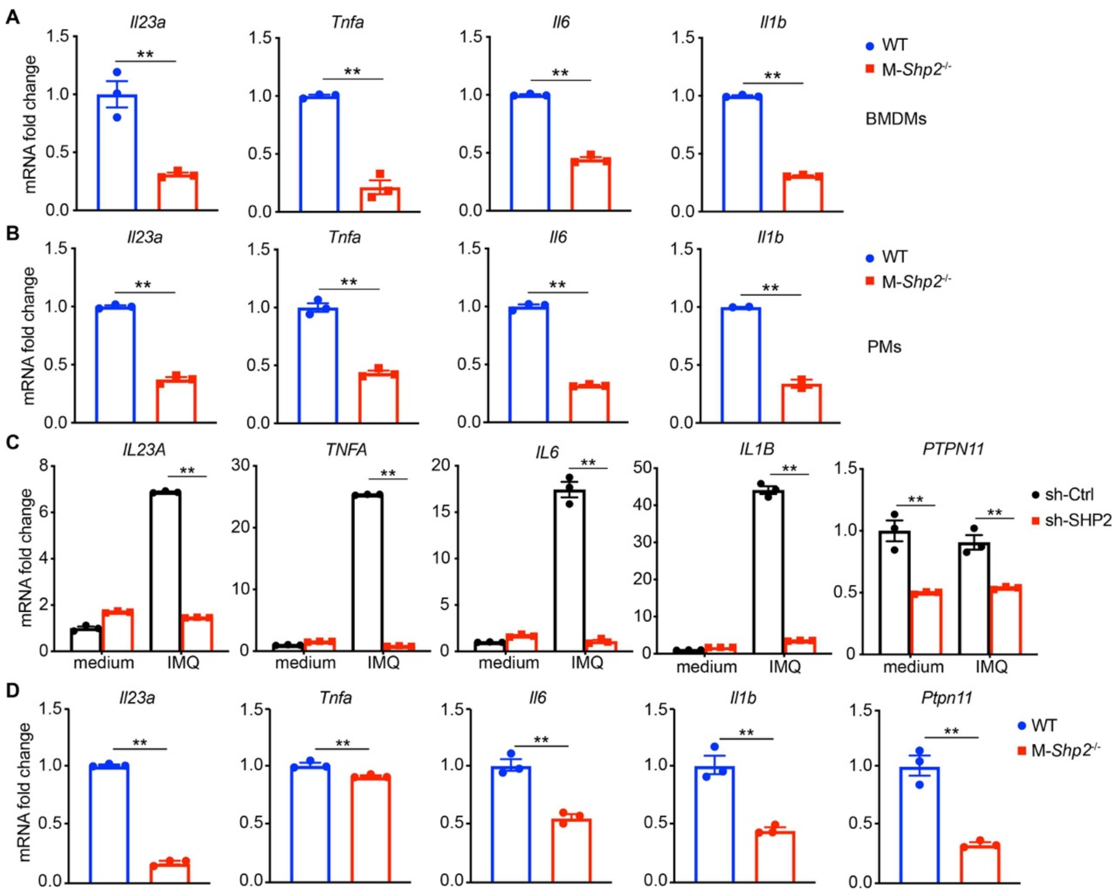

Appendix Fig S11. SHP2-deficient macrophages produced less psoriasis-related cytokines with IMQ stimulation.

A, B Quantitative PCR analysis of mRNA levels in the (A) bone-marrow derived macrophages (BMDMs) and (B) peritoneal macrophages (PMs) derived from wild-type and M-*Shp2*$^{-/-}$ mice treated with IMQ (10 µg/ml). Results were normalized to *Gapdh* expression.

C Quantitative PCR analysis of the indicated genes using PMA-differentiated THP-1 cells with shRNA-Control or shRNA-SHP2 lentivirus stimulated with IMQ (10 µg/ml) for 6 h. mRNA levels were normalized relative to *ACTIN*.

D Expression levels of representative psoriasis-related genes of peritoneal macrophages derived from wild-type and M-*Shp2*$^{-/-}$ mice treated with IL-36 (100 ng/ml) for 6 h.

Data information: Data are represented mean ± SEM. *P* values are determined by two-tailed unpaired Student's *t* test (A, B, D) or Tukey multiple comparison test (C). **P*<0.05, ***P*<0.01.

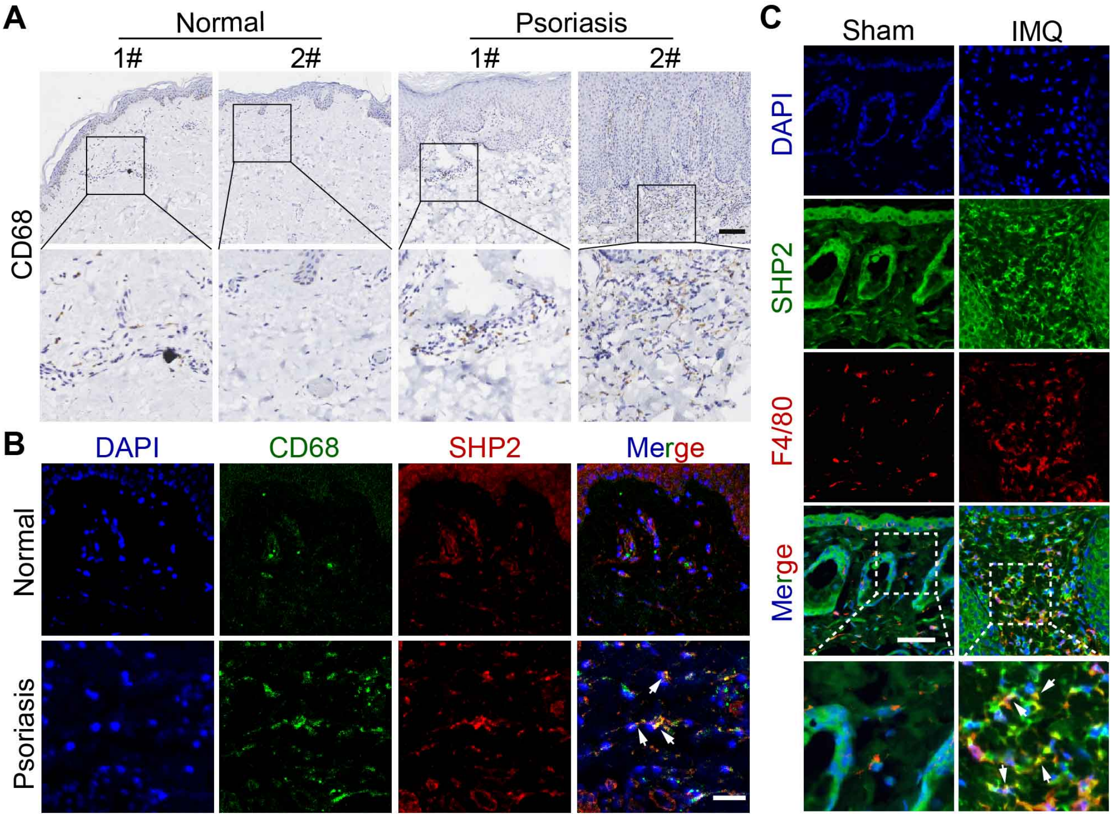

Appendix Fig S12. SHP2 highly expressed in the infiltrated macrophages in both psoriatic skin and IMQ-induced murine skin.

A Representative CD68 staining of skin sections from psoriatic patients (*n*=13) and normal controls (*n*=5). Scale bar: 200 µm.

B Immunofluorescence staining of skin sections from psoriatic patients and normal controls with SHP2 and CD68, showing SHP2 expression in macrophages (arrows). CD68, green; SHP2, red. Scale bar: 100 µm.

C Immunofluorescence staining of skin sections from IMQ-induced and sham mice with SHP2 and F4/80, showing SHP2 expression in macrophages (arrows). SHP2, green; F4/80, red. Scale bar: 50 µm.

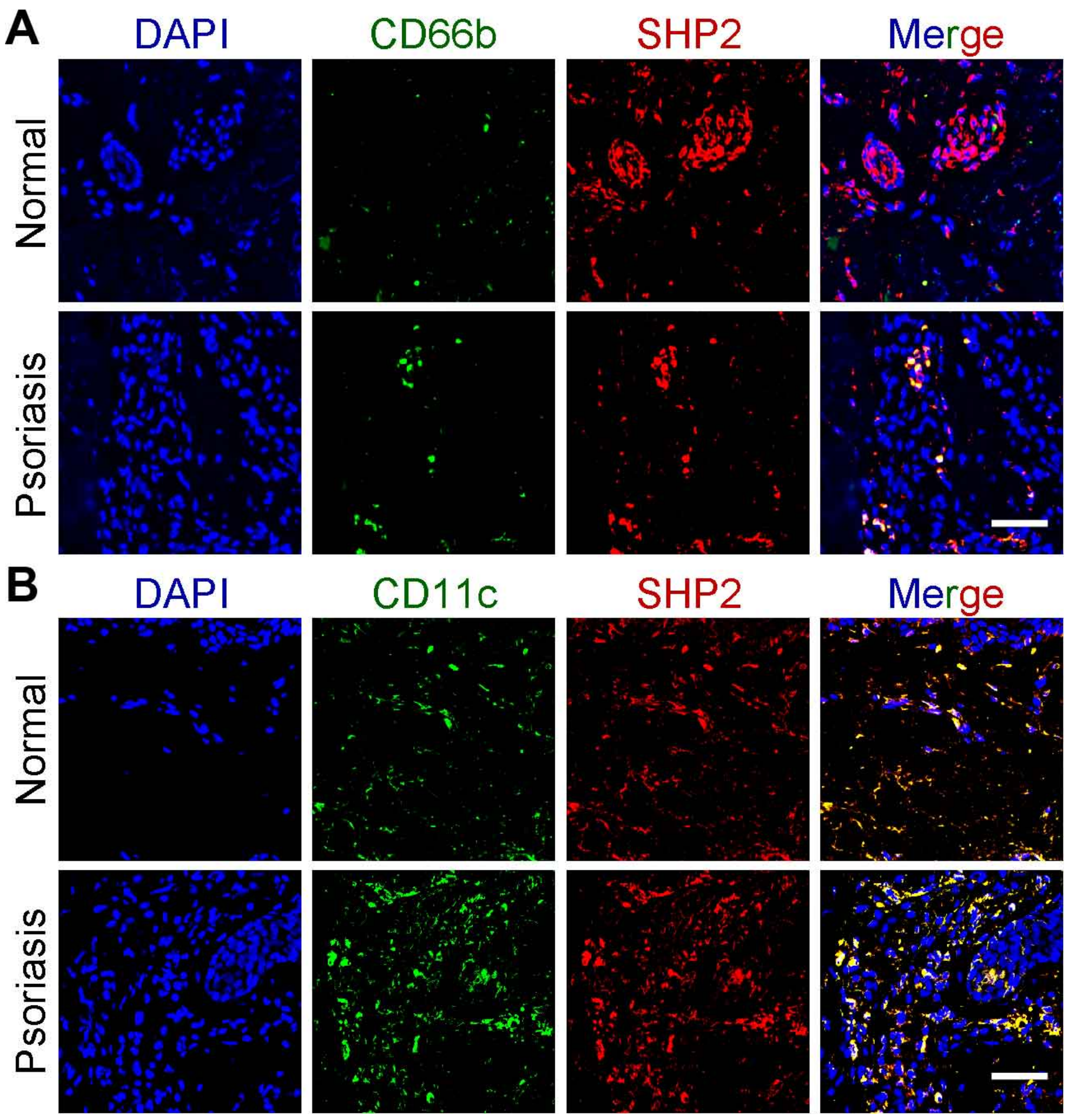

Appendix Fig S13. SHP2 highly expressed in neutrophils and DCs of skin lesion tissues of psoriatic patients.

A, B Immunofluorescence staining of skin sections from psoriatic patients and normal controls with SHP2 and CD66b (A) or CD11c (B), respectively showing SHP2 expression in neutrophils or DCs. Scale bar: 50 μm.

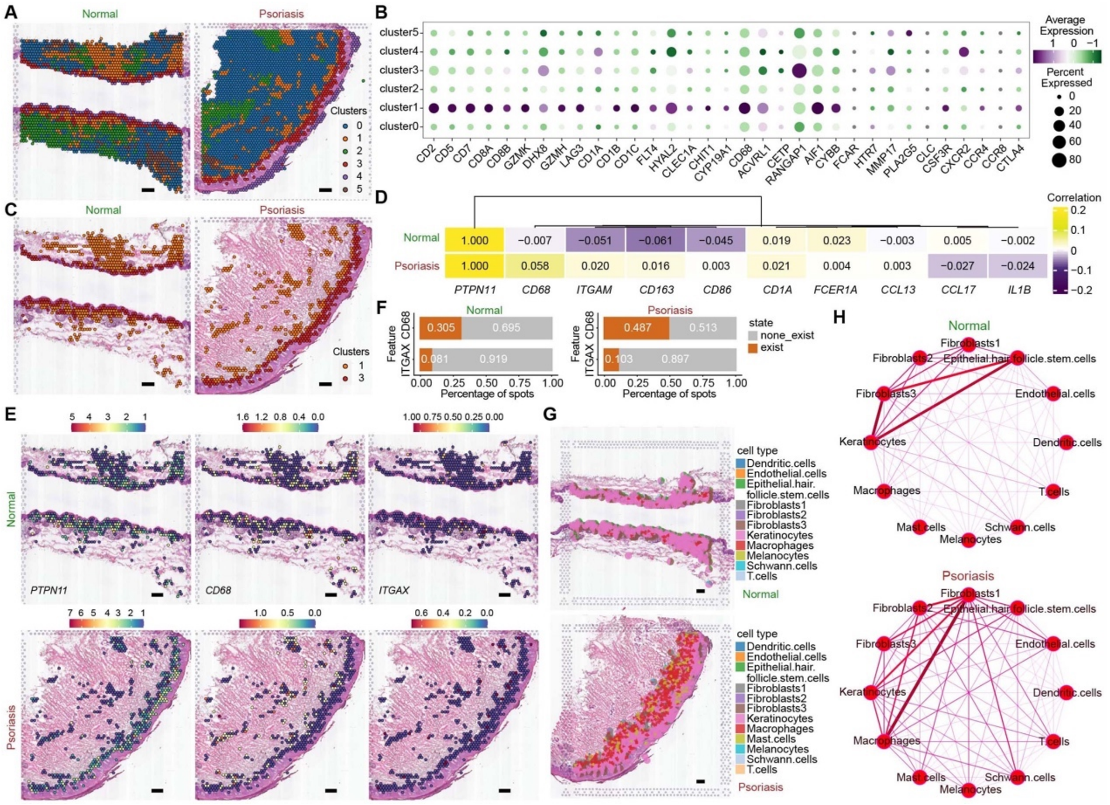

Appendix Fig S14. Spatial transcriptome sequencing analysis revealed that *PTPN11* was most strongly correlated with macrophages in lesional psoriatic skin.

A Six clusters reflecting six anatomical regions on the psoriasis slice and the normal slice. Scale bar: 500 µm.

B Dotplot showing some immune cell marker genes of all cell clusters in (A).

C Prominence of Cluster1 and Cluster3 in the dermis of psoriatic slice and normal slice. Scale bar: 500 µm.

D Heatmap showing the correlation coefficient between *PTPN11* and other genes in two groups respectively.

E Only spots expressing *PTPN11* shown in two slices (*top*) with the expression of *CD68* (*medium*) and *ITGAX* (*bottom*) on these spots. Scale bar: 500 µm.

F Barplot showing the percentage of the spots which expressing *CD68* and *ITGAX* in all spots expressing *PTPN11*, both shown in normal and psoriasis slices. Significant differences between the two groups were calculated by Fisher's exact test. The normal group was 7.623e-23, and the psoriasis was 4.542e-49.

G Spots in Cluster1 and Cluster3 showing the percentage of cell types by Scatterpieplot. Scale bar: 500 µm.

H The cell-cell interaction network between cell types shown in (G). The thickness of the connecting line indicates the strength of the interaction.

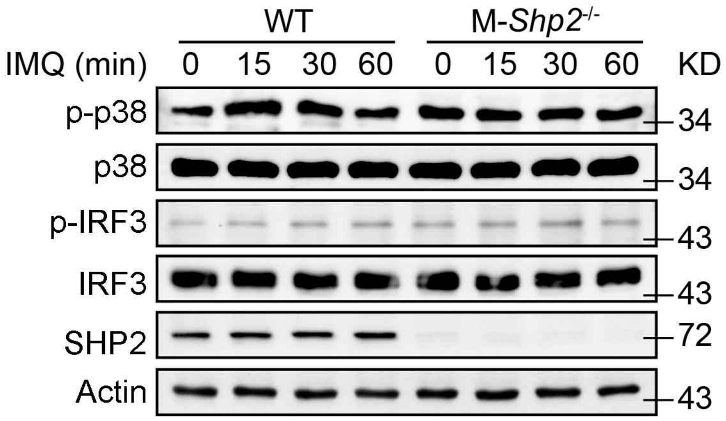

Appendix Fig S15. SHP2 did not affect the IRF and MAPK signaling in macrophages in response to IMQ stimulation.

PMs derived from wild-type and M-*Shp2*$^{-/-}$ mice were stimulated by IMQ (10 µg/ml) for indicated times. Whole cell lysates were subjected to Western blotting.

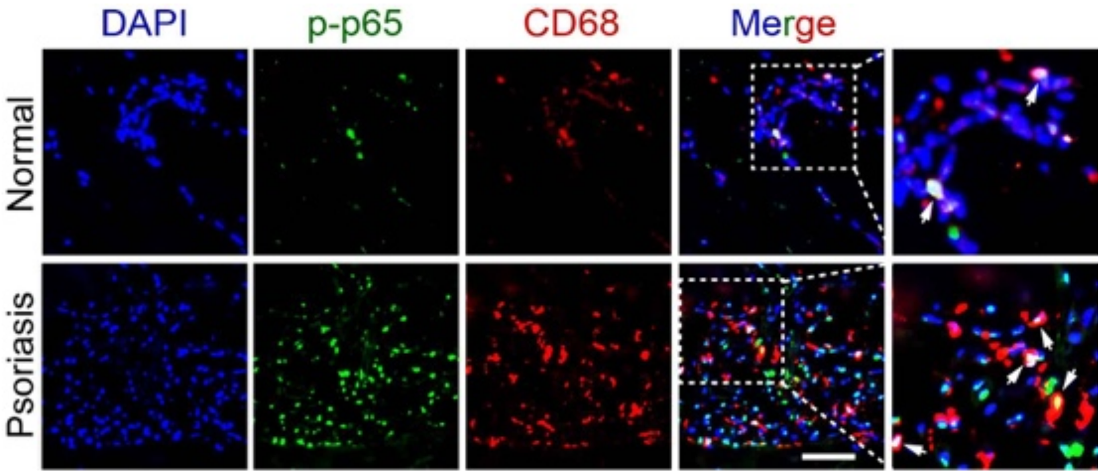

Appendix Fig S16. p-p65 highly expressed in macrophages of skin lesion tissues of IMQ-induced mice and psoriatic patients.

Representative p-p65 immunofluorescence staining of skin sections from psoriatic patients and normal healthy controls. Scale bar: 50 µm.

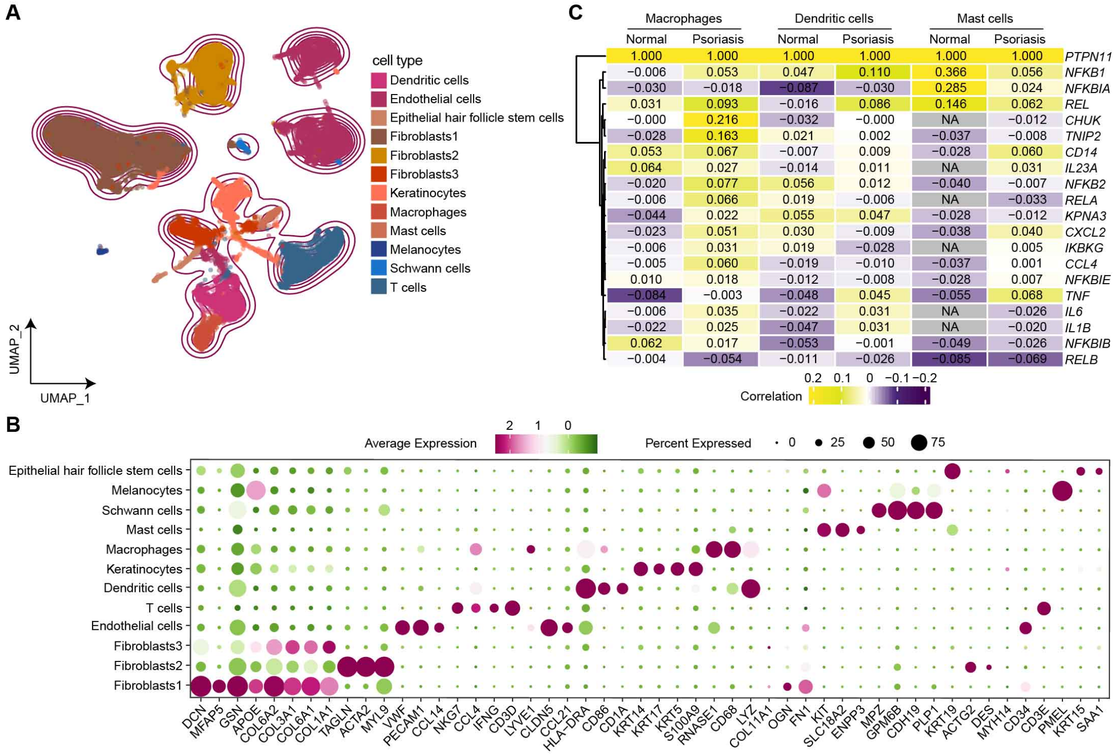

Appendix Fig S17. scRNA-seq analysis revealed that *PTPN11* positively correlated with the NF-kappa B pathway in macrophages of psoriasis lesions.

A Uniform Manifold Approximation (UMAP) plot of all cells colored by their cell type.

B Dotplot showing some marker genes of all cell types in (A).

C Heatmap showing the correlation coefficient between *PTPN11* and each gene from NF-kappa B signaling pathway in six groups (Macrophages in Normal, Macrophages in Psoriasis, Dendritic cells in Normal, Dendritic cells in Psoriasis, Mast cells in Normal, and Mast cells in Psoriasis) respectively.

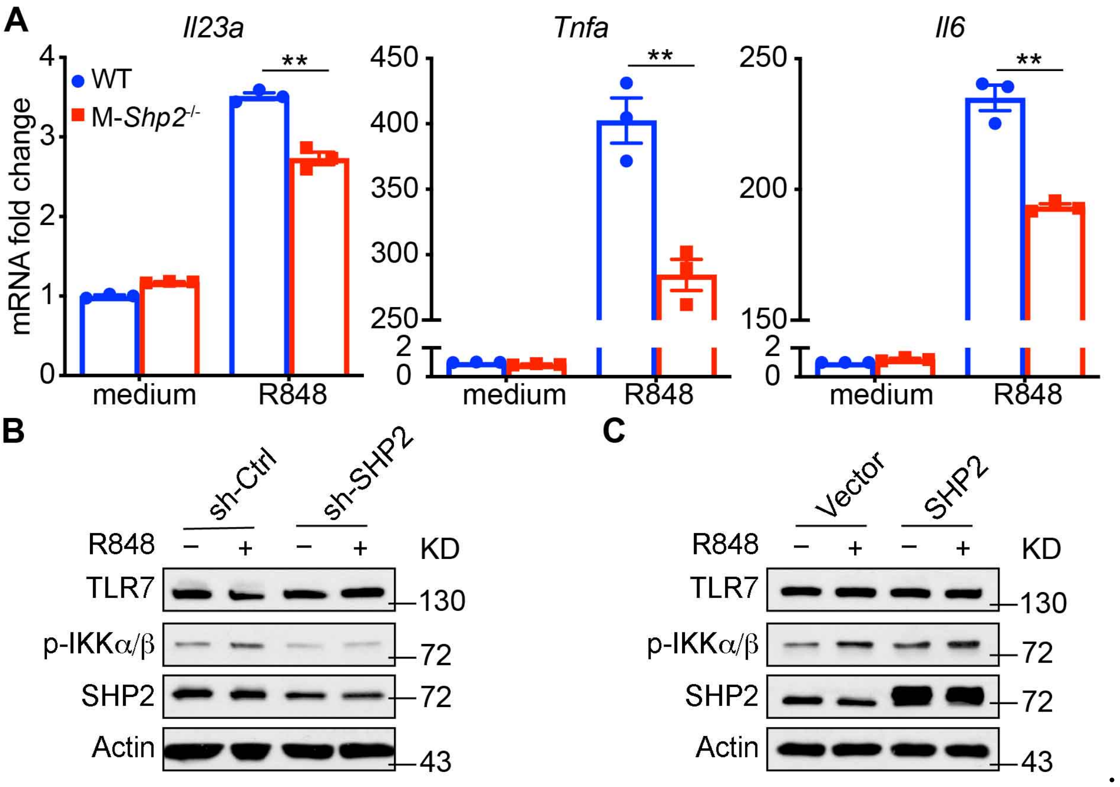

Appendix Fig S18. SHP2 deficiency reduced the psoriasis-related cytokines levels in R848-induced macrophages.

A Expression levels of representative psoriasis-related genes of peritoneal macrophages derived from wild-type and M-*Shp2*$^{-/-}$ mice treated with R848 (10 µg/ml) for 6 h.

B, C PMA-differentiated THP-1 cells with shRNA-Control, shRNA-SHP2 (B), vector or SHP2 lentivirus (C) were unstimulated or stimulated by R848 (10 µg/ml) for 30 min. Whole cell lysates were resolved by 10% SDS-PAGE and then probed by respective antibodies.

Data information: Data are represented mean ± SEM. *P* values are determined by Tukey multiple comparison test (A). ***P*<0.01.

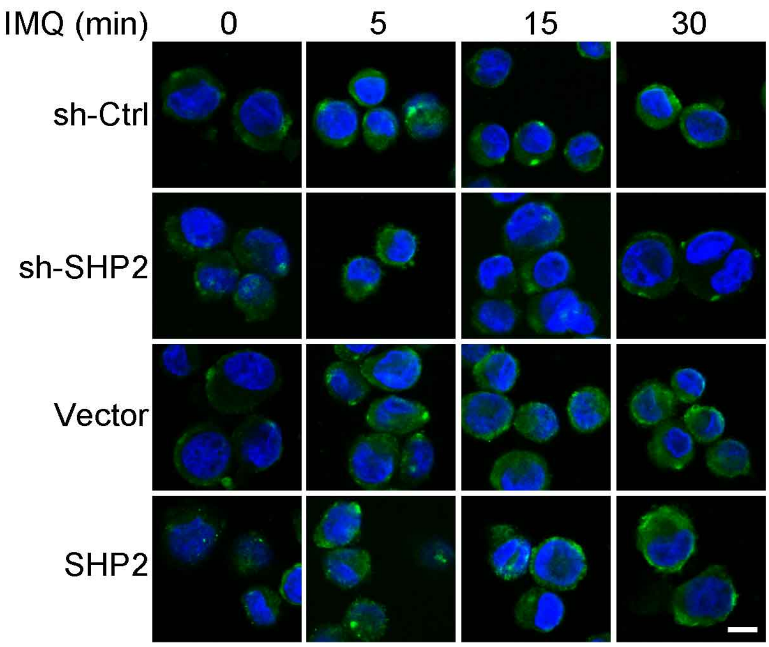

Appendix Fig S19. SHP2 promoted TLR7 localization to the cell membrane.
Confocal microscopy imaging of TLR7 in PMA-differentiated THP-1 cells, lentivirus-infected as indicated, and treated with IMQ (10 µg/ml) for various times. Scale bar: 10 µm.

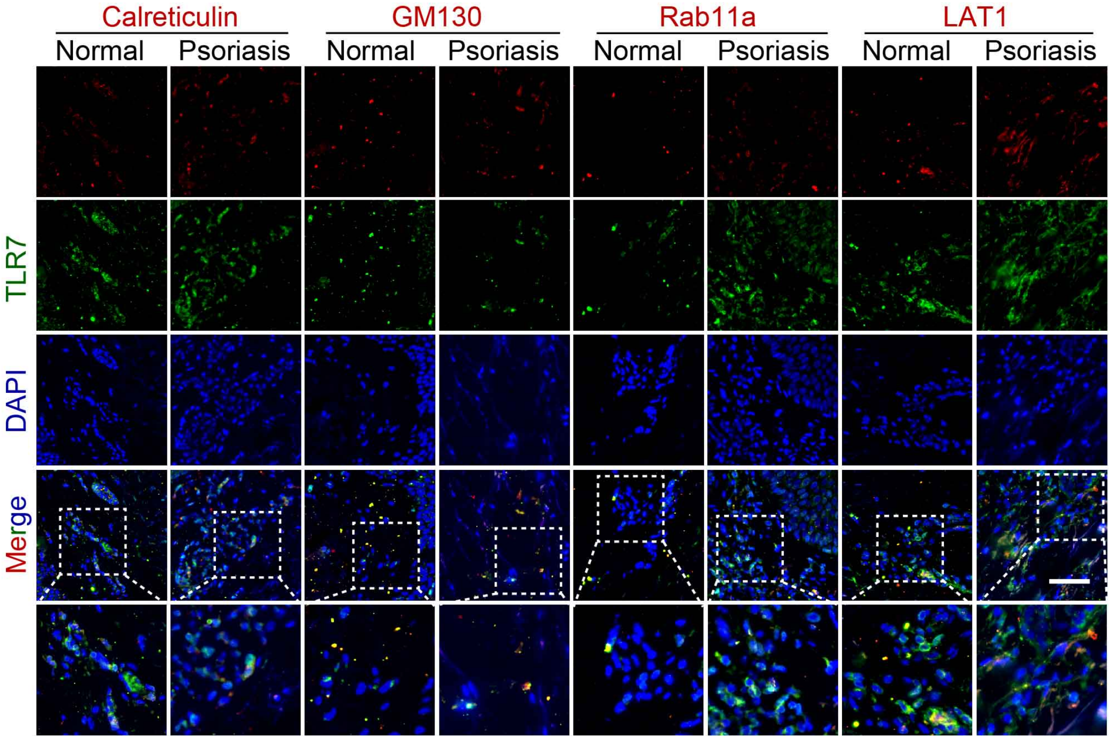

Appendix Fig S20. Cellular localization of TLR7 in psoriatic skin.

Skin sections from psoriasis patients and healthy controls were immune-stained for TLR7 together with a marker of the ER (Calreticulin), Golgi (GM130), and recycling endosome (Rab11a) or cell membrane (LAT1) prior to analysis by confocal microscopy. Scale bar: 50 µm.

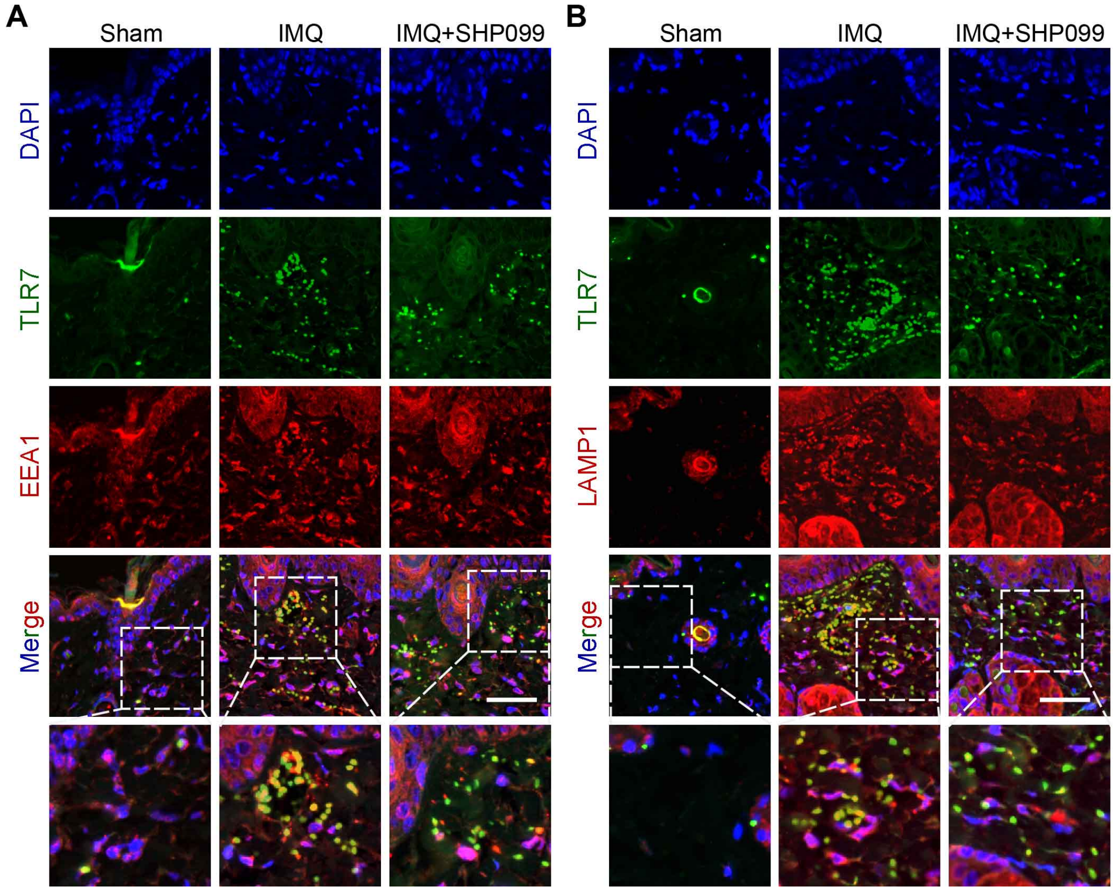

Appendix Fig S21. The localization of TLR7 in the endosome was decreased in SHP099-treated group.

Confocal microscopy imaging of skin sections from indicated mice labelled with anti-TLR7 (green), anti-EEA1 (red) or anti-LAMP1 (red) and DAPI (blue), showing TLR7 expression on early or late endosome, respectively. Scale bar: 50 μm.

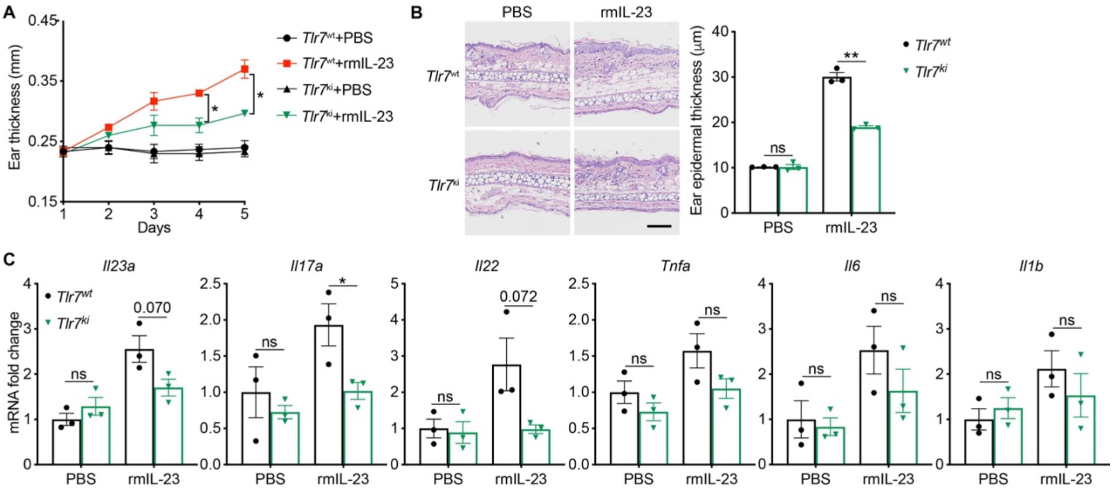

Appendix Fig S22. IL-23-induced psoriasis-like phenotype was alleviated in *Tlr7*ki mice.

*Tlr7*wt male mice (*n*=3) and *Tlr7*-Y1025D mutant *Tlr7*ki male mice (*n*=3) were treated with indicated dose of rmIL-23 or PBS for 4 days.

A, B Ear thickness (A), H&E staining and statistic results (B) of ear skin from *Tlr7*wt and *Tlr7*ki mice injected intradermally with rmIL-23 or PBS for 4 days. Ear thickness was measured daily.

C Quantitative PCR analysis of mRNA encoding IL-23/IL-17A axis cytokines and other psoriasis-related cytokines in the ear skin. Results were normalized to *Gapdh* expression.

Data information: Data are represented mean ± SEM. *P* values are determined by Tukey multiple comparison test (A–C). **P*<0.05, ***P*<0.01, ns, not significant.

Appendix Table S1. Information for healthy donors and psoriatic patients.

Healthy Donors			Psoriatic Patients		
Sample/ID	Age	Gender	Sample/ID	Age	Gender
1	29	M	1	31	M
2	43	M	2	48	M
3	49	M	3	43	F
4	54	M	4	46	M
5	35	M	5	44	F
6	50	M	6	49	M
7	47	M	7	43	M
8	45	M	8	38	M
9	65	M	9	32	M
10	42	M	10	51	F
11	45	M	11	45	M
12	54	F	12	33	M
13	51	M	13	35	M
14	46	M	14	44	M
15	30	M	15	55	M
16	35	M	16	47	M
17	43	F	17	51	M
18	46	F	18	40	M
19	46	M	19	41	M
20	41	M	20	45	M
21	43	M	21	46	M
22	46	M	22	66	M
23	34	F	23	33	F
24	28	F	24	29	F
25	26	F	25	28	F

Appendix Table S2. Primers for quantitative PCR analysis.

Primers	Sequences (5′-3′)
Human-*PTPN11*-F	GAACTGTGCAGATCCTACCTCT
Human-*PTPN11*-R	TCTGGCTCTCTCGTACAAGAAA
Human-*IL23A*-F	CTCAGGGACAACAGTCAGTTC
Human-IL23A-R	ACAGGGCTATCAGGGAGCA
Human-*TNFA*-F	CCTCTCTCTAATCAGCCCTCTG
Human-*TNFA*-R	GAGGACCTGGGAGTAGATGAG
Human-*IL6*-F	ACTCACCTCTTCAGAACGAATTG
Human-*IL6*-R	CCATCTTTGGAAGGTTCAGGTTG
Human-*IL1B*-F	AGCTACGAATCTCCGACCAC
Human-*IL1B*-R	CGTTATCCCATGTGTCGAAGAA
Human-*ACTIN*-F	ATTGGCAATGAGCGGTTC
Human-*ACTIN*-R	GGATGCCACAGGACTCCAT
Mouse-*Ptpn11*-F	GAAACGGTCATTCAGCCACT
Mouse-*Ptpn11*-R	GCAGCCAAGGAGTCATCTTC
Mouse-*Il23a*-F	AATAATGTGCCCCGTATCCAGT
Mouse-*Il23a*-R	GCTCCCCTTTGAAGATGTCAG
Mouse-*Il17a*-F	TCGAGAAGATGCTGGTGGGT
Mouse-*Il17a*-R	CTCTGTTTAGGCTGCCTGGC
Mouse-*Il22*-F	ATGAGTTTTTCCCTTATGGGGAC
Mouse-*Il22*-R	GCTGGAAGTTGGACACCTCAA
Mouse-*Tnfa*-F	CCTCTCTCTAATCAGCCCTCTG
Mouse-*Tnfa*-R	GAGGACCTGGGAGTAGATGAG
Mouse-*Il6*-F	TAGTCCTTCCTACCCCAATTTCC
Mouse-*Il6*-R	TTGGTCCTTAGCCACTCCTTC
Mouse-*Il1b*-F	GCAACTGTTCCTGAACTCAACT
Mouse-*Il1b*-R	ATCTTTTGGGGTCCGTCAACT
Mouse-*Il1a*-F	CGAAGACTACAGTTCTGCCATT
Mouse-*Il1a*-R	GACGTTTCAGAGGTTCTCAGAG
Mouse-*Il1rn*-F	GCTCATTGCTGGGTACTTACAA
Mouse-*Il1rn*-R	CCAGACTTGGCACAAGACAGG
Mouse-*Ptgs2*-F	TGAGCAACTATTCCAAACCAGC
Mouse-*Ptgs2*-R	GCACGTAGTCTTCGATCACTATC
Mouse-*Csf2*-F	GGCCTTGGAAGCATGTAGAGG

Mouse-*Csf2*-R	GGAGAACTCGTTAGAGACGACTT
Mouse-*Nfkb2*-F	GGCCGGAAGACCTATCCTACT
Mouse-*Nfkb2*-R	CTACAGACACAGCGCACACT
Mouse-*Bcl3*-F	CCGGAGGCCCTTTACTACCA
Mouse-*Bcl3*-R	GGAGTAGGGGTGAGTAGGCAG
Mouse-*Ccl9*-F	CCCTCTCCTTCCTCATTCTTACA
Mouse-*Ccl9*-R	AGTCTTGAAAGCCCATGTGAAA
Mouse-*Ccl7*-F	GCTGCTTTCAGCATCCAAGTG
Mouse-*Ccl7*-R	GTCTTGAAGCGACTACTGGTG
Mouse-*Ccl2*-F	TTAAAAACCTGGATCGGAACCAA
Mouse-*Ccl2*-R	GCATTAGCTTCAGATTTACGGGT
Mouse-*Adora2b*-F	AGCTAGAGACGCAAGACGC
Mouse-*Adora2b*-R	GTGGGGGTCTGTAATGCACT
Mouse-*Tarm1*-F	TCTAGGCTCCTTTCCCTTCTC
Mouse-*Tarm1*-R	GGGGTGGGGCTCTTACATT
Mouse-*Gm6377*-F	TACACCGGACAATTCACTTTCC
Mouse-*Gm6377*-R	GCAACAGGTAGCTCATCCATAG
Mouse-*Cish*-F	ATGGTCCTTTGCGTACAGGG
Mouse-*Cish*-R	GGAATGCCCCAGTGGGTAAG
Mouse-*Hbegf*-F	CGGGGAGTGCAGATACCTG
Mouse-*Hbegf*-R	TTCTCCACTGGTAGAGTCAGC
Mouse-*Flrt3*-F	CCTCATCGGGACTAAAATTGGG
Mouse-*Flrt3*-R	GCAAGTTCTTCAAATCGGAAGGA
Mouse-*Osm*-F	CCCGGCACAATATCCTCGG
Mouse-*Osm*-R	TCTGGTGTTGTAGTGGACCGT
Mouse-*Sphk1*-F	AAAATACTGAGAAACTCGGTCGG
Mouse-*Sphk1*-R	GCATCGCTTCTTAAAGTCCAGA
Mouse-*Srgn*-F	CTCGCCTTCGTCCTGGTTT
Mouse-*Srgn*-R	CCTCGATGCAGTTCGCAAAAA
Mouse-*Gapdh*-F	AGGTCGGTGTGAACGGATTTG
Mouse-*Gapdh*-R	TGTAGACCATGTAGTTGAGGTCA

Appendix Table S3. Exact p values for significant comparisons.

Figure 1

Fig 1A		
Two-tailed Mann-Whitney *U* test	Summary	*p* value
Normal **vs** Psoriasis	**	0.0004
Fig 1B		
Two-tailed Mann-Whitney *U* test	Summary	*p* value
Normal **vs** Psoriasis	**	0.0023
Fig 1E		
Two-tailed unpaired Student's *t*-test	Summary	*p* value
Normal **vs** Psoriasis	*	0.0399
Fig 1G		
Two-tailed unpaired Student's *t*-test	Summary	*p* value
Normal **vs** Psoriasis	**	<0.0001
Fig 1I		
Two-tailed unpaired Student's *t*-test	Summary	*p* value
medium (Normal) **vs** medium (Psoriasis)-*IL23A*	**	<0.0001
medium (Psoriasis) **vs** SHP099 (Psoriasis)-*IL23A*	**	<0.0001
medium (Normal) **vs** medium (Psoriasis)-*TNFA*	**	<0.0001
medium (Psoriasis) **vs** SHP099 (Psoriasis)-*TNFA*	**	<0.0001
medium (Normal) **vs** medium (Psoriasis)-*IL6*	**	<0.0001
medium (Psoriasis) **vs** SHP099 (Psoriasis)-*IL6*	**	<0.0001
medium (Normal) **vs** medium (Psoriasis)-*IL1B*	**	<0.0001
medium (Psoriasis) **vs** SHP099 (Psoriasis)-*IL1B*	**	<0.0001

Figure 2

Fig 2A		
Two-tailed unpaired Student's *t*-test	Summary	*p* value
Sham **vs** IMQ	**	<0.0001
IMQ **vs** IMQ+SHP099 1 mg/kg	**	<0.0001
IMQ **vs** IMQ+SHP099 3 mg/kg	**	<0.0001
IMQ **vs** IMQ+SHP099 10 mg/kg	**	<0.0001
Fig 2B		
Tukey multiple comparison test	Summary	*q* value
IMQ **vs** IMQ+SHP099 10 mg/kg-Day 3	*	0.014169
IMQ **vs** IMQ+SHP099 10 mg/kg-Day 4	*	0.020442
Fig 2C		
Two-tailed unpaired Student's *t*-test	Summary	*p* value
Sham **vs** IMQ-*Il23a*	*	0.0168
IMQ **vs** IMQ+SHP099 10 mg/kg-*Il23a*	**	0.0002
Sham **vs** IMQ-*Il17a*	*	0.0172
IMQ **vs** IMQ+SHP099 10 mg/kg-*Il17a*	**	0.0042
Sham **vs** IMQ-*Il22*	**	<0.0001

IMQ **vs** IMQ+SHP099 10 mg/kg-*Il22*	**	<0.0001
Fig 2D		
Two-tailed unpaired Student's *t*-test	Summary	*p* value
Sham **vs** IMQ-*Tnfa*	**	0.0014
IMQ **vs** IMQ+SHP099 10 mg/kg-*Tnfa*	*	0.0107
Sham **vs** IMQ-*Il6*	**	0.0014
IMQ **vs** IMQ+SHP099 10 mg/kg-*Il6*	**	0.0006
Sham **vs** IMQ-*Il1b*	**	<0.0001
IMQ **vs** IMQ+SHP099 10 mg/kg-*Il1b*	**	0.0002
Fig 2E		
Two-tailed unpaired Student's *t*-test	Summary	*p* value
Sham **vs** IMQ-IL23p19	**	<0.0001
IMQ **vs** IMQ+SHP099 10 mg/kg-IL23p19	**	0.0007
Sham **vs** IMQ-IL17A	**	<0.0001
IMQ **vs** IMQ+SHP099 10 mg/kg-IL17A	**	<0.0001

Figure 5

Fig 5B		
Tukey multiple comparison test	Summary	*q* value
WT **vs** M-*Shp2*$^{-/-}$ Sham	ns	0.070304
WT **vs** M-*Shp2*$^{-/-}$ IMQ	**	0.000745
Fig 5C		
Bonferroni multiple comparison test	Summary	*q* value
WT **vs** M-*Shp2*$^{-/-}$ Day 2	ns	0.241504
WT **vs** M-*Shp2*$^{-/-}$ Day 3	*	0.015799
WT **vs** M-*Shp2*$^{-/-}$ Day 4	*	0.063882
WT **vs** M-*Shp2*$^{-/-}$ Day 5	*	0.063882
Fig 5D		
Tukey multiple comparison test	Summary	*q* value
WT **vs** M-*Shp2*$^{-/-}$ Sham-*Il23a*	ns	0.317054
WT **vs** M-*Shp2*$^{-/-}$ IMQ-*Il23a*	*	0.015144
WT **vs** M-*Shp2*$^{-/-}$ Sham-*Tnfa*	ns	0.389736
WT **vs** M-*Shp2*$^{-/-}$ IMQ-*Tnfa*	**	0.002468
WT **vs** M-*Shp2*$^{-/-}$ Sham-*Il6*	ns	0.372468
WT **vs** M-*Shp2*$^{-/-}$ IMQ-*Il6*	**	0.0004
WT **vs** M-*Shp2*$^{-/-}$ Sham-*Il1b*	ns	0.179877
WT **vs** M-*Shp2*$^{-/-}$ IMQ-*Il1b*	**	0.000064
WT **vs** M-*Shp2*$^{-/-}$ Sham-*Ptpn11*	**	<0.000001
WT **vs** M-*Shp2*$^{-/-}$ IMQ-*Ptpn11*	**	<0.000001
Fig 5F		
Tukey multiple comparison test	Summary	*q* value
WT **vs** DC-*Shp2*$^{-/-}$ Sham	ns	0.685064
WT **vs** DC-*Shp2*$^{-/-}$ IMQ	ns	0.685064

Fig 5G		
Bonferroni multiple comparison test	Summary	*q* value
WT **vs** DC-*Shp2*$^{-/-}$ Day 2	ns	0.741511
WT **vs** DC-*Shp2*$^{-/-}$ Day 3	ns	0.741511
WT **vs** DC-*Shp2*$^{-/-}$ Day 4	ns	0.741511
WT **vs** DC-*Shp2*$^{-/-}$ Day 5	ns	0.741511

Fig 5H		
Tukey multiple comparison test	Summary	*q* value
WT **vs** DC-*Shp2*$^{-/-}$ Sham-*Il23a*	ns	0.778329
WT **vs** DC-*Shp2*$^{-/-}$ IMQ-*Il23a*	ns	0.778329
WT **vs** DC-*Shp2*$^{-/-}$ Sham-*Tnfa*	ns	0.817895
WT **vs** DC-*Shp2*$^{-/-}$ IMQ-*Tnfa*	ns	0.865842
WT **vs** DC-*Shp2*$^{-/-}$ Sham-*Il6*	ns	0.670059
WT **vs** DC-*Shp2*$^{-/-}$ IMQ-*Il6*	ns	0.644924
WT **vs** DC-*Shp2*$^{-/-}$ Sham-*Il1b*	ns	0.543732
WT **vs** DC-*Shp2*$^{-/-}$ IMQ-*Il1b*	ns	0.543732
WT **vs** DC-*Shp2*$^{-/-}$ Sham-*Ptpn11*	**	0.000769
WT **vs** DC-*Shp2*$^{-/-}$ IMQ-*Ptpn11*	**	0.000205

Figure 6

Fig 6C		
Tukey multiple comparison test	Summary	*q* value
WT **vs** M-*Shp2*$^{-/-}$ medium-*Il23a*	ns	0.890714
WT **vs** M-*Shp2*$^{-/-}$ IMQ-*Il23a*	**	0.005604
WT **vs** M-*Shp2*$^{-/-}$ medium-*Il6*	ns	0.555227
WT **vs** M-*Shp2*$^{-/-}$ IMQ-*Il6*	**	0.005616
WT **vs** M-*Shp2*$^{-/-}$ medium-*Il1b*	ns	0.890714
WT **vs** M-*Shp2*$^{-/-}$ IMQ-*Il1b*	**	0.000286
WT **vs** M-*Shp2*$^{-/-}$ medium-*Il1a*	ns	0.555227
WT **vs** M-*Shp2*$^{-/-}$ IMQ-*Il1a*	**	0.003306
WT **vs** M-*Shp2*$^{-/-}$ medium-*Il1rn*	ns	0.890714
WT **vs** M-*Shp2*$^{-/-}$ IMQ-*Il1rn*	**	0.005514
WT **vs** M-*Shp2*$^{-/-}$ medium-*Ptgs2*	ns	0.555227
WT **vs** M-*Shp2*$^{-/-}$ IMQ-*Ptgs2*	**	0.004453
WT **vs** M-*Shp2*$^{-/-}$ medium-*Csf2*	ns	0.890714
WT **vs** M-*Shp2*$^{-/-}$ IMQ-*Csf2*	**	0.005514

Figure 8

Fig 8D		
Two-tailed unpaired Student's *t*-test	Summary	*p* value
WT **vs** Y897A	**	<0.0001
WT **vs** 972A	**	<0.0001
WT **vs** 1024A	**	<0.0001

Fig 8G		
Two-tailed unpaired Student's *t*-test	Summary	*p* value

WT **vs** Y897D	ns	0.1135
WT **vs** 972D	ns	0.0647
WT **vs** 1024D	**	<0.0001

Figure 9

Fig 9A		
Two-tailed Mann-Whitney *U* test	Summary	*p* value
wt/wt **vs** ki/wt	**	0.001
ki/wt **vs** ki/ki	**	0.0028
wt/wt **vs** ki/ki	**	<0.0001
Fig 9B		
Tukey multiple comparison test	Summary	*q* value
wt/wt **vs** ki/wt-Day 4	ns	0.089258
wt/wt **vs** ki/ki-Day 4	*	0.038387
wt/wt **vs** ki/wt-Day 5	*	0.048012
wt/wt **vs** ki/ki-Day 5	*	0.038387
Fig 9C		
Tukey multiple comparison test	Summary	*q* value
wt/wt **vs** ki/wt-Day 3	ns	0.591695
wt/wt **vs** ki/ki-Day 3	*	0.027234
wt/wt **vs** ki/wt-Day 4	**	0.008935
wt/wt **vs** ki/ki-Day 4	**	0.000336
wt/wt **vs** ki/wt-Day 5	**	0.003667
wt/wt **vs** ki/ki-Day 5	**	0.000447
Fig 9D		
Two-tailed Mann-Whitney *U* test	Summary	*p* value
wt/wt **vs** ki/wt-*Il23a*	**	0.0003
wt/wt **vs** ki/ki-*Il23a*	**	0.001
wt/wt **vs** ki/wt-*Il17a*	*	0.0426
wt/wt **vs** ki/ki-*Il17a*	*	0.0412
wt/wt **vs** ki/wt-*Il22*	*	0.0362
wt/wt **vs** ki/ki-*Il22*	*	0.0431
wt/wt **vs** ki/wt-*Tnfa*	*	0.0189
wt/wt **vs** ki/ki-*Tnfa*	*	0.0128
wt/wt **vs** ki/wt-*Il6*	*	0.0166
wt/wt **vs** ki/ki-*Il6*	*	0.0272
wt/wt **vs** ki/wt-*Il1b*	*	0.0436
wt/wt **vs** ki/ki-*Il1b*	ns	0.0595

Figure EV1

Figure EV1A		
Tukey multiple comparison test	Summary	*q* value
rmIL-23 **vs** rmIL-23+SHP099 Day 2	ns	0.082054
rmIL-23 **vs** rmIL-23+SHP099 Day 3	ns	0.058563

rmIL-23 **vs** rmIL-23+SHP099 Day 4	**	0.001495
rmIL-23 **vs** rmIL-23+SHP099 Day 5	**	0.000027

Figure EV1B		
Tukey multiple comparison test	Summary	*q* value
PBS **vs** SHP099 PBS	ns	0.446796
PBS **vs** SHP099 rmIL-23	**	0.000027

Figure EV1C		
Tukey multiple comparison test	Summary	*q* value
PBS **vs** SHP099-PBS-*Il23a*	ns	0.989013
PBS **vs** SHP099-rmIL-23-*Il23a*	*	0.038568
PBS **vs** SHP099-PBS-*Il17a*	ns	0.886659
PBS **vs** SHP099-rmIL-23-*Il17a*	*	0.043542
PBS **vs** SHP099-PBS-*Il22*	ns	0.193275
PBS **vs** SHP099-rmIL-23-*Il22*	ns	0.36238
PBS **vs** SHP099-PBS-*Tnfa*	ns	0.819002
PBS **vs** SHP099-rmIL-23-*Tnfa*	ns	0.819002
PBS **vs** SHP099-PBS-*Il6*	ns	0.489505
PBS **vs** SHP099-rmIL-23-*Il6*	ns	0.489505
PBS **vs** SHP099-PBS-*Il1b*	ns	0.411086
PBS **vs** SHP099-rmIL-23-*Il1b*	ns	0.411086

Figure EV2

Figure EV2B		
Tukey multiple comparison test	Summary	*q* value
IMQ **vs** IMQ+SHP099-*Il23a*	**	0.000885
IMQ **vs** IMQ+SHP099-*Il6*	**	0.000015
IMQ **vs** IMQ+SHP099-*Il1b*	**	0.000006
IMQ **vs** IMQ+SHP099-*Il1a*	**	0.000132
IMQ **vs** IMQ+SHP099-*Il1rn*	**	0.000015
IMQ **vs** IMQ+SHP099-*Cox2*	**	0.000091
IMQ **vs** IMQ+SHP099-*Csf2*	**	0.000074
IMQ **vs** IMQ+SHP099-*Nfkb2*	*	0.004634
IMQ **vs** IMQ+SHP099-*Bcl3*	**	0.000445

Figure EV2C		
Tukey multiple comparison test	Summary	*q* value
IMQ **vs** IMQ+SHP099-*Ccl9*	**	0.001656
IMQ **vs** IMQ+SHP099-*Ccl7*	**	0.000018
IMQ **vs** IMQ+SHP099-*Ccl2*	**	0.000188
IMQ **vs** IMQ+SHP099-*Adora2b*	**	0.000023
IMQ **vs** IMQ+SHP099-*Tarm1*	**	0.000897
IMQ **vs** IMQ+SHP099-*Gm6377*	**	0.002053
IMQ **vs** IMQ+SHP099-*Cish*	**	0.000897
IMQ **vs** IMQ+SHP099-*Hbegf*	**	0.000897
IMQ **vs** IMQ+SHP099-*Flrt3*	**	0.005406

IMQ **vs** IMQ+SHP099-*Osm*	**	0.000897
IMQ **vs** IMQ+SHP099-*Sphk1*	*	0.011377
IMQ **vs** IMQ+SHP099-*Srgn*	**	0.004104

Figure EV4

Figure EV4A		
Two-tailed unpaired Student's *t*-test	Summary	*p* value
wt **vs** ki	**	<0.0001
**Figure EV4B**		
Bonferroni multiple comparison test	Summary	*q* value
wt **vs** ki-Day 5	*	0.044182
wt **vs** ki-Day 6	*	0.013767
**Figure EV4C**		
Bonferroni multiple comparison test	Summary	*q* value
wt **vs** ki-Day 4	*	0.011577
wt **vs** ki-Day 5	**	0.004169
wt **vs** ki-Day 6	**	0.002025
**Figure EV4D**		
Two-tailed unpaired Student's *t*-test	Summary	*p* value
wt **vs** ki-*Il23a*	*	0.0109
wt **vs** ki-*Il17a*	**	0.0066
wt **vs** ki-*Il22*	**	0.006
wt **vs** ki-*Tnfa*	*	0.0285
wt **vs** ki-*Il6*	*	0.0285
wt **vs** ki-*Il1b*	*	0.038

Figure EV5

Figure EV5A		
Two-tailed unpaired Student's *t*-test	Summary	*p* value
PBS **vs** SHP099	ns	0.6066
**Figure EV5D**		
Two-tailed unpaired Student's *t*-test	Summary	*p* value
PBS **vs** SHP099-*Il23a*	ns	0.76
PBS **vs** SHP099-*Il17a*	ns	0.4905
PBS **vs** SHP099-*Il22*	ns	0.2805
**Figure EV5E**		
Two-tailed unpaired Student's *t*-test	Summary	*p* value
PBS **vs** SHP099-*Tnfa*	ns	0.554
PBS **vs** SHP099-*Il6*	ns	0.8024
PBS **vs** SHP099-*Il1b*	ns	0.873

Appendix Fig S1

Appendix Fig S1		
Two-tailed unpaired Student's *t*-test	Summary	*p* value

medium **vs** IMQ-*IL23A*	**	<0.0001
IMQ **vs** IMQ+SHP099-*IL23A*	**	0.001
medium **vs** IMQ-*TNFA*	**	<0.0001
IMQ **vs** IMQ+SHP099-*TNFA*	*	0.0136
medium **vs** IMQ-*IL6*	**	<0.0001
IMQ **vs** IMQ+SHP099-*IL6*	**	0.0001
medium **vs** IMQ-*IL1B*	**	<0.0001
IMQ **vs** IMQ+SHP099-*IL1B*	**	0.0002

Appendix Fig S2

Appendix Fig S2		
Two-tailed unpaired Student's *t*-test	Summary	*p* value
medium **vs** SHP099-*IL23A*	ns	0.2898
medium **vs** SHP099-*TNFA*	ns	0.2832
medium **vs** SHP099-*IL6*	ns	0.0938
medium **vs** SHP099-*IL1B*	ns	0.0611

Appendix Fig S3

Appendix Fig S3A		
Two-tailed unpaired Student's *t*-test	Summary	*p* value
Sham **vs** SHP099	ns	0.9403
Appendix Fig S3D		
Two-tailed unpaired Student's *t*-test	Summary	*p* value
Sham **vs** SHP099-*Il23a*	ns	0.8929
Sham **vs** SHP099-*Il17a*	ns	0.815
Sham **vs** SHP099-*Il22*	ns	0.798
Appendix Fig S3E		
Two-tailed unpaired Student's *t*-test	Summary	*p* value
Sham **vs** SHP099-*Tnfa*	ns	0.9273
Sham **vs** SHP099-*Il6*	ns	0.0934
Sham **vs** SHP099-*Il1b*	ns	0.7562
Sham **vs** SHP099-*Ptpn11*	ns	0.0513

Appendix Fig S11

Appendix Fig S11A		
Two-tailed unpaired Student's *t*-test	Summary	*p* value
WT **vs** M-*Shp2*$^{-/-}$ *Il23a*	**	0.0038
WT **vs** M-*Shp2*$^{-/-}$ *Tnfa*	**	0.0002
WT **vs** M-*Shp2*$^{-/-}$ *Il6*	**	<0.0001
WT **vs** M-*Shp2*$^{-/-}$ *Il1b*	**	<0.0001
Appendix Fig S11B		
Two-tailed unpaired Student's *t*-test	Summary	*p* value
WT **vs** M-*Shp2*$^{-/-}$ *Il23a*	**	<0.0001
WT **vs** M-*Shp2*$^{-/-}$ *Tnfa*	**	0.0002
WT **vs** M-*Shp2*$^{-/-}$ *Il6*	**	<0.0001

WT **vs** M-*Shp2*$^{-/-}$ *Il1b*	**	<0.0001

Appendix Fig S11C		
Tukey multiple comparison test	Summary	*q* value
sh-Ctrl **vs** sh-SHP2-*IL23A* IMQ	**	<0.000001
sh-Ctrl **vs** sh-SHP2-*TNFA* IMQ	**	<0.000001
sh-Ctrl **vs** sh-SHP2-*IL6* IMQ	**	0.000087
sh-Ctrl **vs** sh-SHP2-*IL1B* IMQ	**	0.000005
sh-Ctrl **vs** sh-SHP2-*PTPN11* IMQ	**	0.004046

Appendix Fig S11D		
Two-tailed unpaired Student's *t*-test	Summary	*p* value
WT **vs** M-*Shp2*$^{-/-}$ *Il23a*	**	<0.0001
WT **vs** M-*Shp2*$^{-/-}$ *Tnfa*	*	0.0084
WT **vs** M-*Shp2*$^{-/-}$ *Il6*	**	0.0014
WT **vs** M-*Shp2*$^{-/-}$ *Il1b*	**	0.0025
WT **vs** M-*Shp2*$^{-/-}$ *Ptpn11*	**	<0.0001

Appendix Fig S14

Appendix Fig S14G		
Fisher's exact test	Summary	*p* value
Normal	**	7.623e-23
Psoriasis	**	4.542e-49

Appendix Fig S18

Appendix Fig S18A		
Tukey multiple comparison test	Summary	*q* value
WT **vs** M-*Shp2*$^{-/-}$ *Il23a* R848	**	0.000938
WT **vs** M-*Shp2*$^{-/-}$ *Tnfa* R848	**	0.004976
WT **vs** M-*Shp2*$^{-/-}$ *Il6* R848	**	0.001162

Appendix Fig S22

Appendix Fig S22A		
Tukey multiple comparison test	Summary	*q* value
rmIL-23 **vs** rmIL-23+SHP099 Day 2	ns	0.182689
rmIL-23 **vs** rmIL-23+SHP099 Day 3	ns	0.182689
rmIL-23 **vs** rmIL-23+SHP099 Day 4	*	0.040728
rmIL-23 **vs** rmIL-23+SHP099 Day 5	*	0.040728

Appendix Fig S22B		
Tukey multiple comparison test	Summary	*q* value
PBS **vs** SHP099 PBS	ns	0.497776
PBS **vs** SHP099 rmIL-23	**	0.000365

Appendix Fig S22C		
Tukey multiple comparison test	Summary	*q* value
PBS **vs** SHP099-PBS-*Il23a*	ns	0.294604
PBS **vs** SHP099-rmIL-23-*Il23a*	ns	0.070372
PBS **vs** SHP099-PBS-*Il17a*	ns	0.49664

PBS **vs** SHP099-rmIL-23-*Il17a*	*	0.043326
PBS **vs** SHP099-PBS-*Il22*	ns	0.799866
PBS **vs** SHP099-rmIL-23-*Il22*	ns	0.071587
PBS **vs** SHP099-PBS-*Tnfa*	ns	0.248397
PBS **vs** SHP099-rmIL-23-*Tnfa*	ns	0.248397
PBS **vs** SHP099-PBS-*Il6*	ns	0.744017
PBS **vs** SHP099-rmIL-23-*Il6*	ns	0.554322
PBS **vs** SHP099-PBS-*Il1b*	ns	0.501075
PBS **vs** SHP099-rmIL-23-*Il1b*	ns	0.501075
