# Supplementary material for: Allosteric inhibition of SHP2 uncovers aberrant TLR7 trafficking in aggravating psoriasis
Source: EMBO Mol Med. 2021 Dec 22;14(3):e14455. doi: 10.15252/emmm.202114455 (PMC8899919; doi:10.15252/emmm.202114455)
Supplement: Supplementary file 2 — Expanded View Figures PDF [file EMMM-14-e14455-s006.pdf]

Expanded View Figures

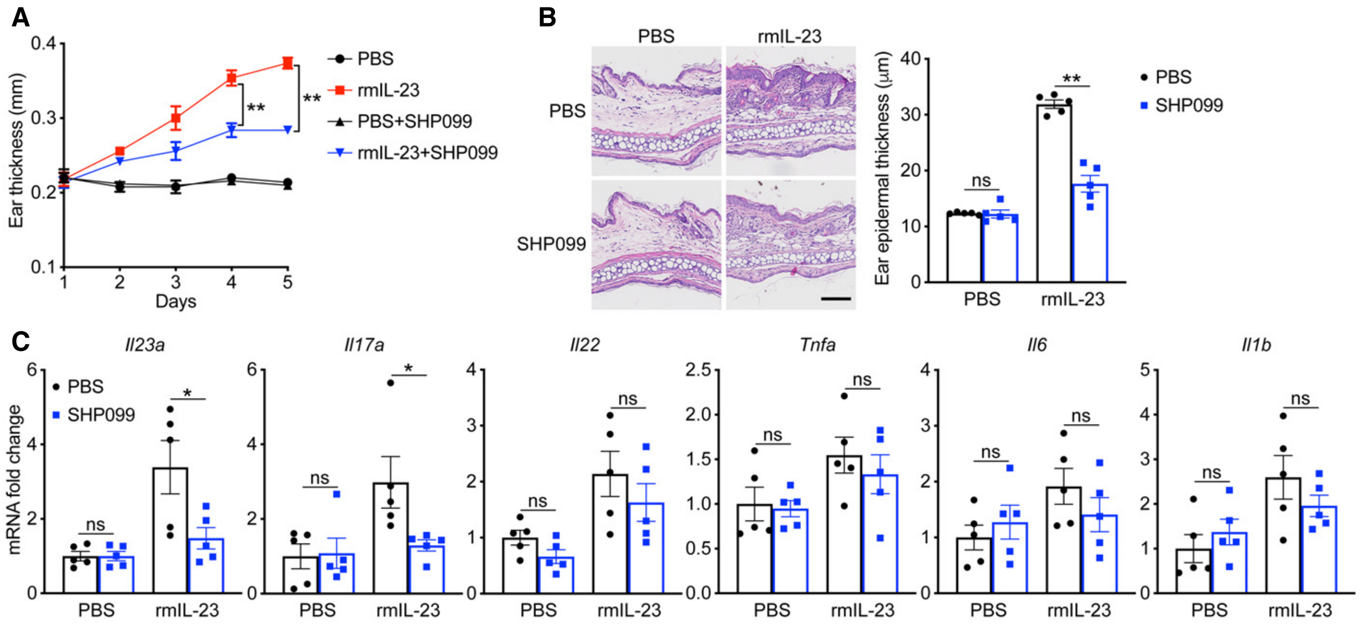

Figure EV1. SHP099 attenuated the psoriasis-like phenotype in the IL-23-induced murine model.

C57BL/6 mice (n = 5/group) were subjected to IL-23-induced psoriasis-like skin inflammation and were treated with 10 mg/kg SHP099 or PBS for 4 days.

A, B Ear thickness (A), H&E staining, and statistic results (B) of ear skin from SHP099 treated with or without C57BL/6 mice injected intradermally with rmIL-23 or PBS for 4 days. Ear thickness was measured daily.

C Quantitative PCR analysis of mRNA encoding IL-23/IL-17A axis cytokines and other psoriasis-related cytokines in the ear skin. Results were normalized to *Gapdh* expression.

Data information: Data are represented as mean ± SEM. *P* values are determined by Tukey multiple-comparison test (A–C). *P < 0.05, **P < 0.01, ns, not significant. Source data are available online for this figure.

© 2021 The Authors

© 2021 The Authors

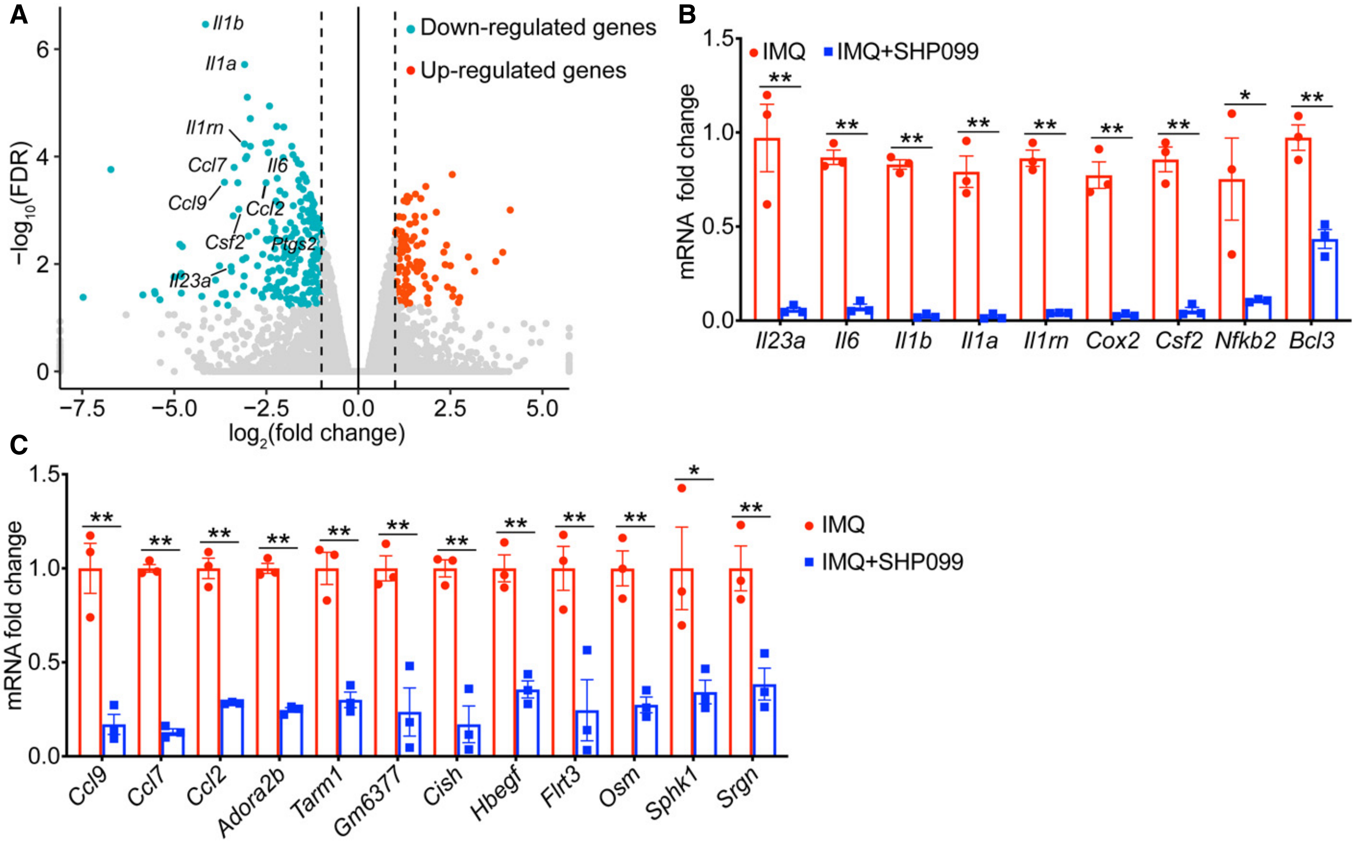

Figure EV2. SHP2-allosteric inhibitor SHP099 prevented psoriasis by downregulating NF-κB activation.

A Volcano plot image of upregulated genes (red) and downregulated genes (green) (treated with SHP099 compared to untreated with SHP099) from peritoneal macrophages derived from C57BL/6 mice untreated or pretreated with SHP099 (10 μM) for 2 h and then stimulated by IMQ (10 μg/ml) for 4 h (n = 3/group).

B, C Peritoneal macrophages derived from C57BL/6 mice were untreated or pretreated with SHP099 (10 μM) for 2 h and then stimulated by IMQ (10 μg/ml) for 4 h. Expression levels of indicated genes decreased in the SHP099 group compared to medium control.

Data information: Data are represented as mean ± SEM. P values are determined by Tukey multiple-comparison test (B, C). $^*P < 0.05$, $^{**}P < 0.01$.
Source data are available online for this figure.

 © 2021 The Authors

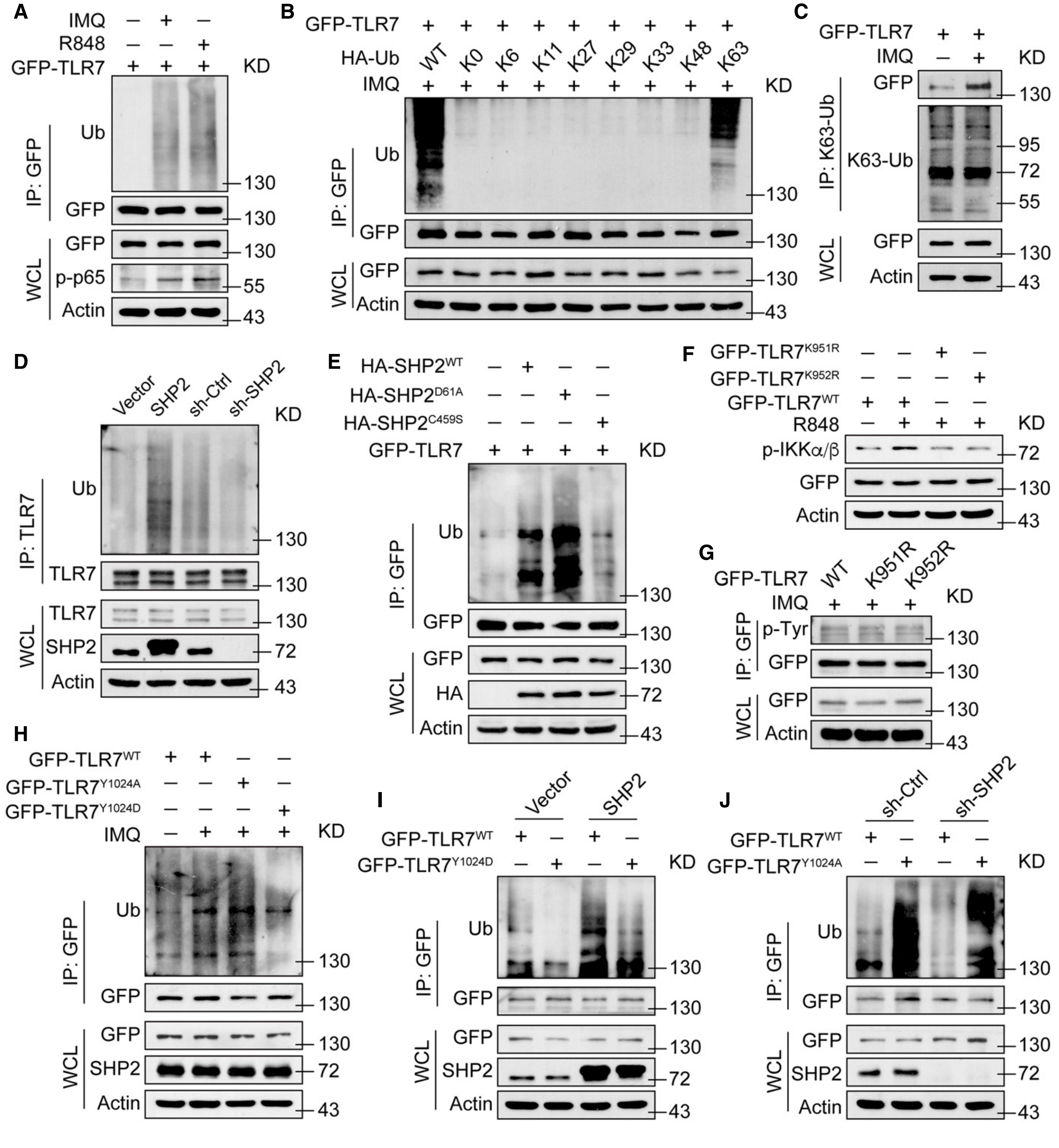

A
IMQ
R848
GFP-TLR7
KD
Ub
IP: GFP
GFP
WCL
p-p65
Actin
B
GFP-TLR7
HA-Ub
WT K0 K6 K11 K27 K29 K33 K48 K63
C
K63-Ub
IP: K63-Ub
D
Vector SHP2 sh-Ctrl sh-SHP2
IP: TLR7
TLR7
SHP2
E
HA-SHP2WT
HA-SHP2D61A
HA-SHP2C459S
HA
F
GFP-TLR7K951R
GFP-TLR7K952R
GFP-TLR7WT
p-IKKα/β
G
WT K951R K952R
p-Tyr
H
GFP-TLR7Y1024A
GFP-TLR7Y1024D
I
J

Figure EV3.

◀ **Figure EV3. SHP2 mediated the ubiquitination of TLR7 via dephosphorylating its Y1024.**

A Immunoblotting of TLR7 ubiquitination in HEK293T cells transfected with GFP-TLR7 for 48 h, then treated with IMQ (10 μg/ml) or R848 (10 μg/ml) for 1 h.
B Immunoblotting of TLR7 ubiquitination in HEK293T cells co-transfected with GFP-TLR7, along with HA-Ub or mutant Ubs and treated with IMQ (10 μg/ml) for 1 h.
C Immunoblotting of HEK293T cells transfected with GFP-TLR7 and treated with or without IMQ (10 μg/ml) for 30 min.
D Immunoblotting of TLR7 ubiquitination in THP1 cells with vector, SHP2, shRNA-Control, or shRNA-SHP2 lentivirus was treated with IMQ (10 μg/ml) for 1 h.
E HEK293T cells co-transfected with GFP-TLR7 and HA-SHP2 and HA-SHP2 mutant vectors for 48 h, and then infected with IMQ (10 μg/ml) for 1 h.
F Immunoblotting of HEK293T cells transfected with GFP-TLR7 and TLR7 mutant vectors and treated with or without R848 (10 μg/ml) for 30 min.
G Immunoblotting of HEK293T cells transfected with GFP-TLR7 and TLR7 mutant vectors and treated with IMQ (10 μg/ml) for 30 min.
H Immunoblotting of HEK293T cells transfected with GFP-TLR7 and TLR7 mutant vectors and treated with or without IMQ (10 μg/ml).
I Immunoblotting of TLR7 ubiquitination in HEK293T cells with vector and SHP2 lentivirus transfected with GFP-TLR7 and GFP-TLR7^{Y1024D} plasmids and infected with IMQ (10 μg/ml) for 1 h.
J Immunoblotting of HEK293T cells with shRNA-Control or shRNA-SHP2 lentivirus transfected with GFP-TLR7 and GFP-TLR7^{Y1024A} plasmids and infected with IMQ (10 μg/ml) for 1 h.

Source data are available online for this figure.

 © 2021 The Authors

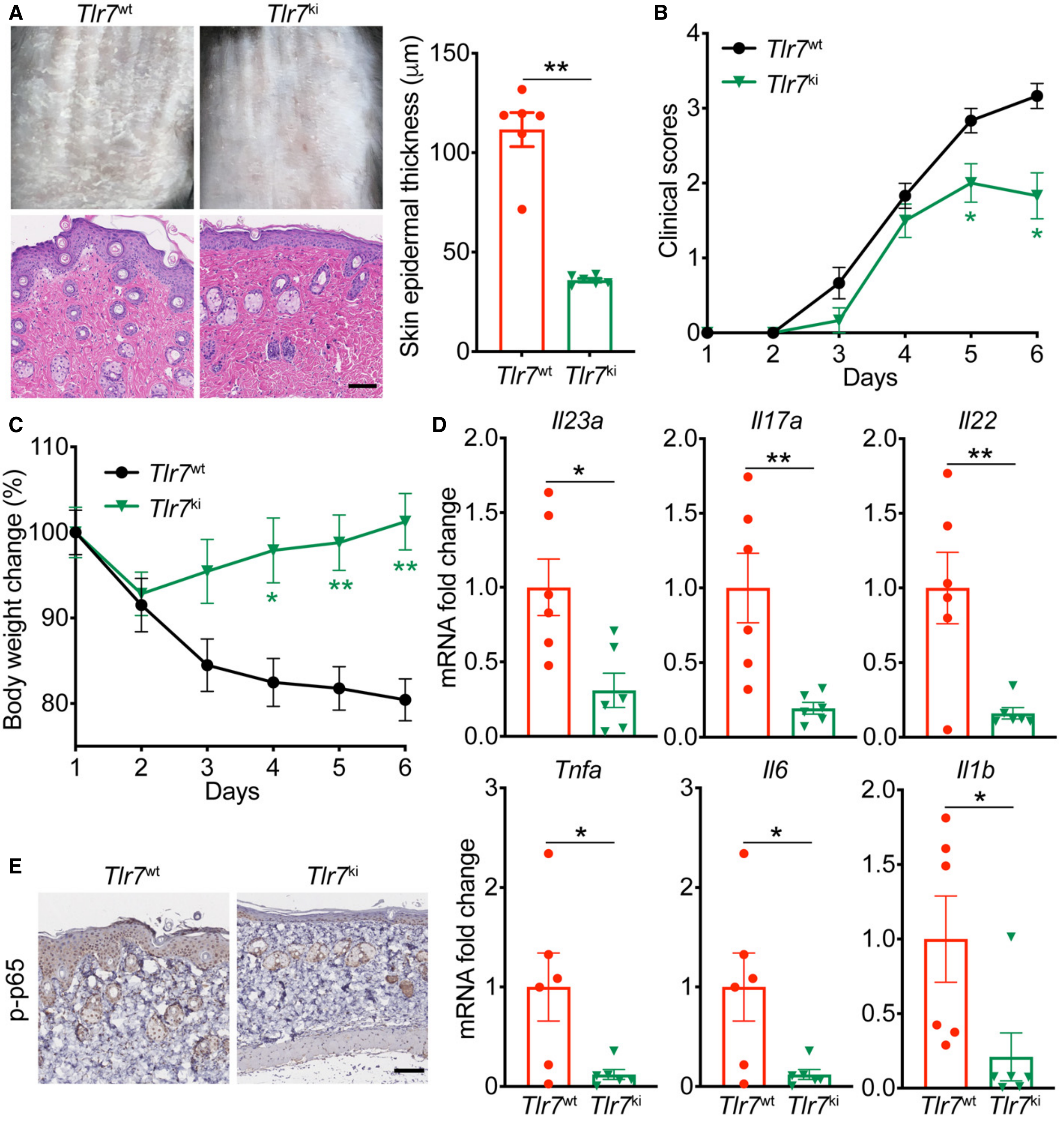
A
Tlr7wt
Tlr7ki
Skin epidermal thickness (μm)
150
100
50
0
**
Tlr7wt Tlr7ki
B
Clinical scores
Tlr7wt
Tlr7ki
*
*
Days
C
Body weight change (%)
Tlr7wt
Tlr7ki
*
**
**
Days
D
Il23a
Il17a
Il22
Tnfa
Il6
Il1b
mRNA fold change
*
**
**
*
*
*
Tlr7wt Tlr7ki
E
Tlr7wt
Tlr7ki
p-p65

Figure EV4.

Figure EV4. The IMQ-induced psoriasis-like phenotype was improved in *Tlr7*^{ki} mice.

Tlr7^{wt} male mice (n = 6) and *Tlr7*-Y1025D mutant *Tlr7*^{ki} male mice (n = 6) were treated with indicated dose of IMQ for 5 days.

A Phenotypic presentation (*top*) and H&E staining (*bottom*) of dorsal skin. Scale bar: 100 μm. Left: H&E staining data; right: statistical data (mean ± SEM).
B Clinical scores plotted with mean ± SEM. * Denotes statistical significance when compared with the *Tlr7*^{wt} group.
C Body weight change plotted with mean ± SEM. * Denotes statistical significance when compared with the *Tlr7*^{wt} group.
D Quantitative PCR analysis of mRNA encoding IL-23/IL-17A axis cytokines and other psoriasis-related cytokines in the dorsal skin. Results were normalized to *Gapdh* expression.
E Representative p-p65 staining of the dorsal skin. Scale bars: 100 μm.

Data information: Data are represented as mean ± SEM. *P*-values are determined by two-tailed unpaired Student's *t*-test (A, D) or Bonferroni multiple-comparison test (B, C). *$P < 0.05$, **$P < 0.01$.
Source data are available online for this figure.

© 2021 The Authors

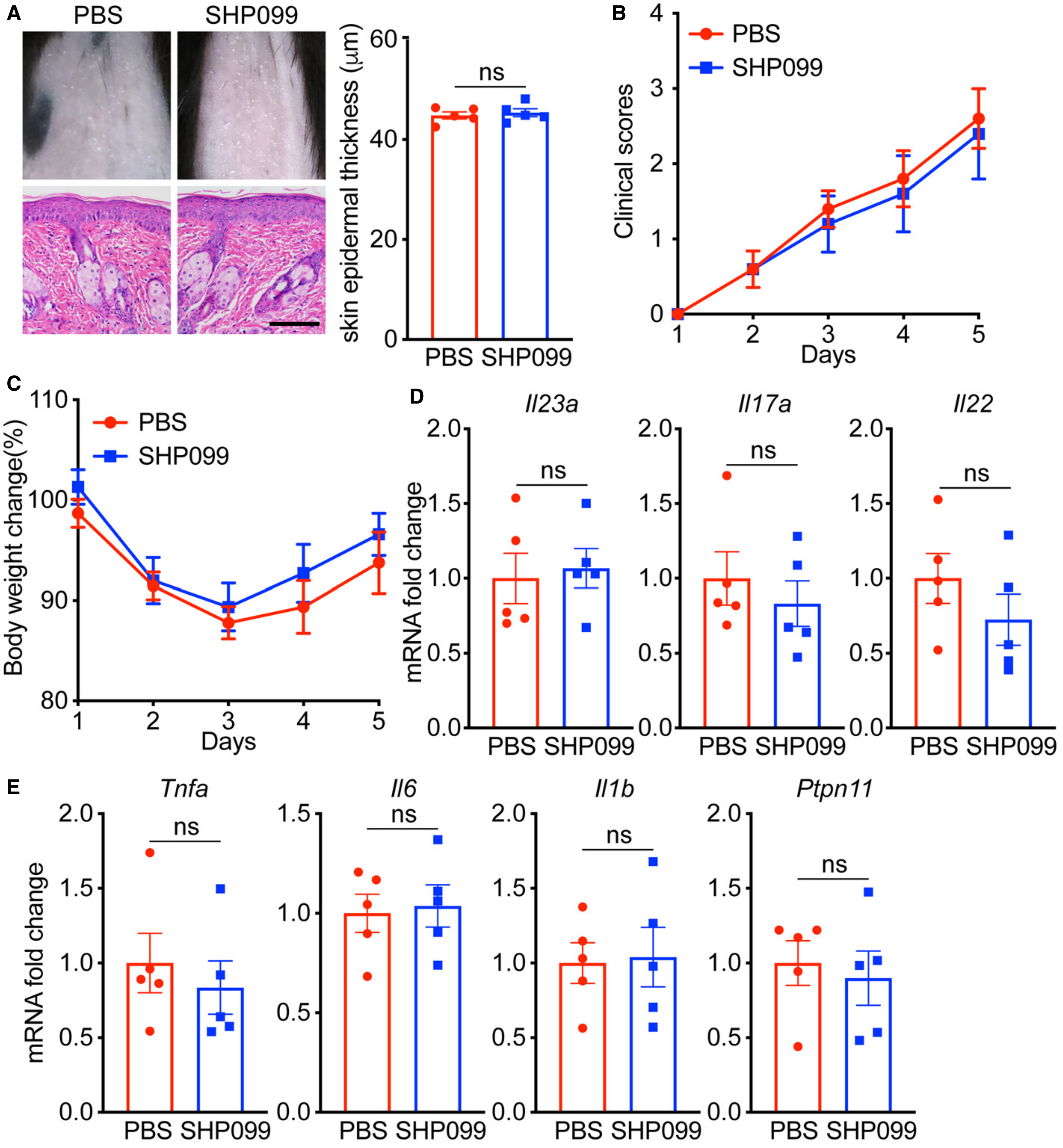
A
PBS
SHP099
skin epidermal thickness (μm)
60
40
20
0
ns
PBS SHP099
B
PBS
SHP099
Clinical scores
4
3
2
1
0
1 2 3 4 5
Days
C
PBS
SHP099
Body weight change(%)
110
100
90
80
1 2 3 4 5
Days
D
Il23a
Il17a
Il22
mRNA fold change
2.0
1.5
1.0
0.5
0.0
ns
PBS SHP099
E
Tnfa
Il6
Il1b
Ptpn11
mRNA fold change
ns
PBS SHP099

Figure EV5.

◀ **Figure EV5. SHP099 provided no benefit in the *Tlr7*-Y1025D mice treated with IMQ.**

Tlr7-Y1025D mutant *Tlr7*^{ki} male mice (n = 5/group) were treated with 10 mg/kg SHP099 or vehicle for 4 days.

A Phenotypic presentation (*top*) and H&E staining (*bottom*) of dorsal skin. Scale bar: 100 μm. Left: H&E staining data; right: statistical data (mean ± SEM).
B Clinical scores plotted with mean ± SEM.
C Body weight change plotted with mean ± SEM.
D, E Quantitative PCR analysis of mRNA encoding IL-23/IL-17A axis cytokines (D) and other psoriasis-related cytokines (E) in the dorsal skin. Results were normalized to *Gapdh* expression.

Data information: Data are represented as mean ± SEM. *P* values are determined by two-tailed Student's *t*-test (A, D, E) or Bonferroni multiple-comparison test (B, C). ns, not significant.
Source data are available online for this figure.

 © 2021 The Authors
