# Supplementary figures and images for: Allosteric inhibition of SHP2 uncovers aberrant TLR7 trafficking in aggravating psoriasis (part 1 of 2)
Source: EMBO Mol Med. 2021 Dec 22;14(3):e14455. doi: 10.15252/emmm.202114455 (PMC8899919; doi:10.15252/emmm.202114455)

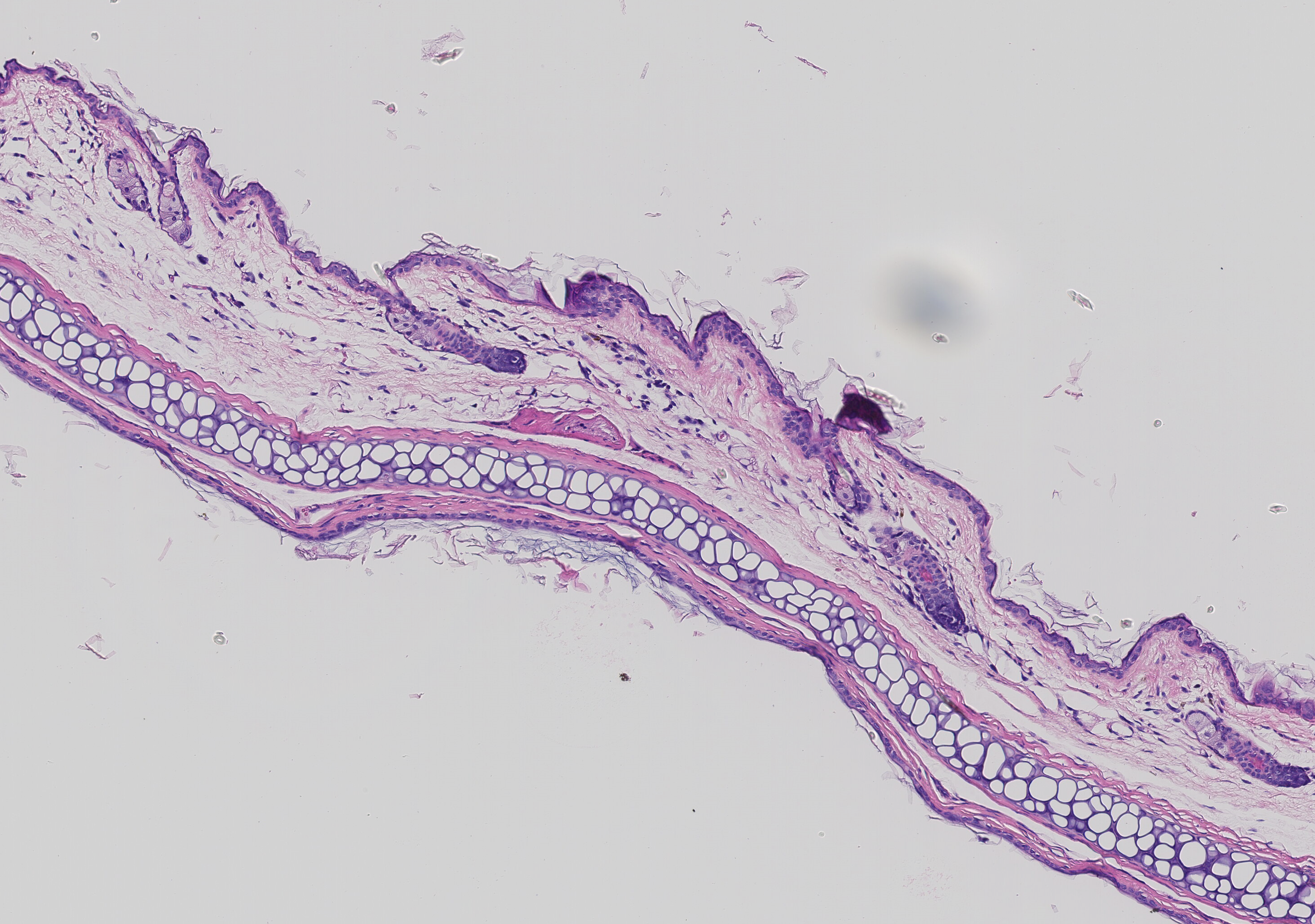

Supplement: Supplementary file 3 — Source Data for Expanded View [file EMMM-14-e14455-s002.zip › EMM-2021-14455_SourceDataForExpandedView/Fig_EV_1/EV1B/PBS.tiff]

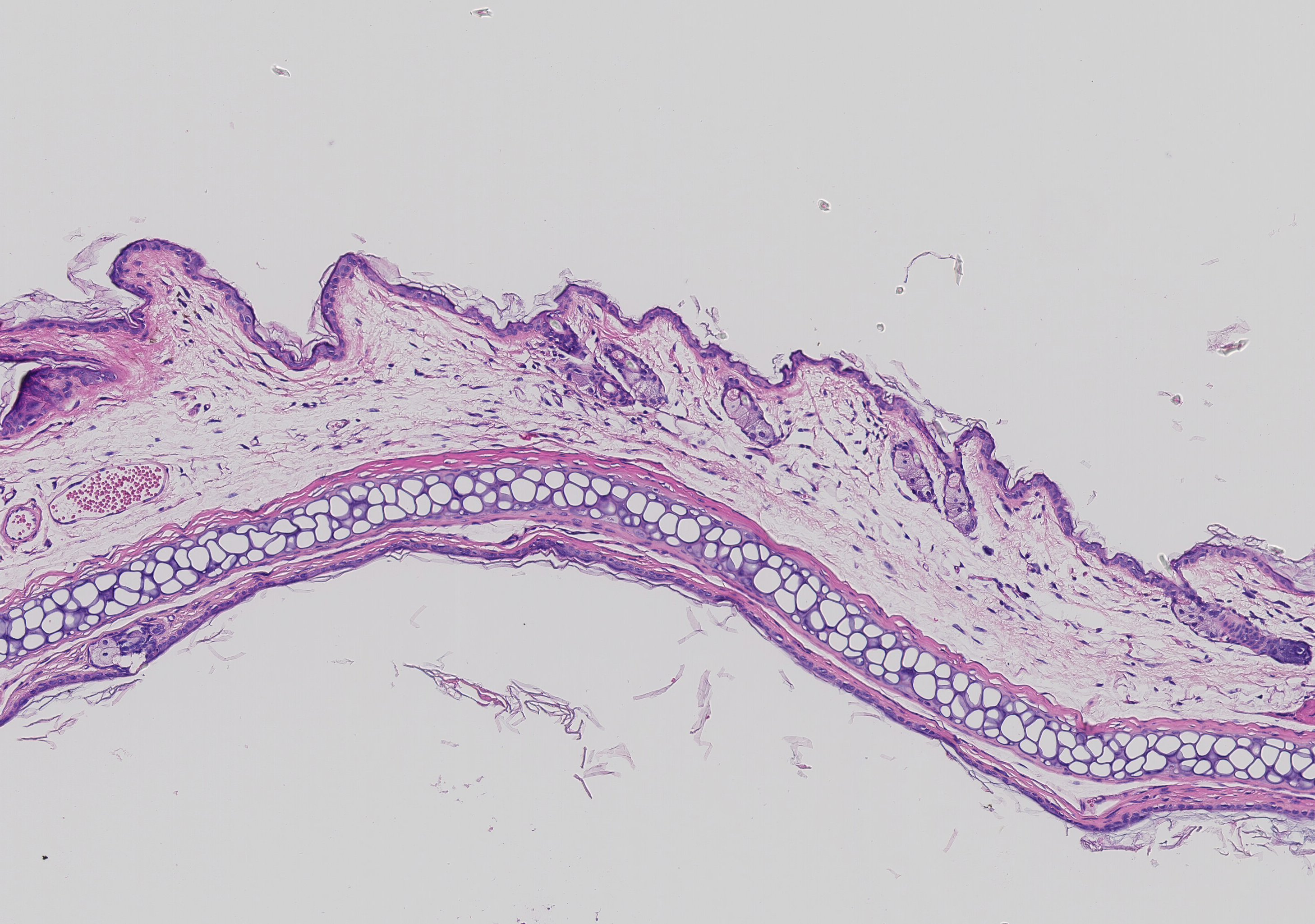

Supplement: Supplementary file 3 — Source Data for Expanded View [file EMMM-14-e14455-s002.zip › EMM-2021-14455_SourceDataForExpandedView/Fig_EV_1/EV1B/PBS_SHP099.tiff]

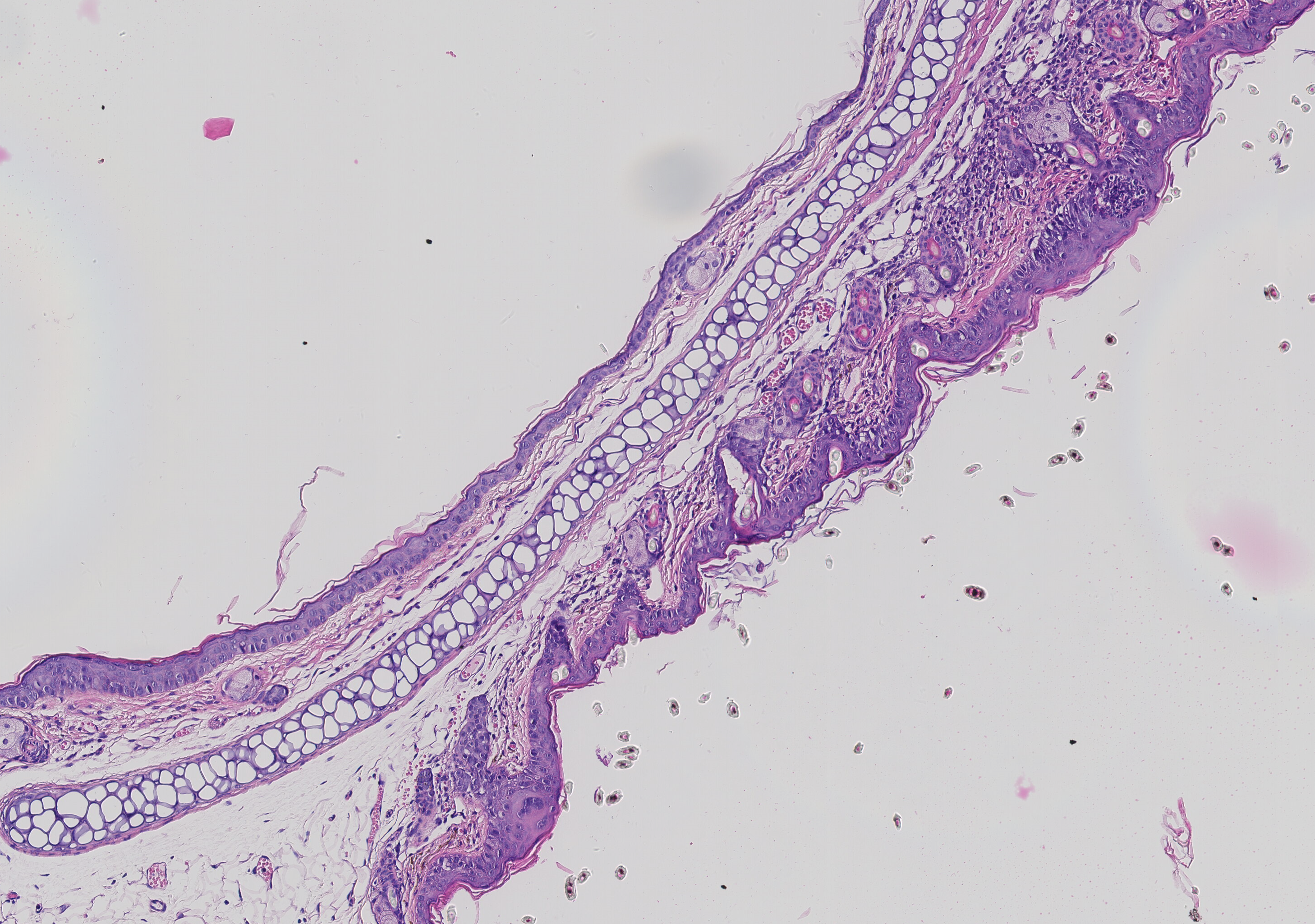

Supplement: Supplementary file 3 — Source Data for Expanded View [file EMMM-14-e14455-s002.zip › EMM-2021-14455_SourceDataForExpandedView/Fig_EV_1/EV1B/rmIL-23_PBS.tiff]

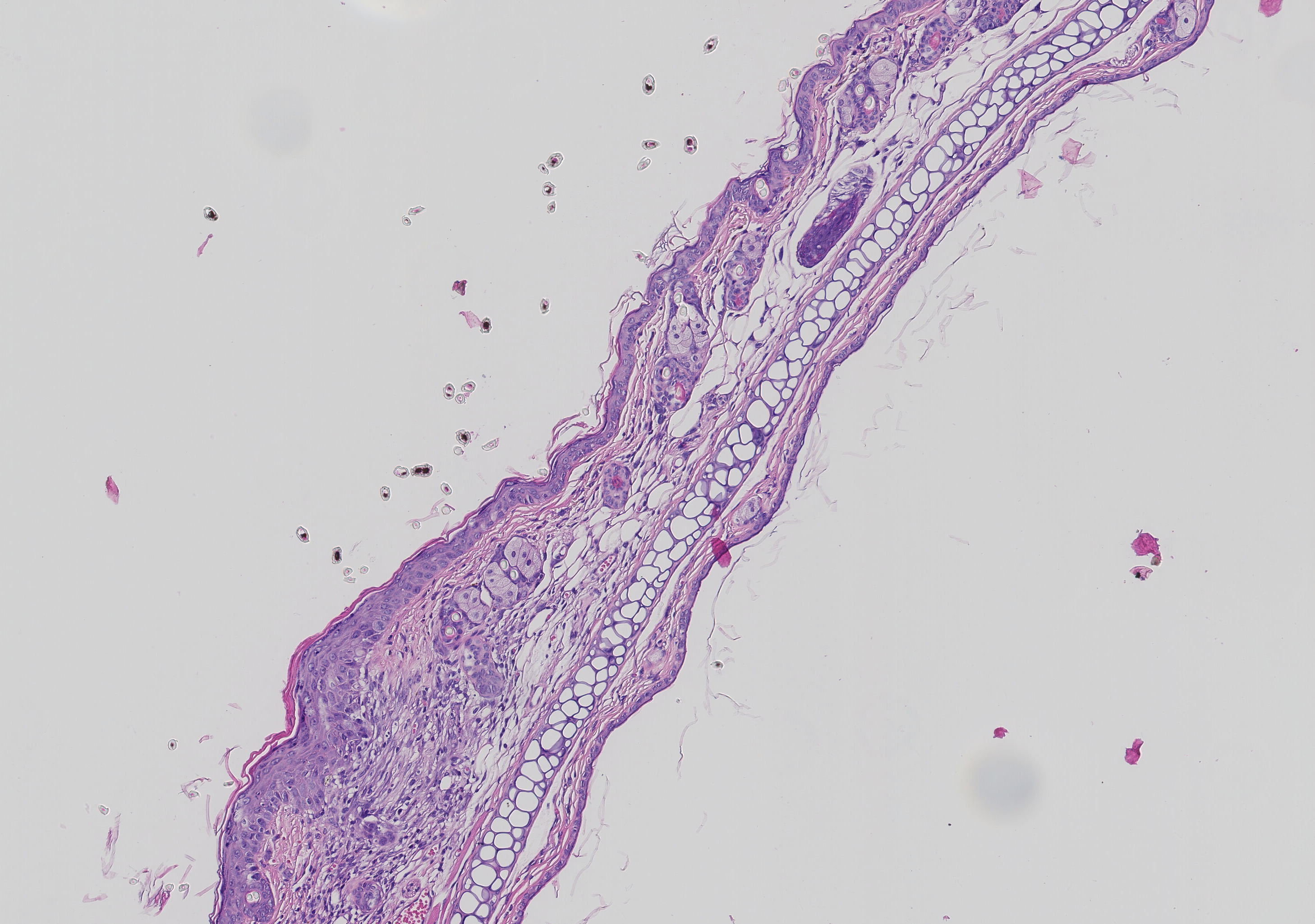

Supplement: Supplementary file 3 — Source Data for Expanded View [file EMMM-14-e14455-s002.zip › EMM-2021-14455_SourceDataForExpandedView/Fig_EV_1/EV1B/rmIL-23_SHP099.tiff]

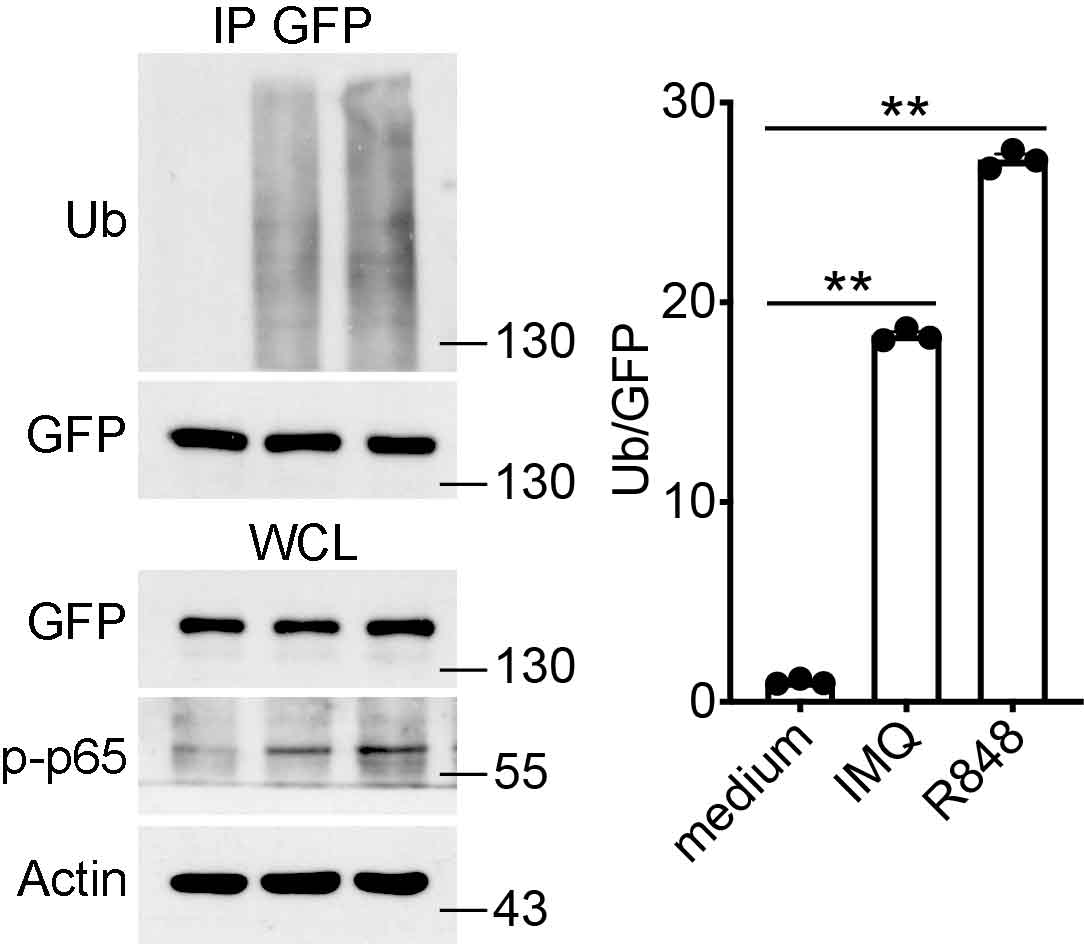

Supplement: Supplementary file 3 — Source Data for Expanded View [file EMMM-14-e14455-s002.zip › EMM-2021-14455_SourceDataForExpandedView/Fig_EV_3/EV3A/EV3A.jpg]

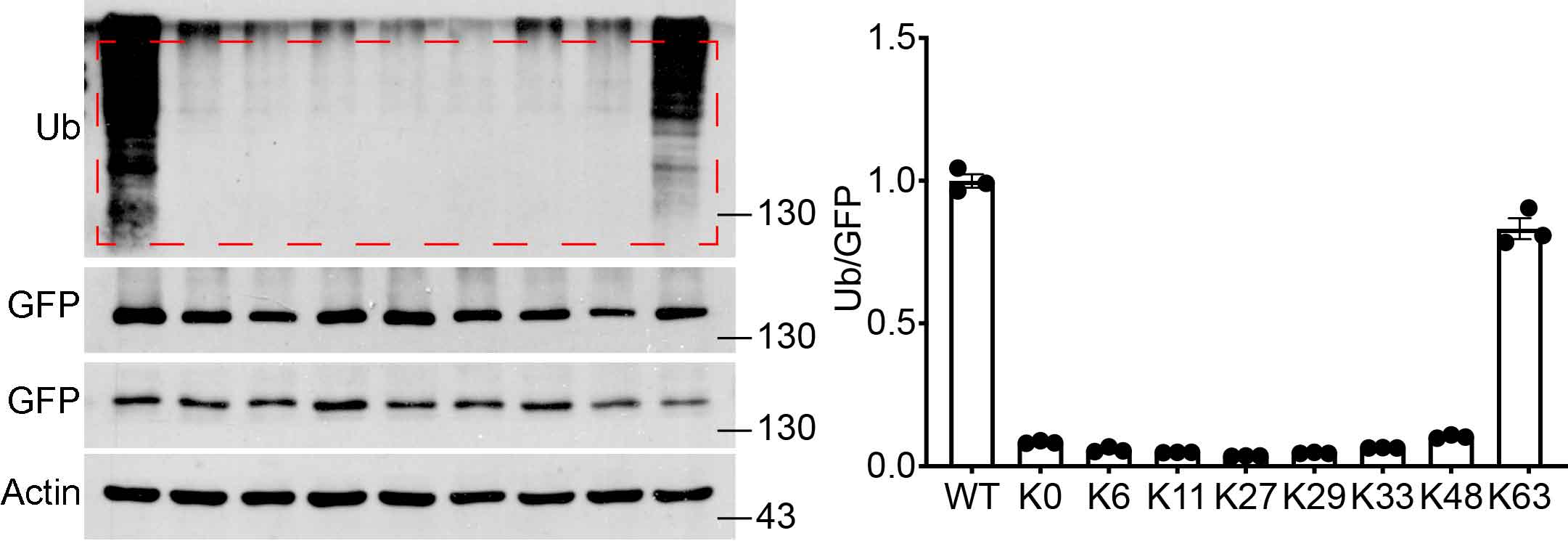

Supplement: Supplementary file 3 — Source Data for Expanded View [file EMMM-14-e14455-s002.zip › EMM-2021-14455_SourceDataForExpandedView/Fig_EV_3/EV3B/EV3B.jpg]

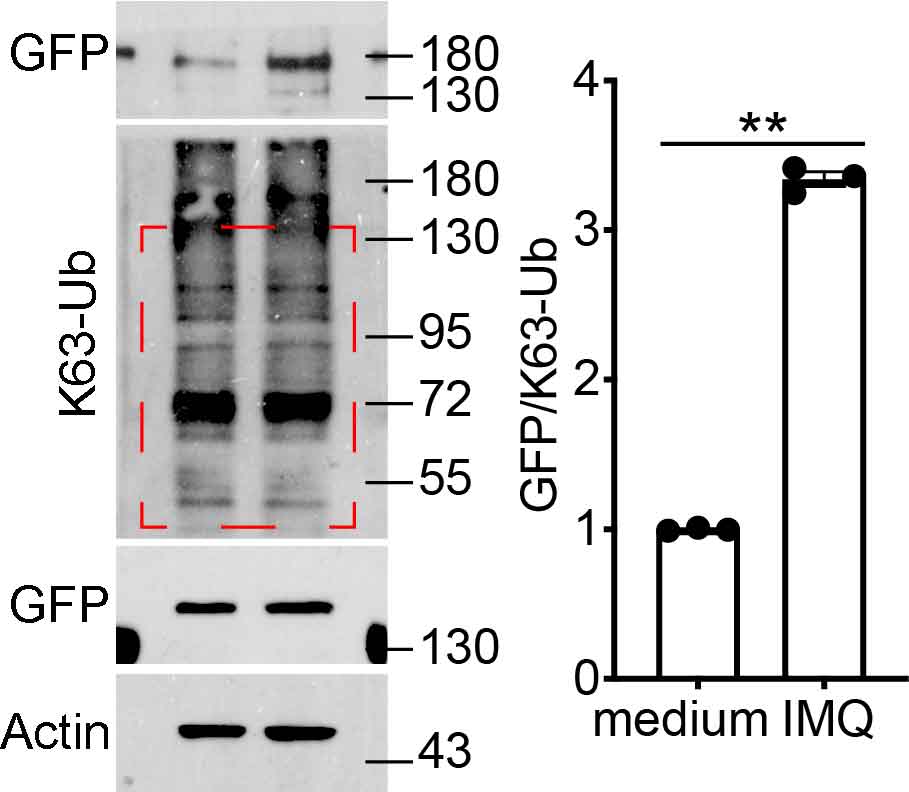

Supplement: Supplementary file 3 — Source Data for Expanded View [file EMMM-14-e14455-s002.zip › EMM-2021-14455_SourceDataForExpandedView/Fig_EV_3/EV3C/EV3C.jpg]

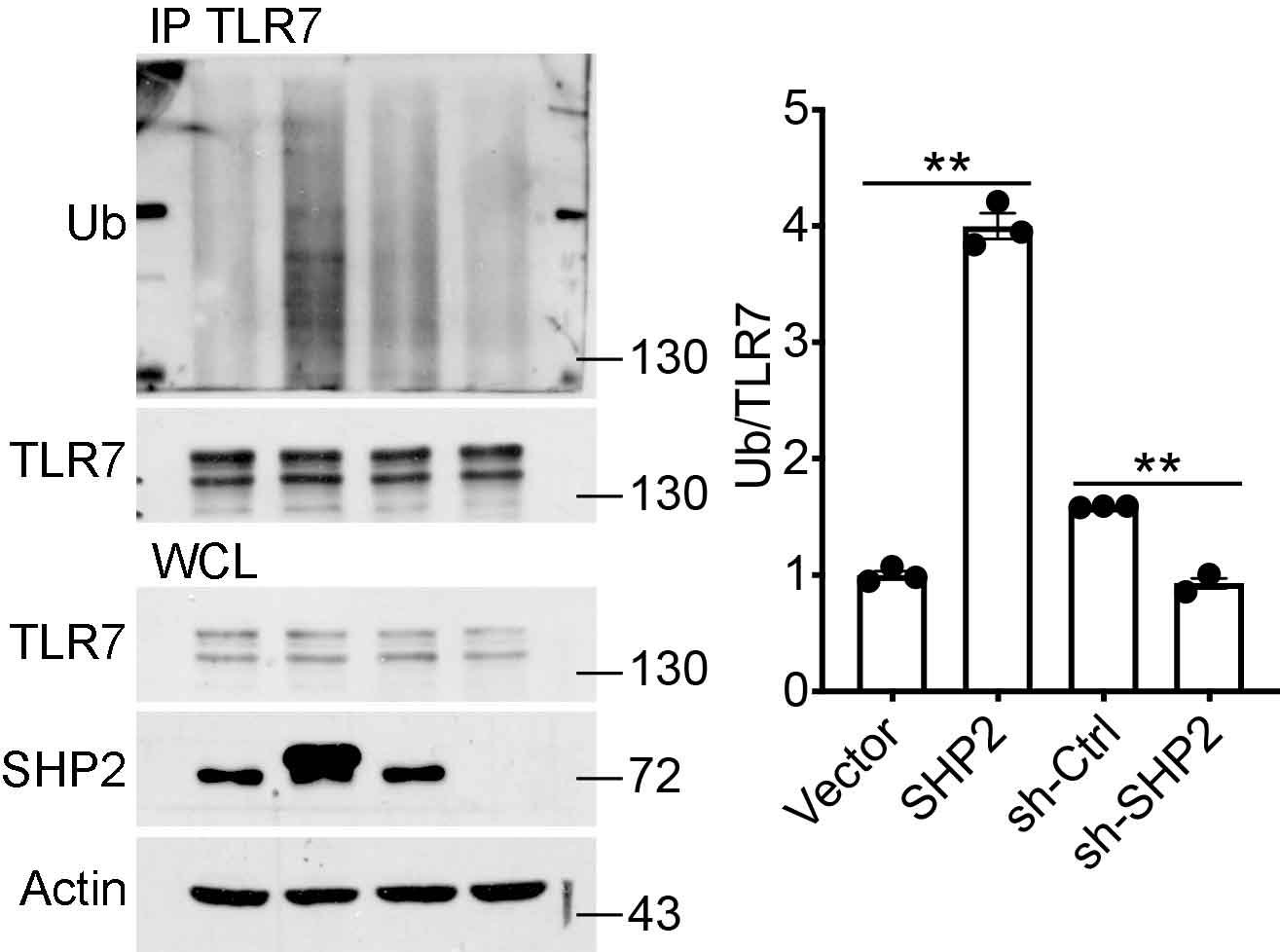

Supplement: Supplementary file 3 — Source Data for Expanded View [file EMMM-14-e14455-s002.zip › EMM-2021-14455_SourceDataForExpandedView/Fig_EV_3/EV3D/EV3D.jpg]

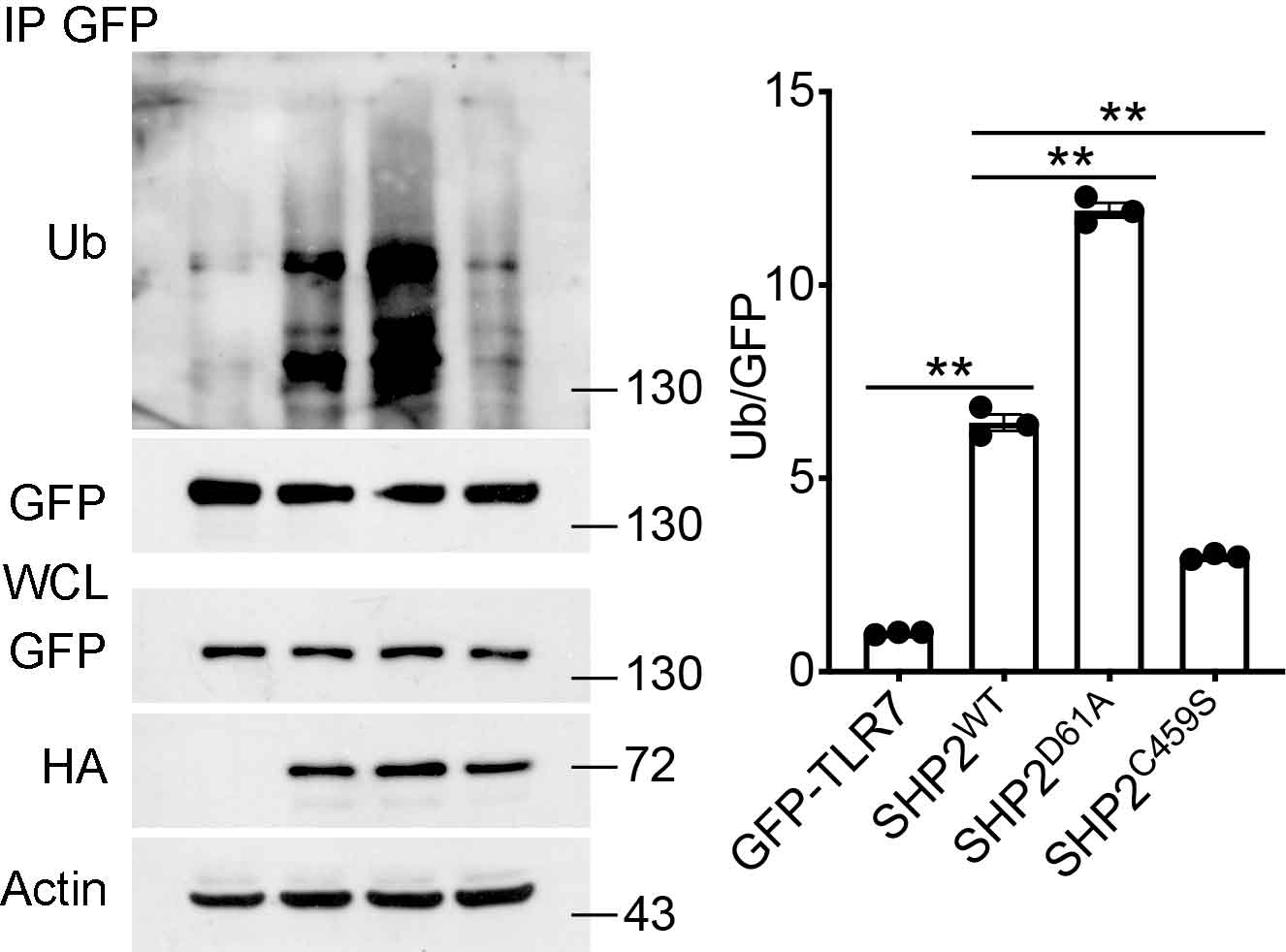

Supplement: Supplementary file 3 — Source Data for Expanded View [file EMMM-14-e14455-s002.zip › EMM-2021-14455_SourceDataForExpandedView/Fig_EV_3/EV3E/EV3E.jpg]

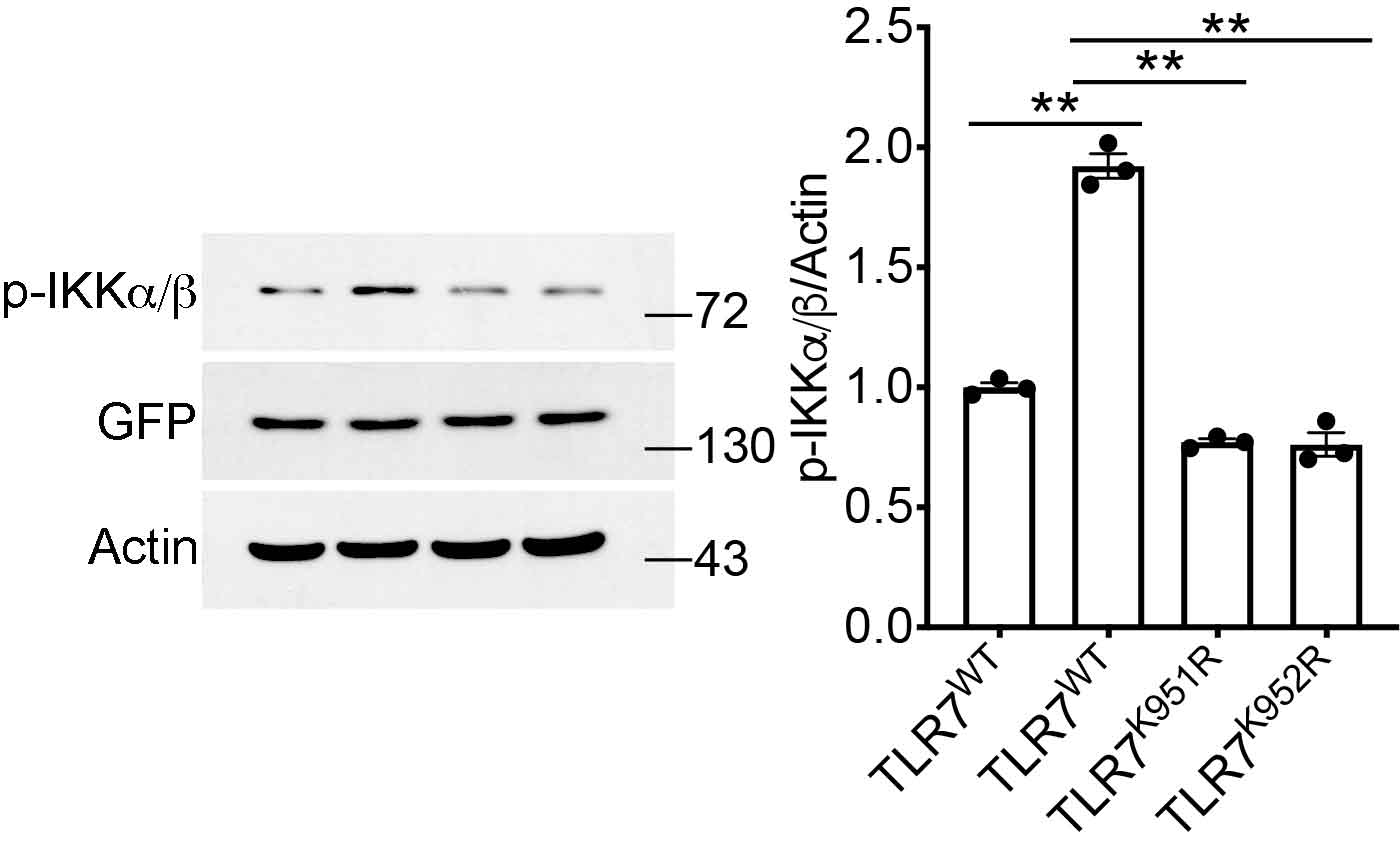

Supplement: Supplementary file 3 — Source Data for Expanded View [file EMMM-14-e14455-s002.zip › EMM-2021-14455_SourceDataForExpandedView/Fig_EV_3/EV3F/EV3F.jpg]

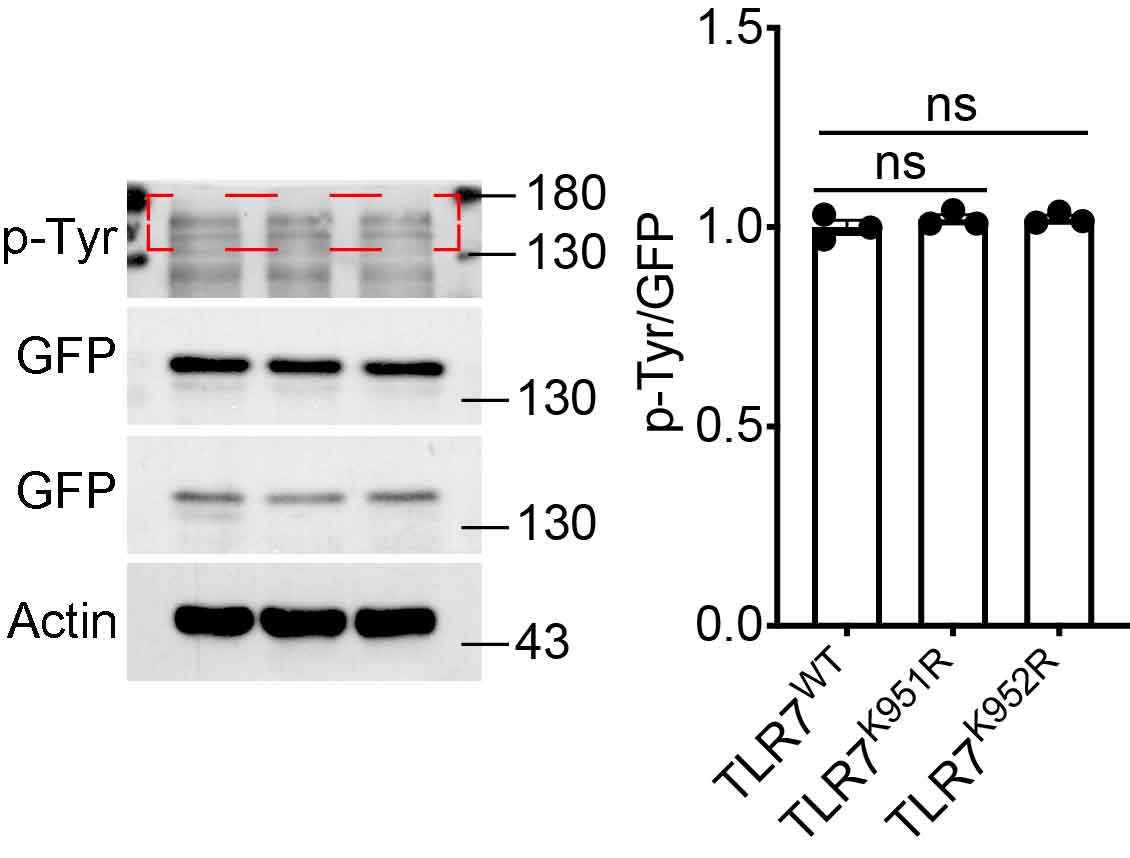

Supplement: Supplementary file 3 — Source Data for Expanded View [file EMMM-14-e14455-s002.zip › EMM-2021-14455_SourceDataForExpandedView/Fig_EV_3/EV3G/EV3G.jpg]

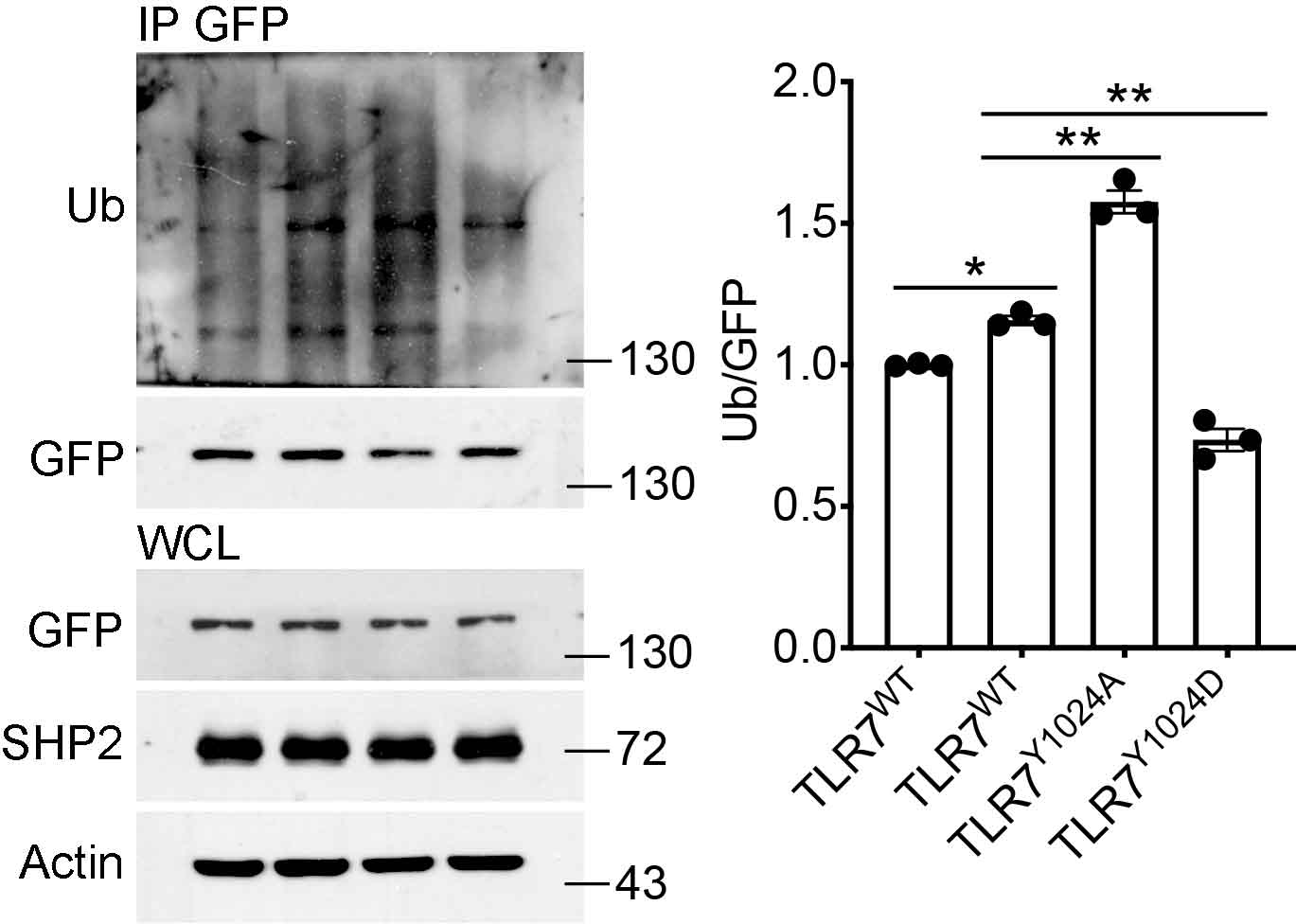

Supplement: Supplementary file 3 — Source Data for Expanded View [file EMMM-14-e14455-s002.zip › EMM-2021-14455_SourceDataForExpandedView/Fig_EV_3/EV3H/EV3H.jpg]

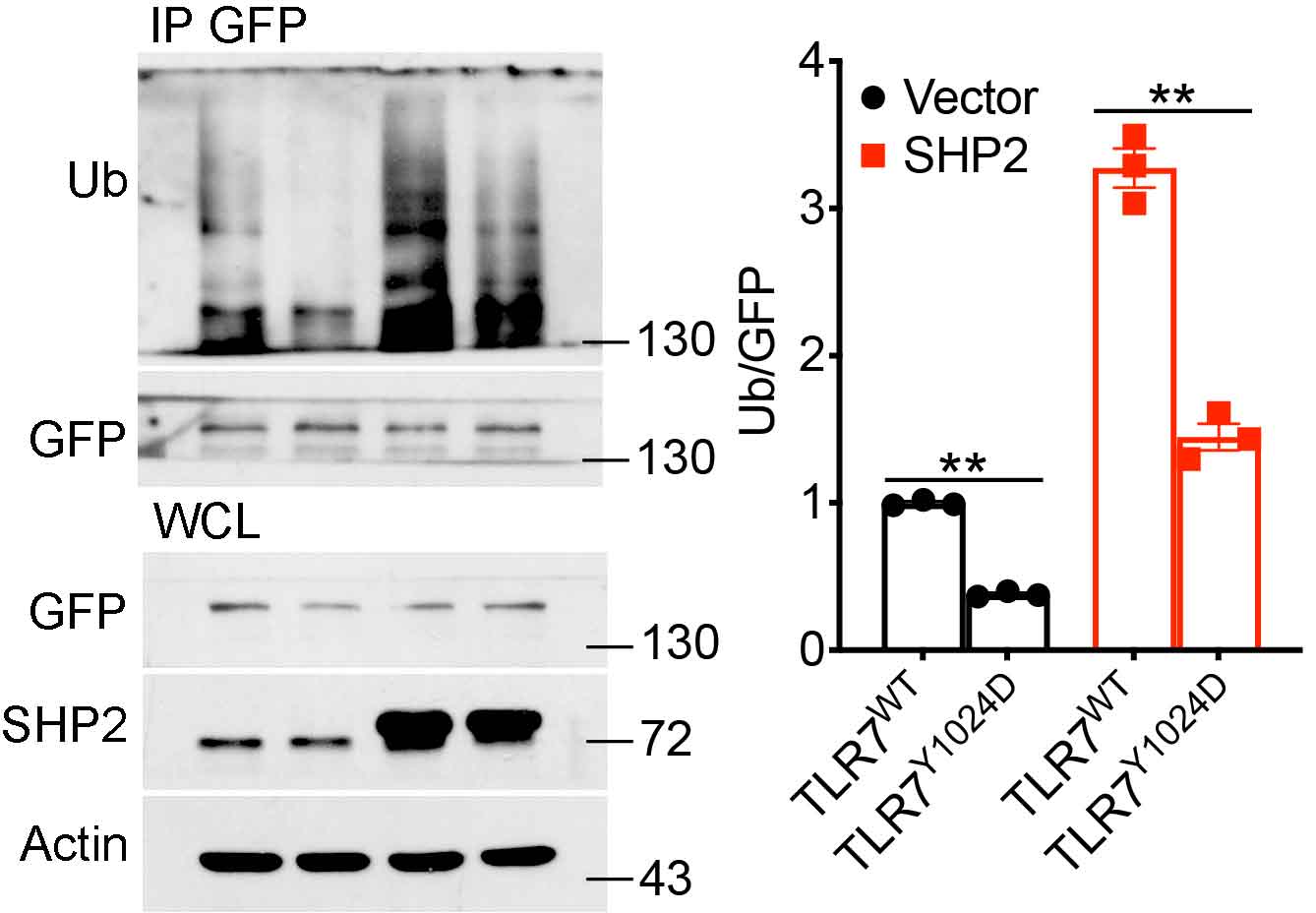

Supplement: Supplementary file 3 — Source Data for Expanded View [file EMMM-14-e14455-s002.zip › EMM-2021-14455_SourceDataForExpandedView/Fig_EV_3/EV3I/EV3I.jpg]

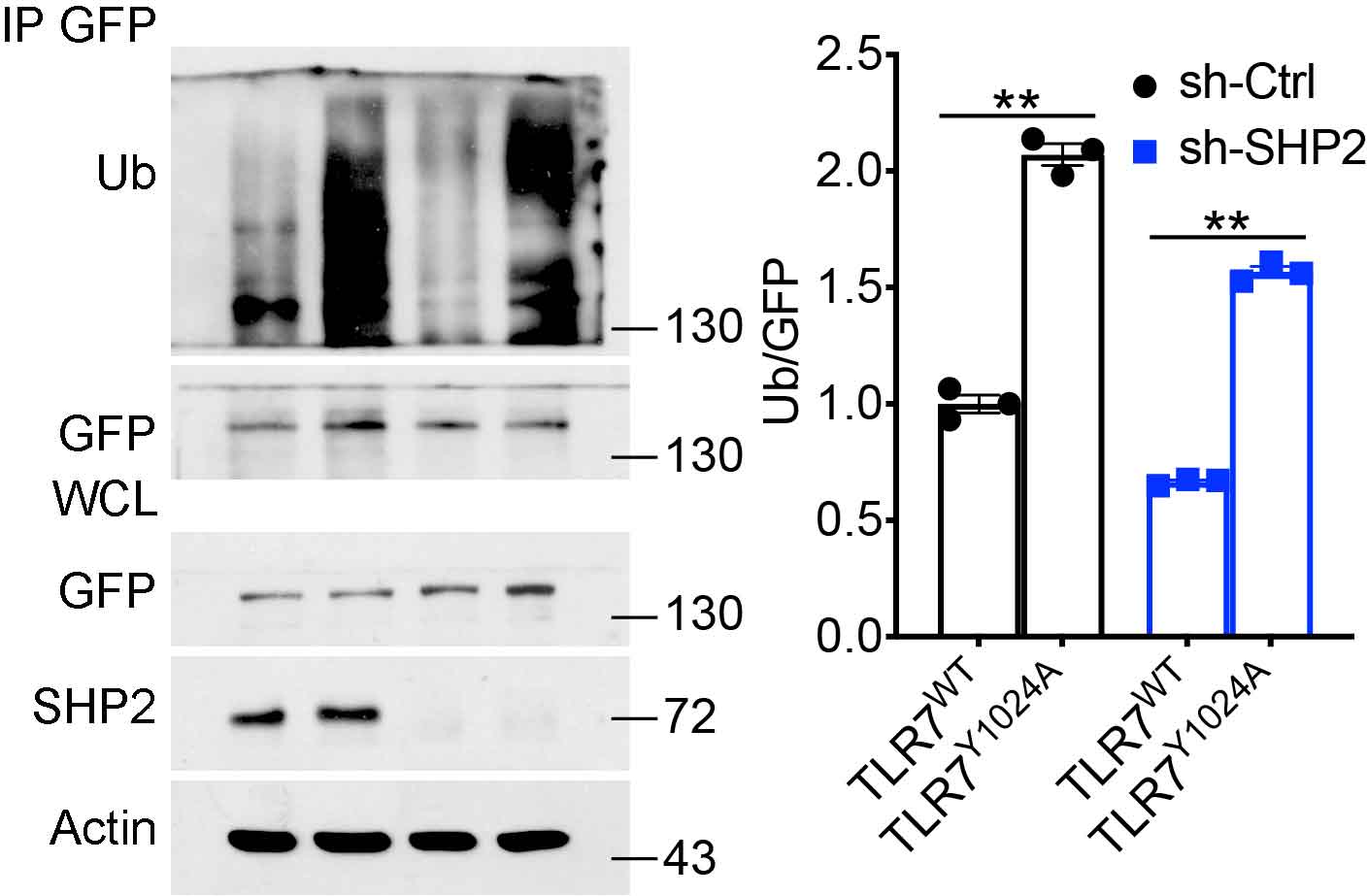

Supplement: Supplementary file 3 — Source Data for Expanded View [file EMMM-14-e14455-s002.zip › EMM-2021-14455_SourceDataForExpandedView/Fig_EV_3/EV3J/EV3J.jpg]

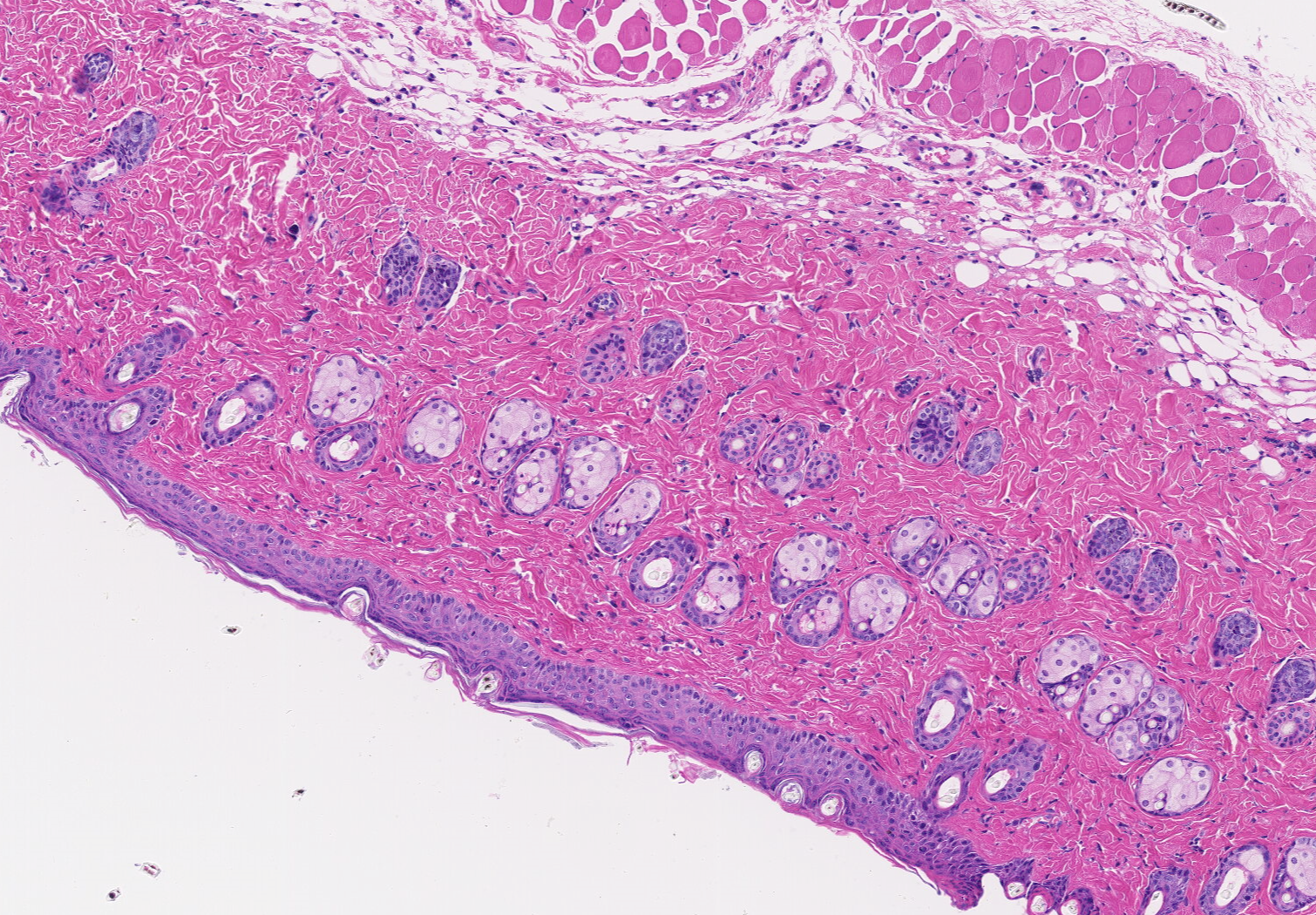

Supplement: Supplementary file 3 — Source Data for Expanded View [file EMMM-14-e14455-s002.zip › EMM-2021-14455_SourceDataForExpandedView/Fig_EV_4/EV4A/H&E/KI.tiff]

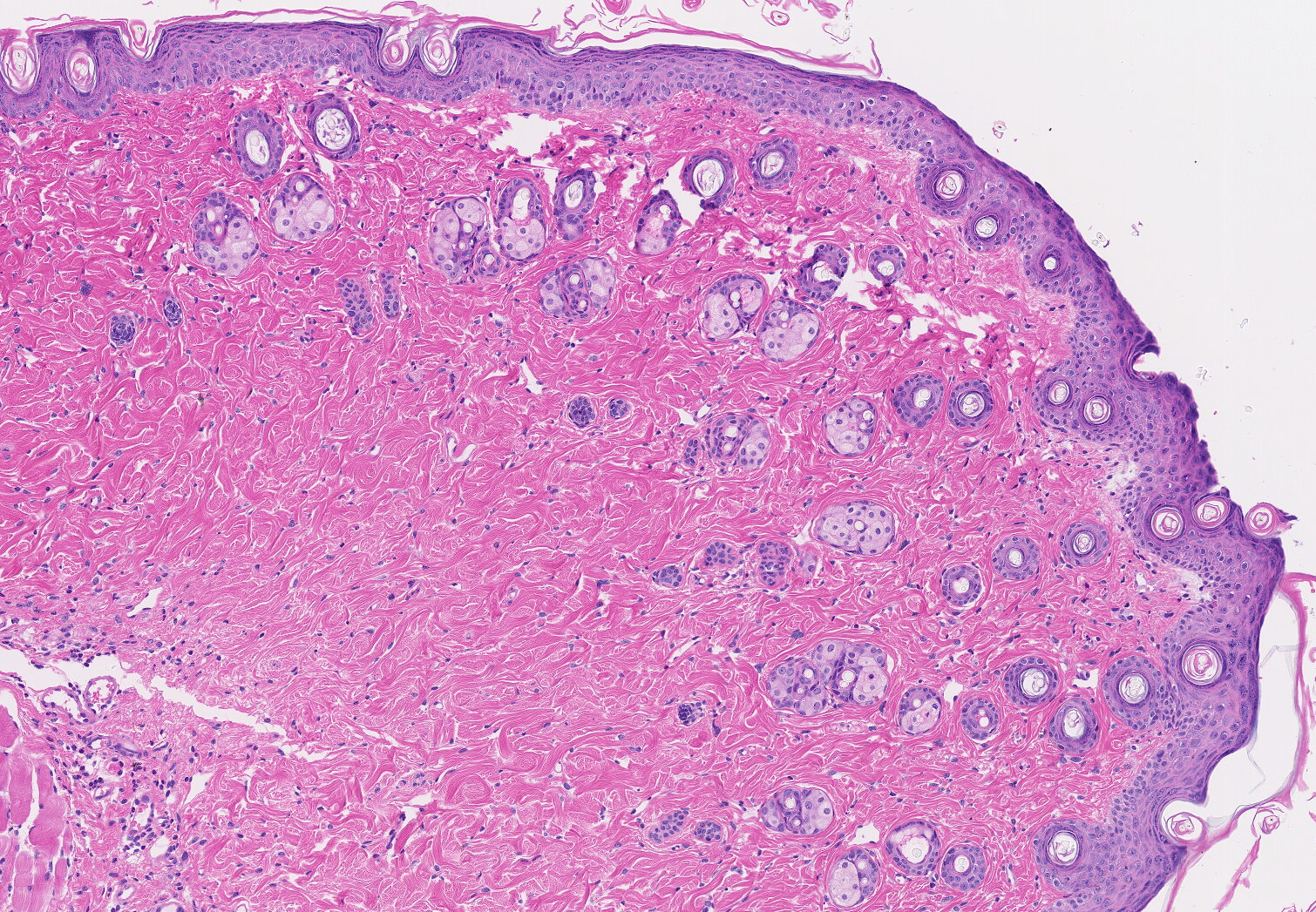

Supplement: Supplementary file 3 — Source Data for Expanded View [file EMMM-14-e14455-s002.zip › EMM-2021-14455_SourceDataForExpandedView/Fig_EV_4/EV4A/H&E/WT.tiff]

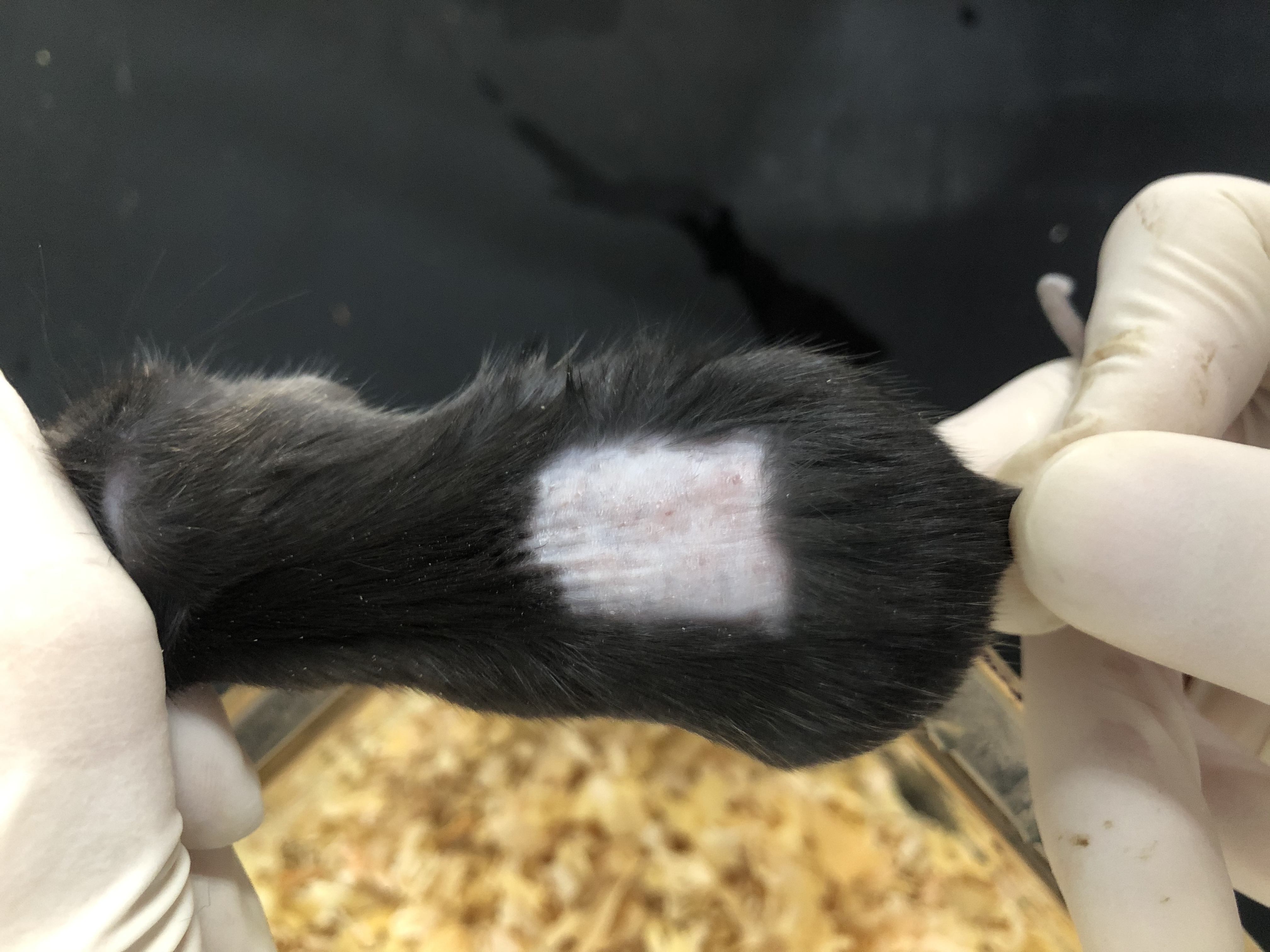

Supplement: Supplementary file 3 — Source Data for Expanded View [file EMMM-14-e14455-s002.zip › EMM-2021-14455_SourceDataForExpandedView/Fig_EV_4/EV4A/photo/Tlr7-ki.jpg]

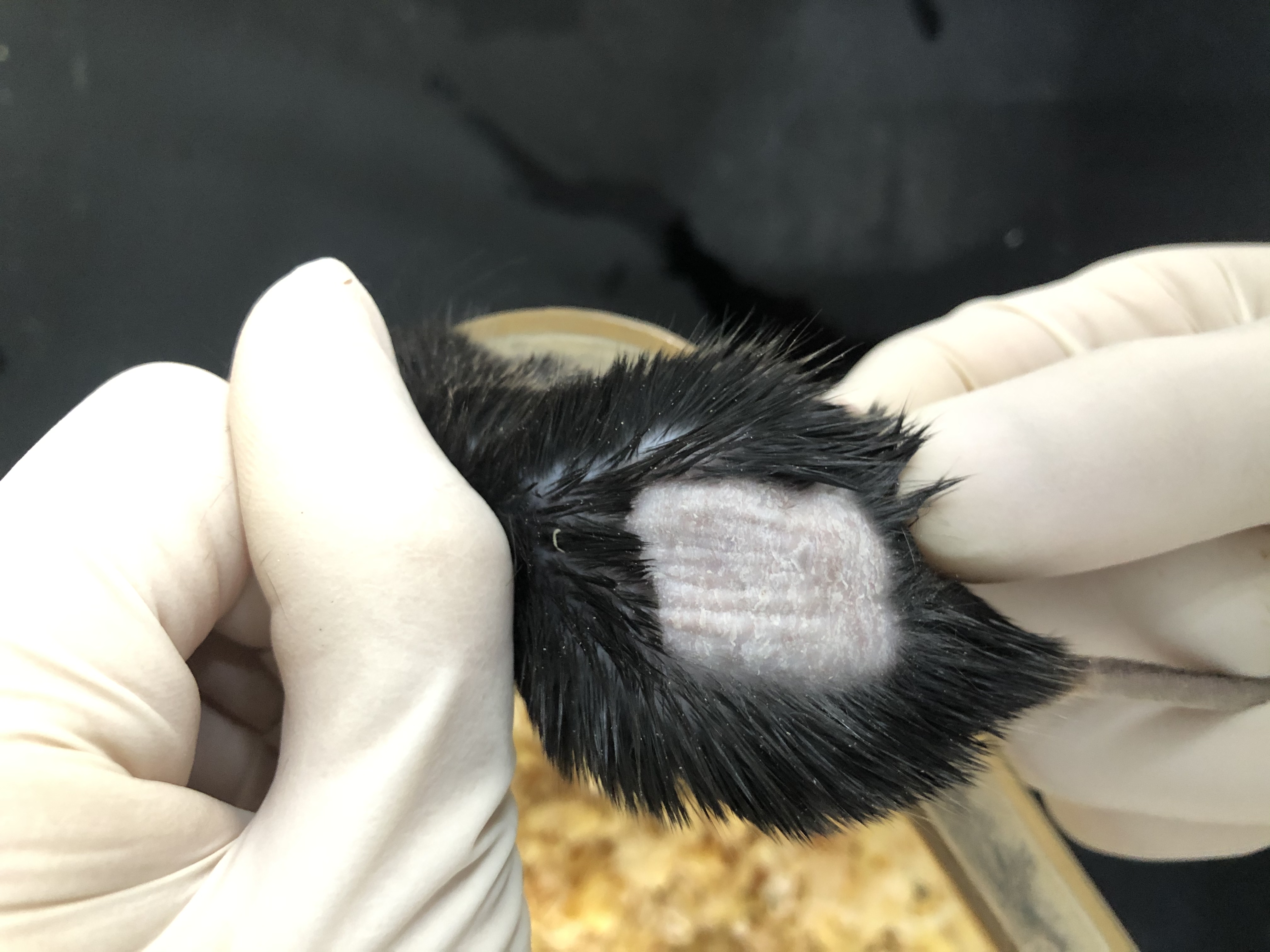

Supplement: Supplementary file 3 — Source Data for Expanded View [file EMMM-14-e14455-s002.zip › EMM-2021-14455_SourceDataForExpandedView/Fig_EV_4/EV4A/photo/Tlr7-wt.jpg]

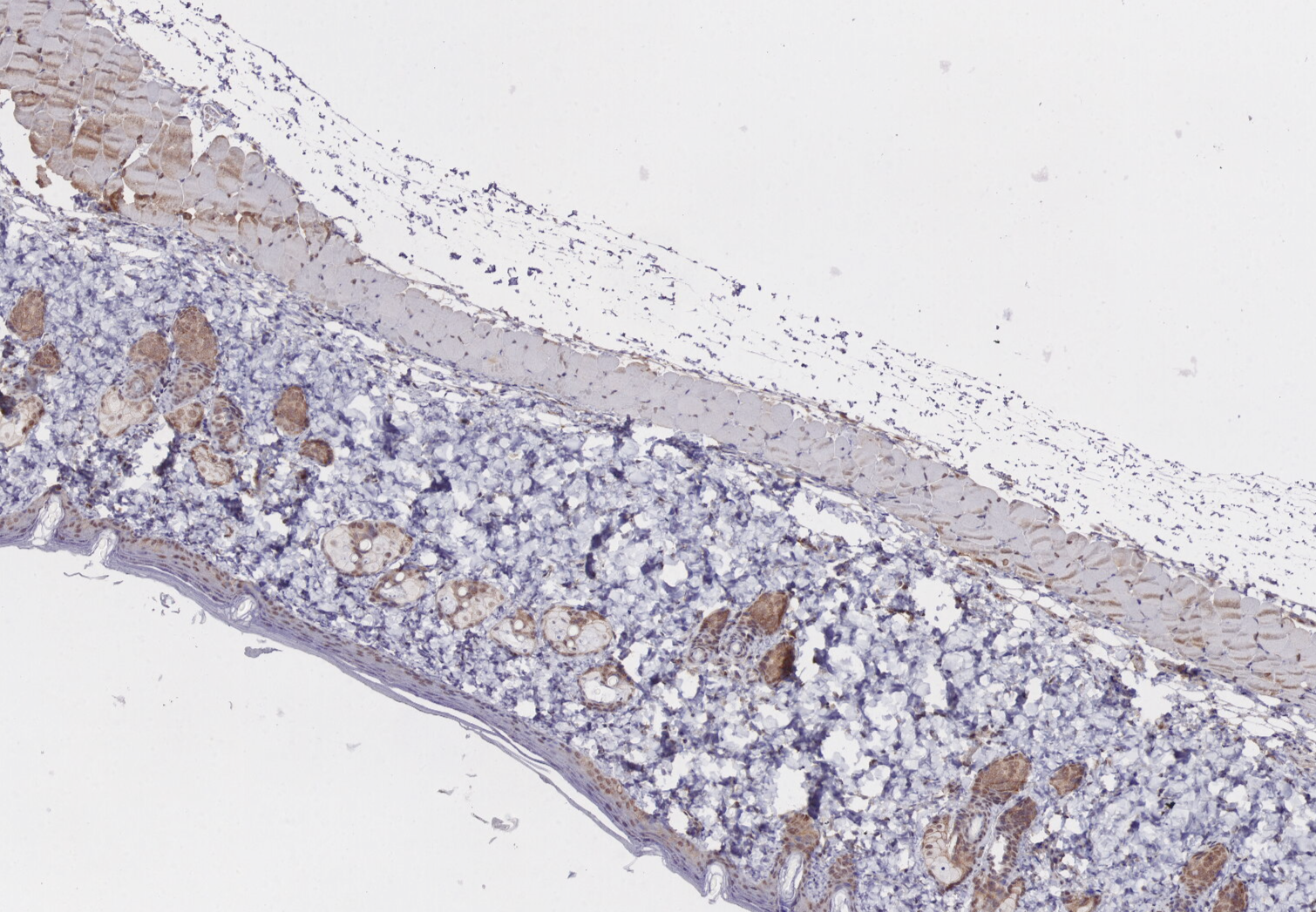

Supplement: Supplementary file 3 — Source Data for Expanded View [file EMMM-14-e14455-s002.zip › EMM-2021-14455_SourceDataForExpandedView/Fig_EV_4/EV4E/KI.tiff]

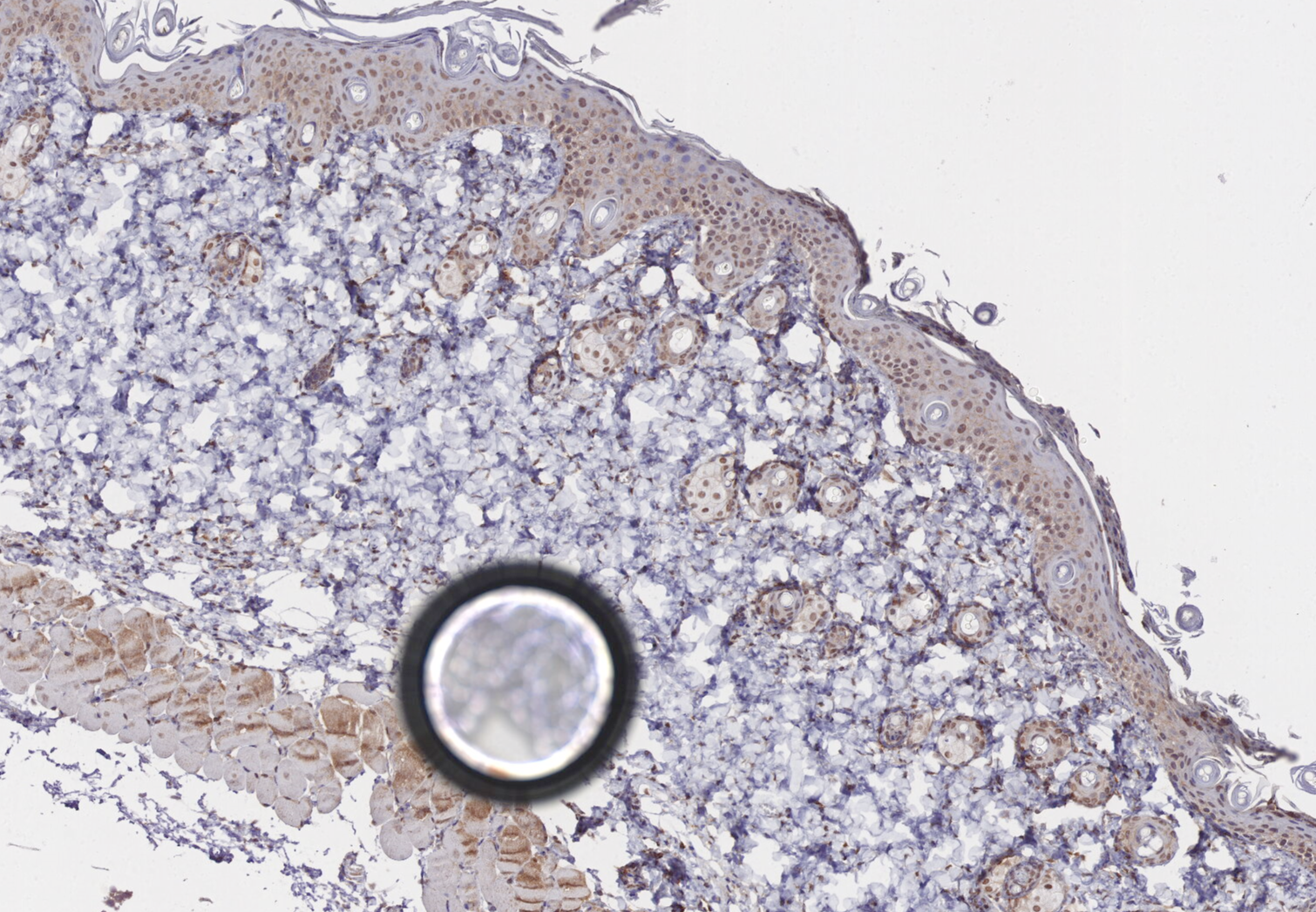

Supplement: Supplementary file 3 — Source Data for Expanded View [file EMMM-14-e14455-s002.zip › EMM-2021-14455_SourceDataForExpandedView/Fig_EV_4/EV4E/WT.tiff]

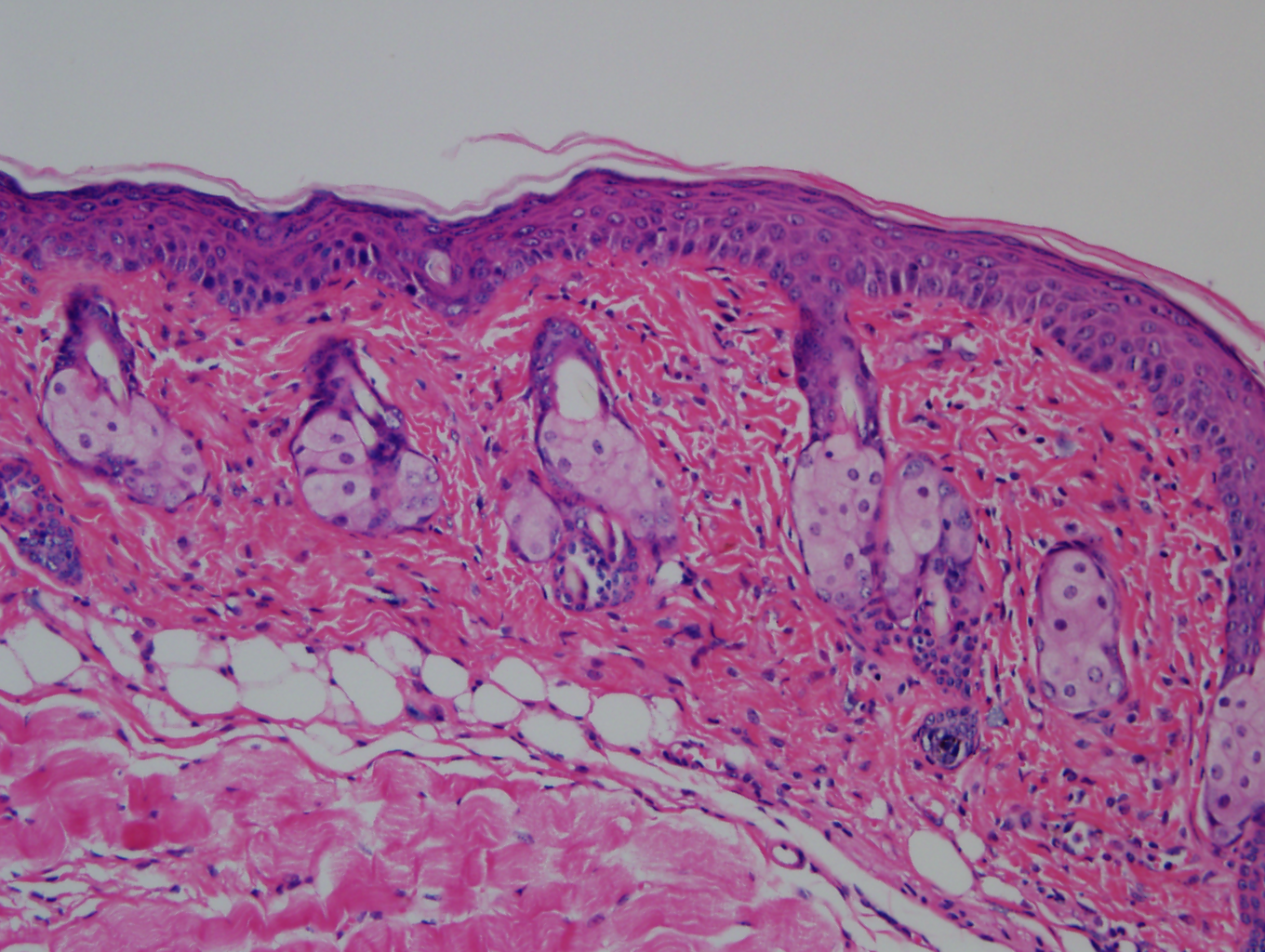

Supplement: Supplementary file 3 — Source Data for Expanded View [file EMMM-14-e14455-s002.zip › EMM-2021-14455_SourceDataForExpandedView/Fig_EV_5/EV5A/H&E/PBS.tif]

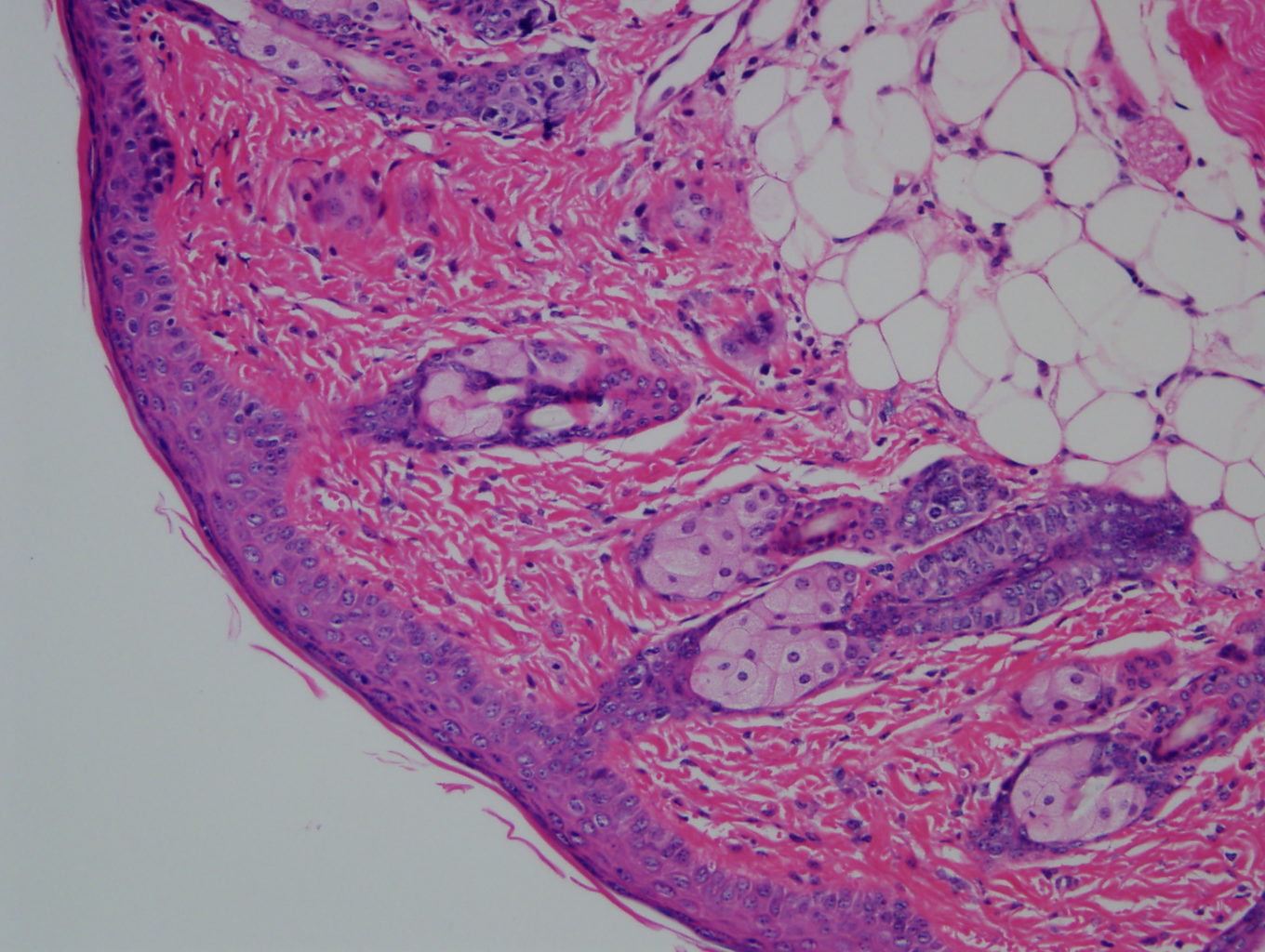

Supplement: Supplementary file 3 — Source Data for Expanded View [file EMMM-14-e14455-s002.zip › EMM-2021-14455_SourceDataForExpandedView/Fig_EV_5/EV5A/H&E/SHP099.tif]

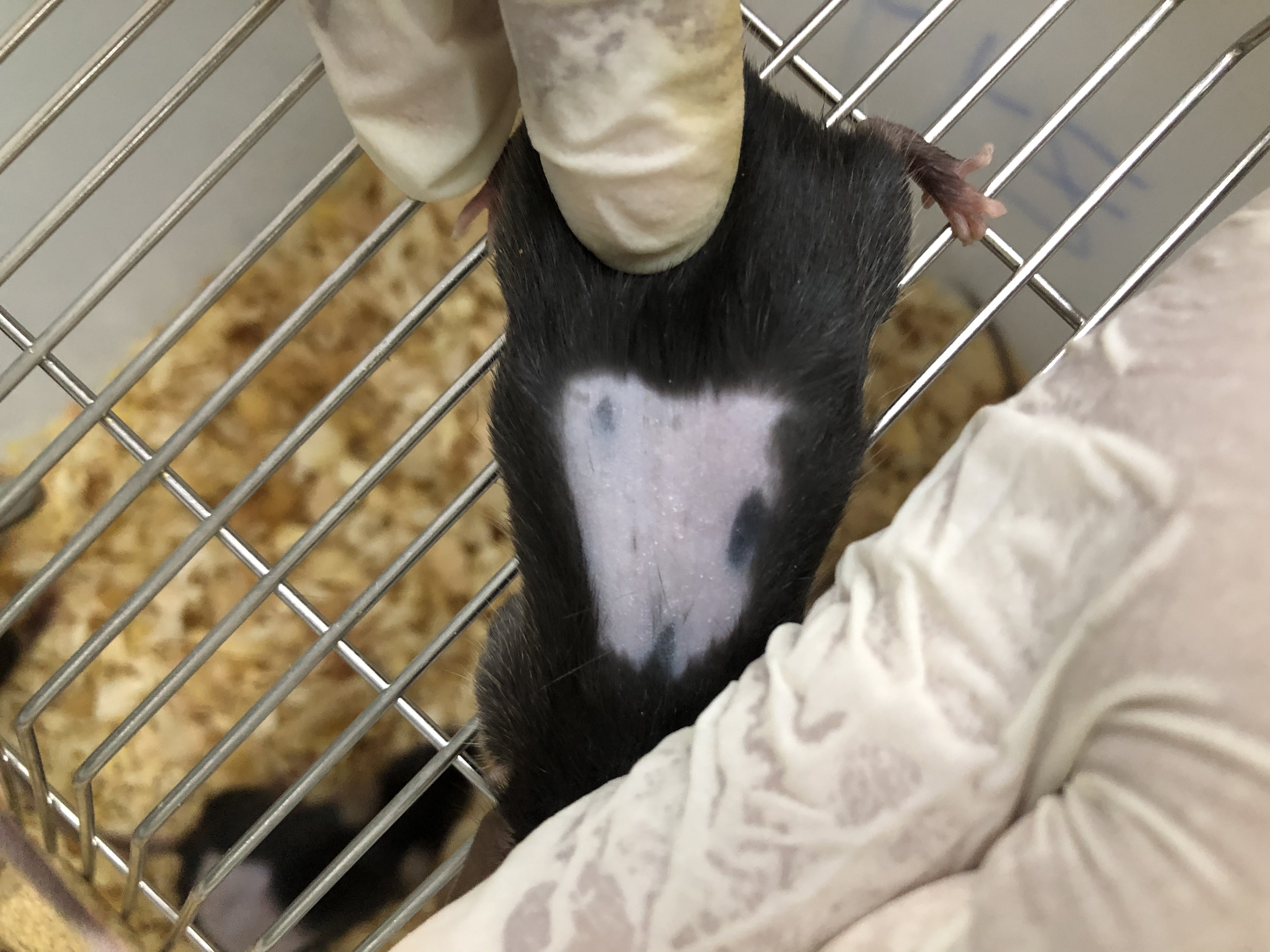

Supplement: Supplementary file 3 — Source Data for Expanded View [file EMMM-14-e14455-s002.zip › EMM-2021-14455_SourceDataForExpandedView/Fig_EV_5/EV5A/photo/PBS.jpg]

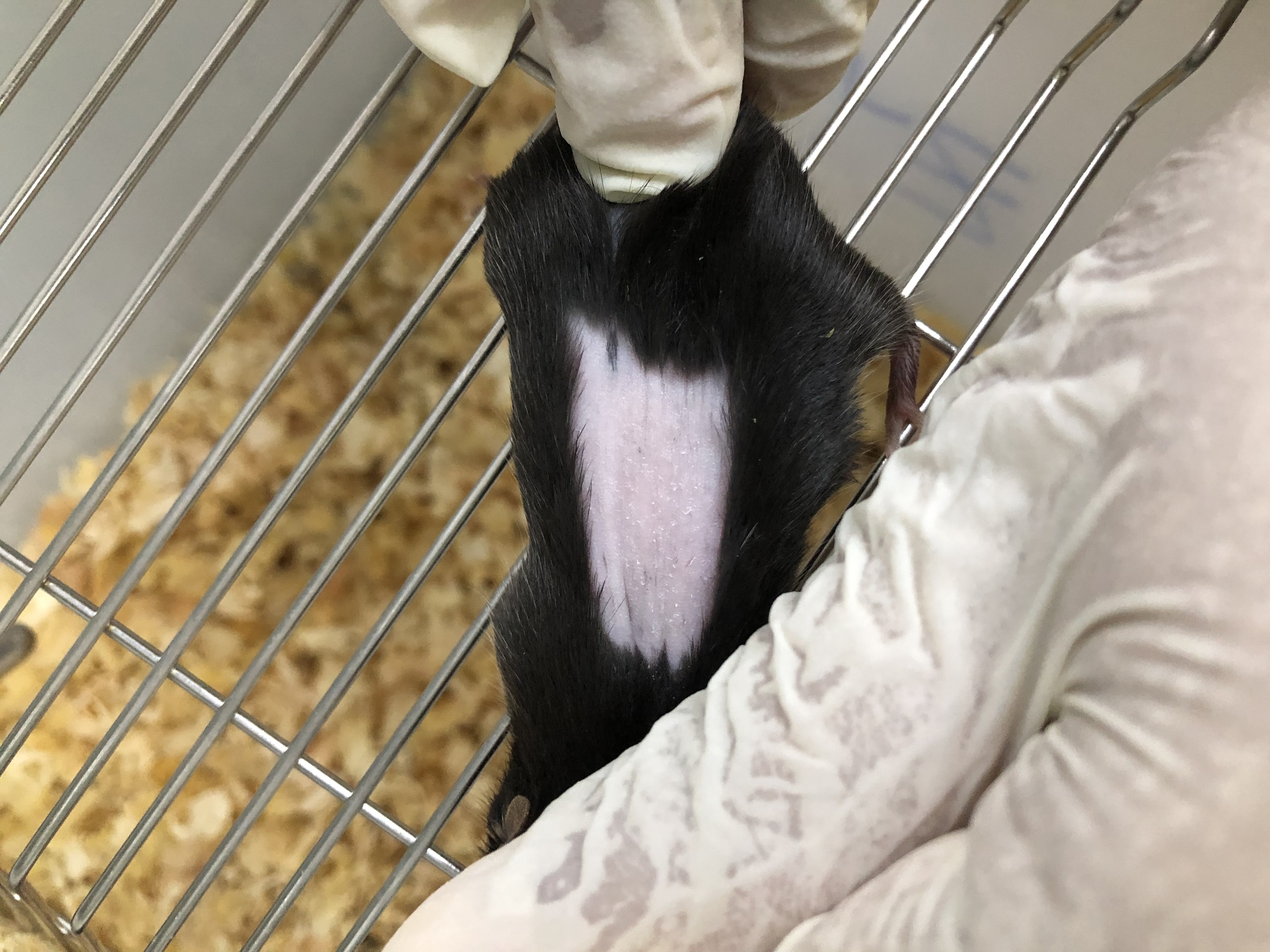

Supplement: Supplementary file 3 — Source Data for Expanded View [file EMMM-14-e14455-s002.zip › EMM-2021-14455_SourceDataForExpandedView/Fig_EV_5/EV5A/photo/SHP099.jpg]

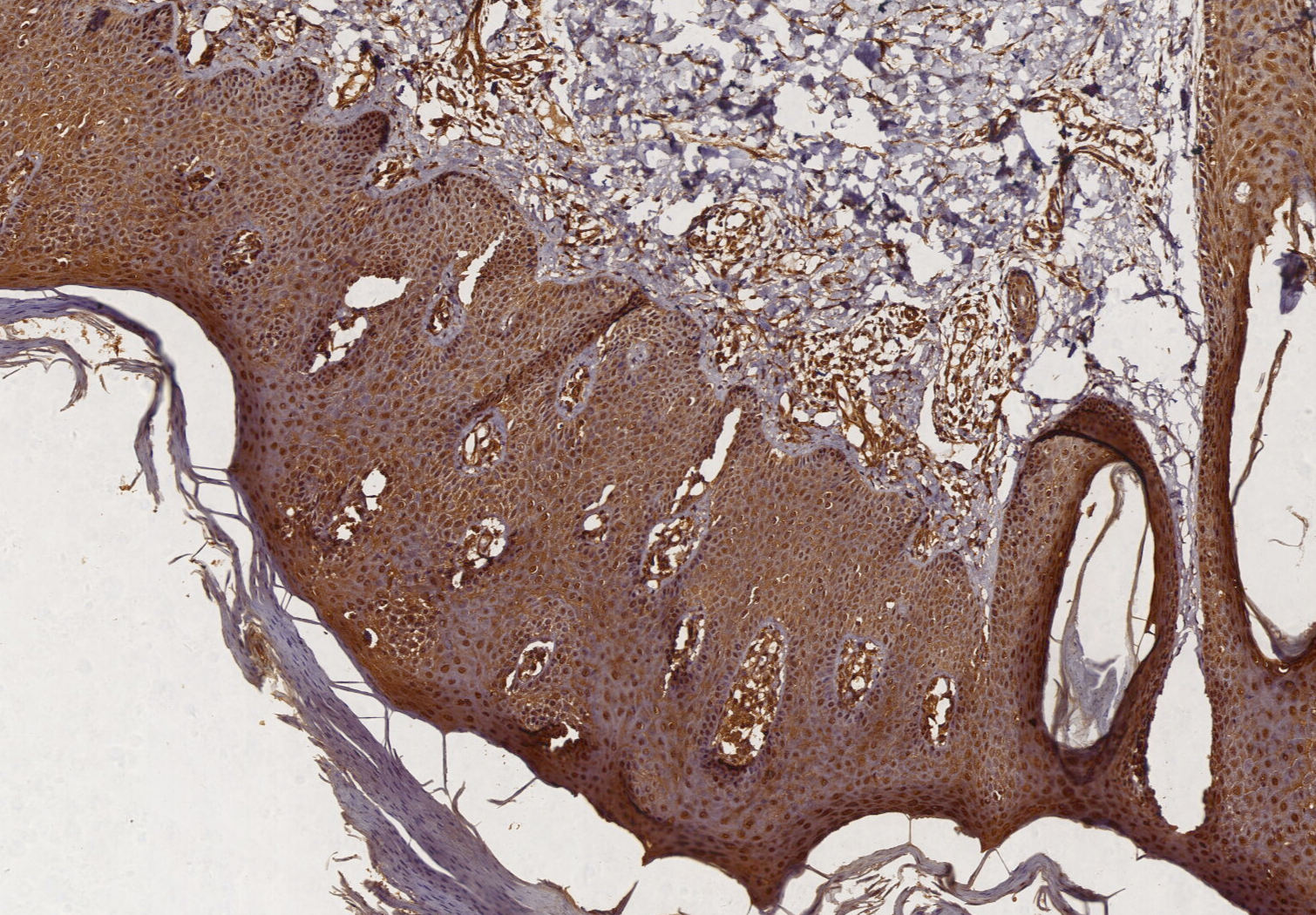

Supplement: Supplementary file 4 — Source Data for Figure 1 [file EMMM-14-e14455-s011.zip › Figure_1/1_C/61887.tiff]

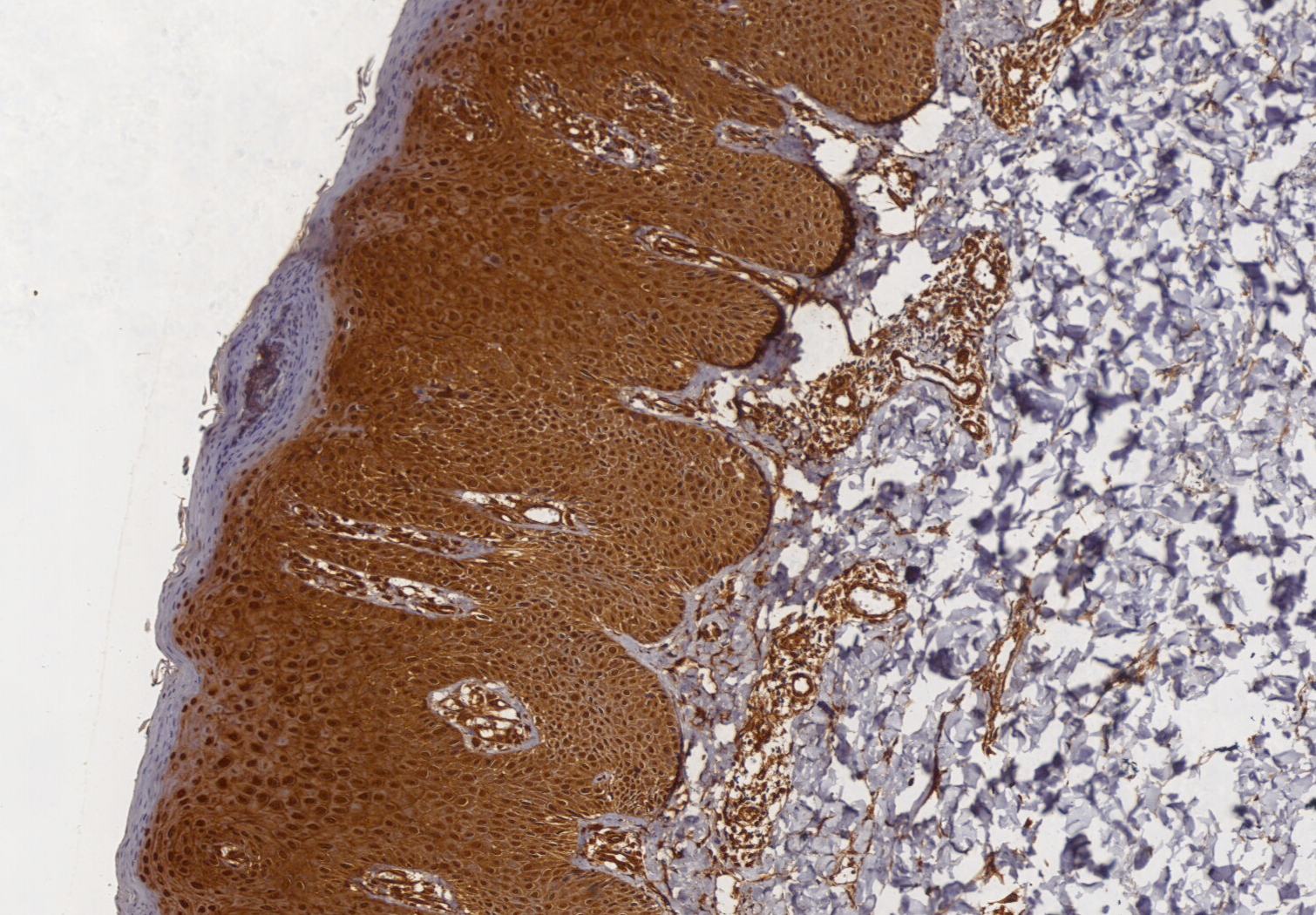

Supplement: Supplementary file 4 — Source Data for Figure 1 [file EMMM-14-e14455-s011.zip › Figure_1/1_C/62034.tiff]

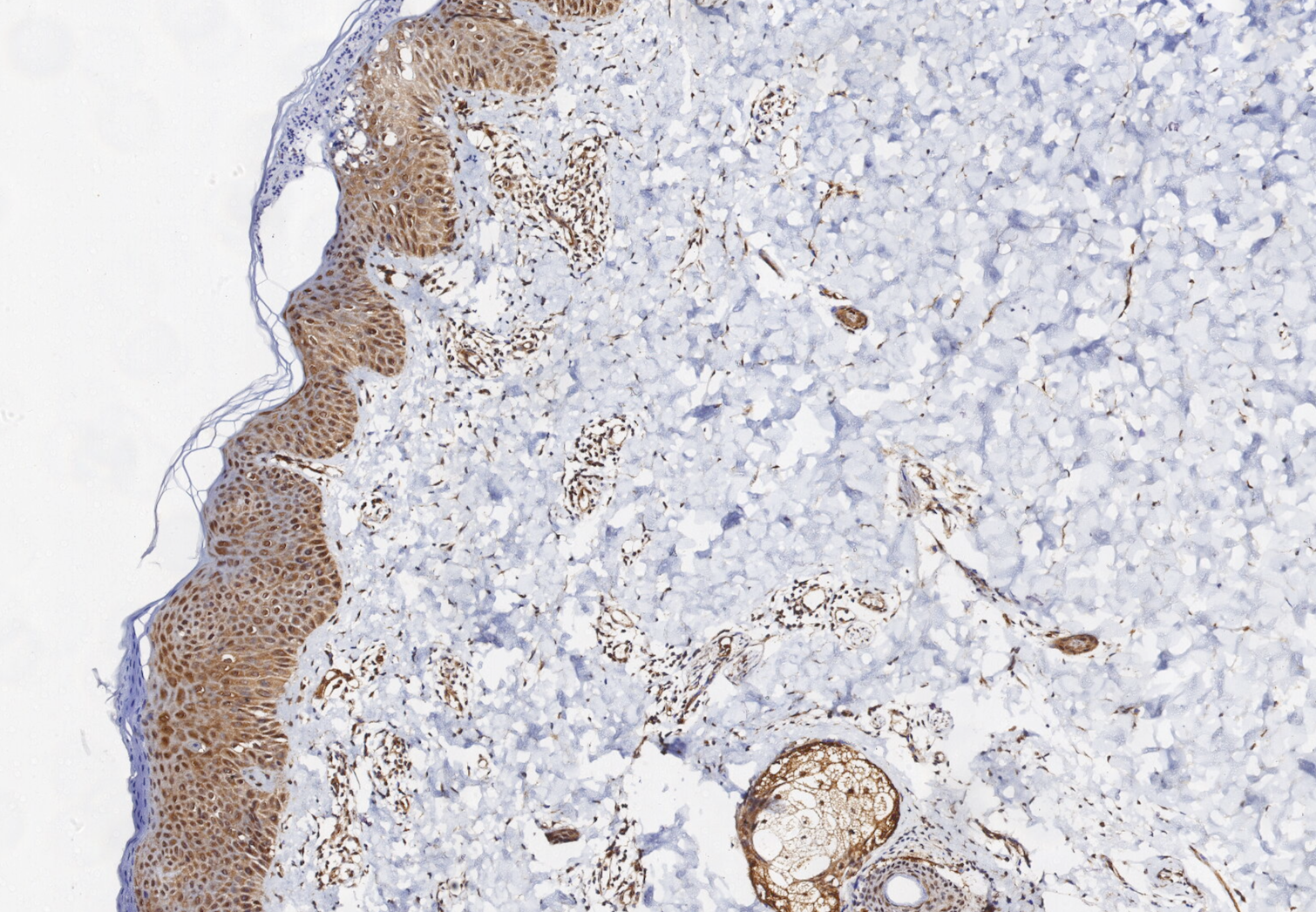

Supplement: Supplementary file 4 — Source Data for Figure 1 [file EMMM-14-e14455-s011.zip › Figure_1/1_C/62278.tiff]

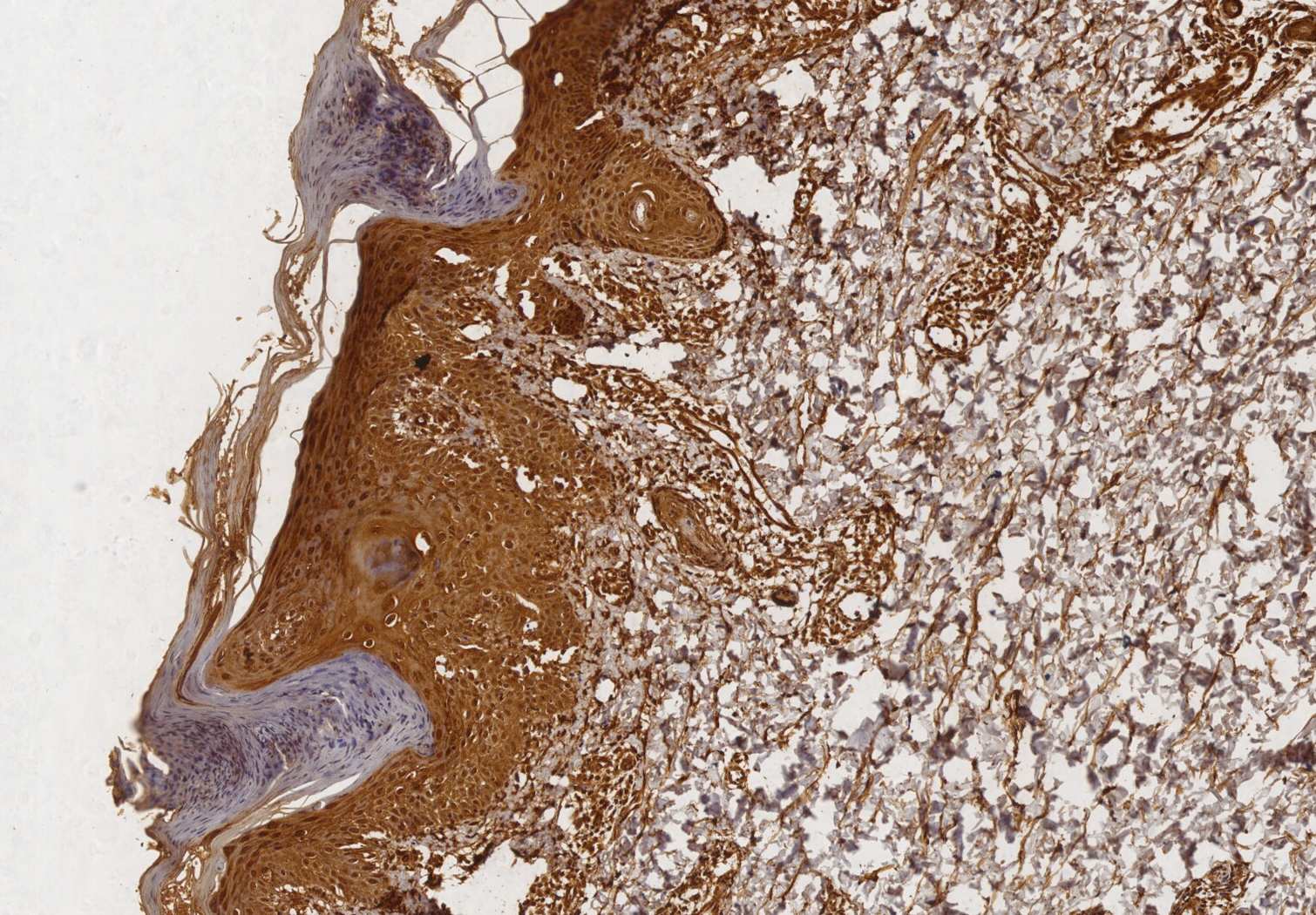

Supplement: Supplementary file 4 — Source Data for Figure 1 [file EMMM-14-e14455-s011.zip › Figure_1/1_C/62374.tiff]

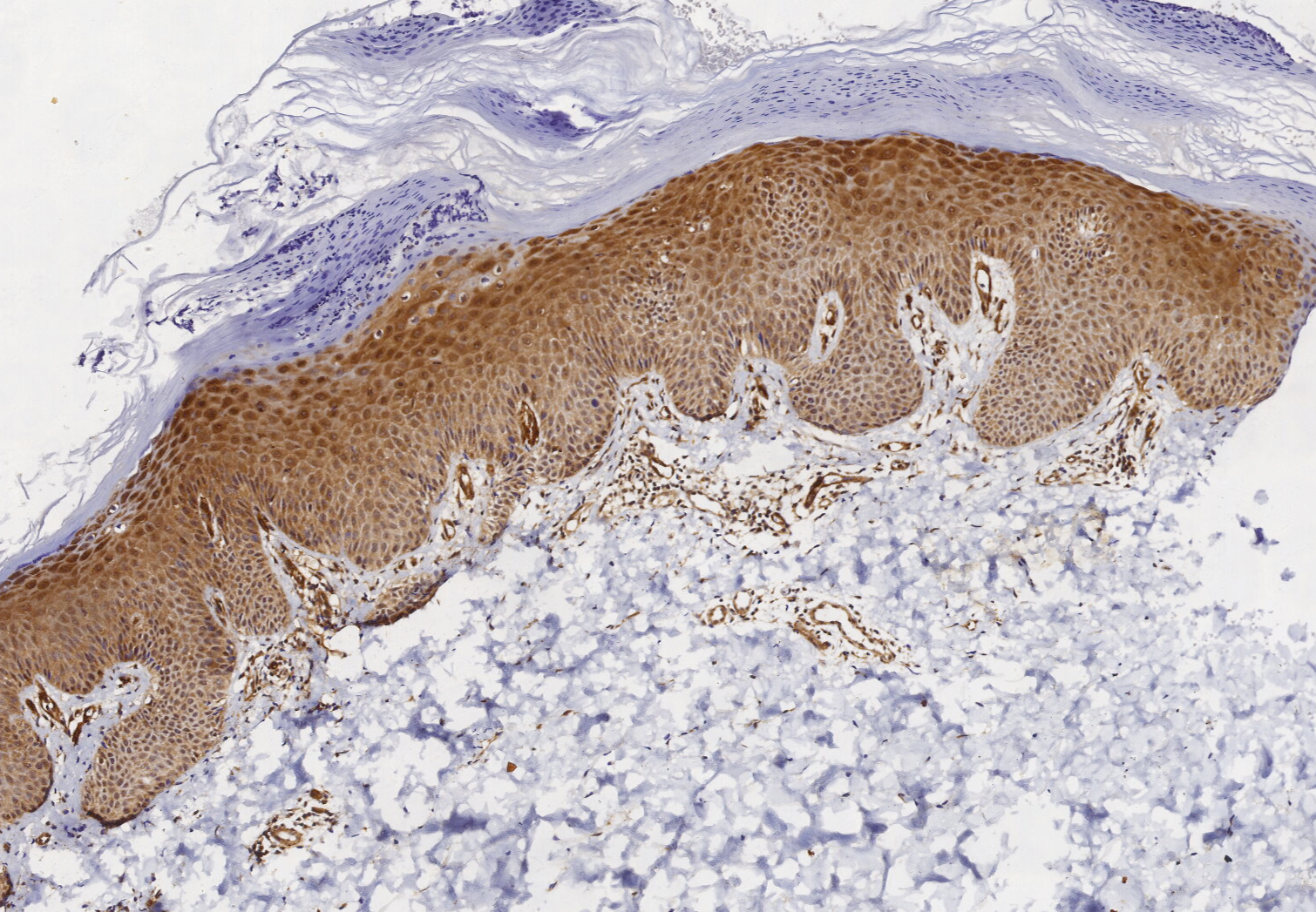

Supplement: Supplementary file 4 — Source Data for Figure 1 [file EMMM-14-e14455-s011.zip › Figure_1/1_C/62430.tiff]

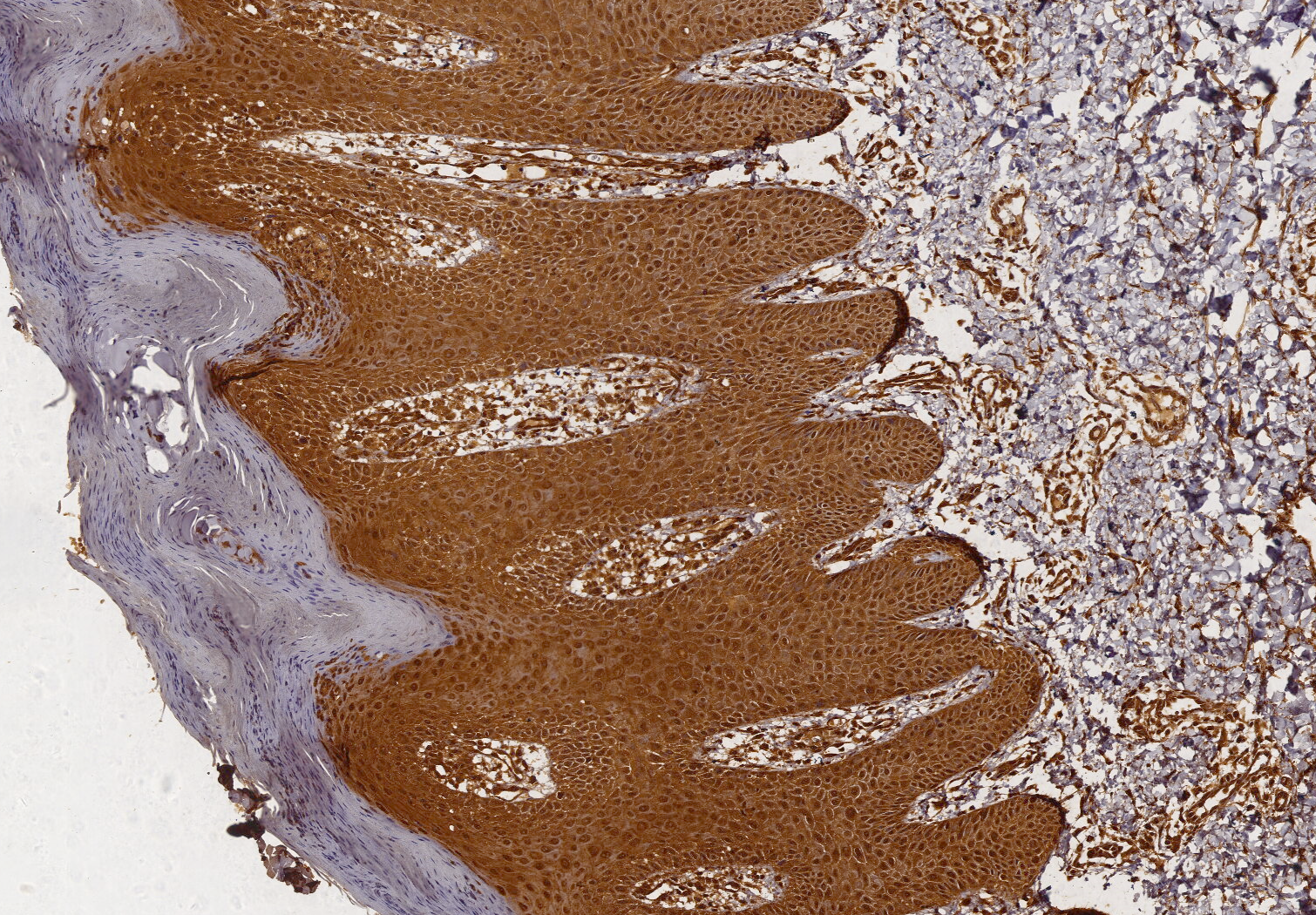

Supplement: Supplementary file 4 — Source Data for Figure 1 [file EMMM-14-e14455-s011.zip › Figure_1/1_C/62492.tiff]

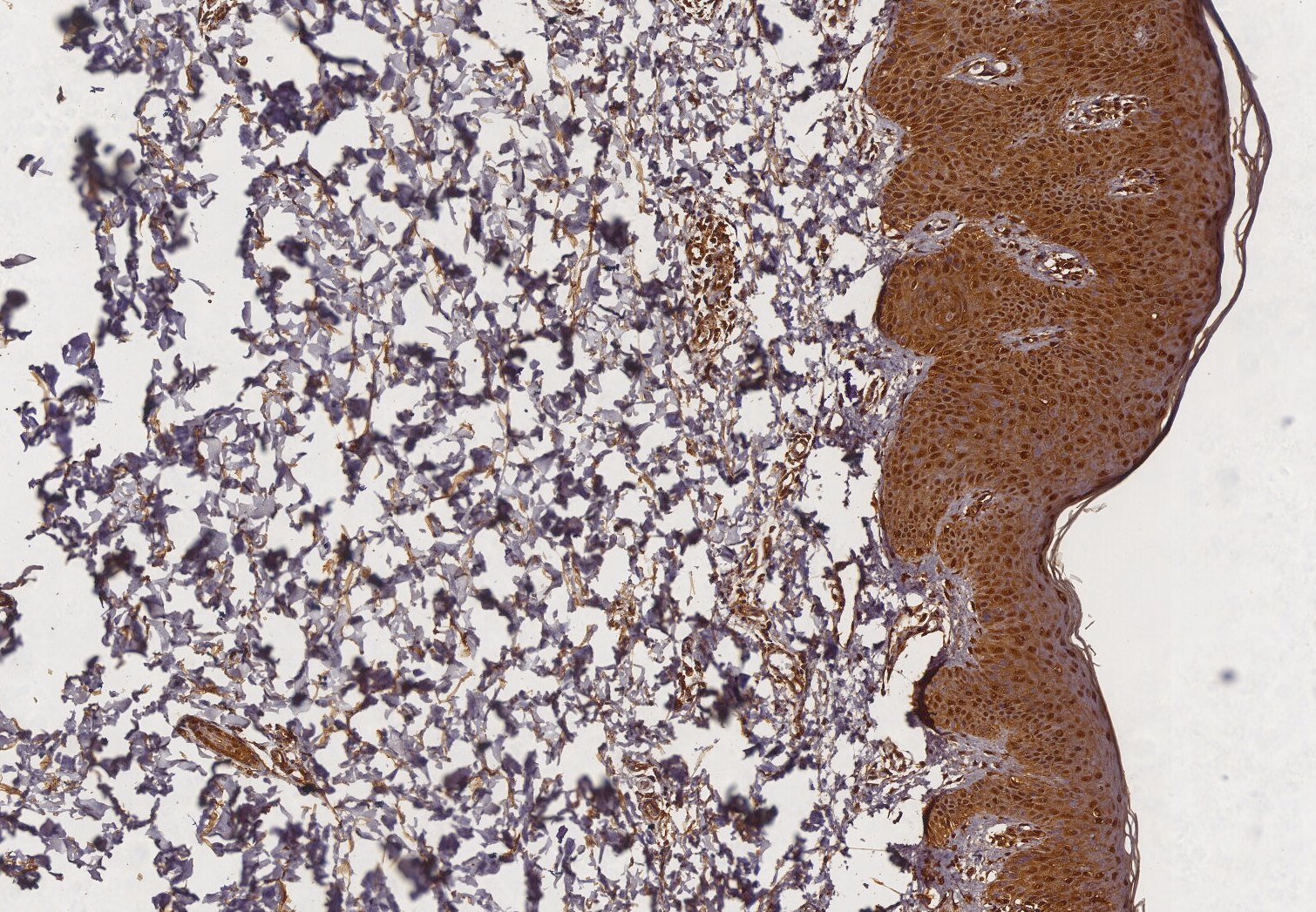

Supplement: Supplementary file 4 — Source Data for Figure 1 [file EMMM-14-e14455-s011.zip › Figure_1/1_C/62559.tiff]

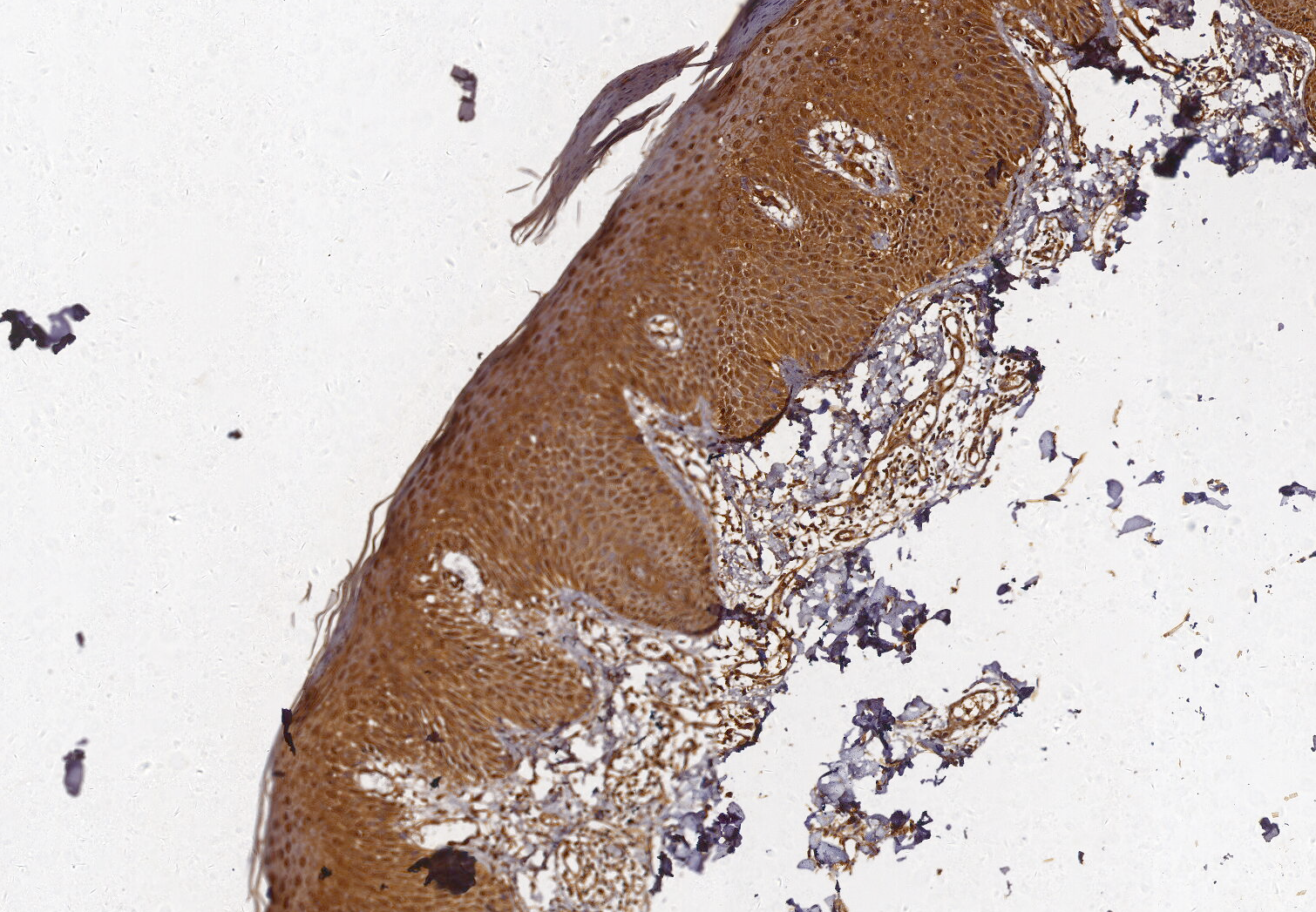

Supplement: Supplementary file 4 — Source Data for Figure 1 [file EMMM-14-e14455-s011.zip › Figure_1/1_C/62634.tiff]

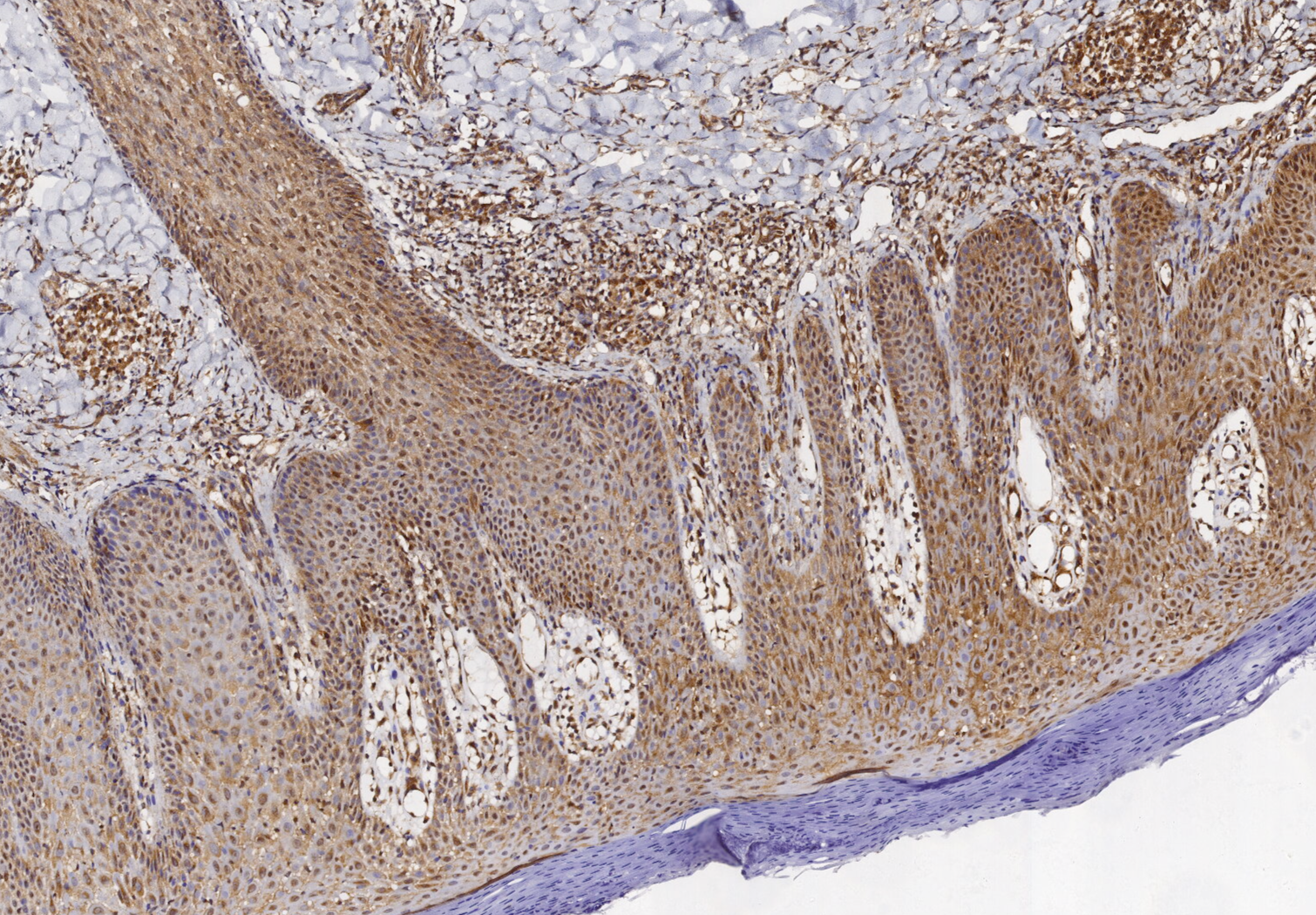

Supplement: Supplementary file 4 — Source Data for Figure 1 [file EMMM-14-e14455-s011.zip › Figure_1/1_C/62880.tiff]

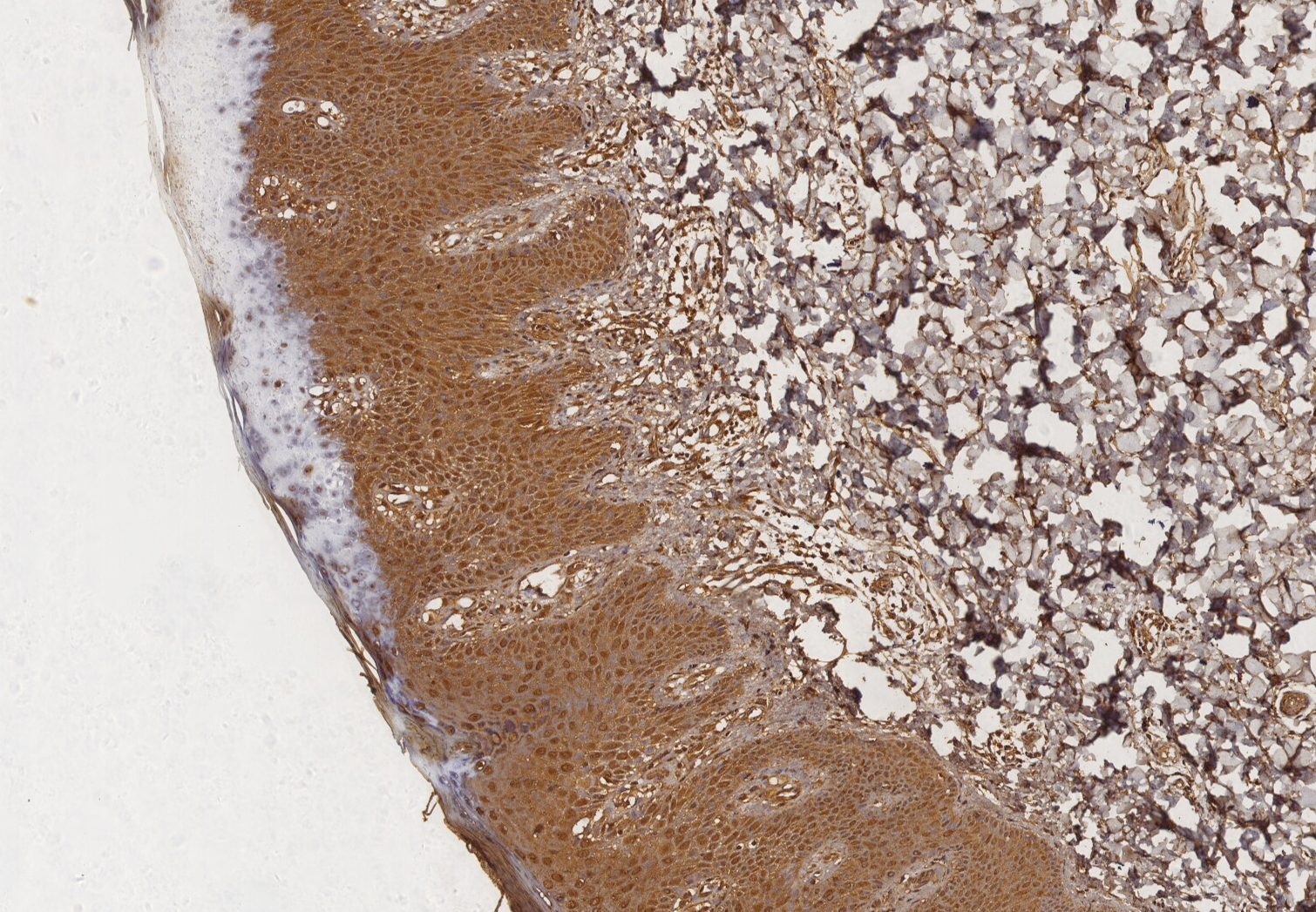

Supplement: Supplementary file 4 — Source Data for Figure 1 [file EMMM-14-e14455-s011.zip › Figure_1/1_C/64357.tiff]

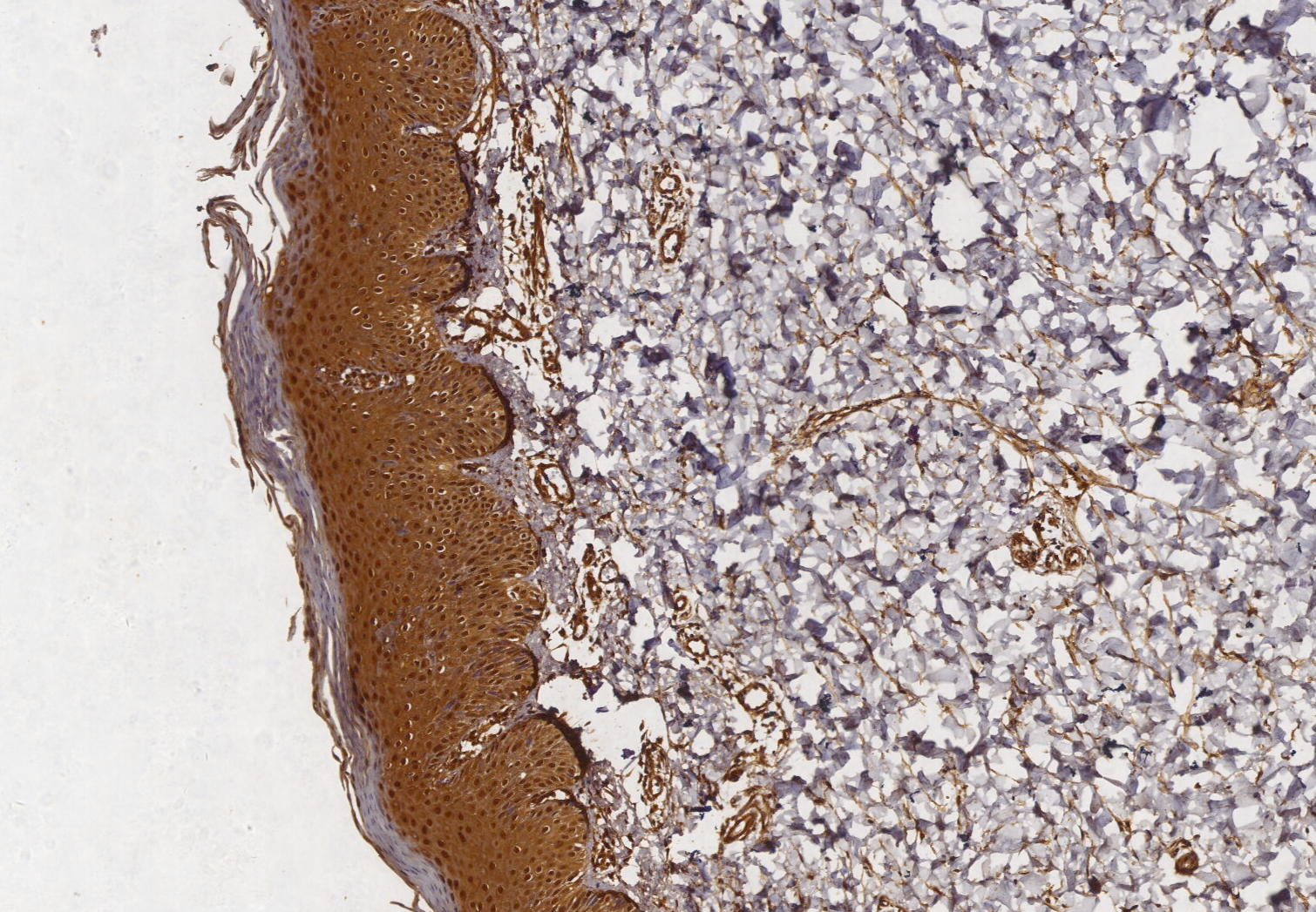

Supplement: Supplementary file 4 — Source Data for Figure 1 [file EMMM-14-e14455-s011.zip › Figure_1/1_C/64384.tiff]

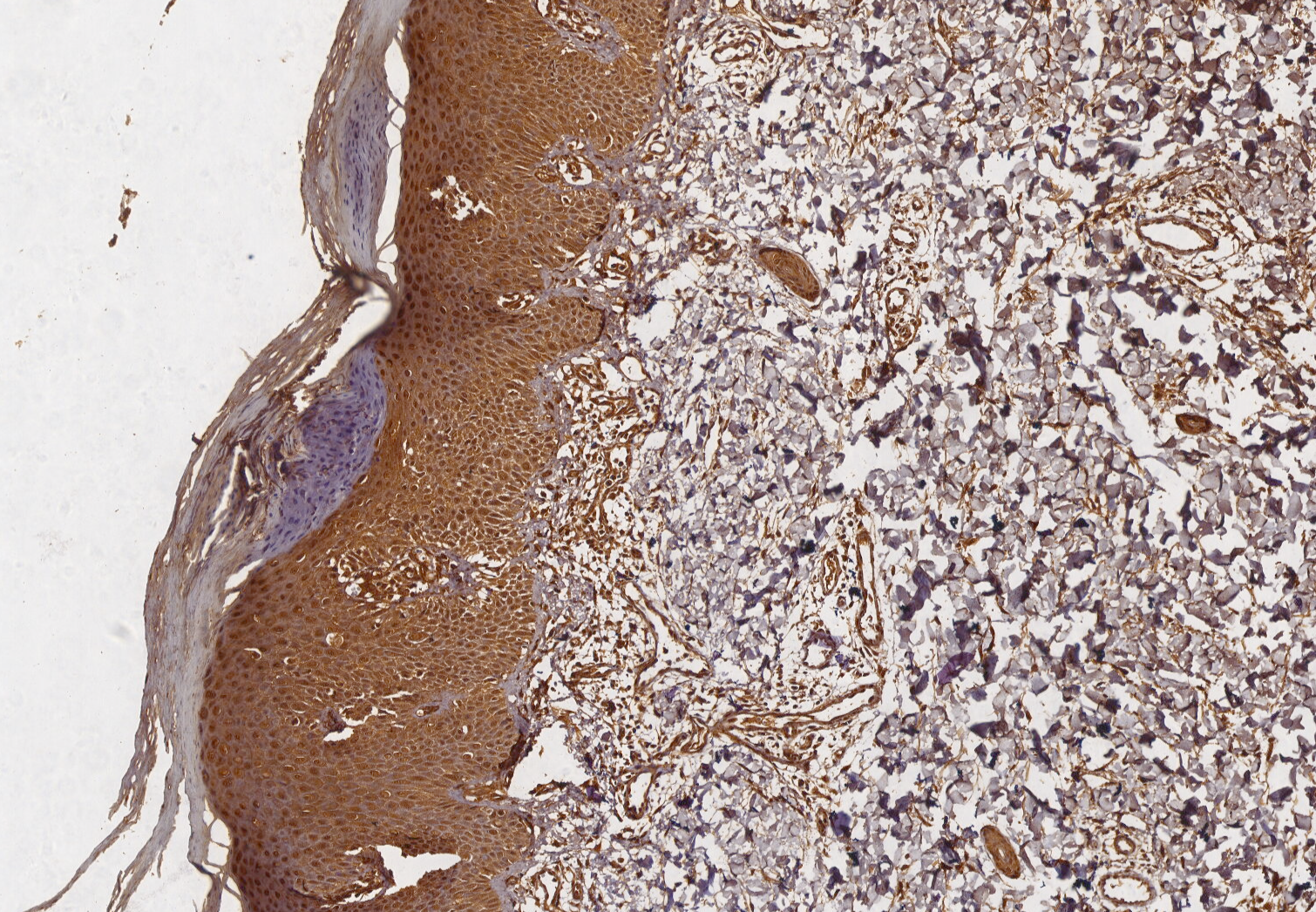

Supplement: Supplementary file 4 — Source Data for Figure 1 [file EMMM-14-e14455-s011.zip › Figure_1/1_C/64459.tiff]

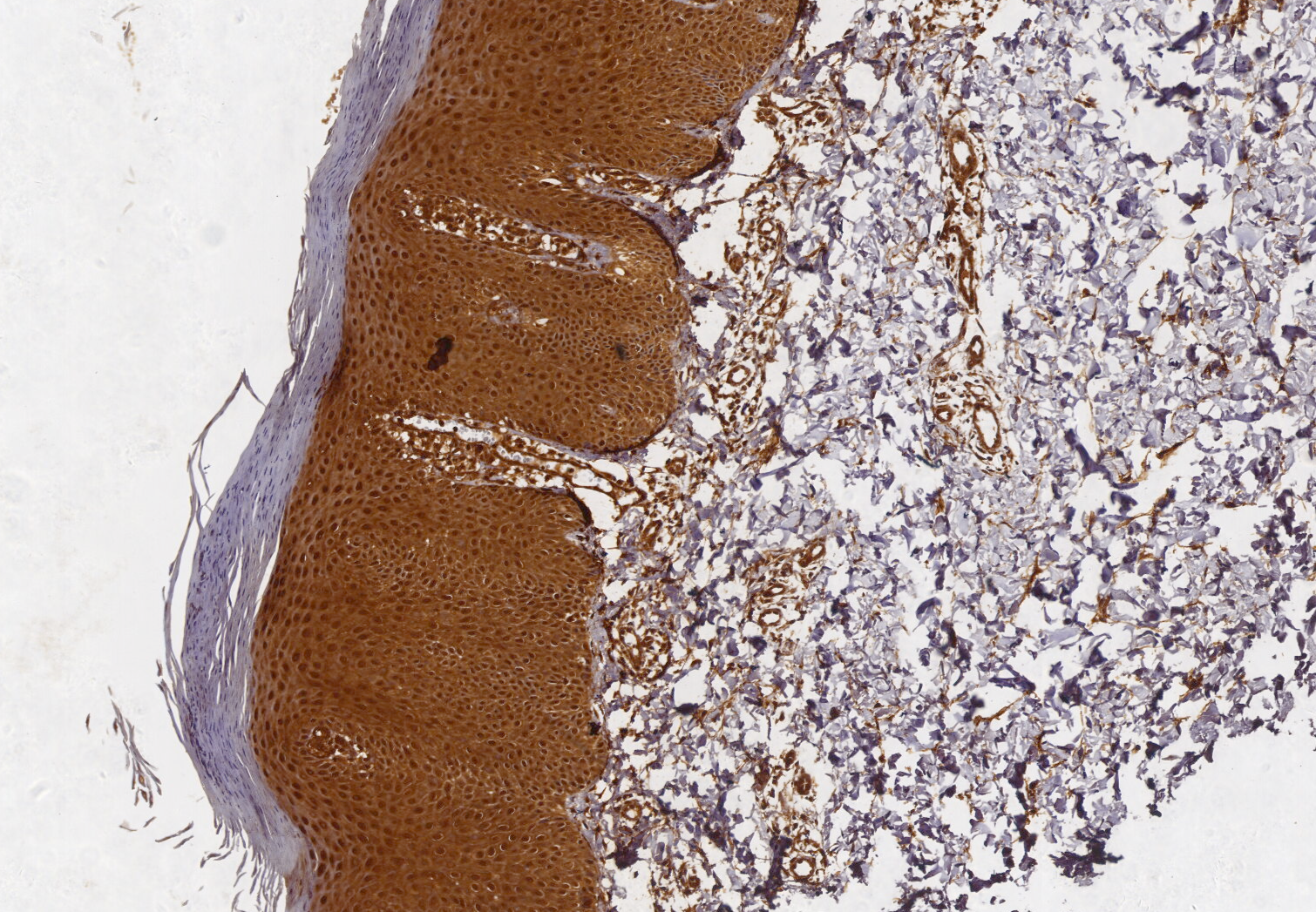

Supplement: Supplementary file 4 — Source Data for Figure 1 [file EMMM-14-e14455-s011.zip › Figure_1/1_C/64464.tiff]

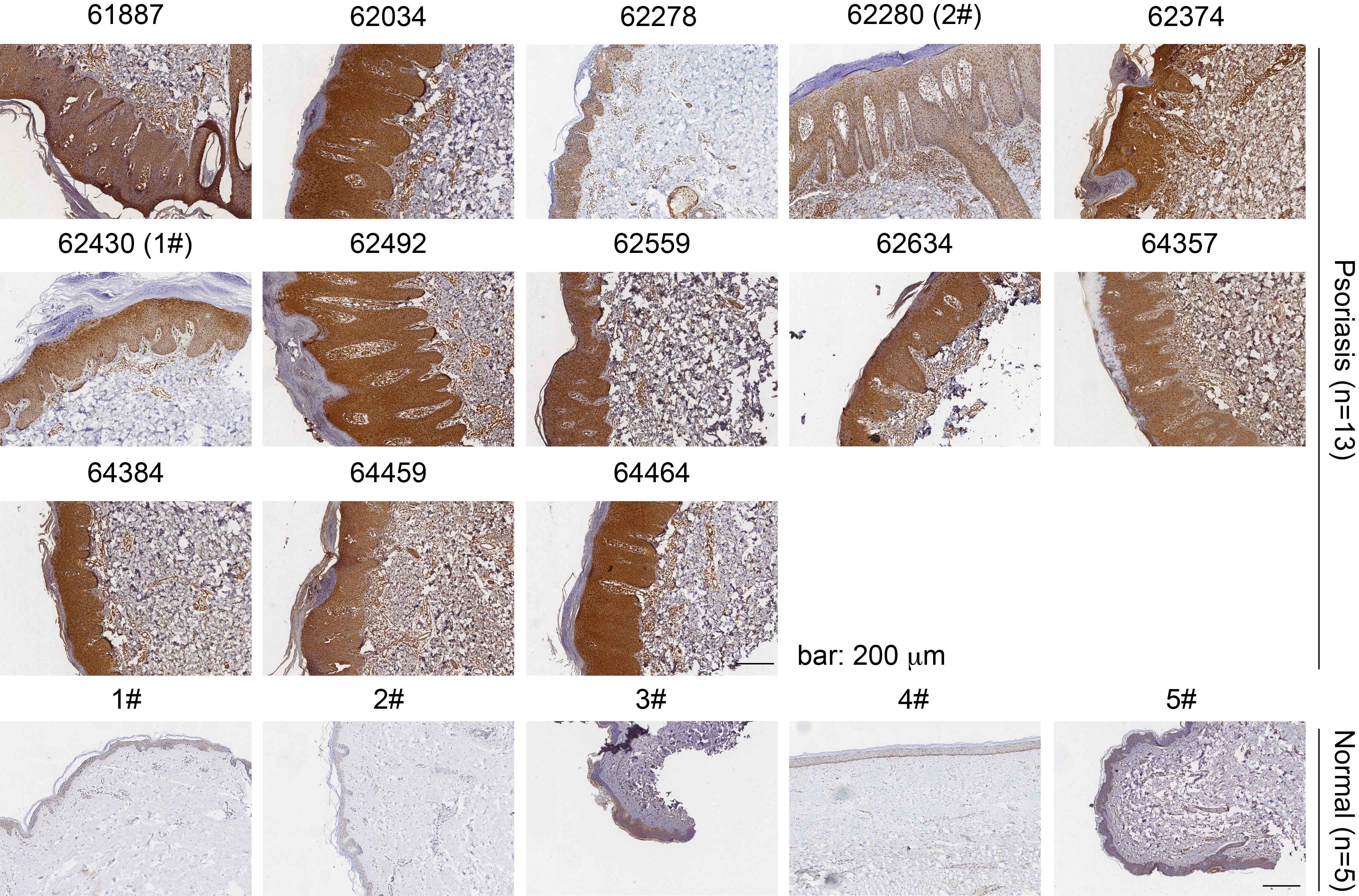

Supplement: Supplementary file 4 — Source Data for Figure 1 [file EMMM-14-e14455-s011.zip › Figure_1/1_C/IHC_SHP2.jpg]

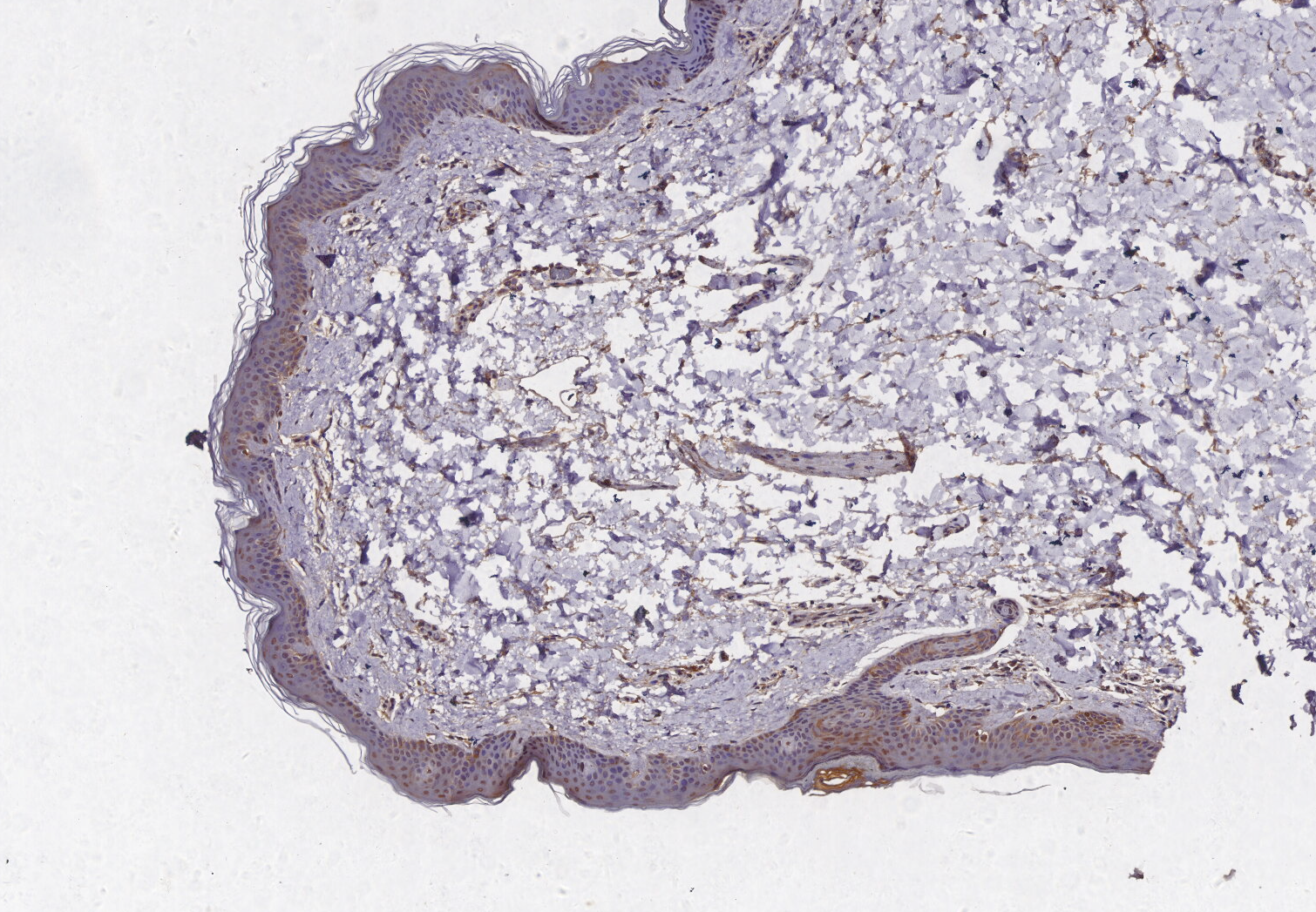

Supplement: Supplementary file 4 — Source Data for Figure 1 [file EMMM-14-e14455-s011.zip › Figure_1/1_C/N1.tiff]

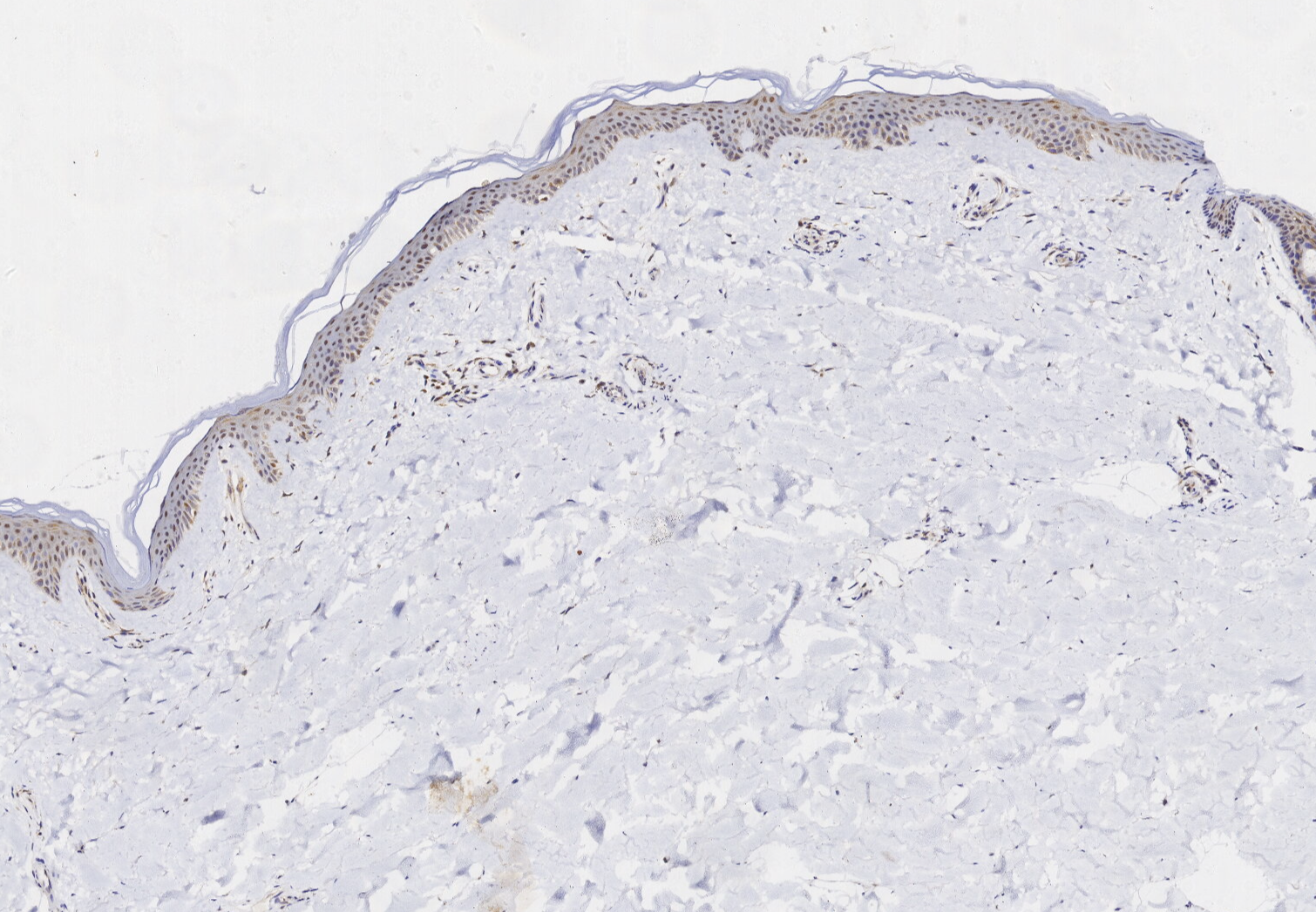

Supplement: Supplementary file 4 — Source Data for Figure 1 [file EMMM-14-e14455-s011.zip › Figure_1/1_C/N2.tiff]

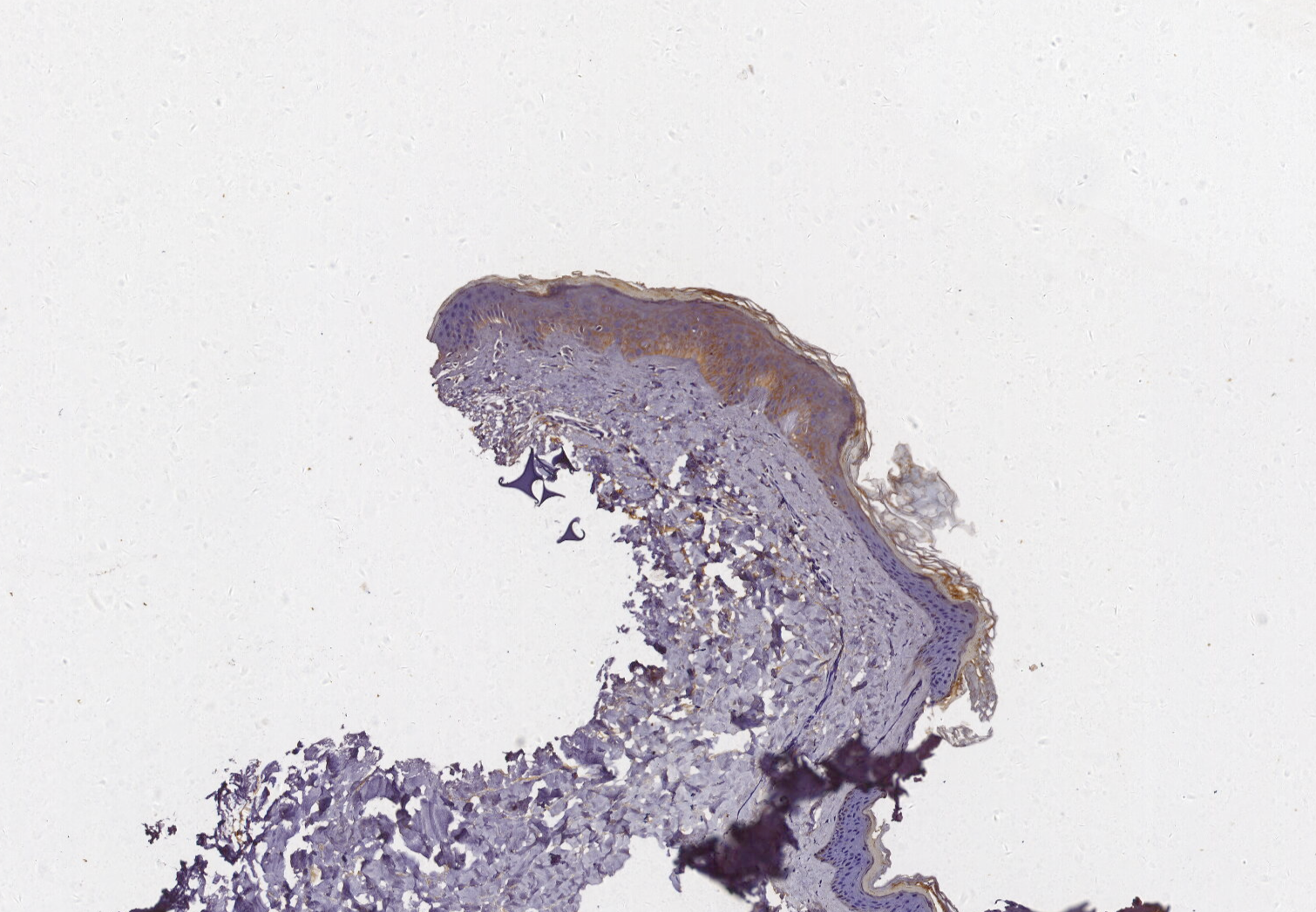

Supplement: Supplementary file 4 — Source Data for Figure 1 [file EMMM-14-e14455-s011.zip › Figure_1/1_C/N3.tiff]

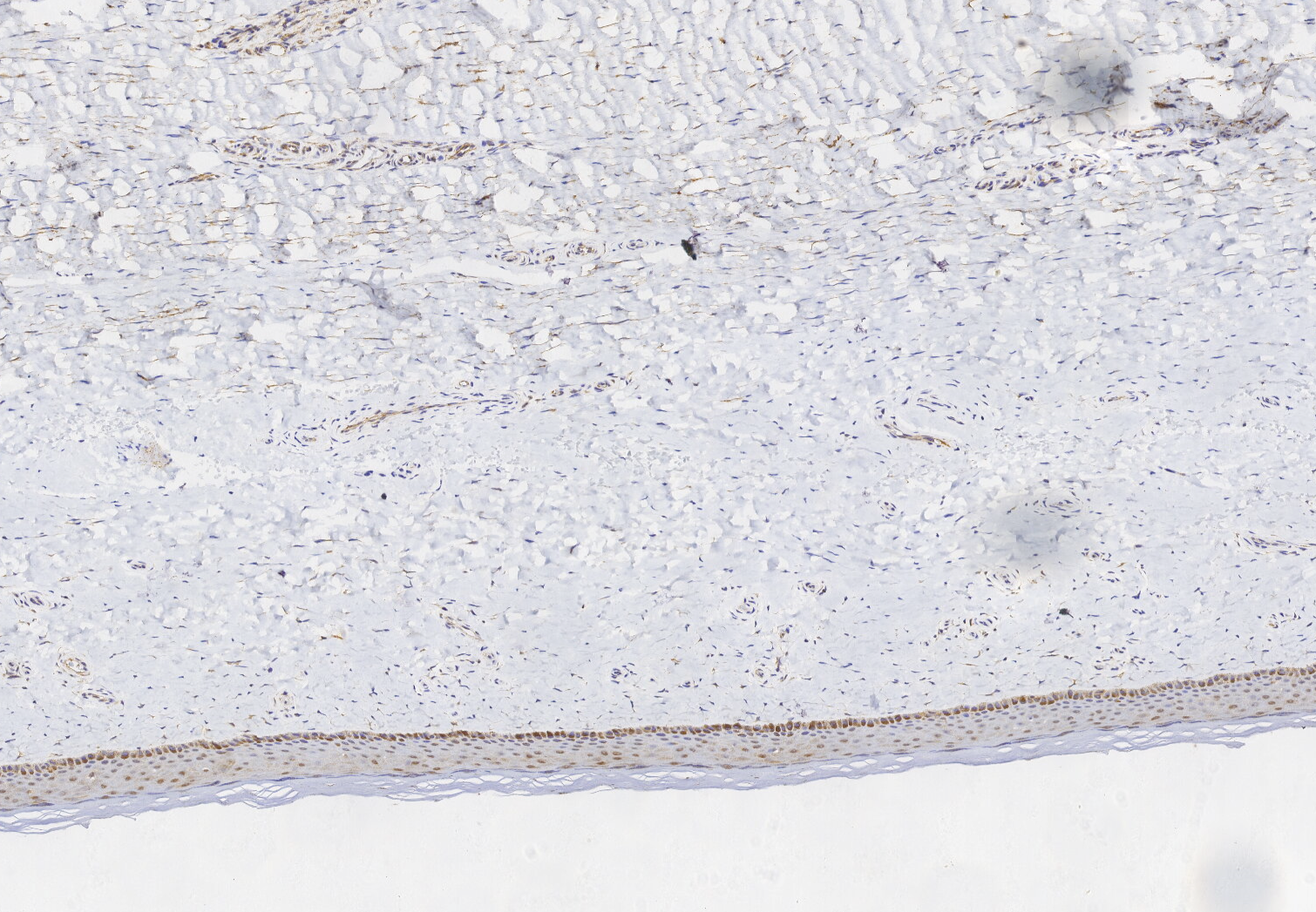

Supplement: Supplementary file 4 — Source Data for Figure 1 [file EMMM-14-e14455-s011.zip › Figure_1/1_C/N4.tiff]

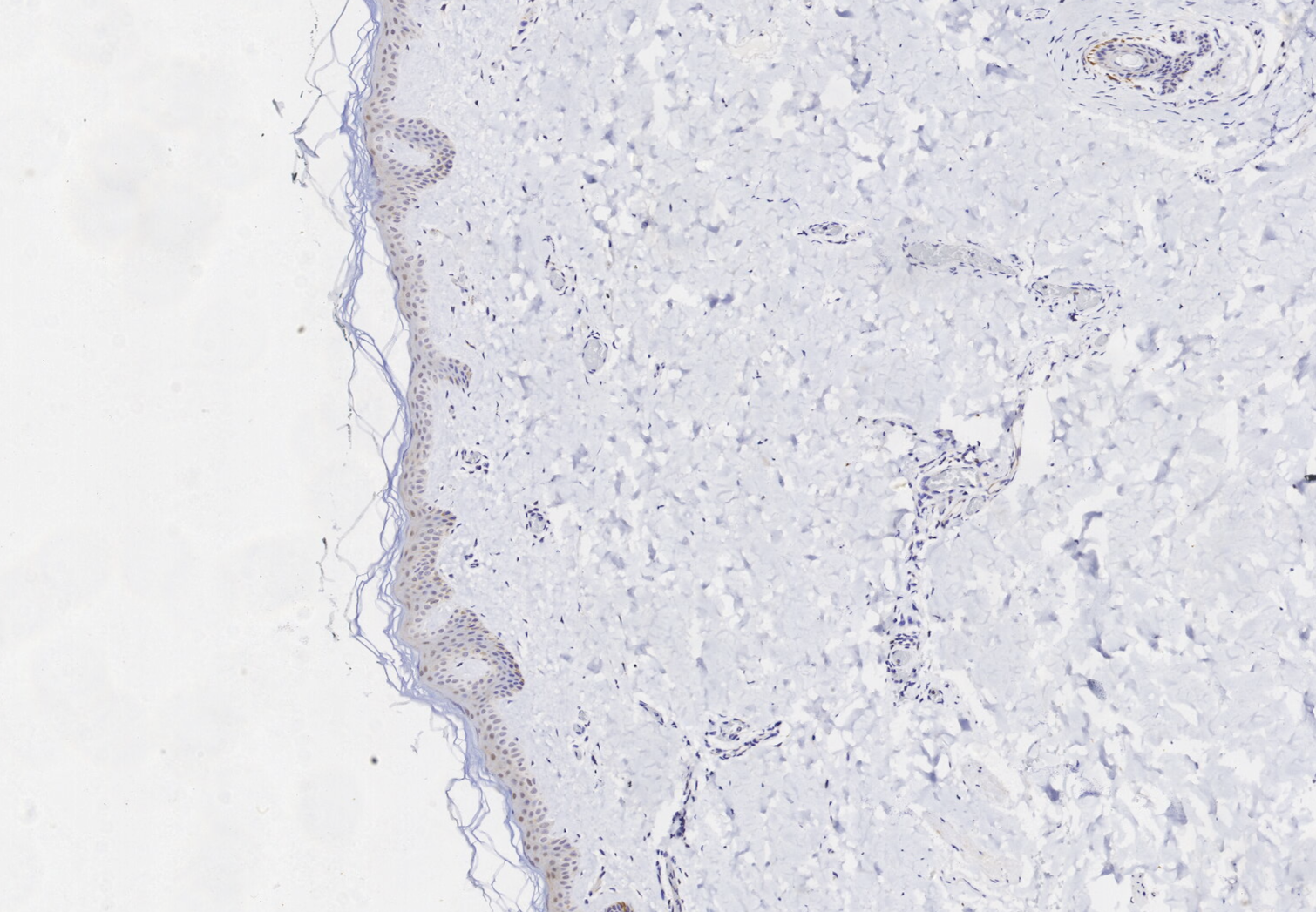

Supplement: Supplementary file 4 — Source Data for Figure 1 [file EMMM-14-e14455-s011.zip › Figure_1/1_C/N5.tiff]

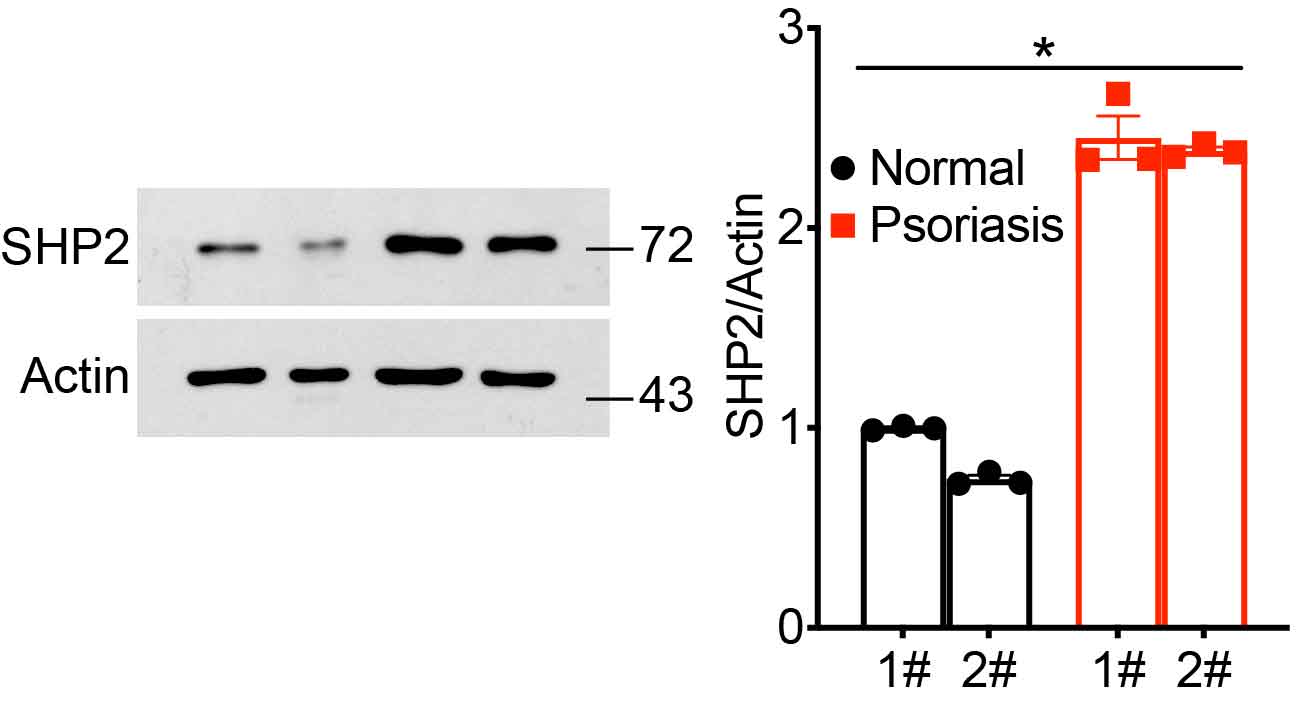

Supplement: Supplementary file 4 — Source Data for Figure 1 [file EMMM-14-e14455-s011.zip › Figure_1/1_D/1_D.jpg]

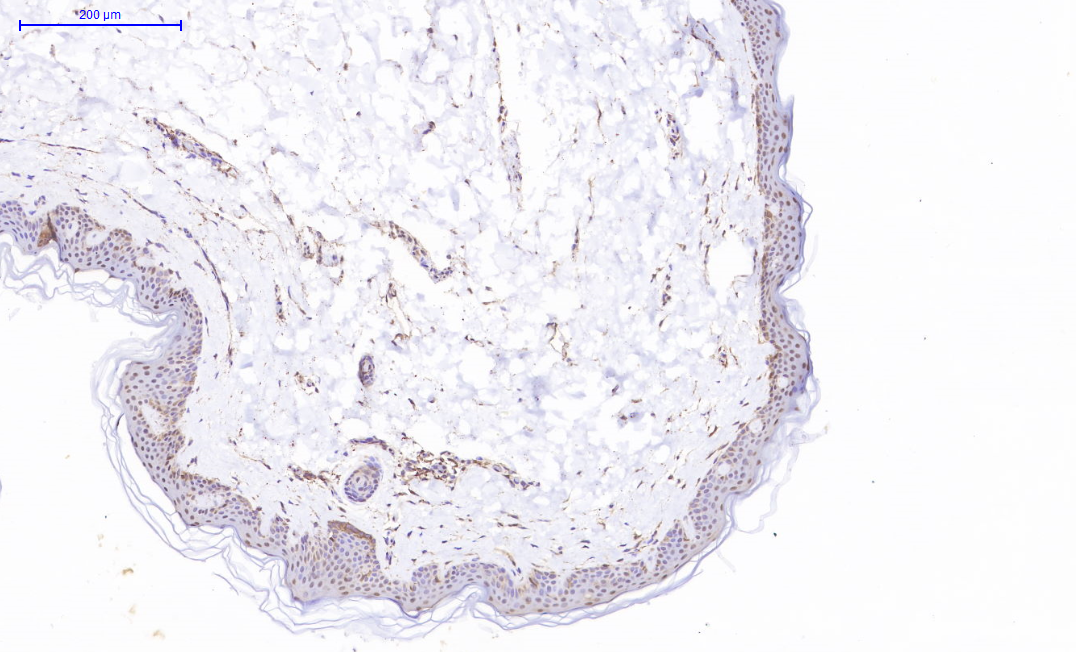

Supplement: Supplementary file 4 — Source Data for Figure 1 [file EMMM-14-e14455-s011.zip › Figure_1/1_F/Normal-100X.tif]

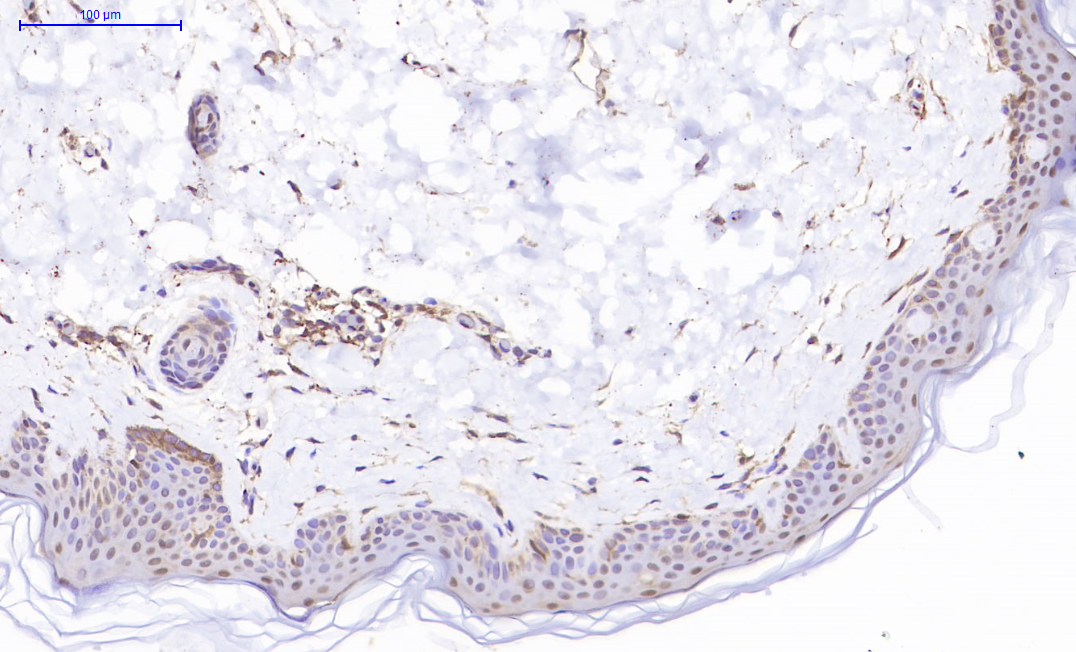

Supplement: Supplementary file 4 — Source Data for Figure 1 [file EMMM-14-e14455-s011.zip › Figure_1/1_F/Normal-200X.tif]

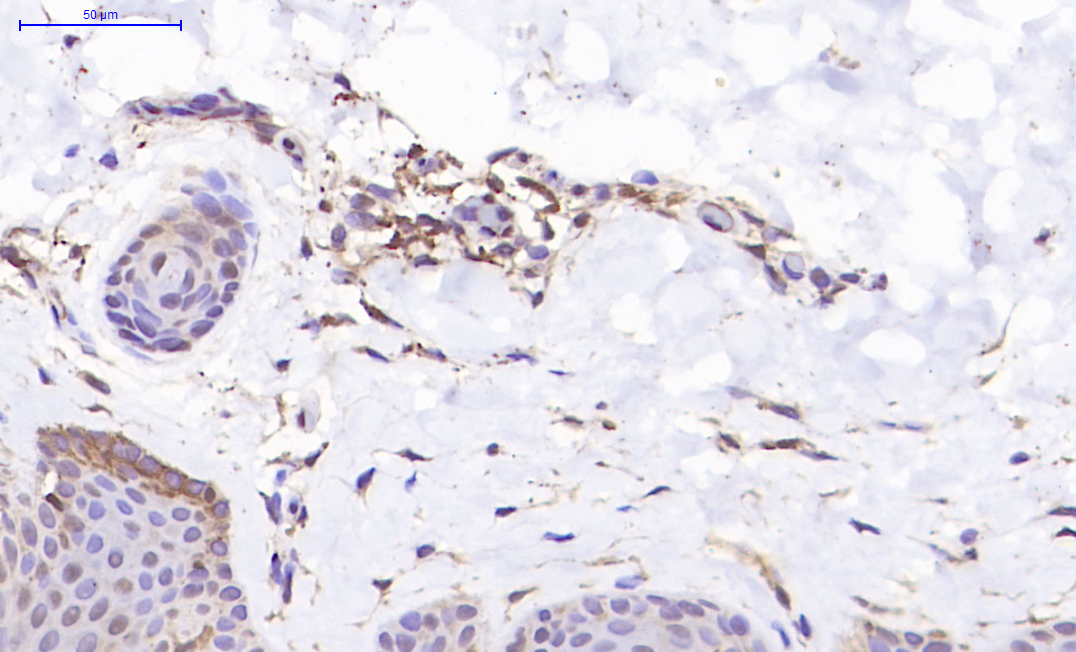

Supplement: Supplementary file 4 — Source Data for Figure 1 [file EMMM-14-e14455-s011.zip › Figure_1/1_F/Normal-400X.tif]

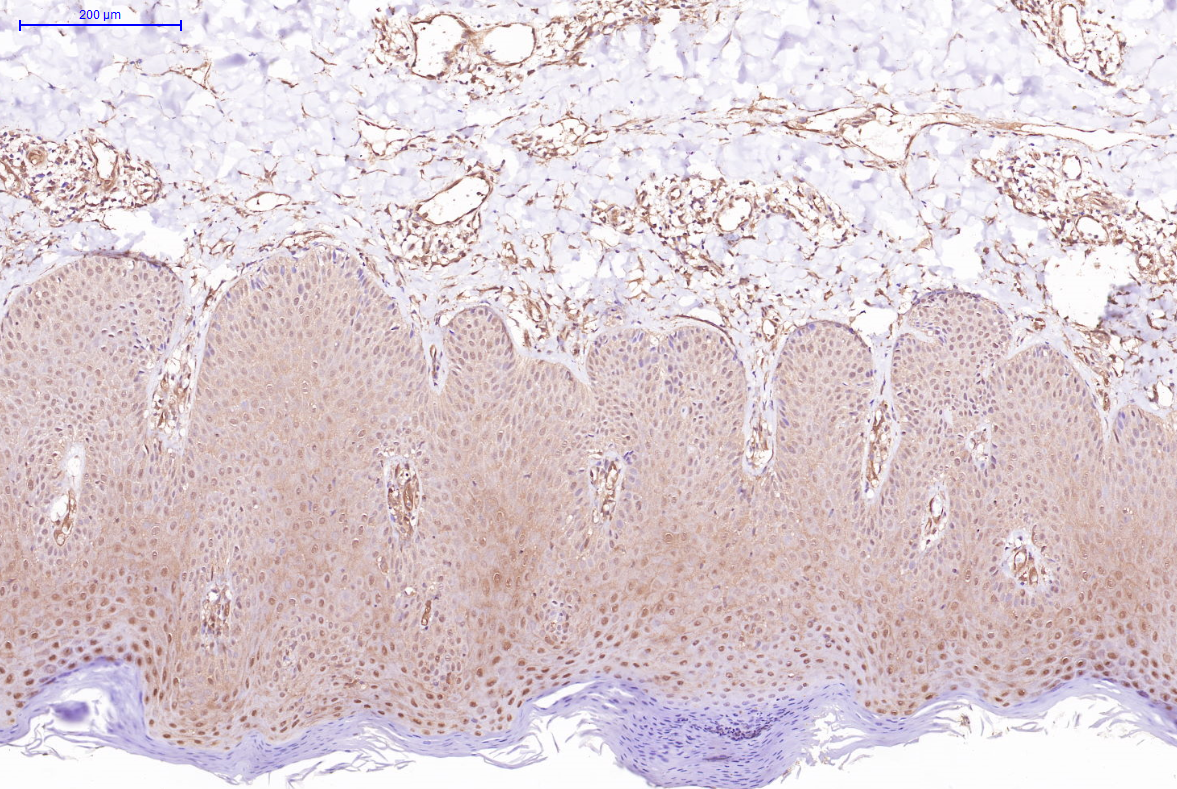

Supplement: Supplementary file 4 — Source Data for Figure 1 [file EMMM-14-e14455-s011.zip › Figure_1/1_F/Psoriasis-100X.tif]

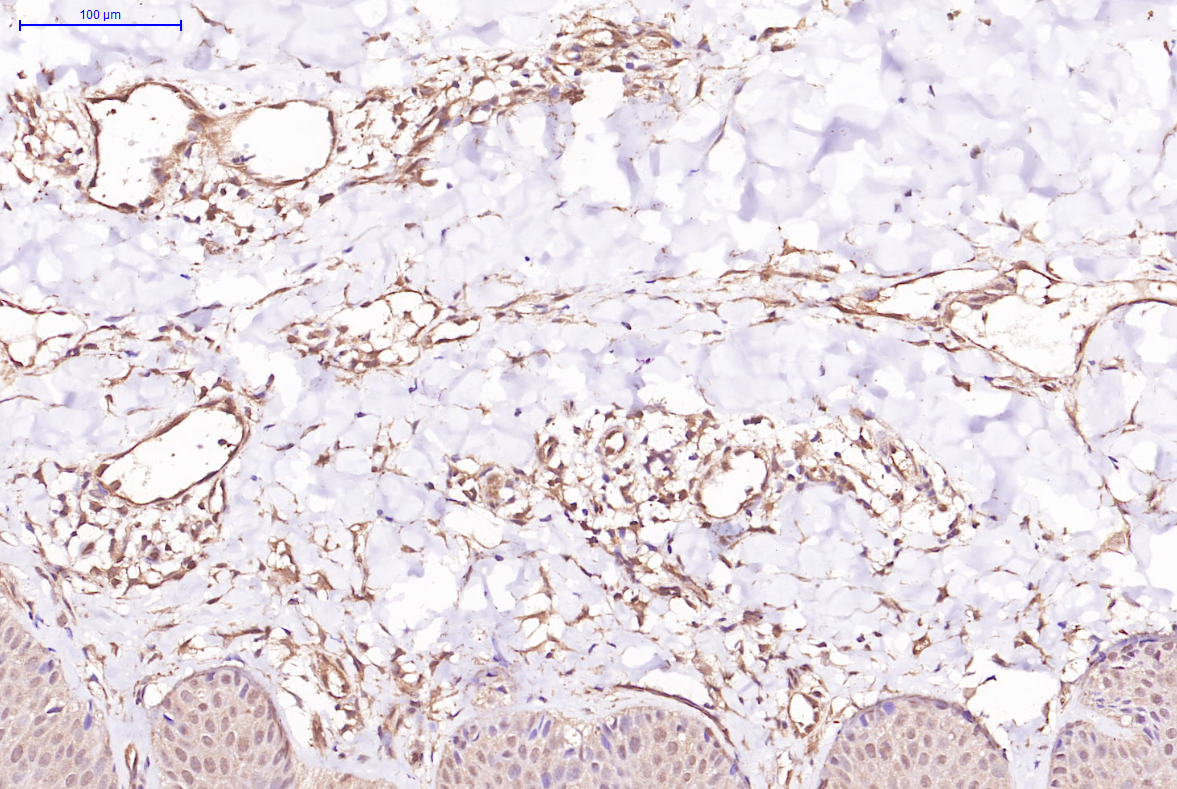

Supplement: Supplementary file 4 — Source Data for Figure 1 [file EMMM-14-e14455-s011.zip › Figure_1/1_F/Psoriasis-200X.tif]

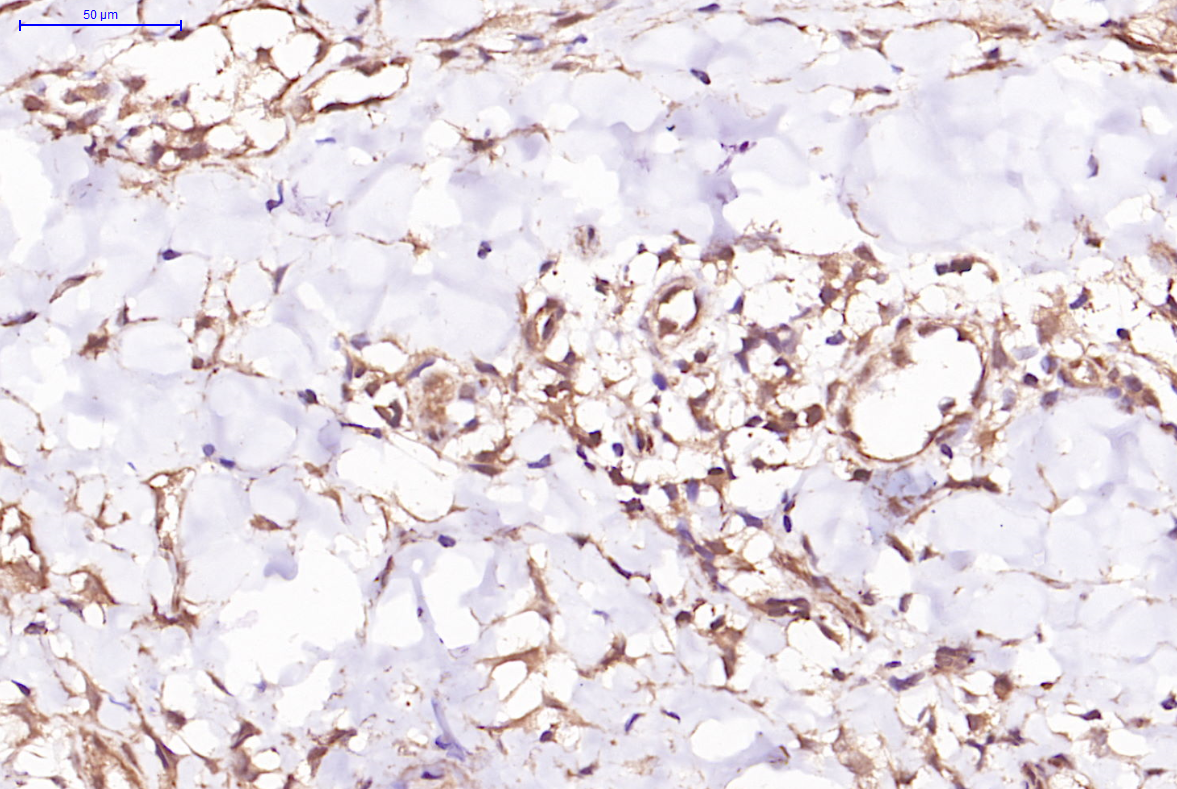

Supplement: Supplementary file 4 — Source Data for Figure 1 [file EMMM-14-e14455-s011.zip › Figure_1/1_F/Psoriasis-400X.tif]

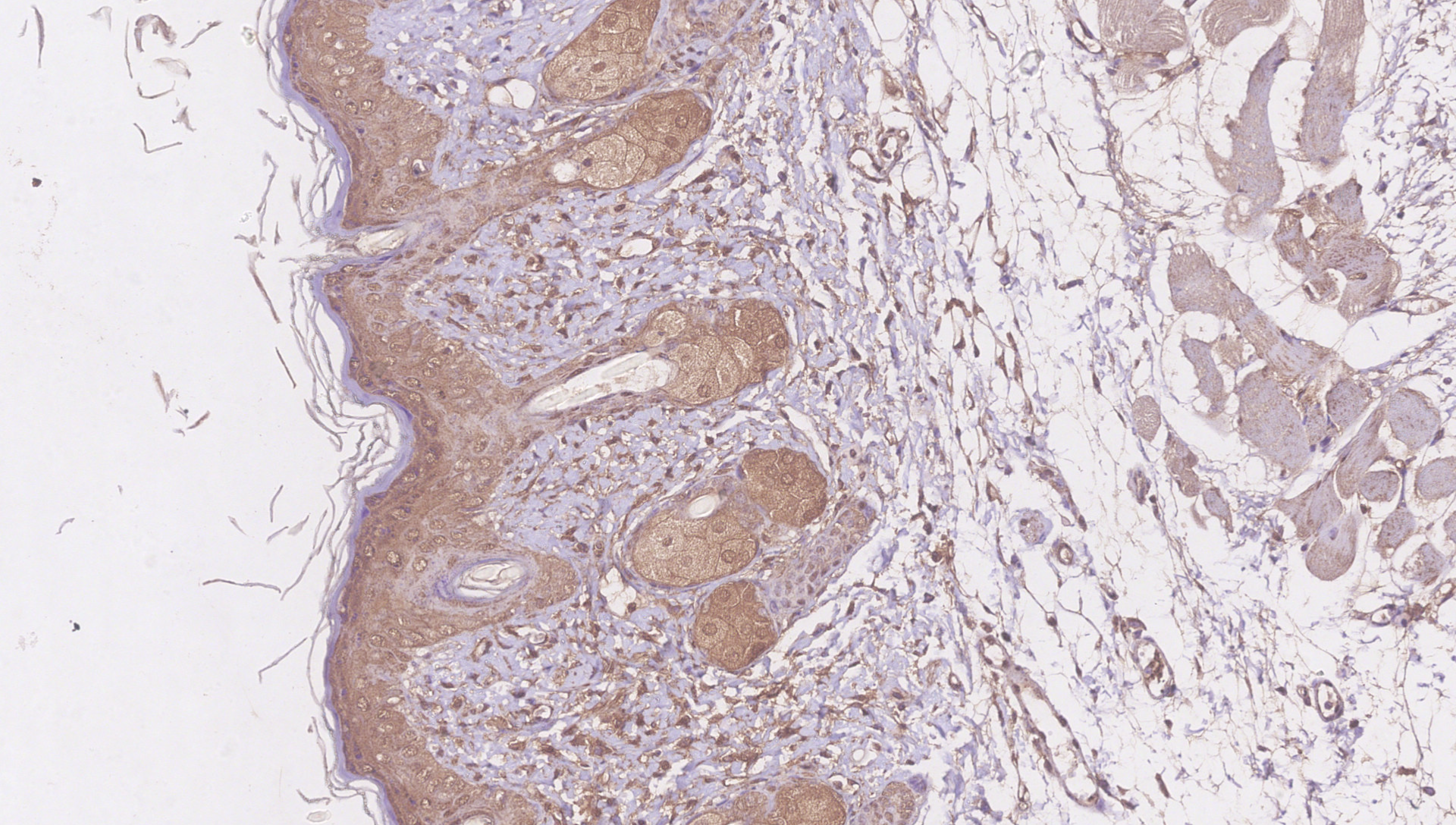

Supplement: Supplementary file 4 — Source Data for Figure 1 [file EMMM-14-e14455-s011.zip › Figure_1/1_H/IMQ_200X.tif]

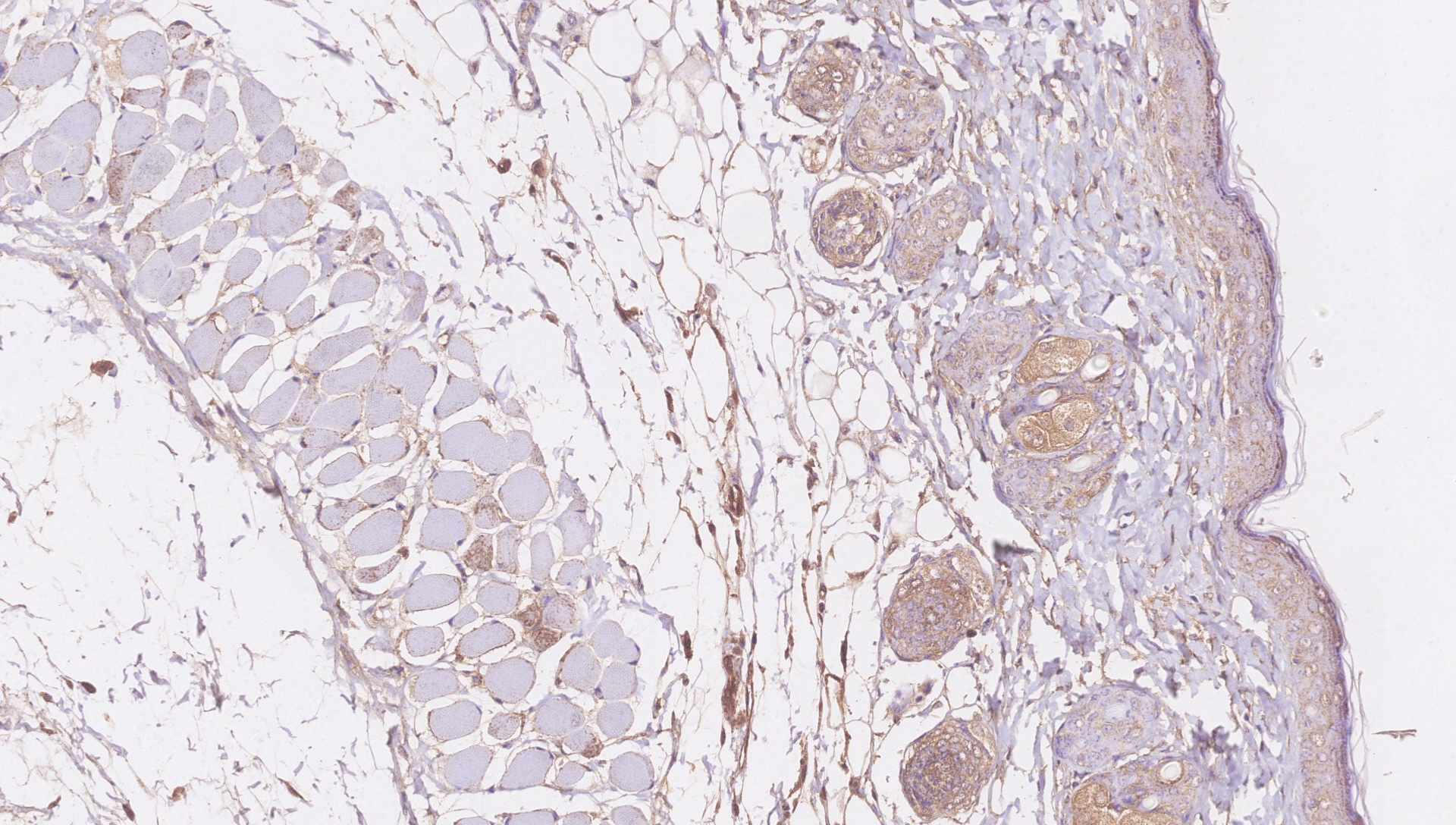

Supplement: Supplementary file 4 — Source Data for Figure 1 [file EMMM-14-e14455-s011.zip › Figure_1/1_H/Sham_200X.tif]

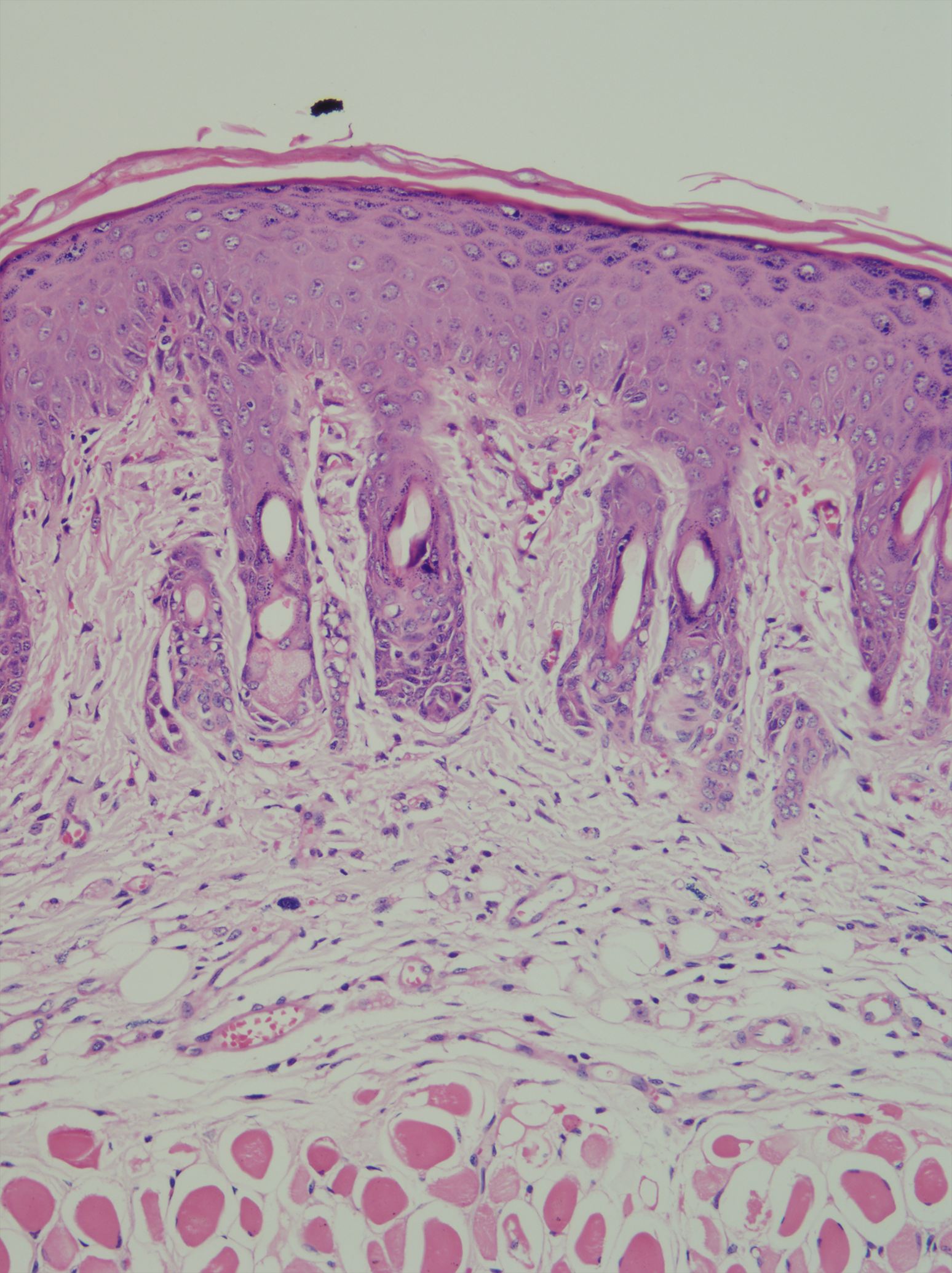

Supplement: Supplementary file 5 — Source Data for Figure 2 [file EMMM-14-e14455-s003.zip › Figure_2/2_A/H&E/IMQ.tif]

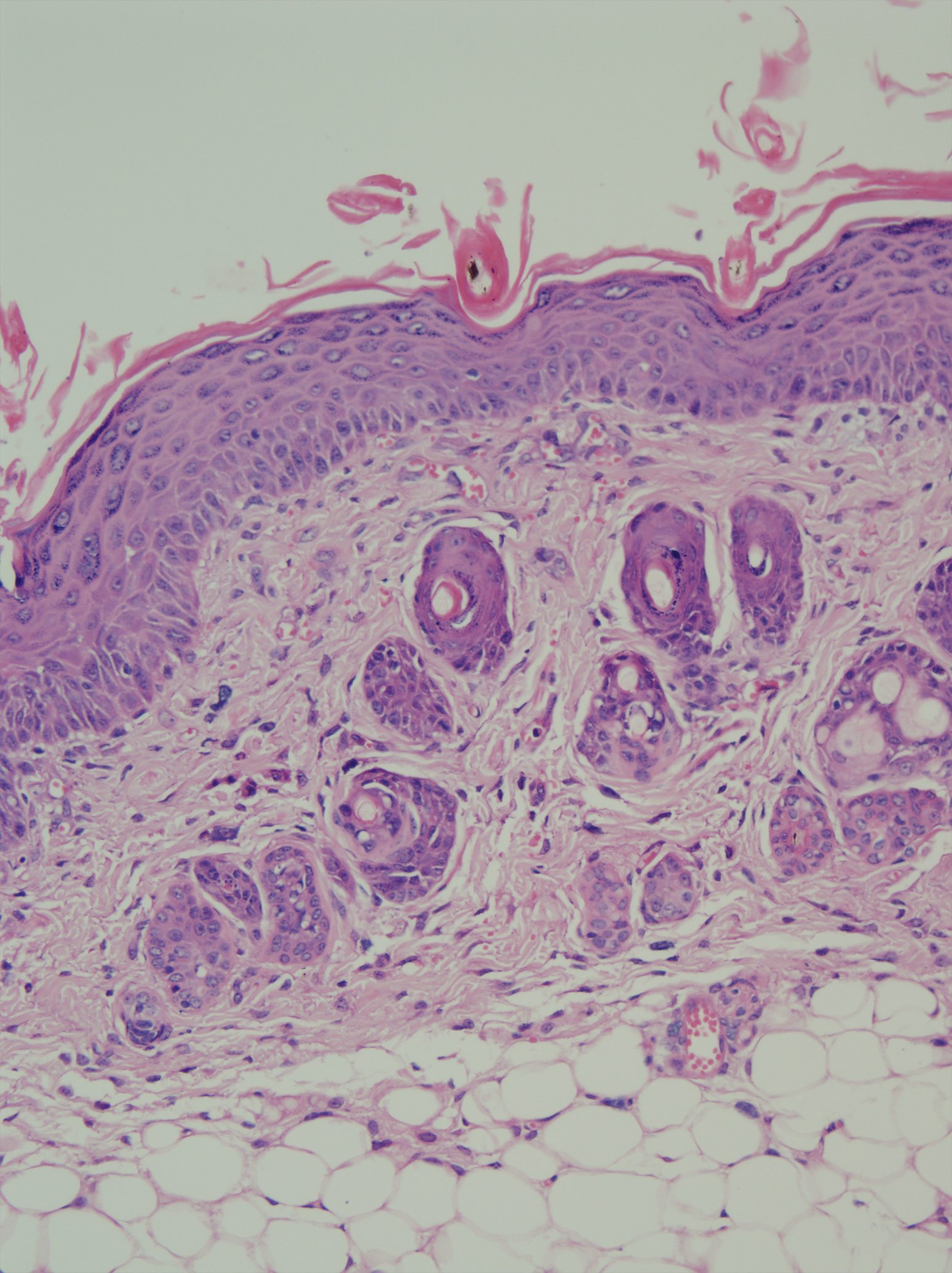

Supplement: Supplementary file 5 — Source Data for Figure 2 [file EMMM-14-e14455-s003.zip › Figure_2/2_A/H&E/S1.tif]

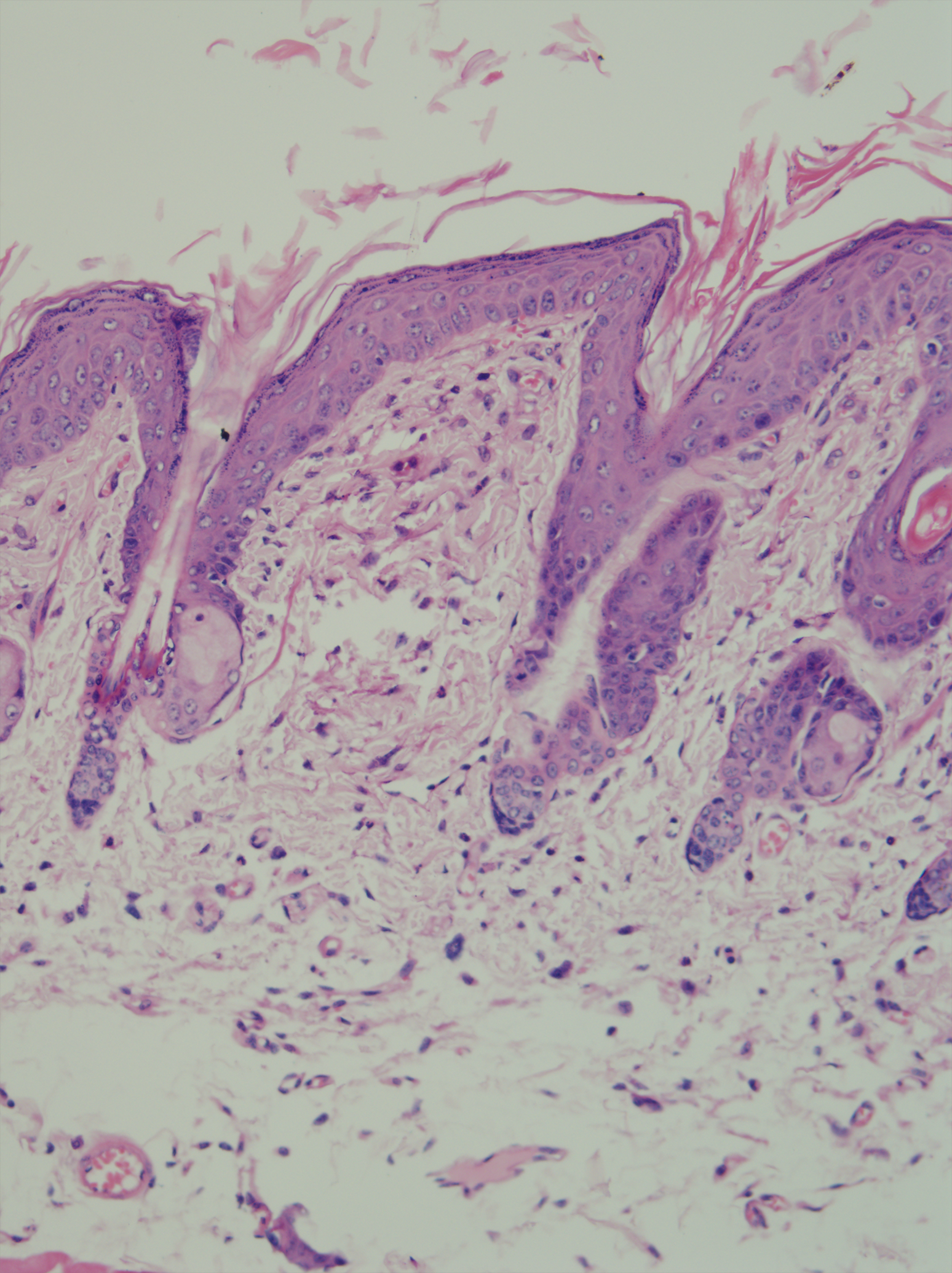

Supplement: Supplementary file 5 — Source Data for Figure 2 [file EMMM-14-e14455-s003.zip › Figure_2/2_A/H&E/S10.tif]

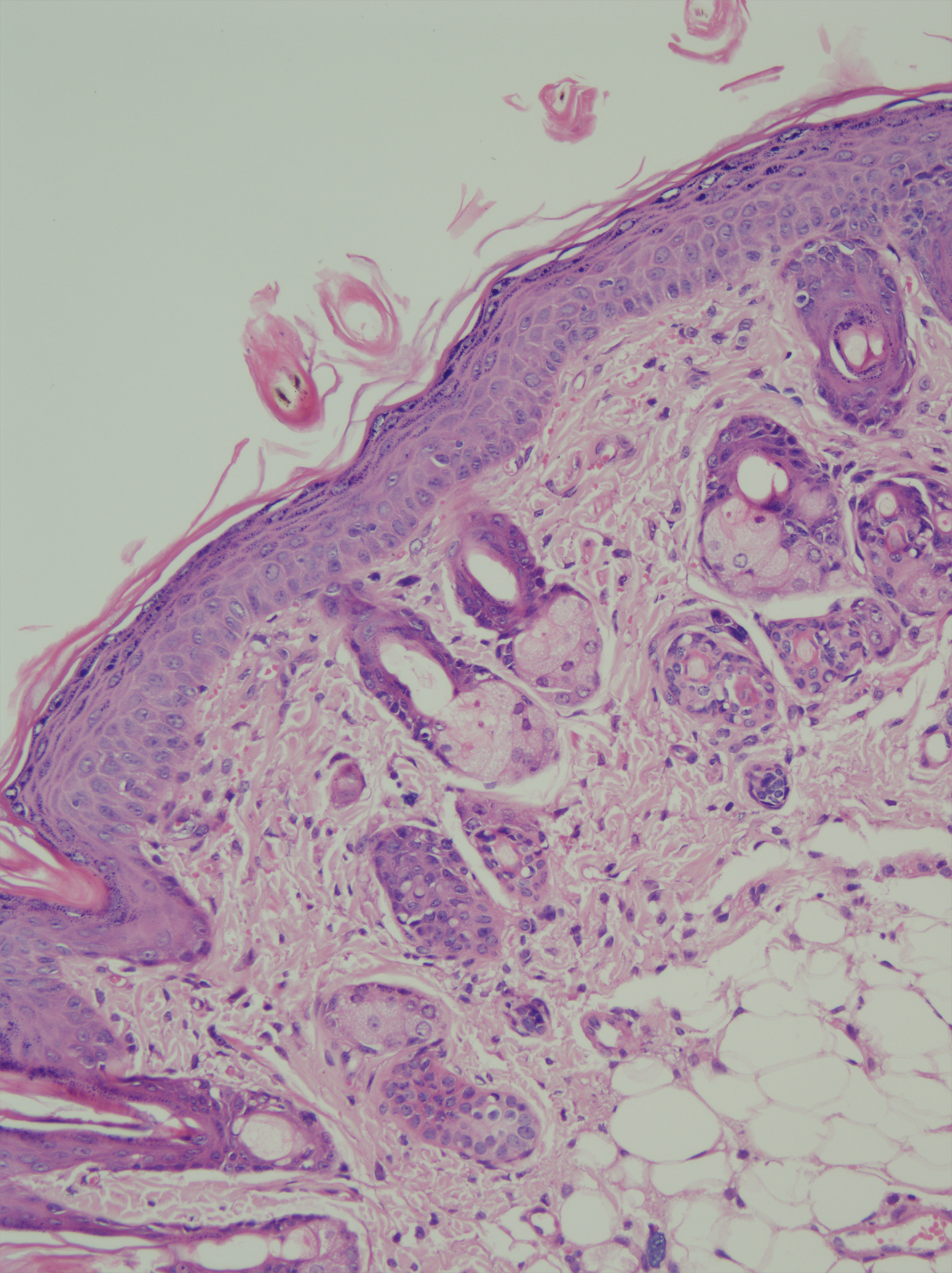

Supplement: Supplementary file 5 — Source Data for Figure 2 [file EMMM-14-e14455-s003.zip › Figure_2/2_A/H&E/S3.tif]

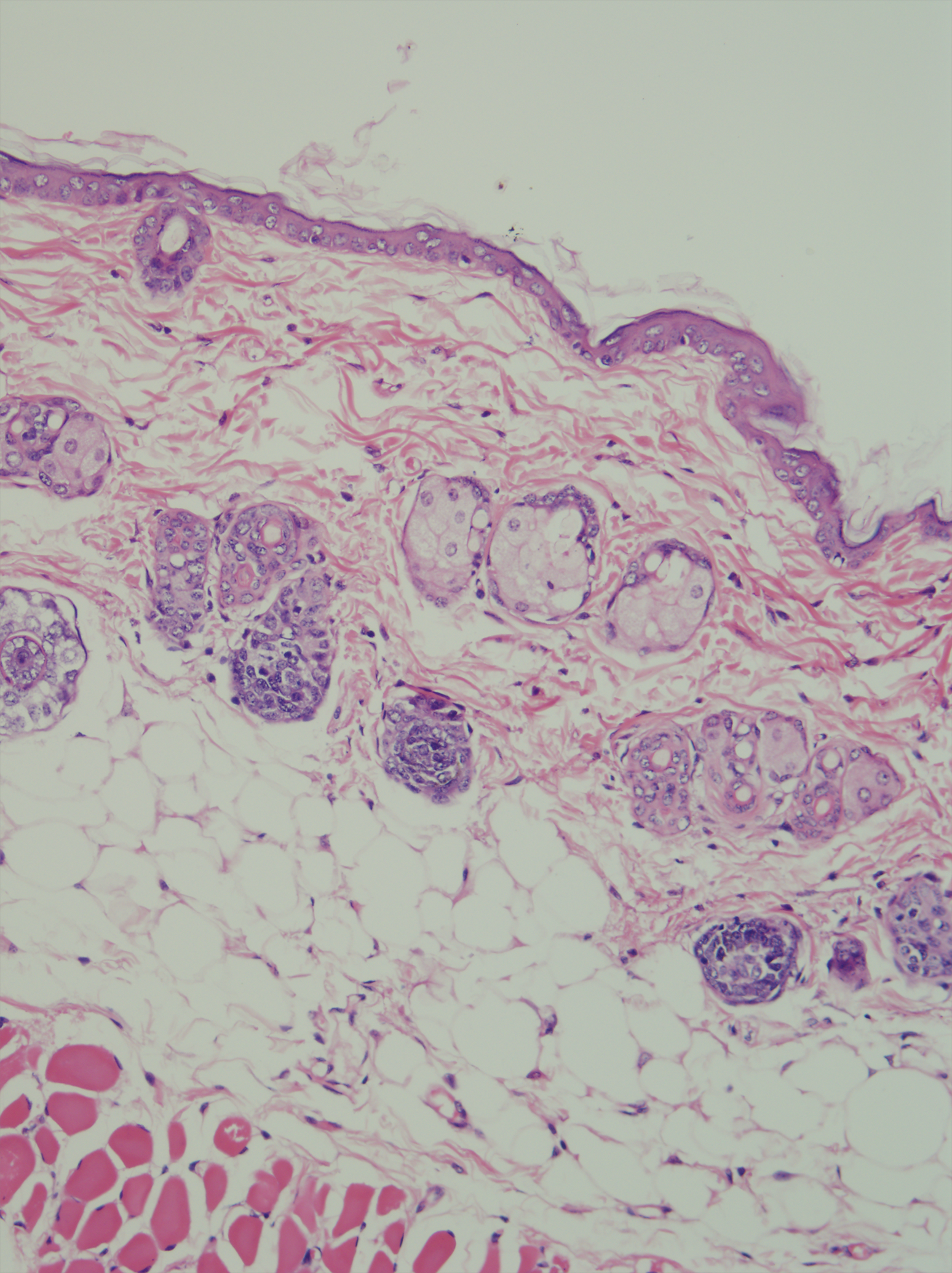

Supplement: Supplementary file 5 — Source Data for Figure 2 [file EMMM-14-e14455-s003.zip › Figure_2/2_A/H&E/Sham.tif]

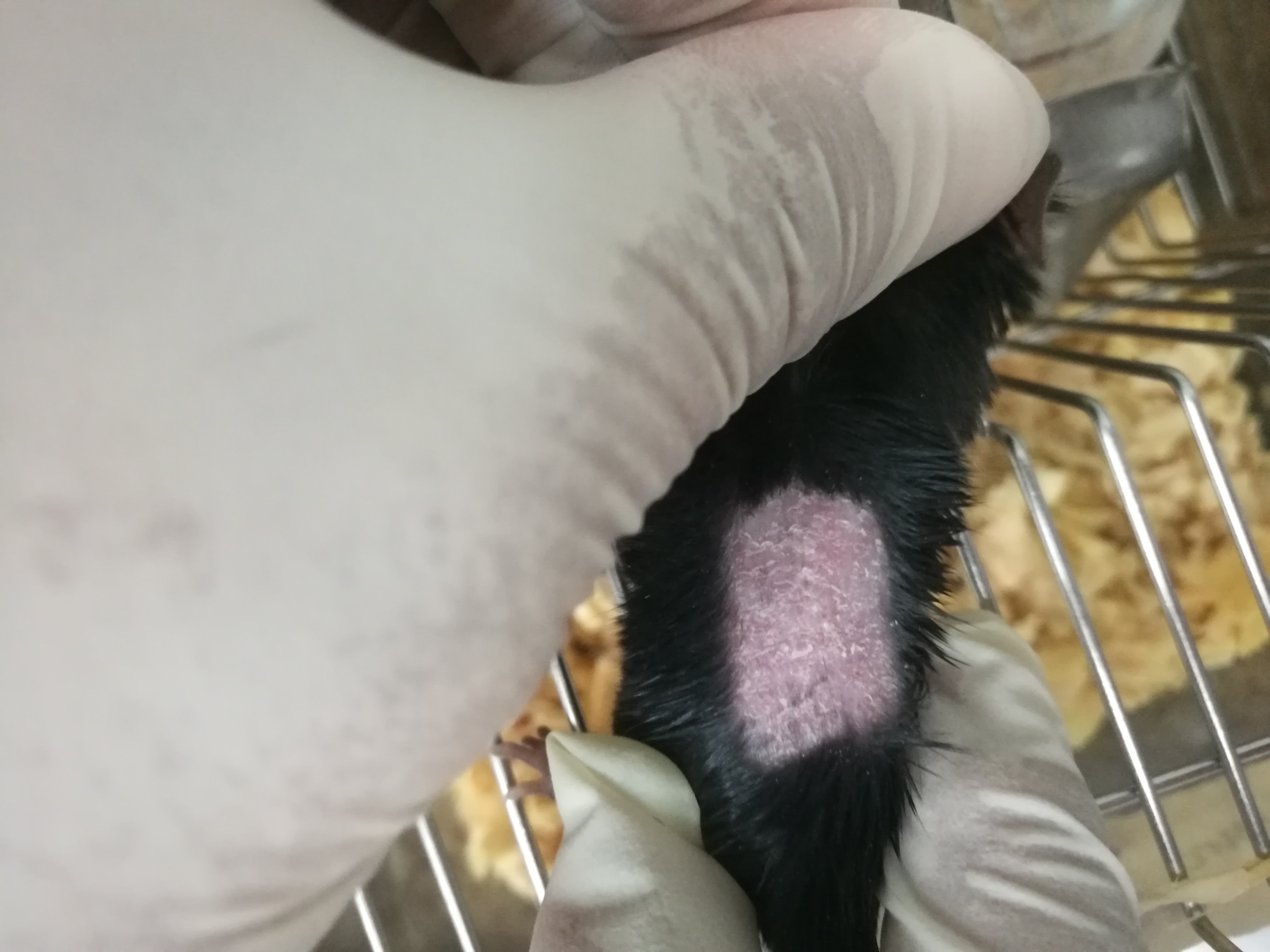

Supplement: Supplementary file 5 — Source Data for Figure 2 [file EMMM-14-e14455-s003.zip › Figure_2/2_A/Photos/IMQ.jpg]

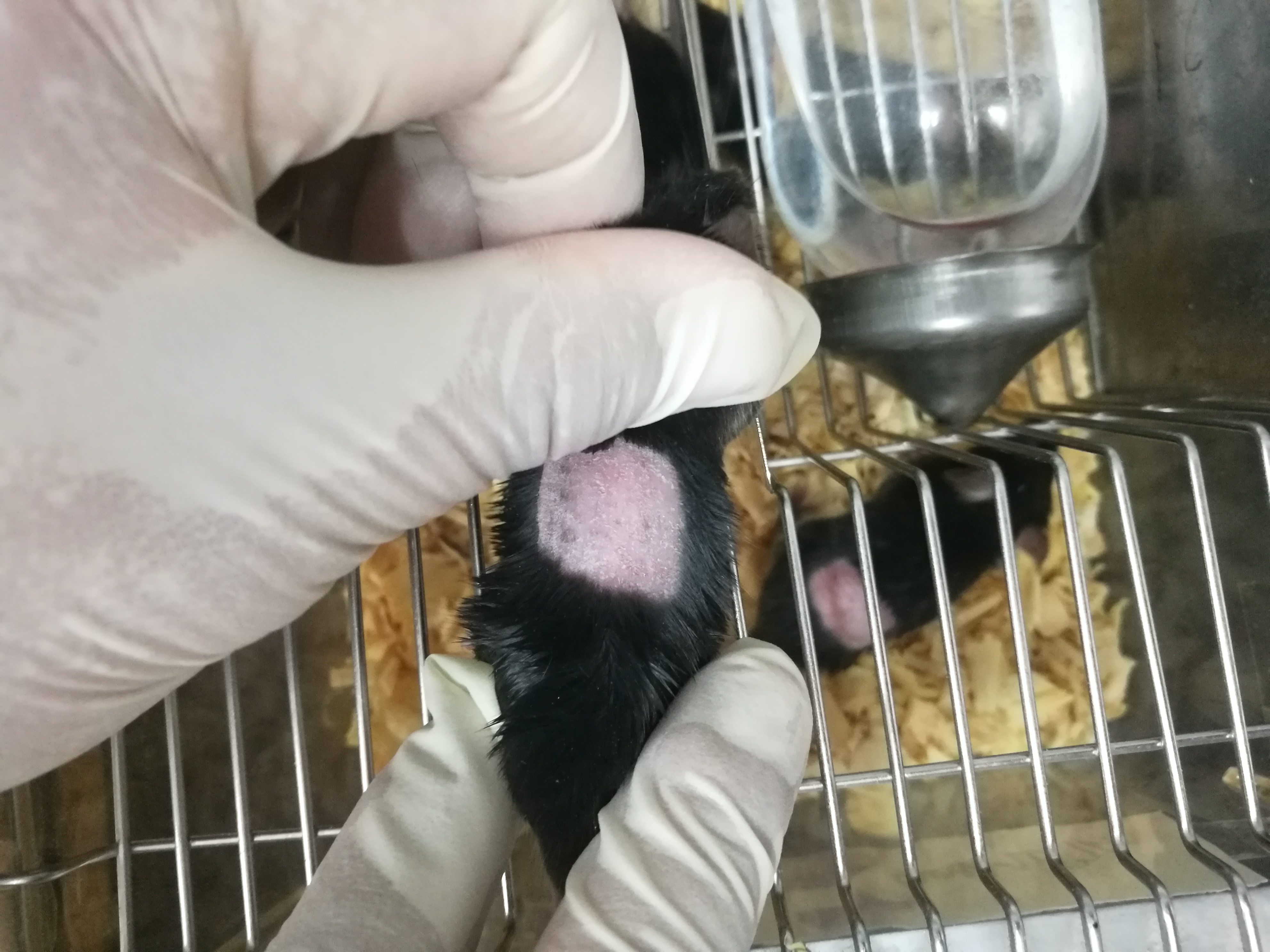

Supplement: Supplementary file 5 — Source Data for Figure 2 [file EMMM-14-e14455-s003.zip › Figure_2/2_A/Photos/S1.jpg]

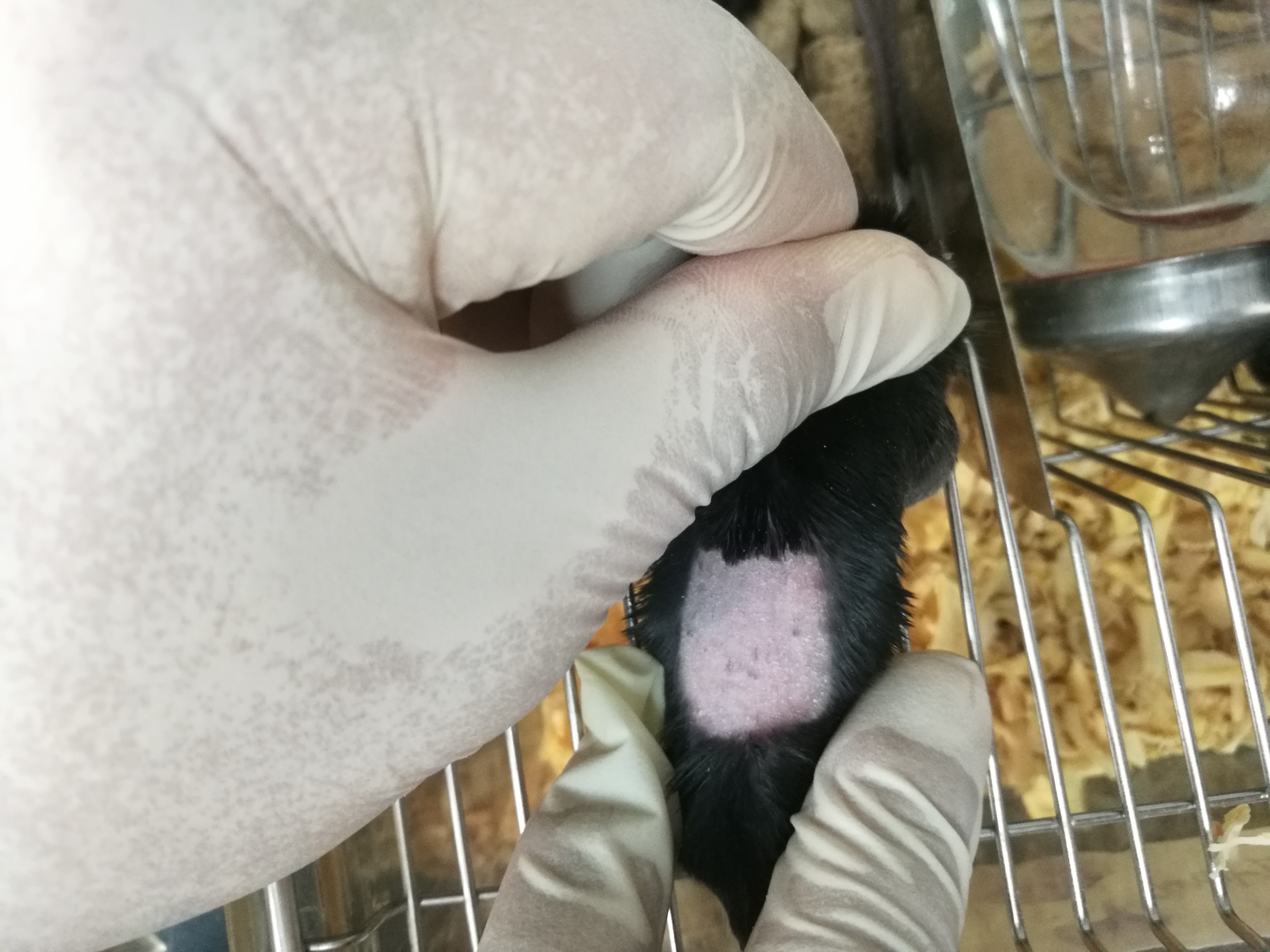

Supplement: Supplementary file 5 — Source Data for Figure 2 [file EMMM-14-e14455-s003.zip › Figure_2/2_A/Photos/S10.jpg]

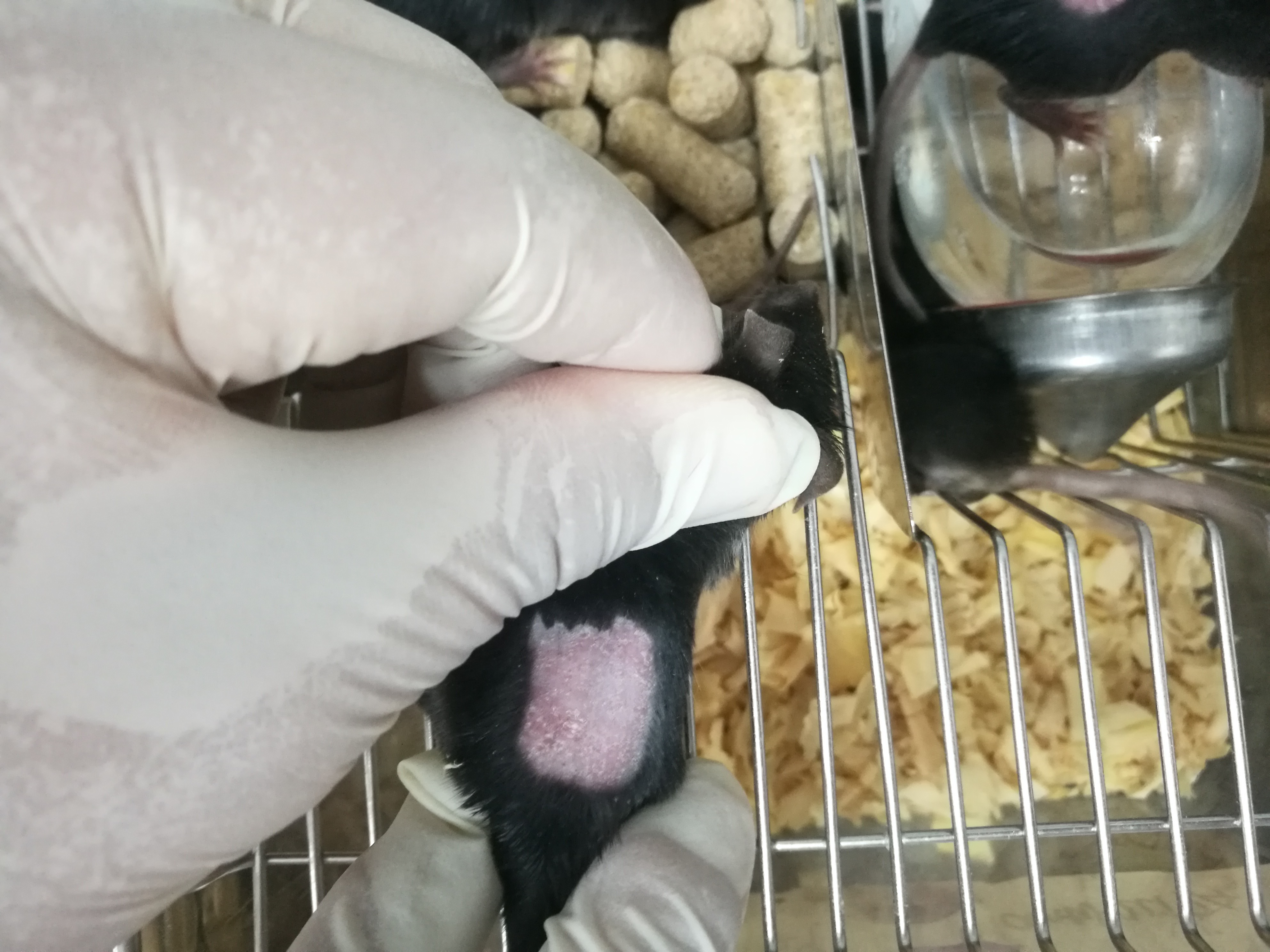

Supplement: Supplementary file 5 — Source Data for Figure 2 [file EMMM-14-e14455-s003.zip › Figure_2/2_A/Photos/S3.jpg]

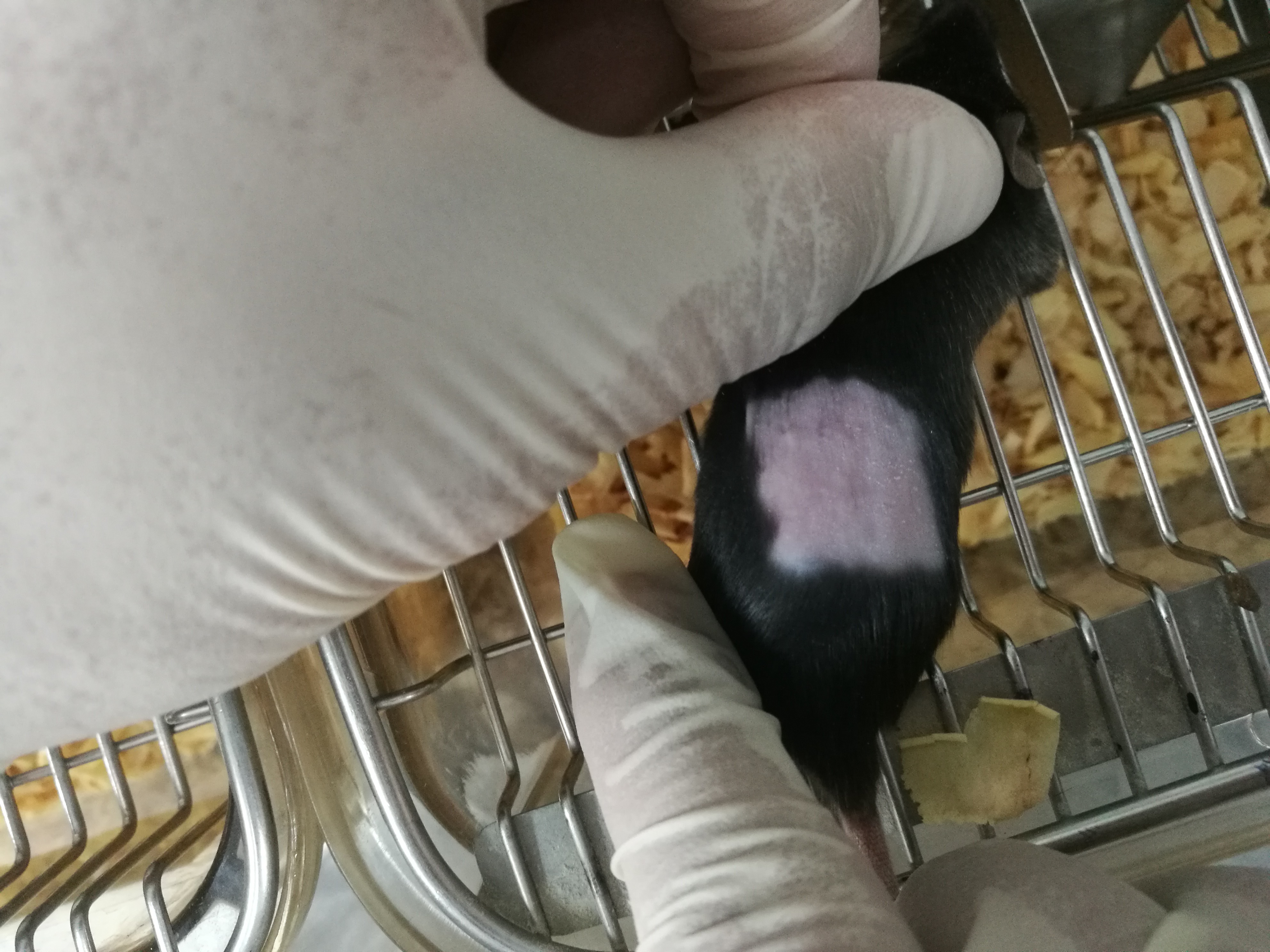

Supplement: Supplementary file 5 — Source Data for Figure 2 [file EMMM-14-e14455-s003.zip › Figure_2/2_A/Photos/Sham.jpg]

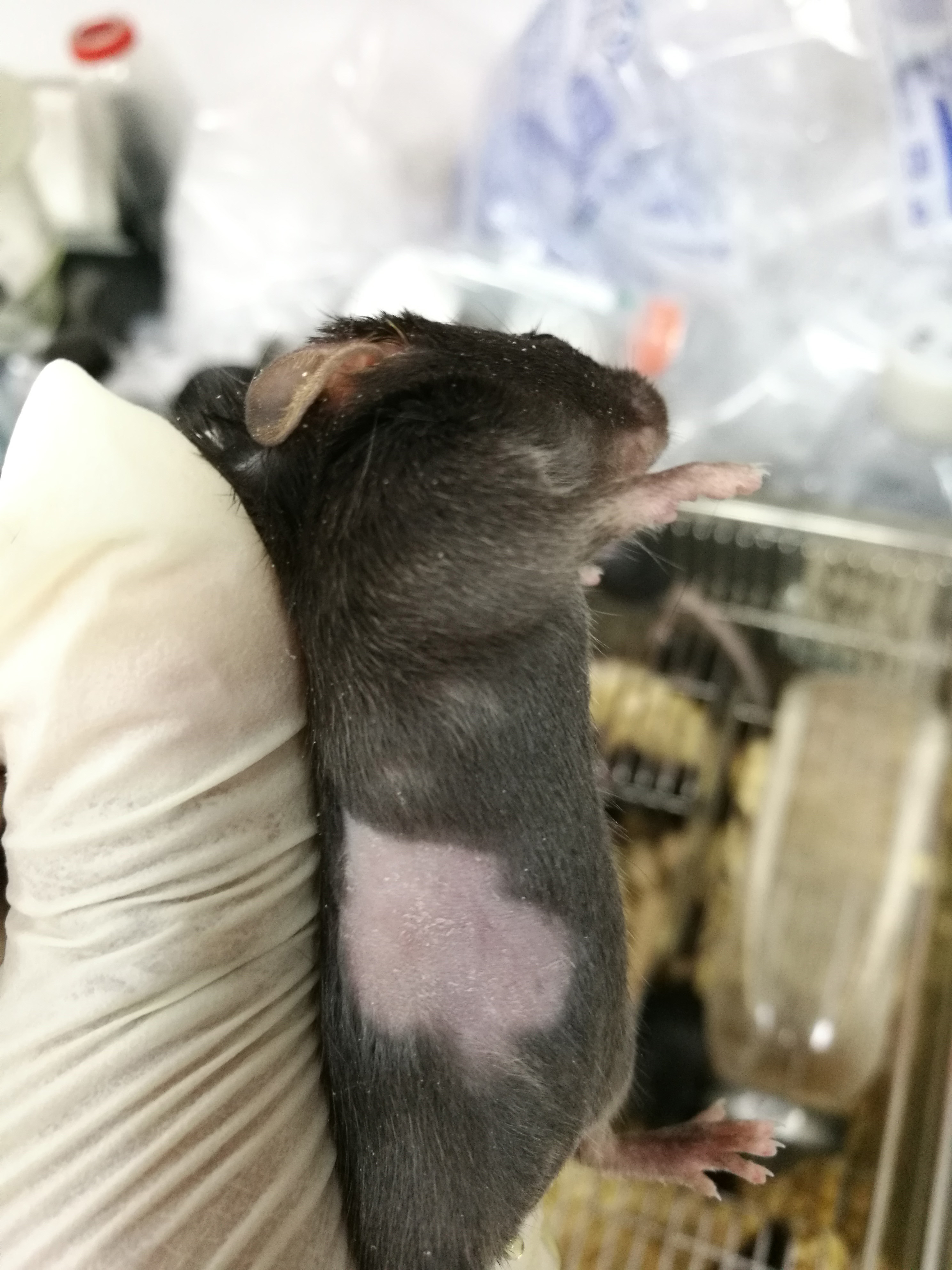

Supplement: Supplementary file 7 — Source Data for Figure 5 [file EMMM-14-e14455-s001.zip › Figure_5-RAW_DATA/5_A/KO_IMQ.jpg]

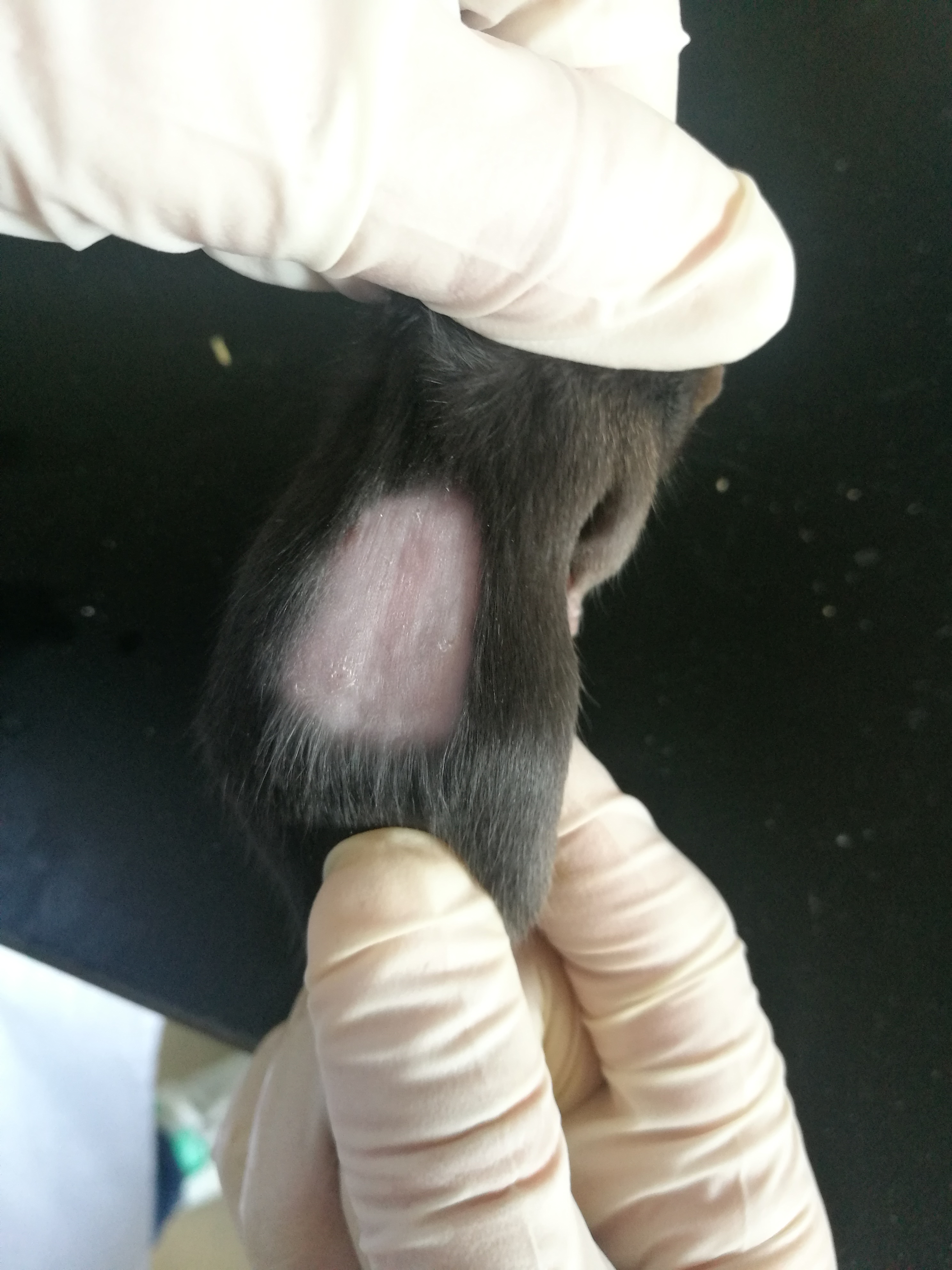

Supplement: Supplementary file 7 — Source Data for Figure 5 [file EMMM-14-e14455-s001.zip › Figure_5-RAW_DATA/5_A/KO_Sham.jpg]

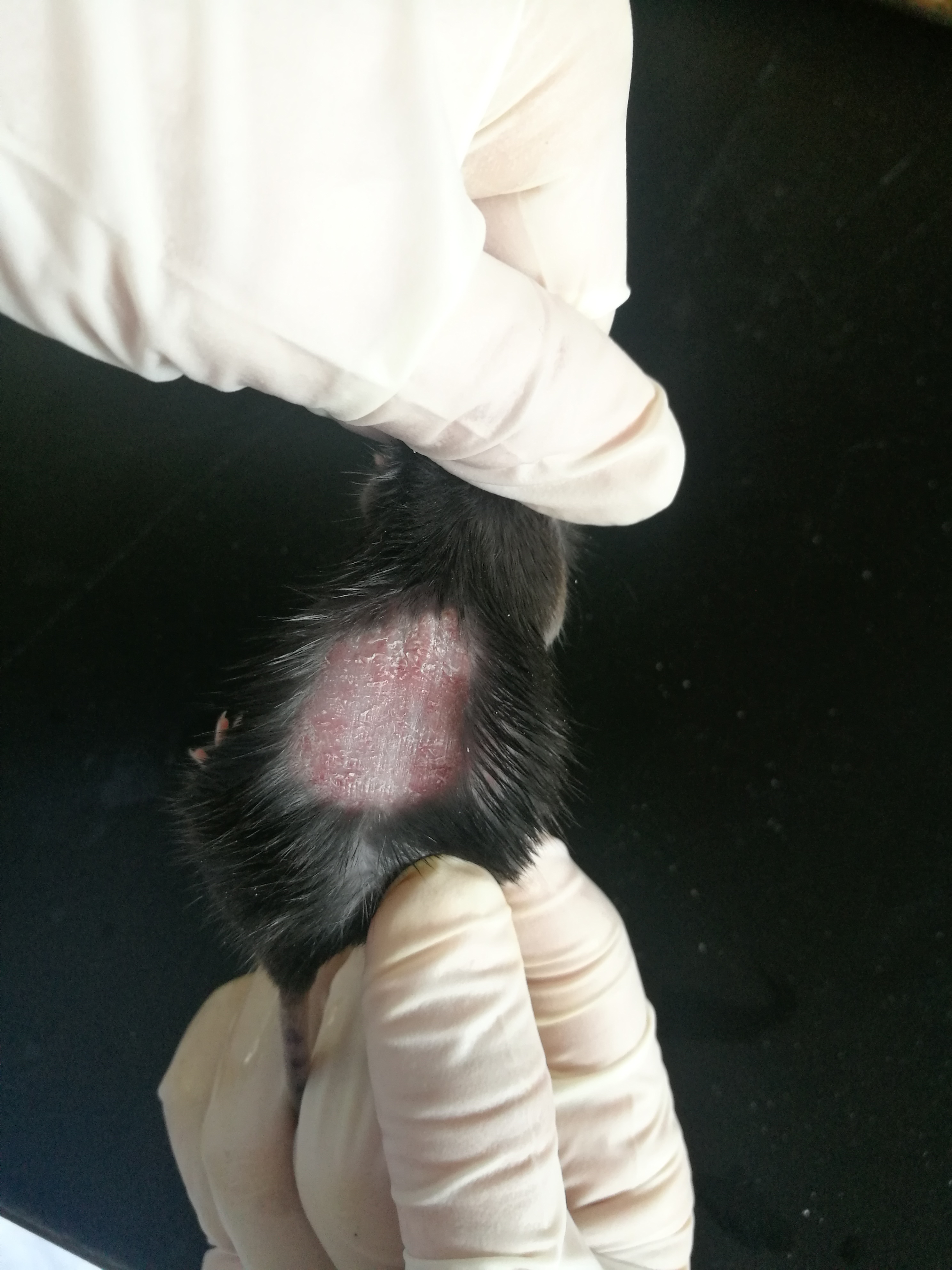

Supplement: Supplementary file 7 — Source Data for Figure 5 [file EMMM-14-e14455-s001.zip › Figure_5-RAW_DATA/5_A/WT_IMQ.jpg]

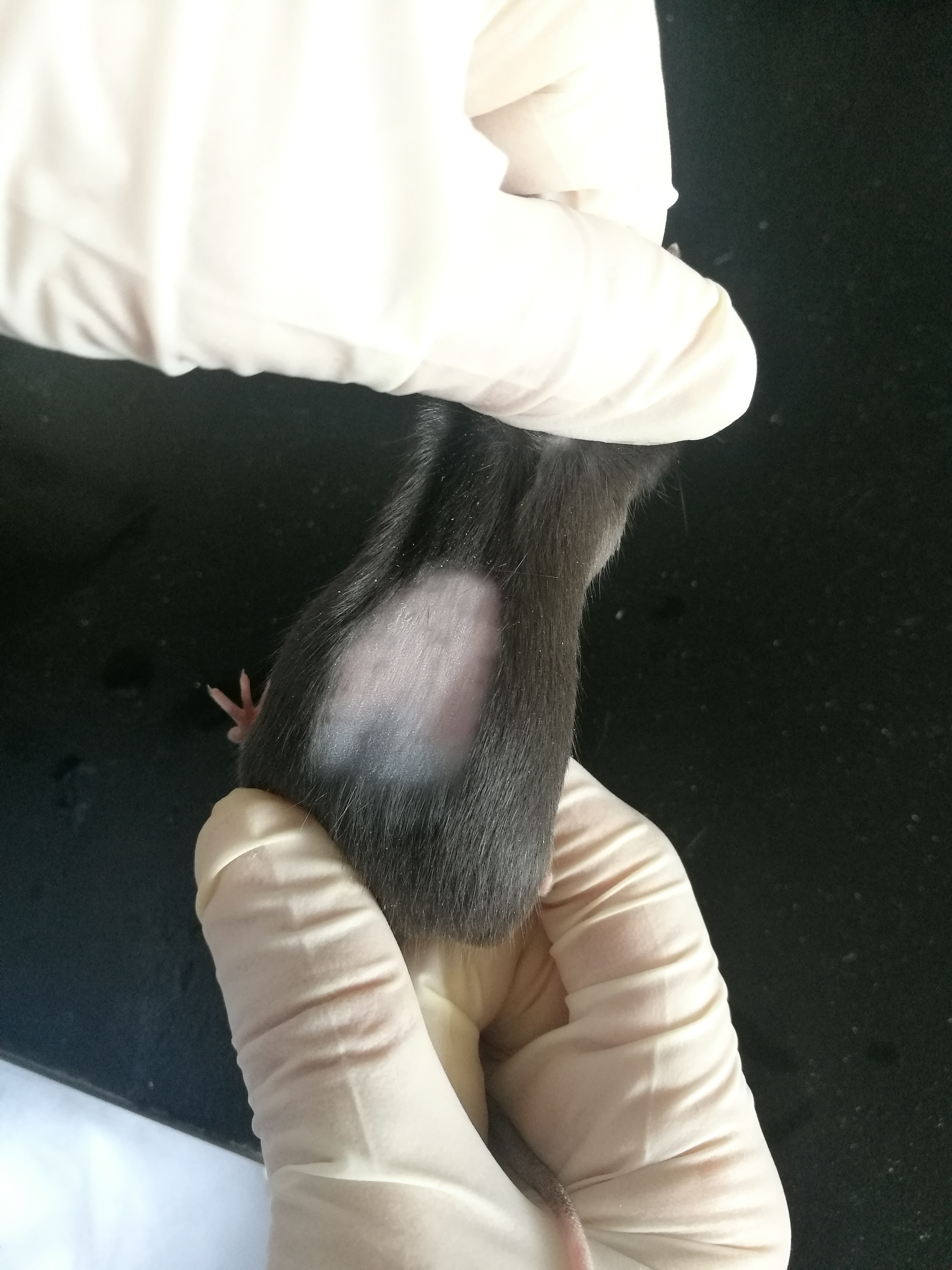

Supplement: Supplementary file 7 — Source Data for Figure 5 [file EMMM-14-e14455-s001.zip › Figure_5-RAW_DATA/5_A/WT_Sham.jpg]

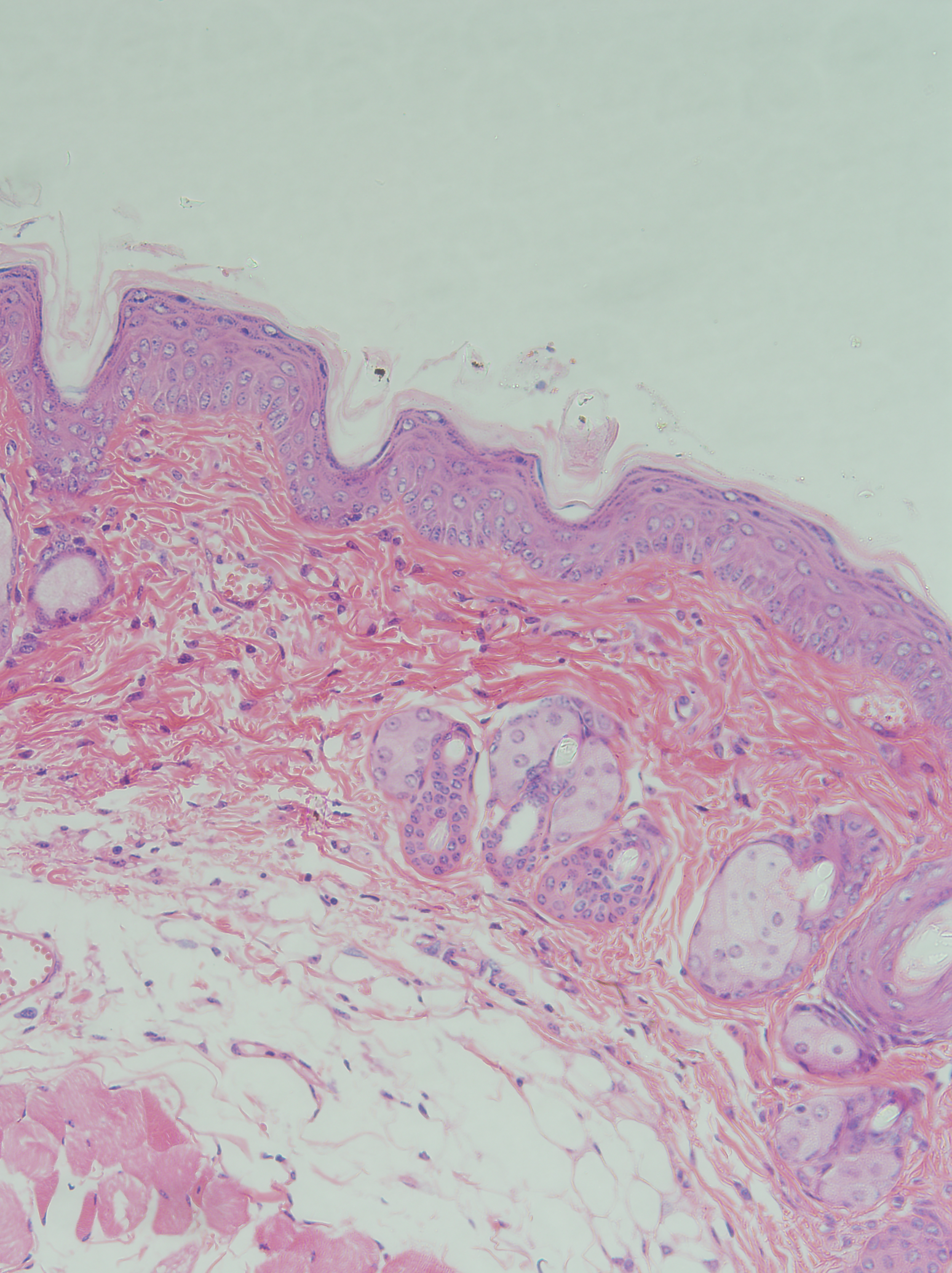

Supplement: Supplementary file 7 — Source Data for Figure 5 [file EMMM-14-e14455-s001.zip › Figure_5-RAW_DATA/5_B/KO_IMQ.tif]

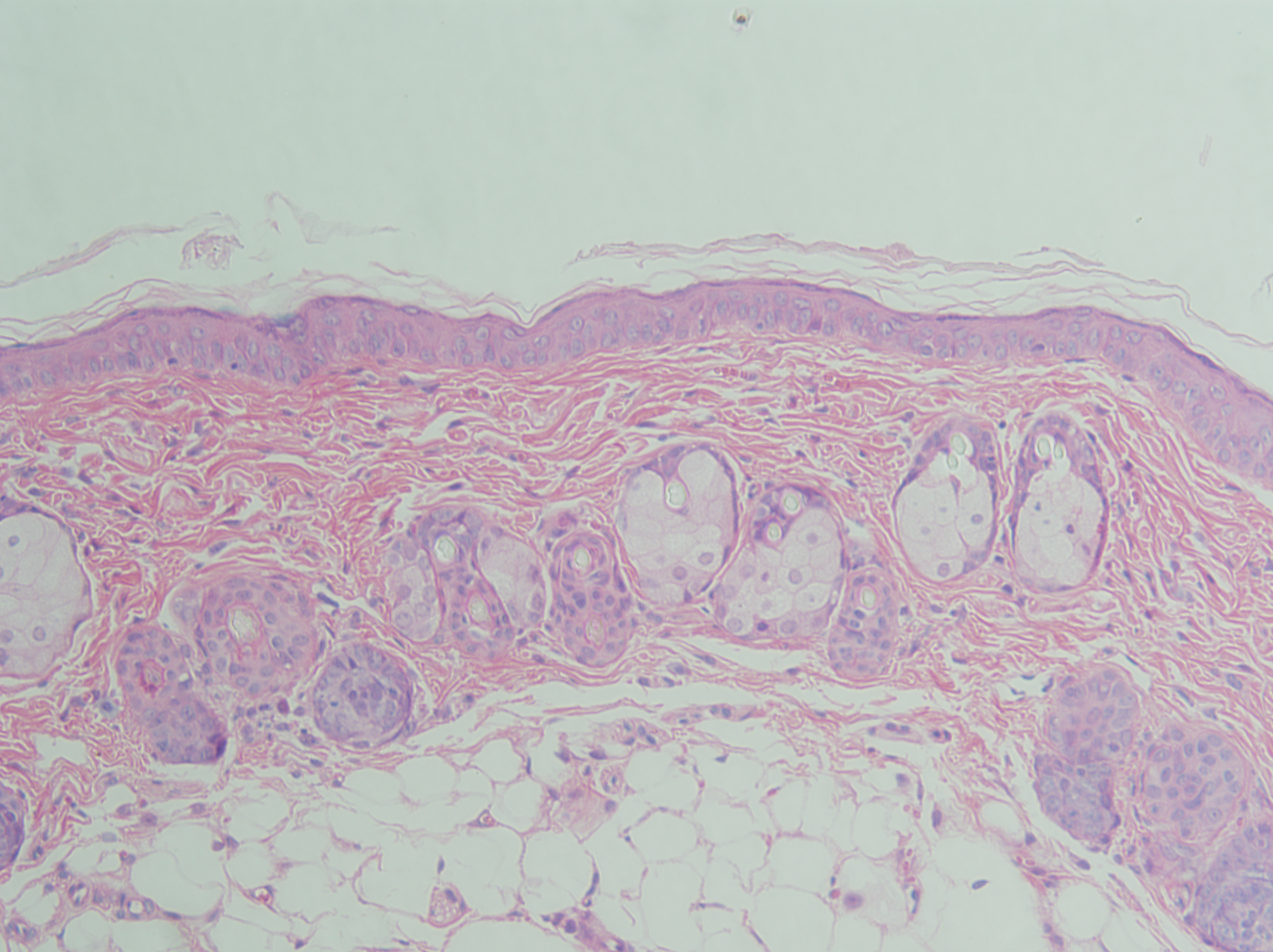

Supplement: Supplementary file 7 — Source Data for Figure 5 [file EMMM-14-e14455-s001.zip › Figure_5-RAW_DATA/5_B/KO_Sham.tif]

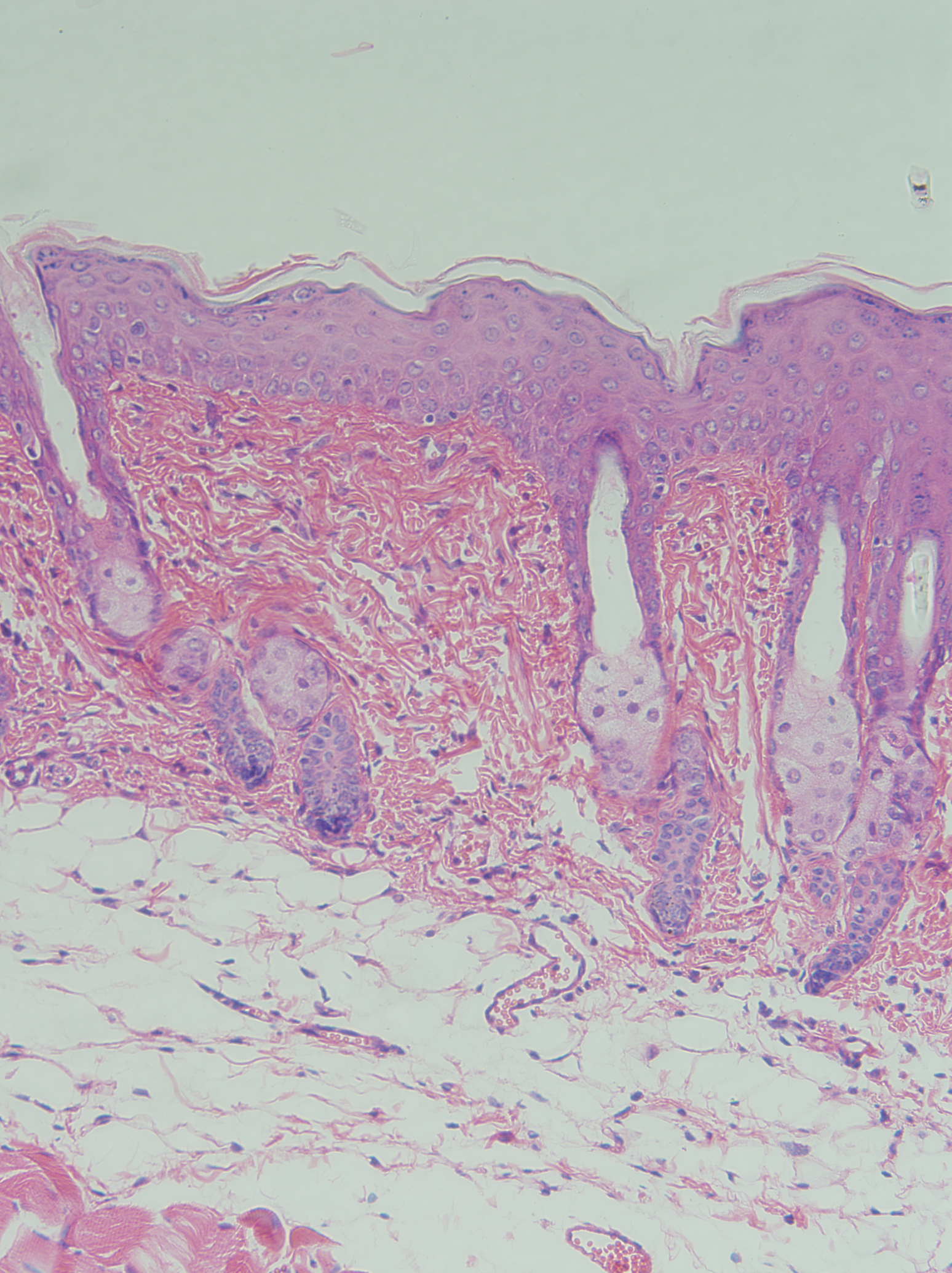

Supplement: Supplementary file 7 — Source Data for Figure 5 [file EMMM-14-e14455-s001.zip › Figure_5-RAW_DATA/5_B/WT_IMQ.tif]

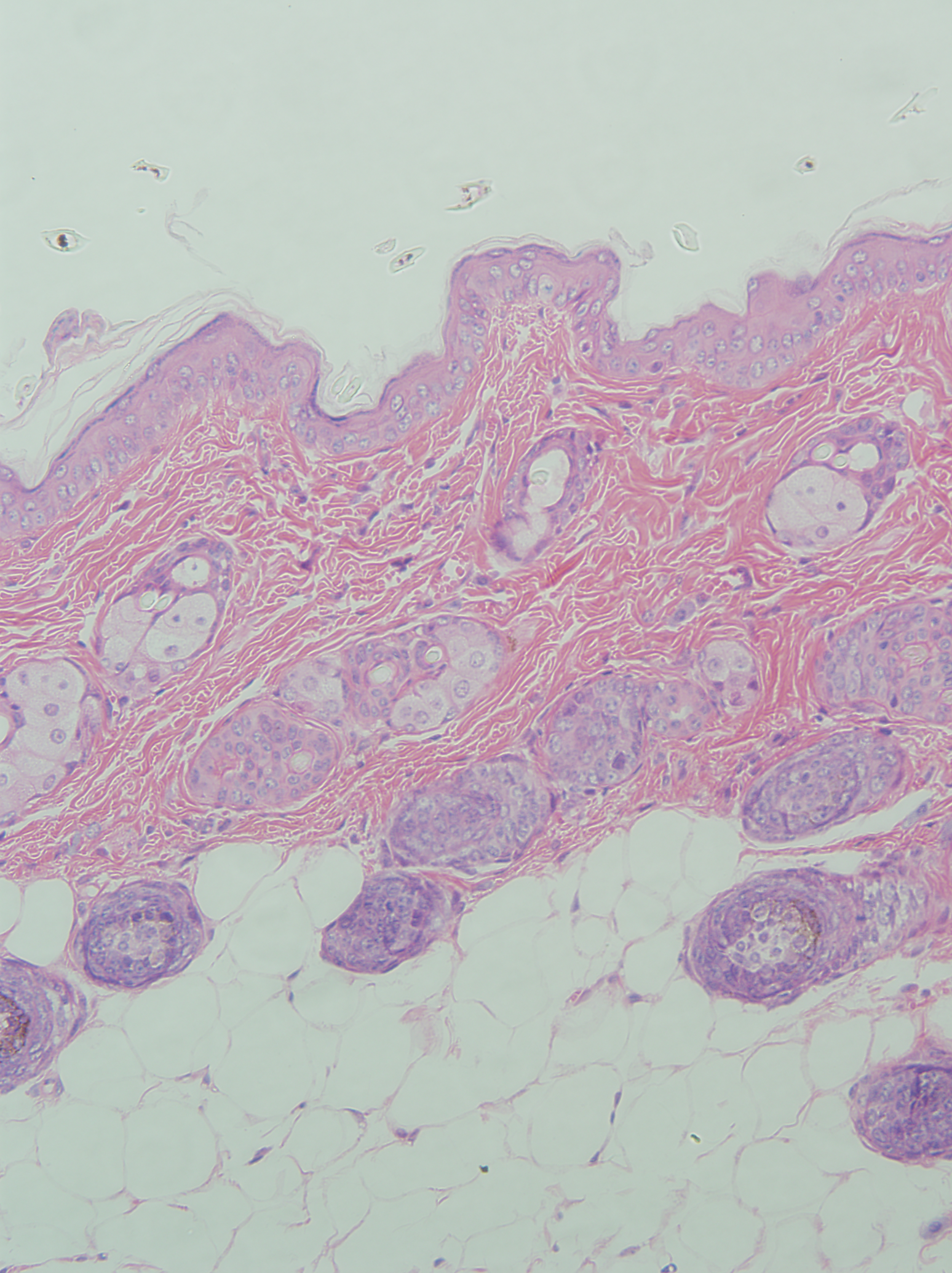

Supplement: Supplementary file 7 — Source Data for Figure 5 [file EMMM-14-e14455-s001.zip › Figure_5-RAW_DATA/5_B/WT_Sham.tif]

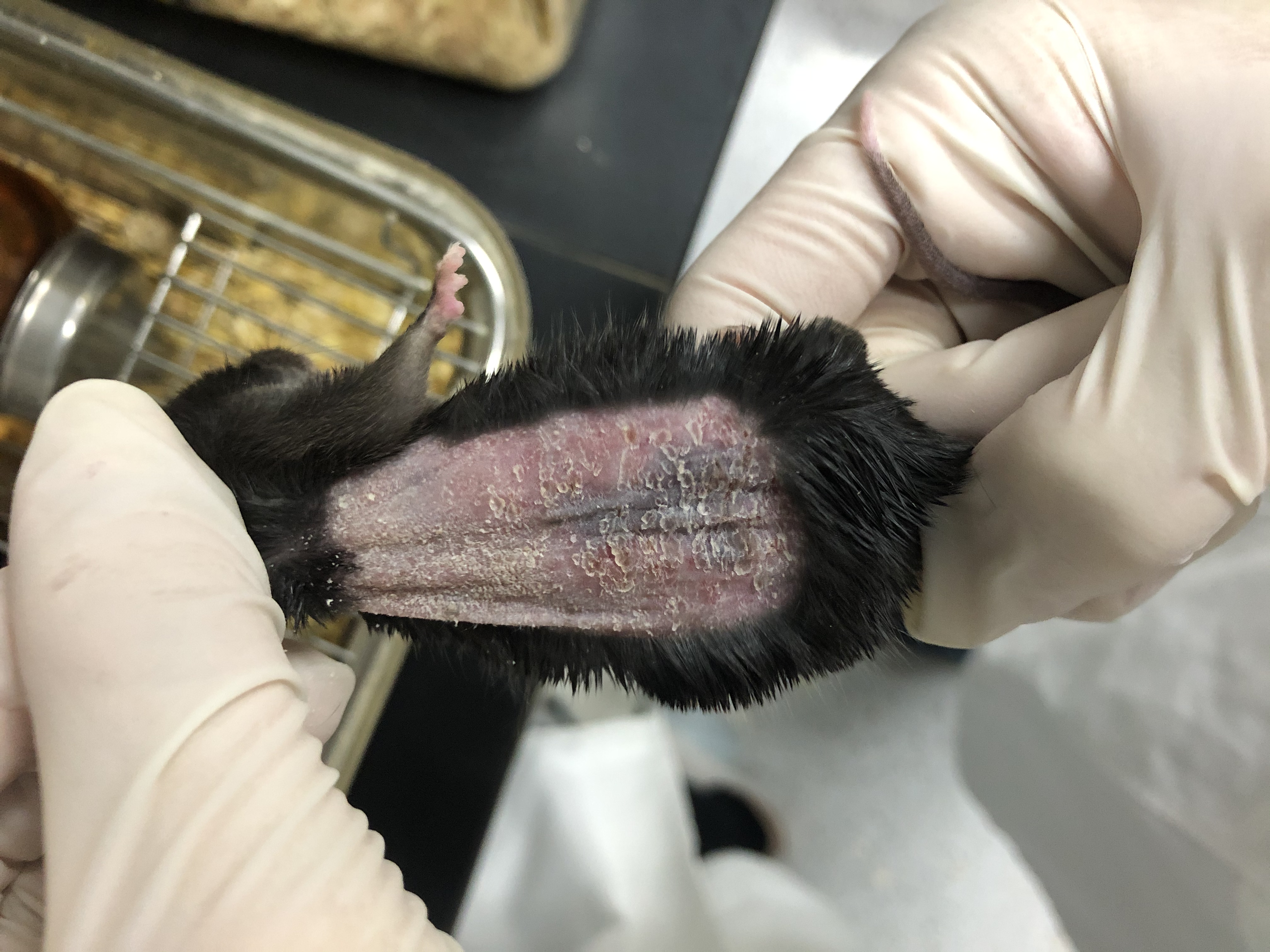

Supplement: Supplementary file 7 — Source Data for Figure 5 [file EMMM-14-e14455-s001.zip › Figure_5-RAW_DATA/5_E/KO_IMQ.jpg]

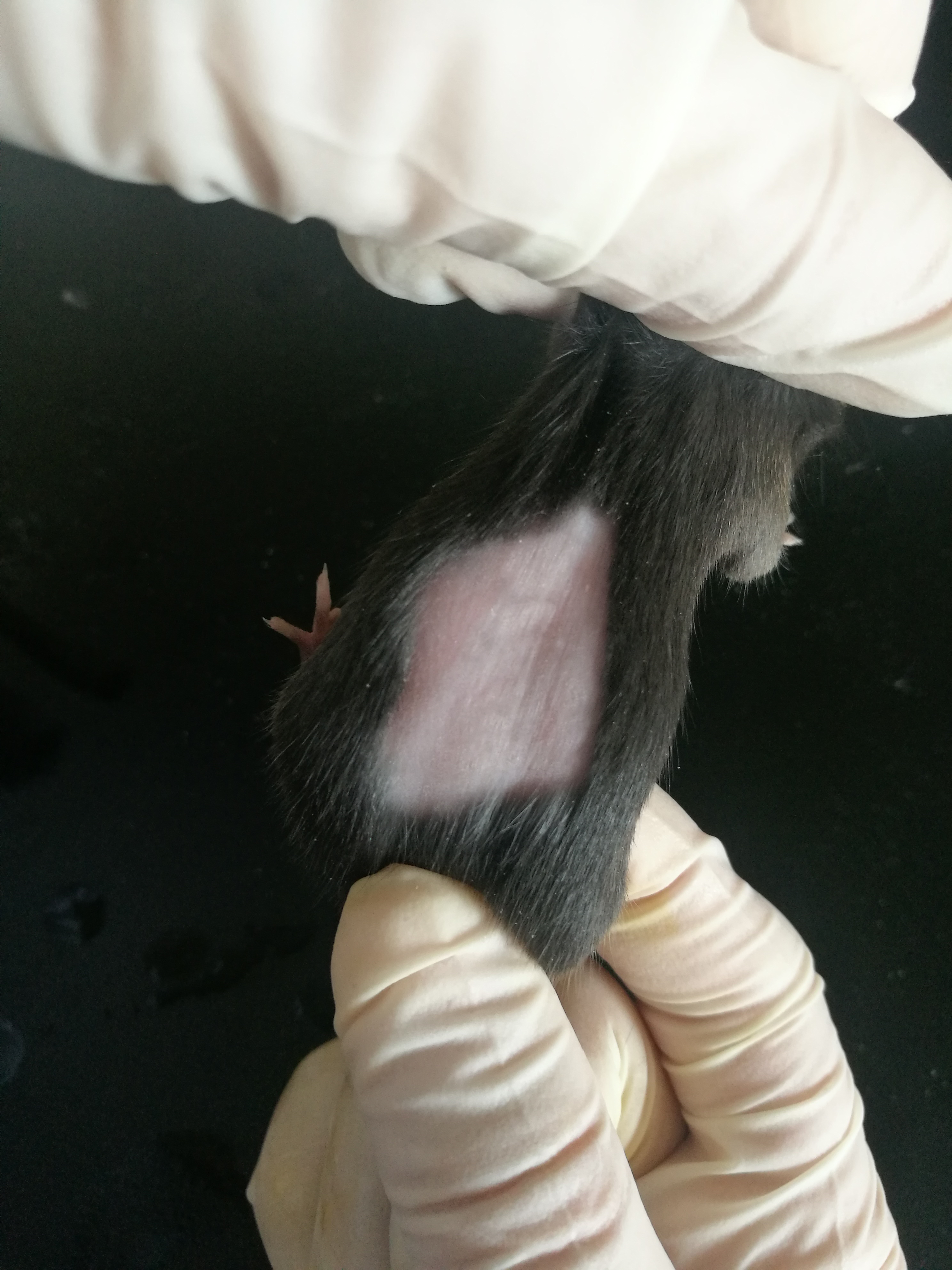

Supplement: Supplementary file 7 — Source Data for Figure 5 [file EMMM-14-e14455-s001.zip › Figure_5-RAW_DATA/5_E/KO_Sham.jpg]

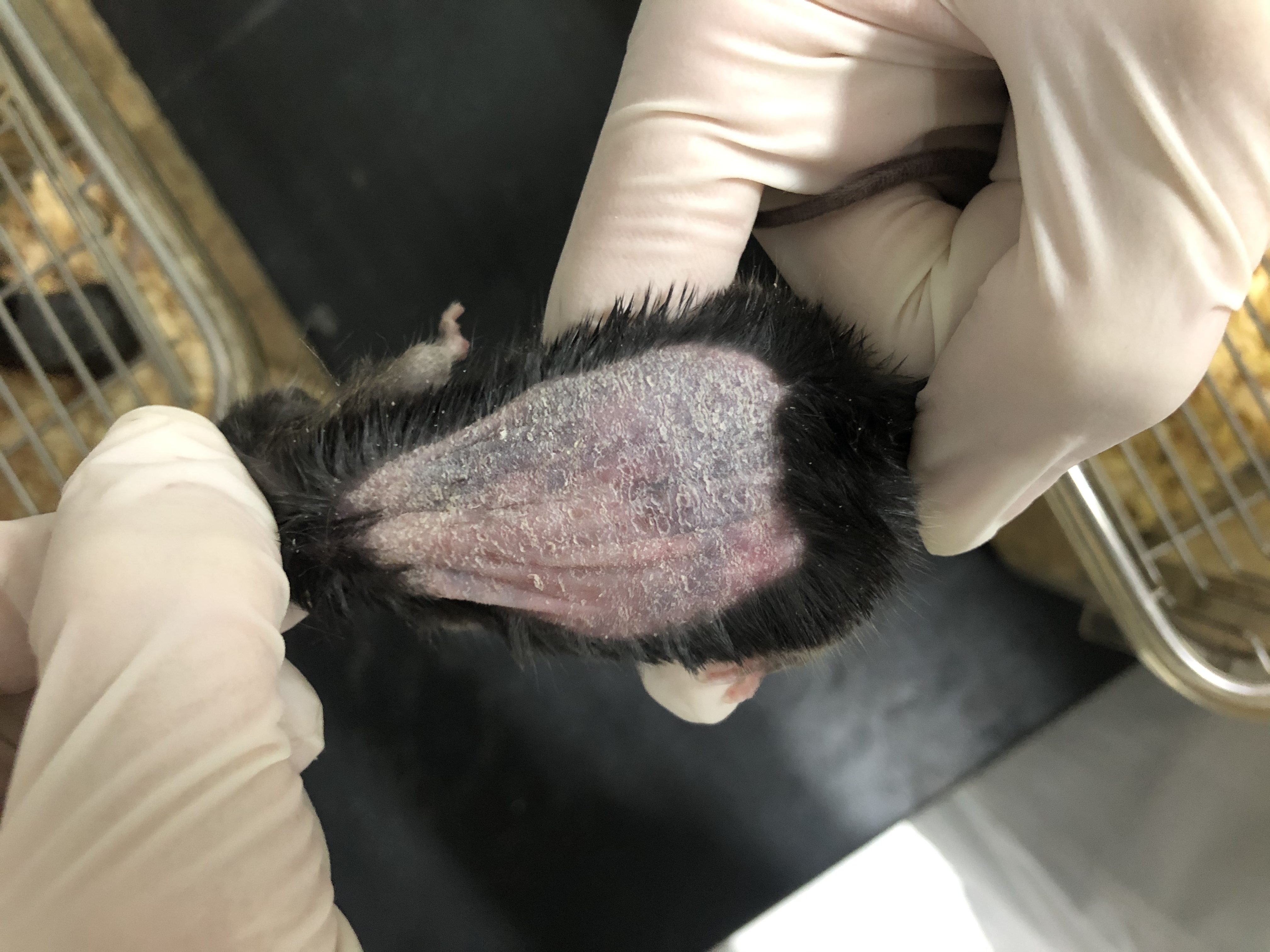

Supplement: Supplementary file 7 — Source Data for Figure 5 [file EMMM-14-e14455-s001.zip › Figure_5-RAW_DATA/5_E/WT_IMQ.jpg]

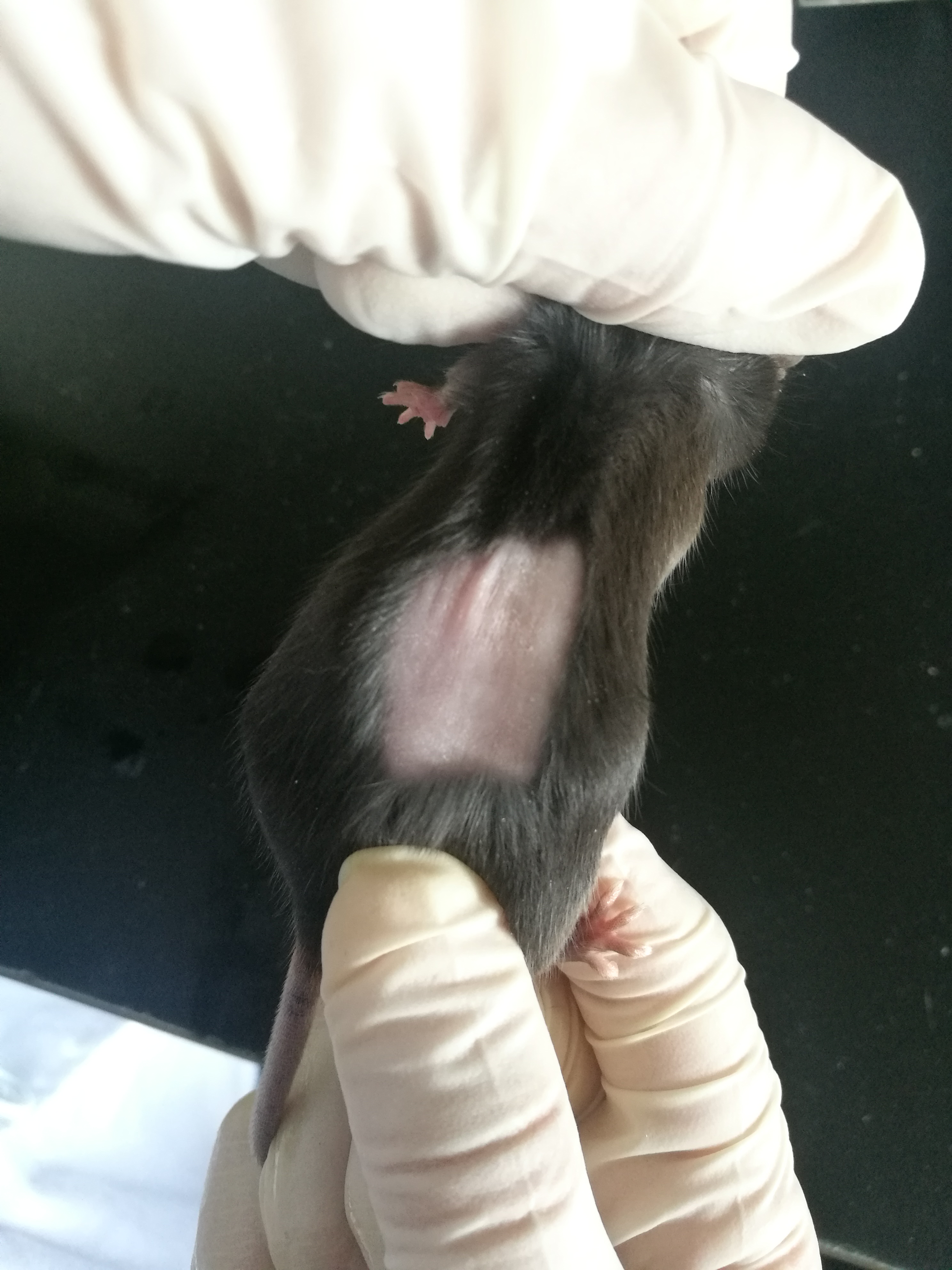

Supplement: Supplementary file 7 — Source Data for Figure 5 [file EMMM-14-e14455-s001.zip › Figure_5-RAW_DATA/5_E/WT_Sham.jpg]

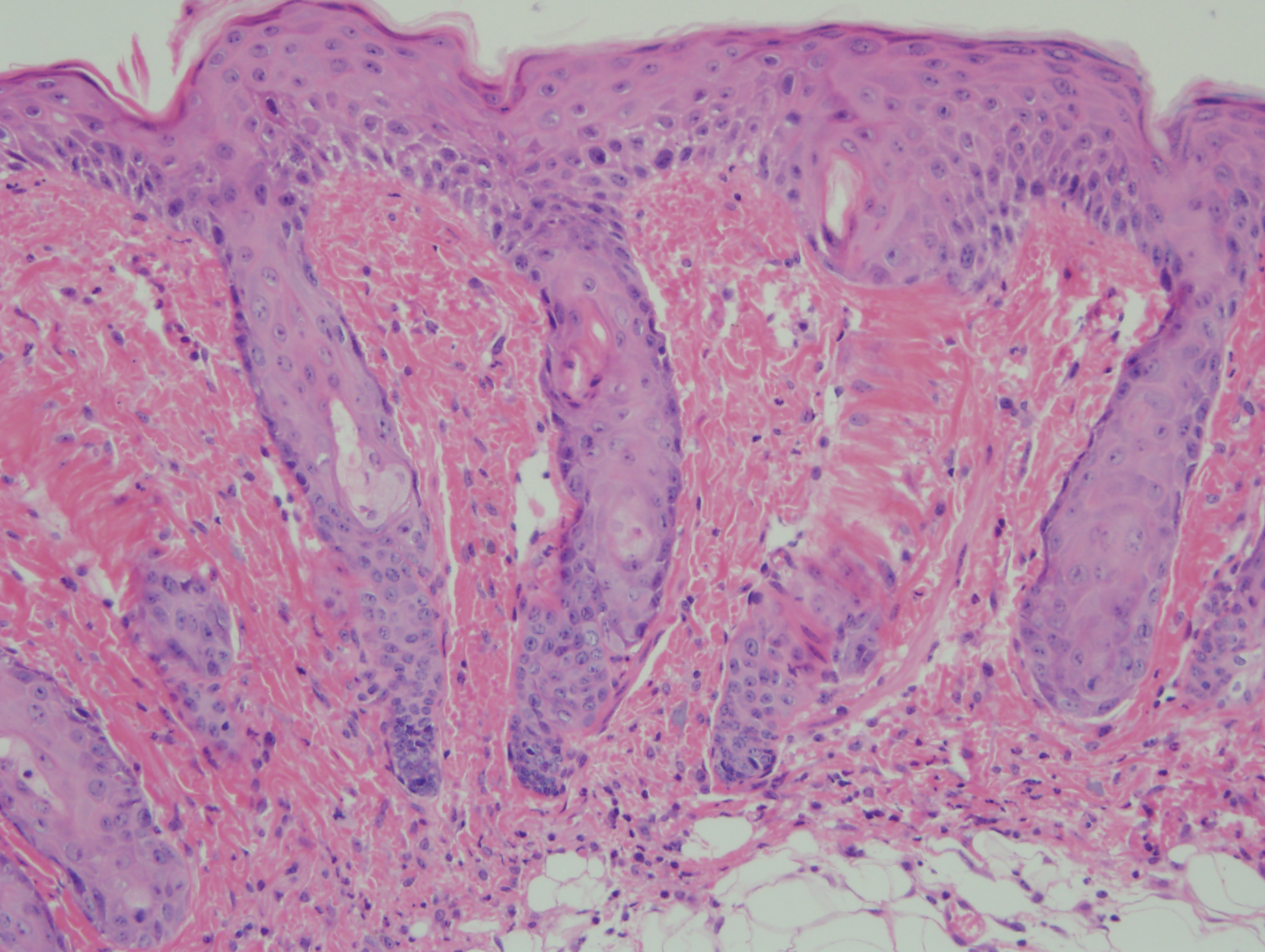

Supplement: Supplementary file 7 — Source Data for Figure 5 [file EMMM-14-e14455-s001.zip › Figure_5-RAW_DATA/5_F/KO_IMQ.tif]

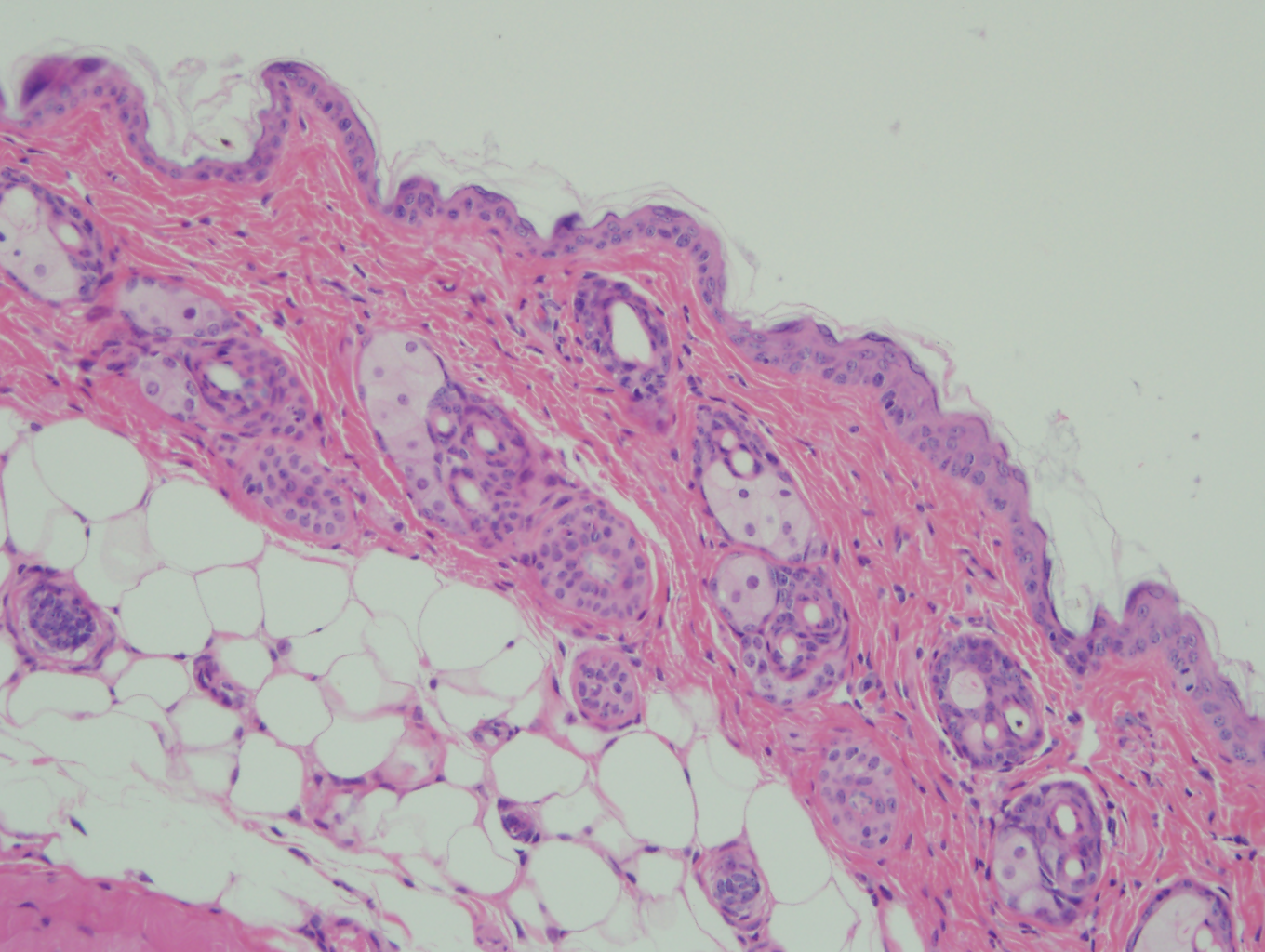

Supplement: Supplementary file 7 — Source Data for Figure 5 [file EMMM-14-e14455-s001.zip › Figure_5-RAW_DATA/5_F/KO_Sham.tif]

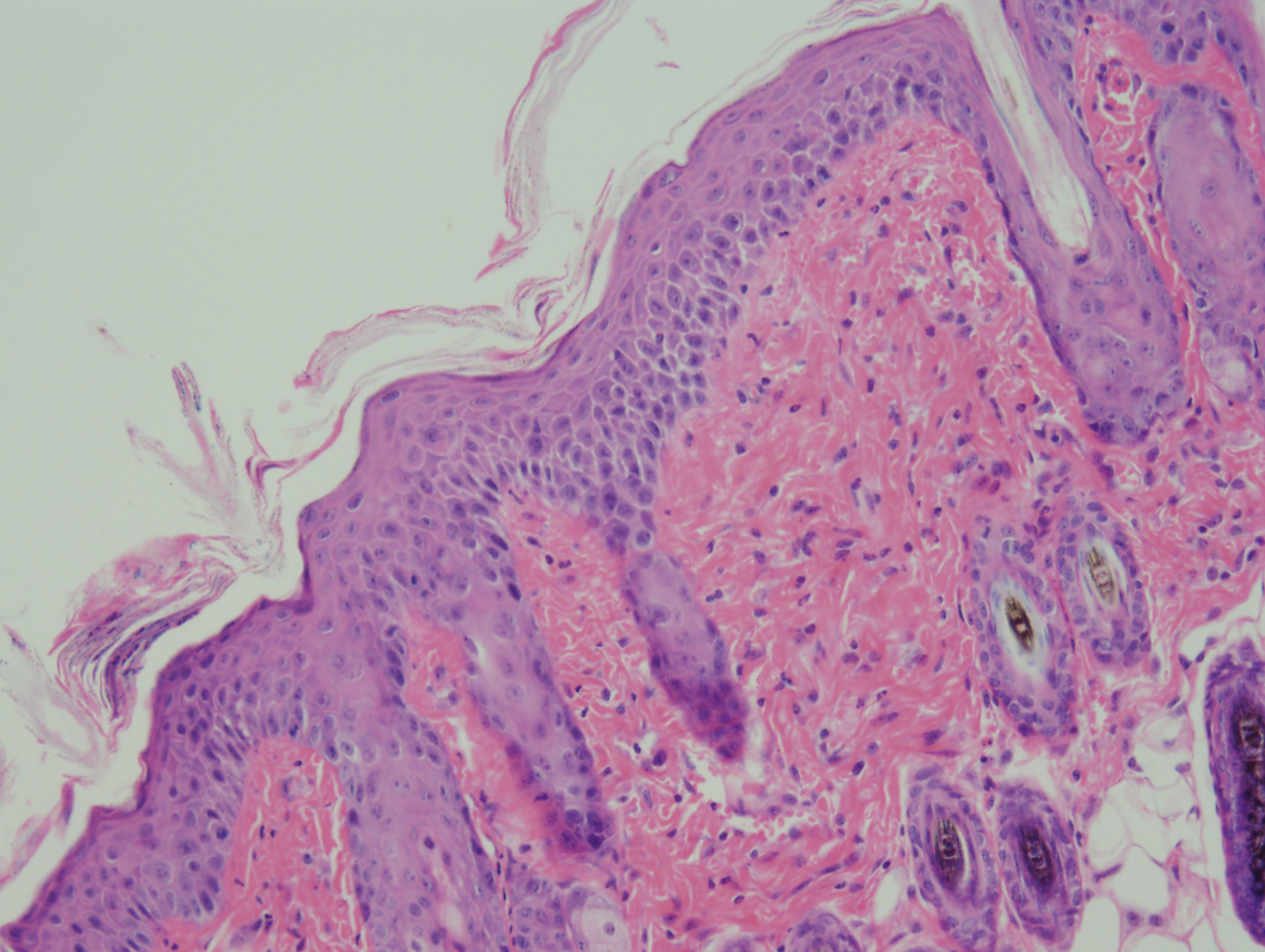

Supplement: Supplementary file 7 — Source Data for Figure 5 [file EMMM-14-e14455-s001.zip › Figure_5-RAW_DATA/5_F/WT_IMQ.tif]

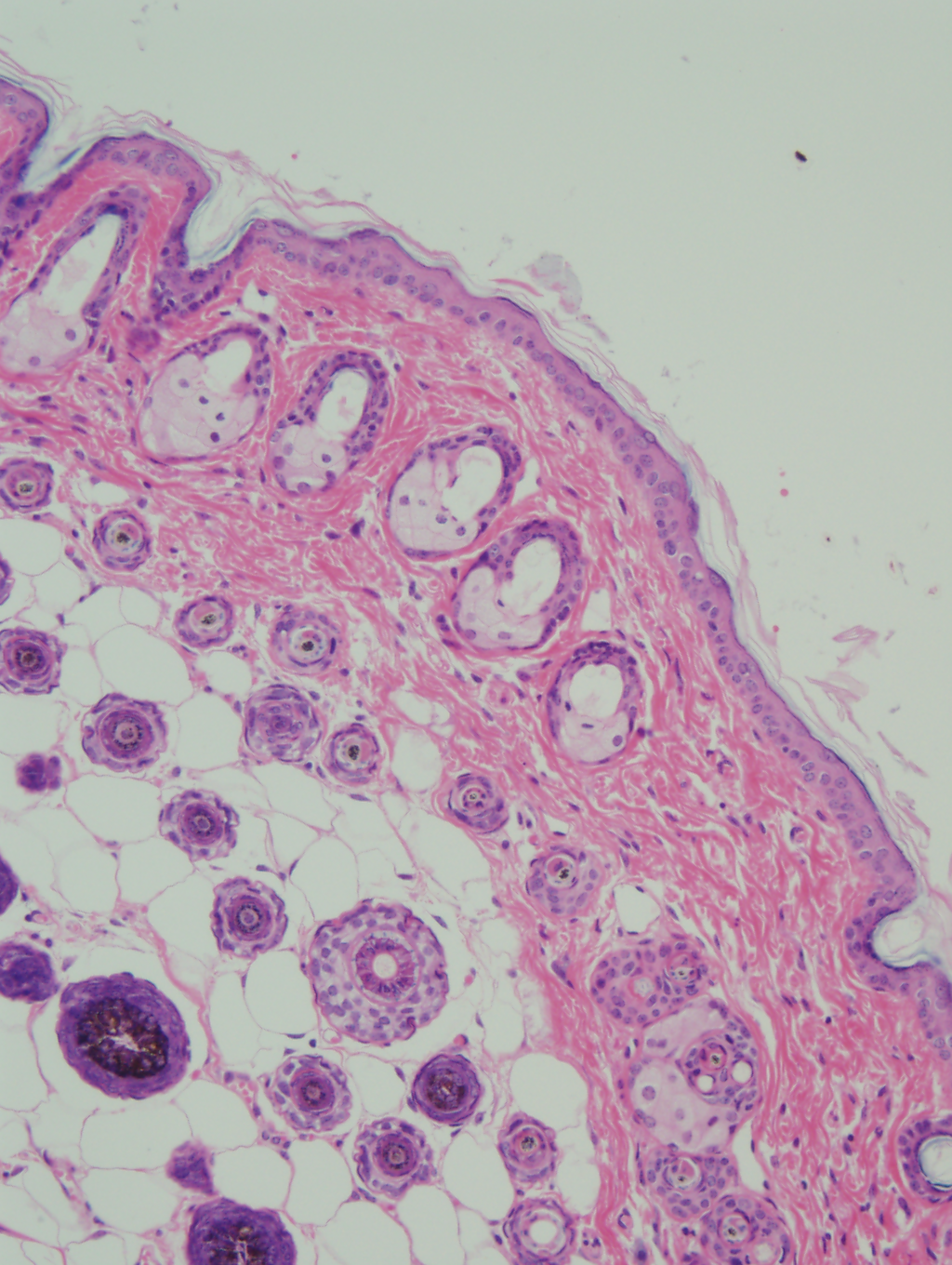

Supplement: Supplementary file 7 — Source Data for Figure 5 [file EMMM-14-e14455-s001.zip › Figure_5-RAW_DATA/5_F/WT_Sham.tif]

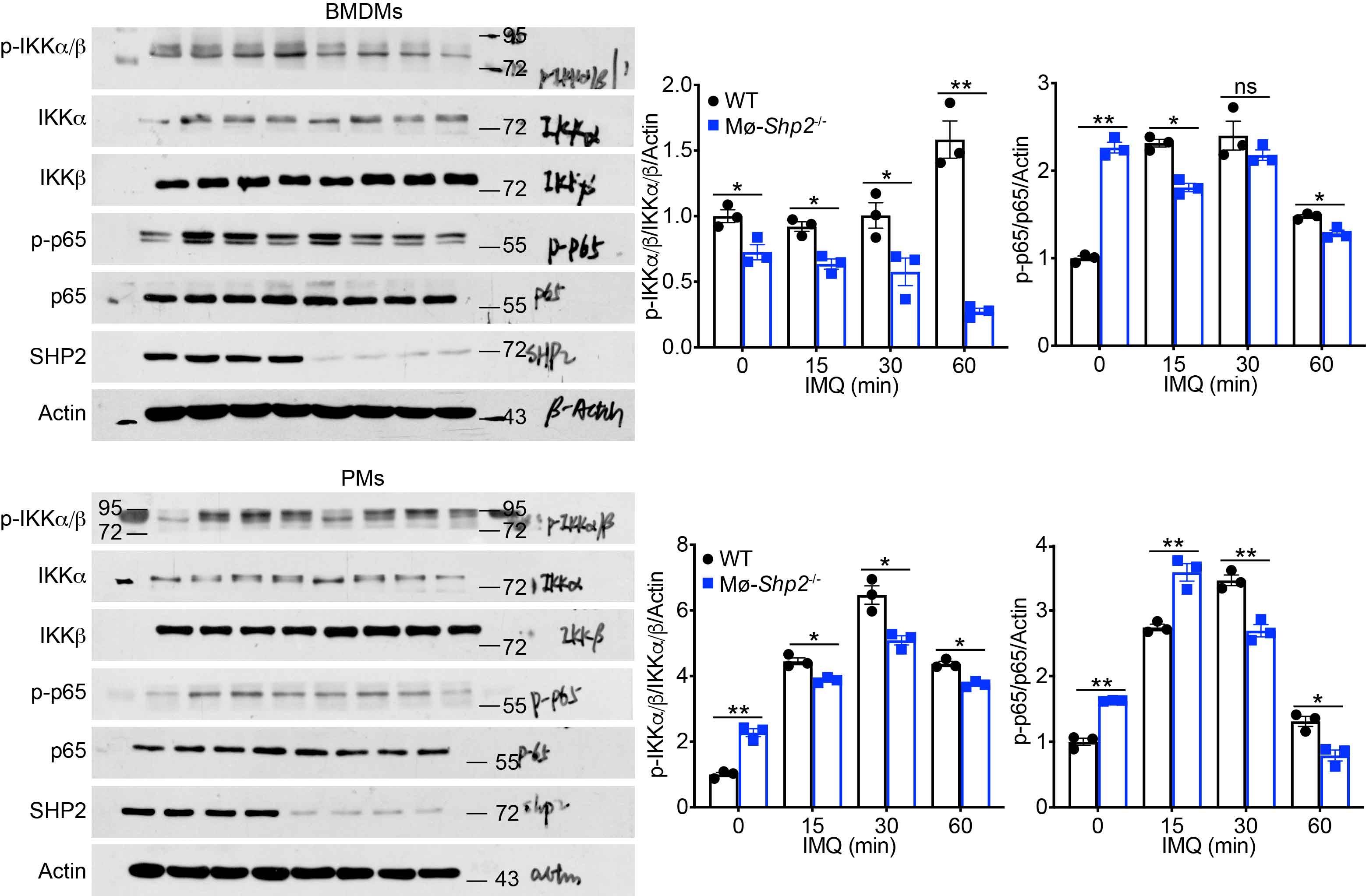

Supplement: Supplementary file 8 — Source Data for Figure 6 [file EMMM-14-e14455-s010.zip › Figure 6/6 D/6 D.jpg]

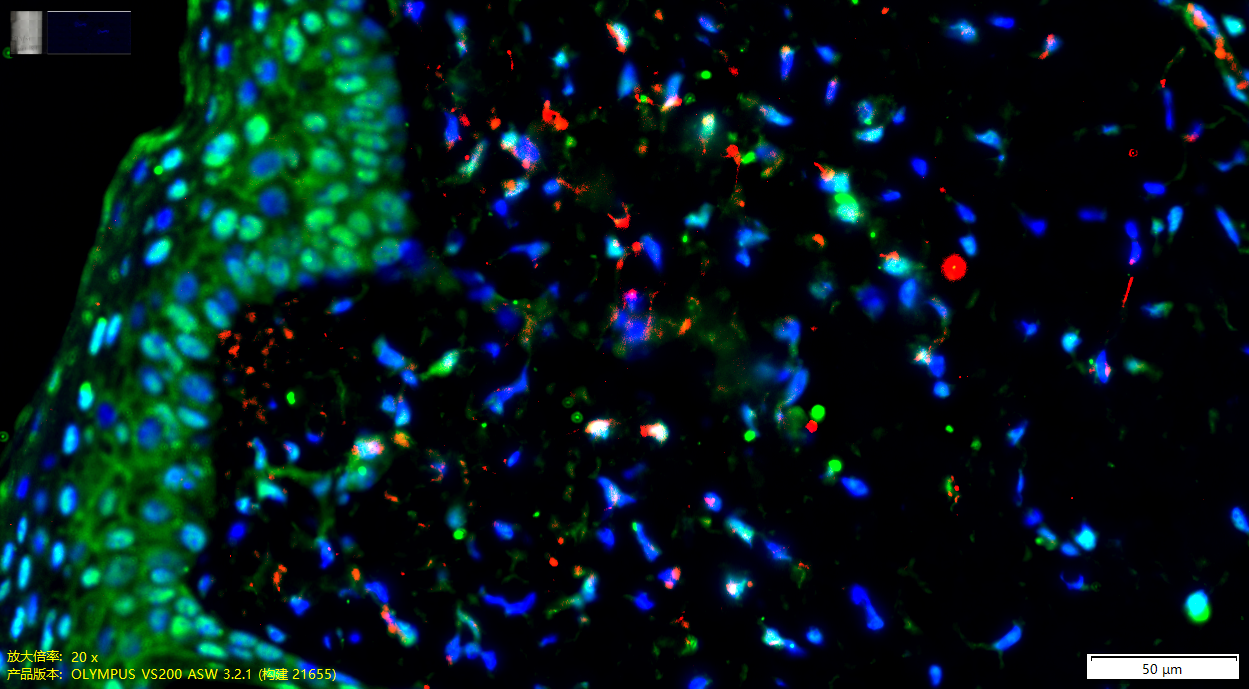

Supplement: Supplementary file 8 — Source Data for Figure 6 [file EMMM-14-e14455-s010.zip › Figure 6/6 E/IMQ-MERGE.tif]

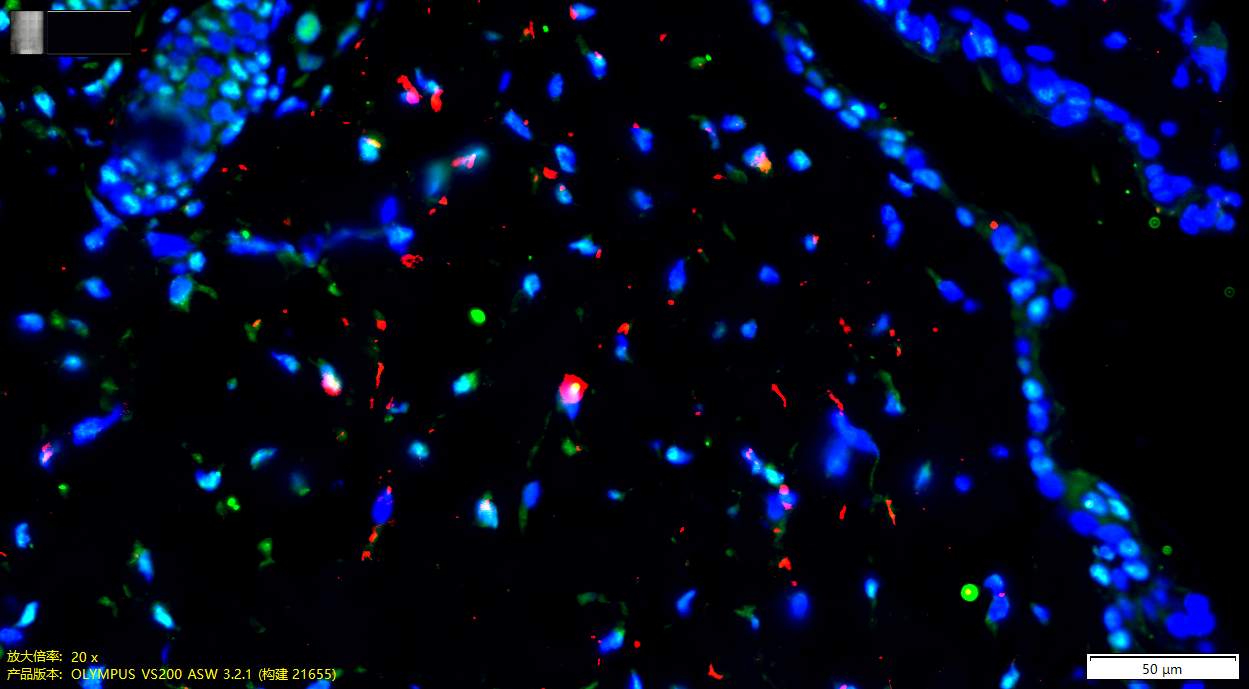

Supplement: Supplementary file 8 — Source Data for Figure 6 [file EMMM-14-e14455-s010.zip › Figure 6/6 E/Sham-MERGE.tif]

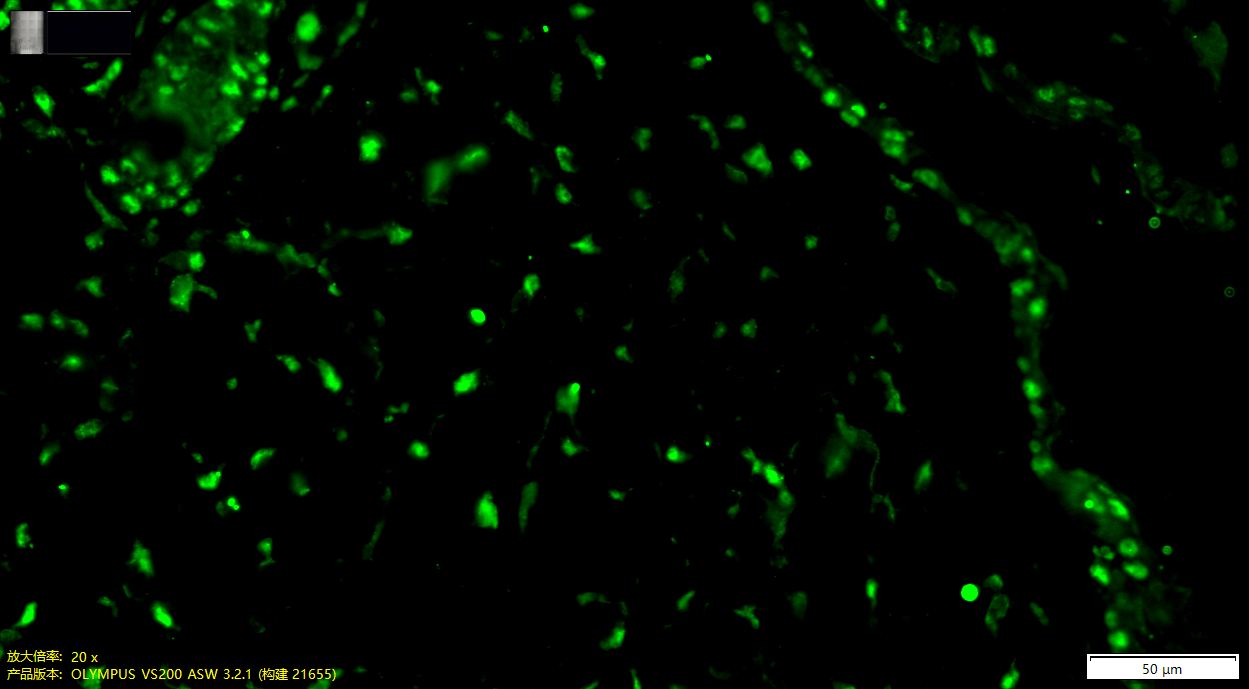

Supplement: Supplementary file 8 — Source Data for Figure 6 [file EMMM-14-e14455-s010.zip › Figure 6/6 E/Sham-P-P65.tif]

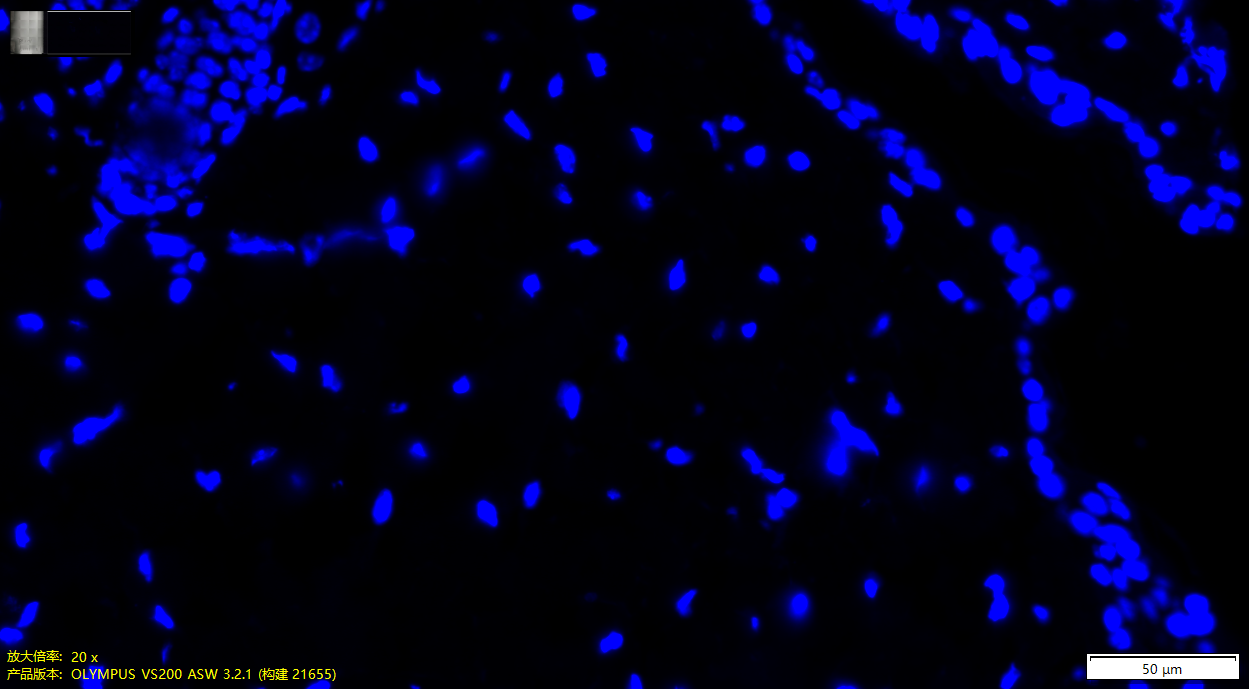

Supplement: Supplementary file 8 — Source Data for Figure 6 [file EMMM-14-e14455-s010.zip › Figure 6/6 E/Sham-DAPI.tif]

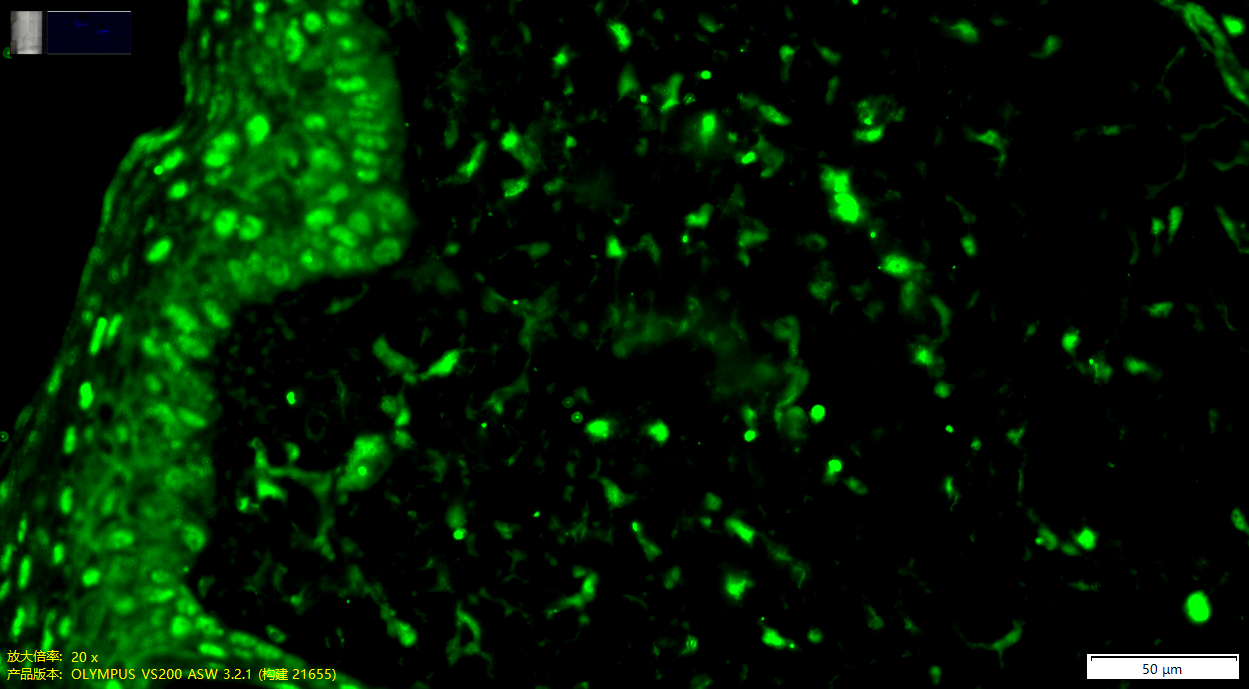

Supplement: Supplementary file 8 — Source Data for Figure 6 [file EMMM-14-e14455-s010.zip › Figure 6/6 E/IMQ-P-P65.tif]

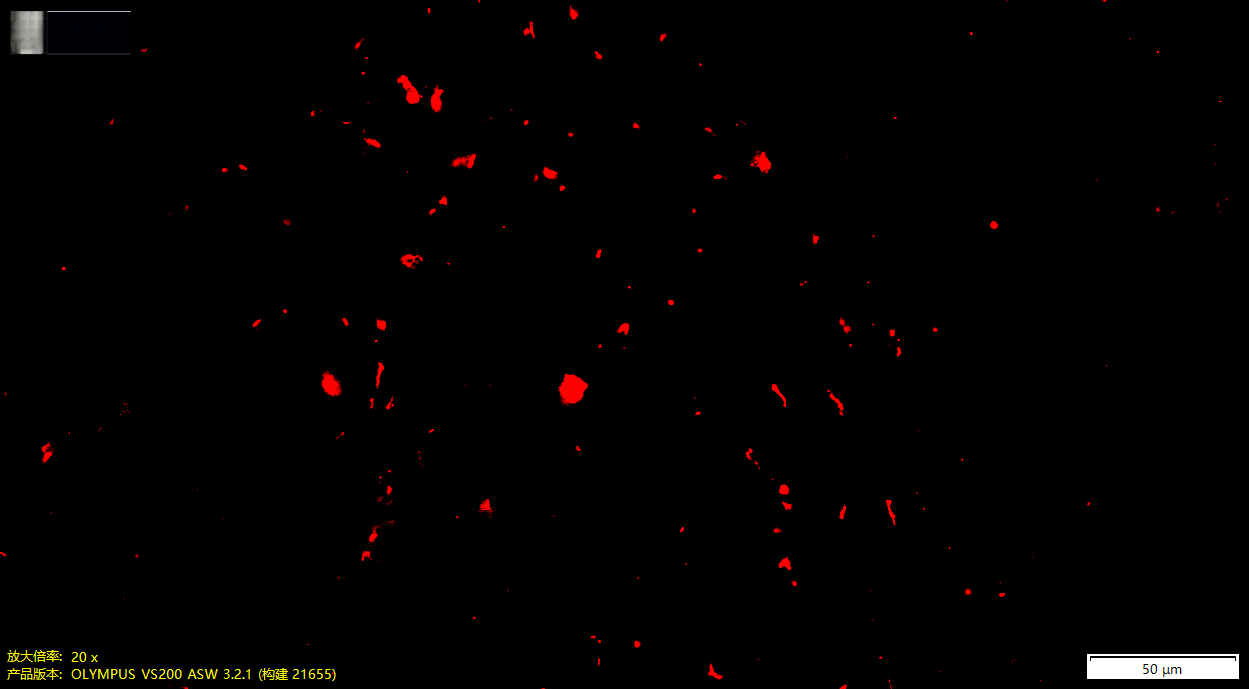

Supplement: Supplementary file 8 — Source Data for Figure 6 [file EMMM-14-e14455-s010.zip › Figure 6/6 E/Sham-F4:80.tif]

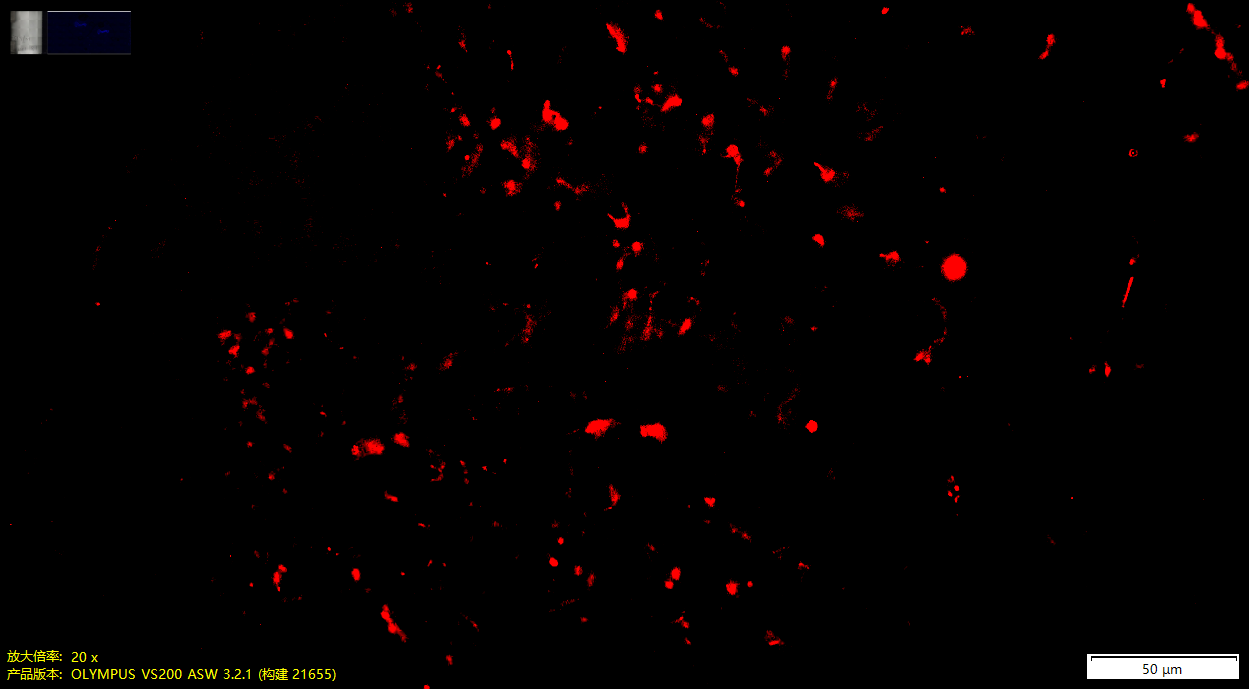

Supplement: Supplementary file 8 — Source Data for Figure 6 [file EMMM-14-e14455-s010.zip › Figure 6/6 E/IMQ-F4:80.tif]

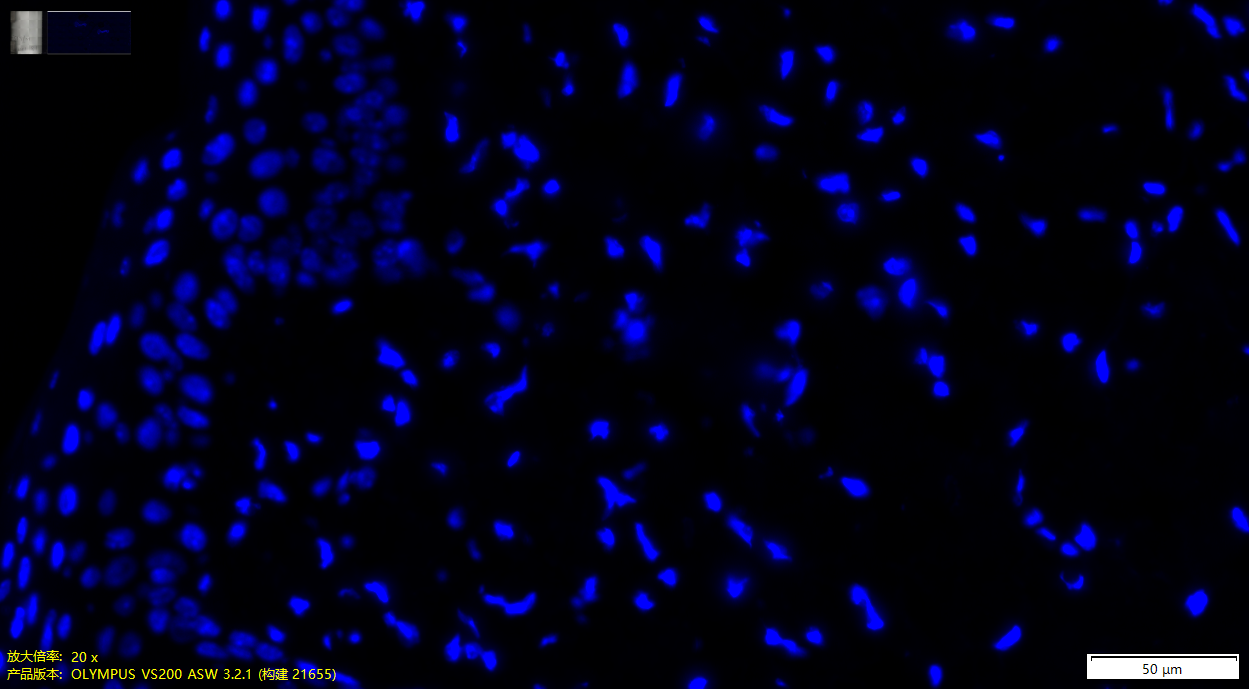

Supplement: Supplementary file 8 — Source Data for Figure 6 [file EMMM-14-e14455-s010.zip › Figure 6/6 E/IMQ-DAPI.tif]

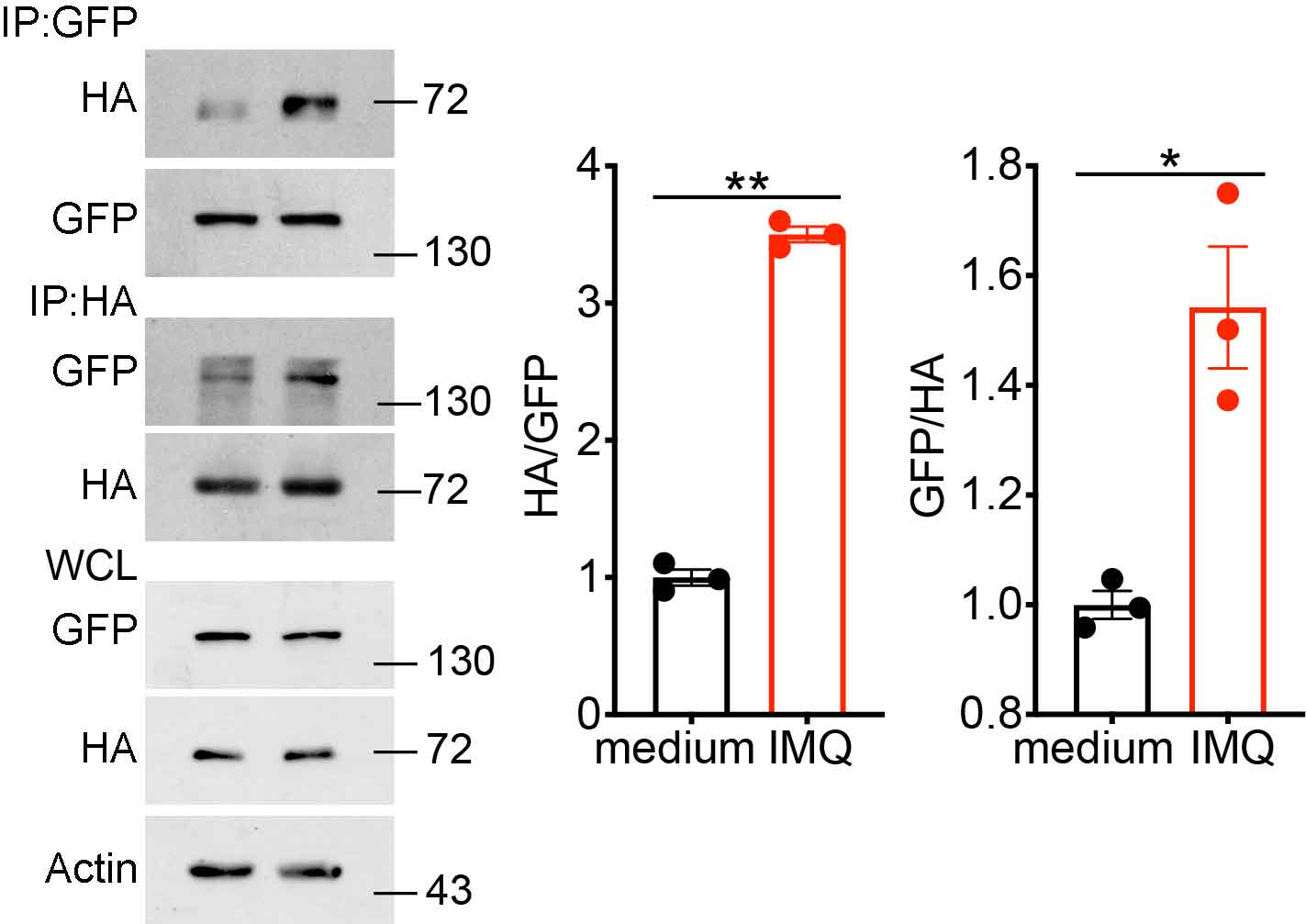

Supplement: Supplementary file 9 — Source Data for Figure 7 [file EMMM-14-e14455-s009.zip › Figure_7/7_A/7_A.jpg]

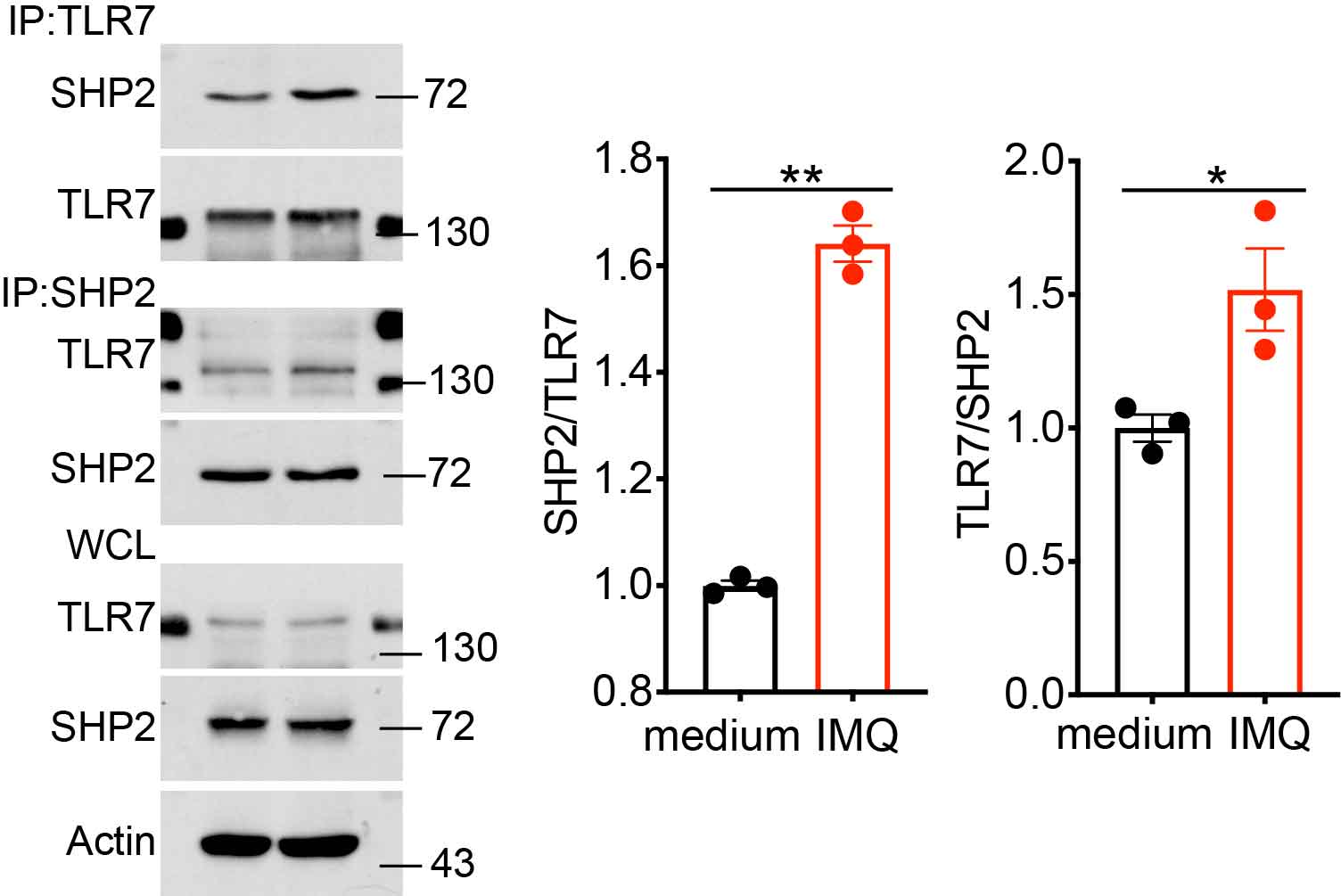

Supplement: Supplementary file 9 — Source Data for Figure 7 [file EMMM-14-e14455-s009.zip › Figure_7/7_B/7_B.jpg]

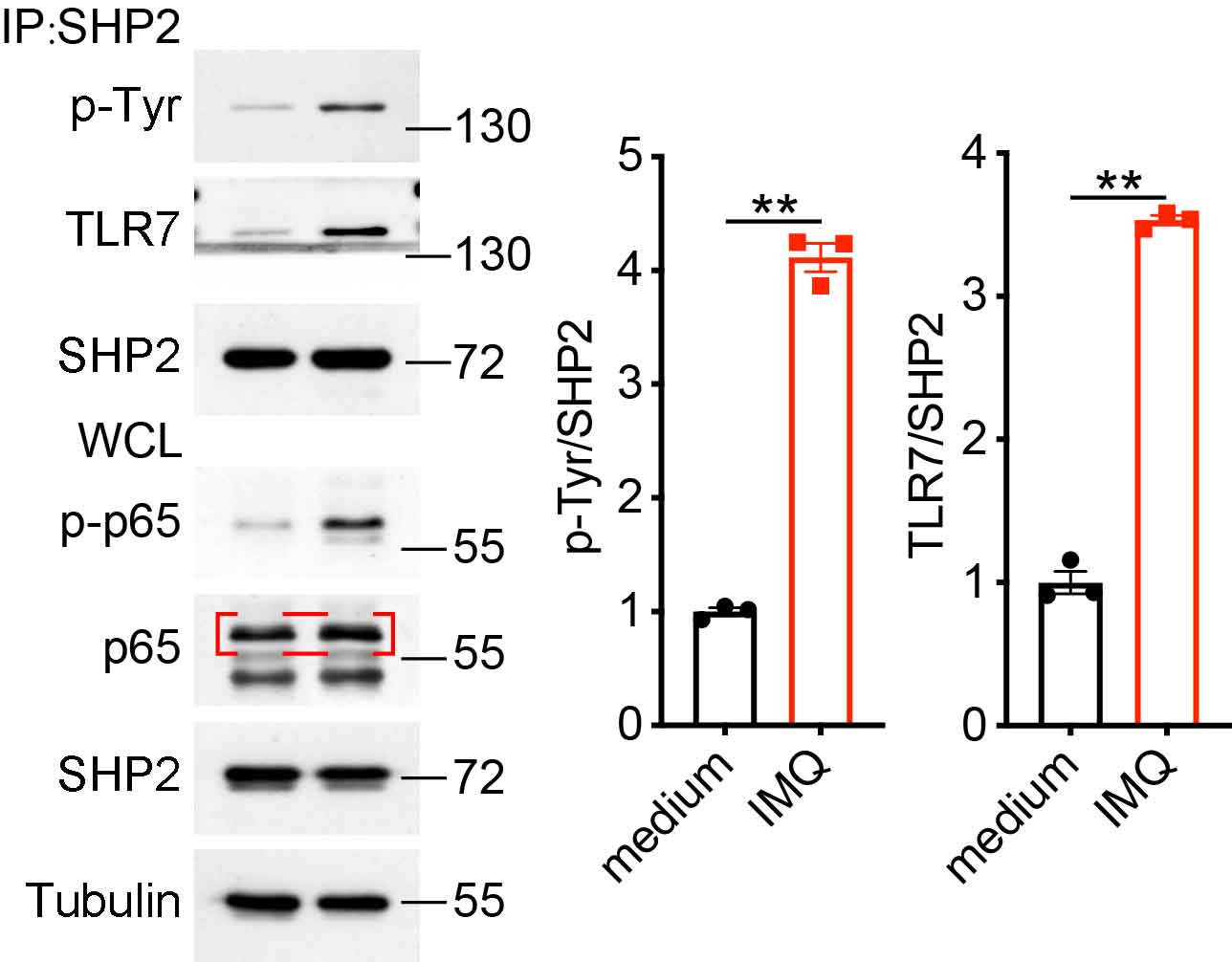

Supplement: Supplementary file 9 — Source Data for Figure 7 [file EMMM-14-e14455-s009.zip › Figure_7/7_C/7_C.jpg]

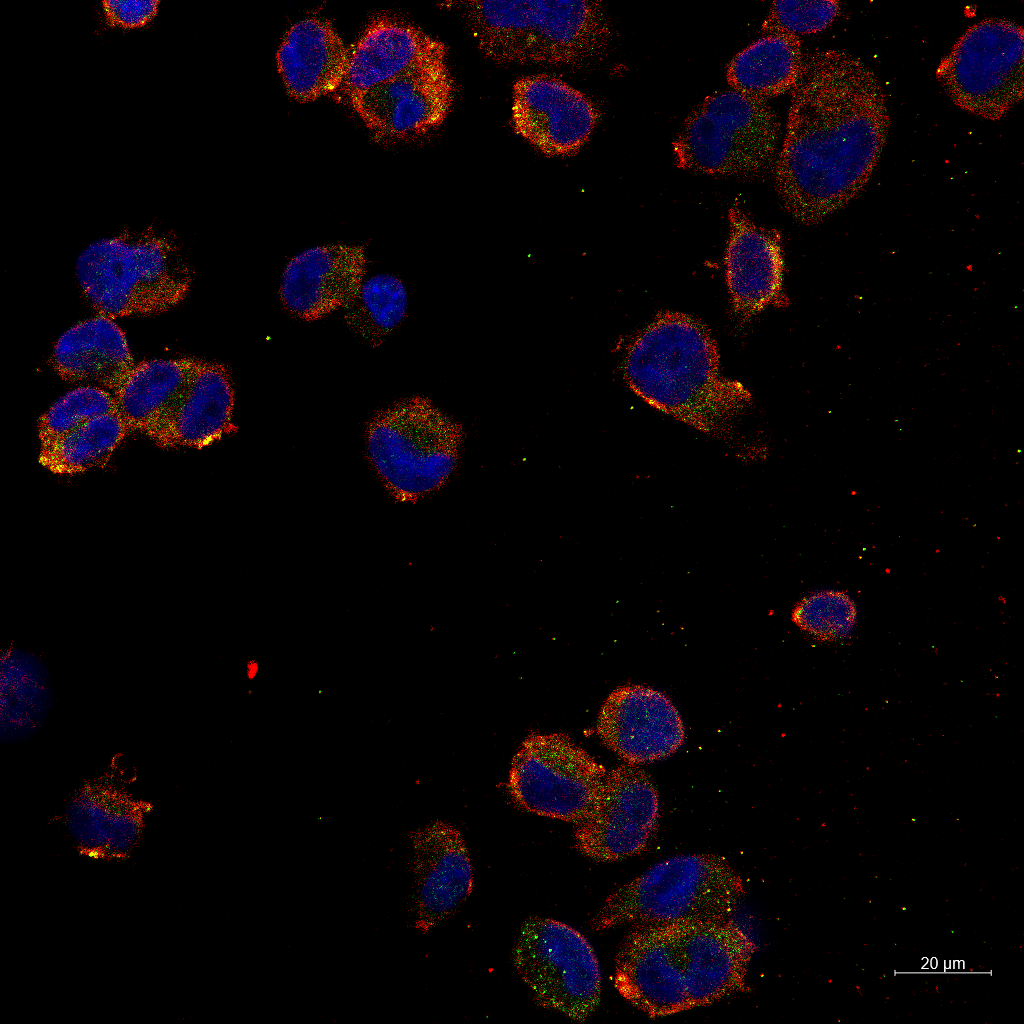

Supplement: Supplementary file 9 — Source Data for Figure 7 [file EMMM-14-e14455-s009.zip › Figure_7/7_D/0_min/0.tif]

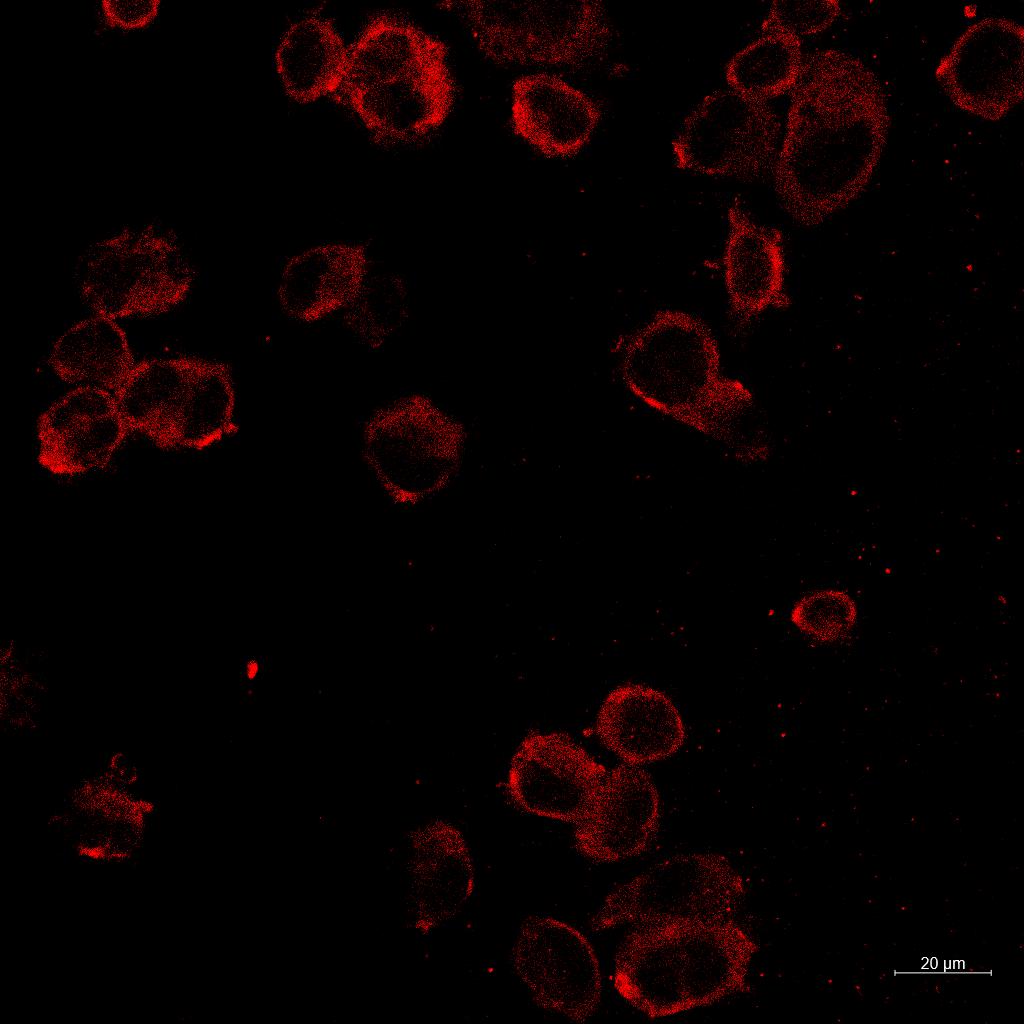

Supplement: Supplementary file 9 — Source Data for Figure 7 [file EMMM-14-e14455-s009.zip › Figure_7/7_D/0_min/Image_38_c1.tif]

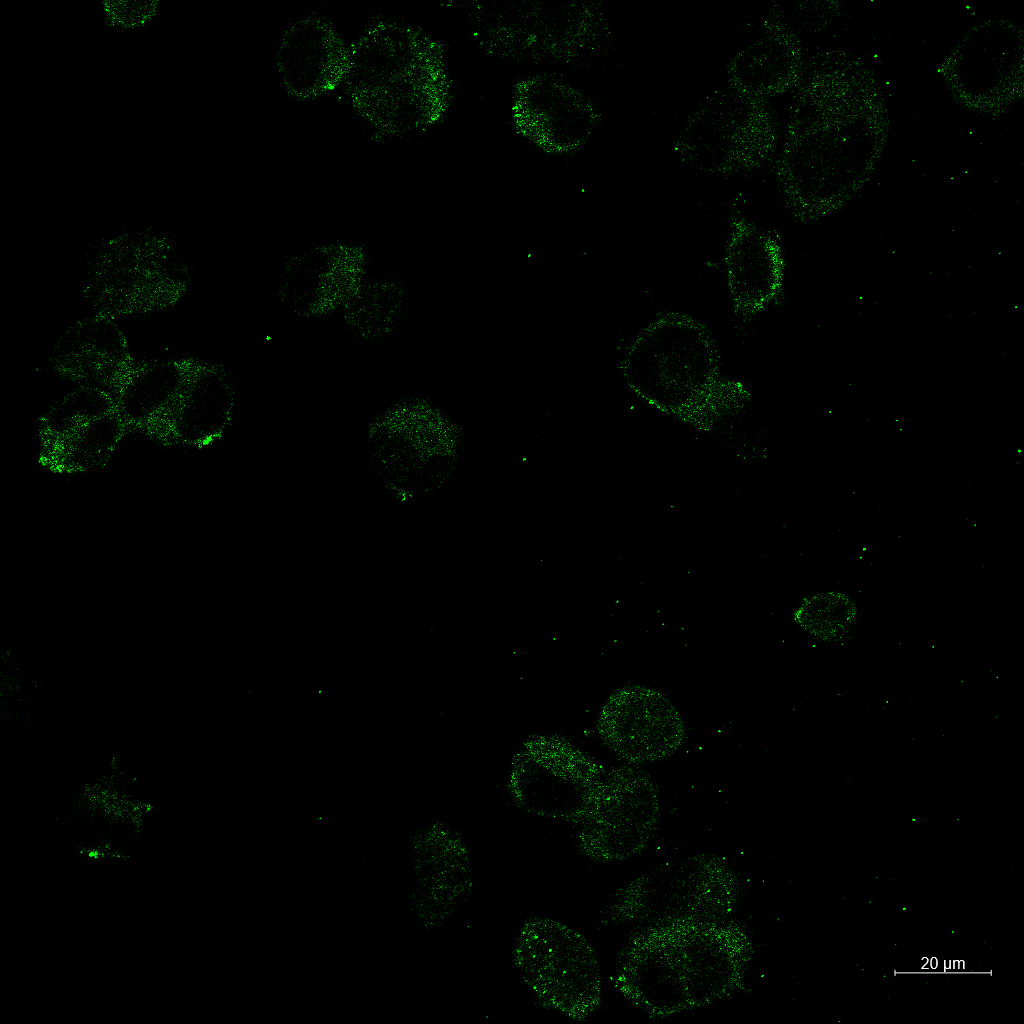

Supplement: Supplementary file 9 — Source Data for Figure 7 [file EMMM-14-e14455-s009.zip › Figure_7/7_D/0_min/Image_38_c3_2.tif]

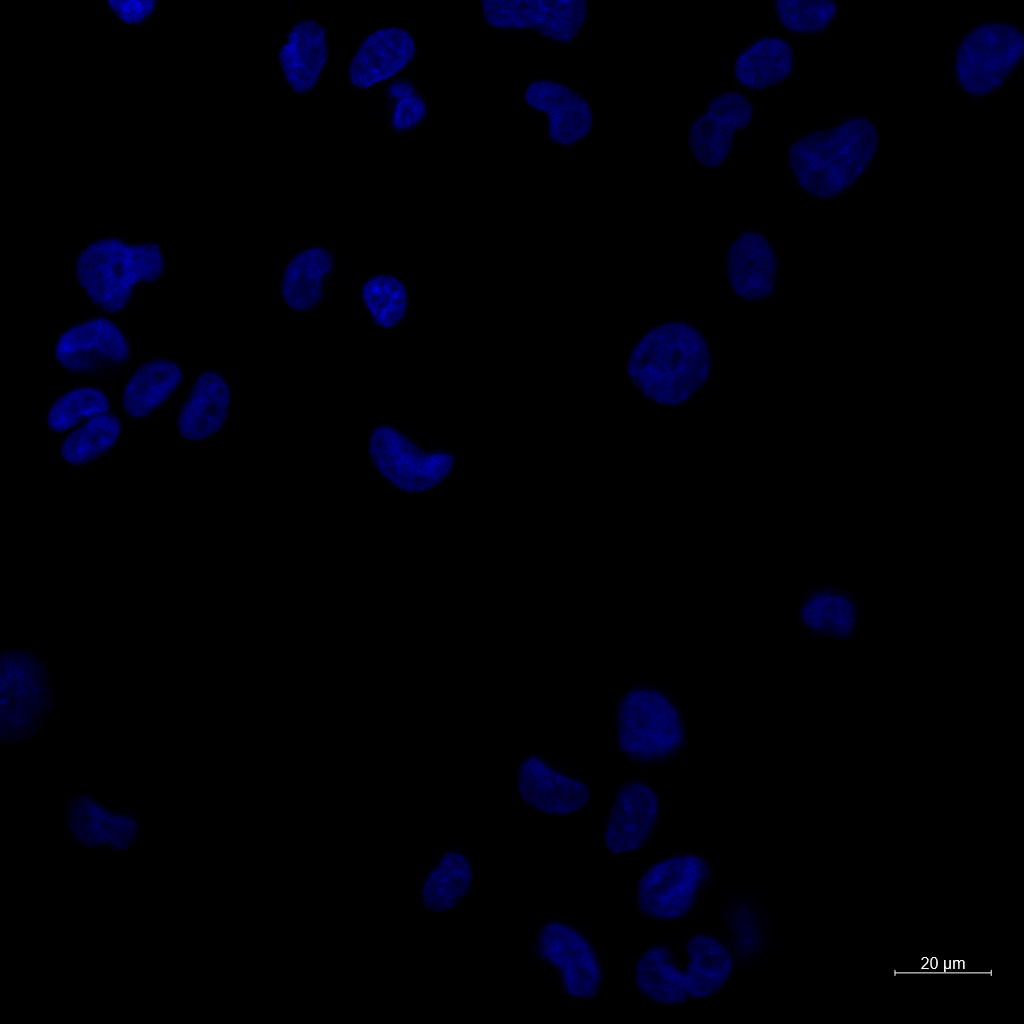

Supplement: Supplementary file 9 — Source Data for Figure 7 [file EMMM-14-e14455-s009.zip › Figure_7/7_D/0_min/Image_38_c4.tif]

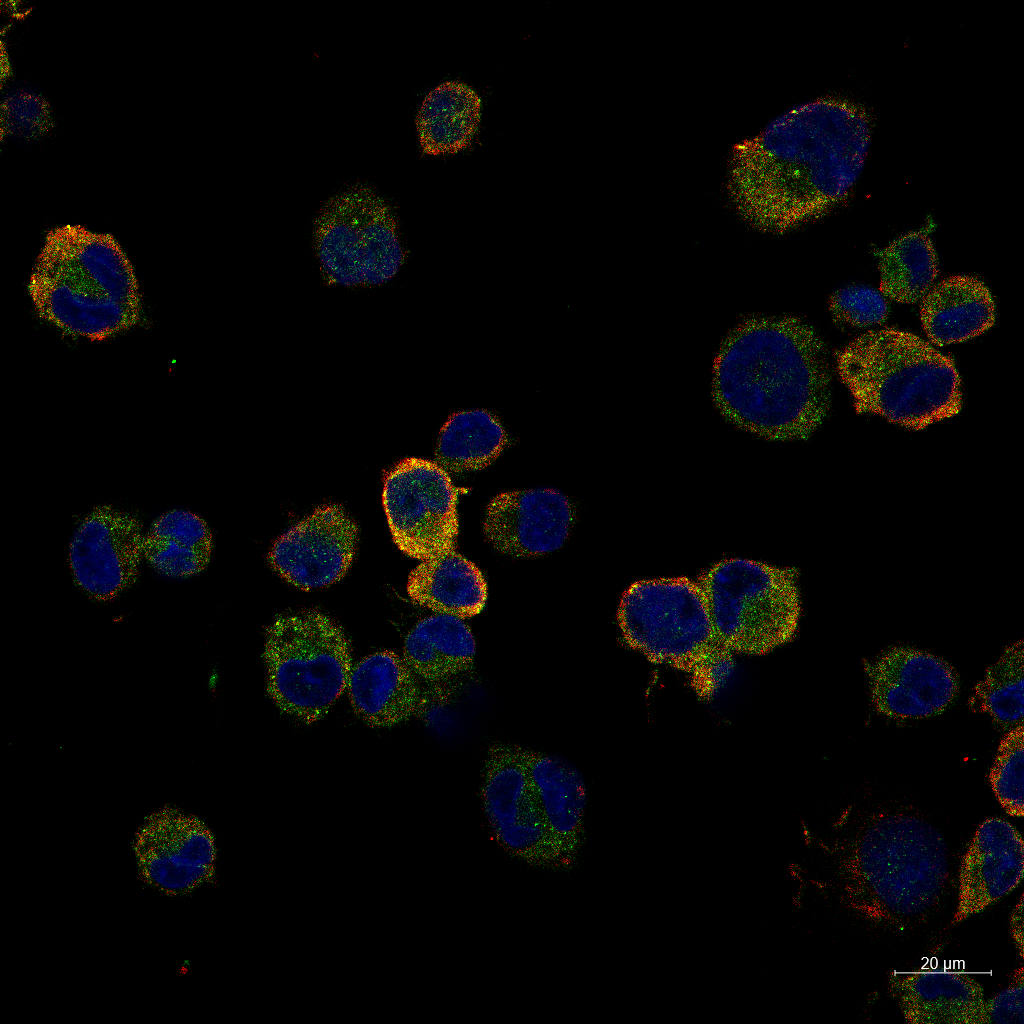

Supplement: Supplementary file 9 — Source Data for Figure 7 [file EMMM-14-e14455-s009.zip › Figure_7/7_D/15_min/15.tif]

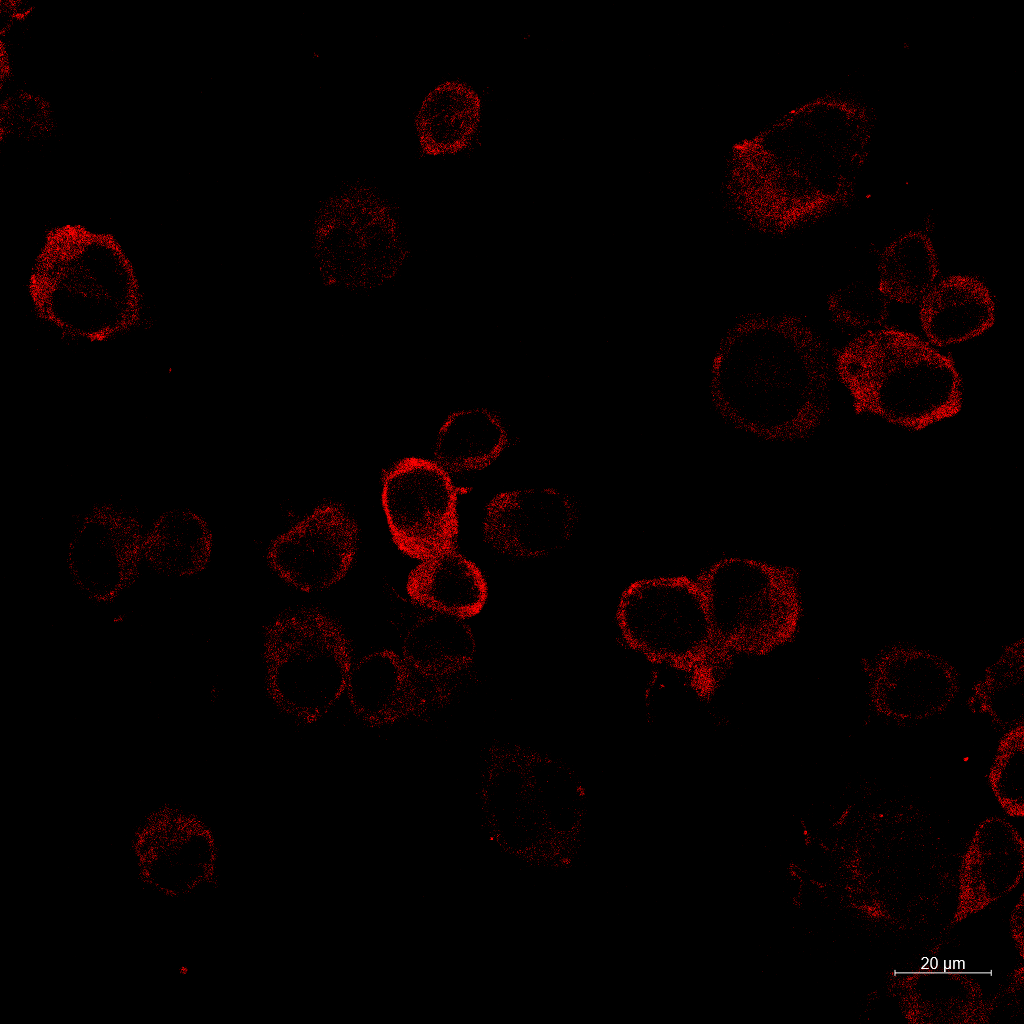

Supplement: Supplementary file 9 — Source Data for Figure 7 [file EMMM-14-e14455-s009.zip › Figure_7/7_D/15_min/Image_53_c1.tif]

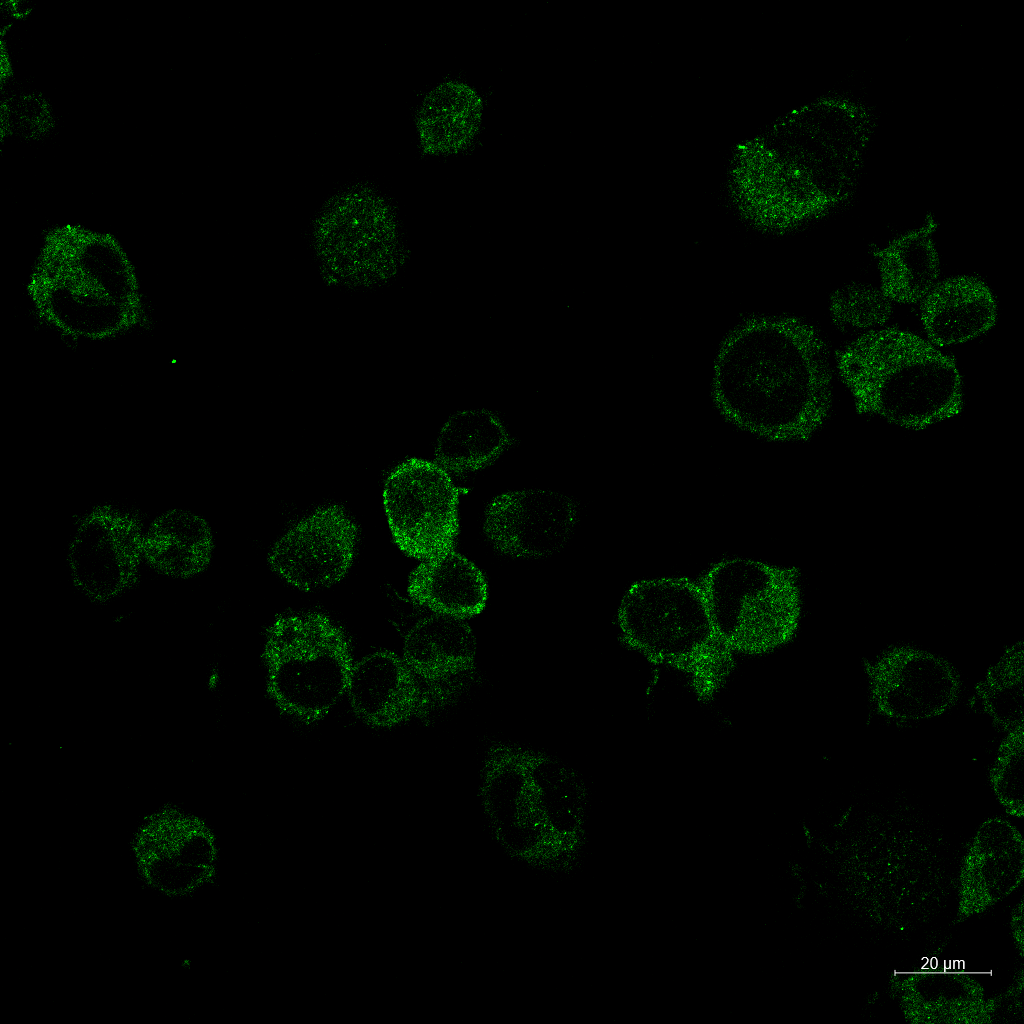

Supplement: Supplementary file 9 — Source Data for Figure 7 [file EMMM-14-e14455-s009.zip › Figure_7/7_D/15_min/Image_53_c3_2.tif]

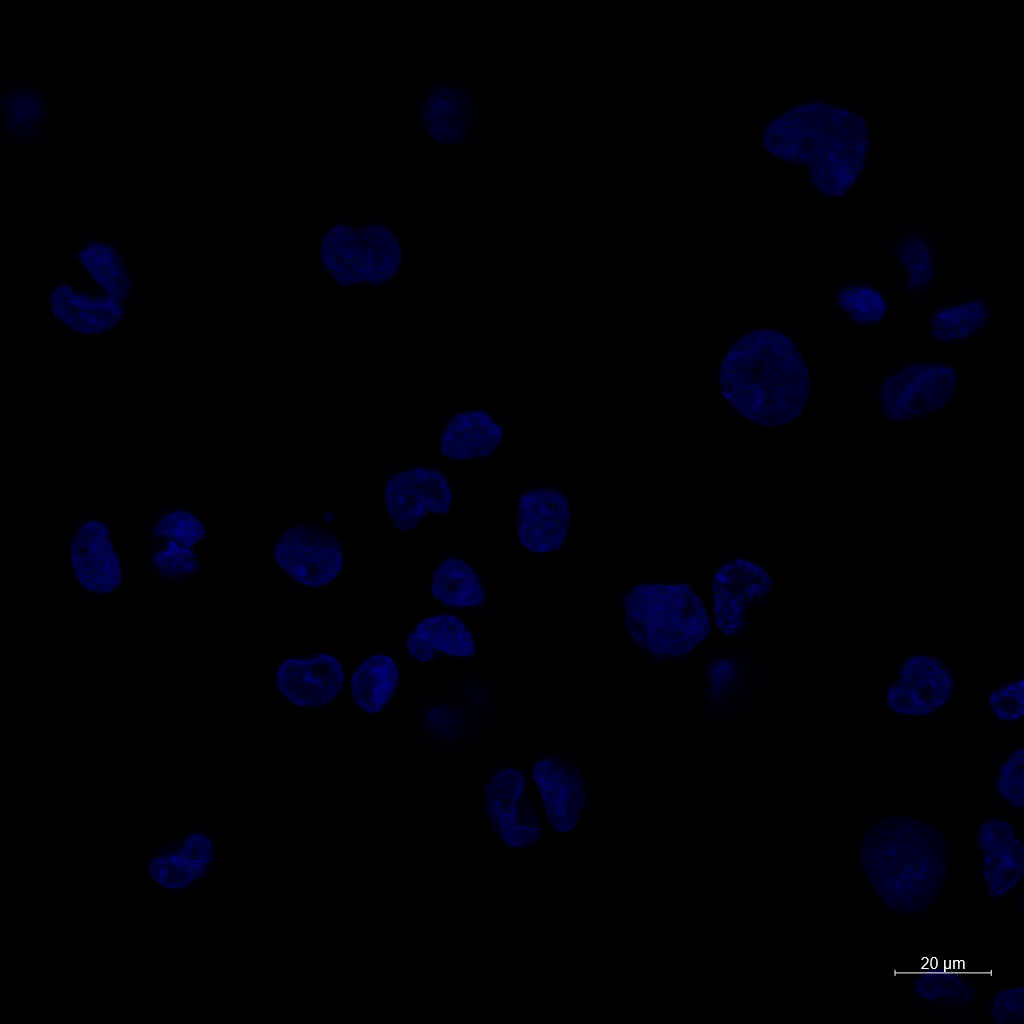

Supplement: Supplementary file 9 — Source Data for Figure 7 [file EMMM-14-e14455-s009.zip › Figure_7/7_D/15_min/Image_53_c4.tif]

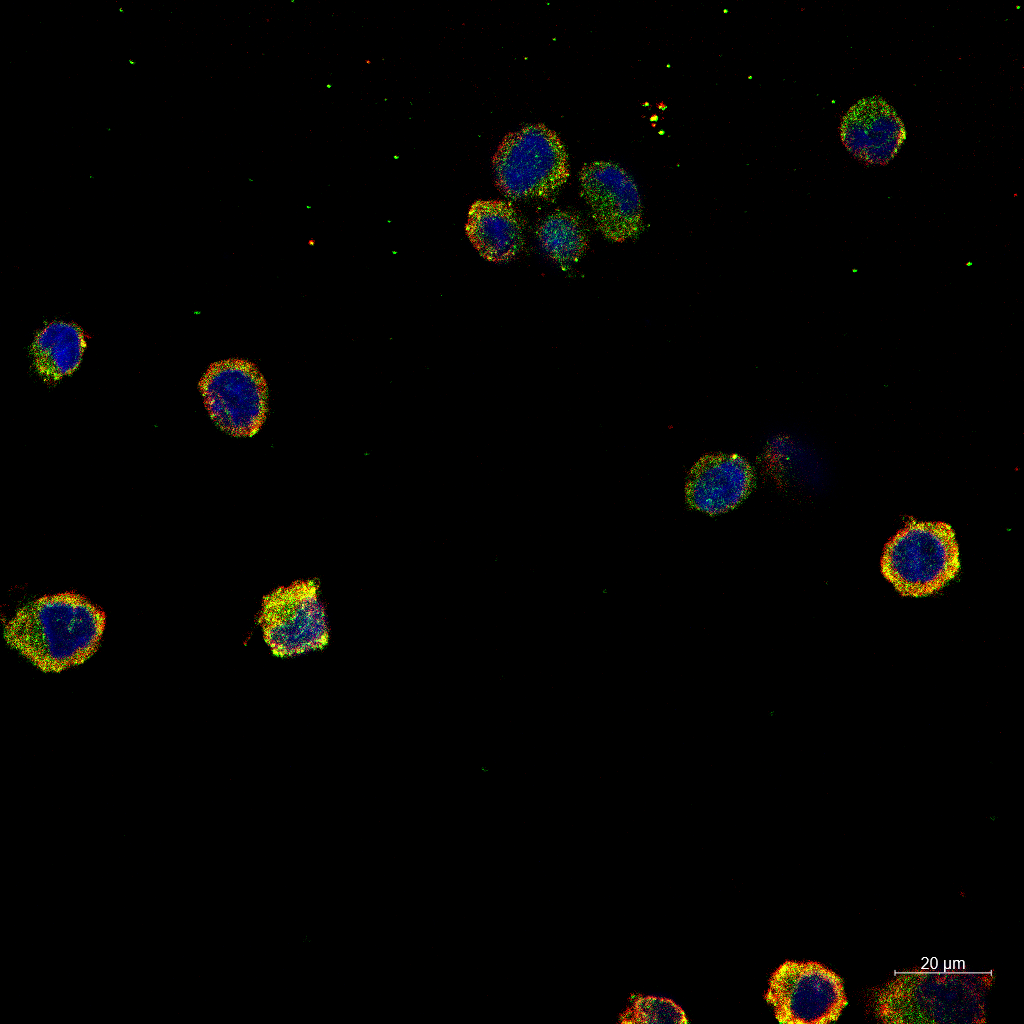

Supplement: Supplementary file 9 — Source Data for Figure 7 [file EMMM-14-e14455-s009.zip › Figure_7/7_D/5_min/5.tif]

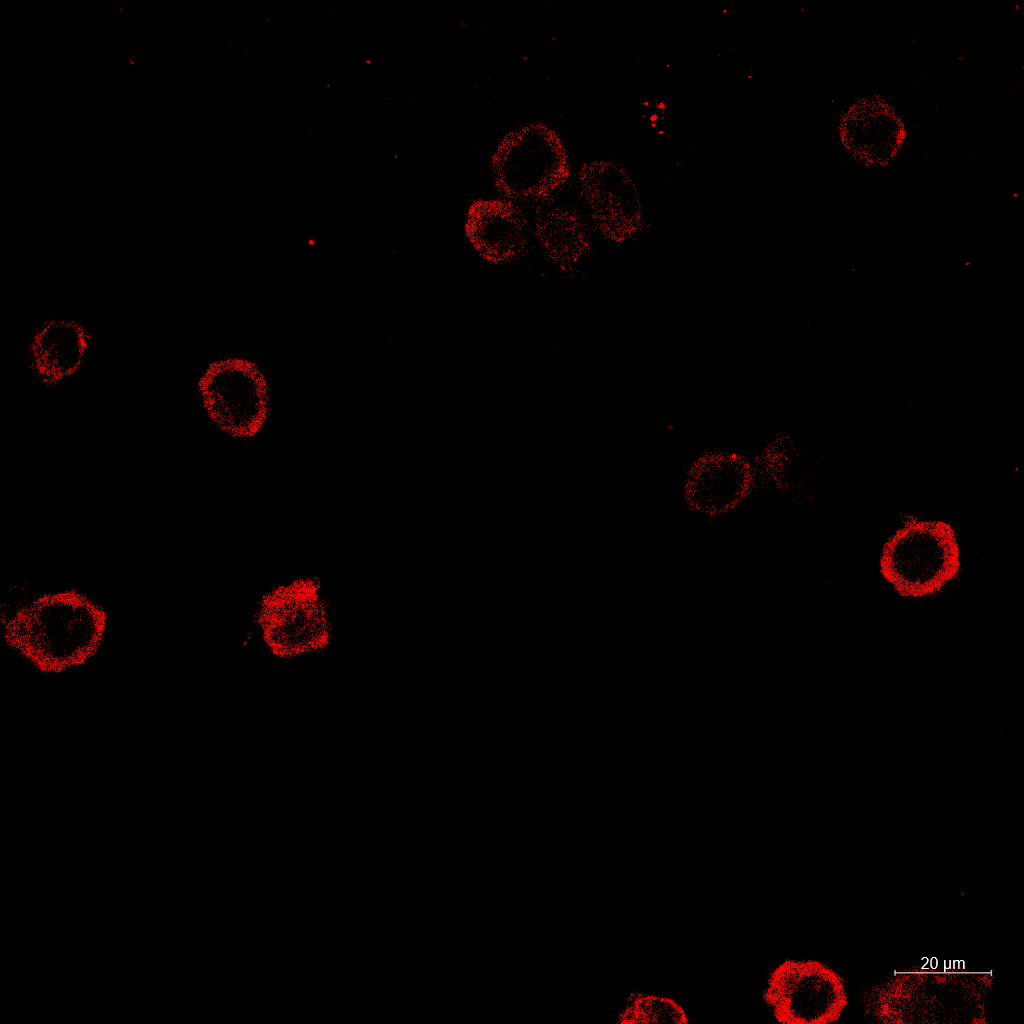

Supplement: Supplementary file 9 — Source Data for Figure 7 [file EMMM-14-e14455-s009.zip › Figure_7/7_D/5_min/Image_42_c1.tif]
