# Supplementary figures and images for: Allosteric inhibition of SHP2 uncovers aberrant TLR7 trafficking in aggravating psoriasis (part 2 of 2)
Source: EMBO Mol Med. 2021 Dec 22;14(3):e14455. doi: 10.15252/emmm.202114455 (PMC8899919; doi:10.15252/emmm.202114455)

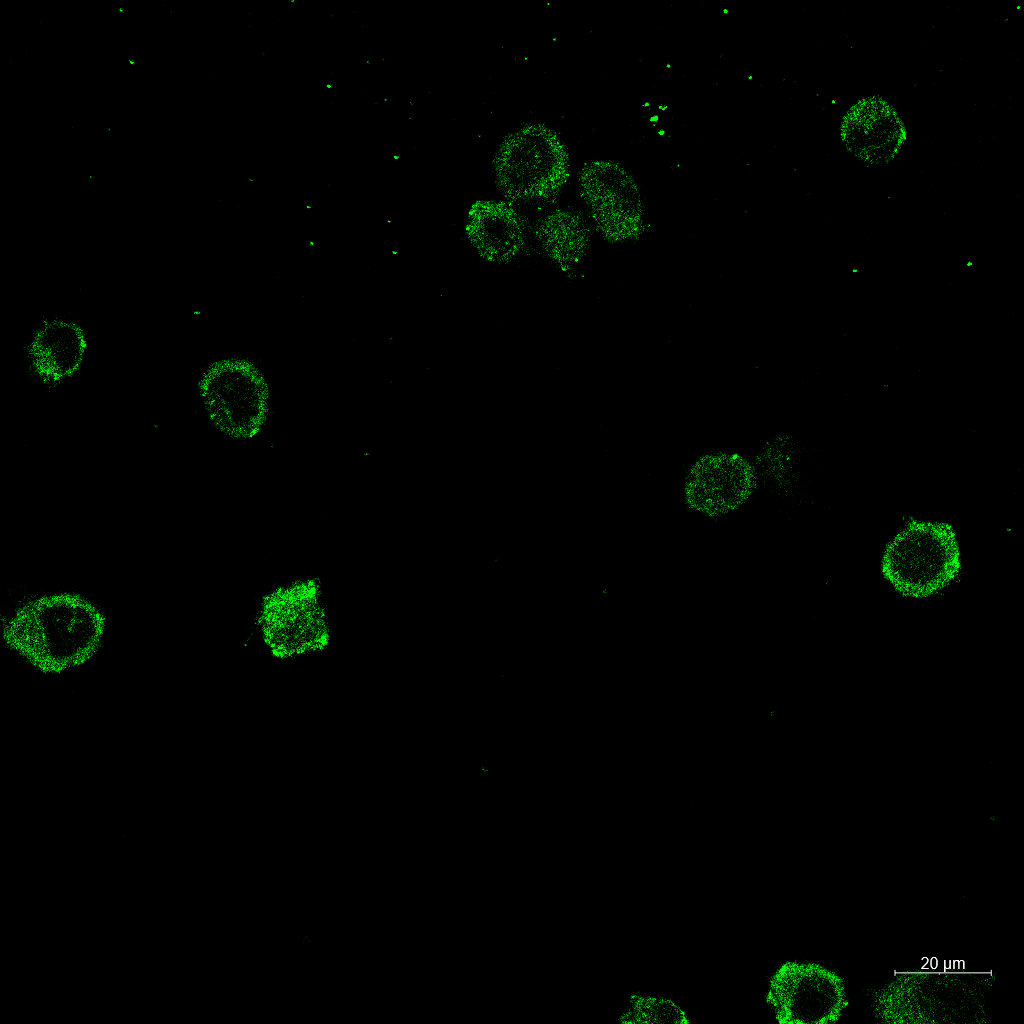

Supplement: Supplementary file 9 — Source Data for Figure 7 [file EMMM-14-e14455-s009.zip › Figure_7/7_D/5_min/Image_42_c3_2.tif]

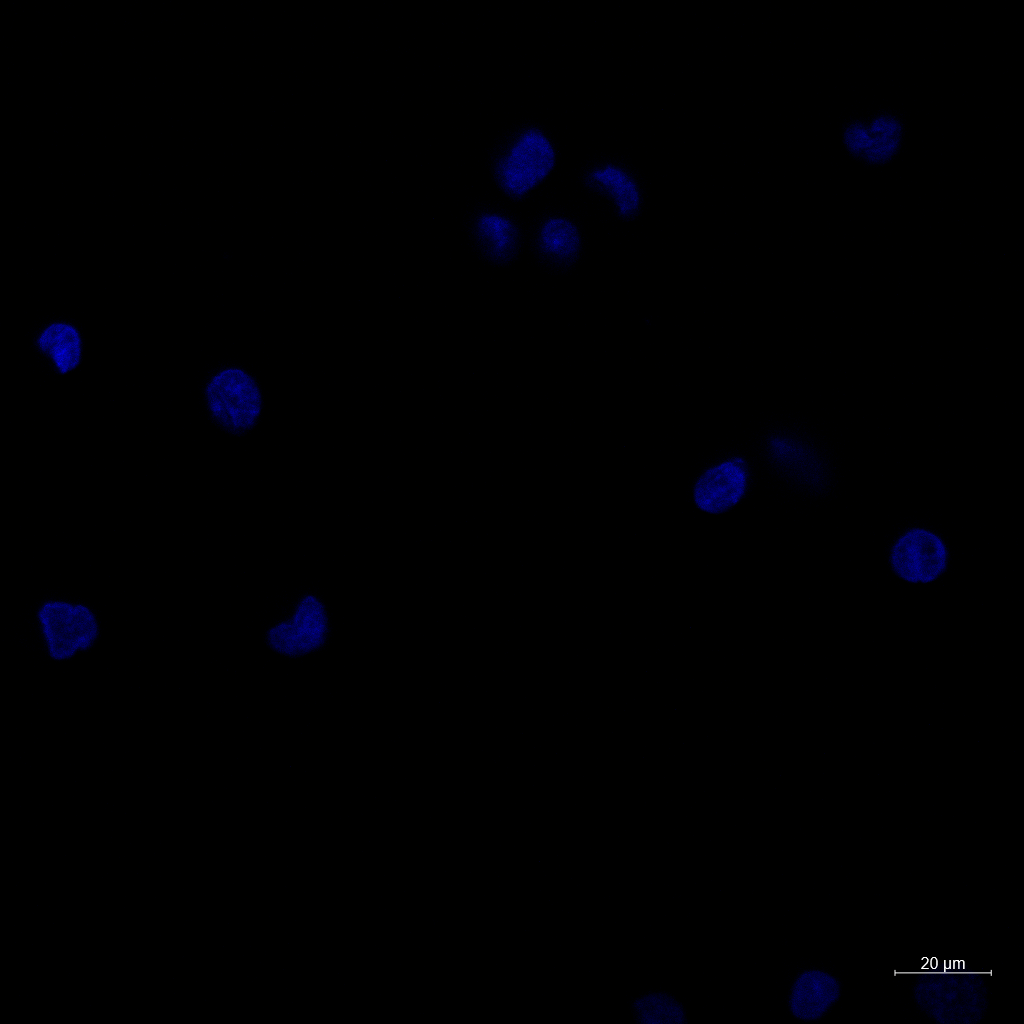

Supplement: Supplementary file 9 — Source Data for Figure 7 [file EMMM-14-e14455-s009.zip › Figure_7/7_D/5_min/Image_42_c4.tif]

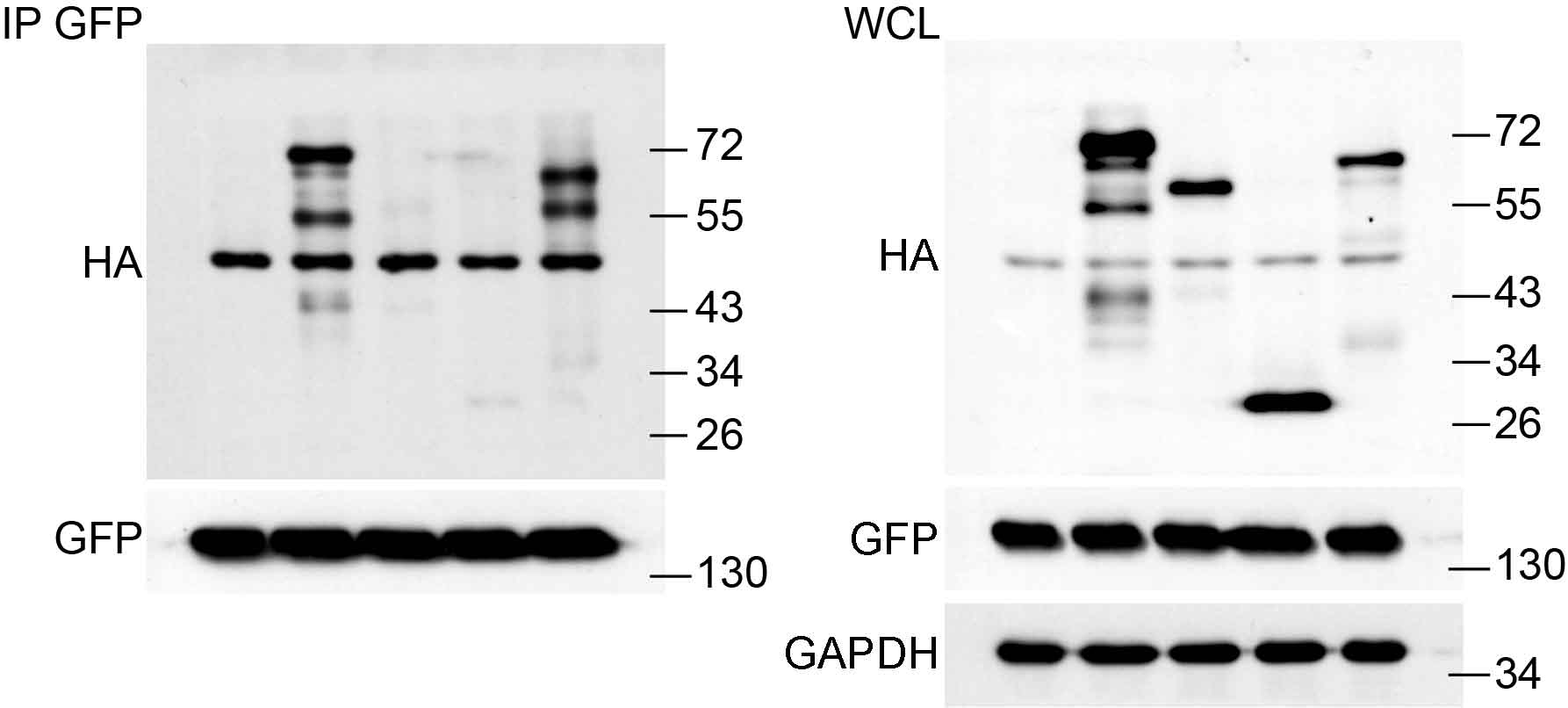

Supplement: Supplementary file 9 — Source Data for Figure 7 [file EMMM-14-e14455-s009.zip › Figure_7/7_E/7_E.jpg]

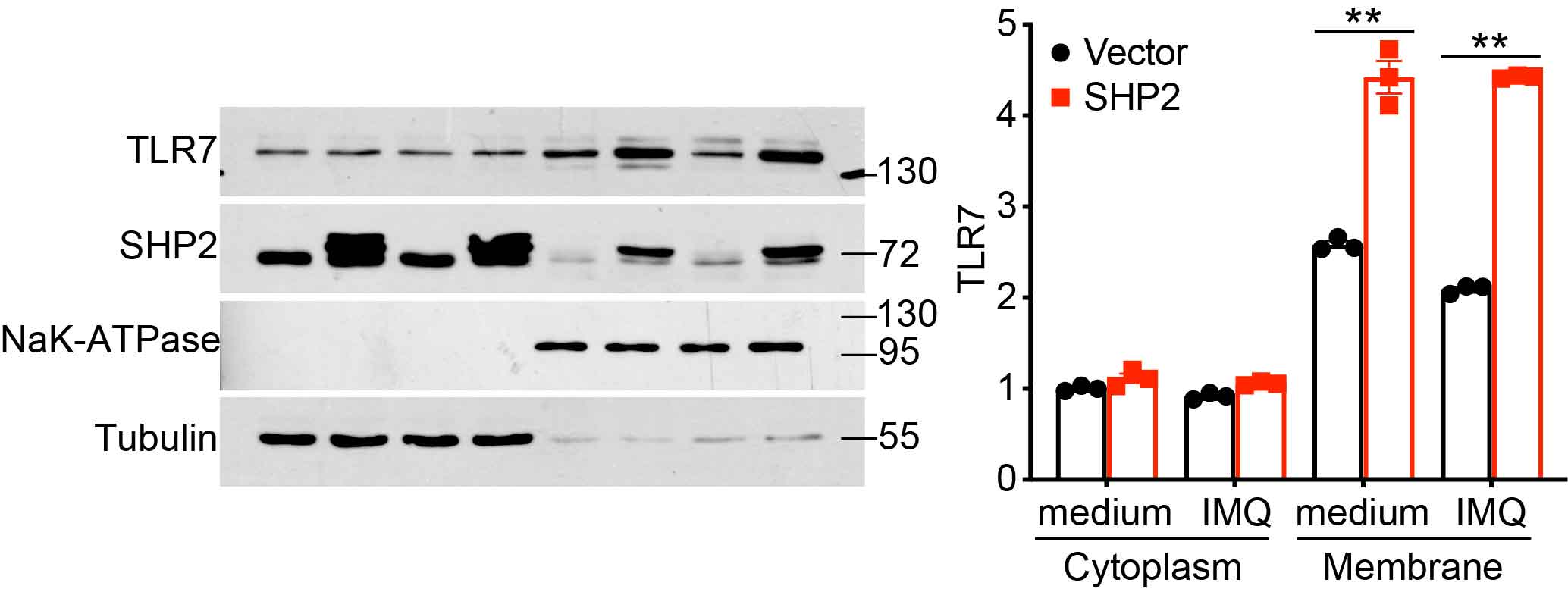

Supplement: Supplementary file 9 — Source Data for Figure 7 [file EMMM-14-e14455-s009.zip › Figure_7/7_F/7_F.jpg]

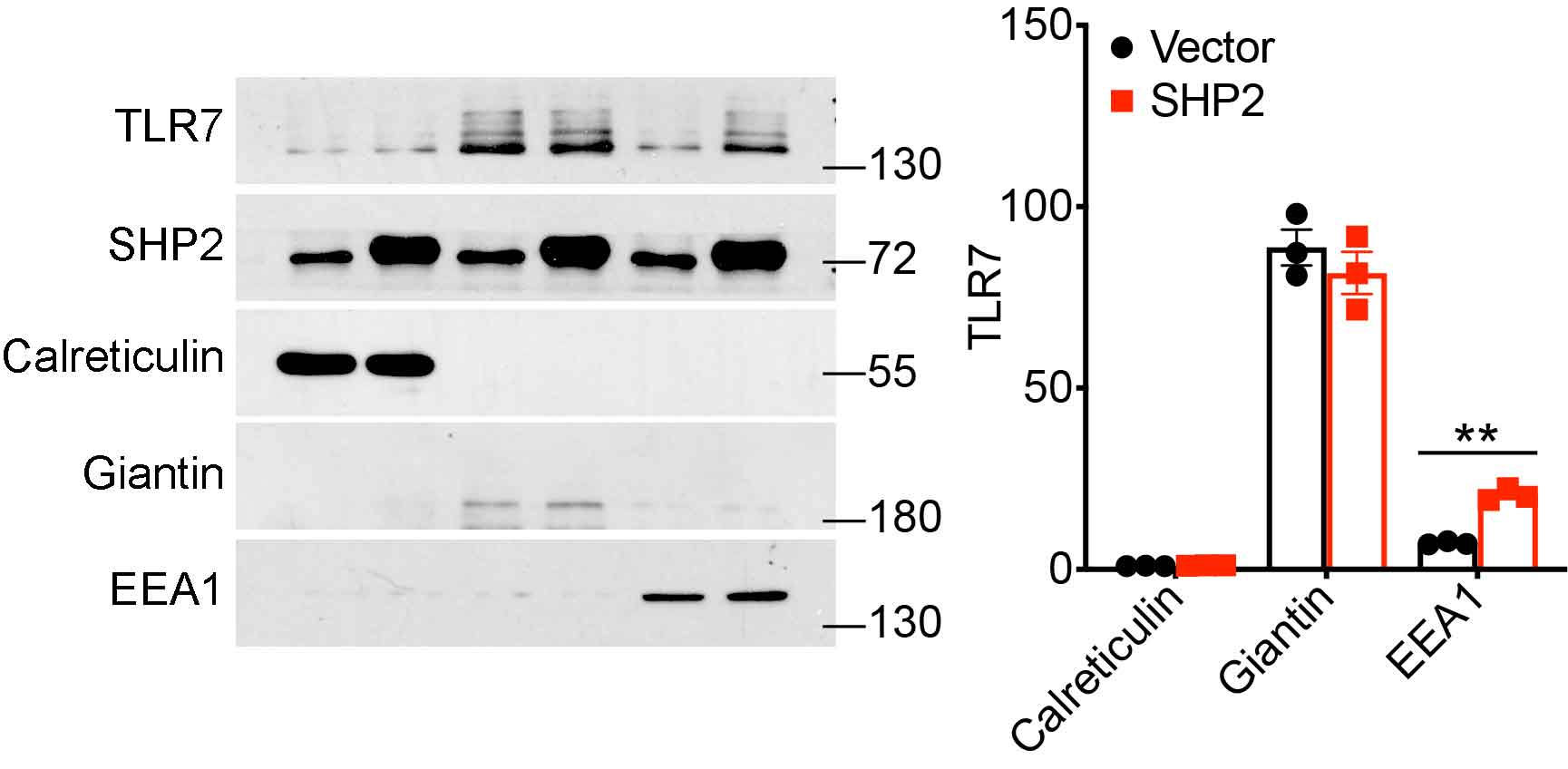

Supplement: Supplementary file 9 — Source Data for Figure 7 [file EMMM-14-e14455-s009.zip › Figure_7/7_G/7_G.jpg]

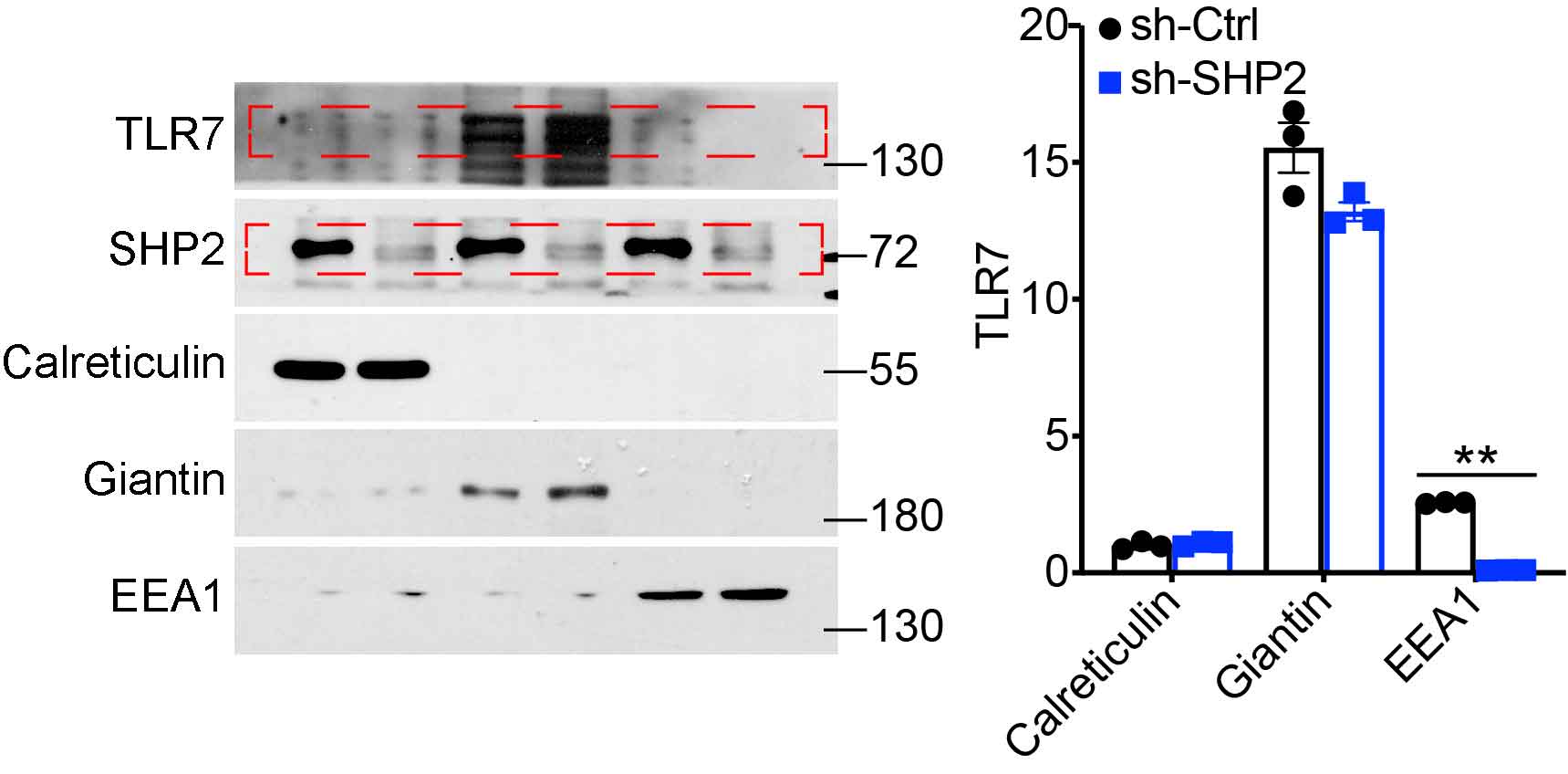

Supplement: Supplementary file 9 — Source Data for Figure 7 [file EMMM-14-e14455-s009.zip › Figure_7/7_H/7_H.jpg]

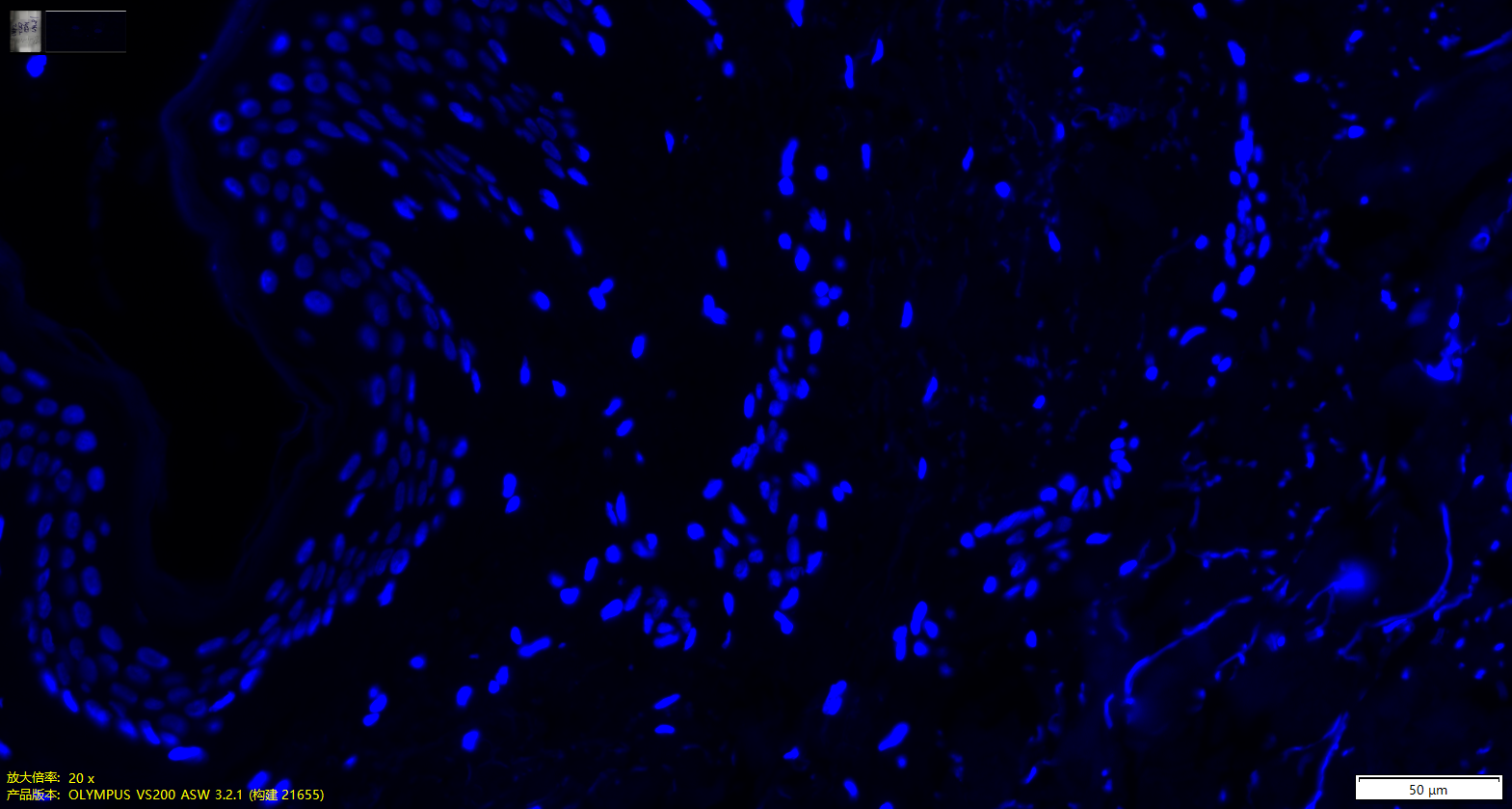

Supplement: Supplementary file 9 — Source Data for Figure 7 [file EMMM-14-e14455-s009.zip › Figure_7/7_I/EEA1/Normal-DAPI.tif]

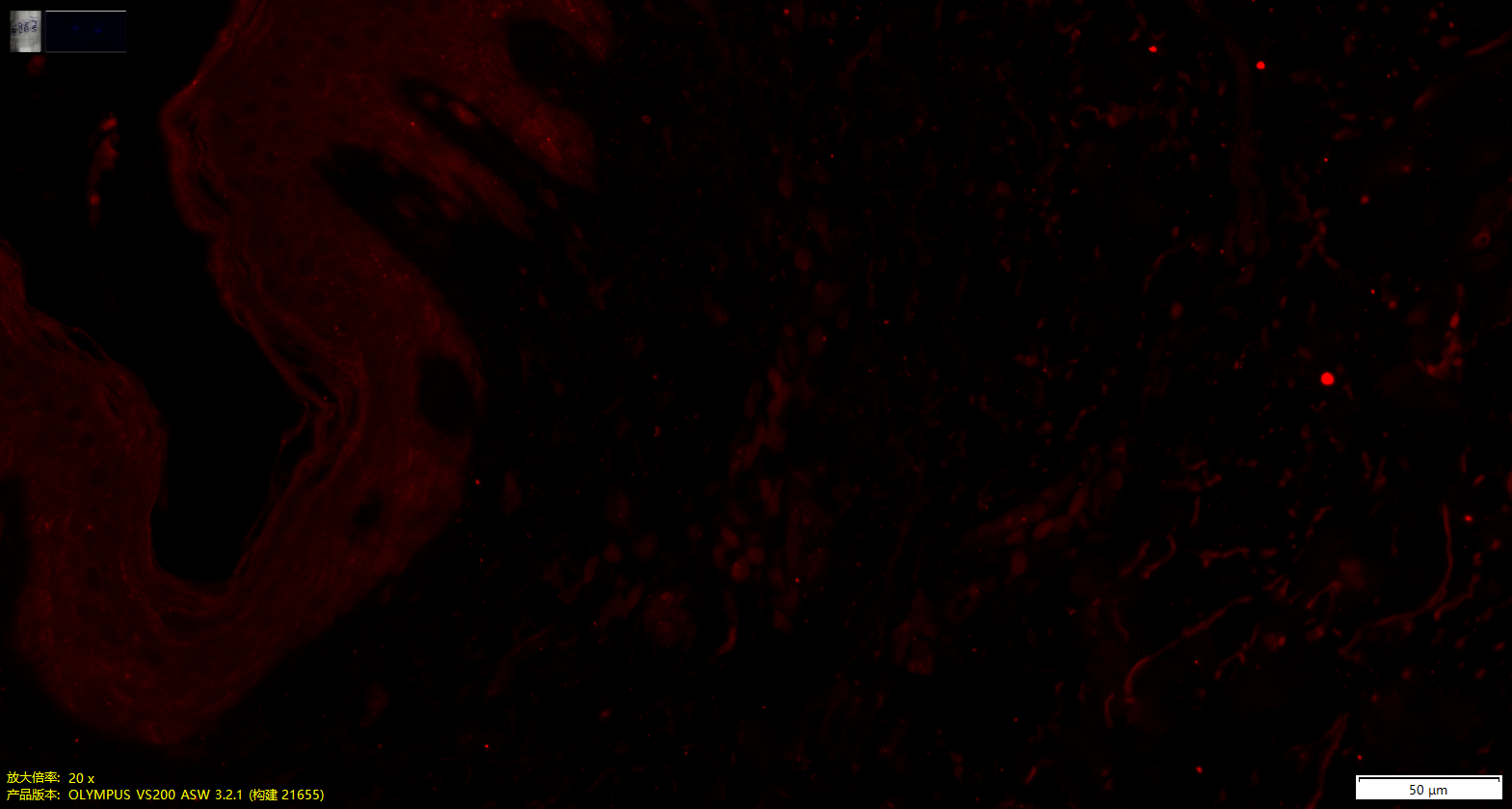

Supplement: Supplementary file 9 — Source Data for Figure 7 [file EMMM-14-e14455-s009.zip › Figure_7/7_I/EEA1/Normal-EEA1.tif]

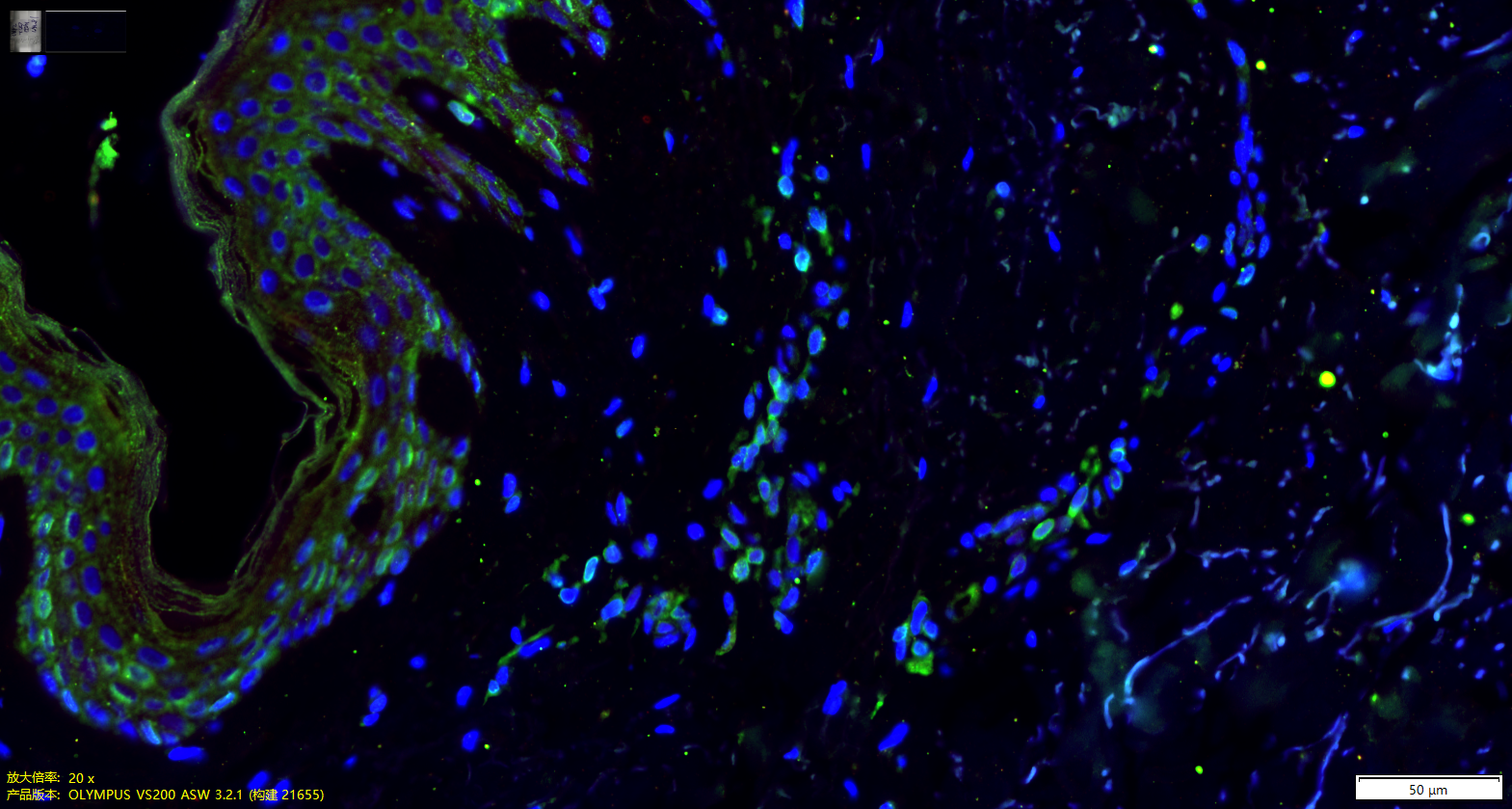

Supplement: Supplementary file 9 — Source Data for Figure 7 [file EMMM-14-e14455-s009.zip › Figure_7/7_I/EEA1/Normal-MERGE.tif]

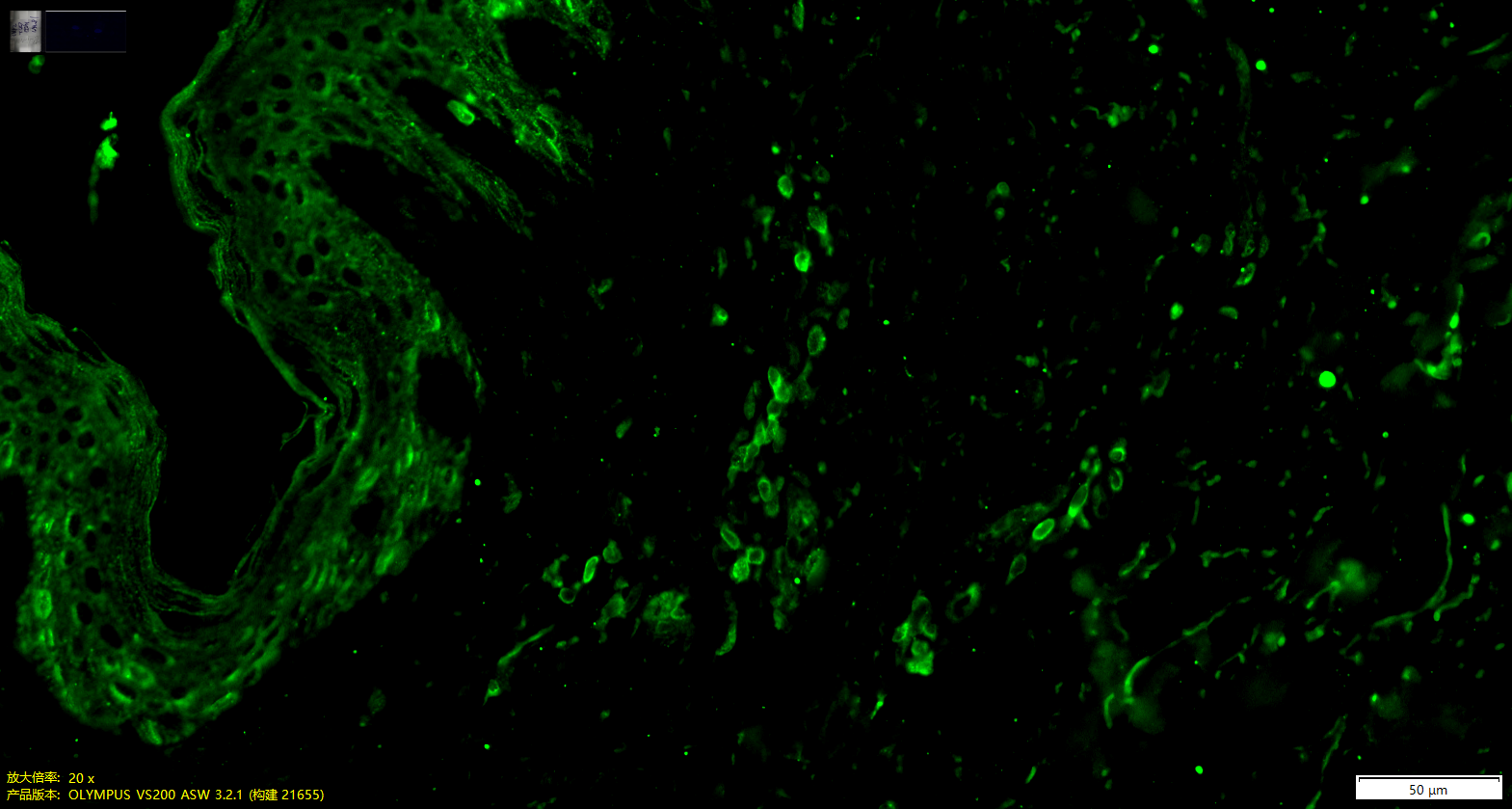

Supplement: Supplementary file 9 — Source Data for Figure 7 [file EMMM-14-e14455-s009.zip › Figure_7/7_I/EEA1/Normal-TLR7.tif]

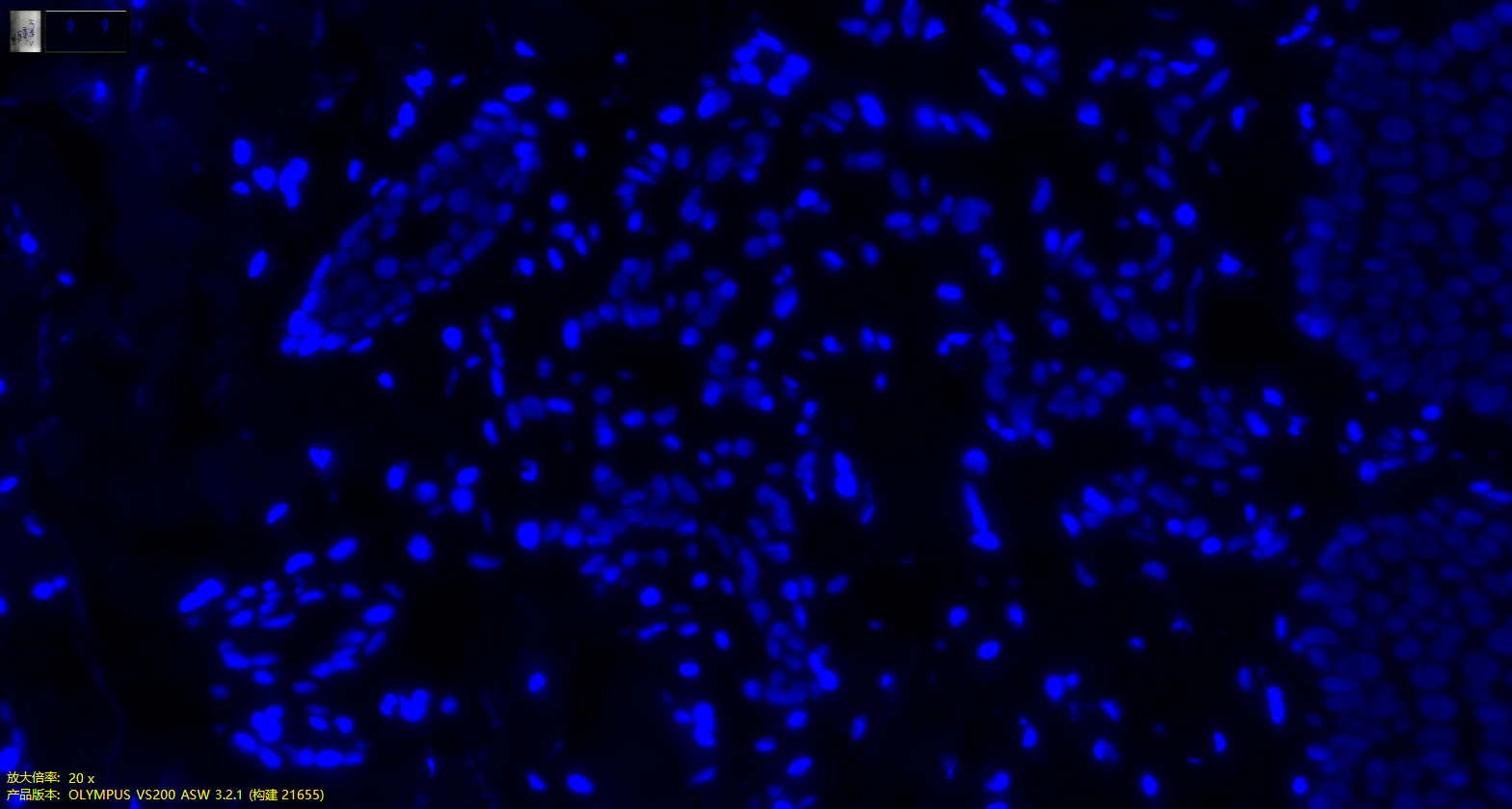

Supplement: Supplementary file 9 — Source Data for Figure 7 [file EMMM-14-e14455-s009.zip › Figure_7/7_I/EEA1/Psoriasis-DAPI.tif]

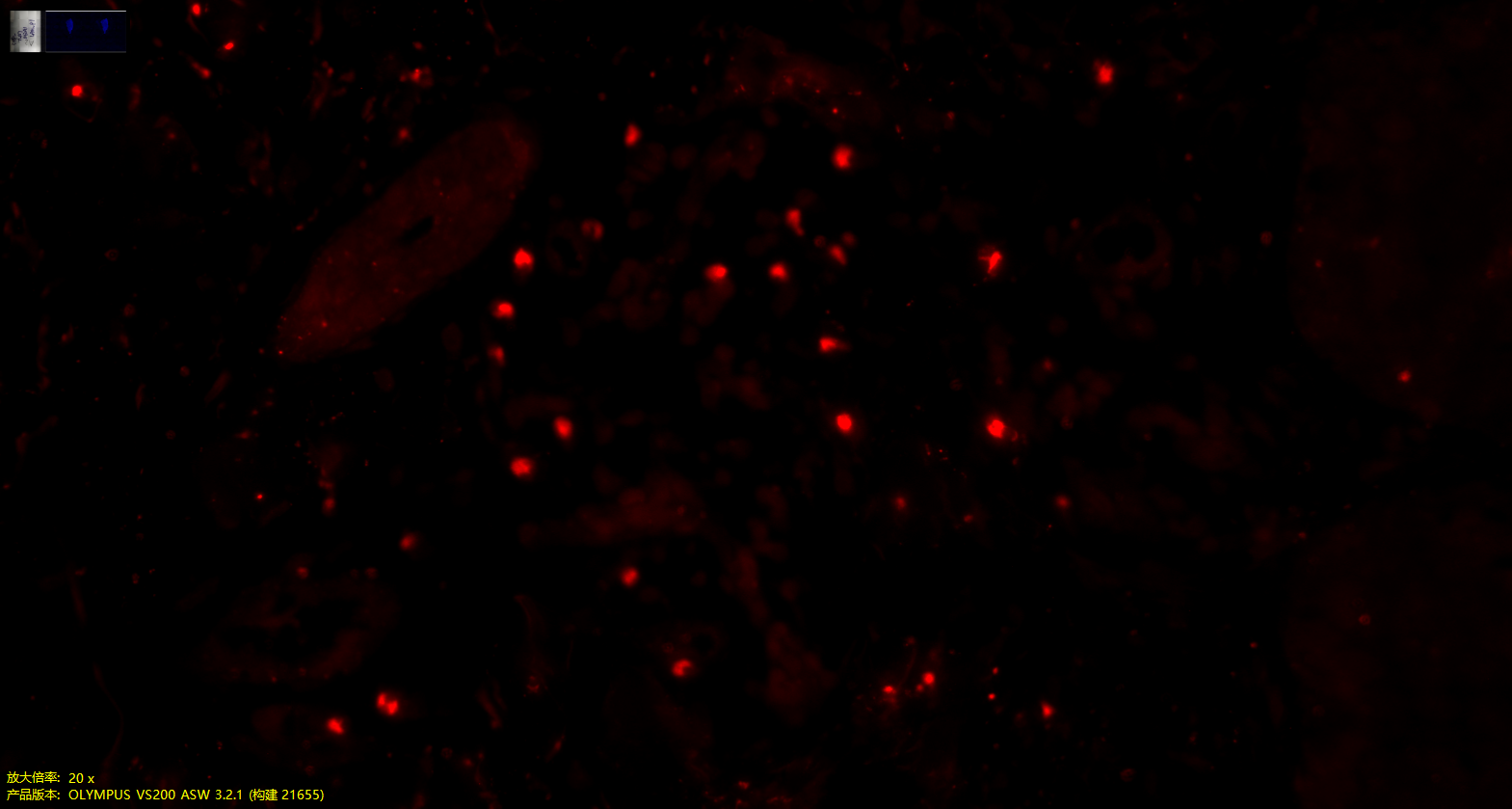

Supplement: Supplementary file 9 — Source Data for Figure 7 [file EMMM-14-e14455-s009.zip › Figure_7/7_I/EEA1/Psoriasis-EEA1.tif]

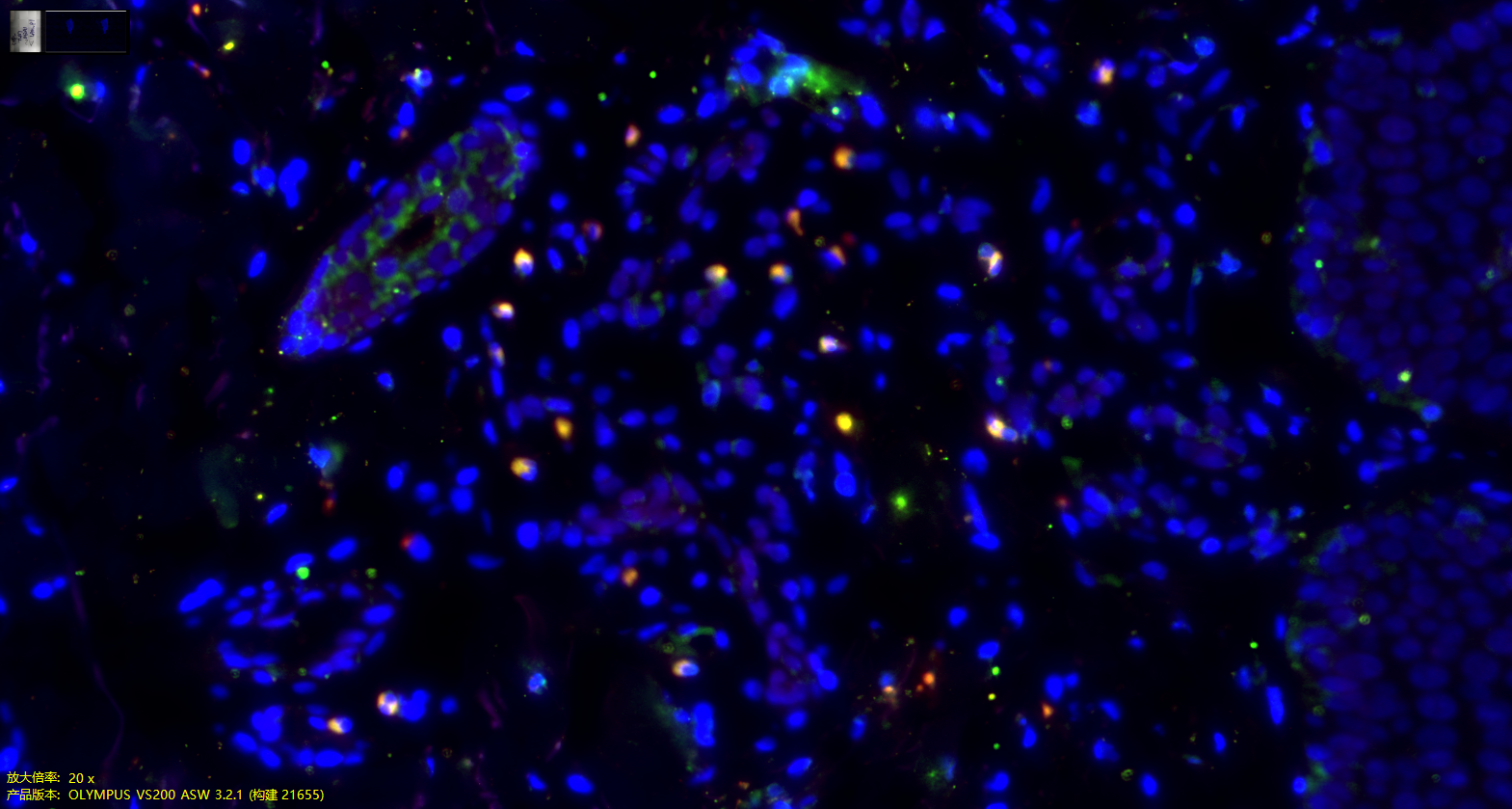

Supplement: Supplementary file 9 — Source Data for Figure 7 [file EMMM-14-e14455-s009.zip › Figure_7/7_I/EEA1/Psoriasis-MERGE.tif]

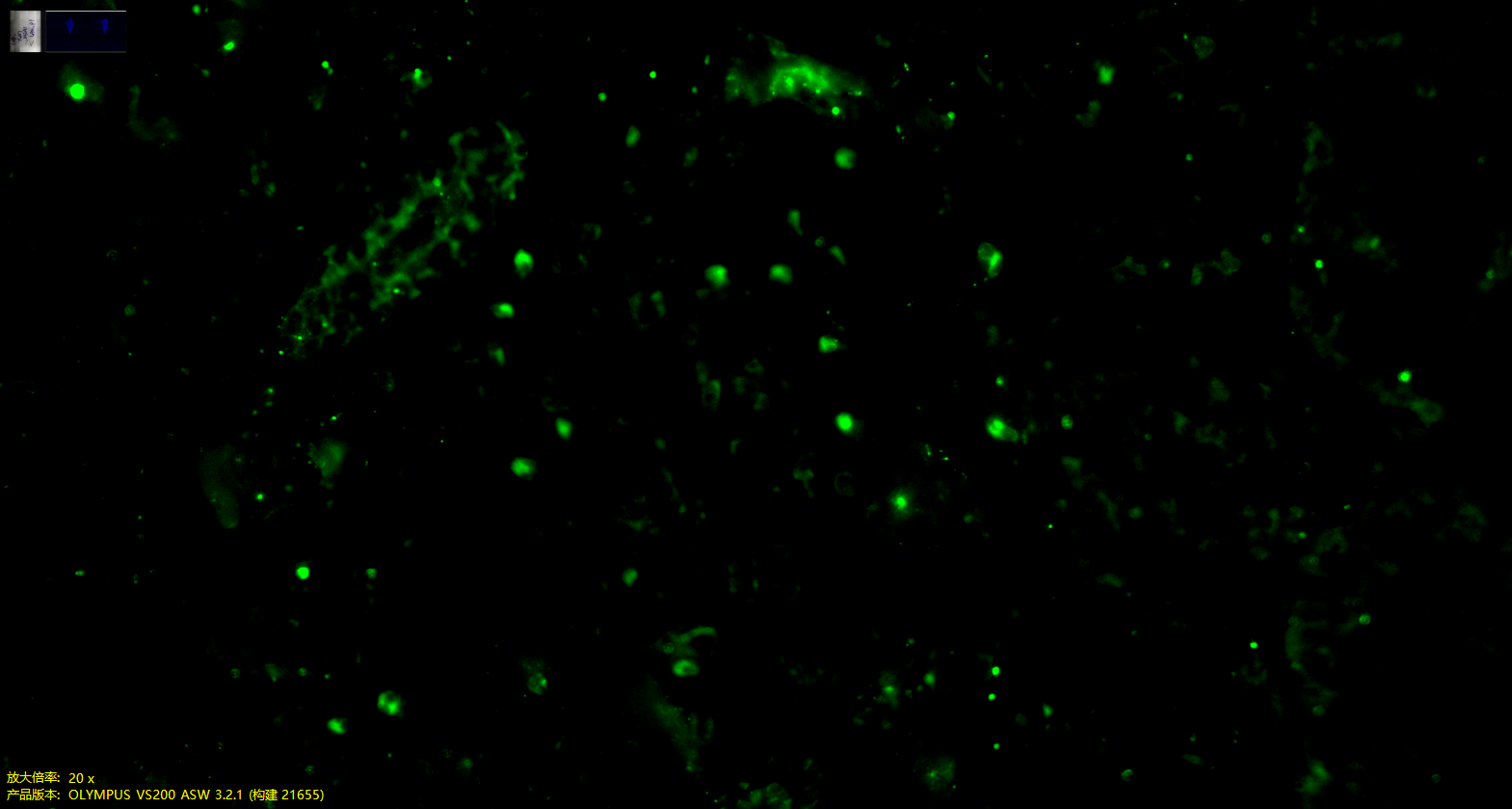

Supplement: Supplementary file 9 — Source Data for Figure 7 [file EMMM-14-e14455-s009.zip › Figure_7/7_I/EEA1/Psoriasis-TLR7.tif]

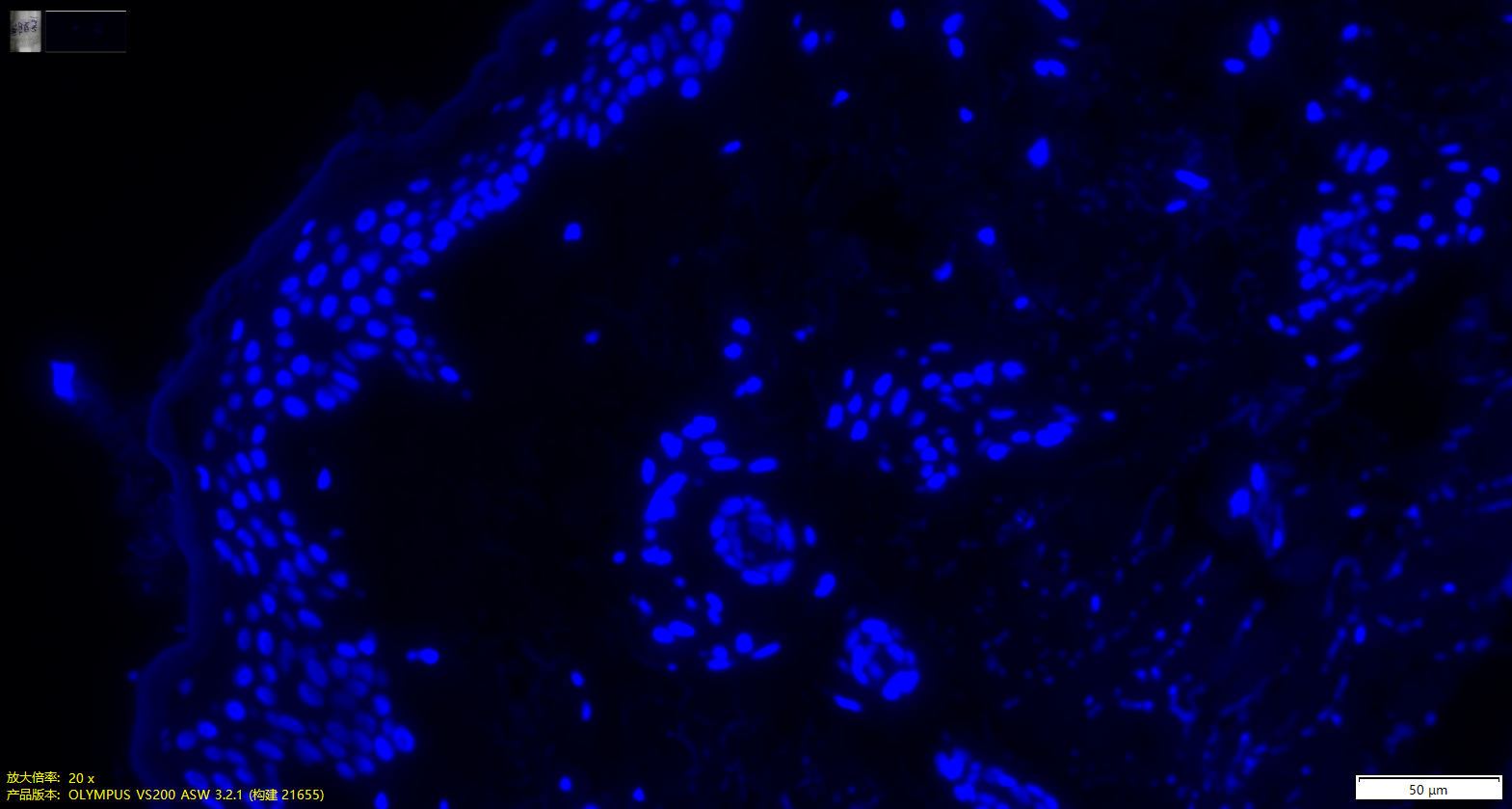

Supplement: Supplementary file 9 — Source Data for Figure 7 [file EMMM-14-e14455-s009.zip › Figure_7/7_I/LAMP1/Normal-DAPI.tif]

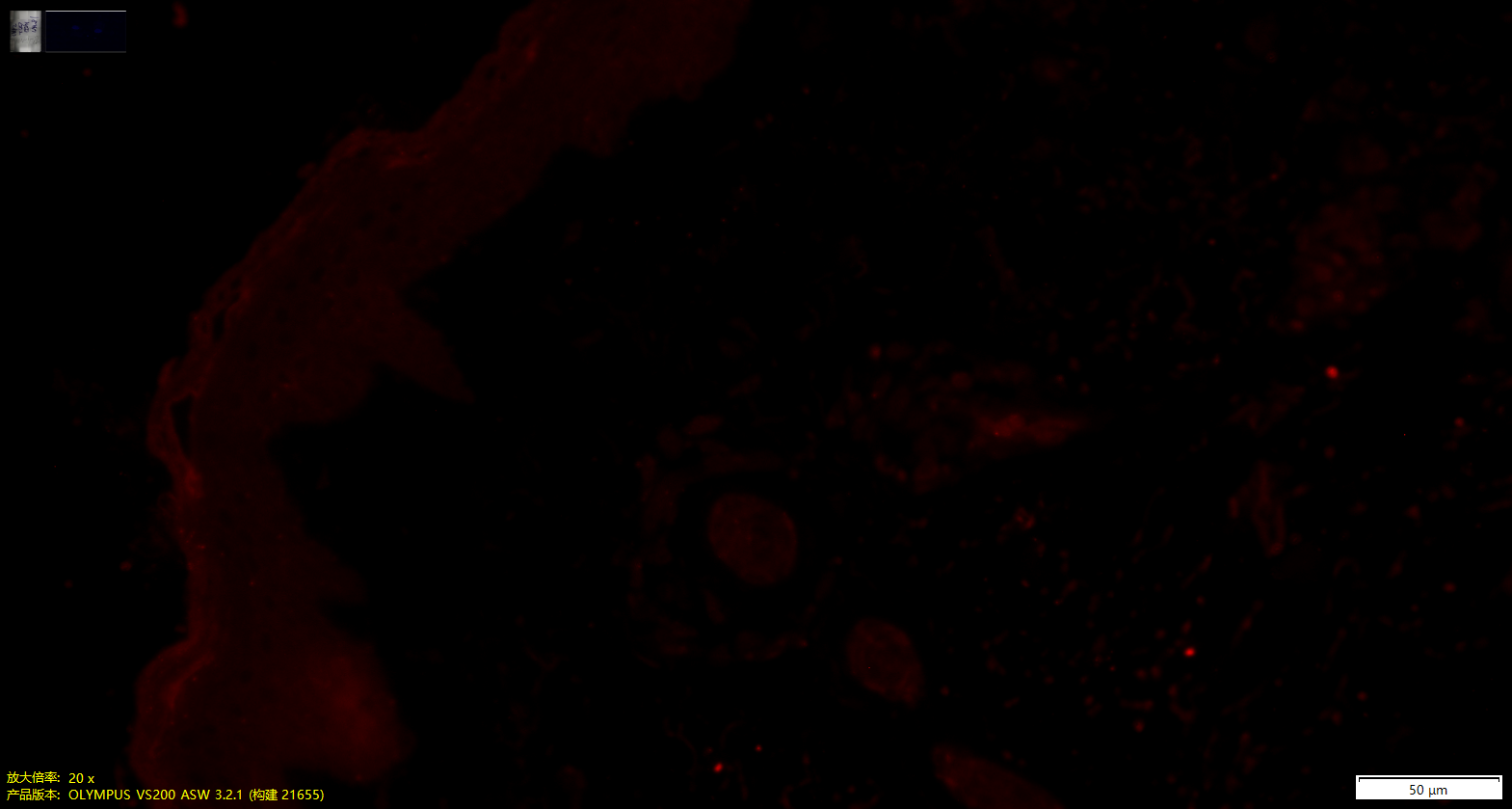

Supplement: Supplementary file 9 — Source Data for Figure 7 [file EMMM-14-e14455-s009.zip › Figure_7/7_I/LAMP1/Normal-LAMP1.tif]

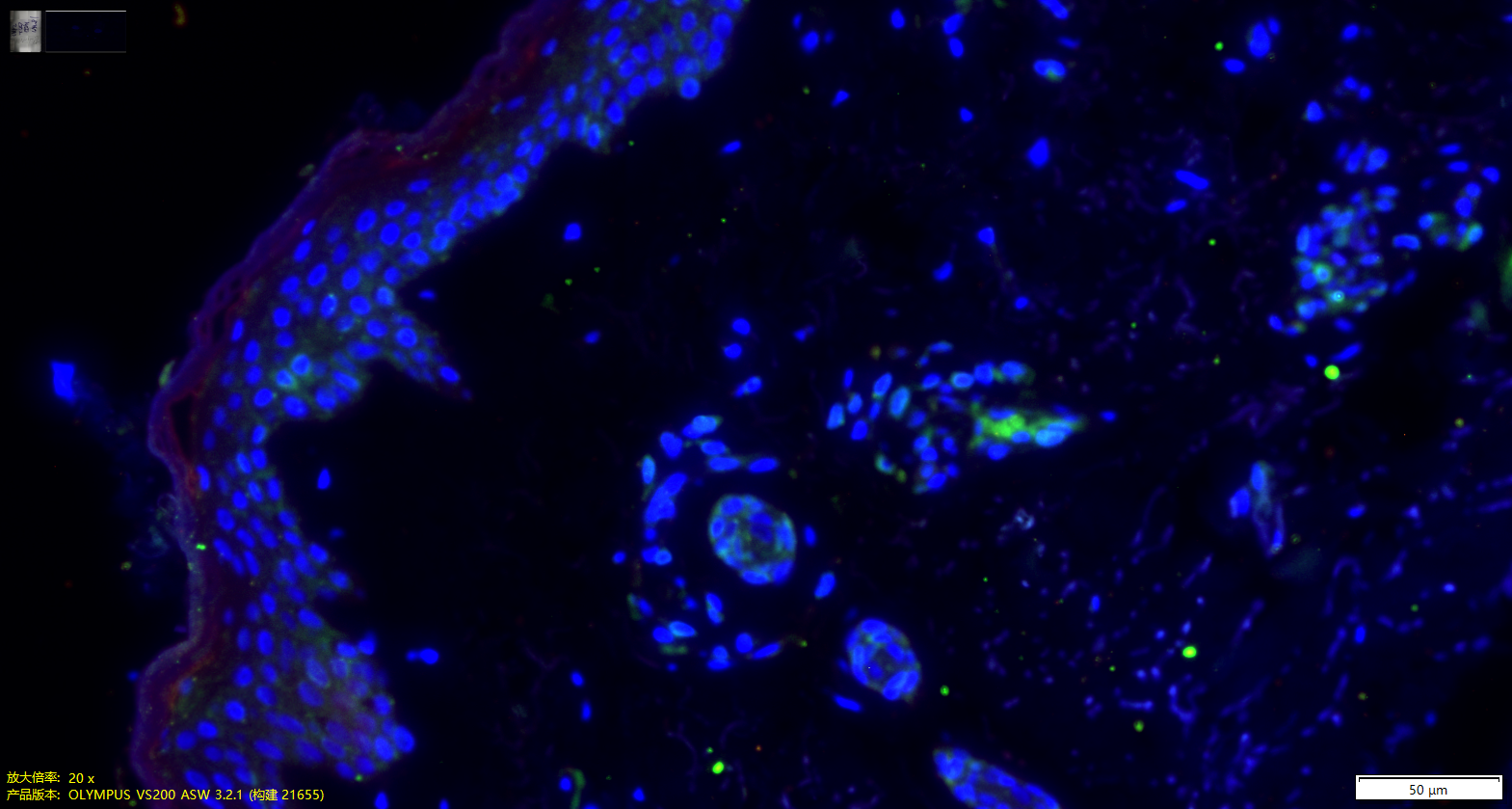

Supplement: Supplementary file 9 — Source Data for Figure 7 [file EMMM-14-e14455-s009.zip › Figure_7/7_I/LAMP1/Normal-MERGE.tif]

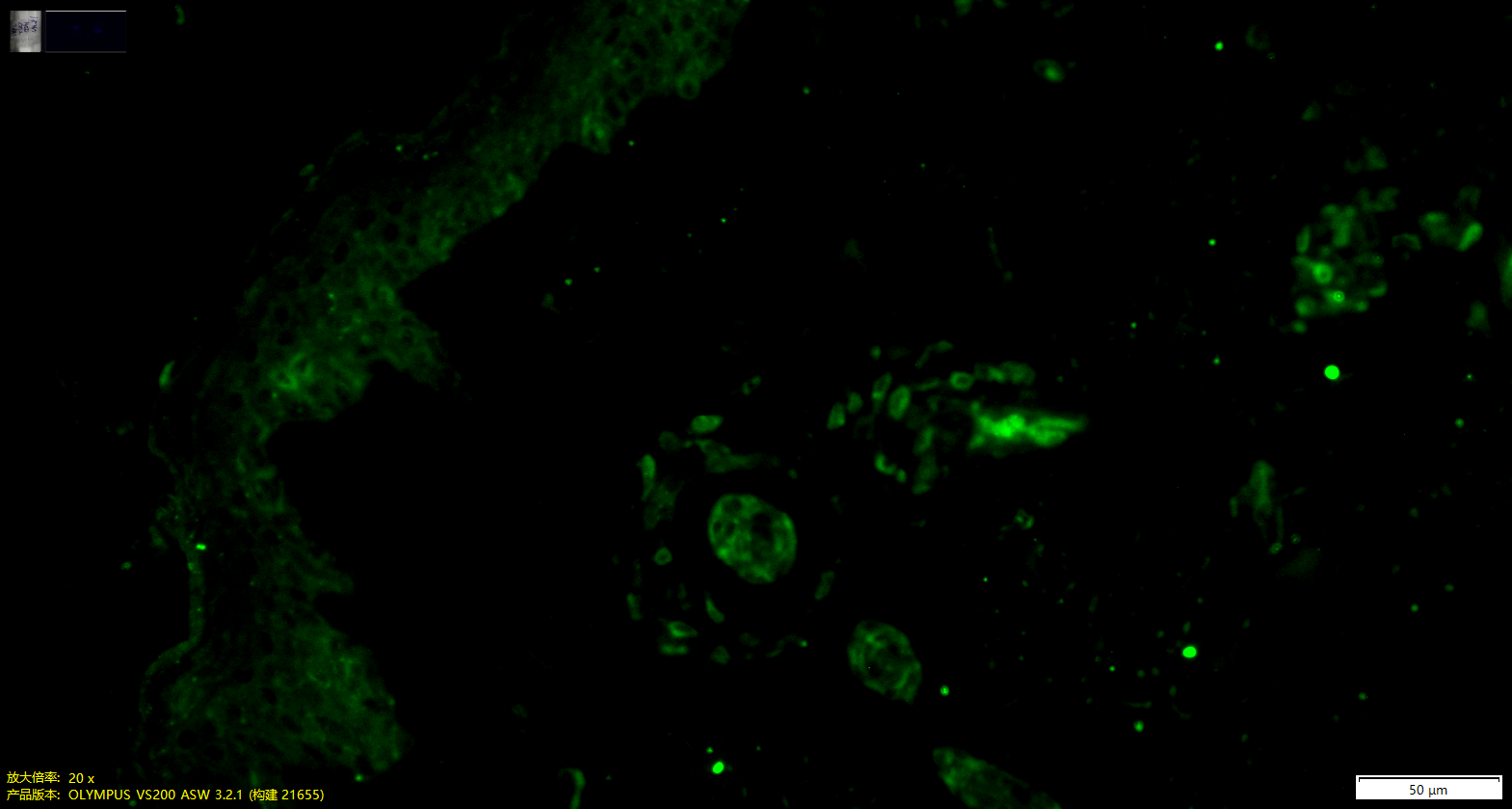

Supplement: Supplementary file 9 — Source Data for Figure 7 [file EMMM-14-e14455-s009.zip › Figure_7/7_I/LAMP1/Normal-TLR7.tif]

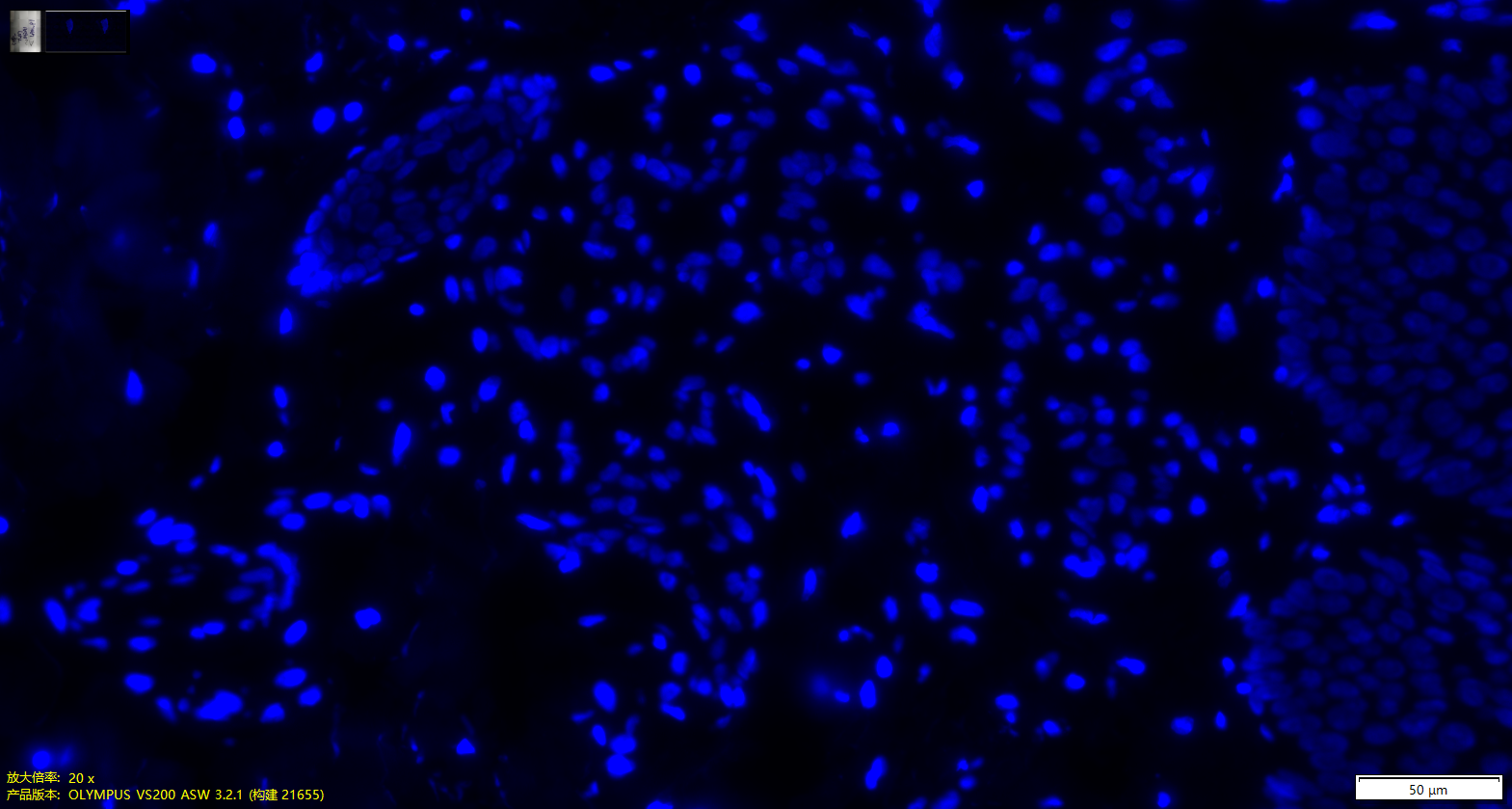

Supplement: Supplementary file 9 — Source Data for Figure 7 [file EMMM-14-e14455-s009.zip › Figure_7/7_I/LAMP1/Psoriasis-DAPI.tif]

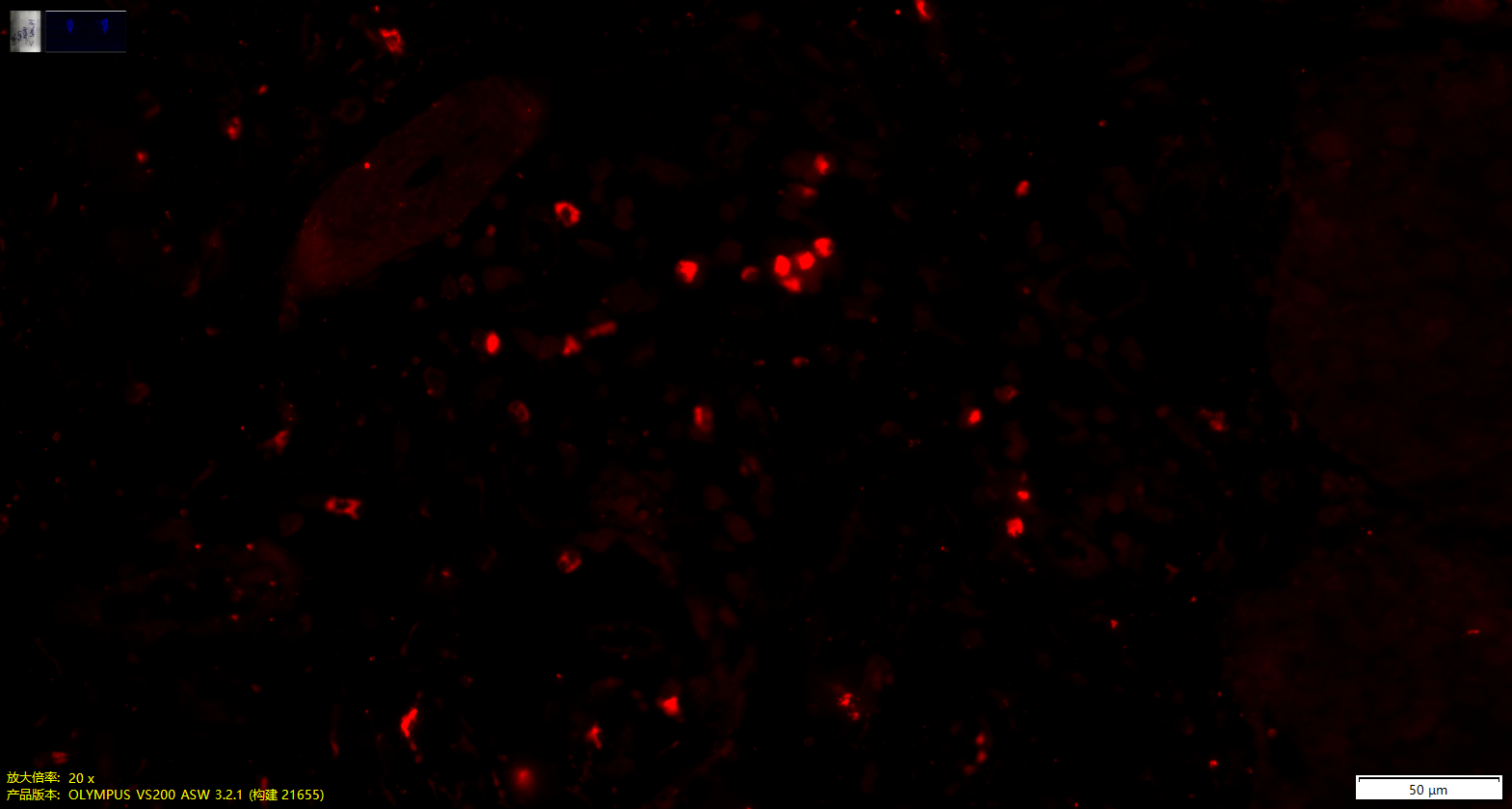

Supplement: Supplementary file 9 — Source Data for Figure 7 [file EMMM-14-e14455-s009.zip › Figure_7/7_I/LAMP1/Psoriasis-LAMP1.tif]

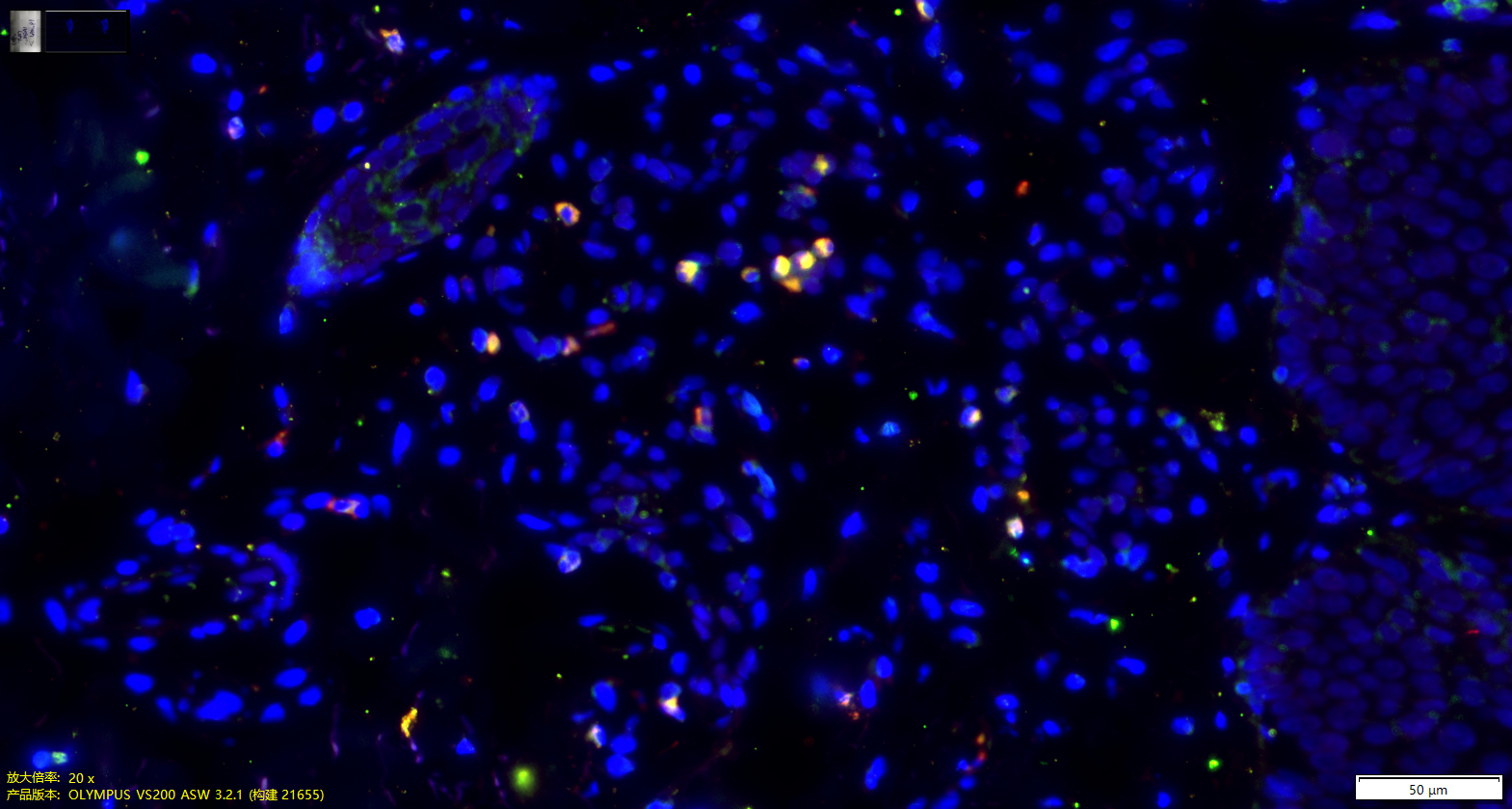

Supplement: Supplementary file 9 — Source Data for Figure 7 [file EMMM-14-e14455-s009.zip › Figure_7/7_I/LAMP1/Psoriasis-MERGE.tif]

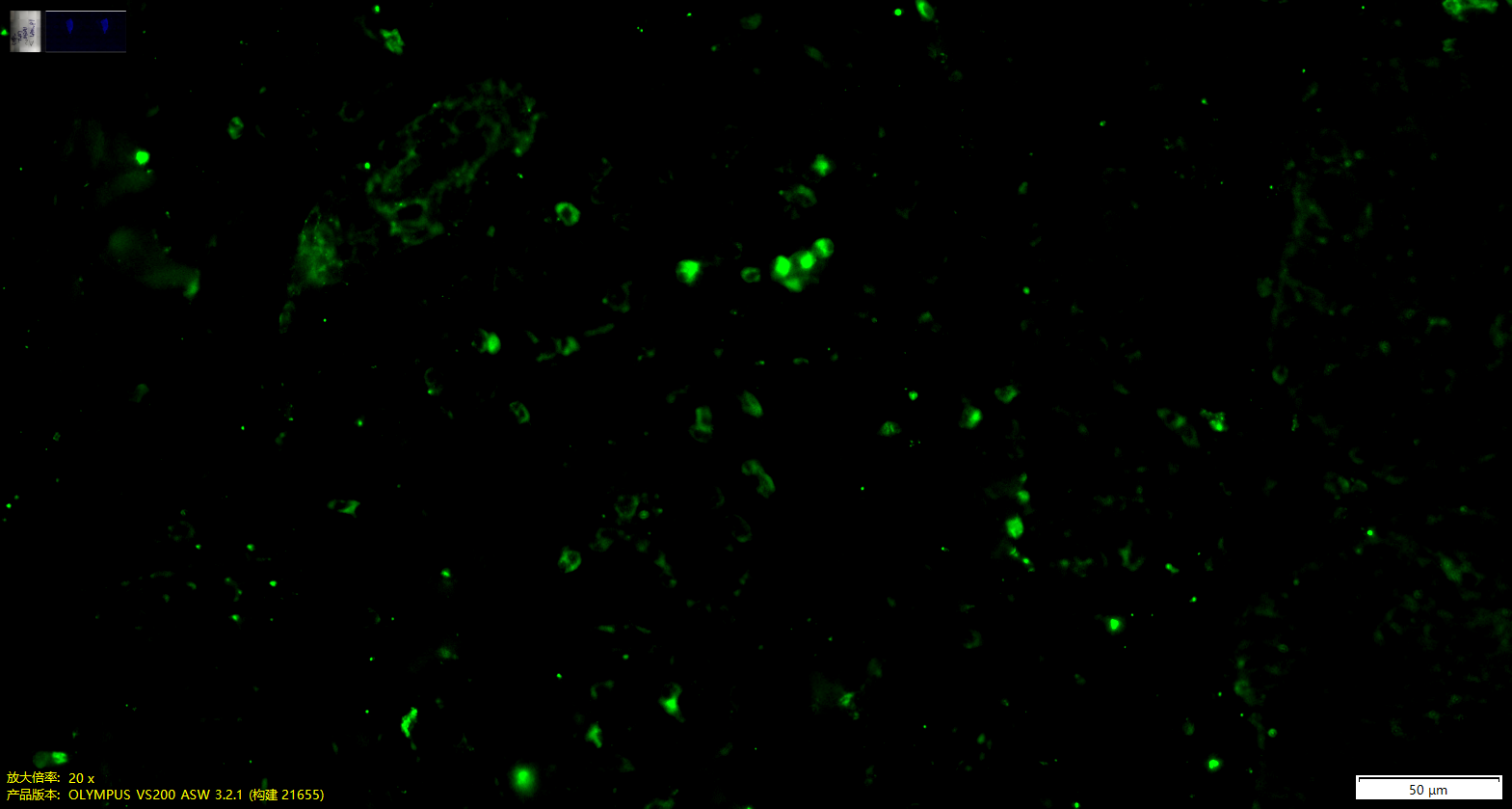

Supplement: Supplementary file 9 — Source Data for Figure 7 [file EMMM-14-e14455-s009.zip › Figure_7/7_I/LAMP1/Psoriasis-TLR7.tif]

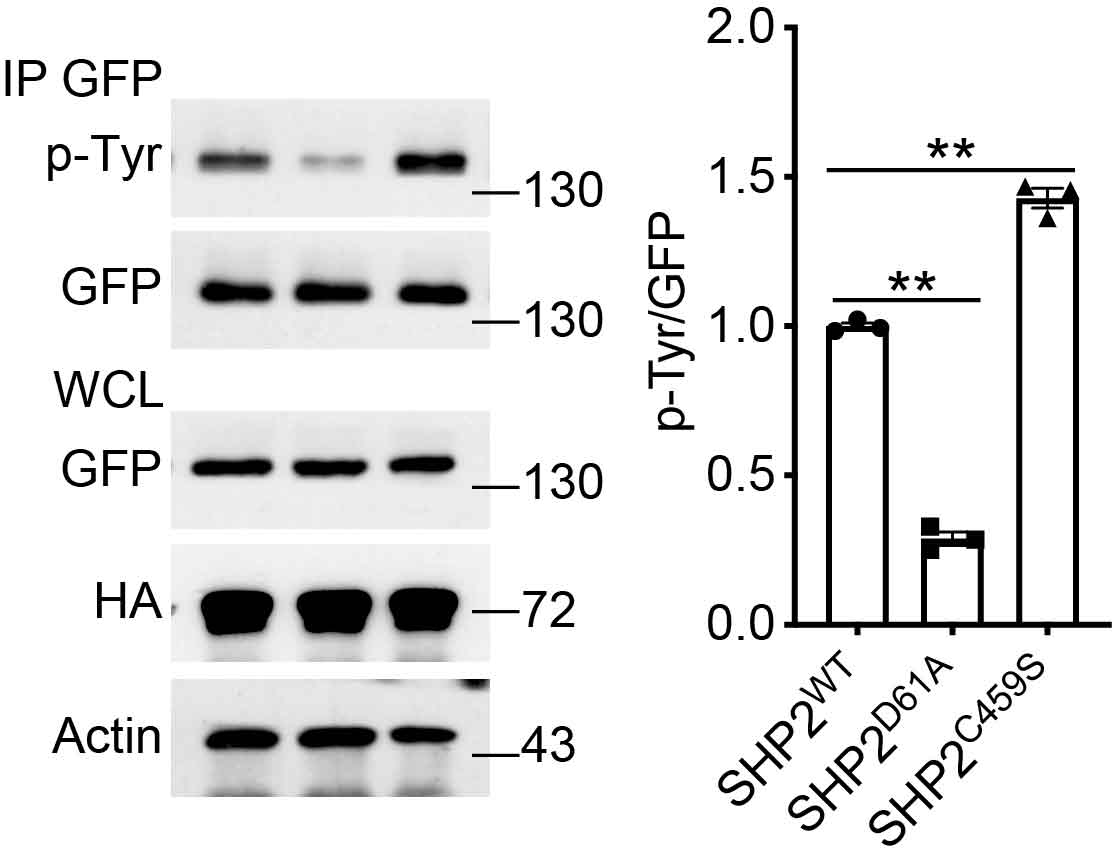

Supplement: Supplementary file 10 — Source Data for Figure 8 [file EMMM-14-e14455-s012.zip › Figure_8/8_A/8_A.jpg]

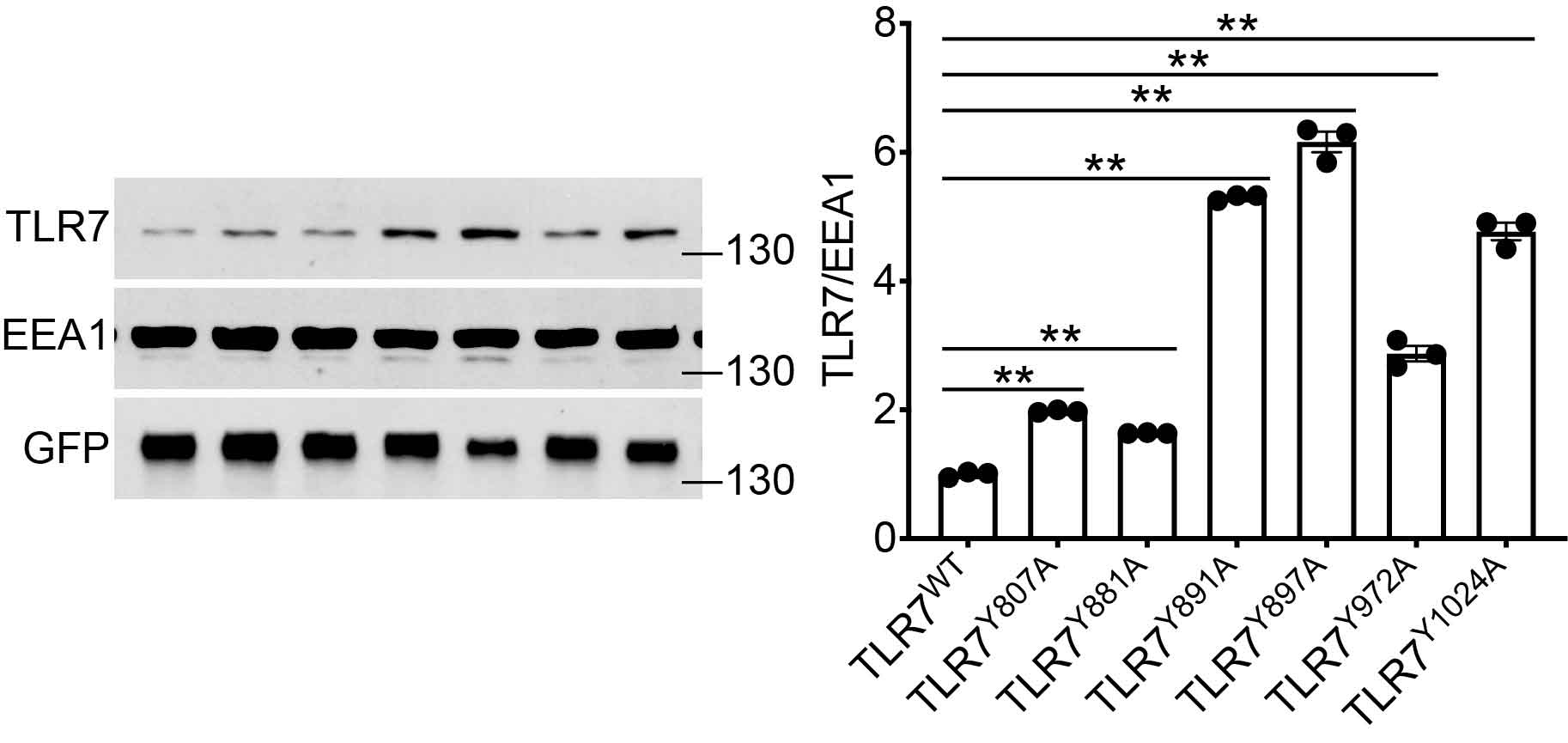

Supplement: Supplementary file 10 — Source Data for Figure 8 [file EMMM-14-e14455-s012.zip › Figure_8/8_B/8_B.jpg]

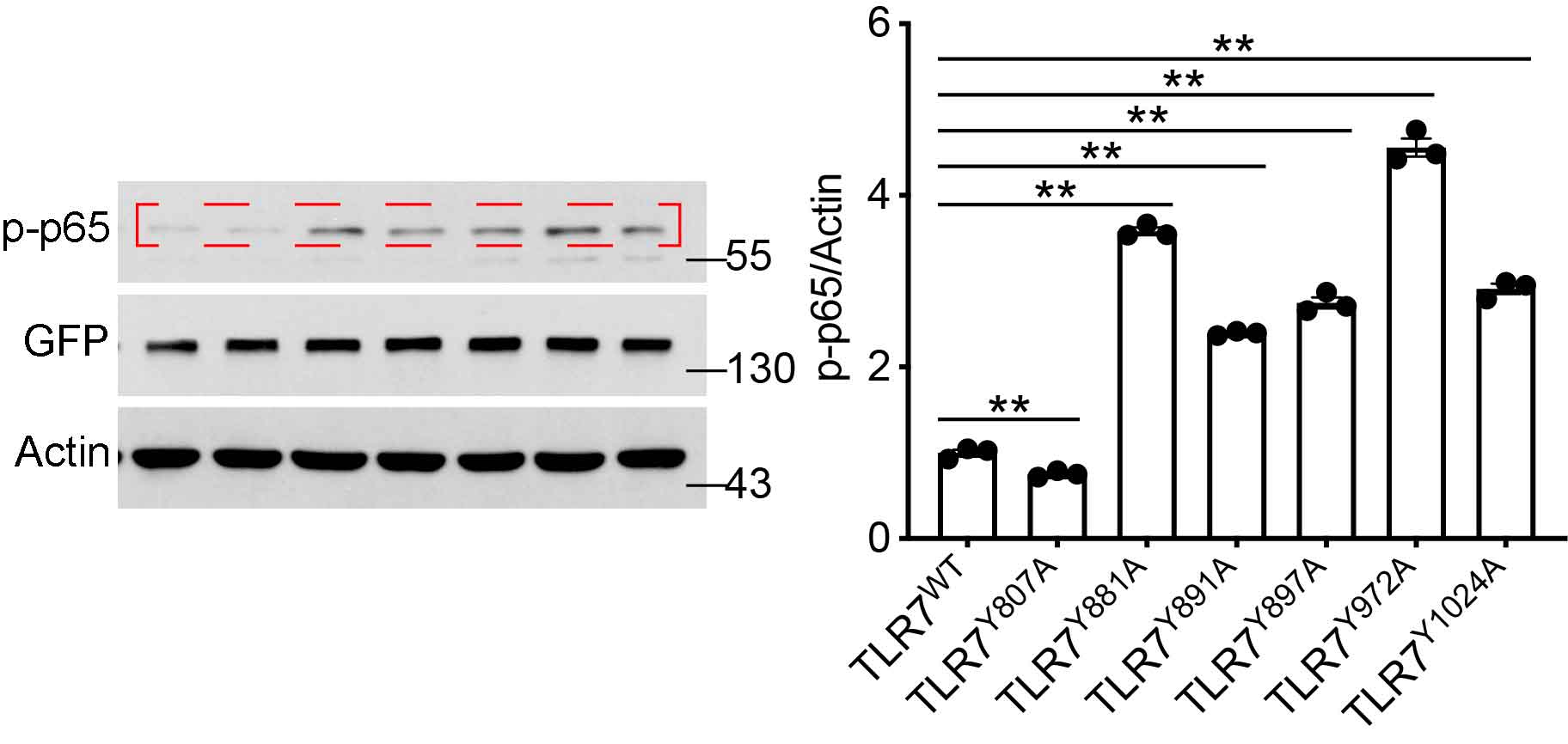

Supplement: Supplementary file 10 — Source Data for Figure 8 [file EMMM-14-e14455-s012.zip › Figure_8/8_C/8_C.jpg]

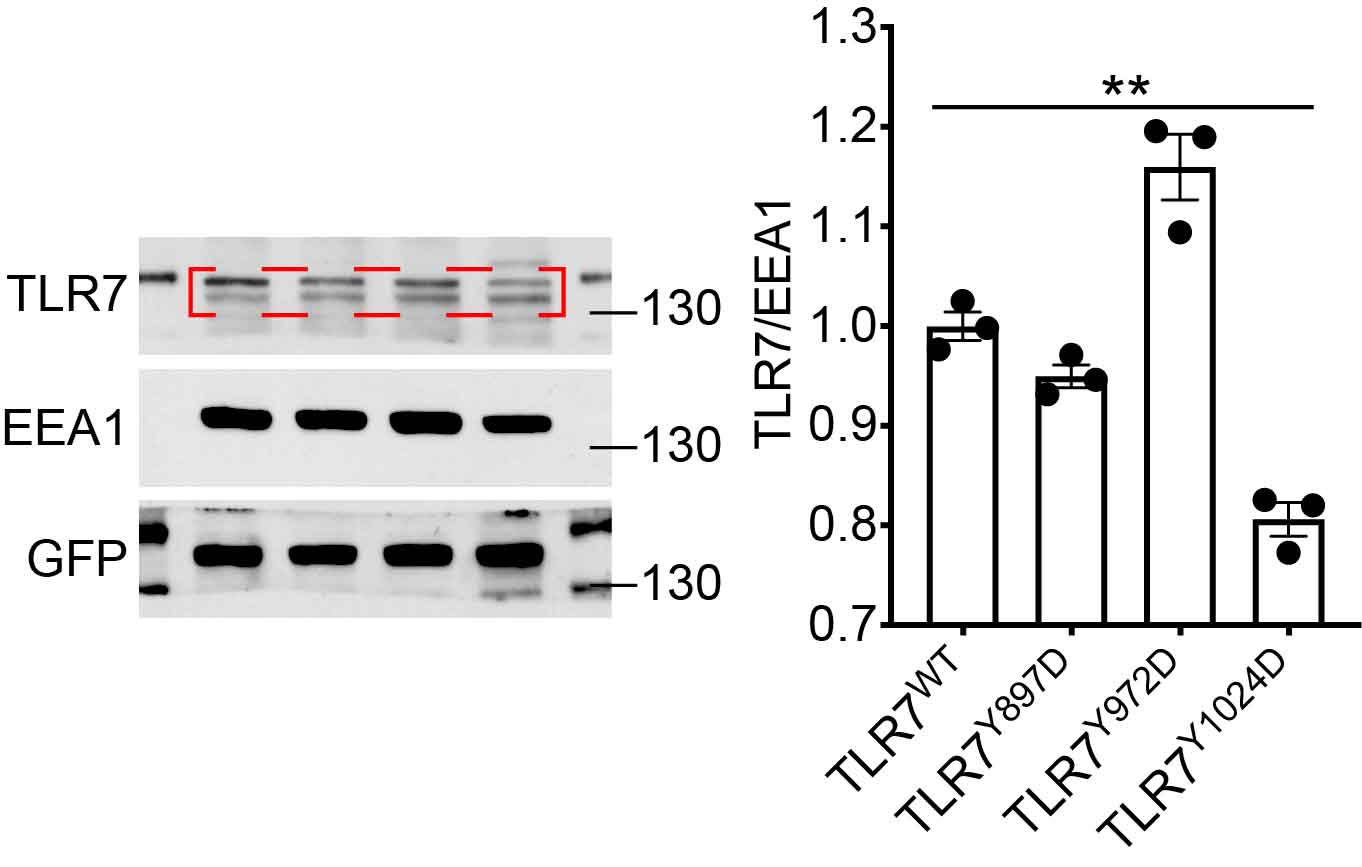

Supplement: Supplementary file 10 — Source Data for Figure 8 [file EMMM-14-e14455-s012.zip › Figure_8/8_E/8_E.jpg]

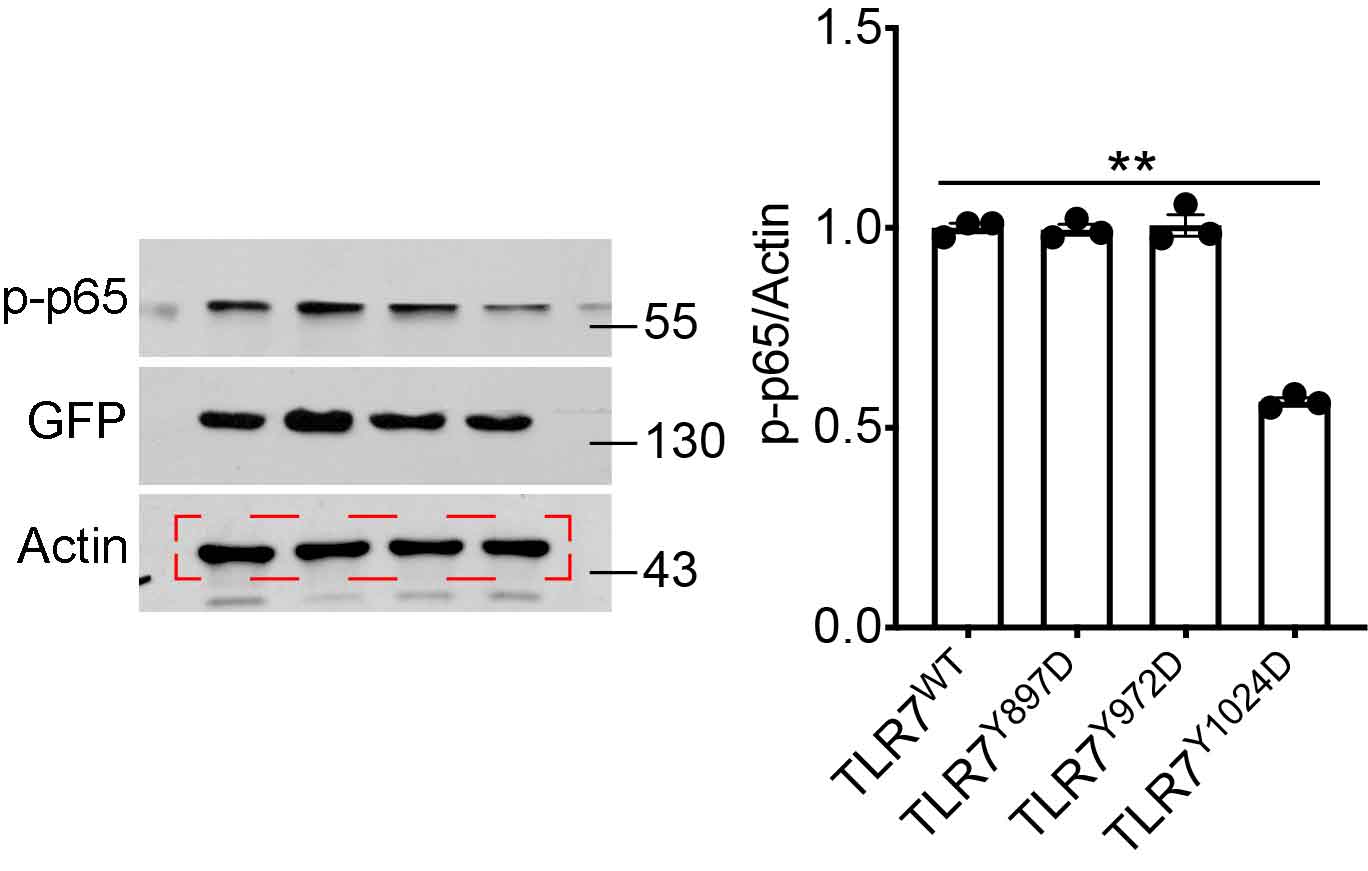

Supplement: Supplementary file 10 — Source Data for Figure 8 [file EMMM-14-e14455-s012.zip › Figure_8/8_F/8_F.jpg]

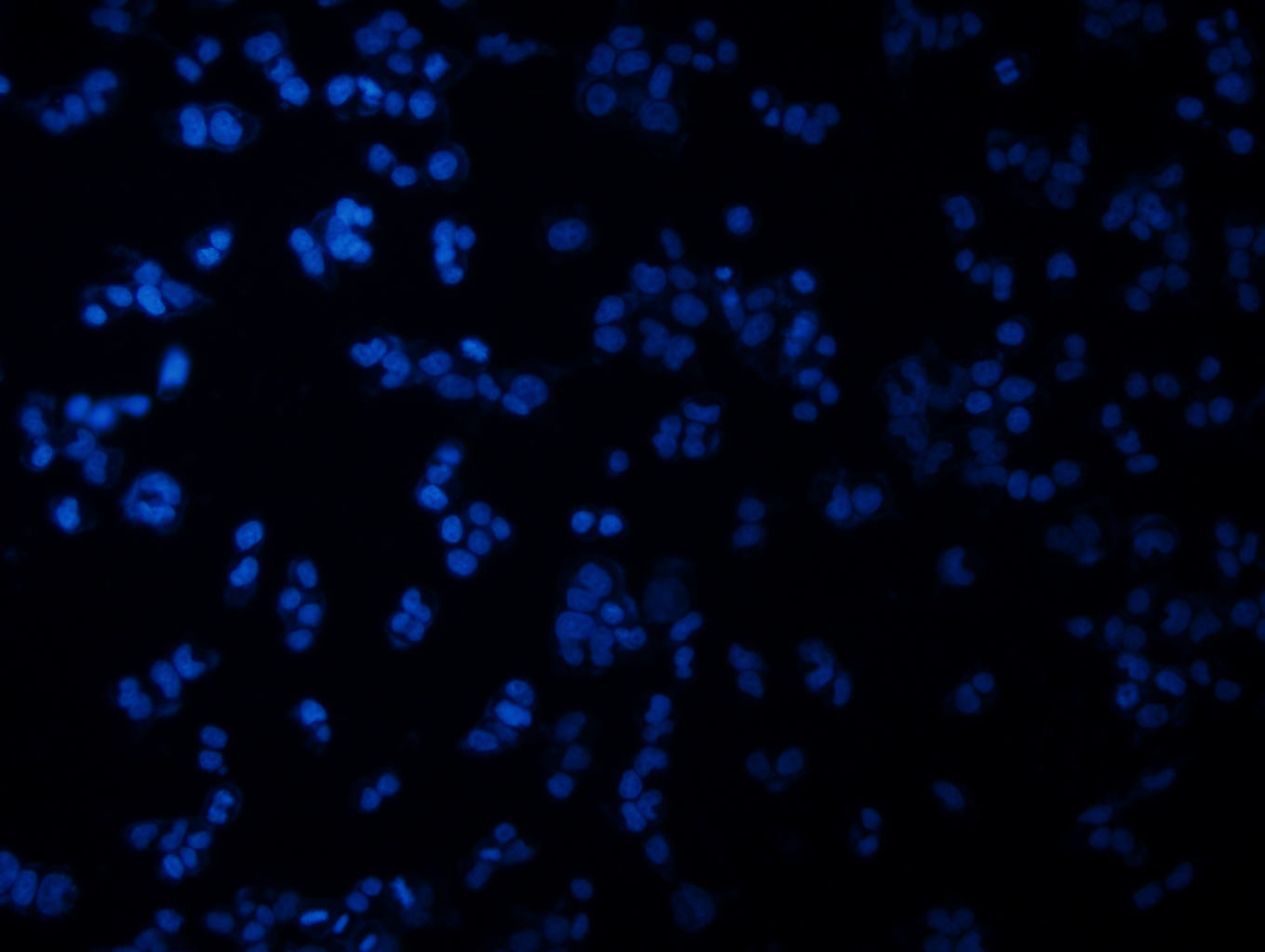

Supplement: Supplementary file 10 — Source Data for Figure 8 [file EMMM-14-e14455-s012.zip › Figure_8/8_H/TLR7_R848/DAPI-blank_R848.tif]

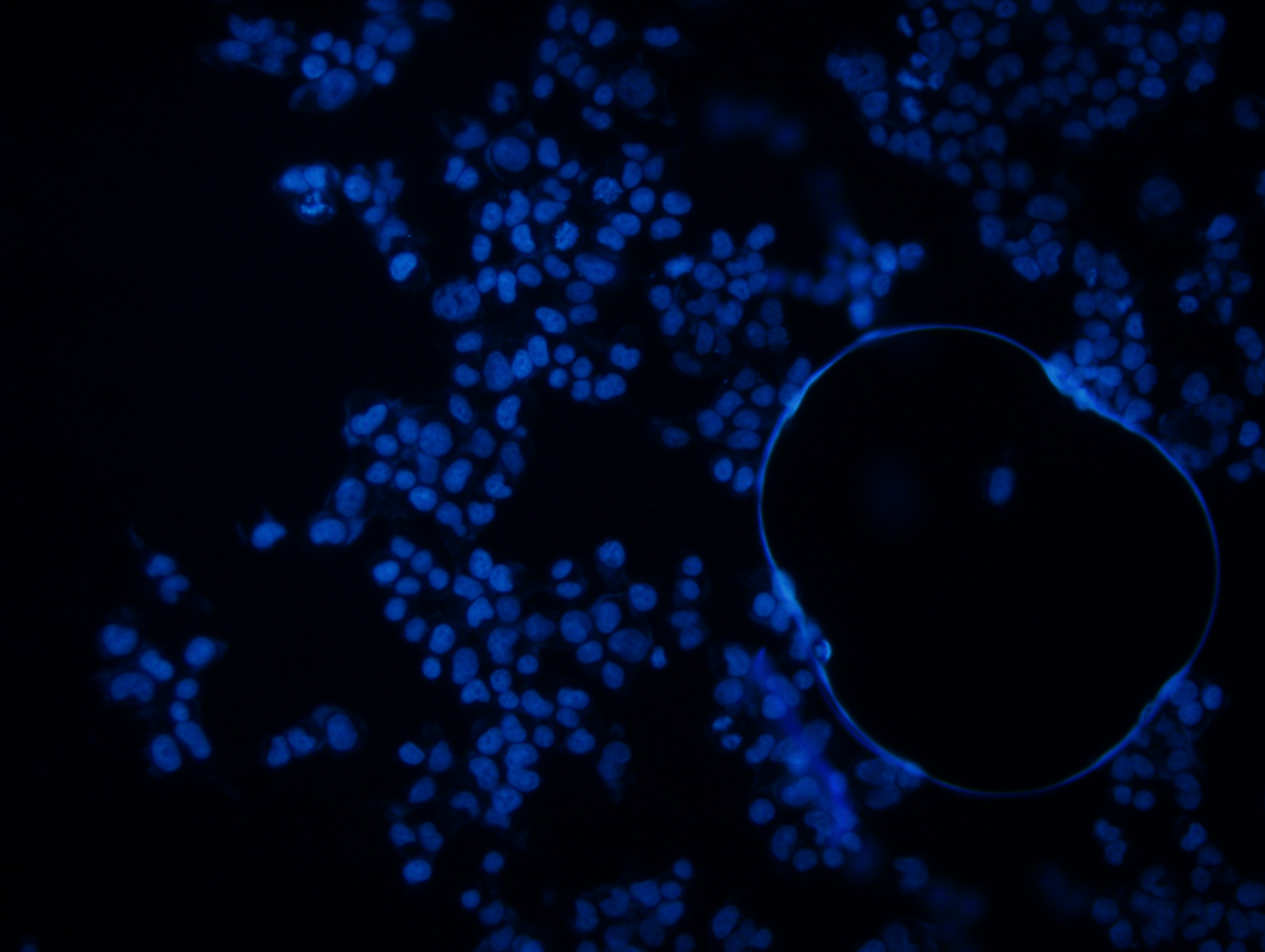

Supplement: Supplementary file 10 — Source Data for Figure 8 [file EMMM-14-e14455-s012.zip › Figure_8/8_H/TLR7_R848/DAPI-TLR7_R848.tif]

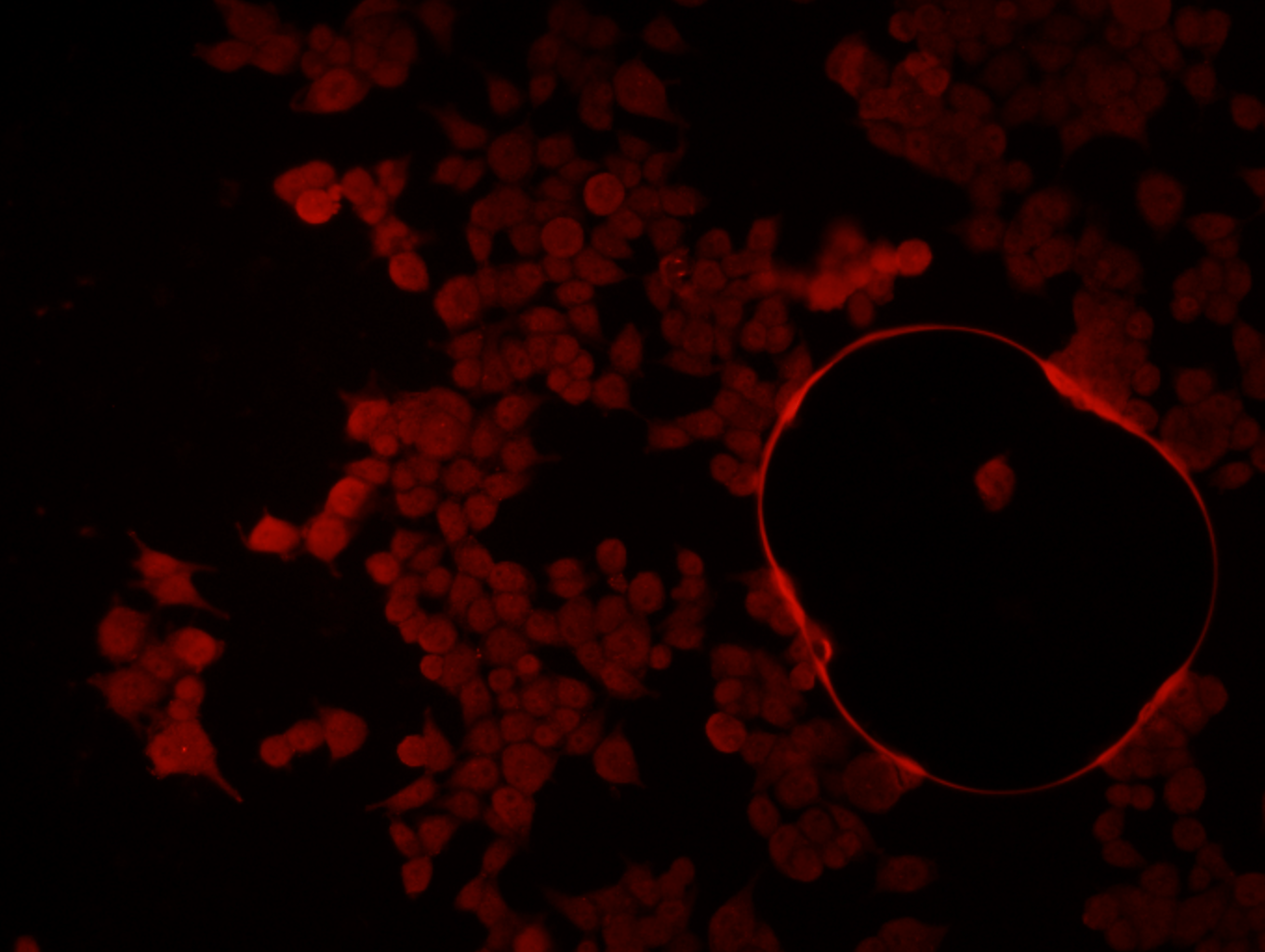

Supplement: Supplementary file 10 — Source Data for Figure 8 [file EMMM-14-e14455-s012.zip › Figure_8/8_H/TLR7_R848/P-P65-TLR7_R848.tif]

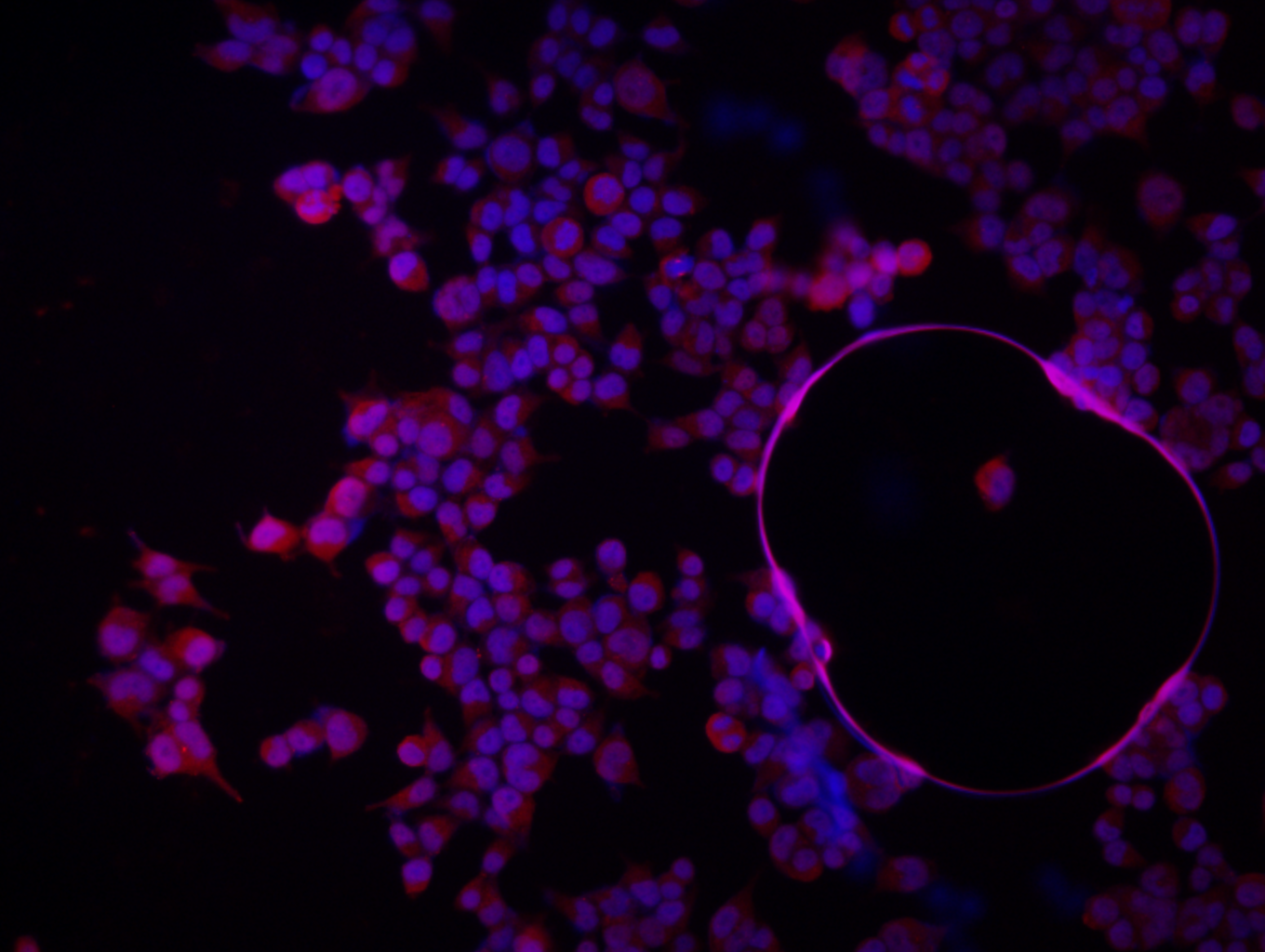

Supplement: Supplementary file 10 — Source Data for Figure 8 [file EMMM-14-e14455-s012.zip › Figure_8/8_H/TLR7_R848/TLR7_R848.tif]

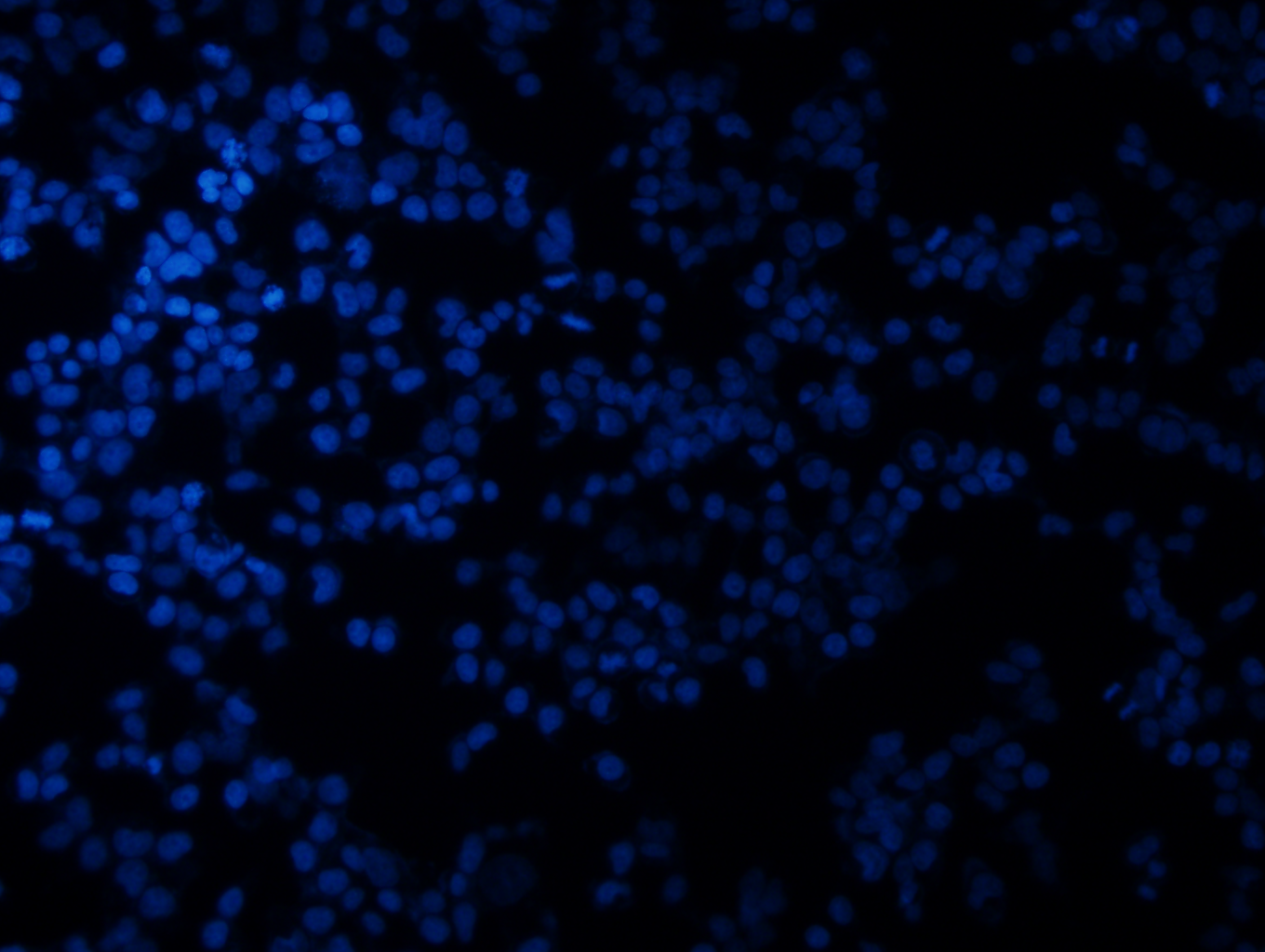

Supplement: Supplementary file 10 — Source Data for Figure 8 [file EMMM-14-e14455-s012.zip › Figure_8/8_H/Vector/DAPI-Vector.tif]

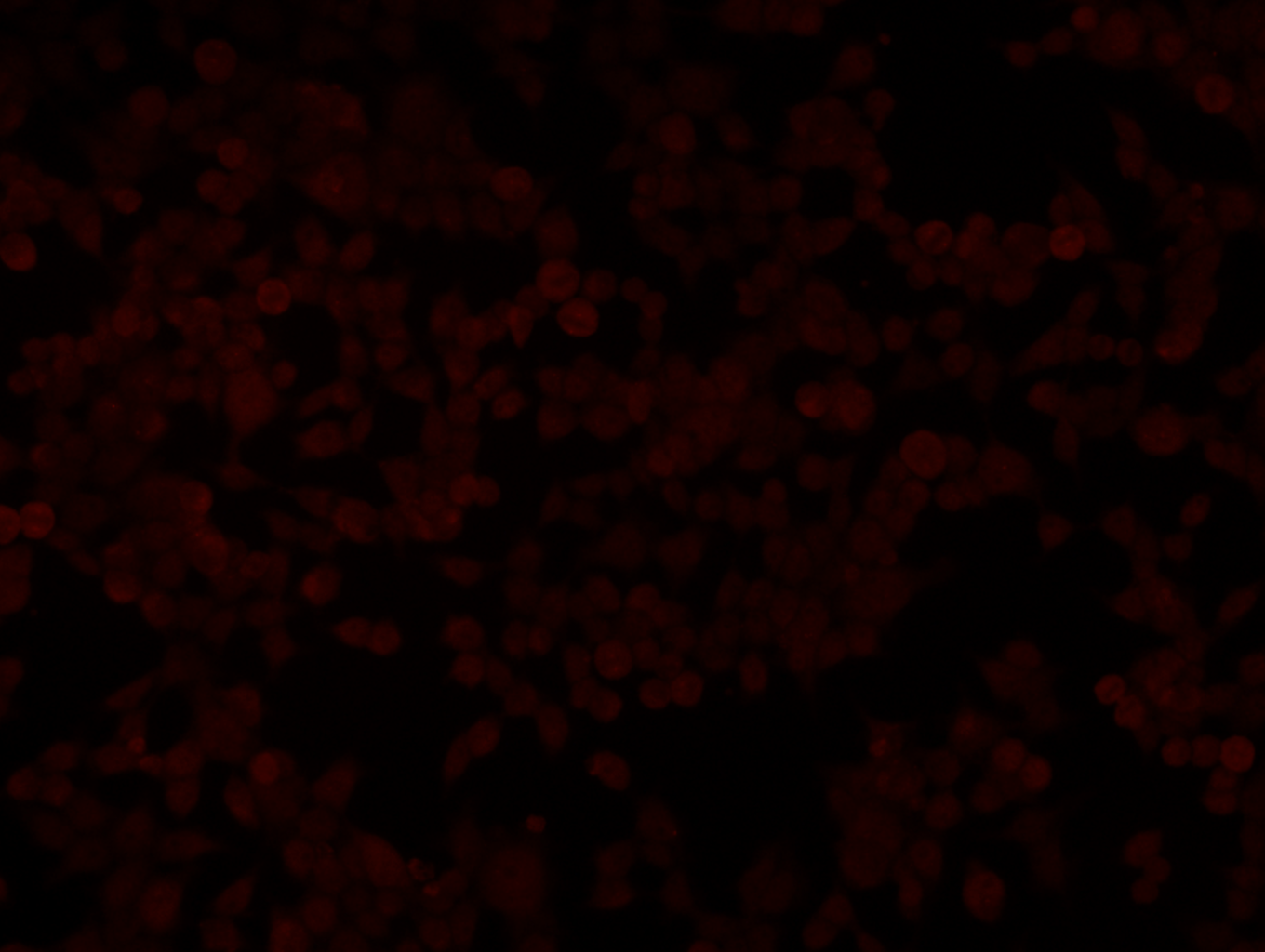

Supplement: Supplementary file 10 — Source Data for Figure 8 [file EMMM-14-e14455-s012.zip › Figure_8/8_H/Vector/P-P65-Vector.tif]

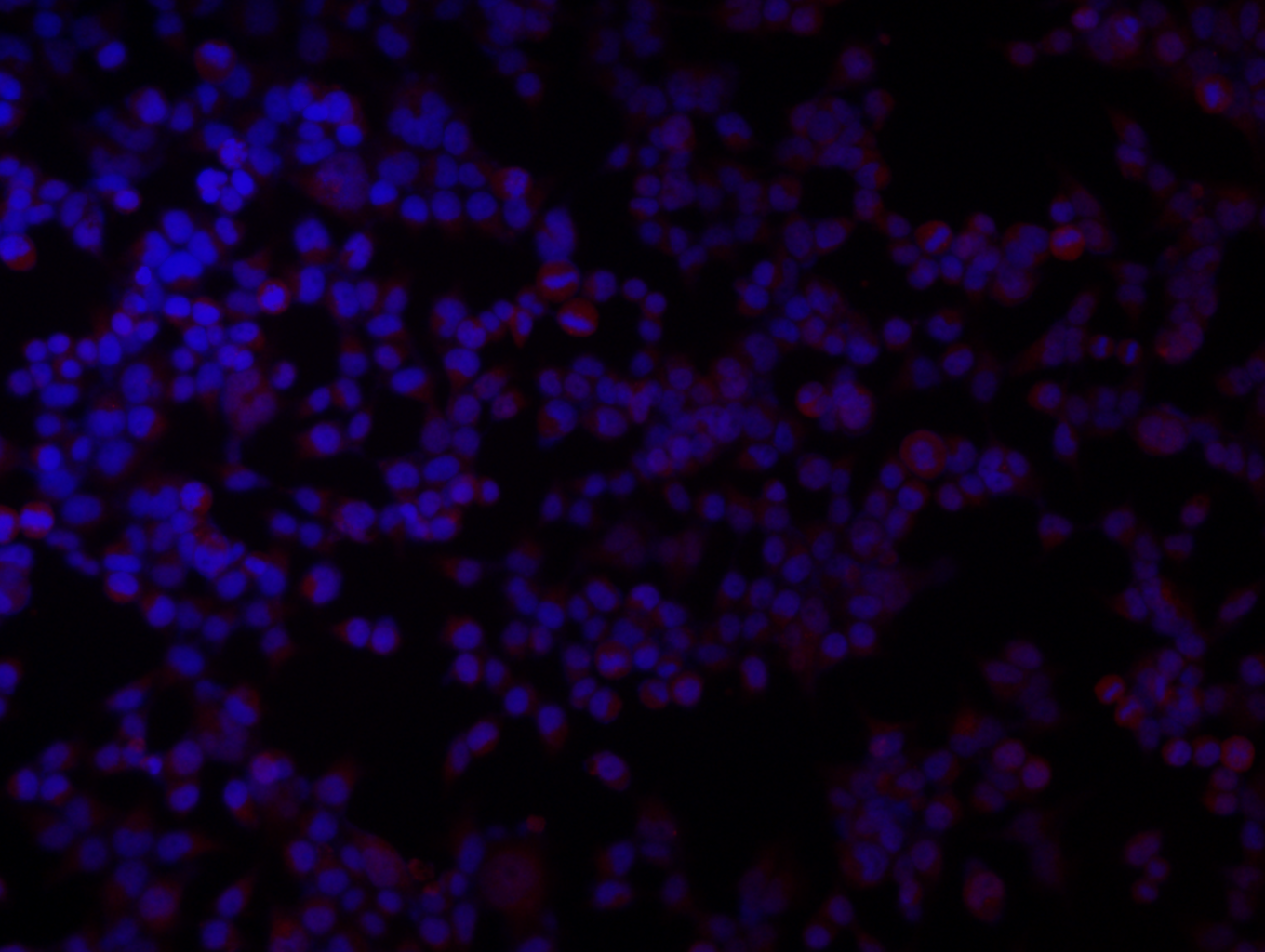

Supplement: Supplementary file 10 — Source Data for Figure 8 [file EMMM-14-e14455-s012.zip › Figure_8/8_H/Vector/Vector.tif]

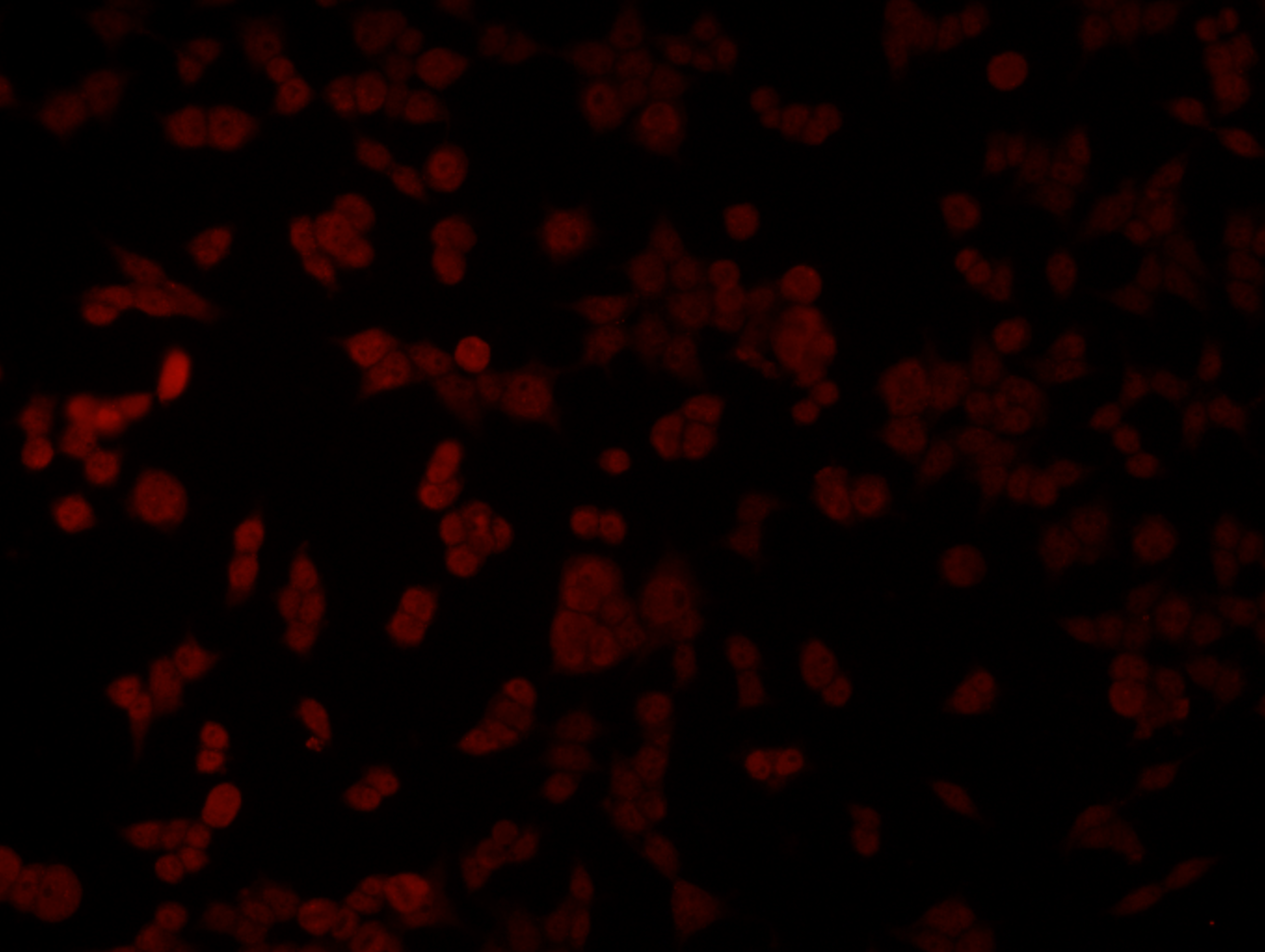

Supplement: Supplementary file 10 — Source Data for Figure 8 [file EMMM-14-e14455-s012.zip › Figure_8/8_H/Vector_R848/P-P65-Vector_R848.tif]

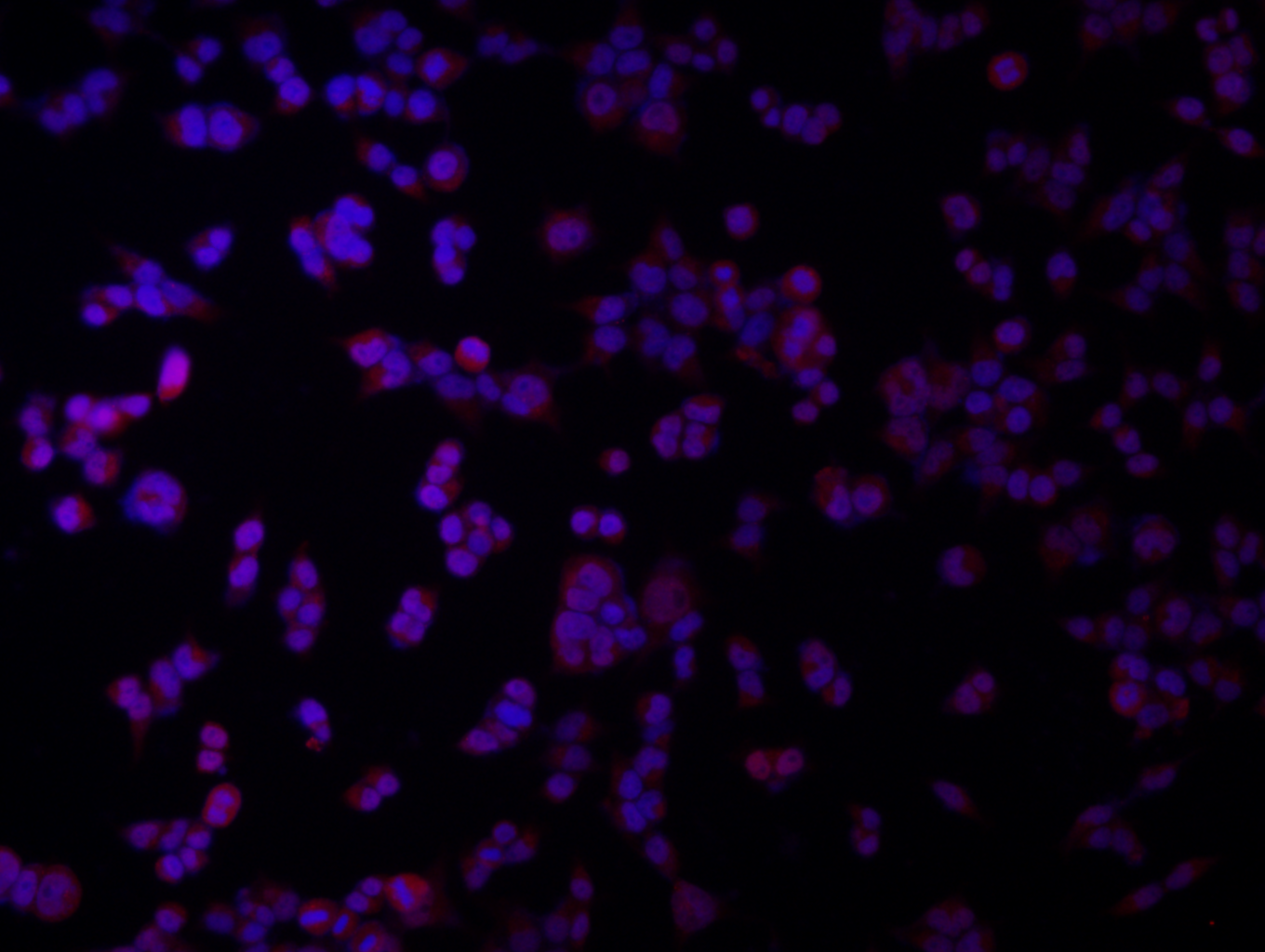

Supplement: Supplementary file 10 — Source Data for Figure 8 [file EMMM-14-e14455-s012.zip › Figure_8/8_H/Vector_R848/Vector_R848.tif]

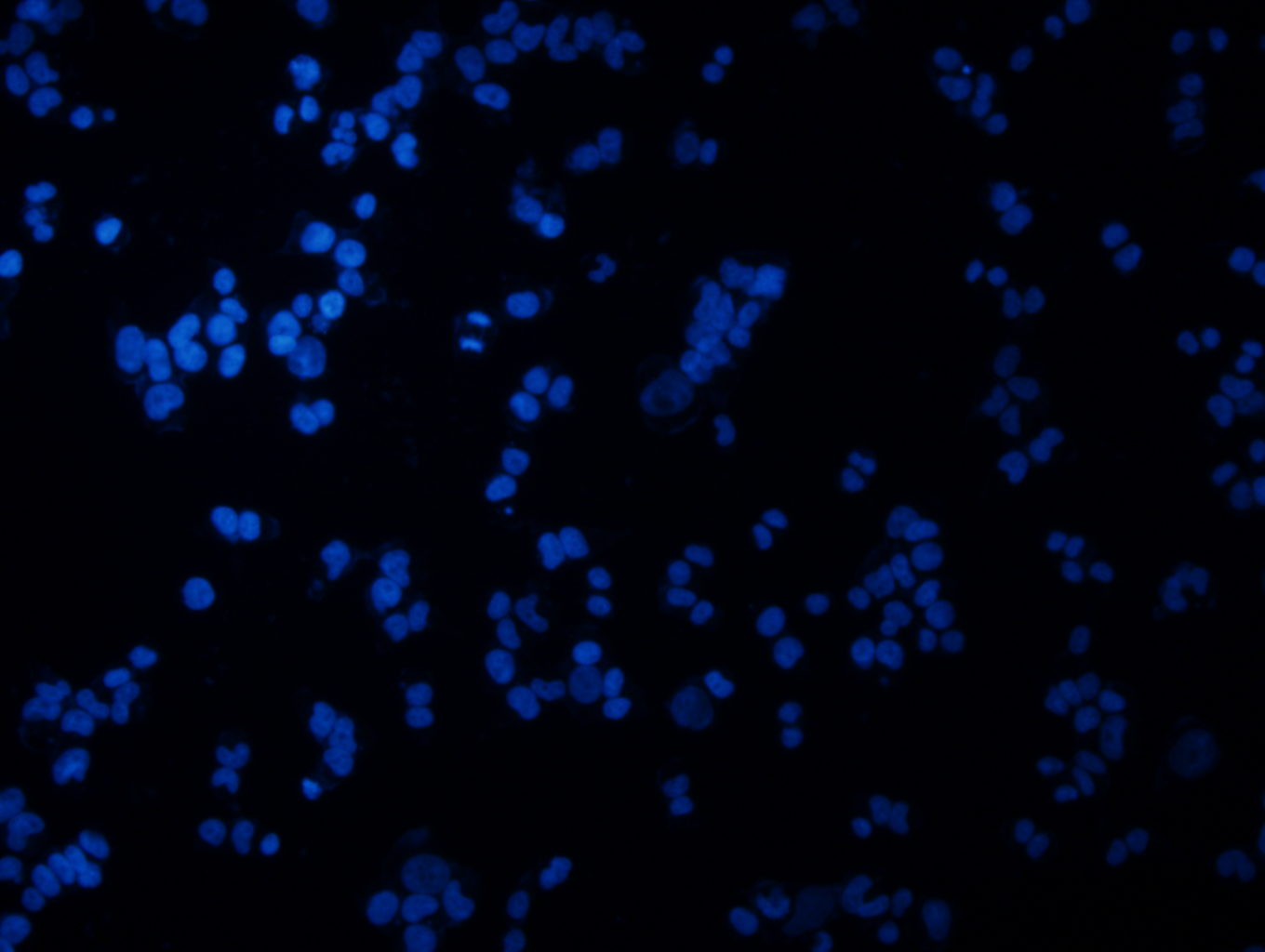

Supplement: Supplementary file 10 — Source Data for Figure 8 [file EMMM-14-e14455-s012.zip › Figure_8/8_H/Y1024A_R848/DAPI-Y1024A_R848.tif]

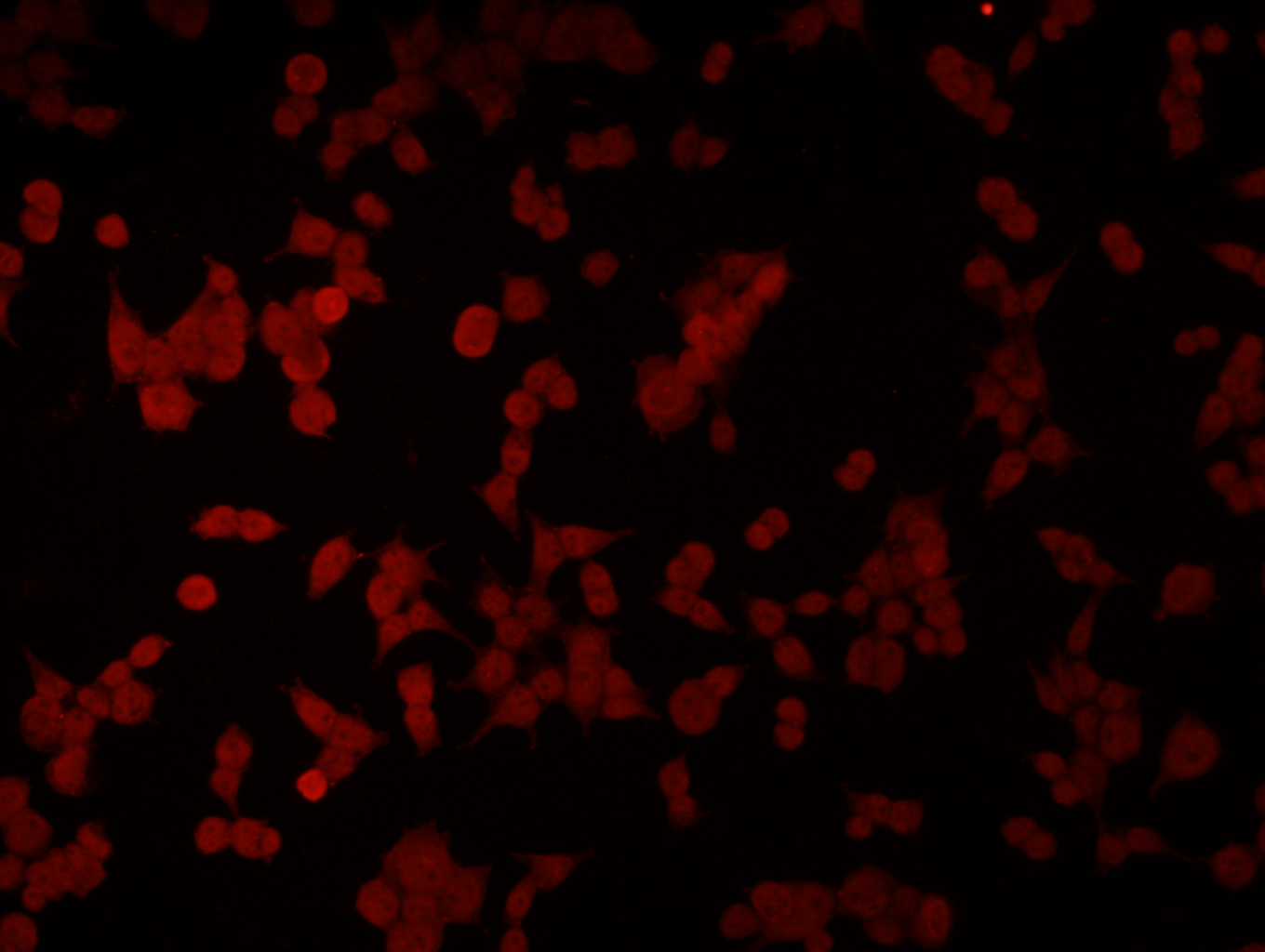

Supplement: Supplementary file 10 — Source Data for Figure 8 [file EMMM-14-e14455-s012.zip › Figure_8/8_H/Y1024A_R848/P-P65-Y1024A_R848.tif]

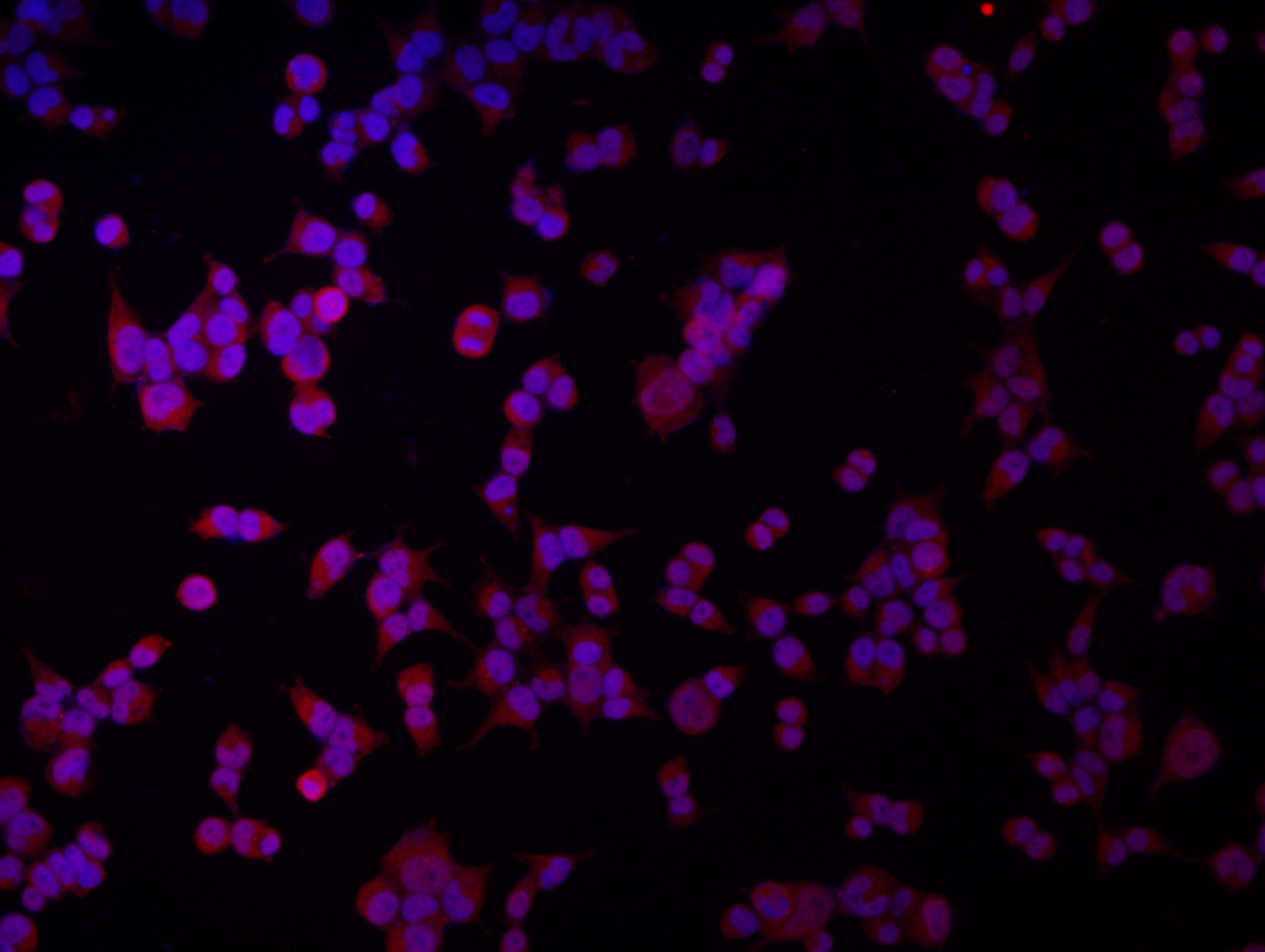

Supplement: Supplementary file 10 — Source Data for Figure 8 [file EMMM-14-e14455-s012.zip › Figure_8/8_H/Y1024A_R848/Y1024A_R848.tif]

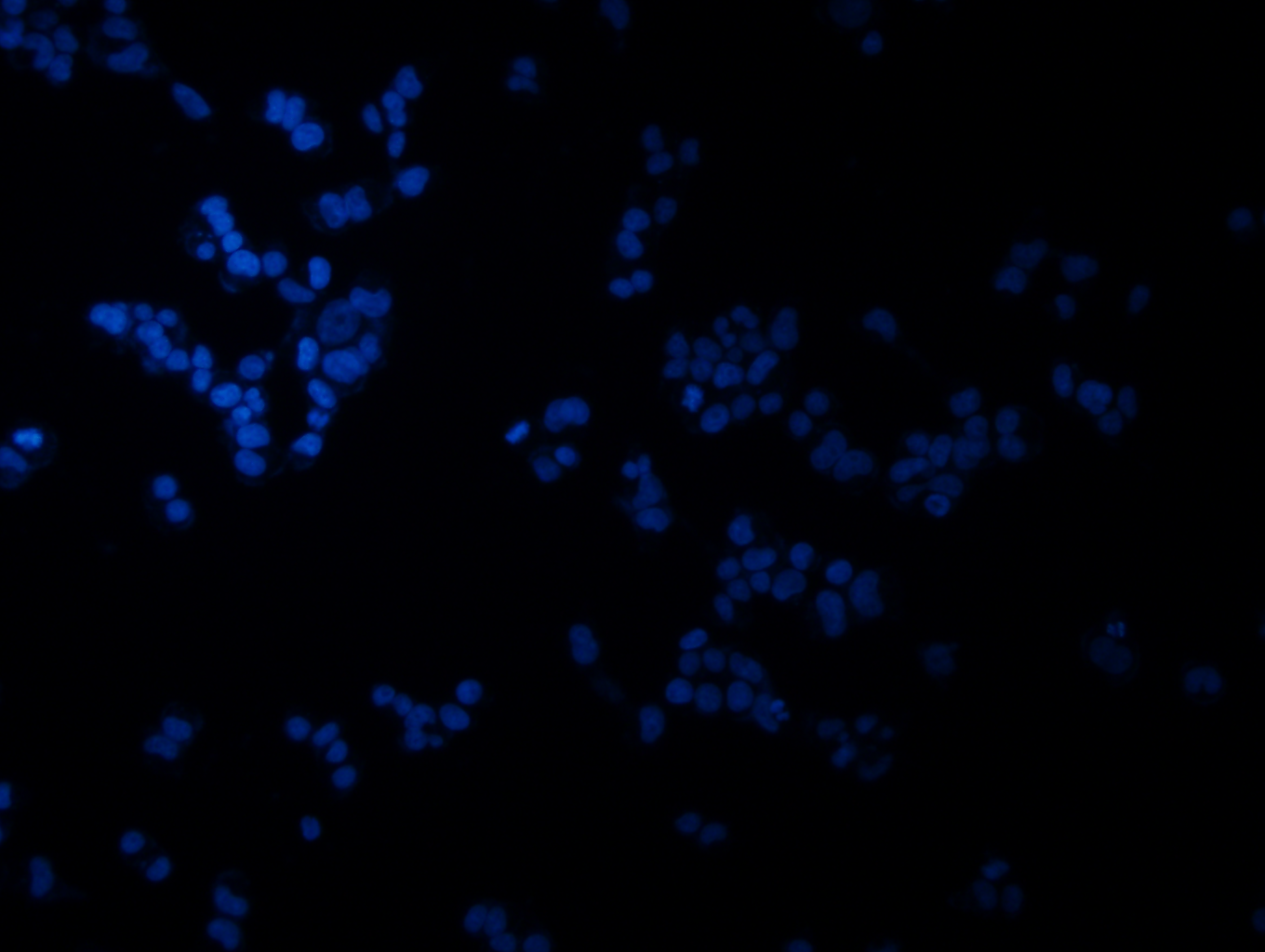

Supplement: Supplementary file 10 — Source Data for Figure 8 [file EMMM-14-e14455-s012.zip › Figure_8/8_H/Y1024D_R848/DAPI-Y1024D_R848.tif]

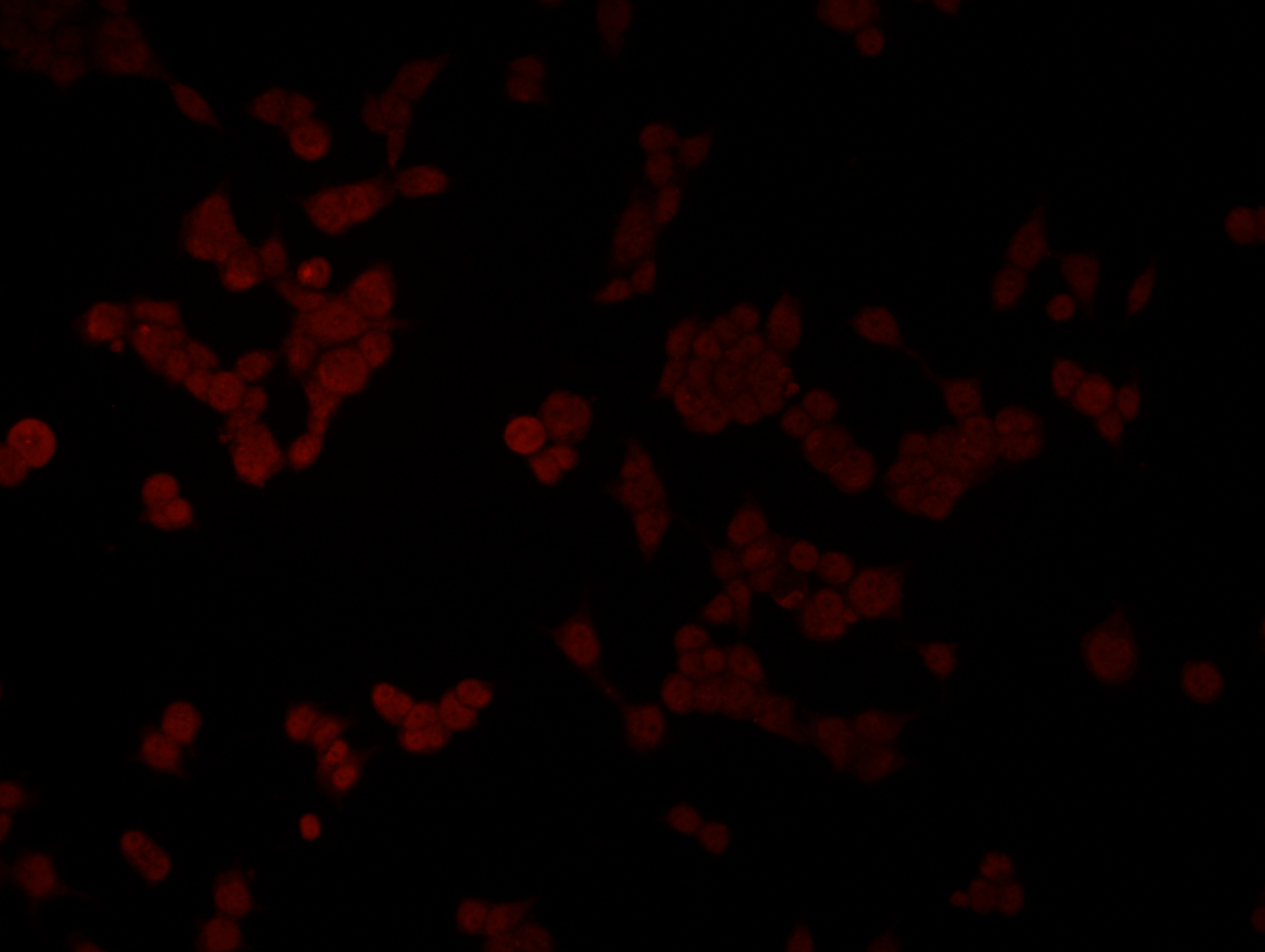

Supplement: Supplementary file 10 — Source Data for Figure 8 [file EMMM-14-e14455-s012.zip › Figure_8/8_H/Y1024D_R848/P-P65-Y1024D_R848.tif]

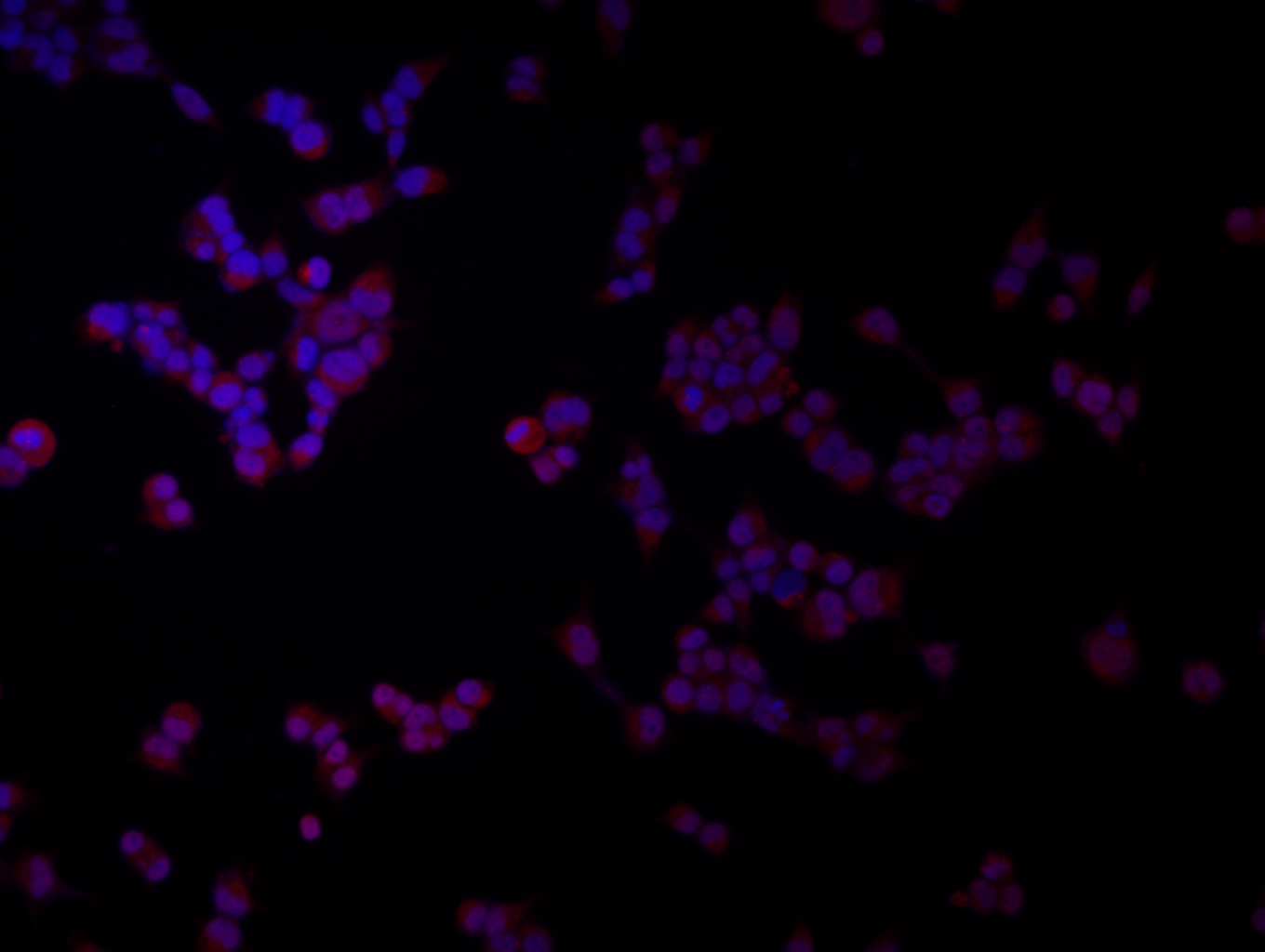

Supplement: Supplementary file 10 — Source Data for Figure 8 [file EMMM-14-e14455-s012.zip › Figure_8/8_H/Y1024D_R848/Y1024D_R848.tif]

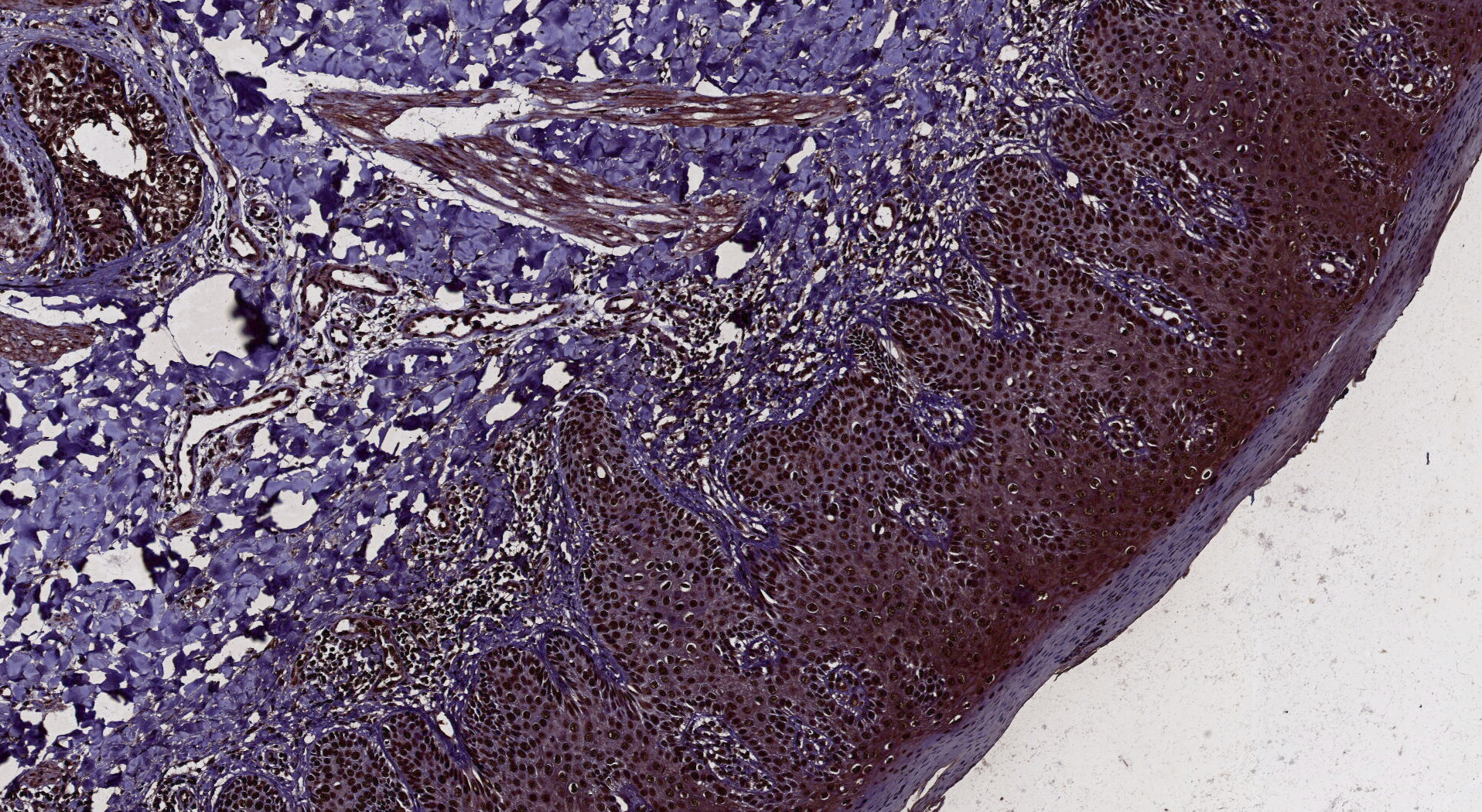

Supplement: Supplementary file 10 — Source Data for Figure 8 [file EMMM-14-e14455-s012.zip › Figure_8/8_I/20195056_1#.tiff]

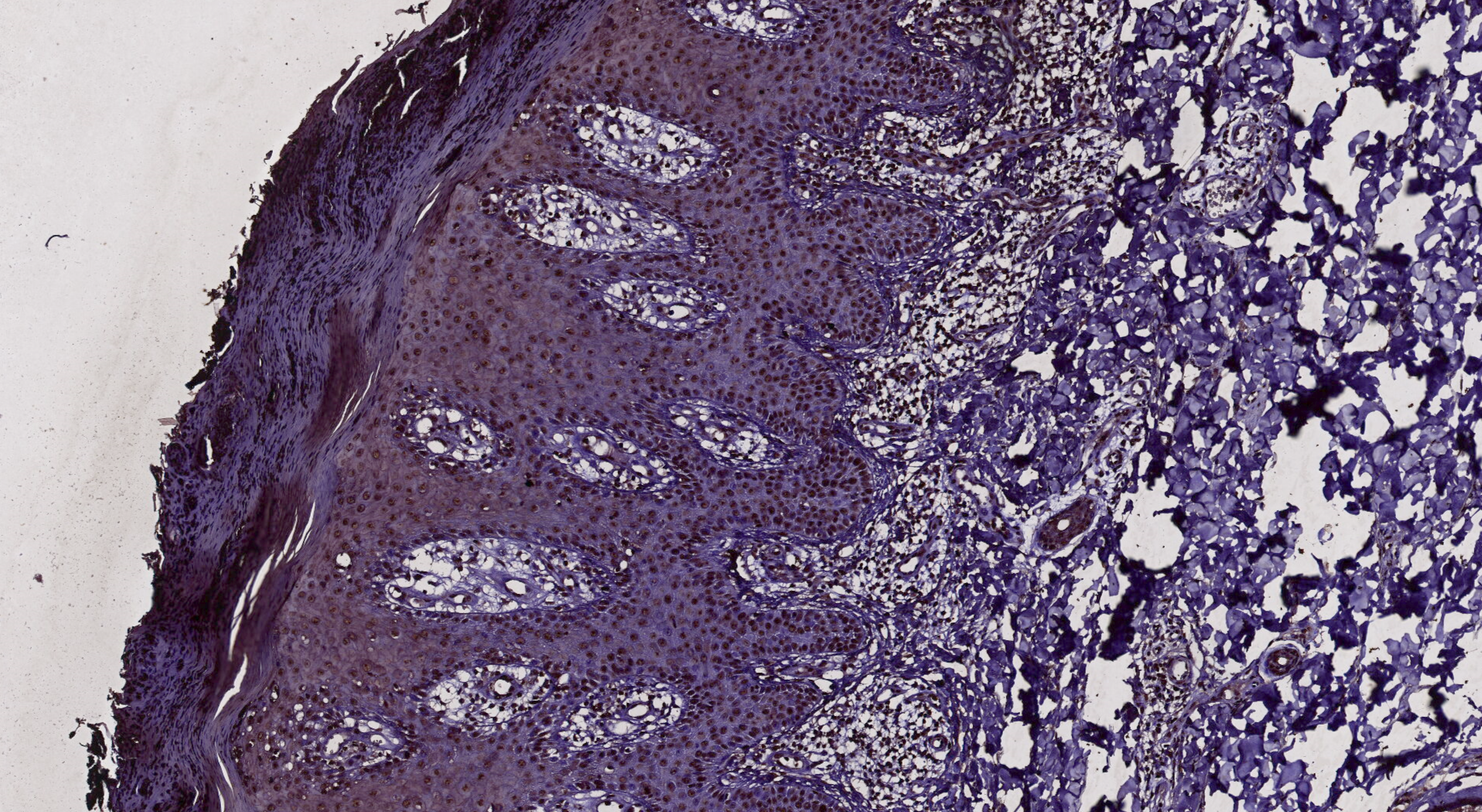

Supplement: Supplementary file 10 — Source Data for Figure 8 [file EMMM-14-e14455-s012.zip › Figure_8/8_I/20200197_2#.tiff]

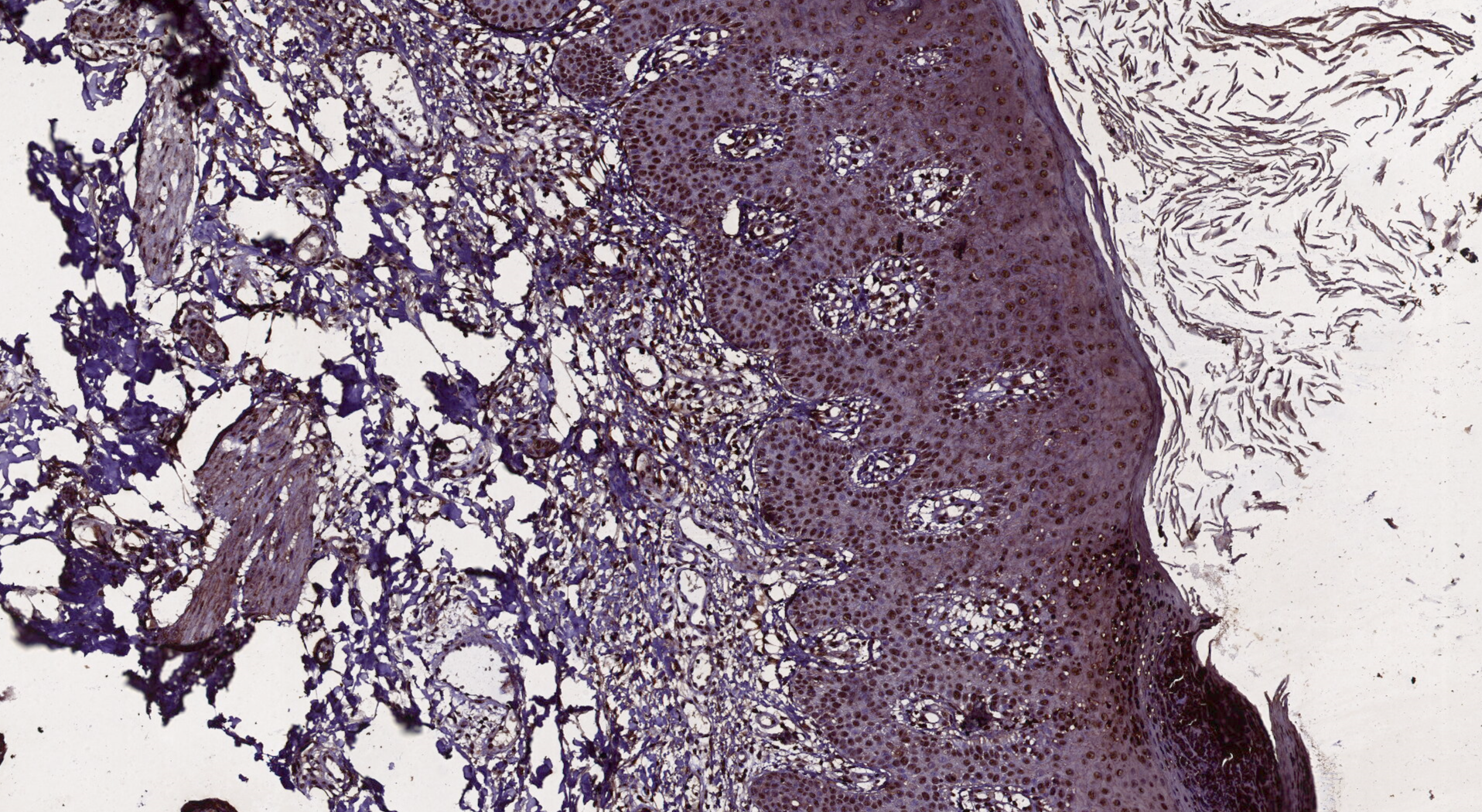

Supplement: Supplementary file 10 — Source Data for Figure 8 [file EMMM-14-e14455-s012.zip › Figure_8/8_I/20200230_3#.tiff]

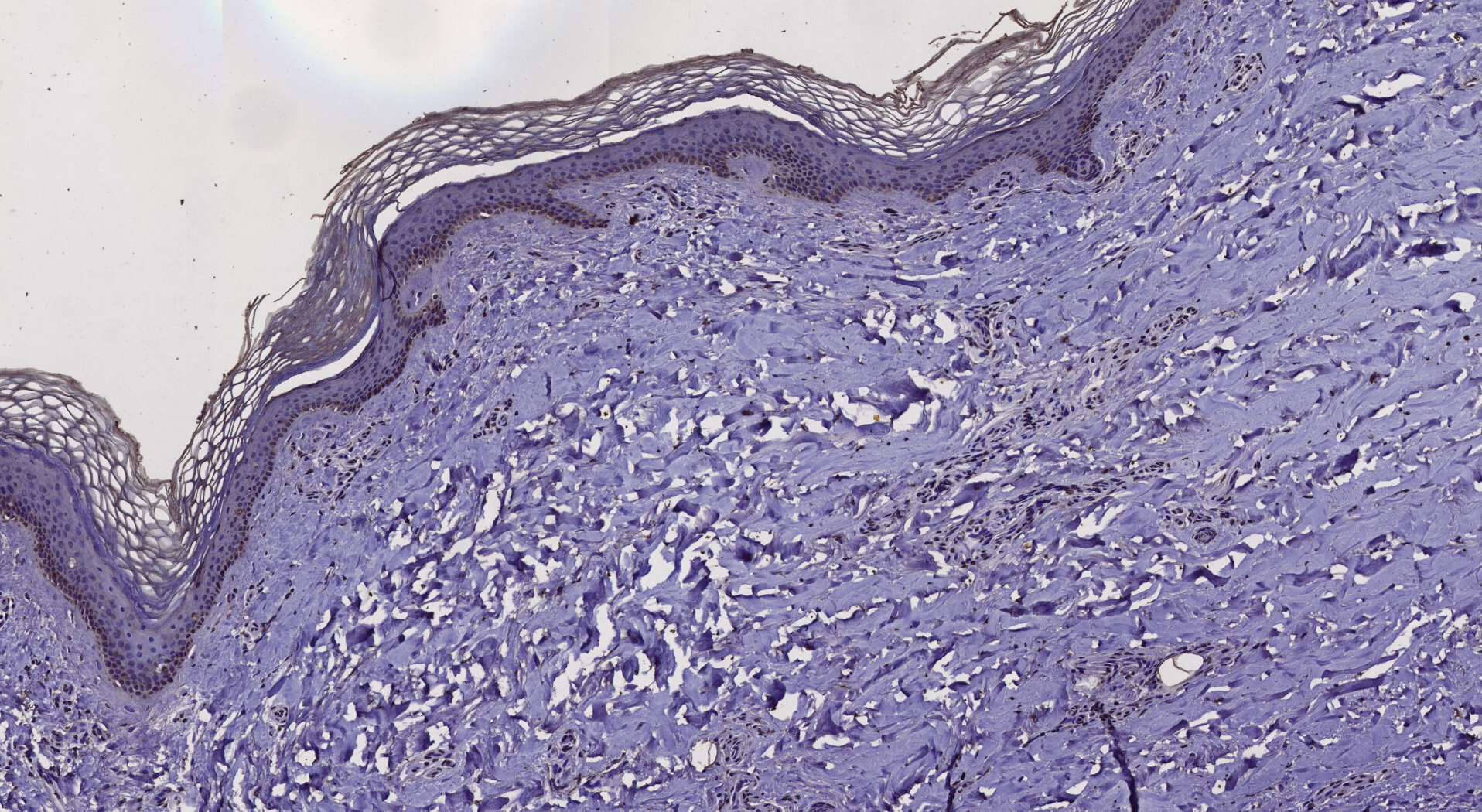

Supplement: Supplementary file 10 — Source Data for Figure 8 [file EMMM-14-e14455-s012.zip › Figure_8/8_I/Normal_1#.tiff]

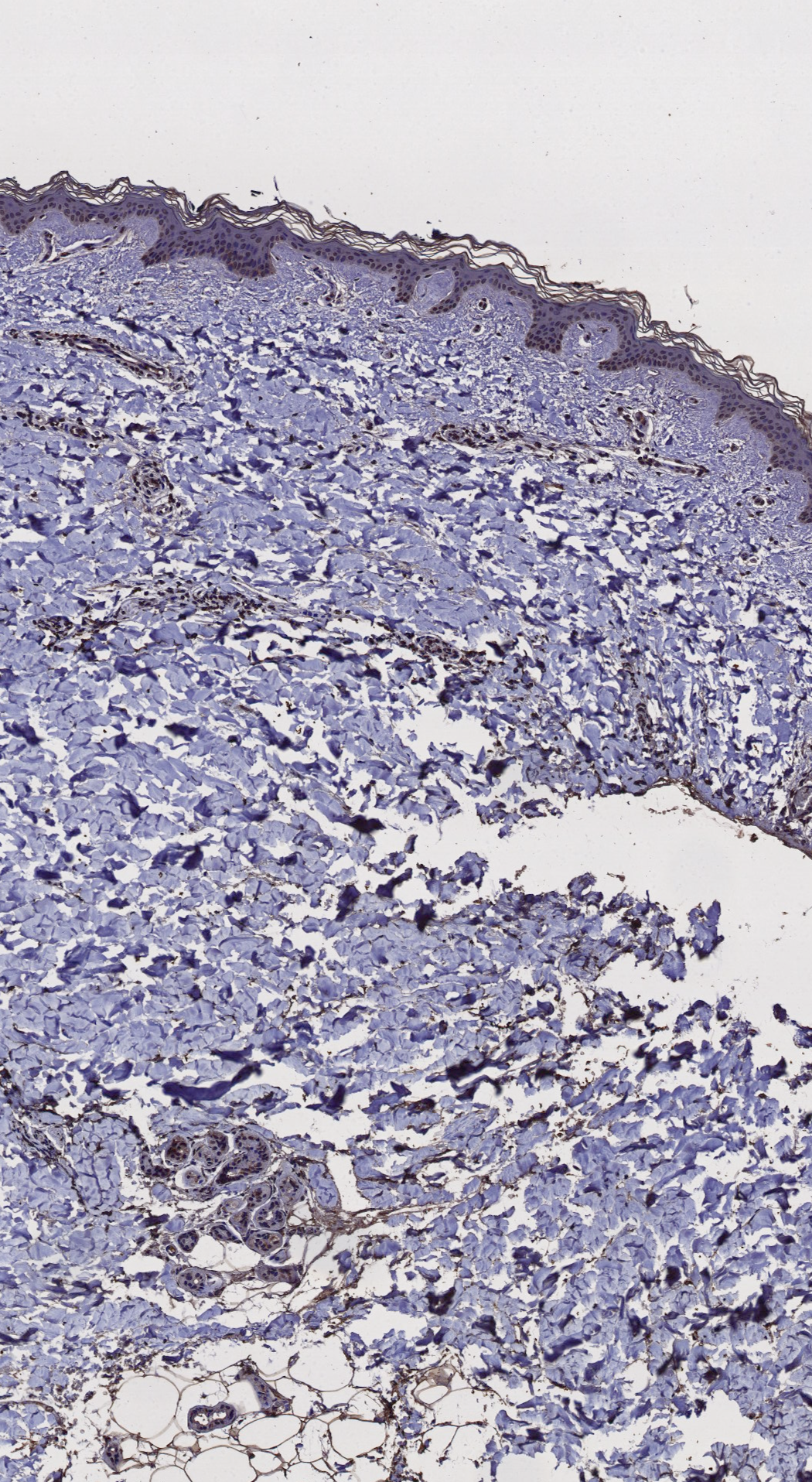

Supplement: Supplementary file 10 — Source Data for Figure 8 [file EMMM-14-e14455-s012.zip › Figure_8/8_I/Normal_2#.tiff]

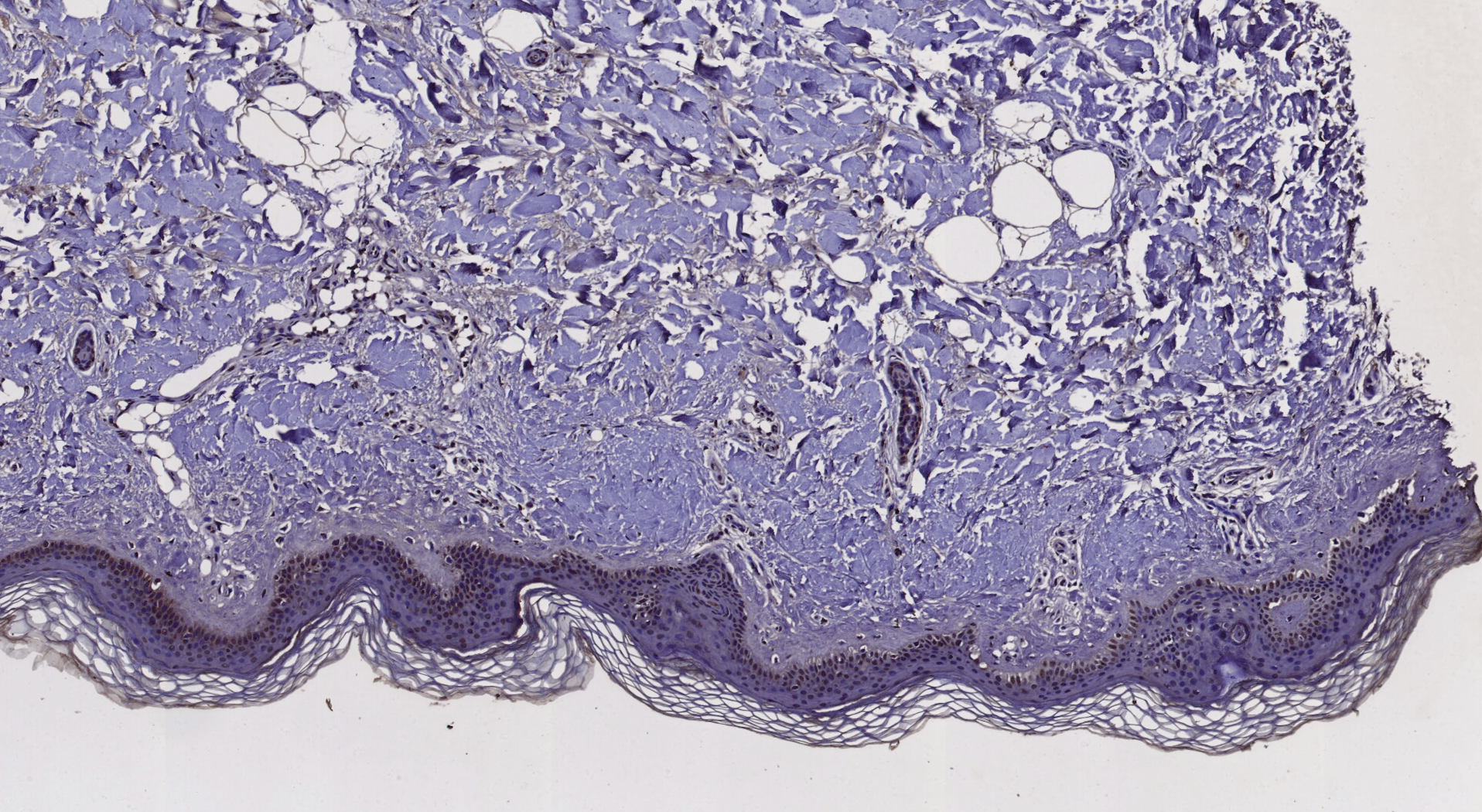

Supplement: Supplementary file 10 — Source Data for Figure 8 [file EMMM-14-e14455-s012.zip › Figure_8/8_I/Normal_3#.tiff]

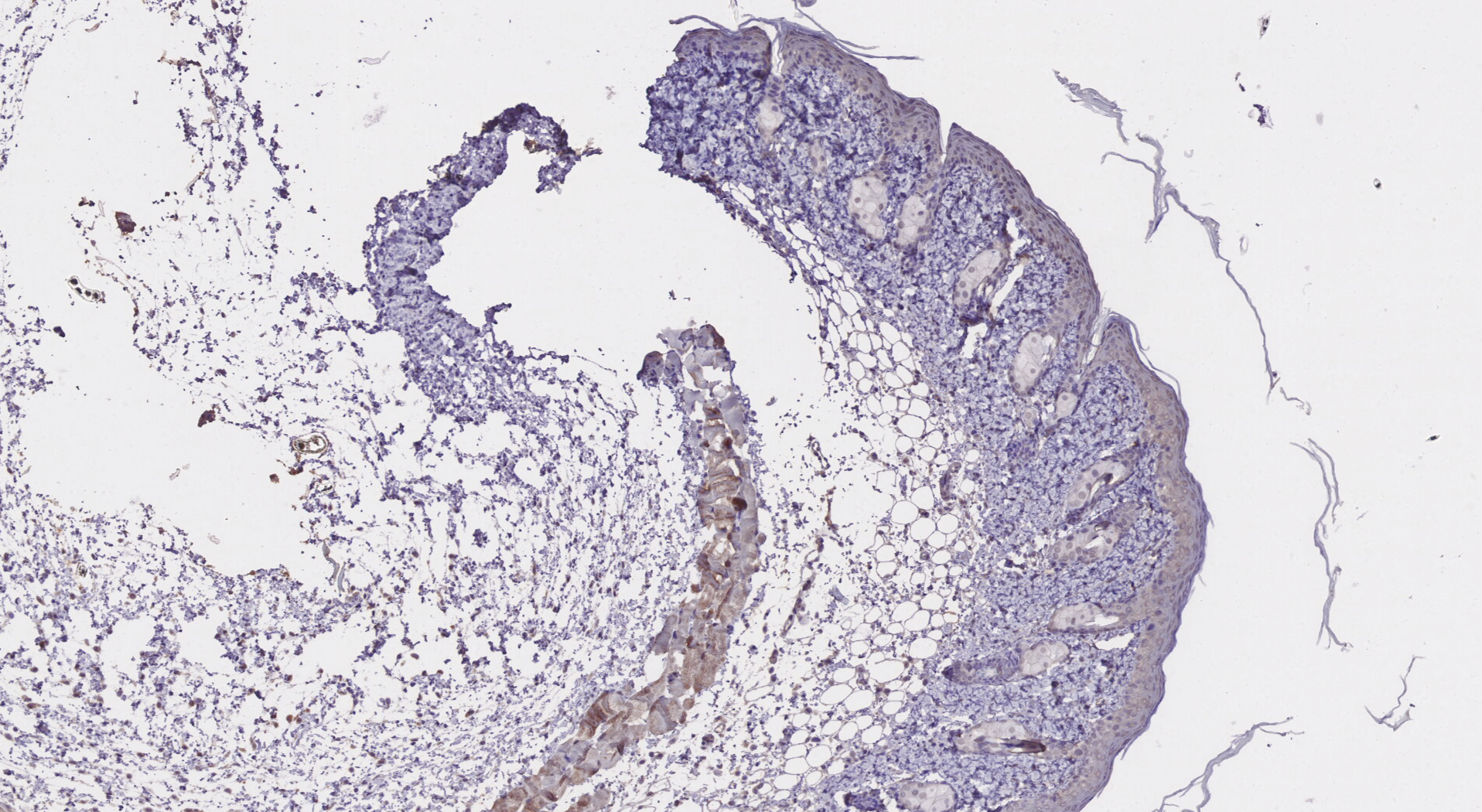

Supplement: Supplementary file 11 — Source Data for Figure 9 [file EMMM-14-e14455-s005.zip › Figure 9/9 E/KI:WT.tiff]

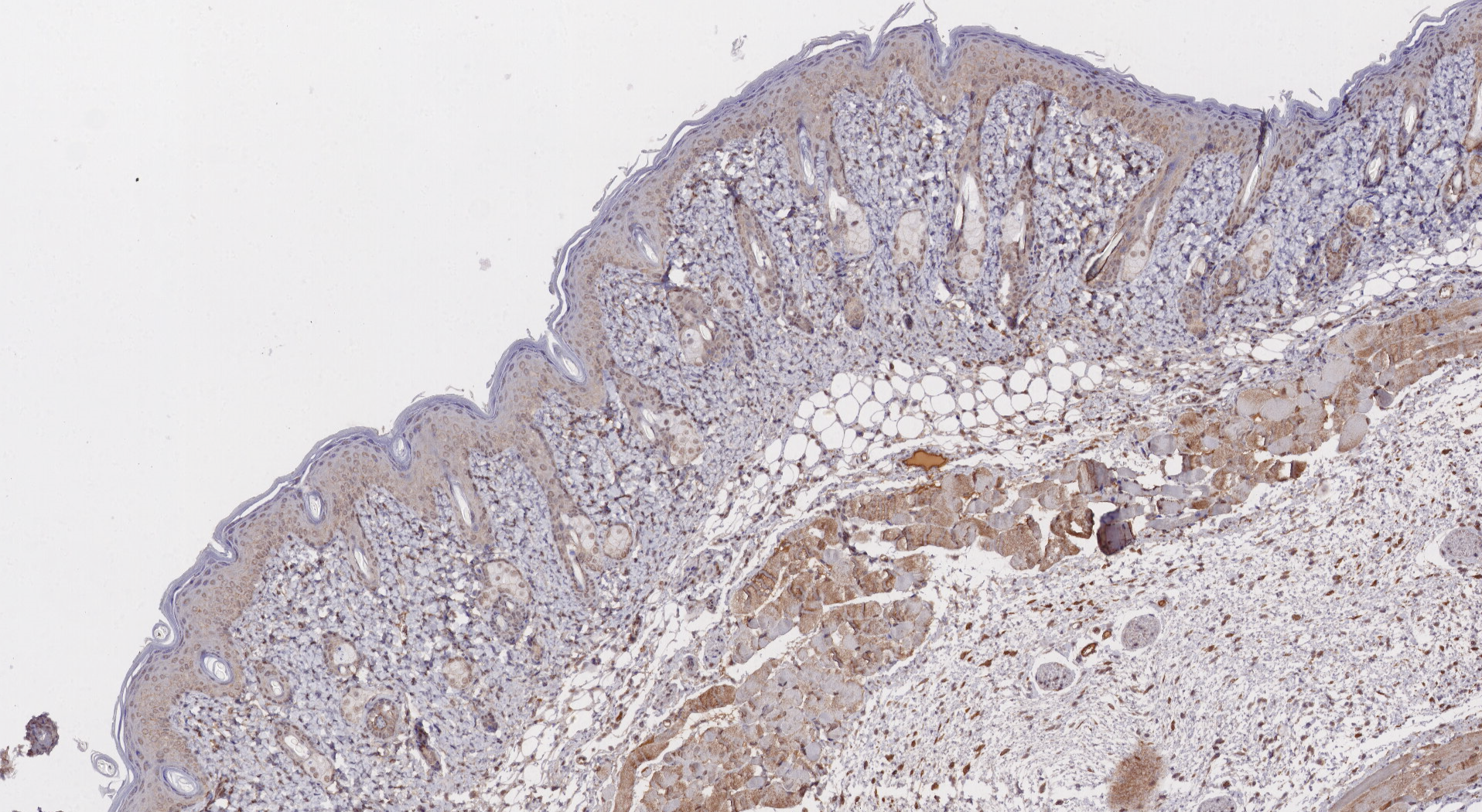

Supplement: Supplementary file 11 — Source Data for Figure 9 [file EMMM-14-e14455-s005.zip › Figure 9/9 E/WT.tiff]

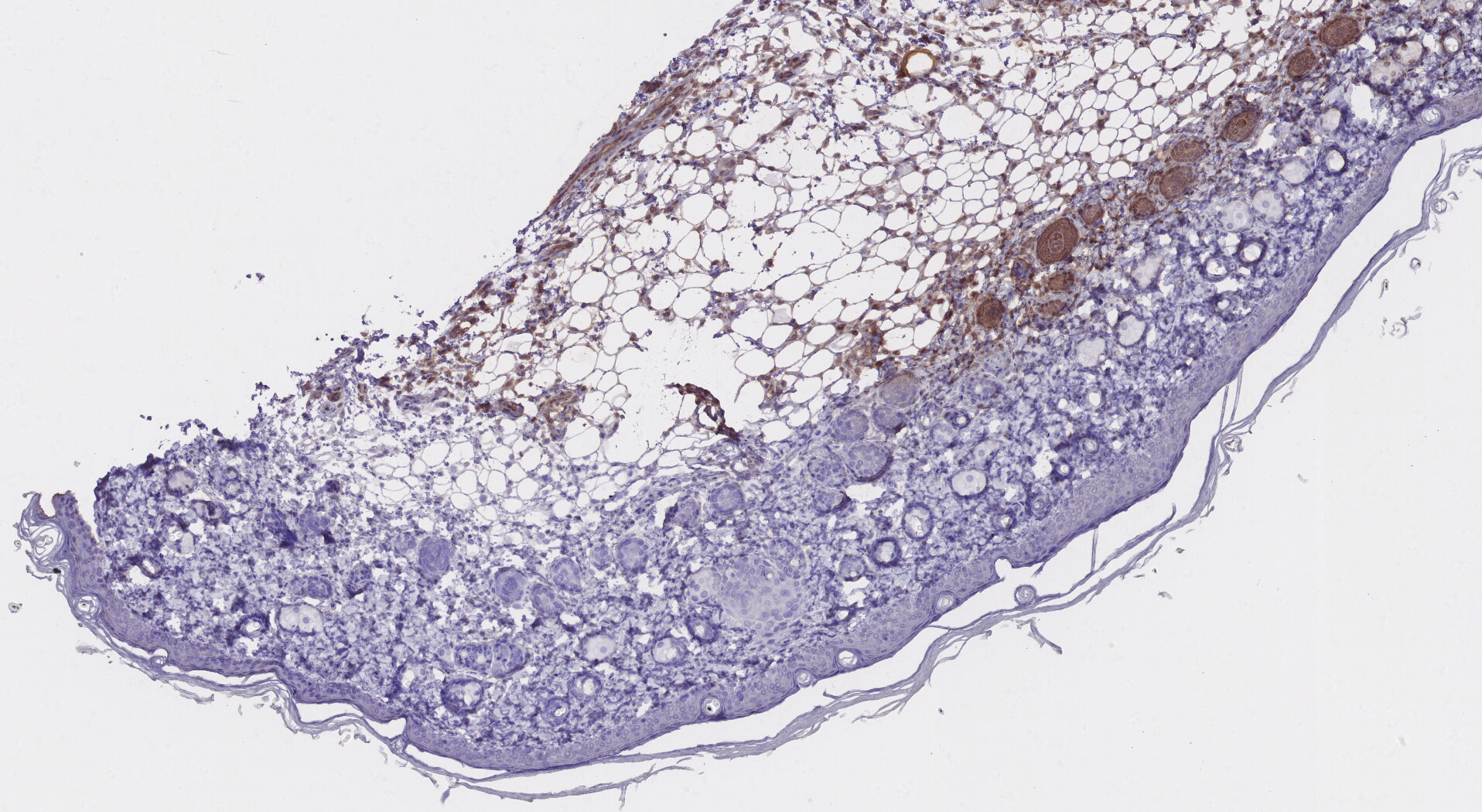

Supplement: Supplementary file 11 — Source Data for Figure 9 [file EMMM-14-e14455-s005.zip › Figure 9/9 E/KI:KI.tiff]

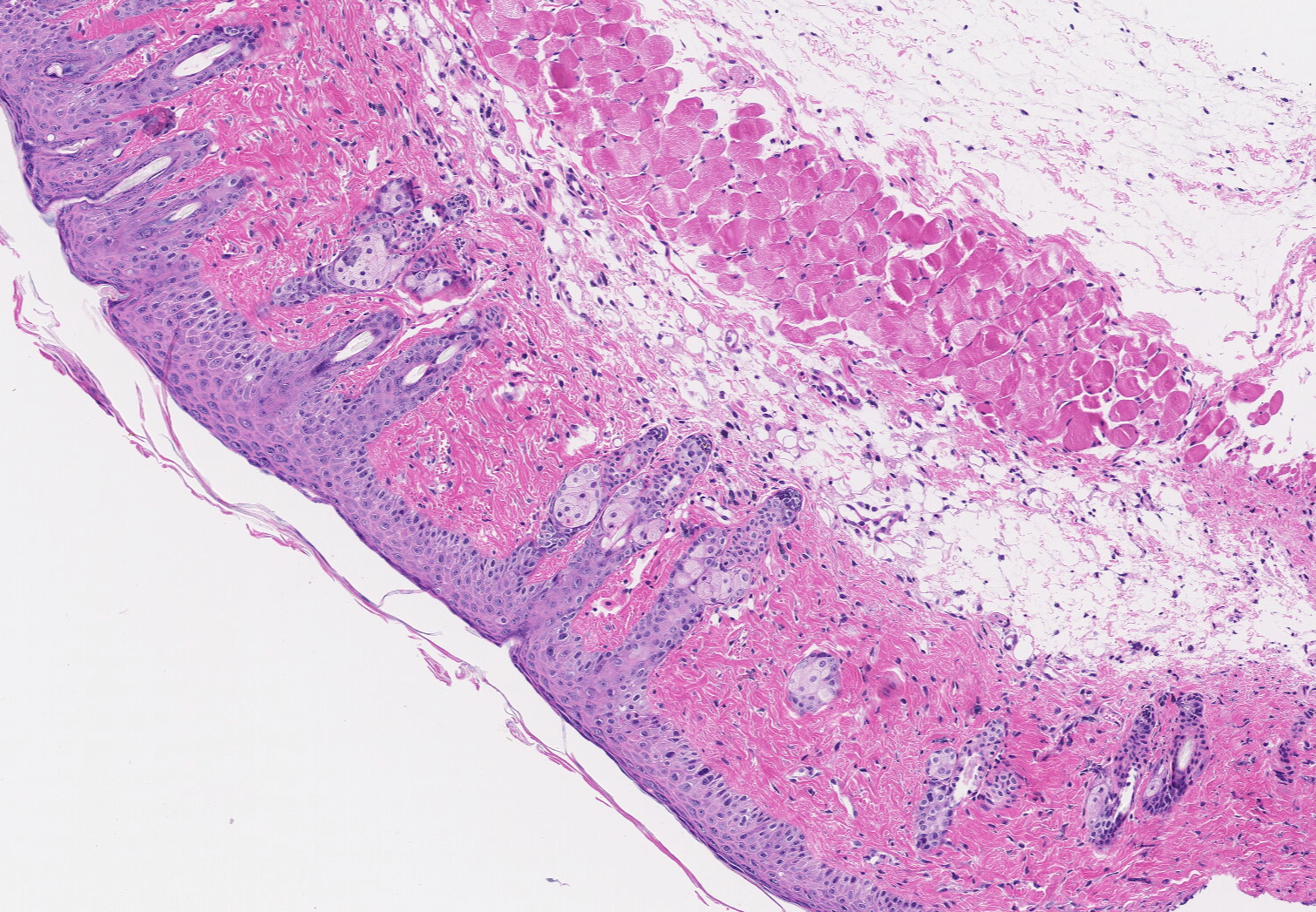

Supplement: Supplementary file 11 — Source Data for Figure 9 [file EMMM-14-e14455-s005.zip › Figure 9/9 A/H&E/KI:WT.tiff]

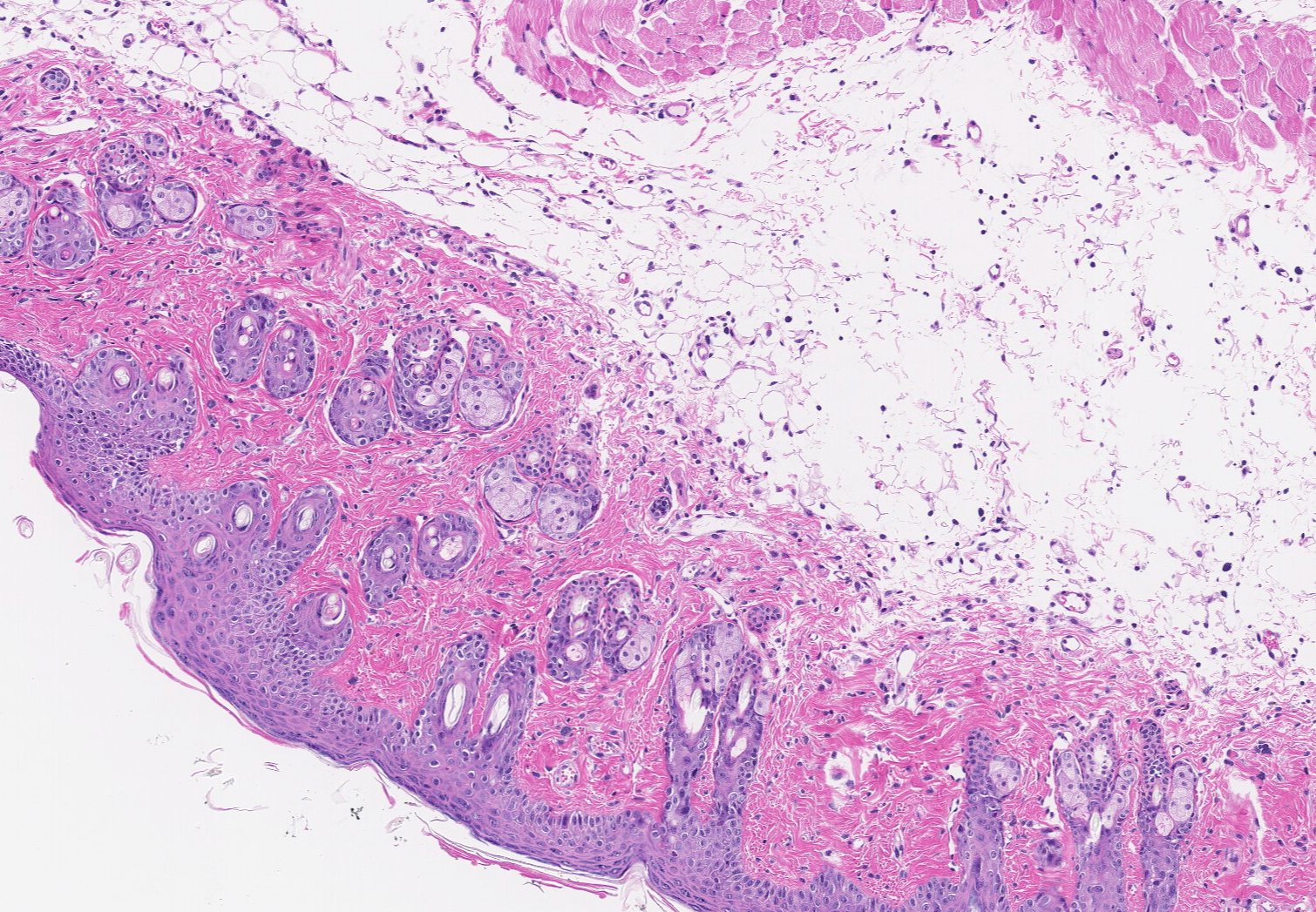

Supplement: Supplementary file 11 — Source Data for Figure 9 [file EMMM-14-e14455-s005.zip › Figure 9/9 A/H&E/WT.tiff]

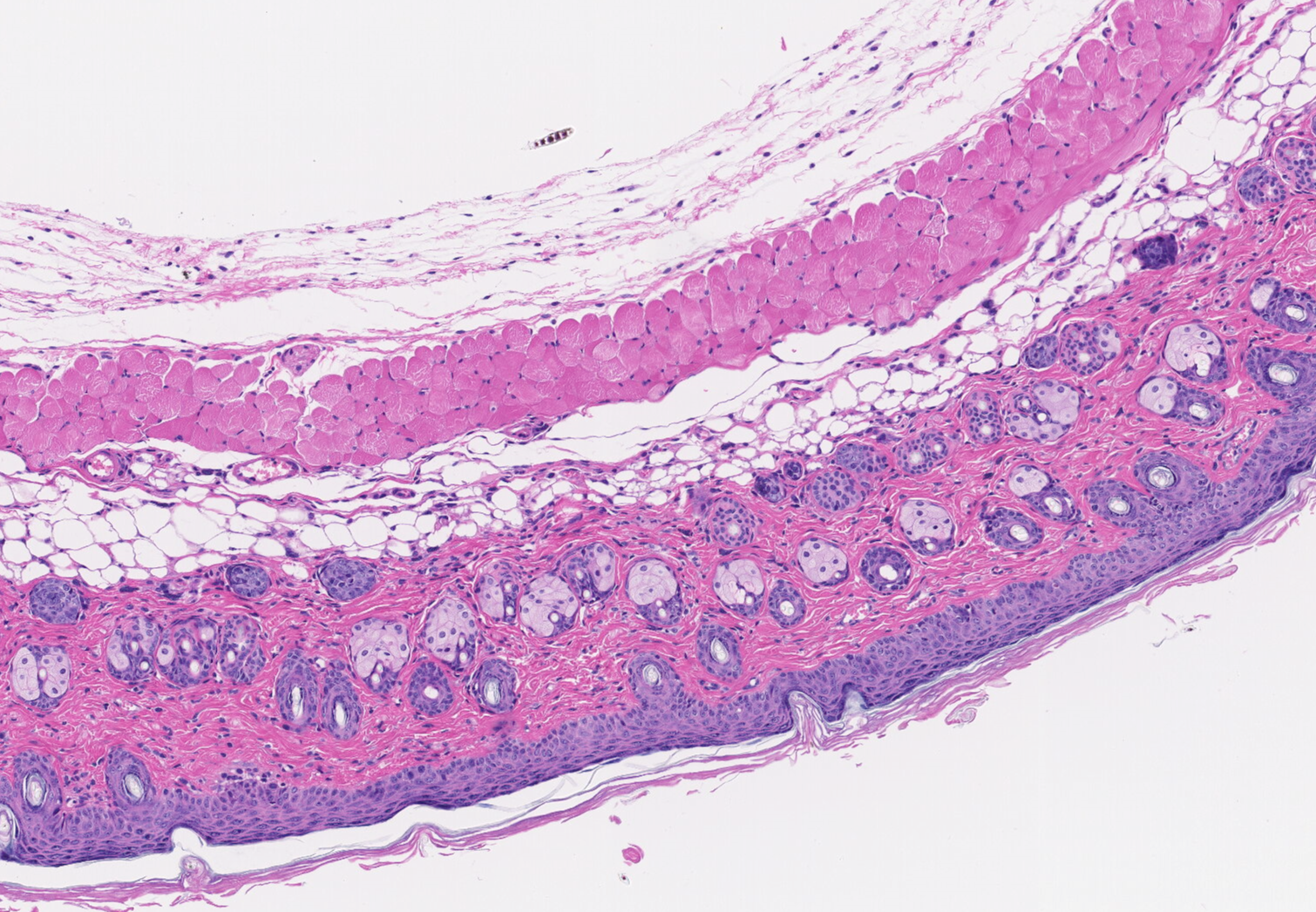

Supplement: Supplementary file 11 — Source Data for Figure 9 [file EMMM-14-e14455-s005.zip › Figure 9/9 A/H&E/KI:KI.tiff]

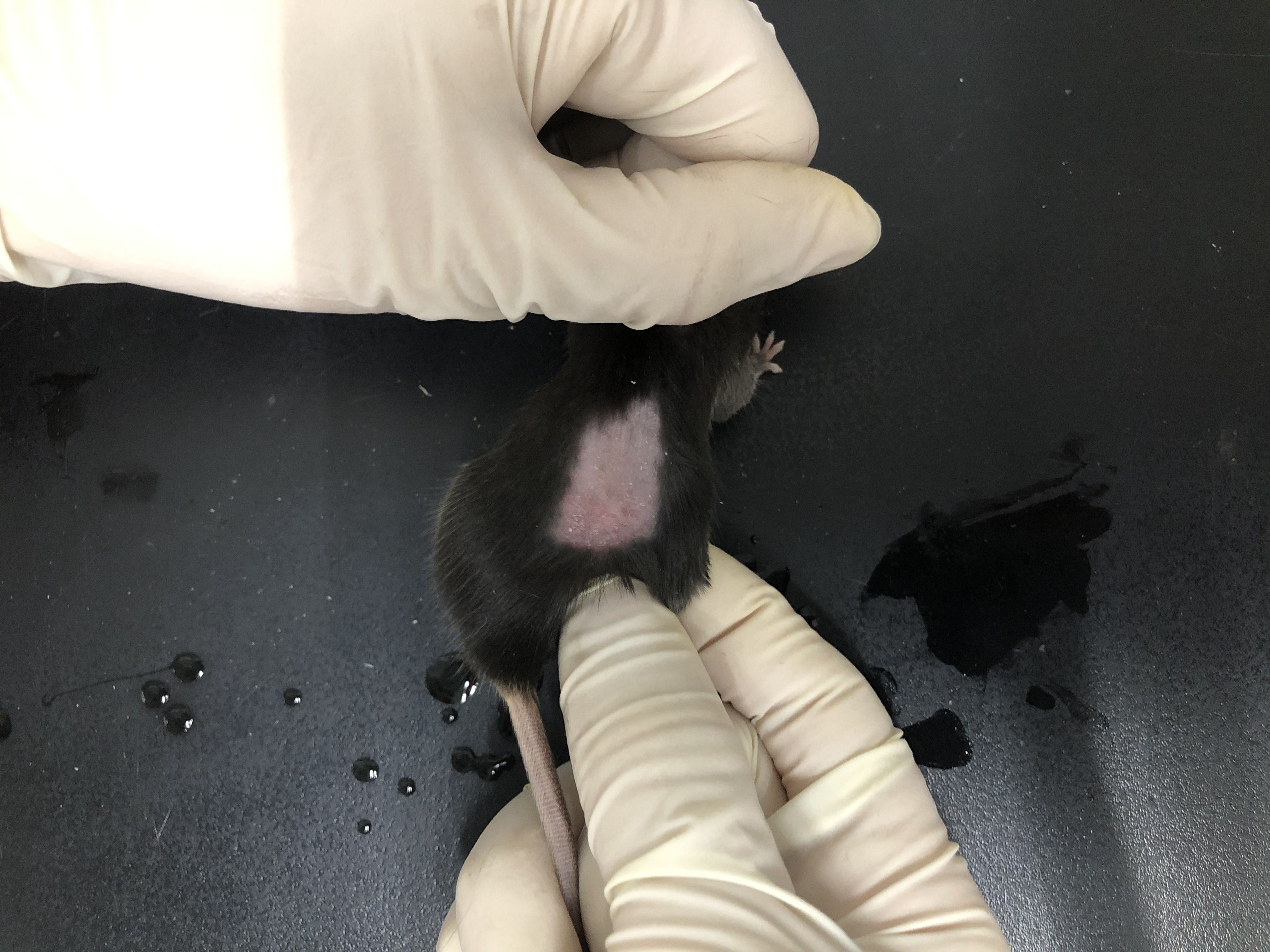

Supplement: Supplementary file 11 — Source Data for Figure 9 [file EMMM-14-e14455-s005.zip › Figure 9/9 A/photo/ki:ki.jpg]

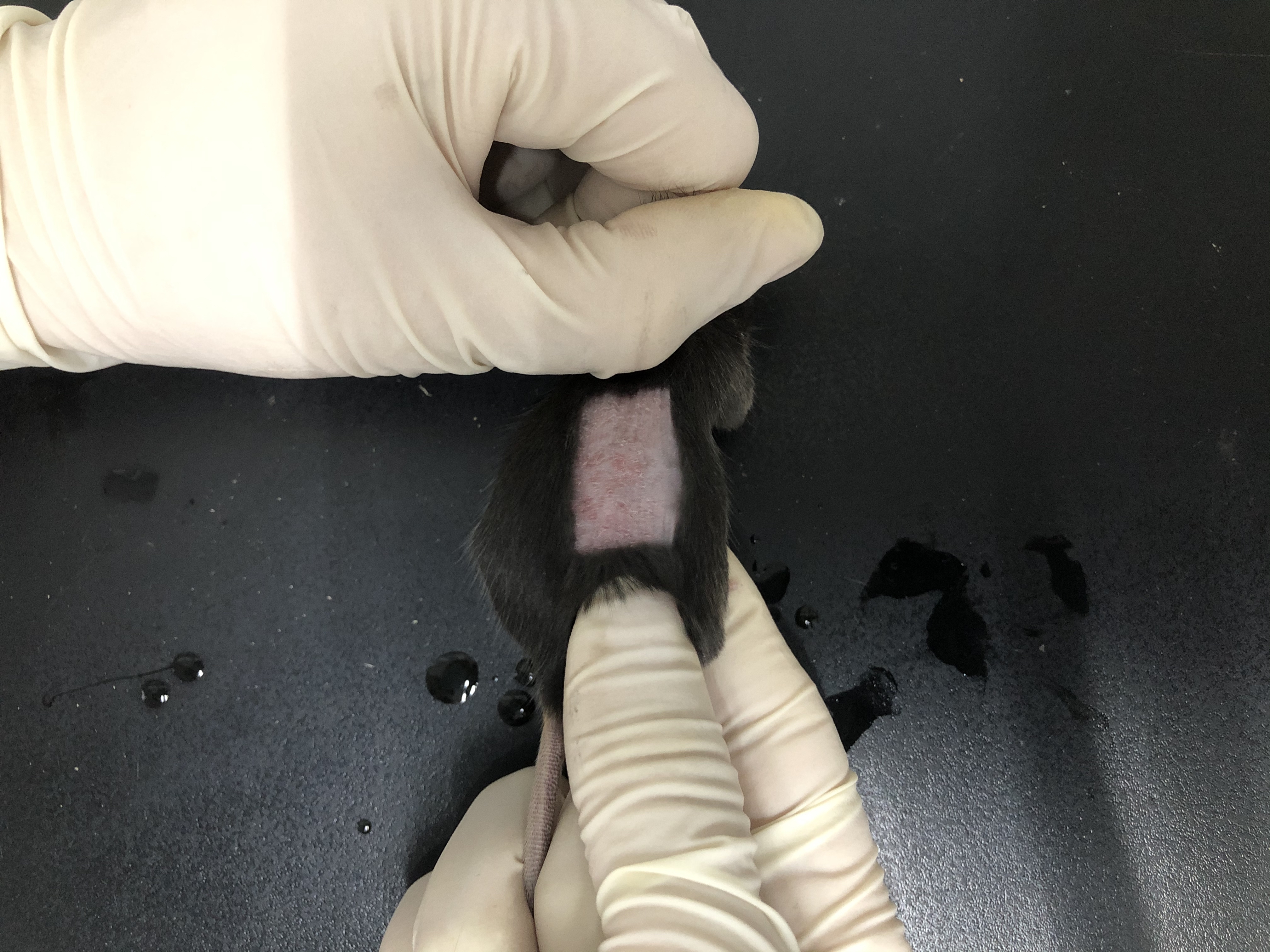

Supplement: Supplementary file 11 — Source Data for Figure 9 [file EMMM-14-e14455-s005.zip › Figure 9/9 A/photo/ki:wt.jpg]

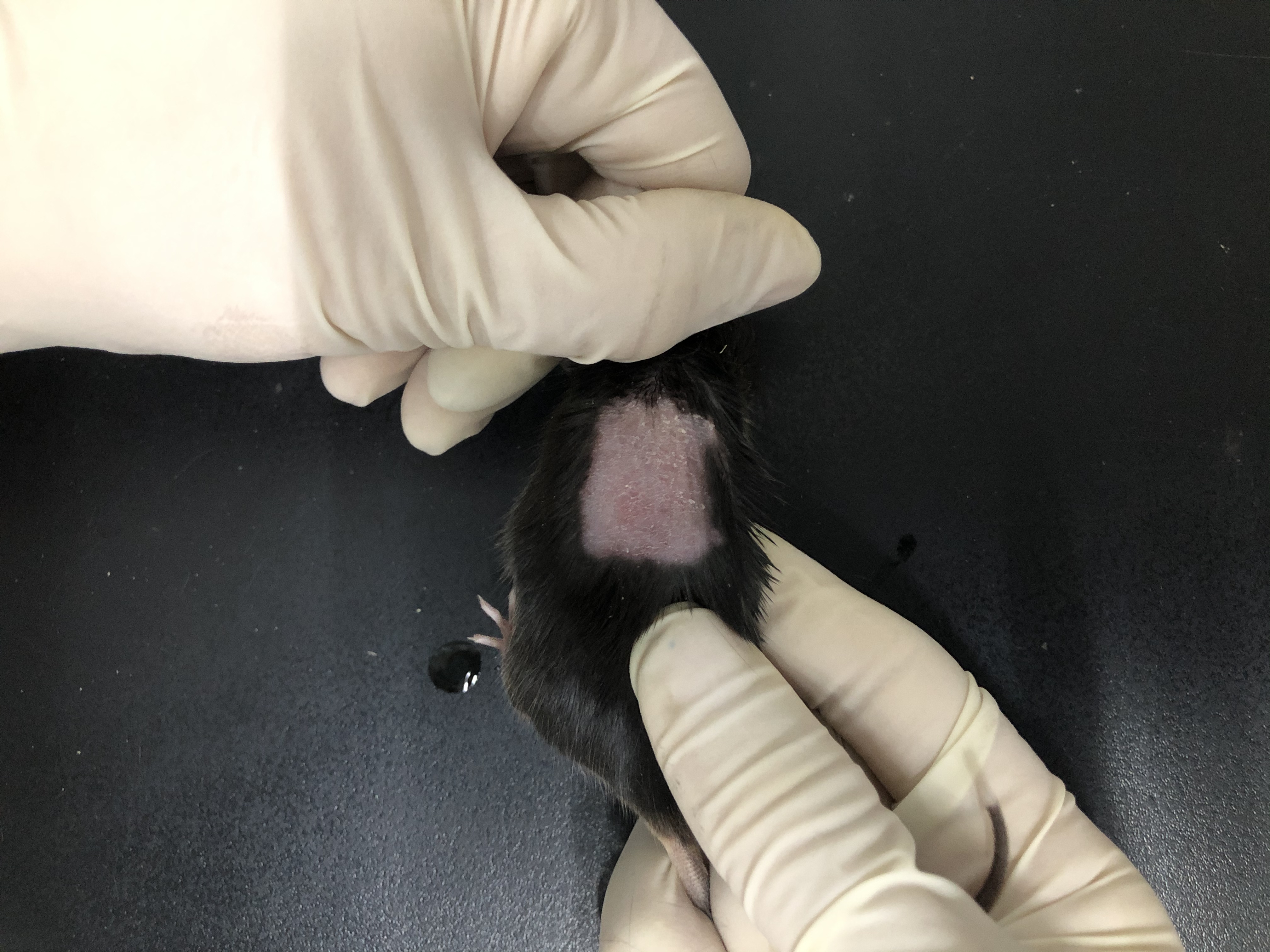

Supplement: Supplementary file 11 — Source Data for Figure 9 [file EMMM-14-e14455-s005.zip › Figure 9/9 A/photo/wt:wt.jpg]
