# Supplementary material for: Allosteric inhibition of SHP2 uncovers aberrant TLR7 trafficking in aggravating psoriasis
Source: EMBO Mol Med. 2021 Dec 22;14(3):e14455. doi: 10.15252/emmm.202114455 (PMC8899919; doi:10.15252/emmm.202114455)
Supplement: Supplementary file 12 [file EMMM-14-e14455-s004.pdf]

EMBO Molecular Medicine

# Allosteric inhibition of SHP2 uncovers aberrant TLR7 trafficking in aggravating psoriasis

Yuyu Zhu, Zhigui Wu, Wei Yan, Fenli Shao, Bowen Ke, Xian Jiang, Jian Gao, Wenjie Guo, Yuping Lai, Hongyue Ma, Dijun Chen, Qiang Xu, and Yang Sun
**DOI: 10.15252/emmm.202114455**

*Corresponding authors: Yang Sun (yangsun@nju.edu.cn)*



## Review Timeline:

| | |
|---|---|
| Submission Date: | 26th Apr 21 |
| Editorial Decision: | 18th May 21 |
| Revision Received: | 19th Sep 21 |
| Editorial Decision: | 8th Oct 21 |
| Revision Received: | 14th Nov 21 |
| Editorial Decision: | 1st Dec 21 |
| Revision Received: | 8th Dec 21 |
| Accepted: | 8th Dec 21 |

*Editor: Zeljko Durdevic*


## Transaction Report:

(Note: With the exception of the correction of typographical or spelling errors that could be a source of ambiguity, letters and reports are not edited. Depending on transfer agreements, referee reports obtained elsewhere may or may not be included in this compilation. Referee reports are anonymous unless the Referee chooses to sign their reports.)

18th May 2021

Dear Prof. Sun,

Thank you for the submission of your manuscript to EMBO Molecular Medicine. We have now received feedback from the three reviewers who agreed to evaluate your manuscript. As you will see from the reports below, the referees acknowledge the interest of the study but also raise serious and partially overlapping concerns that should be addressed in a major revision. Particular attention should be given to validation of the findings in an additional, more relevant psoriasis model and to providing more mechanistic insights.

Further consideration of a revision that addresses reviewers' concerns in full will entail a second round of review. EMBO Molecular Medicine encourages a single round of revision only and therefore, acceptance or rejection of the manuscript will depend on the completeness of your responses included in the next, final version of the manuscript. For this reason, and to save you from any frustrations in the end, I would strongly advise against returning an incomplete revision.

We would welcome the submission of a revised version within six months for further consideration. However, we realize that the current situation is exceptional on the account of the COVID-19/SARS-CoV-2 pandemic. Please let us know if you require longer to complete the revision.

I look forward to receiving your revised manuscript.

Yours sincerely,

Zeljko Durdevic

Zeljko Durdevic
Editor
EMBO Molecular Medicine

**** Reviewer's comments *****

Referee #1 (Comments on Novelty/Model System for Author):

see also comments below. The IMQ model is commonly used as a model of inflammatory skin disease resembling psoriasis, however, verification in additional more relevant models would be suitable. In the case here there are sufficient data in human psoriasis to strengthen the human relevance of their findings

Referee #1 (Remarks for Author):

The manuscript by Zhu et. al. offers an extensive characterization of the role of SHP2 in psoriasis, a phosphatase so far poorly studied in the etiology of this inflammatory skin disease. The authors show increased expression of SHP2 in human psoriatic skin and in a mouse model for psoriasis, based on the topical treatment with the TLR7/8 agonist Imiquimod (Imq). In overexpression and mutation studies performed in cell lines (THP-1 and HEK293 cells) they show that in myeloid cells SHP2 binds to TLR7, thereby dephosphorylating TLR7, modulating its expression and leading to translocation to the early endosomes and activation of downstream signaling via NF-kB. Overall, the data shown in this manuscript is relevant and provides insights into the role of SHP2 in psoriasis. However, some of their claims are not fully supported by the data presented, for example that macrophages are the key players for SHP2 in psoriasis. To strengthen the conclusion of their manuscript more experiments are needed.

Major points:

1) The authors claim that SHP2 plays a role mainly in macrophages. However, e.g. Figures 1D, H, and 6F also show prominent expression in keratinocytes and endothelial cells (especially in human psoriatic skin). What about expression of SHP2 in other immune cells of the skin? These expression analyses should be included. This also applies to e.g. p-p65 which is claimed to be activated downstream of TLR7 (Fig. 6E,F). These findings cannot simply be ignored and need to be discussed. A special focus should be given to dendritic cells (DC) and plasmacytoid dendritic cells (pDC), two populations expressing TLR7 and shown to play prominent roles in the pathogenesis of psoriasis and also great producers of key cytokines like IL-23 and TNFa (Ganguly D. 2009; Riol-Blanco L. 2014; Brunner PM. 2013).
2) In genetically engineered mouse models with cell-specific deletion of SHP2, the authors show that myeloid cells, but not CD11c-positive cells (mainly DCs) contribute to the observed phenotype. Unfortunately, the recombination efficiency of SHP2 is only shown in BMDC of CD11c-Cre+ mice. Recombination in skin-derived DC subpopulations (cDC, pDC) should also be shown as this would be more relevant for psoriasis development, particularly when considering that the recombination efficiency by Cd-11c-Cre is typically not complete in pDC or CD11c-low DCs. Without these results no conclusive statement on the role of DCs is possible.
3) Even more importantly the authors do not provide any data on the recombination efficiency of SHP2 in macrophages and other myeloid cells of the skin in the LysM-Cre mouse model. This is however essential since deletion efficiency in macrophages (and other myeloid populations) with LysM-Cre can vary a lot dependent on the "floxed" gene and is not always complete. In addition, LysM-Cre also recombines in neutrophils and granulocytes. Indeed, the authors show in Figure S 5B that the highest expression of the inflammatory genes is found in cluster M3, which are designated as neutrophils, and in Cluster M2 (Langerhans Cells). A clarification of the role of granulocytes in this phenotype is important.
4) Figure 3B. The scRNA-Seq shows different cell-types defined in clusters. However, cells related to hair-follicles, or also monocytes, which should be present in IMQ-inflamed skin are missing. Could one of the clusters (M0 or M1) also contain/encompass or represent cells related to monocytes?
5) The analysis for KEGG pathways and GSEA etc. (Fig. 4) have been performed on the whole myeloid cell subset but not on subclusters. Performing such analysis on the subclusters could give more insight into the relevance of the individual populations defined.
6) Additional information on SHP2 expression (and other family members), for example in the scRNA Seq data for the specific cell populations would be important. SHP2 is pro-inflammatory following TLR7 triggering in macrophages, but what about its role in TNF-α or IL-22 signalling (two prominent cytokines in psoriasis). Does it have a pro- or maybe even anti-inflammatory role?
7) A number of Western Blots claim changes in e.g. p-IKKa/b, p-p65 or p-Tyr. However, it is sometimes difficult to see the subtle differences. The authors should quantify the bands and normalize them to total protein expression and depict the changes as numbers above the respective graph in all cases where quantitative statements are made. Ideally, the average of more than one

experiment should be shown.
8) Figure 6. Shows that SHP2 controls NF-KB activation. However, other signaling pathways relevant for inflammatory cytokine production are also activated by TLR7 stimulation (IRF and MAPK signaling) and should be at least discussed.
9) The authors nicely analyze the signaling pathways with overexpression studies combined with mutated versions of the involved proteins in THP-1 and HEK 293 cells. These are important studies to mechanistically dissect the pathway. However, to strengthen the relevance of their findings, they need to be verified also in the primary cells claimed to be involved in the etiology of the disease (in the case here, in macrophages or other myeloid cells).
10) Figure 8B: Why does TLR7 protein amount not correlate to GFP amount? Have these cells not been stimulated with IMQ? If not, how does IMQ stimulation affect the expression of the different mutants? In Figures 8 and S16 mainly GFP is used to detect TLR7 protein. However, in Fig. 16F suddenly anti-TLR7 antibody is used. Why that? and is there a difference between the two stainings, e.g. doers GFP expression reflect TLR7 levels?
11) Figure 7G, H: Can the authors explain, why SHP2 protein can be found at equal amounts in each sub-cellular compartment?
12) Figure S14: Although it is clear that there is a difference between WT and M-SHP2-/- peritoneal macrophages in respect of the co-localization of TLR7 and EEA1 at the 0 min time point, it rather seems that at later time points there is less co-localization in WT cells. Changes seem to mainly result from the EEA1 staining. This is different to the data in THP1 (Figure 7C) where there is at least stable co-localization and the difference might be attributed to the anti-mouse TLR7 antibody, which also seems to stain the nucleus. However, in any case it is difficult to draw conclusions from these pictures. This should be clarified. Is it possible to perform flow cytometric analysis at least from the human cells?
13) The quality of some of the IF images is poor and does not allow following the conclusions of the authors. It is necessary to increase the resolution of the confocal images and to show enlarged areas of cells indicated by arrows, at least for the merged images. This is even more important since the authors want to demonstrate co-localizations at the subcellular level (e.g. Figure 7I, S13). A number of these staining rely on the quality of the TLR7 staining, which, however, is often not convincing at all (e.g. Figure 7J, I; S14: here TLR7 staining is found in the nucleus?). TLR7 knock-out skin should be used to control for the specificity of the anti-TLR7 antibody employed here.
14) Additionally, the CD68 staining in Figure S10 is not convincing since there seems to be no macrophages in healthy skin, neither by IHC nor by IF staining.
15) There is also some confusion about the designation of these 2 Clusters (M2 and M3) in Figure 4, where they are inversely labeled between B and G! Which one is correct? This is also important for understanding Fig S 5B. Even more confusion comes from the fact that the Cluster M5 is missing in Figure S3!
16) Most of the statistical analysis was performed by using Student´s t-test (e.g. cytokine analyses). However, in experiments with more than two experimental groups an ANOVA test with a post test that corrects for multiple testing should be applied!
17) Fig 2A. SHP099 alleviates IMQ induced skin inflammation as quantified by epidermal thickness. However, differences in acanthosis are not clearly visible in the hematoxylin and eosin staining shown. These pictures also do not correspond to the quantification shown below, where a reduction in skin thickness of more than 1/3 is shown, irrespective of the dose.
18) Figure 5G. and Figure 6C both look at psoriasis-related genes decreased in the IMQ model of Shp2-/- mice compared to wild-type mice. Maybe this can be combined to reduce redundancy.

Referee #2 (Comments on Novelty/Model System for Author):

The so-called imiquimod model does not reflect all the features of the human disease (see Comments to Authors).

Referee #2 (Remarks for Author):

The manuscript by Zhu et al. provides some evidence for a role of SHP2 as a regulator of psoriasis-like skin lesions of imiquimod-treated mice and, much less so, of the human disease. In this context, one should remember that the so-called imiquimod model does not reflect many of the salient features of psoriasis such as the lack of co-morbidities and of chronicity.
This reviewer has a few questions, comments and criticisms:
1. Fig. 1D illustrates the differences in SHP2 expression between NHS and psoriatic skin. Were all of the positively stained cells macrophages?
What about TIPP-DCs, i.e. TNF-alpha and iNOS-expressing cells which supposedly play a critical role in the pathogenic IL-23/IL-17 axis?
2. Have the authors verified that the DC- and macrophage knockout mice were indeed devoid of the respective cell populations?
3. Ample evidence exists that application of imiquimod to both human and murine skin results in the accumulation of substantial numbers of plasmacytoid dendritic cells (pDC. These cells, by the way, are to great extent responsible for the efficacy of imiquimod in the treatment of genital warts, actinic keratoses and basal cell carcinomas.
In the scRNA-seq analysis (Fig. 3A-C) I could not found pDC in any of the 6 clusters/25 subclusters. How can this be explained?
4. Of minor importance: the manuscript contains many typos and linguistic flaws. They should be corrected.

Referee #3 (Comments on Novelty/Model System for Author):

LysMcre mice may be useful for Cre-lox studies, but authors need to be careful with their language; LysMcre-lox will delete in all myeloid cell lineages, which include monocytes, mature macrophages and granulocytes. Therefore, the authors cannot specifically assume that the effects of SHP2 in psoriasis stem strictly from macrophage expression.

Referee #3 (Remarks for Author):

The manuscript entitled, "Allosteric inhibition of SHP2 uncovers aberrant TLR7 trafficking in aggravating psoriasis" by Zhu et. al. sought to investigate whether TLR7 regulation of NFkB signaling in macrophage is regulated by SHP2 in psoriasis. This is an elegant paper that reveals that SHP2 may be an accelerator of psoriasis and that its inhibition may be a promising therapeutic for patients. There are a few concerns, however, that if rectified would strengthen the manuscript. Specifically, the conclusions are overstated somewhat based on the experimentation presented. Therefore, there is a need for some additional mechanistic data to prove these potential findings. In particular, no data is presenting showing the binding site of SHP2 to TLR7. Moreover, the role of TLR7 (as opposed to other downstream effectors) specifically in the regulation of psoriasis-associated inflammation in response to SHP2 activity is not clearly justified, as no other downstream effectors were evaluated directly. Finally, quantification of all western data is needed, as well as more information on statistical assessments and n values used for experiments. Details on these and other concerns are outlined below:

Major concerns:
1) Control experiments are largely missing throughout. For example, in Fig 1I, normal PBMCs should similarly be treated with SHP099 as the patient PBMCs, to assess cytokine effects of the inhibitor in control cells. Similarly, in Figs 2A and 3A, sham mice should also be treated with the inhibitor to compare the effects at baseline in normal mice.
2) Fig 3: It is clear that the lymphocytes, and to a lesser extent myeloid cells, play a key role in upregulating inflammatory genes in psoriasis. However, which cell type is most affected by SHP099 treatment, ie which cell type becomes most normalized?
3) LysMcre mice may be useful for Cre-lox studies, but authors need to be careful with their language; LysMcre-lox will delete in all myeloid cell lineages, which include monocytes, mature macrophages and granulocytes. Therefore, the authors cannot specifically assume that the effects of SHP2 in psoriasis stem strictly from macrophage expression.
4) Fig 5: It is clear that skin lesions are normalized in IMQ-treated SHP2-deleted monocytes; however, the data for IMQ-treated SHP2 deleted dendritic cells is missing and integral to show that the effects are predominantly affecting macrophage and not DCs.
5) Fig 6: Baseline macrophage data are needed to compare and determine normalization of genes in macrophage in response to IMQ treatment in SHP2 deleted cells.
6) Why did the authors overexpress SHP2 instead of measure its effects in SHP2 immunocomplexes endogenously? For example, in response to IMQ, an experiment to IP SHP2 blot pY and reprobe for TLR7 would show whether SHP2 binds to TLR7 and affects its phosphorylation directly. This should be demonstrated in macrophage and BMDCs.
7) As well, the authors mention that they identified several SHP2-interacting proteins but only focused on TLR7. The authors need to identify these interacting proteins and then validate that it is indeed TLR7 that is integral to the SHP2-mediated effects in psoriasis.
8) Authors also indicate that TLR7 binds SHP2 in the PTP domain; how is this possible, at what site/residue? Validation here is required as this is not the normal binding for SHP2 to proteins; almost all binding for SHP2 occurs through its SH2 domains to pY proteins (like TLR7) or through C-terminal tail binding at its own pY residues. The size of the PTP deletion mutant suggests that it is a truncated protein that may not express the c-terminus either?
9) Fig 8: The authors suggest SHP2 affects TLR phosphorylation, but this was never directly measures. Authors instead show that mutations at various tyr residues affects expression, but this is not the same as showing its phosphorylation.
10) Fig S16: Authors indicate that ubiquitination of TLR7 is increased when SHP2 is overexpressed; however, if the role of SHP2 is to increase TLR7 expression in general, wouldn't the increase in ub be an indirect consequence based on this fact? It seems therefore that the ub of TLR7 may be strictly a consequence of there being more of it in the cell, and not directly due to regulation by SHP2.
11) What about other TLR proteins? Likely SHP2 regulates TLR 3, 8, 9, etc as well; authors should demonstrate the effects of SHP2 and its inhibition on these proteins as well. In addition, effects on ACT1 should also be shown.
12) All western blot analyses should be quantified, with statistics, and represented by results from at least 3 independent experimental replicates. As well, downstream activities of SHP2 in response to IMQ or in psoriasis in the presence or absence of SHP099 should be shown by western blot (p-ERK and p-AKT).
13) Number of mice used for omics data is not indicated.

Minor concerns:
1) Grammatical corrections are needed throughout.
2) Scale bars for most histological sections are missing.

3) Several figures have missing x or y axis labels.
4) ELISA and qRT-PCR data should be measured for fold change (not absolute) and normalized to gapdh or other housekeeping genes.

Dear Editor and reviewers,

Thank you and reviewers for the valuable time and insightful comments. We appreciate the opportunity to revise and improve our manuscript. We have performed additional experiments and revised our manuscript according to the reviewers' comments and suggestions. All changes have been highlighted in **Red** in the revised files.

Below are a list of the major changes and the point-by-point responses to the comments. We appreciate your insightful suggestion and we provide an IL-23-induced psoriasis-like mouse model (Figure EV1 and Appendix Fig 20) that leads to the similarly conclusions as the IMQ-induced mouse model.

We show some of the previous data in our responses to the comments and have reassembled the figures for your convenience and that of the reviewers.

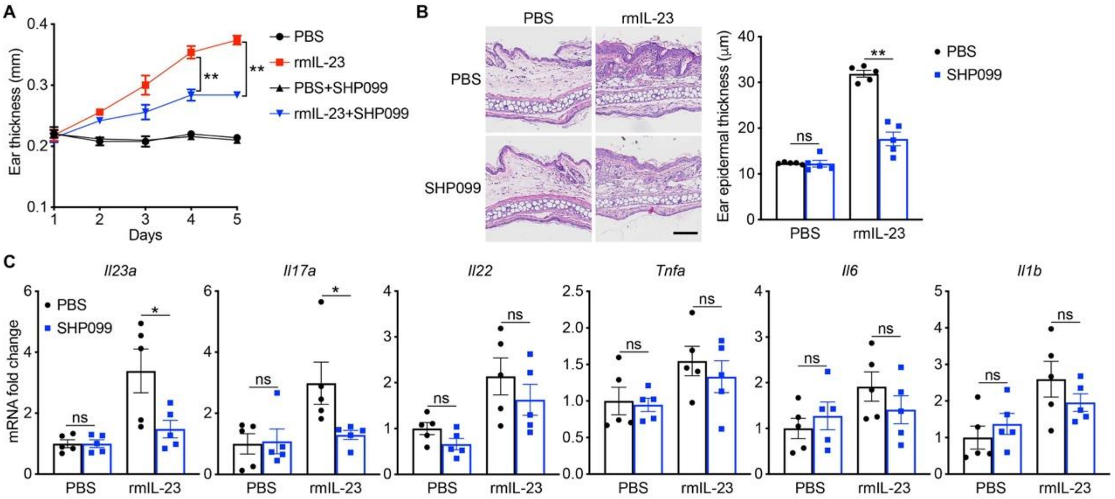


Figure EV1. SHP099 attenuated the psoriasis-like phenotype in the IL-23-induced murine model.

C57BL/6 mice (*n*=5/group) were subjected to IL-23-induced psoriasis-like skin inflammation and were treated with 10 mg/kg SHP099 or PBS for 4 days.

A, B Ear thickness (A), H&E staining and statistic results (B) of ear skin from SHP099 treated with or without C57BL/6 mice injected intradermally with rmIL-23 or PBS for 4 days. Ear thickness was measured daily.

C Quantitative PCR analysis of mRNA encoding IL-23/IL-17A axis cytokines and other psoriasis-related cytokines in the ear skin. Results were normalized to *Gapdh* expression.

Data information: Data are represented mean ± SEM. *P* values are determined by Tukey multiple comparison test (A–C). *$P$<0.05, **$P$<0.01, ns, not significant.

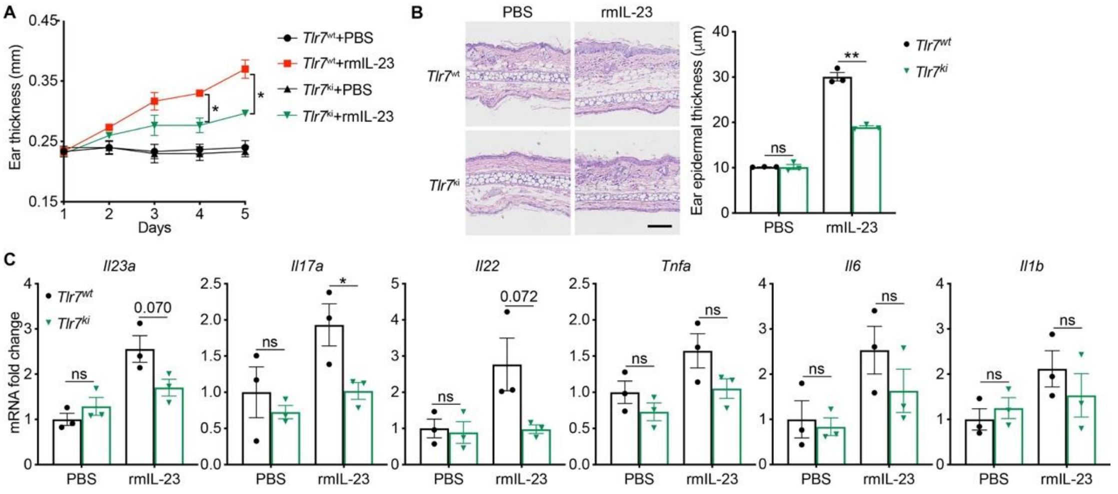


Appendix Fig S20. IL-23-induced psoriasis-like phenotype was alleviated in *Tlr7*$^{ki}$ mice. *Tlr7*$^{wt}$ male mice (*n*=3) and *Tlr7*-Y1025D mutant *Tlr7*$^{ki}$ male mice (*n*=3) were treated with indicated dose of rmIL-23 or PBS for 4 days.

A, B Ear thickness (A), H&E staining and statistic results (B) of ear skin from *Tlr7*$^{wt}$ and *Tlr7*$^{ki}$ mice injected intradermally with rmIL-23 or PBS for 4 days. Ear thickness was measured daily.

C Quantitative PCR analysis of mRNA encoding IL-23/IL-17A axis cytokines and other psoriasis-related cytokines in the ear skin. Results were normalized to *Gapdh* expression.

Data information: Data are represented mean ± SEM. *P* values are determined by Tukey multiple comparison test (A–C). **P*<0.05, ***P*<0.01, ns, not significant.

***** Reviewer's comments *****

Referee #1 (Comments on Novelty/Model System for Author):

see also comments below. The IMQ model is commonly used as a model of inflammatory skin disease resembling psoriasis, however, verification in additional more relevant models would be suitable. In the case here there are sufficient data in human psoriasis to strengthen the human relevance of their findings

Referee #1 (Remarks for Author):

The manuscript by Zhu et al. offers an extensive characterization of the role of SHP2 in psoriasis, a phosphatase so far poorly studied in the etiology of this inflammatory skin disease. The authors show increased expression of SHP2 in human psoriatic skin and in a mouse model for psoriasis, based on the topical treatment with the TLR7/8 agonist Imiquimod (Imq). In overexpression and mutation studies performed in cell lines (THP-1 and HEK293 cells) they show that in myeloid cells SHP2 binds to TLR7, thereby dephosphorylating TLR7, modulating its expression and leading to translocation to the early endosomes and activation of downstream signaling via NF-kB. Overall, the

data shown in this manuscript is relevant and provides insights into the role of SHP2 in psoriasis. However, some of their claims are not fully supported by the data presented, for example that macrophages are the key players for SHP2 in psoriasis. To strengthen the conclusion of their manuscript more experiments are needed.

Reply: Thank you so much for positive and constructive evaluation.

Major points:

1) The authors claim that SHP2 plays a role mainly in macrophages. However, e.g. Figures 1D, H, and 6F also show prominent expression in keratinocytes and endothelial cells (especially in human psoriatic skin).

Reply: We agree with you. In Figures 1D and H, we examined the expression of SHP2 in human and mouse skin tissues and found that SHP2 was highly expressed in the skin tissues of psoriatic patients and IMQ-induced mice.

Violin plot (A) and feature plot (B) showed the expression and distribution of *Ptpn11* in subpopulations of mouse skin cells, indicating that *Ptpn11* is expressed in a variety of cells.

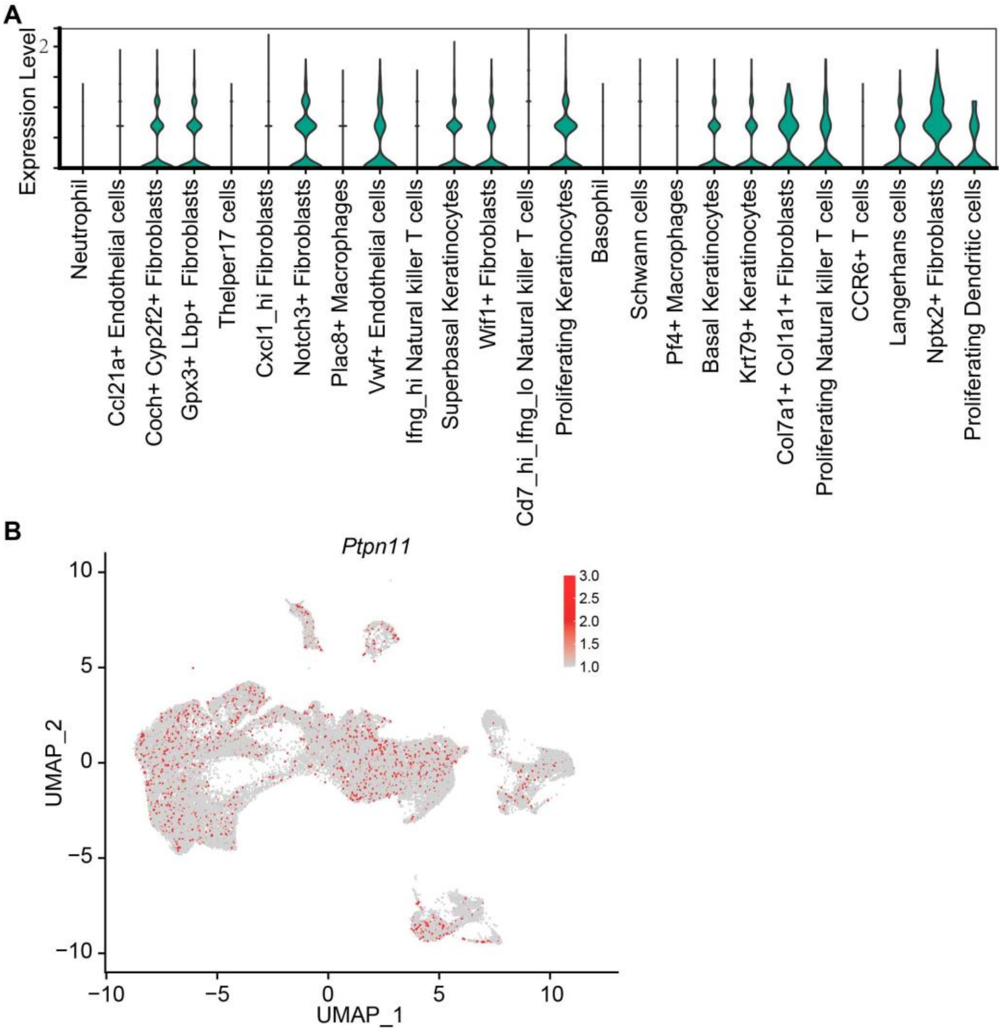


Response Figure 1. *Ptpn11* expression in all clusters of mouse skin cells.

(A) Violin plot showed the distribution of the normalized expression level of *Ptpn11*.

(B) Feature plots of expression distribution for *Ptpn11*. Expression levels are color-coded and overlaid onto UMAP plot.

What about expression of SHP2 in other immune cells of the skin? These expression analyses should be included.

Reply: This is a good question. Violin plot analysis showed *Ptpn11* expression in mouse skin immune cells, as a result of integrating data from three groups of mice. Thank you for your advice.

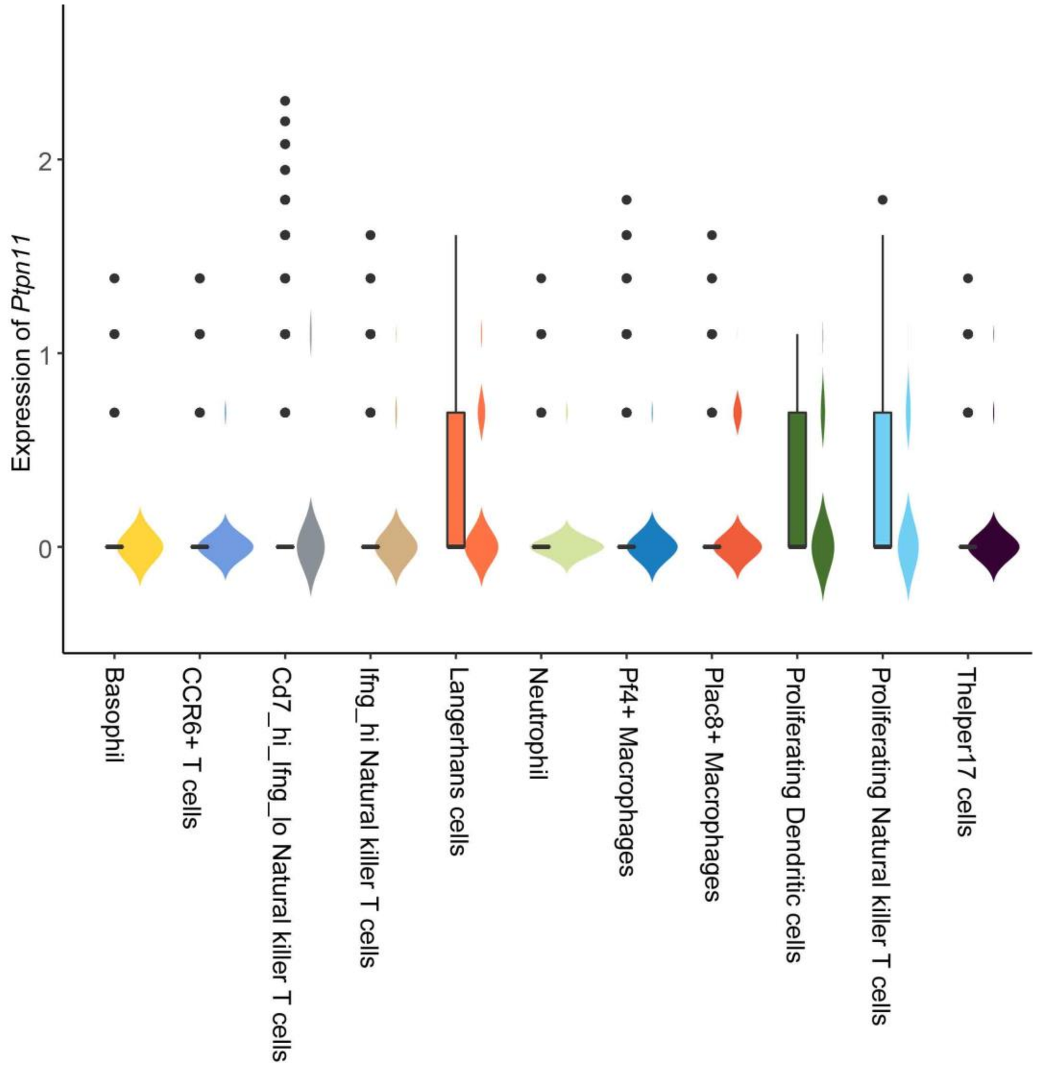


Response Figure 2. *Ptpn11* expression in immune cells of mouse skin.

Violin plot showed the distribution of the normalized expression level of *Ptpn11* in immune cells. Sham (left), IMQ (middle, column), IMQ+SHP099 (right, violin).

In addition, immunofluorescence staining demonstrated that SHP2 was also highly expressed in neutrophils (A) and DCs (B) in the skin of psoriasis patients compared to healthy control.

We have updated these data in **Appendix Fig S13** in our revised manuscript.

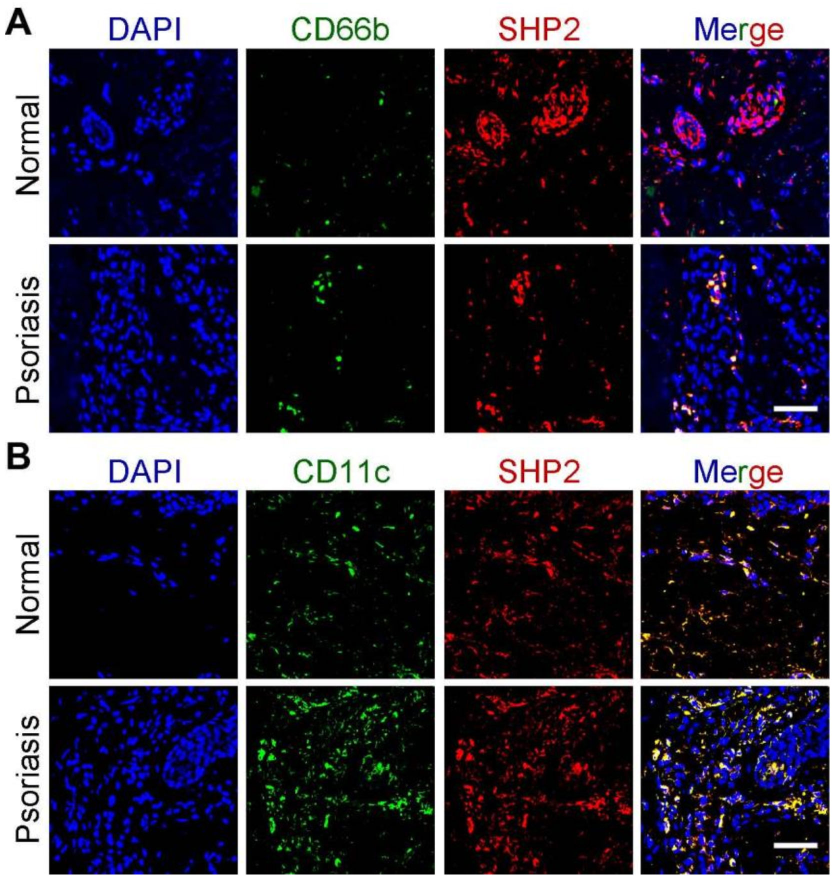


Response Figure 3. SHP2 highly expressed in neutrophils and DCs of skin lesion tissues of psoriatic patients.

(A and B) Immunofluorescence staining of skin sections from psoriatic patients and normal controls with SHP2 and CD66b (A) or CD11c (B), respectively showing SHP2 expression in neutrophils or DCs. Scale bar: 50 μm.

This also applies to e.g. p-p65 which is claimed to be activated downstream of TLR7 (Fig. 6E, F). These findings cannot simply be ignored and need to be discussed.

Reply: We have followed the reviewer's helpful suggestion and revealed significantly higher levels of p-p65 protein in the infiltrated macrophages of the skin lesions collected from IMQ-induced mice (A) and psoriatic patients (B).

We have updated these data in **Figure 6E** and **Appendix Fig S15** in our revised manuscript.

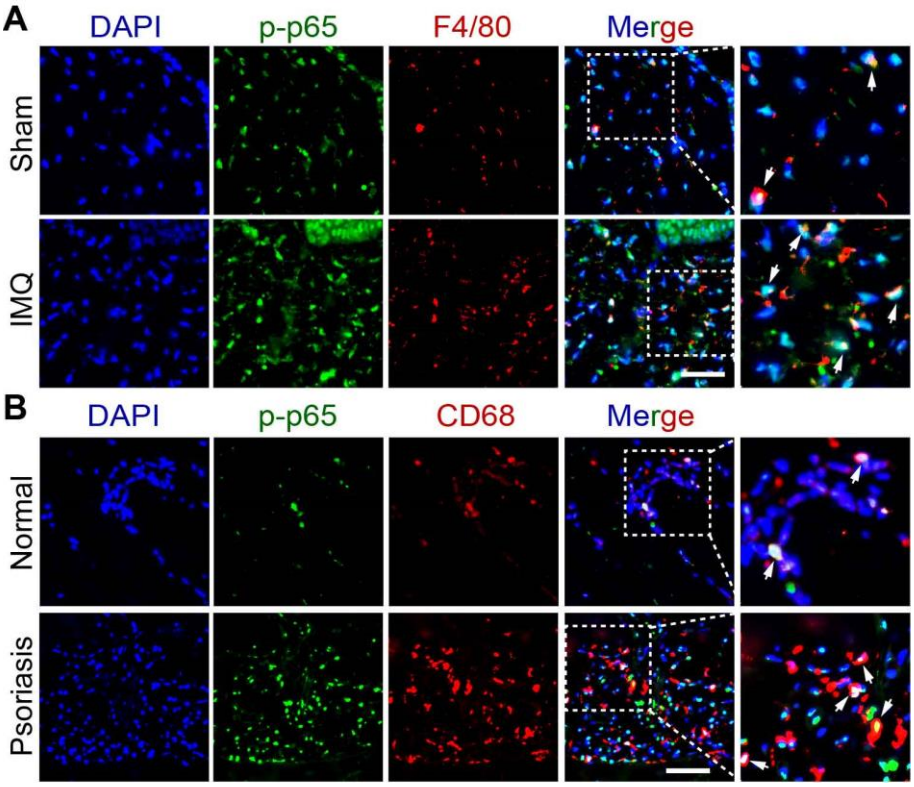


Response Figure 4. p-p65 highly expressed in macrophages of skin lesion tissues of IMQ-induced mice and psoriatic patients.

(A and B) Representative p-p65 immunofluorescence staining of skin sections from mouse (A) and human (B). Scale bar: 50 μm.

A special focus should be given to dendritic cells (DC) and plasmacytoid dendritic cells (pDC), two populations expressing TLR7 and shown to play prominent roles in the pathogenesis of psoriasis and also great producers of key cytokines like IL-23 and TNFa (Ganguly D. 2009; Riol-Blanco L. 2014; Brunner PM. 2013).

Reply: We totally agree with you. Dendritic cells (DCs) play an important role in both the initiation (Wang & Bai, 2020, PMID: 31833093) and developmental stages (Ganguly *et al*, 2009, PMID: 19703986; Riol-Blanco *et al*, 2014, PMID: 24759321; Brunner *et al*, 2013, PMID: 23890755) of psoriasis. However, knockout of SHP2 in monocytes only improved psoriasis-like disease in mice, but not in DCs or $\gamma\delta$ Th17 cells (Kadekar *et al*, 2020, PMID: 32092146), suggesting that SHP2 promotes psoriasis progression with monocyte specificity. In addition, top ten hub genes were mainly expressed in macrophages (Fig. 4 G), implying that macrophages may function as master regulators in a cell type-specific manner.

We have updated these data in **Figure 4G** in our revised manuscript.

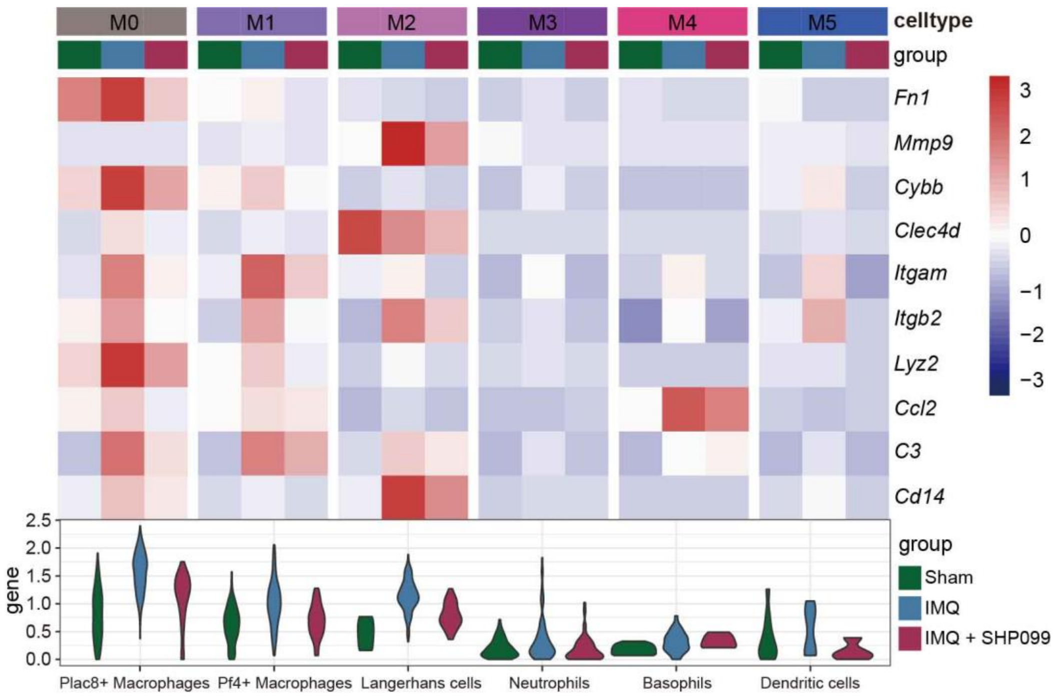


Figure 4G. Expression patterns of the top ten hub genes in Fig. 4F.
Heatmap (*top*) displays the average expression in the three groups and in different myeloid cell subsets. Boxplots (*bottom*) show the corresponding expression distribution.

2) In genetically engineered mouse models with cell-specific deletion of SHP2, the authors show that myeloid cells, but not CD11c-positive cells (mainly DCs) contribute to the observed phenotype. Unfortunately, the recombination efficiency of SHP2 is only shown in BMDC of CD11c-Cre+ mice. Recombination in skin-derived DC subpopulations (cDC, pDC) should also be shown as this would be more relevant for psoriasis development, particularly when considering that the recombination efficiency by Cd-11c-Cre is typically not complete in pDC or CD11c-low DCs. Without these results no conclusive statement on the role of DCs is possible.

Reply: The dendritic cell-specific SHP2 knockout mice (DC-*Shp2*$^{-/-}$ mice) were generated by crossing *Shp2* floxed mice with *Itgax*-Cre transgenic mice, which were purchased from Jackson Laboratory (JAX: 008068).

*Itgax*-Cre has 100% knockdown efficiency at cDCs and pDCs (Caton *et al*, 2007, PMID: 17591855; Durai & Murphy, 2016, PMID: 27760337), thus, SHP2 was indeed knocked out in DCs. Interestingly, the absence of SHP2 in DCs did not improve the development of psoriasis in mice, a similar phenomenon was also observed in $\gamma\delta$ Th17 cells (Kadekar *et al*, 2020, PMID: 32092146), indicating a cell-specific role of SHP2 in psoriasis.

**Table 2. Comparison of Cre Strains for Conditional Deletion of Genes in Dendritic Cells**

| Strain | Cells Affected | Caveats |
| --- | --- | --- |
| *Itgax-cre* (BAC) (Caton et al., 2007) | ~100% of cDCs, pDCs, alveolar macrophages, ~70% of red pulp macrophages, 20%–40% of monocytes, marginal zone macrophages, peritoneal macrophages, ~5%–12% of T, B, NK cells | Germline deletion possible |
| *Itgax-cre* (Transgene) (Stranges et al., 2007) | cDCs, pDCs | Deletion in other cell types was not analyzed, most likely similar to other *Itgax-cre* strain |
| *Clec9a-cre* (Schraml et al., 2013) | ~100% of cDC1s, ~50% of cDC2s, ~20% of pDCs | Begins deletion at CDP stage, low or transient expression might affect deletion efficiency in cDC2s and pDCs |
| *Zbtb46-cre* (Loschko et al., 2016b) | 65%–95% of cDCs, <10% of other immune cells | Deletion efficiency variable depending on floxed allele |
| *Cd207-cre* (Zahner et al., 2011) | Langerhans cells, Langerin$^+$ cDC1s in dermis, skin-draining LNs, and lung | |
| *Xcr1-cre* (Ohta et al., 2016) | cDC1s | |
| *Siglech-cre* (Puttur et al., 2013) | ~30% of pDCs, ~2% of other lymphoid cells | Incomplete deletion in pDCs. Deletion in marginal zone macrophages untested but possible |

Durai V & Murphy KM. Functions of Murine Dendritic Cells. *Immunity* (2016). PMID: 27760337

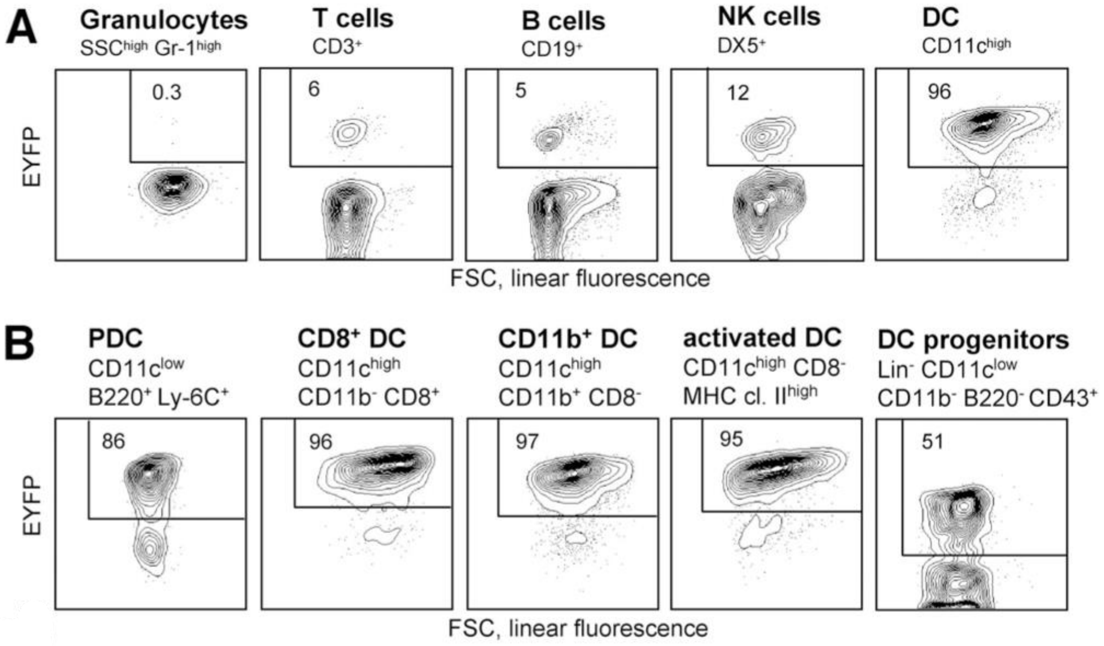


Caton ML, *et. al*. Notch-RBP-J signaling controls the homeostasis of CD8$^-$ dendritic cells in the spleen. *J Exp Med* (2007). PMID: 17591855

3) Even more importantly the authors do not provide any data on the recombination efficiency of SHP2 in macrophages and other myeloid cells of the skin in the LysM-Cre mouse model. This is however essential since deletion efficiency in macrophages (and other myeloid populations) with LysM-Cre can vary a lot dependent on the "floxed" gene and is not always complete.

Reply: Thank you for this comment. In fact, we have showed these data in Fig 6D. SHP2 was significantly knocked out in BMDMs and PMs of Mø-*Shp2*$^{-/-}$ mice compared to wild-type mice.

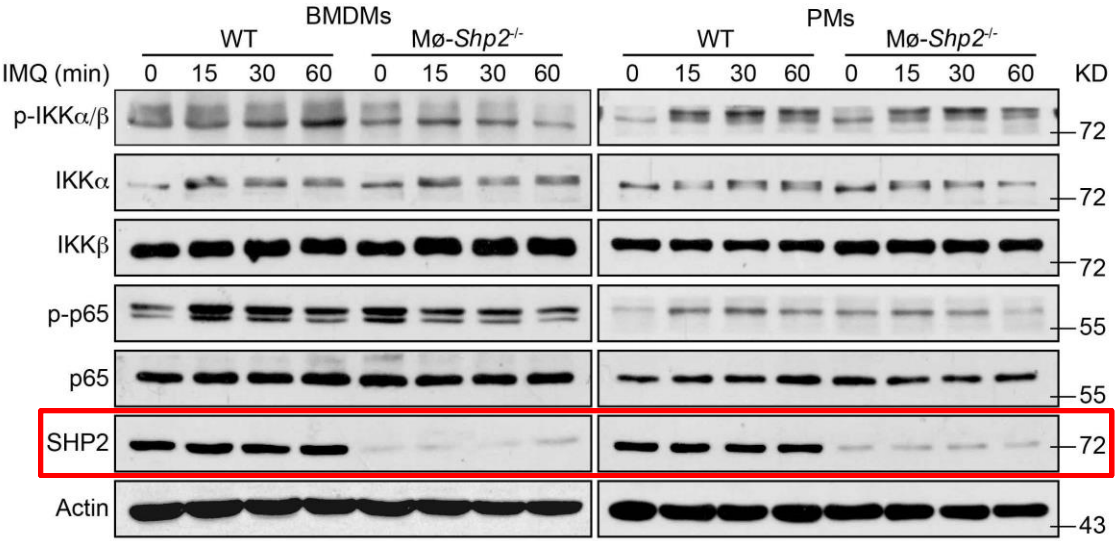


Figure 6D. BMDMs and PMs derived from wild-type and Mø-*Shp2*$^{-/-}$ mice were stimulated by IMQ (10 µg/ml) for indicated times. Whole cell lysates were subjected to Western blotting.

We followed the reviewer's comments and detected SHP2 expression in BMDMs and BMDCs of Mø-*Shp2*$^{-/-}$ mice, and we confirmed that SHP2 was knocked out in BMDMs only.
We have updated these data in **Appendix Fig S10C** in our revised manuscript.

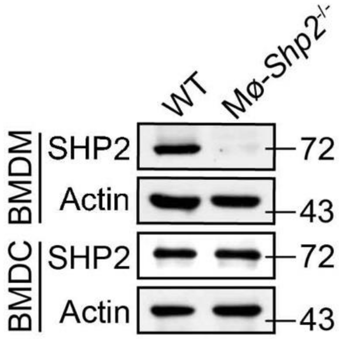


Response Figure 5. Deletion efficiency of SHP2 in Mø-*Shp2*$^{-/-}$ mice.
Cell lysates from BMDMs and BMDCs generated from Mø-*Shp2*$^{-/-}$ mice and wild-type mice were probed by anti-SHP2 and anti-Actin.

Coincidentally, Hui Xiao and his colleagues documented that *Lyz2*-cre:*Shp2*$^{fl/fl}$ mice are deficient for SHP2 in macrophages, but not in BMDCs, B cells and T cells (Deng *et al*, 2015, PMID: 25915733).

In addition, LysM-Cre also recombines in neutrophils and granulocytes. Indeed, the authors show in Figure S5B that the highest expression of the inflammatory genes is found in cluster M3, which are designated as neutrophils, and in Cluster M2 (Langerhans Cells). A clarification of the role of granulocytes in this phenotype is important.

Reply: We agree with you. Oxidative stress, granule composition and neutrophil extracellular traps of neutrophils are associated with the initial and maintenance phases of psoriasis (Chiang *et al*, 2019, PMID: 31649677; Jiang *et al*, 2019, PMID: 31539277; Lin *et al*, 2011, PMID: 21606249; Wang & Jin, 2020, PMID: 32587871; Katayama, 2018, PMID: 30019426; Wójcik *et al*, 2020, PMID: 32947961).

In fact, other colleagues in our laboratory are conducting a related study in which they found that neutrophil conditional knockout SHP2 mice have reduced psoriasis phenotype compared to wild-type mice. This part of the data has not been published and is only available to the editor and reviewers.

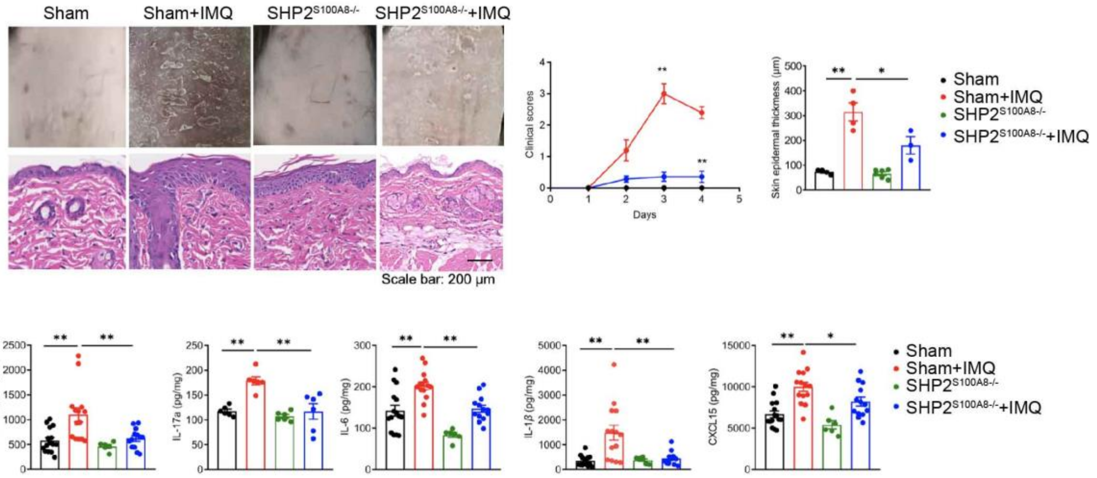


Response Figure 6. Neutrophil conditional knockout SHP2 mice with remission of psoriasis symptoms (Unpublished observations).

4) Figure 3B. The scRNA-Seq shows different cell-types defined in clusters. However, cells related to hair-follicles, or also monocytes, which should be present in IMQ-inflamed skin are missing. Could one of the clusters (M0 or M1) also contain/encompass or represent cells related to monocytes?

Reply: This is a very valid concern raised by the reviewer. Single-cell subpopulations are annotated according to marker genes, and marker genes of some cells, like hair-follicles cells or monocytes, may be included in other cell subpopulations because of their expression.

Feature plot (A) and violin plot (B) show that *Krt15*, hair-follicles cell marker, is mainly expressed in Basal Keratinocytes (K1) and *Krt79*$^{+}$ Keratinocytes (K2).

Feature plot (C) and violin plot (D) show that *Ly6c2*, monocytes marker, is mainly expressed in *Plac8*$^{+}$ Macrophages (M0), Neutrophils (M3) and *Ifng*_hi Natural Killer T cells (L1).

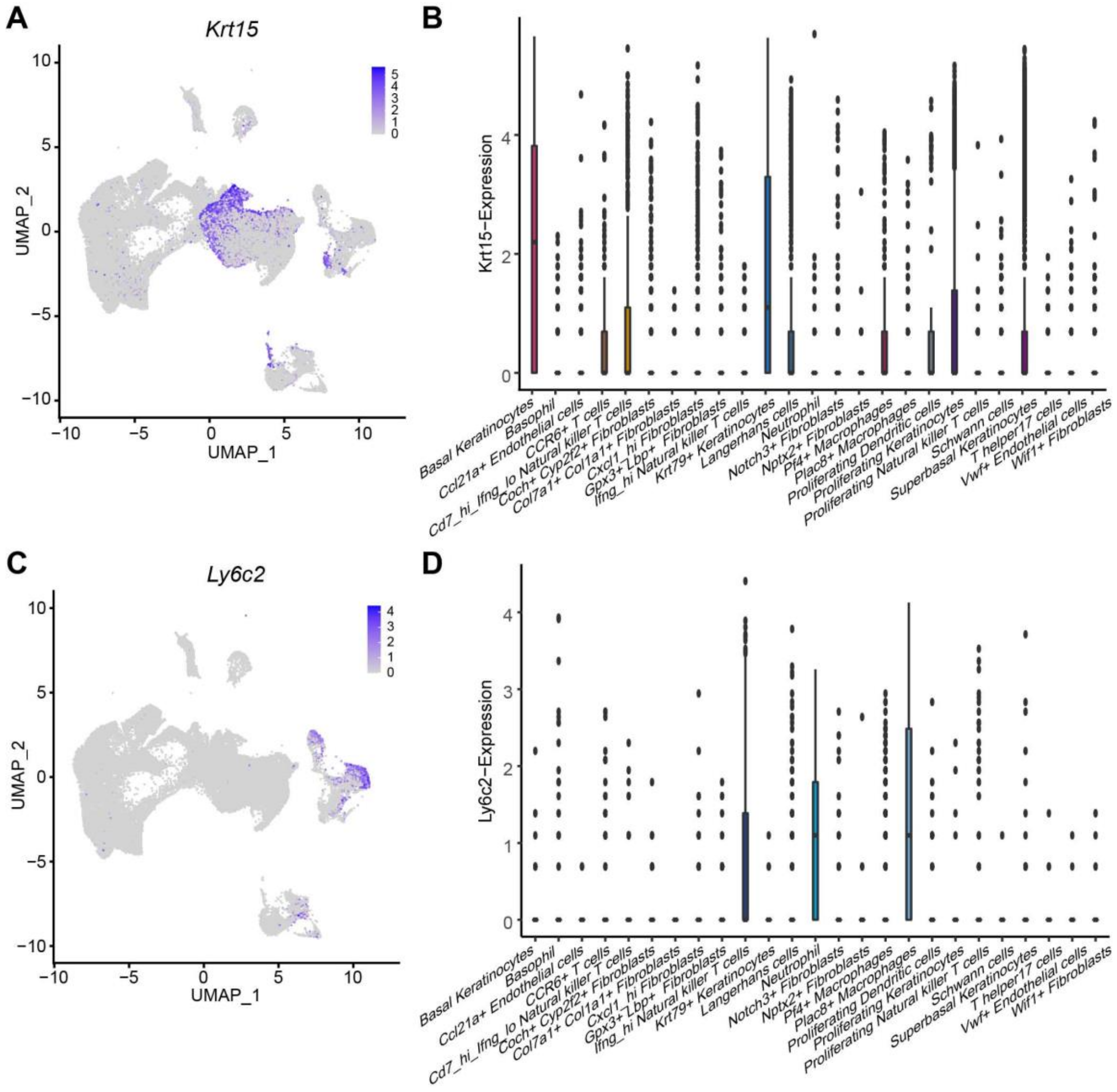


Response Figure 7. *Krt15* and *Ly6c2* expression in all clusters of mouse skin cells.

(A and C) Feature plots of expression distribution for *Krt15* (A) or *Ly6c2* (C). Expression levels are color-coded and overlaid onto UMAP plot.

(B and D) Violin plot showed the distribution of the normalized expression level of *Krt15* (B) or *Ly6c2* (D).

5) The analysis for KEGG pathways and GSEA etc. (Fig. 4) have been performed on the whole myeloid cell subset but not on subclusters. Performing such analysis on the subclusters could give more insight into the relevance of the individual populations defined.

Reply: We have performed this experiment and supplemented these results in Appendix Fig S8. KEGG pathways analysis of M0 and M1, and GSEA analysis of M0, are consistent with the results of the analysis on whole myeloid cell subset.

We have updated these data in **Appendix Fig S8** in our revised manuscript.

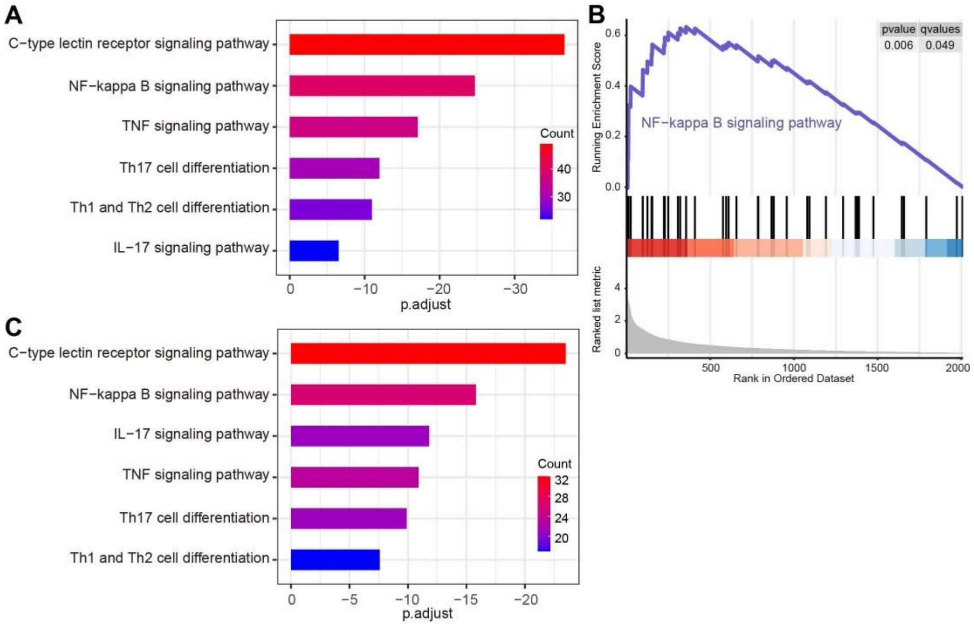


Response Figure 8. Pathway enrichment analysis in M0 and M1.

(A and C) Representative KEGG pathways enriched in upregulated genes in M0 or M1. (B) Gene set enrichment analysis (GSEA) of the NF-kappa B signaling pathway in M0.

6) Additional information on SHP2 expression (and other family members), for example in the scRNA Seq data for the specific cell populations would be important.

Reply: It is a very nice advice. To follow the reviewer's suggestion, we analyzed the expression of *Ptpn1* (encoding PTP1B), *Ptpn6* (encoding SHP1), *Ptpn11* (encoding SHP2) in 25 subpopulations of mouse skin. It was observed that *Ptpn1* was expressed in most cells, *Ptpn6* was expressed only in immune cells, while *Ptpn11* was mainly expressed in keratinocytes, fibroblasts, endothelial cells and immune cells.

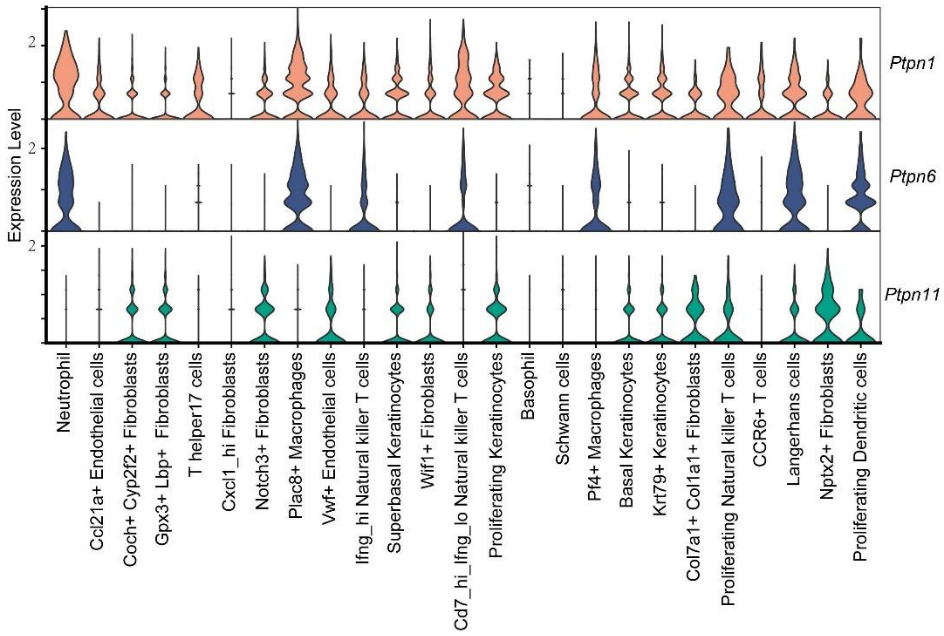

Response Figure 9. *Ptpn1*, *Ptpn6* and *Ptpn11* expression in all clusters.
Violin plot showed the distribution of the normalized expression level of *Ptpn11*.

SHP2 is pro-inflammatory following TLR7 triggering in macrophages, but what about its role in TNF-α or IL-22 signalling (two prominent cytokines in psoriasis).

Reply: This is a valid concern. According to our experimental results, SHP099, a potent allosteric inhibitor of SHP2 (Chen *et al*, 2016, PMID: 27362227), significantly inhibited the expression of *Il22* (A) and *Tnfa* (B) in mouse skin. *Tnfa* expression was significantly decreased in the dorsal skin, BMDMs and PMs of Mø-*Shp2*$^{-/-}$ mice than in wild-type mice. Similarly, *Tnfa* expression was significantly reduced in THP1 cells with knockdown SHP2 than in controls. Overall, inhibition or knockdown of SHP2 in the animal model and cells indeed down-regulates TNF-α and IL-22 signalling.

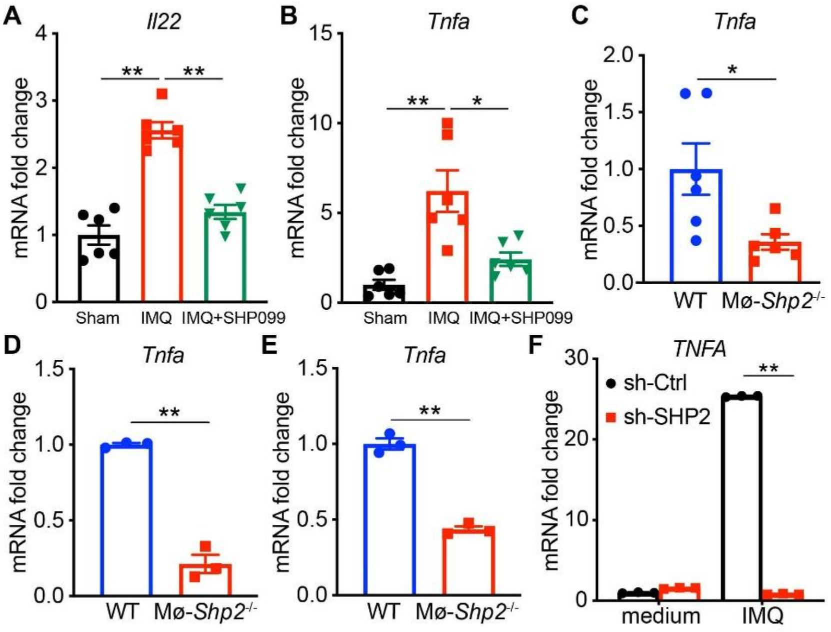


Response Figure 10. Effect of inhibition or knockout of SHP2 on IL22 and TNF-α.
(A and B) Quantitative PCR analysis of *Il22* (A) or *Tnfa* (B) in the dorsal skin of C57BL/6 mice treated with indicated dose of SHP099 or vehicle for 4 days. (C–E) Quantitative PCR analysis of *Tnfa* in dorsal skin (C) or BMDMs (D) or PMs (E) from wild-type and Mø-*Shp2*$^{-/-}$ mice. Results were normalized to *Gapdh* expression (A–E).
(F) Quantitative PCR analysis of *TNFA* using PMA-differentiated THP-1 cells with shRNA-Control or shRNA-SHP2 lentivirus stimulated with IMQ (10 μg/ml) for 6 h. mRNA levels were normalized relative to *ACTIN*.
Data represent mean ± SEM. *P* values are determined by two-tailed Student's *t* test (A–E) or Tukey multiple comparison test (F). **P*<0.05, ***P*<0.01.

Does SHP2 have a pro- or maybe even anti-inflammatory role?

Reply: This is one of the big questions we've been exploring. The role of SHP2 in inflammatory diseases is well documented, as it acts as both an anti-

inflammatory and a pro-inflammatory regulator of inflammation. Previously, it was found that upon stimulation with NLRP3 inflammasome activator, SHP2 translocates to mitochondria and then interacts with ANT1, an inner mitochondrial membrane protein, and dephosphorylates ANT1 to maintain mitochondrial homeostasis (Guo *et al*, 2017, PMID: 29255148). At this point, SHP2 acts as an anti-inflammatory factor to negatively regulate the activation of NLRP3 inflammasome. In inflammatory bowel disease (IBD), the phosphatase SHP2 exacerbates IBD by disrupting IL-10-STAT3 signaling in macrophages and its dependent anti-inflammatory response (Xiao *et al*, 2019, PMID: 30610104). SHP2 acts as a pro-inflammatory factor that contributes to the development of SLE (Wang *et al*, 2016, PMID: 27183387). In the present study, it is suggested that SHP2 is a positive regulator of psoriasis progression. In infiltrated macrophages of psoriatic skin, SHP2 is overexpressed. Hyper-dephosphorylation of TLR7 at Tyr1024 by SHP2 leads to hyper-ubiquitination of TLR7 and overactivation of NF-*κ*B activation, resulting in unrestrained skin inflammation. In conclusion, the pro- or anti-inflammatory role of SHP2 in inflammatory disorders is substrate protein-dependent and disease-dependent, which involves a complex balance between multiple immune cells and cytokines and chemokines. Thanks a lot for your comments.

7) A number of Western Blots claim changes in e.g. p-IKKa/b, p-p65 or p-Tyr. However, it is sometimes difficult to see the subtle differences. The authors should quantify the bands and normalize them to total protein expression and depict the changes as numbers above the respective graph in all cases where quantitative statements are made. Ideally, the average of more than one experiment should be shown.
Reply: We appreciate your constructive advice. According to the reviewer’s suggestion, we have displayed and grayed out the original strips of Western Blots that appear in the manuscript.

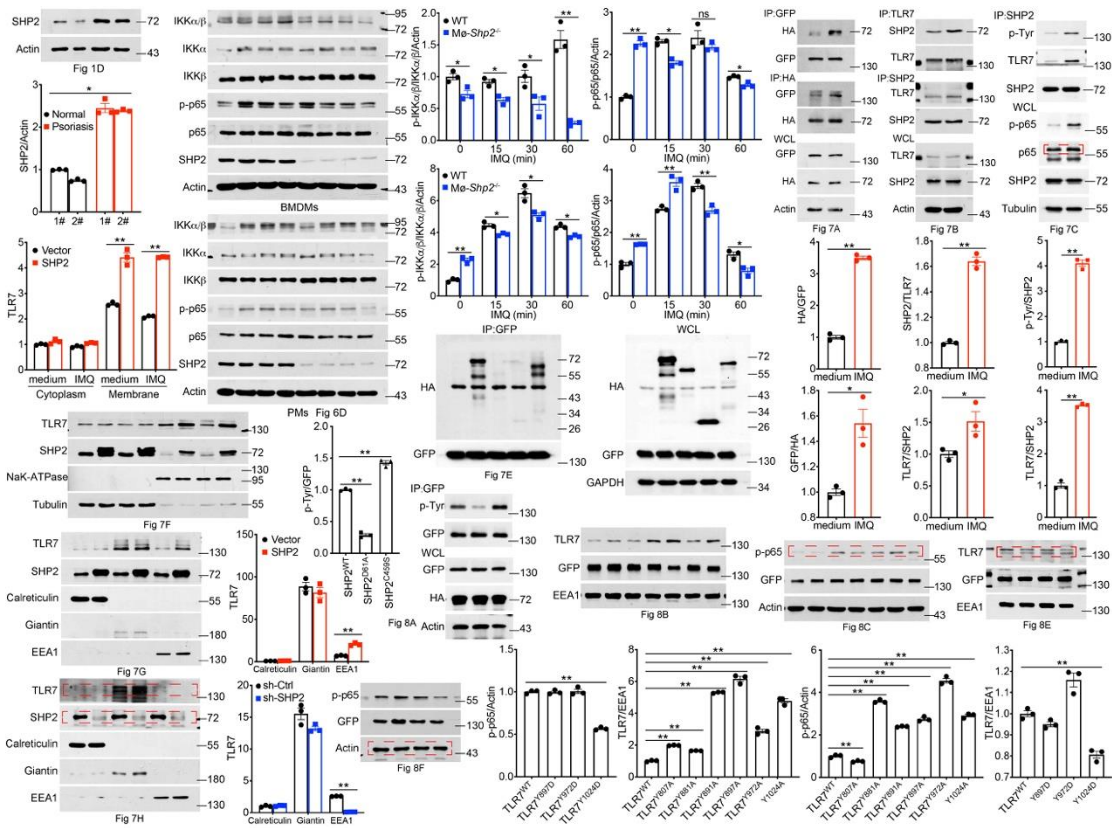

Response Figure 11. Raw data for Figure 1, Figure 6, Figure 7 and Figure 8. Panels corresponding to the figures in the manuscript are indicated.

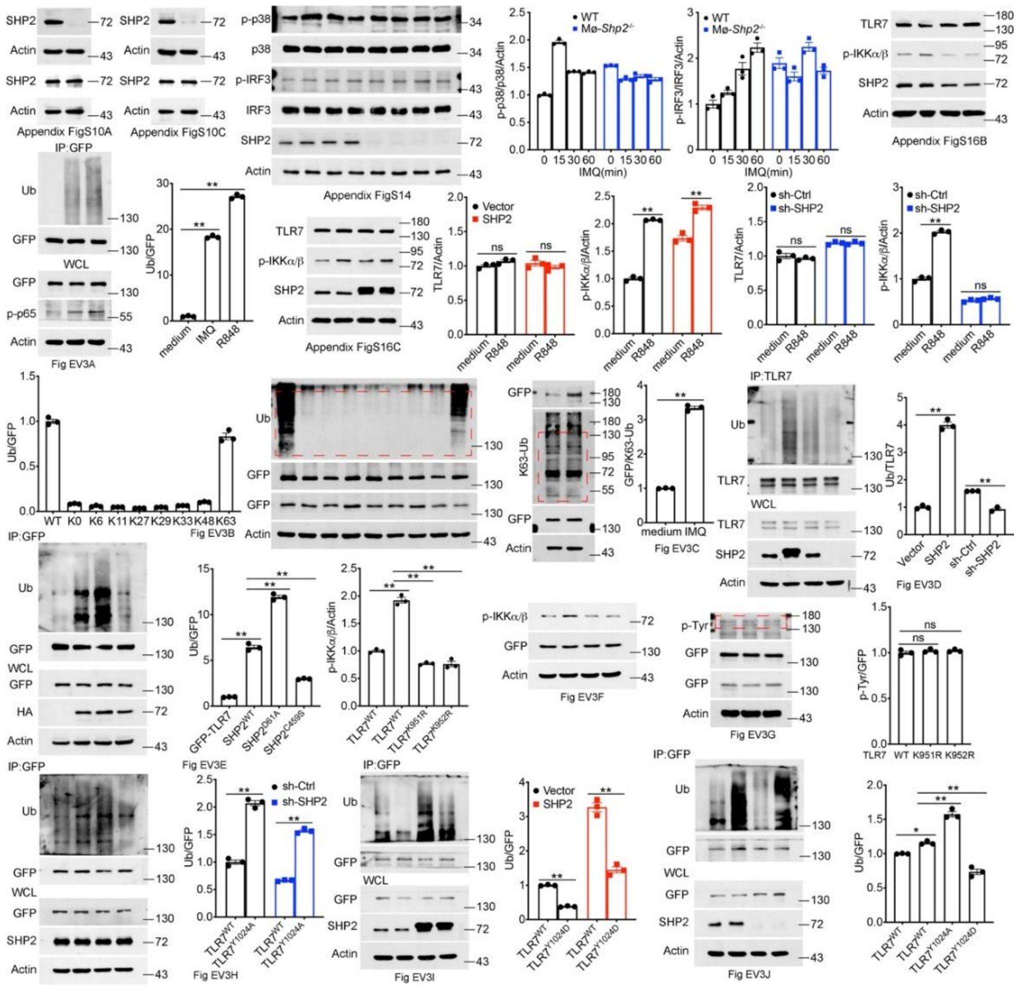


Response Figure 12. Raw data for Figure EV3, Appendix Fig S10, Appendix Fig S14 and Appendix Fig S16. Panels corresponding to the figures in the manuscript are indicated.

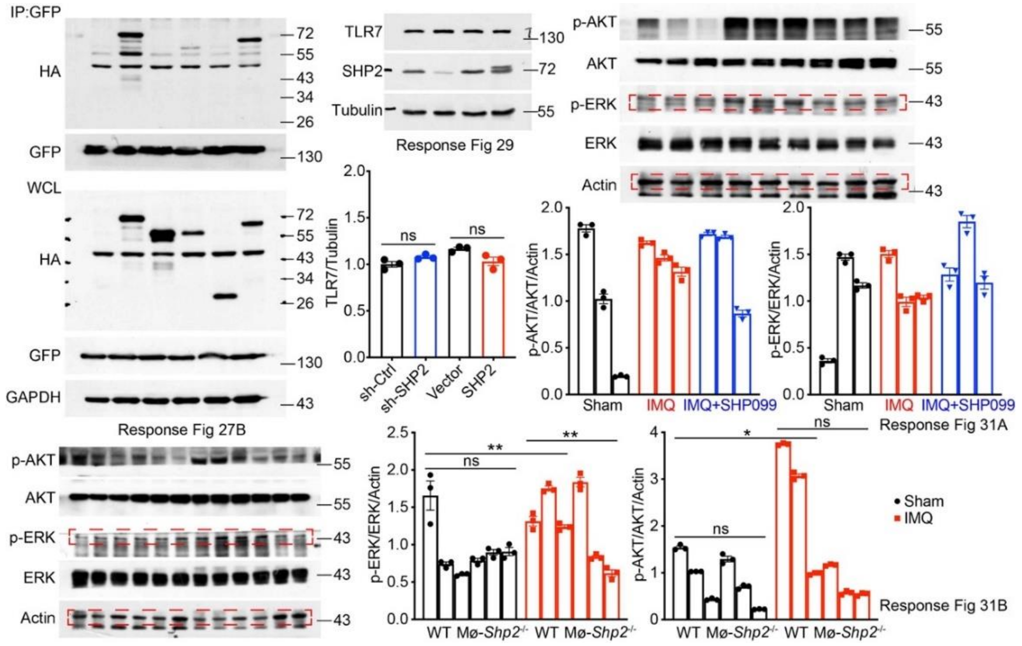

Response Figure 13. Raw data for Response Figures. Panels corresponding to the figures in the respond letter are indicated.

8) Figure 6. Shows that SHP2 controls NF-KB activation. However, other signaling pathways relevant for inflammatory cytokine production are also activated by TLR7 stimulation (IRF and MAPK signaling) and should be at least discussed.

Reply: We followed the reviewer's comments, and demonstrated significantly higher phosphorylation levels of p38 and IRF3 in peritoneal macrophages from Mø-*Shp2*$^{-/-}$ mice at 0 min. However, after IMQ stimulation, the levels of p-p38 and p-IRF3 were not differentially expressed in the peritoneal macrophages of the Mø-*Shp2*$^{-/-}$ mice and wild-type mice.

We have updated these data in **Appendix Fig S14** in our revised manuscript.

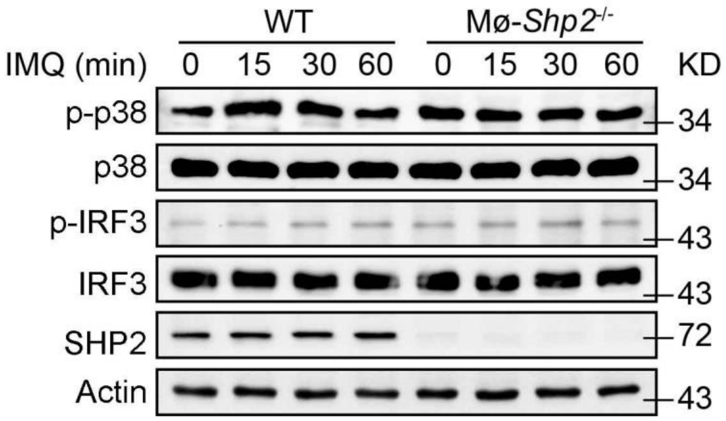


Response Figure 14. SHP2 did not affect the IRF and MAPK signaling in macrophages in response to IMQ stimulation.

PMs derived from wild-type and Mø-*Shp2*$^{-/-}$ mice were stimulated by IMQ (10 µg/ml) for indicated times. Whole cell lysates were subjected to Western blotting.

9) The authors nicely analyze the signaling pathways with overexpression studies combined with mutated versions of the involved proteins in THP-1 and HEK 293 cells. These are important studies to mechanistically dissect the pathway. However, to strengthen the relevance of their findings, they need to be verified also in the primary cells claimed to be involved in the etiology of the disease (in the case here, in macrophages or other myeloid cells).

Reply: This is a very helpful suggestion. In fact, the data of BMDMs and PMs derived from Mø-*Shp2*$^{-/-}$ mice and wild-type mice demonstrated that SHP2 deficiency in macrophage mitigates psoriasis by suppressing NF-κB activation (A). We also demonstrated in BMDMs that SHP2 can bind to phosphorylated TLR7 (B).

We have updated Response Figure 15B in **Figure 7C** in our revised manuscript.

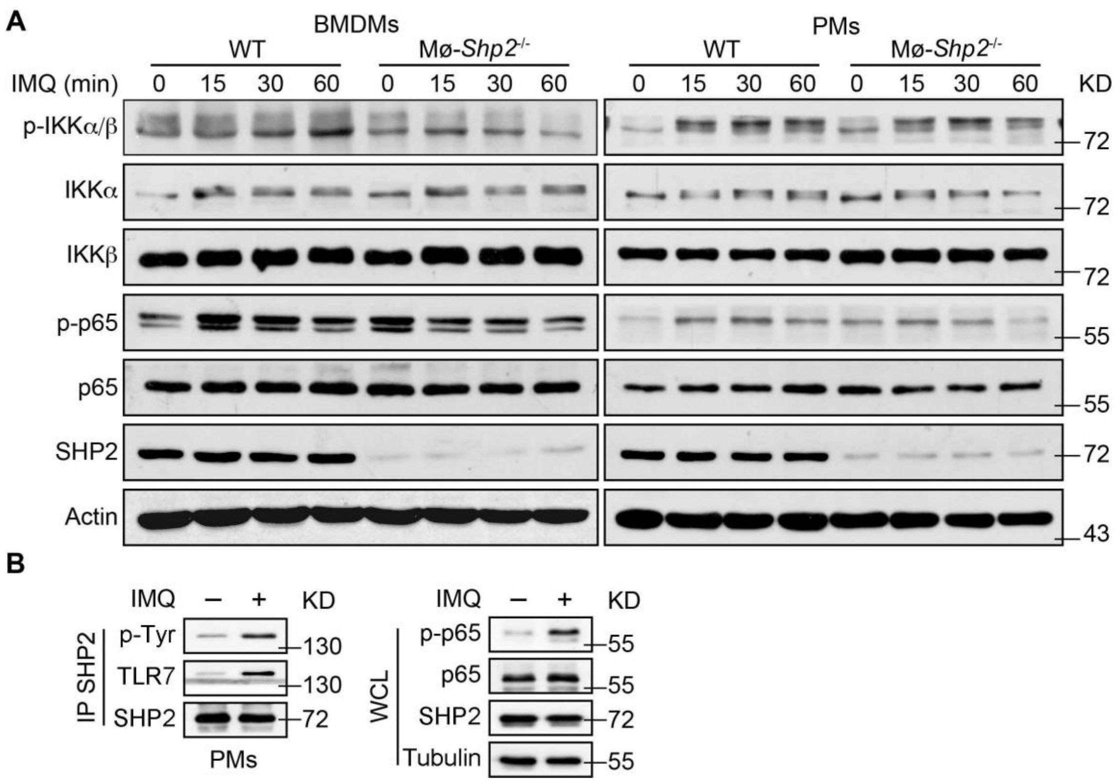


Response Figure 15. Experiments carried out on primary cells.

(A) BMDMs and PMs derived from wild-type and Mø-*Shp2*$^{-/-}$ mice were stimulated by IMQ (10 μg/ml) for indicated times. Whole cell lysates were subjected to Western blotting.

(B) BMDMs derived from wild-type mice were stimulated by IMQ (10 μg/ml) for 30 min. Cell lysates were immunoprecipitated by anti-SHP2 and probed by indicated antibodies.

10) Figure 8B: Why does TLR7 protein amount not correlate to GFP amount? Have these cells not been stimulated with IMQ? If not, how does IMQ stimulation affect the expression of the different mutants? In Figures 8 and S16 mainly GFP is used to detect TLR7 protein. However, in Fig. 16F suddenly anti-TLR7 antibody is used. Why that? and is there a difference between the two stainings, e.g. doers GFP expression reflect TLR7 levels?

Reply: We apologize for the confusion and would like to provide the following explanation. In Fig 8B and Fig 8E, the expression of GFP in whole cell lysates was detected to verify the transfection efficiency of GFP-TLR7 or mutant vectors. For TLR7 and EEA1, the expression of endosomes was detected, where EEA1 is the marker for endosomes and is used here as a normalized protein. These cells have been stimulated with IMQ, and we have added this in the legends.

In Fig. S16F, we regret that the figure legend was written incorrectly. We used SHP2-deficient THP-1 cells or SHP2-overexpressed THP-1 cells, without transfecting any plasmids, so that only endogenous TLR7 could be IP.

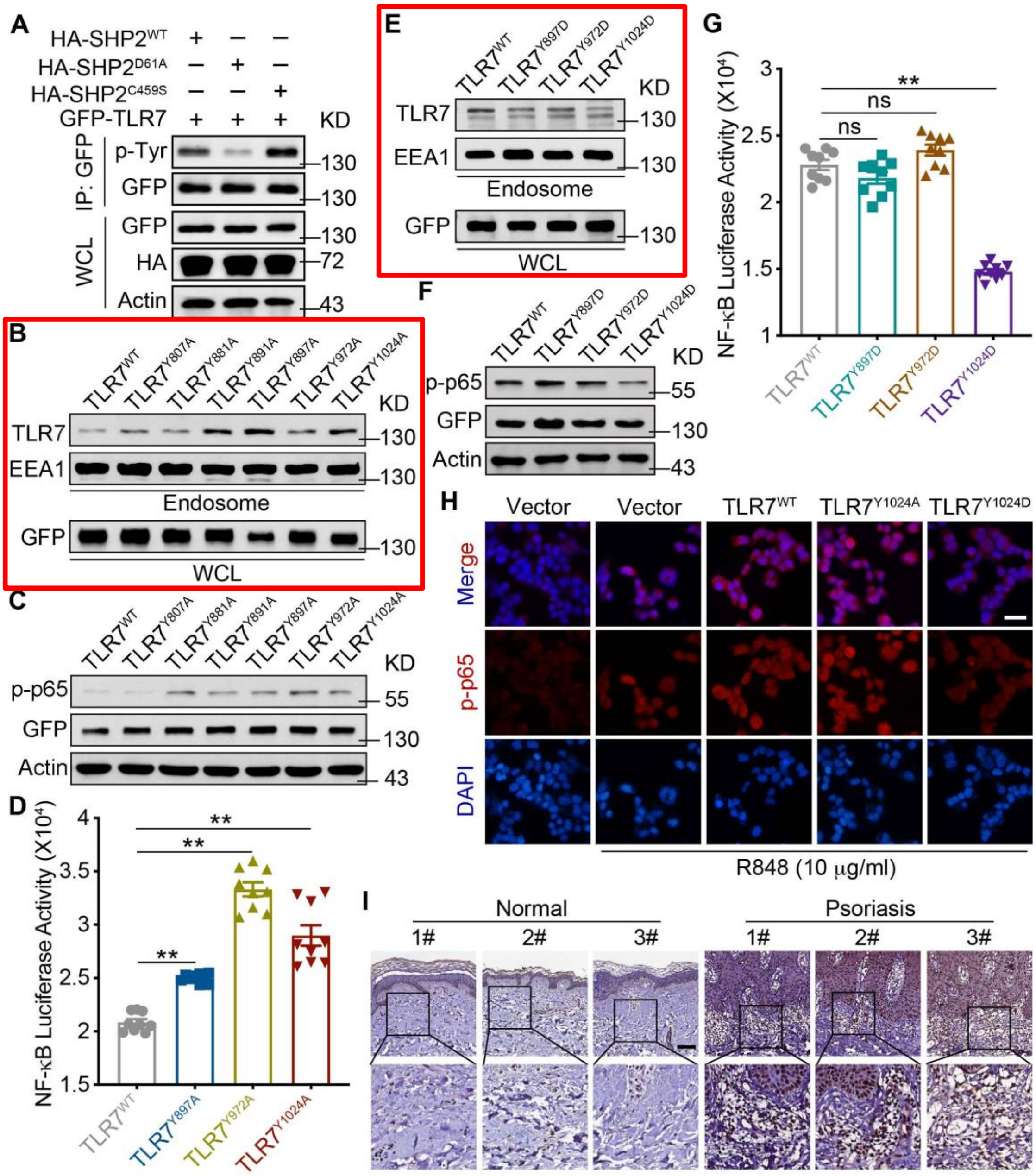


Figure 8. SHP2 enhanced activation of TLR7/NF-*κ*B signaling in a phosphatase-dependent manner.

(A) Immunoblotting of HEK293T cells co-transfected for 48 h with GFP-TLR7, plus HA-tagged SHP2 or SHP2 mutant vectors, followed by immunoprecipitation with anti-GFP.

(B and E) Immunoblotting of TLR7 in the endosome isolated from HEK293T cells that were transfected with GFP-TLR7 or GFP-TLR7 mutant vectors.

(C and F) Immunoblotting of p-p65 in lysates of HEK293T cells transfected with GFP-TLR7 and TLR7 mutant vectors and then treated with IMQ (10 μg/ml) for 30 min.

(D and G) HEK293T cells transfected with NF-*κ*B-luciferase reporter and indicated TLR7 mutant vectors and then treated with IMQ (10 μg/ml) for 24 h were harvested for luciferase assay.

(H) Immunofluorescence staining of HEK293T cells transfected with GFP-TLR7 and TLR7 mutant vectors, prior to treatment of R848 (10 μg/ml) for 30 min, and labelled with specific antibodies. Scale bar: 50 μm.

(I) Representative p-TLR7 Y1024 staining of skin sections from human. Scale bar: 100 μm.

Data represent the mean ± SEM. *P* values are determined by two-tailed Student's *t* test (D and G). $^{**}P<0.01$, ns, not significant.

11) Figure 7G, H: Can the authors explain, why SHP2 protein can be found at equal amounts in each sub-cellular compartment?

Reply: Thank you for this comment. We used SHP2-overexpressed THP-1 cells in Fig 7G and SHP2-deficient THP-1 cells in Fig 7H, which were not fully representative of what happened in the physiological state.

12) Figure S14: Although it is clear that there is a difference between WT and M-SHP2-/- peritoneal macrophages in respect of the co-localization of TLR7 and EEA1 at the 0 min time point, it rather seems that at later time points there is less co-localization in WT cells. Changes seem to mainly result from the EEA1 staining. This is different to the data in THP1 (Figure 7C) where there is at least stable co-localization and the difference might be attributed to the anti-mouse TLR7 antibody, which also seems to stain the nucleus. However, in any case it is difficult to draw conclusions from these pictures. This should be clarified. Is it possible to perform flow cytometric analysis at least from the human cells?

Reply: We thank the referee for this comment. Given the poor quality of the IF images in Fig. S14, we removed this part of the data, and the previous data are sufficient to support our conclusion. Your advice about flow cytometric analysis of human cells is a constructive suggestion. Of course, we will absolutely adopt your suggestion in future research. Thank you again.

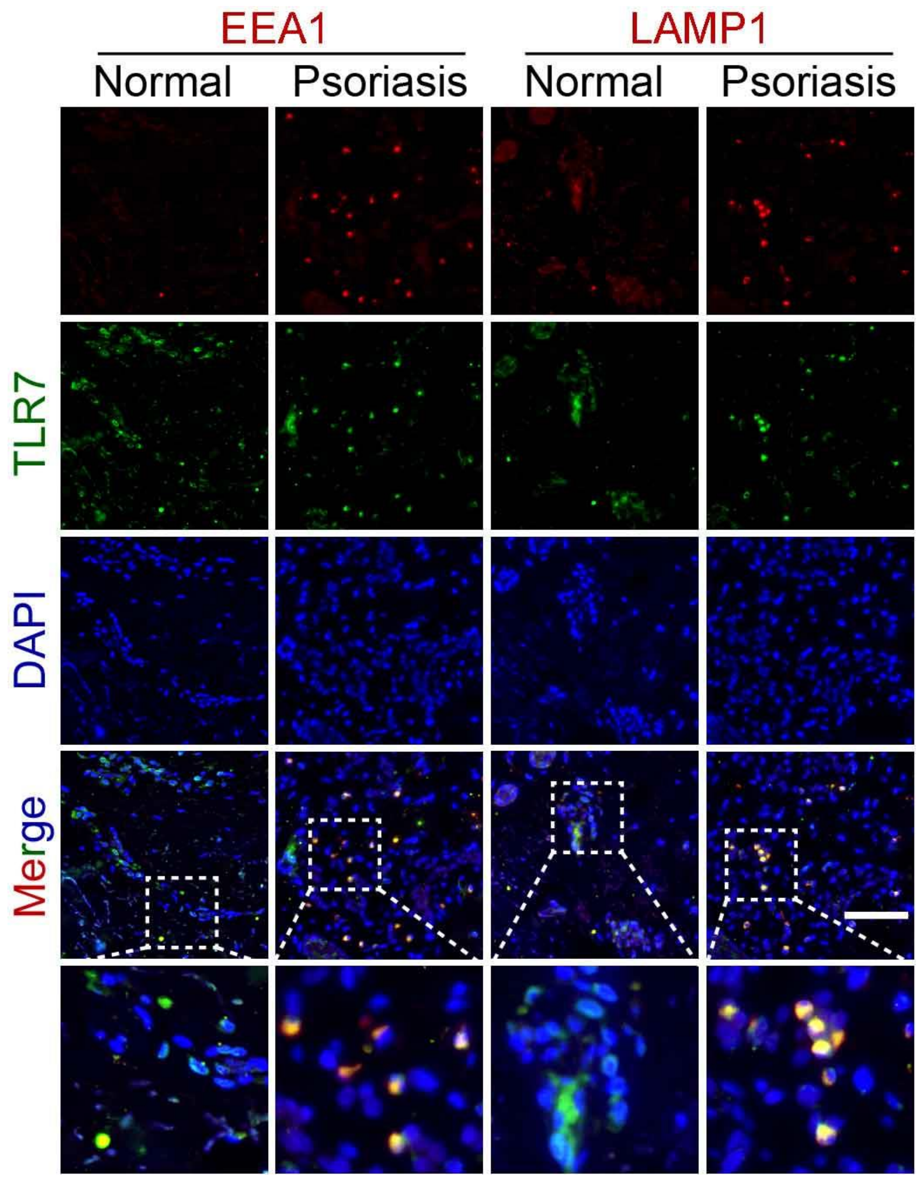


Figure 7I. Skin sections from psoriatic patients and healthy controls were stained for TLR7 together with a marker of the early endosome (EEA1) or late endosome/lysosome (LAMP1) prior to analysis by confocal microscopy. Scale bar: 50 μm.

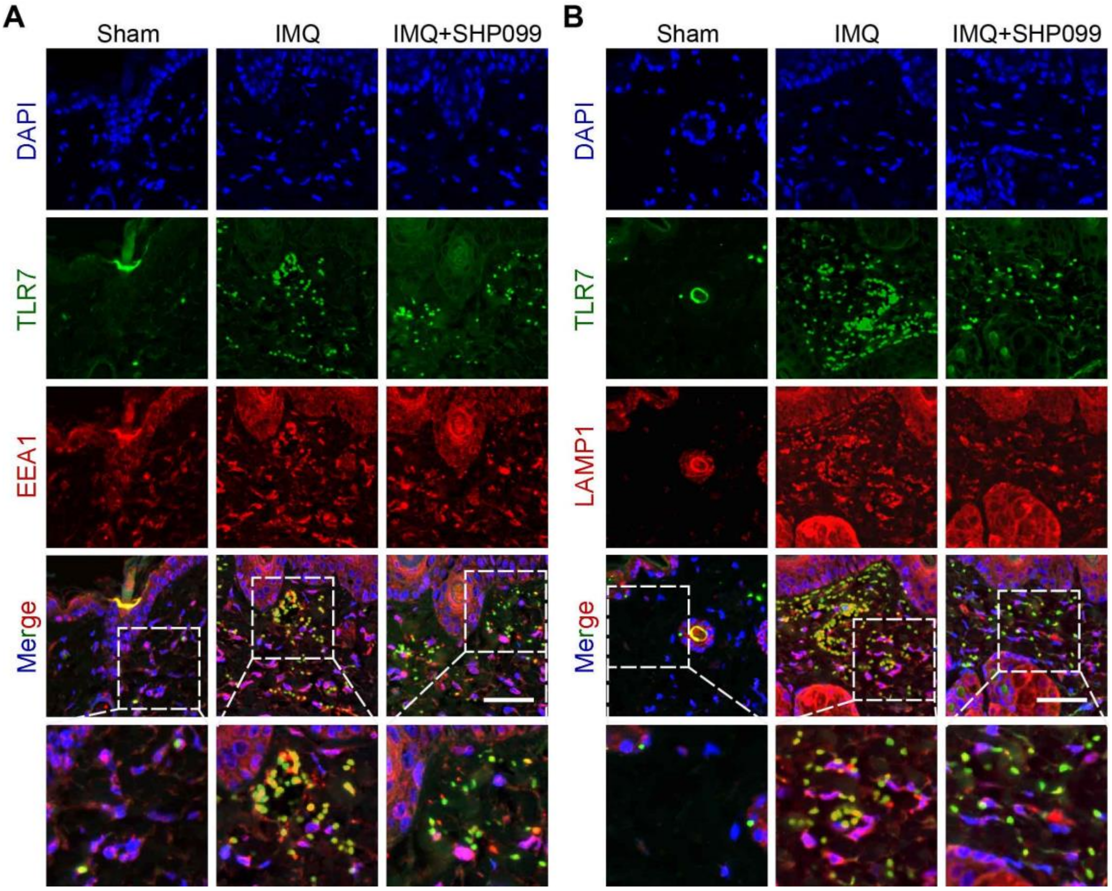


Appendix Fig S19. The localization of TLR7 in the endosome was decreased in SHP099-treated group.

Confocal microscopy imaging of skin sections from indicated mice labelled with anti-TLR7 (green), anti-EEA1 (red) or anti-LAMP1 (red) and DAPI (blue), showing TLR7 expression on early or lately endosome, respectively. Scale bar: 50 μm.

13) The quality of some of the IF images is poor and does not allow following the conclusions of the authors. It is necessary to increase the resolution of the confocal images and to show enlarged areas of cells indicated by arrows, at least for the merged images. This is even more important since the authors want to demonstrate co-localizations at the subcellular level (e.g. Figure 7I, S13).

Reply: We are sorry for some poor IF images in the original manuscript and have updated the images.

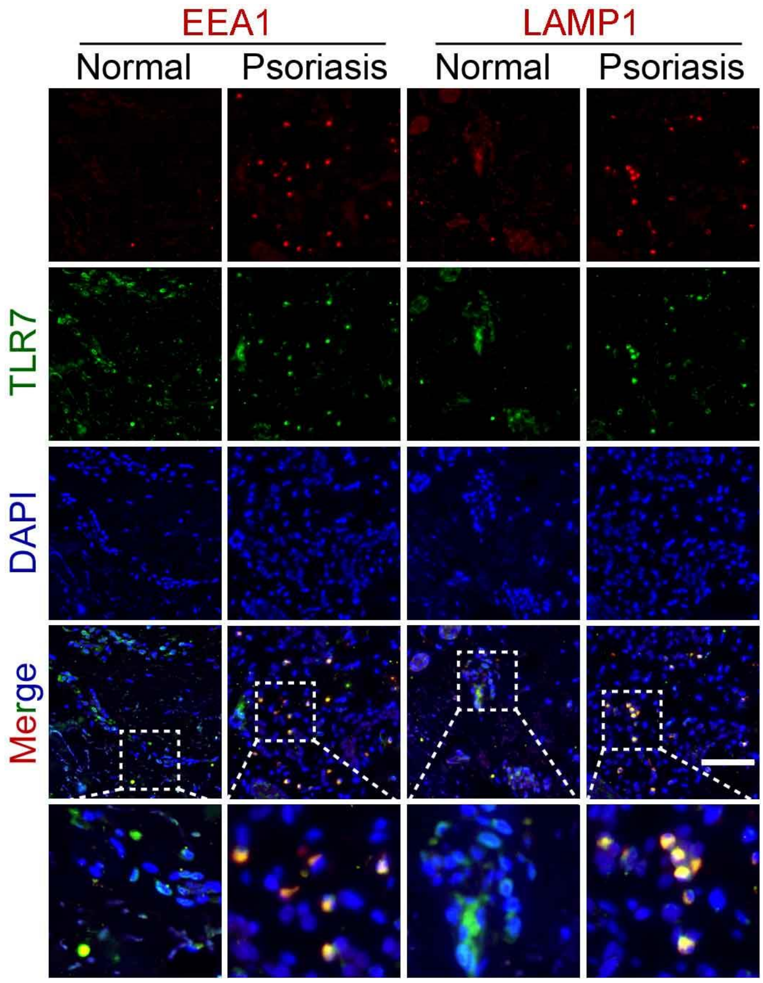


Figure 7I. Skin sections from psoriatic patients and healthy controls were stained for TLR7 together with a marker of the early endosome (EEA1) or late endosome/lysosome (LAMP1) prior to analysis by confocal microscopy. Scale bar: 50 µm.

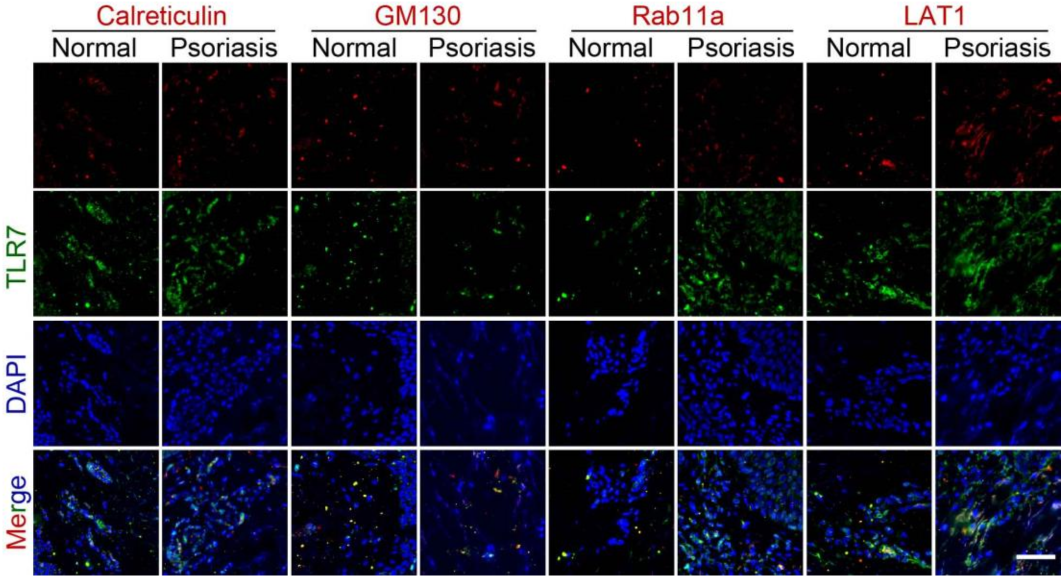


Appendix Fig S18. Cellular localization of TLR7 in psoriatic skin.

Skin sections from psoriasis patients and healthy controls were stained for TLR7 together with a marker of the ER (Calreticulin), Golgi (GM130) and recycling endosome (Rab11a) or cell membrane (LAT1) prior to analysis by confocal microscopy. Scale bar: 50 μm.

A number of these staining rely on the quality of the TLR7 staining, which, however, is often not convincing at all (e.g. Figure 7J, I; S14: here TLR7 staining is found in the nucleus?).

Reply: We are sorry for this confusion. Regarding Fig S14, given the poor quality of the IF images, we removed this part of the data, and other data are sufficient to support our conclusion.

TLR7 knock-out skin should be used to control for the specificity of the anti-TLR7 antibody employed here.

Reply: Thank you for your advice. We used B6.129S1-*Tlr7*$^{tm1Flv}$/J mice (TLR7 knock-out mice) from Jackson Laboratory (JAX: 008380) to confirm the specificity of the anti-TLR7 antibody.

Immunohistochemical staining demonstrates the specificity of TLR7 antibody.

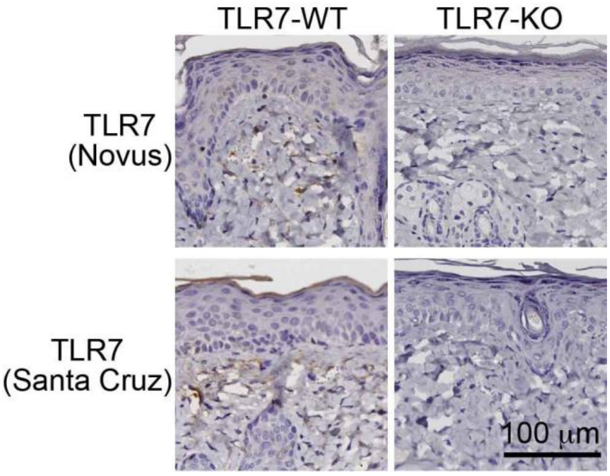


Response Figure 16. Validation of TLR7 antibody specificity.

Representative TLR7 staining of the dorsal skin from TLR7-WT and TLR7-KO mice. Scale bars: 100 μm.

In addition, we applied IMQ to the dorsal skin of wild-type mice (TLR7-WT) and *Tlr7*-knockout mice (TLR7-KO), and the TLR7-KO mice showed reduced morbidity, including scaling, erythema, epidermal thickness, clinical scores, weight changes, and expression of psoriasis-associated inflammatory factors.

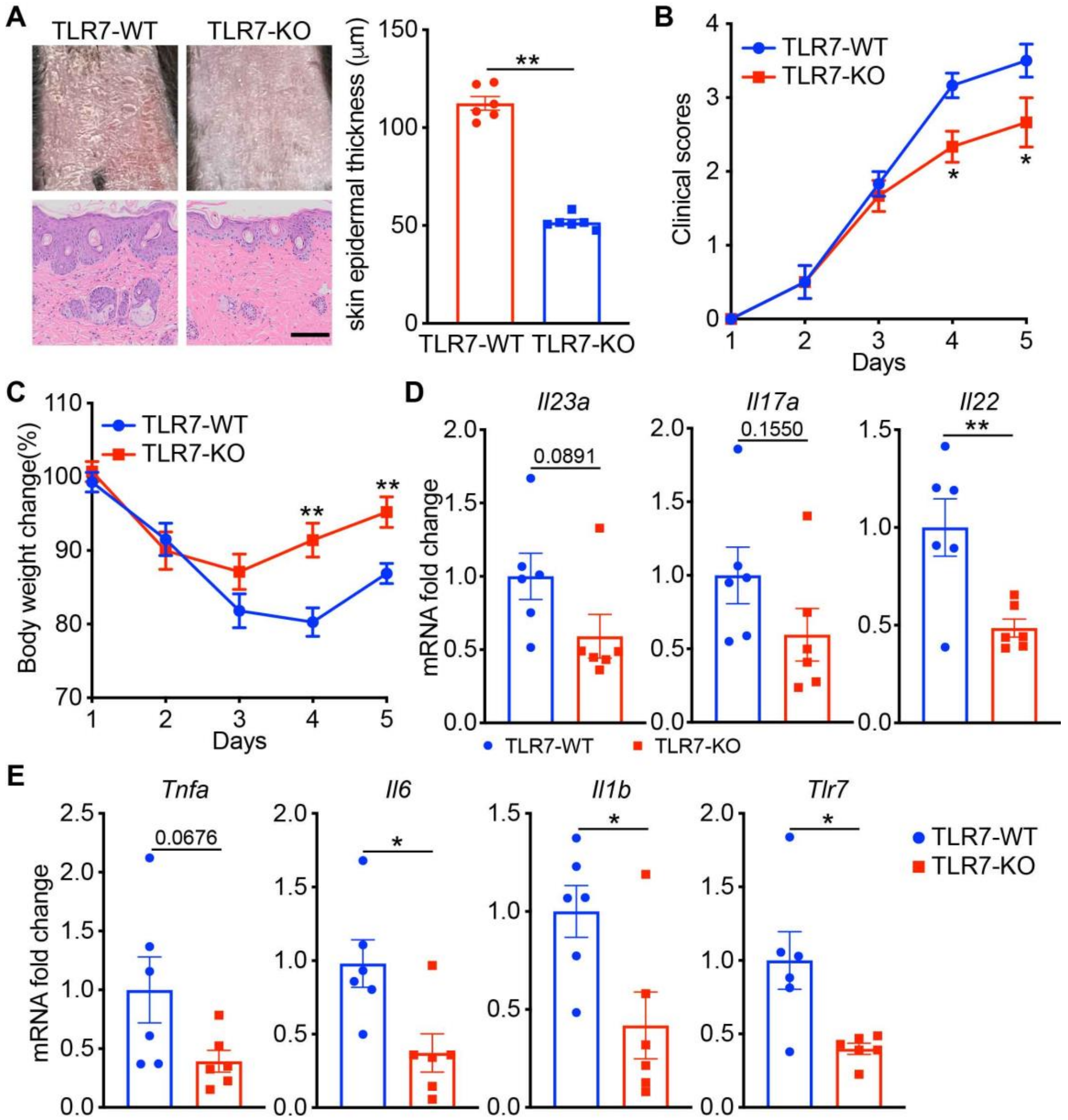


Response Figure 17. TLR7 deficiency alleviated psoriasis-like phenotype in the IMQ-induced murine model.

TLR7-WT female mice (*n*=6) and TLR7-KO female mice (*n*=6) were treated with indicated dose of IMQ for 4 days.

(A) Phenotypic presentation (*top*) and H&E staining (*bottom*) of dorsal skin. Scale bar: 100 μm. Left: H&E staining data; right: statistical data (mean ± SEM).

(B) Clinical scores plotted with mean ± SEM.

(C) Body weight change plotted with mean ± SEM.

(D and E) Quantitative PCR analysis of mRNA encoding IL-23/IL-17A axis cytokines (D) and other psoriasis-related cytokines (E) in the dorsal skin. Results were normalized to *Gapdh* expression.

Data information: Data are represented mean ± SEM. *P* values are determined by two-tailed Student's *t* test (A, D, E) or Bonferroni multiple comparison test (B, C). *$P<0.05$, **$P<0.01$.

14) Additionally, the CD68 staining in Figure S10 is not convincing since there seems to be no macrophages in healthy skin, neither by IHC nor by IF staining.

Reply: Thank you for your suggestion. We have updated the image of Figure S10A to a more representative image that shows expression of CD68 positive cells in the skin of normal healthy donors and psoriasis patients. Actually, in healthy skin, there is little macrophage infiltration in the dermis in the absence of inflammation.

We have updated these data in **Appendix Fig S12** in our revised manuscript.

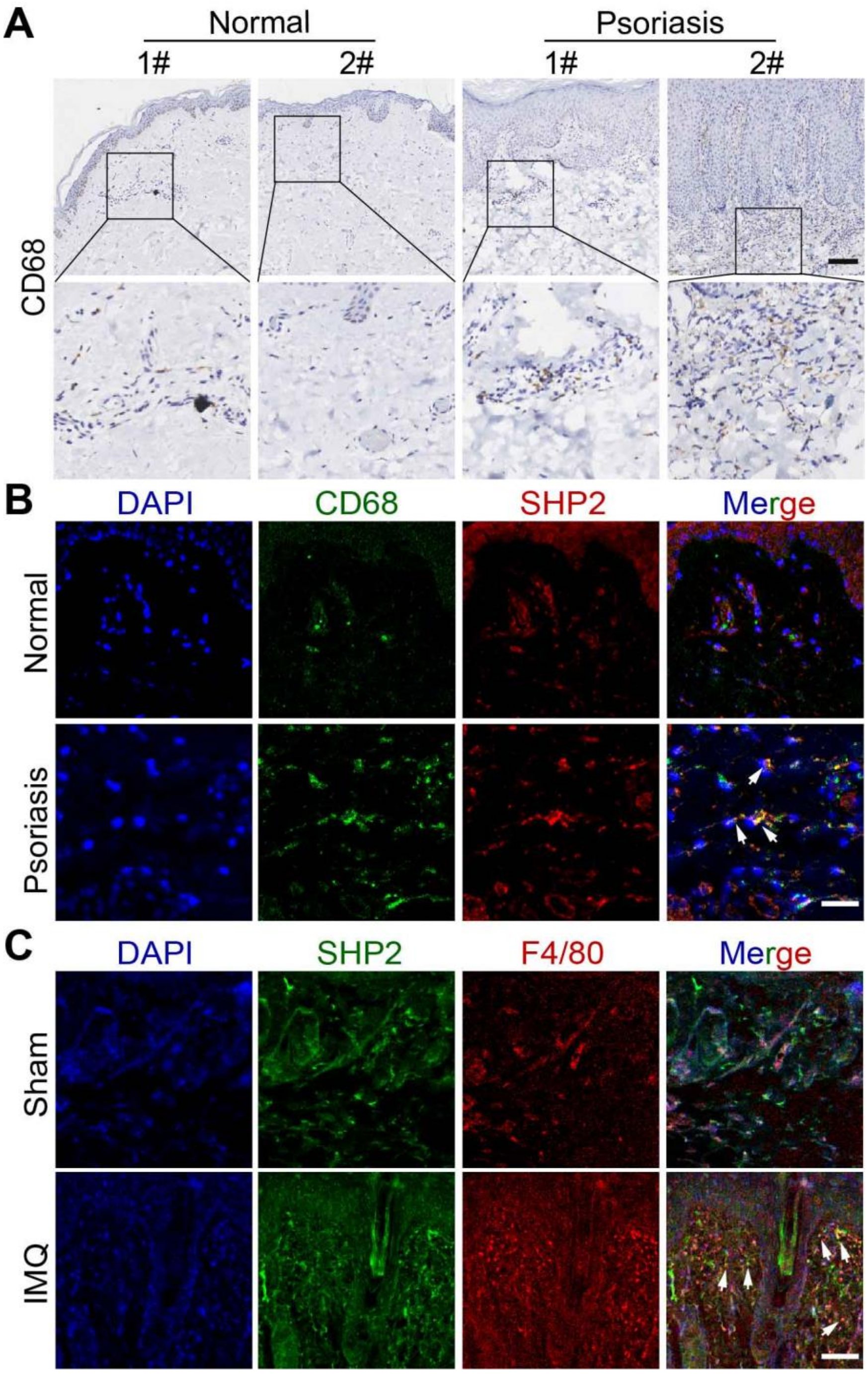

Appendix Fig S12. SHP2 highly expressed in the infiltrated macrophages in both psoriatic skin and IMQ-induced murine skin.

(A) Representative CD68 staining of skin sections from psoriatic patients (*n*=13) and normal controls (*n*=5). Scale bar: 100 μm.

(B) Immunofluorescence staining of skin sections from psoriatic patients and normal controls with SHP2 and CD68, showing SHP2 expression in macrophages (arrows). CD68, green; SHP2, red. Scale bar: 100 μm.

(C) Immunofluorescence staining of skin sections from IMQ-induced and sham mice with SHP2 and F4/80, showing SHP2 expression in macrophages (arrows). SHP2, green; F4/80, red. Scale bar: 100 μm.

15) There is also some confusion about the designation of these 2 Clusters (M2 and M3) in Figure 4, where they are inversely labeled between B and G! Which one is correct? This is also important for understanding Fig S 5B. Even more confusion comes from the fact that the Cluster M5 is missing in Figure S3!

Reply: We thank the reviewer for reading our manuscript carefully and pointing out this mistake. We have corrected the error in the revised manuscript.

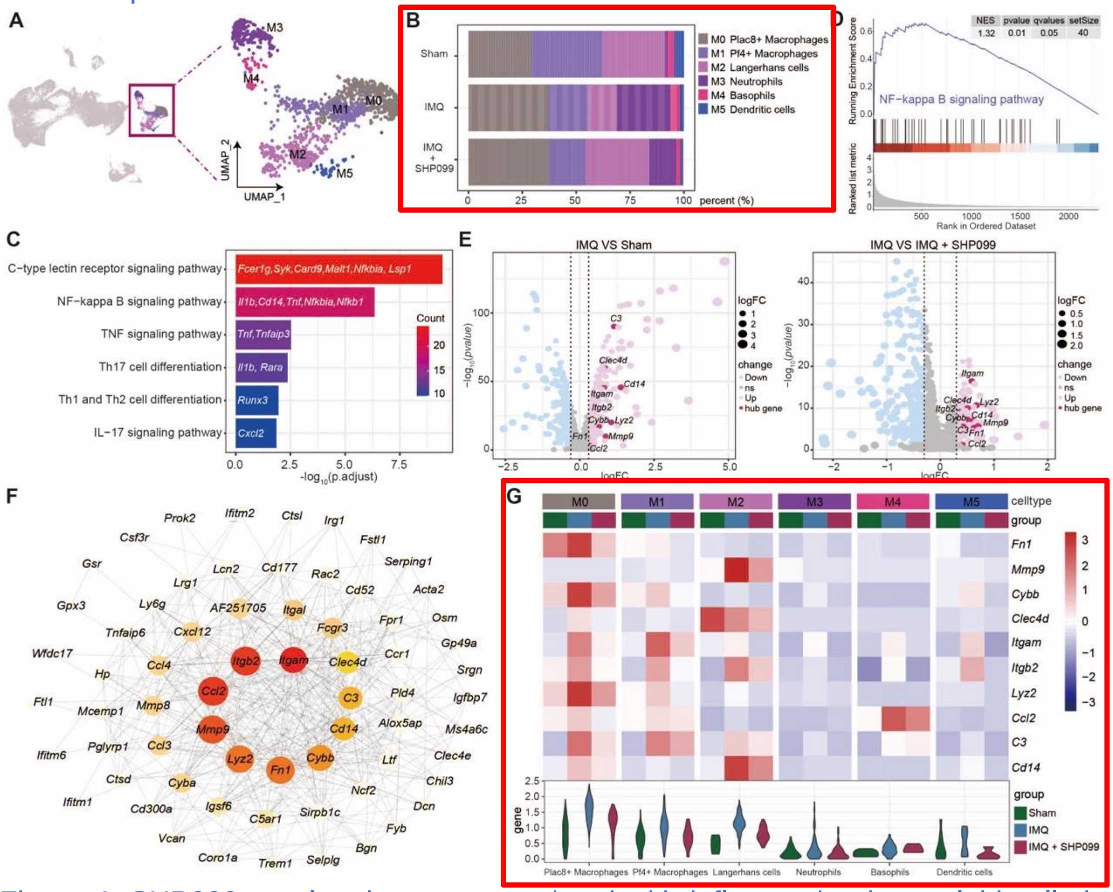

Figure 4. SHP099 regulated genes associated with inflammation in myeloid cells in mouse skin.

(A) UMAP plot of myeloid cells sub-clusters.

(B) Bar plots showing the percentage (%) of cell types in each sample group.

(C) Representative KEGG pathways enriched in upregulated genes in myeloid cells.
(D) Gene set enrichment analysis (GSEA) of the NF-kappa B signaling pathway.
(E) Volcano plots of DEGs that were upregulated (red) or downregulated (blue) in myeloid cells. Examples of DEGs were labeled.
(F) Gene networks of DEGs that were upregulated in the IMQ group compared with the sham and SHP099 groups.
(G) Expression patterns of the top ten hub genes in (F). Heatmap (top) displays the average expression in the three groups and in different myeloid cell subsets. Boxplots (bottom) show the corresponding expression distribution.

We have updated these data in **Appendix Fig S5** in our revised manuscript.

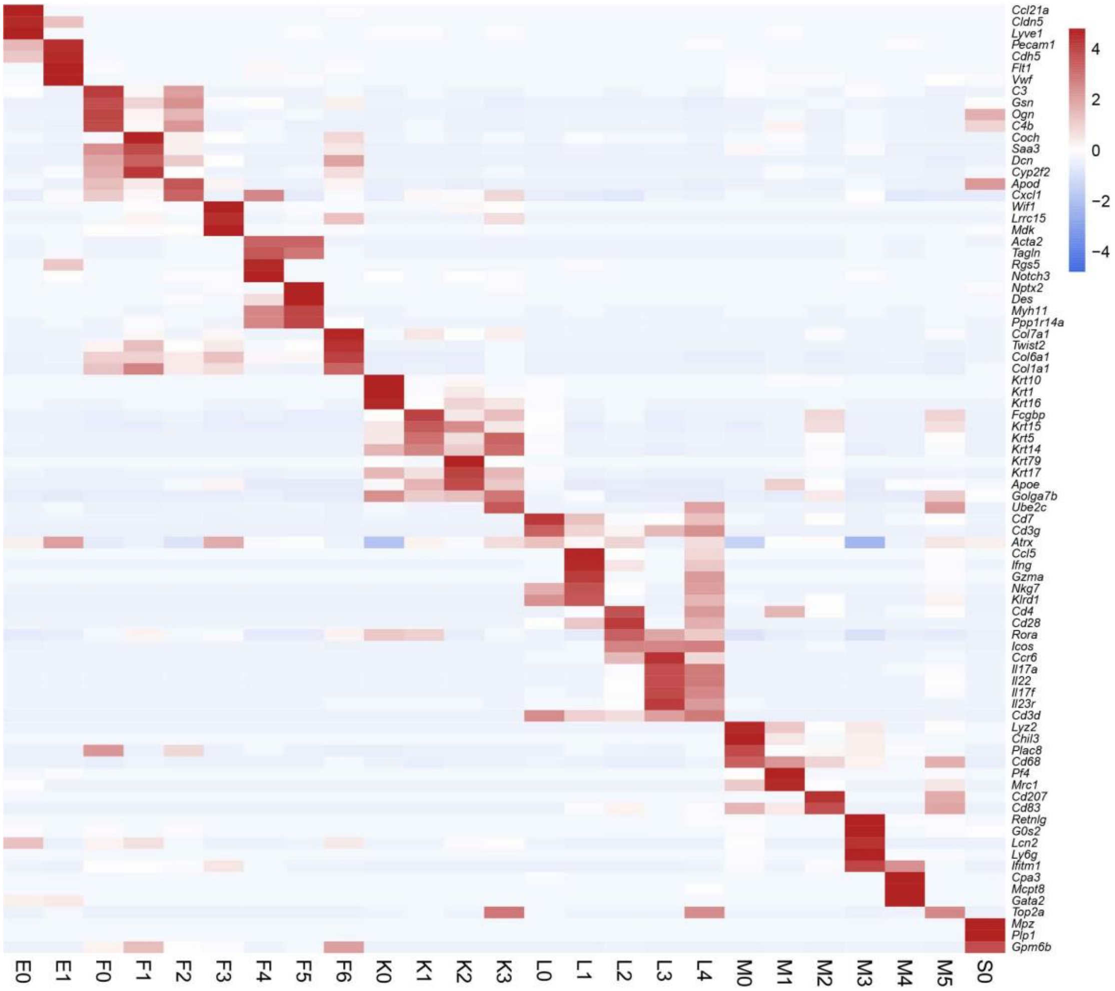

Appendix Fig S3. Distinct gene signatures of mouse skin cell clusters.
Heatmap of the top genes marking mouse skin cell clusters (from Fig. 3 D).

16) Most of the statistical analysis was performed by using Student´s t-test (e.g. cytokine analyses). However, in experiments with more than two experimental groups an ANOVA test with a post test that corrects for multiple testing should be applied!
Reply: Thanks again for reading our manuscript carefully and kindly pointing out this important issue. We have carefully checked and corrected them.

17) Fig 2A. SHP099 alleviates IMQ induced skin inflammation as quantified by epidermal thickness. However, differences in acanthosis are not clearly visible in the hematoxylin and eosin staining shown. These pictures also do not correspond to the quantification shown below, where a reduction in skin thickness of more than 1/3 is shown, irrespective of the dose.

Reply: Thank you for pointing out this error. We have corrected it.

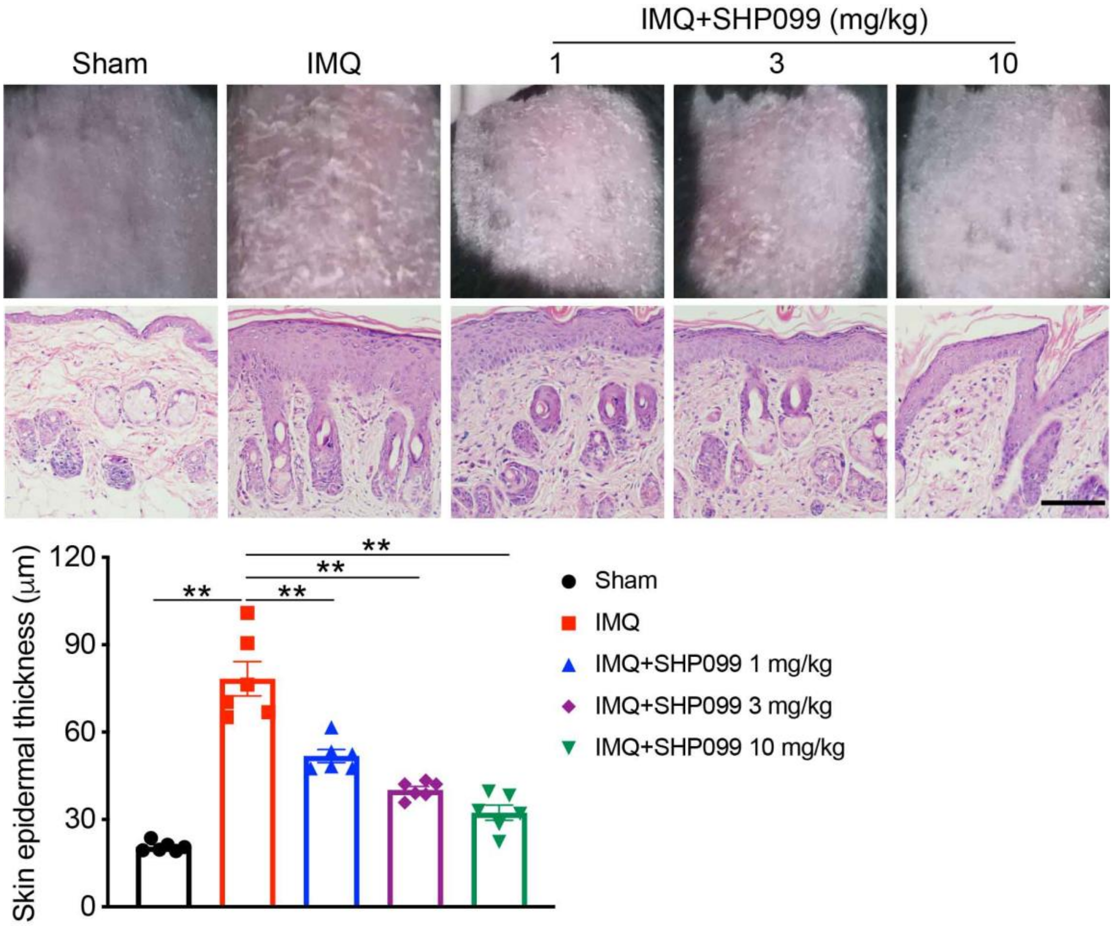


Figure 2A. C57BL/6 mice (*n*=6/group) were treated with indicated dose of SHP099 or vehicle for 4 days. Phenotypic presentation (*top*), H&E staining (*middle*) and statistical data (*bottom*) (mean ± SEM) of dorsal skin. Scale bar: 100 μm.

Data information: Statistical data are represented mean ± SEM. *P* values are determined by two-tailed unpaired Student's *t* test. $^{**}P<0.01$.

18) Figure 5G. and Figure 6C both look at psoriasis-related genes decreased in the IMQ model of Shp2-/- mice compared to wild-type mice. Maybe this can be combined to reduce redundancy.

Reply: Thank you for your suggestion. In Fig 5G, psoriasis-related genes decreased in dorsal skin of the IMQ model of *Shp2*$^{-/-}$ mice compared to wild-type mice. While in Fig 6C, psoriasis-related genes decreased in peritoneal macrophages derived from *Shp2*$^{-/-}$ mice compared to wild-type mice after IMQ stimulation. There are some differences between the two, and we have not

combined them in the revised manuscript. We have re-arranged in Figure 5 and 5G is now 5H.

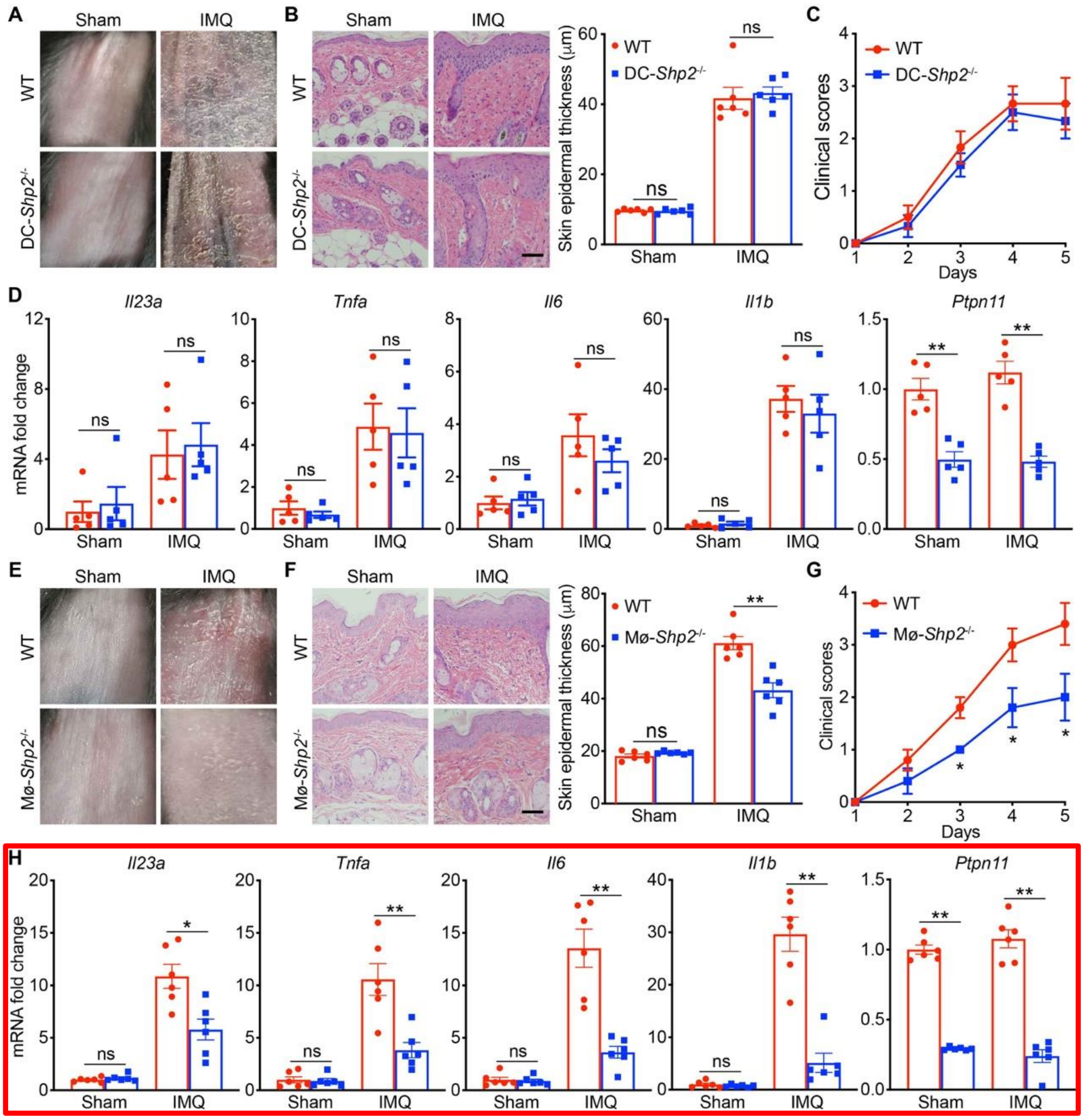


Figure 5. SHP2 deficiency in macrophages, but not in dendritic cell, alleviated the psoriasis-like phenotype in the IMQ-induced murine model.

Representative images (A and E), histological sections of dorsal back (B and F) and clinical scores (C and G) and quantitative PCR analysis of mRNA levels in dorsal skin (D and H) from wild-type (*n*=6) and DC-*Shp2*$^{-/-}$ mice or Mø-*Shp2*$^{-/-}$ mice (*n*=6) treated with IMQ for 4 days. Scale bar: 100 μm. Left: H&E (hematoxylin and eosin) staining data; right: statistical data (mean ± SEM). Results were normalized to *Gapdh* expression.

Data information: Data are represented mean ± SEM. *P* values were calculated by Tukey multiple comparison test (B, D, F, H) or Bonferroni multiple comparison test (C, G). **P*<0.05, ***P*<0.01, ns, not significant.

We have updated these data in **Figure 6C** in our revised manuscript.

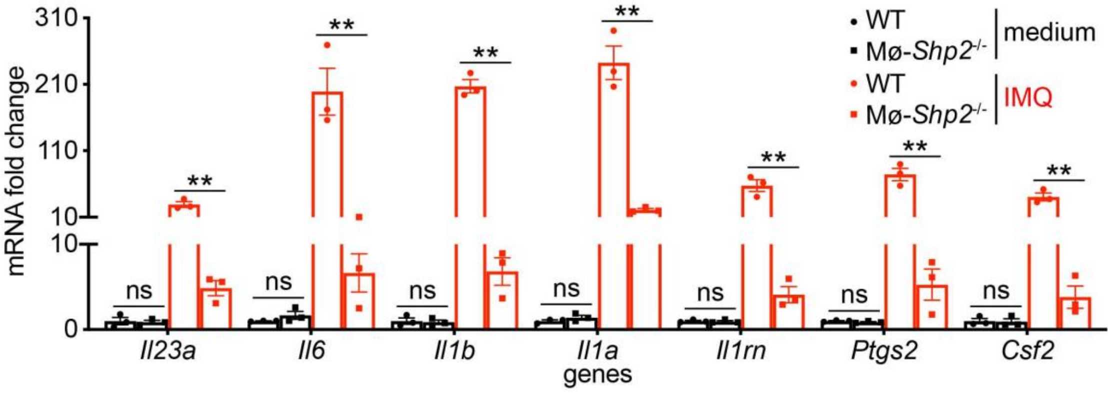


Figure 6C. Expression levels of representative psoriasis-related genes decreased in the IMQ model of Mø-*Shp2*$^{-/-}$ mice compared to wild-type mice.

Data information: Data are represented mean ± SEM. *P* values are determined by Tukey multiple comparison test (C). ***P*<0.01, ns, not significant.

## References


Brunner PM, Koszik F, Reininger B, Kalb ML, Bauer W & Stingl G (2013) Infliximab induces downregulation of the IL-12/IL-23 axis in 6-sulfo-LacNac (slan)+ dendritic cells and macrophages. *J Allergy Clin Immunol* 132: 1184-1193.e8

Caton ML, Smith-Raska MR & Reizis B (2007) Notch-RBP-J signaling controls the homeostasis of CD8- dendritic cells in the spleen. *J Exp Med* 204: 1653–1664

Chen Y-NP, LaMarche MJ, Chan HM, Fekkes P, Garcia-Fortanet J, Acker MG, Antonakos B, Chen CH-T, Chen Z, Cooke VG, et al (2016) Allosteric inhibition of SHP2 phosphatase inhibits cancers driven by receptor tyrosine kinases. Nature 535: 148–152

Chiang C-C, Cheng W-J, Korinek M, Lin C-Y & Hwang T-L (2019) Neutrophils in Psoriasis. Front Immunol 10: 2376

Deng Z, Ma S, Zhou H, Zang A, Fang Y, Li T, Shi H, Liu M, Du M, Taylor PR, et al (2015) Tyrosine phosphatase SHP-2 mediates C-type lectin receptor-induced activation of the kinase Syk and anti-fungal TH17 responses. Nat Immunol 16: 642–652

Durai V & Murphy KM (2016) Functions of Murine Dendritic Cells. *Immunity* 45: 719–736

Ganguly D, Chamilos G, Lande R, Gregorio J, Meller S, Facchinetti V, Homey B, Barrat FJ, Zal T & Gilliet M (2009) Self-RNA–antimicrobial peptide complexes activate human dendritic cells through TLR7 and TLR8. Journal of Experimental Medicine 206: 1983–1994

Guo W, Liu W, Chen Z, Gu Y, Peng S, Shen L, Shen Y, Wang X, Feng G-S, Sun Y, et al (2017) Tyrosine phosphatase SHP2 negatively regulates NLRP3 inflammasome activation via ANT1-dependent mitochondrial homeostasis. Nat Commun 8: 2168

Jiang M, Fang H, Shao S, Dang E, Zhang J, Qiao P, Yang A & Wang G (2019) Keratinocyte exosomes activate neutrophils and enhance skin inflammation in psoriasis. FASEB J 33: 13241–13253

Kadekar D, Agerholm R, Viñals MT, Rizk J & Bekiaris V (2020) The immune checkpoint receptor associated phosphatases SHP-1 and SHP-2 are not required for γδT17 cell development, activation, or skin inflammation. Eur J Immunol 50: 873–879

Katayama H (2018) Development of psoriasis by continuous neutrophil infiltration into the epidermis. *Exp Dermatol* 27: 1084–1091

Lin AM, Rubin CJ, Khandpur R, Wang JY, Riblett M, Yalavarthi S, Villanueva EC, Shah P, Kaplan MJ & Bruce AT (2011) Mast cells and neutrophils release IL-17 through extracellular trap formation in psoriasis. J Immunol 187: 490–500

Riol-Blanco L, Ordovas-Montanes J, Perro M, Naval E, Thiriot A, Alvarez D, Paust S, Wood JN & von Andrian UH (2014) Nociceptive sensory neurons drive interleukin-23-mediated psoriasiform skin inflammation. Nature 510: 157–161

Wang A & Bai Y (2020) Dendritic cells: The driver of psoriasis. J Dermatol 47: 104–113

Wang J, Mizui M, Zeng L-F, Bronson R, Finnell M, Terhorst C, Kyttaris VC, Tsokos GC, Zhang Z-Y & Kontaridis MI (2016) Inhibition of SHP2 ameliorates the pathogenesis of systemic lupus erythematosus. J Clin Invest 126: 2077–2092

Wang W-M & Jin H-Z (2020) Role of Neutrophils in Psoriasis. J Immunol Res 2020: 3709749

Wójcik P, Garley M, Wroński A, Jabłońska E & Skrzydlewska E (2020) Cannabidiol Modifies the Formation of NETs in Neutrophils of Psoriatic Patients. Int J Mol Sci 21: E6795

Xiao P, Zhang H, Zhang Y, Zheng M, Liu R, Zhao Y, Zhang X, Cheng H, Cao Q & Ke Y (2019) Phosphatase Shp2 exacerbates intestinal inflammation by disrupting macrophage responsiveness to interleukin-10. *J Exp Med* 216: 337–349

Referee #2 (Comments on Novelty/Model System for Author):

The so-called imiquimod model does not reflect all the features of the human disease (see Comments to Authors).

Referee #2 (Remarks for Author):

The manuscript by Zhu et al. provides some evidence for a role of SHP2 as a regulator of psoriasis-like skin lesions of imiquimod-treated mice and, much less so, of the human disease. In this context, one should remember that the so-called imiquimod model does not reflect many of the salient features of psoriasis such as the lack of co-morbidities and of chronicity.

Reply: We fully agree with you. Although IMQ-induced psoriasis-like murine model is the most widely used and recognized mouse model in mice (Zhu *et al*, 2017, PMID: 28377495; Glitzner *et al*, 2014, PMID: 25216727; Novoszel *et al*, 2021, PMID: 33724710), but it does not fully recapitulate human psoriasis. In our study, we also combined clinical data to try to compensate for the shortcomings in animal experiments. In addition, we provided an IL-23-induced psoriasis-like mouse model (Figure EV1 and Appendix Fig 20). These two models led to the similarly conclusion.

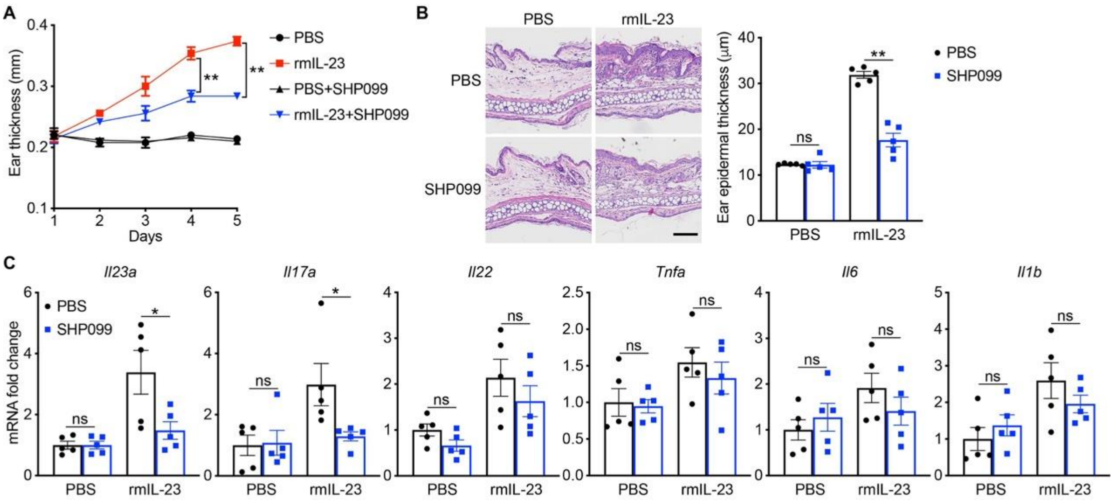


Figure EV1. SHP099 attenuated the psoriasis-like phenotype in the IL-23-induced murine model.

C57BL/6 mice (*n*=5/group) were subjected to IL-23-induced psoriasis-like skin inflammation and were treated with 10 mg/kg SHP099 or PBS for 4 days.

A, B Ear thickness (A), H&E staining and statistic results (B) of ear skin from SHP099 treated with or without C57BL/6 mice injected intradermally with rmIL-23 or PBS for 4 days. Ear thickness was measured daily.

C Quantitative PCR analysis of mRNA encoding IL-23/IL-17A axis cytokines and other psoriasis-related cytokines in the ear skin. Results were normalized to *Gapdh* expression.

Data information: Data are represented mean ± SEM. *P* values are determined by Tukey multiple comparison test (A–C). *$P<0.05$, **$P<0.01$, ns, not significant.

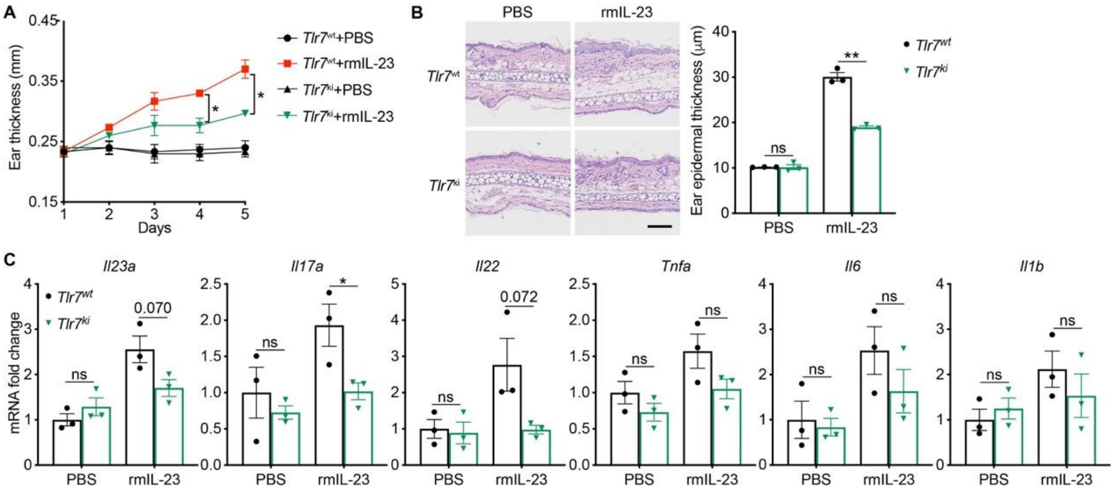


Appendix Fig S20. IL-23-induced psoriasis-like phenotype was alleviated in *Tlr7*$^{ki}$ mice. *Tlr7*$^{wt}$ male mice (*n*=3) and *Tlr7*-Y1025D mutant *Tlr7*$^{ki}$ male mice (*n*=3) were treated with indicated dose of rmIL-23 or PBS for 4 days.

A, B Ear thickness (A), H&E staining and statistic results (B) of ear skin from *Tlr7*$^{wt}$ and *Tlr7*$^{ki}$ mice injected intradermally with rmIL-23 or PBS for 4 days. Ear thickness was measured daily.

C Quantitative PCR analysis of mRNA encoding IL-23/IL-17A axis cytokines and other psoriasis-related cytokines in the ear skin. Results were normalized to *Gapdh* expression.

Data information: Data are represented mean ± SEM. *P* values are determined by Tukey multiple comparison test (A–C). **P*<0.05, ***P*<0.01, ns, not significant.

This reviewer has a few questions, comments and criticisms:

1. Fig. 1D illustrates the differences in SHP2 expression between NHS and psoriatic skin. Were all of the positively stained cells macrophages?

Reply: Thank you for your comment. In Fig. 1D, we examined the expression of SHP2 in the overall skin tissues. Here we conclude that SHP2 is highly expressed in the skin tissues of psoriatic patients. The positively stained cells including keratinocytes, fibroblasts, endothelial cells and immune cells.

Violin plot (A) and feature plot (B) show the expression and distribution of *Ptpn11* in subpopulations of mouse skin cells. *Ptpn11* is expressed in a variety of cells.

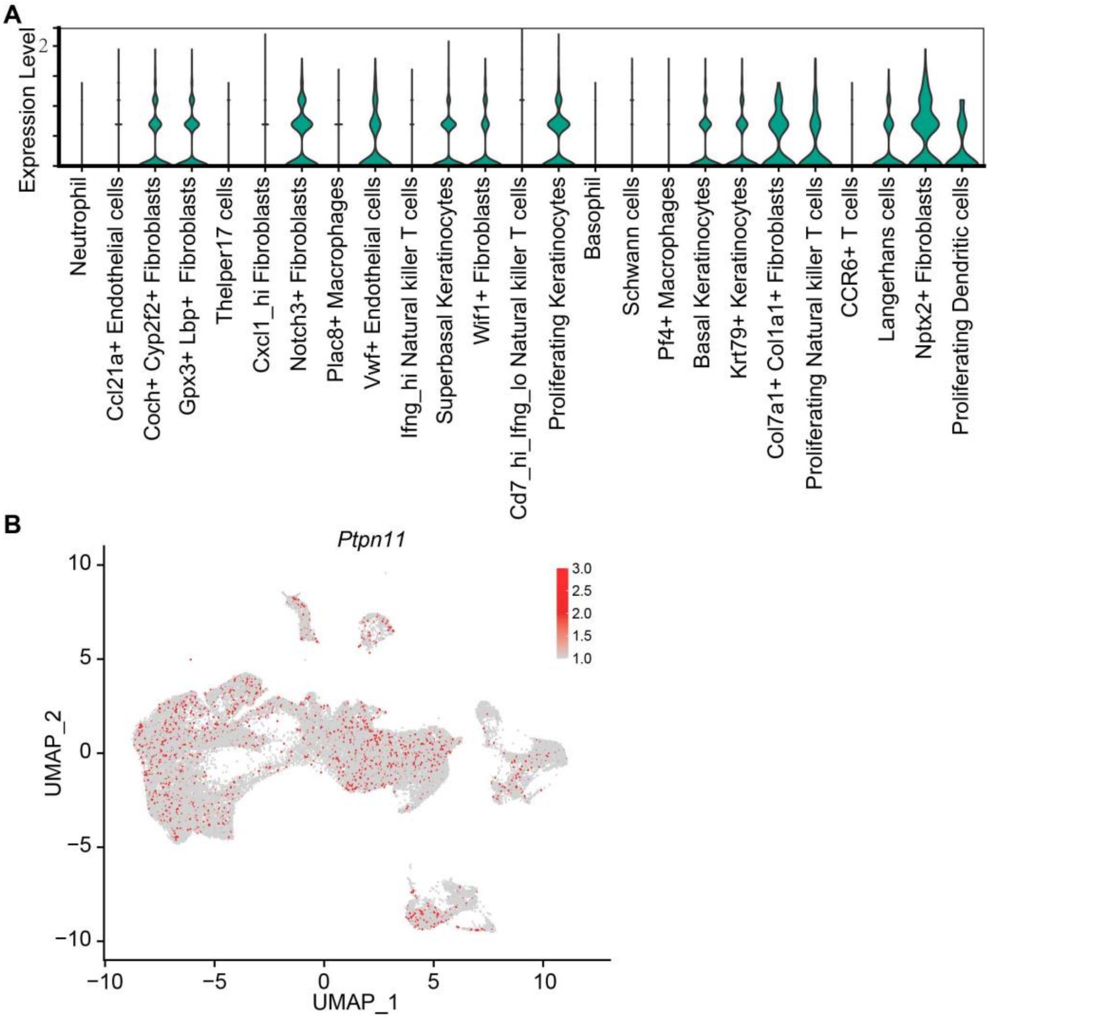


Response Figure 1. *Ptpn11* expression in all clusters.

(A) Violin plot showed the distribution of the normalized expression level of *Ptpn11*.

(B) Feature plots of expression distribution for *Ptpn11*. Expression levels are color-coded and overlaid onto UMAP plot.

Immunofluorescence staining showed that SHP2 was highly expressed in macrophages of skin lesions in psoriasis patients compared to healthy donors.

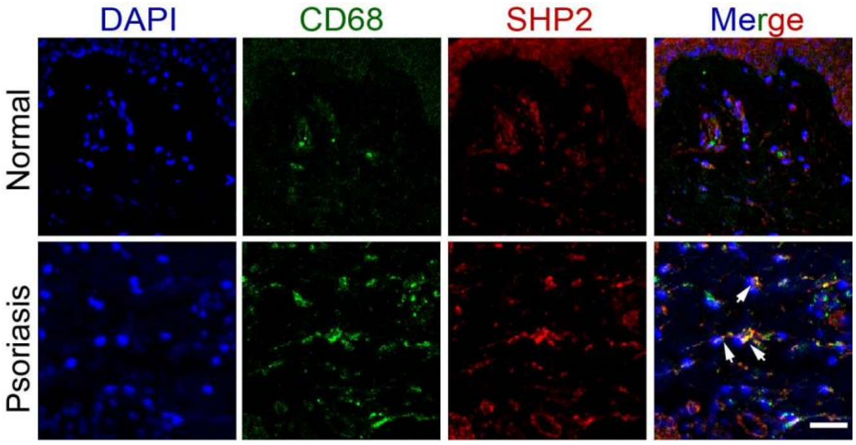

Appendix Fig S12B. Immunofluorescence staining of skin sections from psoriatic patients and normal controls with SHP2 and CD68, showing SHP2 expression in macrophages (arrows). CD68, green; SHP2, red. Scale bar: 100 μm.

What about TIPP-DCs, i.e. TNF-alpha and iNOS-expressing cells which supposedly play a critical role in the pathogenic IL-23/IL-17 axis?

Reply: We appreciate this constructive advice raised by the reviewer. TNF-alpha and iNOS-expressing cells have been shown to play an important role in inflammatory diseases (Wilsmann-Theis *et al*, 2013, PMID: 23742057; Bosschaerts *et al*, 2010, PMID: 20714353; Chong *et al*, 2011, PMID: 21469104).

We analyzed the *Tnfa* (encoding TNF-alpha) and *Nos2* (encoding iNOS) expression in dorsal skin of mice by scRNA-seq. TIPP-DCs were found to be barely expressed in mice skin, so their role in the IL-23/IL-17 axis could not be explored in depth.

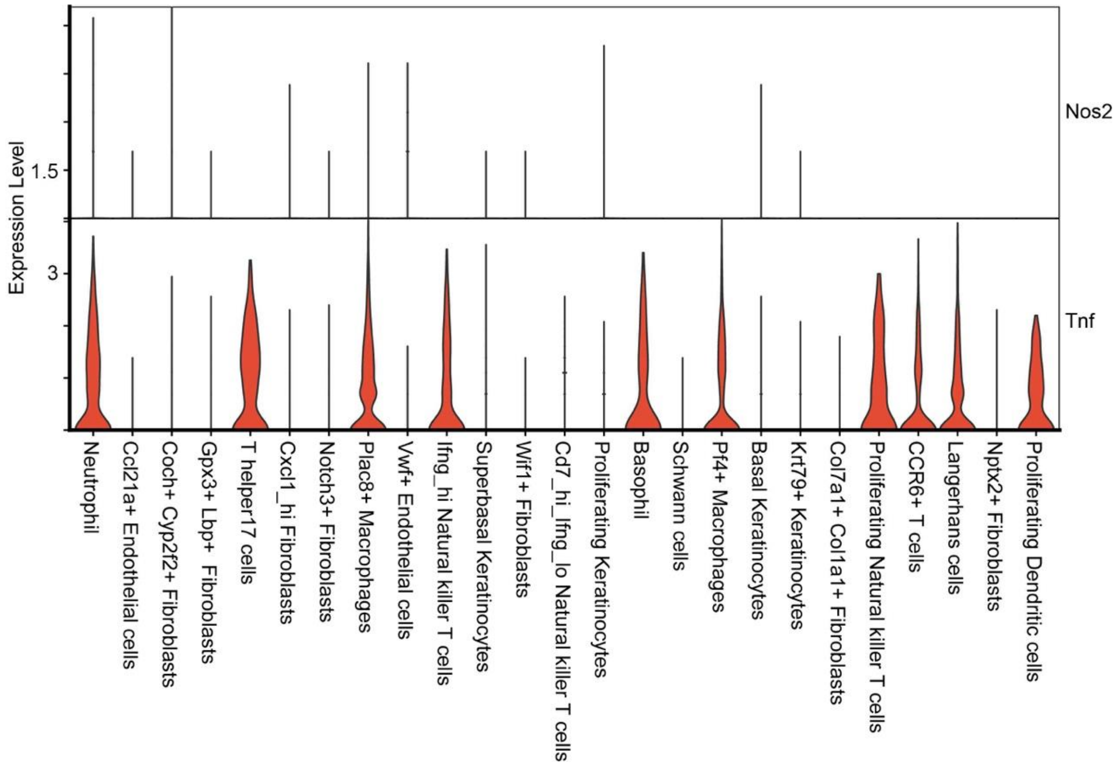


Response Figure 18. Expression of TNF-alpha and iNOS-expressing cells in mouse skin. Violin plot showed the distribution of *Tnf* and *Nos2*.

2. Have the authors verified that the DC- and macrophage knockout mice were indeed devoid of the respective cell populations?

Reply: Thank you for your suggestion. We have performed western blot to detect SHP2 expression in BMDMs and BMDCs cells derived from macrophage- and DC knockout mice. Conditional knockout mice have good specificity, SHP2 was not deleted in DCs isolated from Mø-*Shp2*$^{-/-}$ mice.

Similarly, SHP2 was not deleted in macrophages isolated from DC-*Shp2*$^{-/-}$ mice.
We have updated these data in **Appendix Fig S10A** and **S10C** in our revised manuscript.

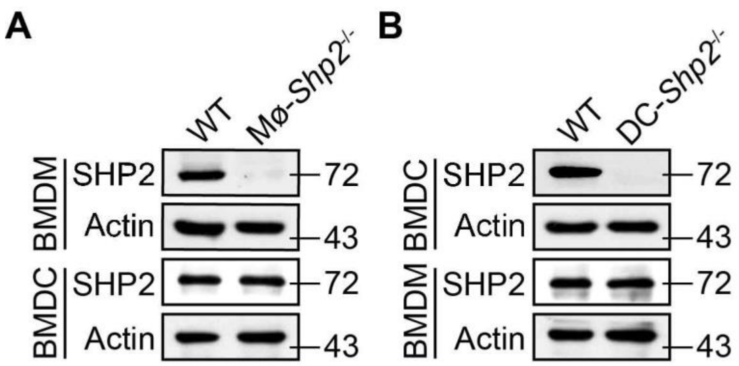


Response Figure 19. Deletion efficiency of SHP2 in macrophage- and DC knockout mice. (A and B) Cell lysates from BMDMs and BMDCs generated from wild-type mice and Mø-*Shp2*$^{-/-}$ mice (A) or DC-*Shp2*$^{-/-}$ mice (B) were probed by anti-SHP2 and anti-Actin.

3. Ample evidence exists that application of imiquimod to both human and murine skin results in the accumulation of substantial numbers of plasmacytoid dendritic cells (pDC. These cells, by the way, are to great extent responsible for the efficacy of imiquimod in the treatment of genital warts, actinic keratoses and basal cell carcinomas.
In the scRNA-seq analysis (Fig. 3A-C) I could not found pDC in any of the 6 clusters/25 subclusters. How can this be explained?
Reply: It is a good question. scRNA-seq clusters were annotated according to marker genes and pDC were included in the cluster M0-M2 because of their low marker gene expression.
Feature plot (A) and violin plot (B) show that *Siglech*, pDC marker, is mainly expressed in Langerhans cells (M2), *Pf4*$^{+}$ Macrophages (M1) and *Plac8*$^{+}$ Macrophages (M0).

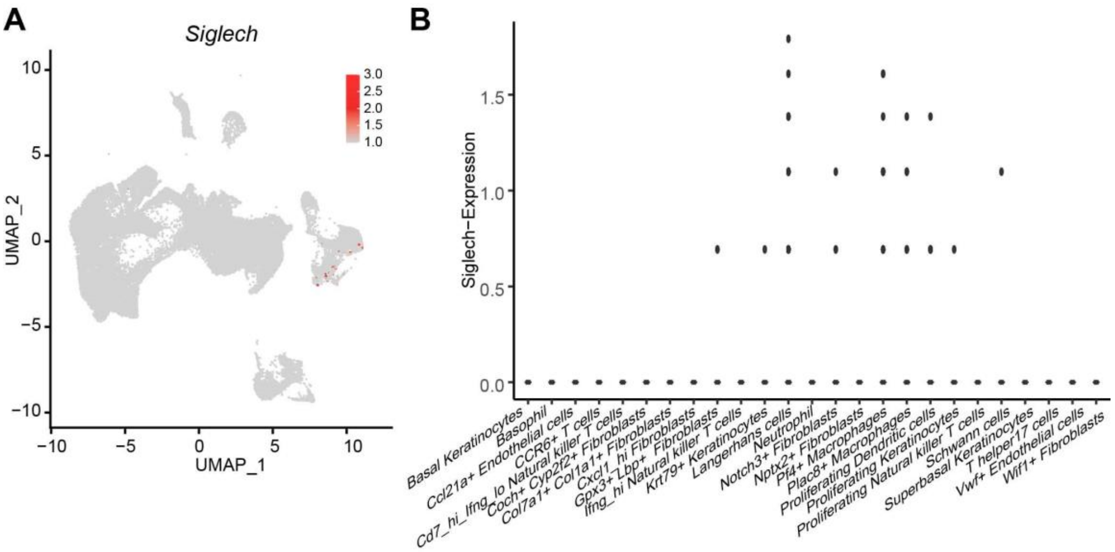

Response Figure 20. *Siglech* expression in all clusters of mouse skin cells.

(A) Violin plot showed the distribution of the normalized expression level of *Siglech*.

(B) Feature plots of expression distribution for *Siglech*. Expression levels are color-coded and overlaid onto UMAP plot.

4. Of minor importance: the manuscript contains many typos and linguistic flaws. They should be corrected.

Reply: Thank you for your suggestion. We have got our paper professionally edited for English language by the highly qualified native English-speaking editors at Scribendi Editing Services. English language, grammar, punctuation, spelling, and overall style have been greatly polished. For your convenience, the language editing certificate is attached below.

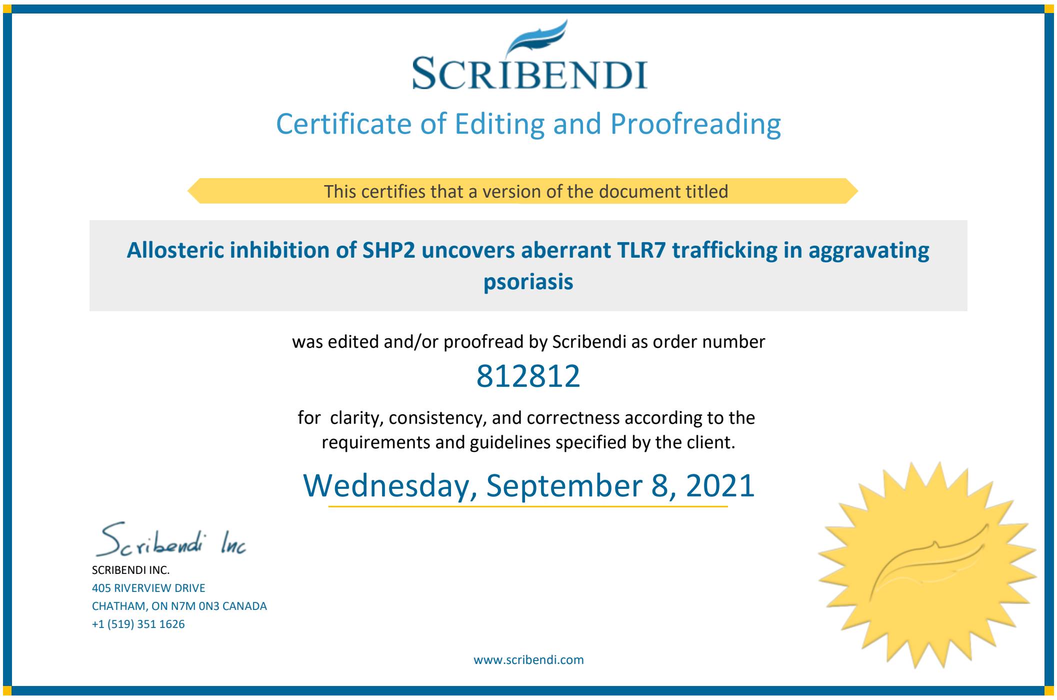

SCRIBENDI

Certificate of Editing and Proofreading

This certifies that a version of the document titled

**Allosteric inhibition of SHP2 uncovers aberrant TLR7 trafficking in aggravating psoriasis**

was edited and/or proofread by Scribendi as order number

812812

for clarity, consistency, and correctness according to the requirements and guidelines specified by the client.

Wednesday, September 8, 2021

Scribendi Inc

SCRIBENDI INC.
405 RIVERVIEW DRIVE
CHATHAM, ON N7M 0N3 CANADA
+1 (519) 351 1626

www.scribendi.com

Printed on 2021-09-08 at 1:34 PM

## References


Bosschaerts T, Guilliams M, Stijlemans B, Morias Y, Engel D, Tacke F, Hérin M, De Baetselier P & Beschin A (2010) Tip-DC development during parasitic infection is regulated by IL-10 and requires CCL2/CCR2, IFN-gamma and MyD88 signaling. PLoS Pathog 6: e1001045

Chong SZ, Wong KL, Lin G, Yang CM, Wong S-C, Angeli V, Macary PA & Kemeny DM (2011) Human CD8+ T cells drive Th1 responses through the differentiation of TNF/iNOS-producing dendritic cells. Eur J Immunol 41: 1639–1651

Glitzner E, Korosec A, Brunner PM, Drobits B, Amberg N, Schonthaler HB, Kopp T, Wagner EF, Stingl G, Holcmann M, et al (2014) Specific roles for dendritic cell subsets

during initiation and progression of psoriasis. EMBO Mol Med 6: 1312–1327

Novoszel P, Holcmann M, Stulnig G, De Sa Fernandes C, Zyulina V, Borek I, Linder M, Bogusch A, Drobits B, Bauer T, et al (2021) Psoriatic skin inflammation is promoted by c-Jun/AP-1-dependent CCL2 and IL-23 expression in dendritic cells. EMBO Mol Med 13: e12409

Wilsmann-Theis D, Koch S, Mindnich C, Bonness S, Schnautz S, von Bubnoff D & Bieber T (2013) Generation and functional analysis of human TNF-α/iNOS-producing dendritic cells (Tip-DC). Allergy 68: 890–898

Zhu H, Lou F, Yin Q, Gao Y, Sun Y, Bai J, Xu Z, Liu Z, Cai W, Ke F, et al (2017) RIG-I antiviral signaling drives interleukin-23 production and psoriasis-like skin disease. EMBO Mol Med 9: 589–604

Referee #3 (Comments on Novelty/Model System for Author):
LysMcre mice may be useful for Cre-lox studies, but authors need to be careful with their language; LysMcre-lox will delete in all myeloid cell lineages, which include monocytes, mature macrophages and granulocytes. Therefore, the authors cannot specifically assume that the effects of SHP2 in psoriasis stem strictly from macrophage expression.

Referee #3 (Remarks for Author):
The manuscript entitled, "Allosteric inhibition of SHP2 uncovers aberrant TLR7 trafficking in aggravating psoriasis" by Zhu et. al. sought to investigate whether TLR7 regulation of NFkB signaling in macrophage is regulated by SHP2 in psoriasis. This is an elegant paper that reveals that SHP2 may be an accelerator of psoriasis and that its inhibition may be a promising therapeutic for patients. There are a few concerns, however, that if rectified would strengthen the manuscript. Specifically, the conclusions are overstated somewhat based on the experimentation presented. Therefore, there is a need for some additional mechanistic data to prove these potential findings. In particular, no data is presenting showing the binding site of SHP2 to TLR7. Moreover, the role of TLR7 (as opposed to other downstream effectors) specifically in the regulation of psoriasis-associated inflammation in response to SHP2 activity is not clearly justified, as no other downstream effectors were evaluated directly. Finally, quantification of all western data is needed, as well as more information on statistical assessments and n values used for experiments. Details on these and other concerns are outlined below:
Reply: Thank you for your positive evaluation and encouragement.

Major concerns:
1) Control experiments are largely missing throughout. For example, in Fig 1I, normal PBMCs should similarly be treated with SHP099 as the patient PBMCs, to assess cytokine effects of the inhibitor in control cells.
Reply: As suggested by the reviewer, we have assessed cytokine effects of the inhibitor in normal PBMCs. SHP099 did not affect the expression of inflammatory factors in normal PBMCs.
We have updated these data in **Appendix Fig S2** in our revised manuscript.

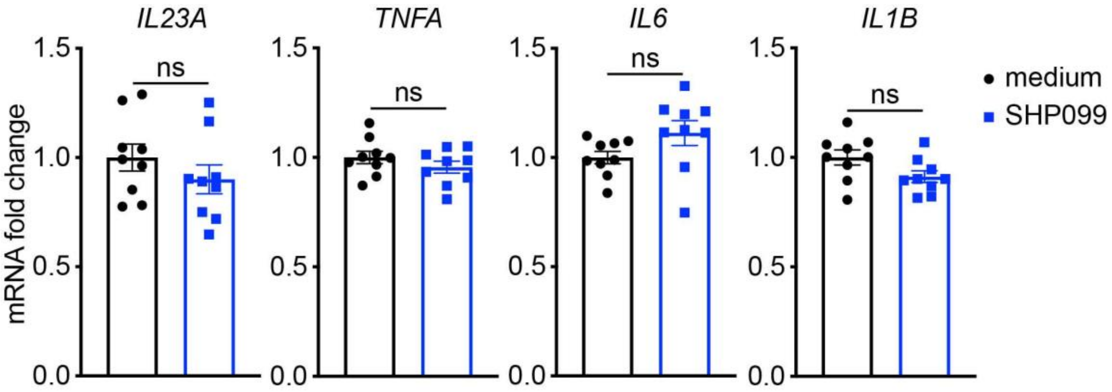

Response Figure 21. SHP099 did not affect the expression of inflammatory factors in normal PBMCs.

Quantitative PCR analysis of mRNA levels in human PBMCs derived from normal healthy controls (*n*=9) were treated with or without SHP099 (10 μM).

Data information: Data are represented mean ± SEM. *P* values are determined by Two-tailed Student's *t* test. ns, no significant.

Similarly, in Fig 2A and Fig 3A, sham mice should also be treated with the inhibitor to compare the effects at baseline in normal mice.

Reply: We have followed the reviewer's suggestion and treated sham mice with SHP099. As a result, SHP099 did not affect the skin condition of normal mice.

We have updated these data in **Appendix Fig S3** in our revised manuscript.

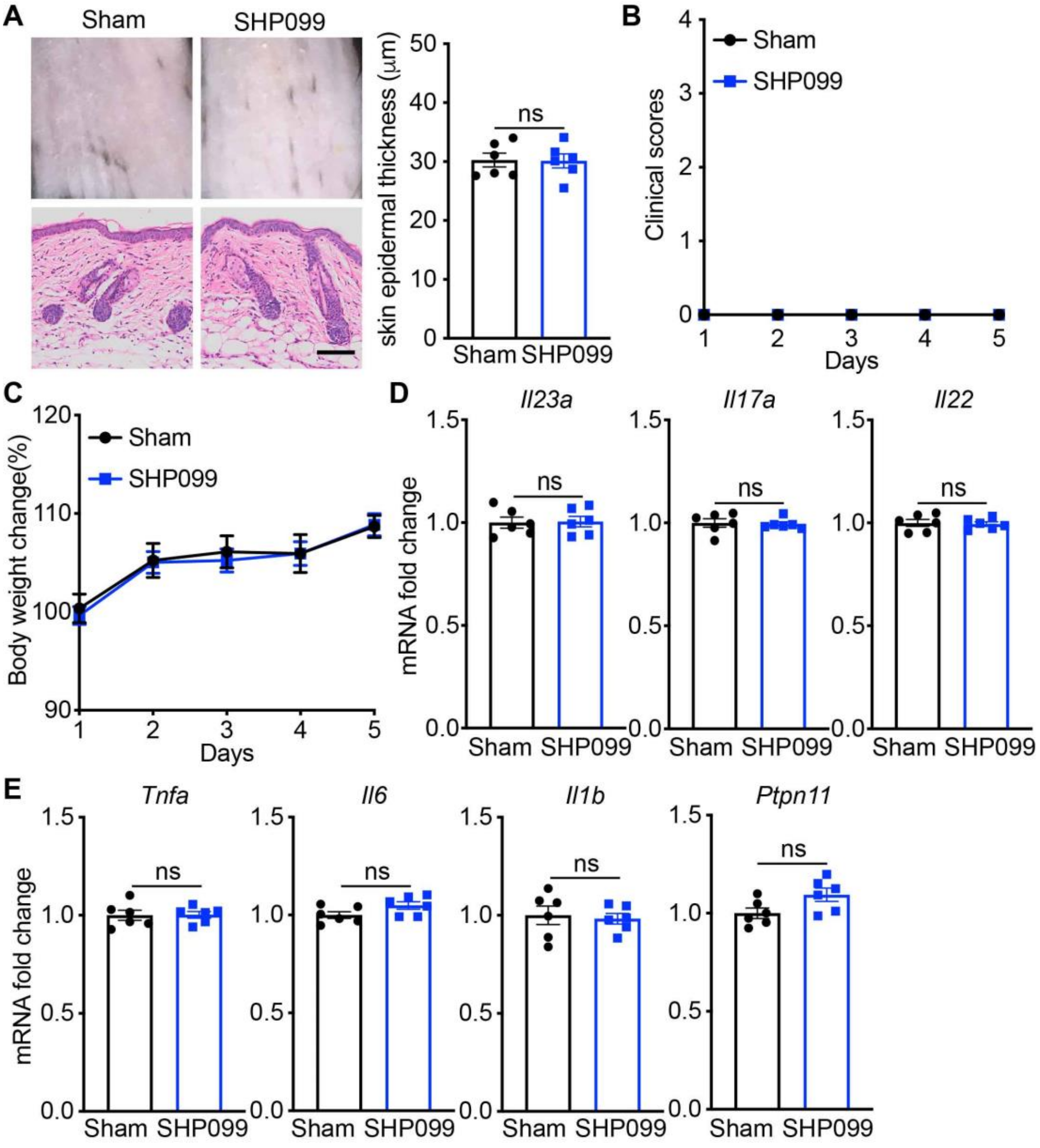


Response Figure 22. SHP099 did not affect the skin condition of normal mice.

C57BL/6 mice (*n*=6/group) were treated with 10 mg/kg SHP099 or vehicle for 4 days. (A) Phenotypic presentation (*top*) and H&E staining (*bottom*) of dorsal skin. Scale bar: 100 µm. Left: H&E staining data; right: statistical data (mean ± SEM).

(B) Clinical scores plotted with mean ± SEM.

(C) Body weight change plotted with mean ± SEM.

(D and E) Quantitative PCR analysis of mRNA encoding IL-23/IL-17A axis cytokines (D) and other psoriasis-related cytokines (E) in the dorsal skin. Results were normalized to *Gapdh* expression.

Data information: Data are represented mean ± SEM. *P* values are determined by two-tailed Student's *t* test (A, D, E). ns, no significant.

Given that SHP099 does not affect the expression of inflammatory factors in normal PBMCs and the skin of sham mice, and that single-cell sequencing is expensive, single-cell sequencing of the sham+SHP099 group was not performed.

2) Fig 3: It is clear that the lymphocytes, and to a lesser extent myeloid cells, play a key role in upregulating inflammatory genes in psoriasis. However, which cell type is most affected by SHP099 treatment, ie which cell type becomes most normalized?

Reply: We thank this very helpful question raised by the reviewer. The violin plot shows the expression of inflammation-related genes in 3 groups (Sham, IMQ and IMQ+SHP099) of cells, and it is clear that SHP099 treatment normalized Myeloid cells and Endothelial cells in psoriasis-like mouse skin lesions.

We have updated these data in **Figure 3F** in our revised manuscript.

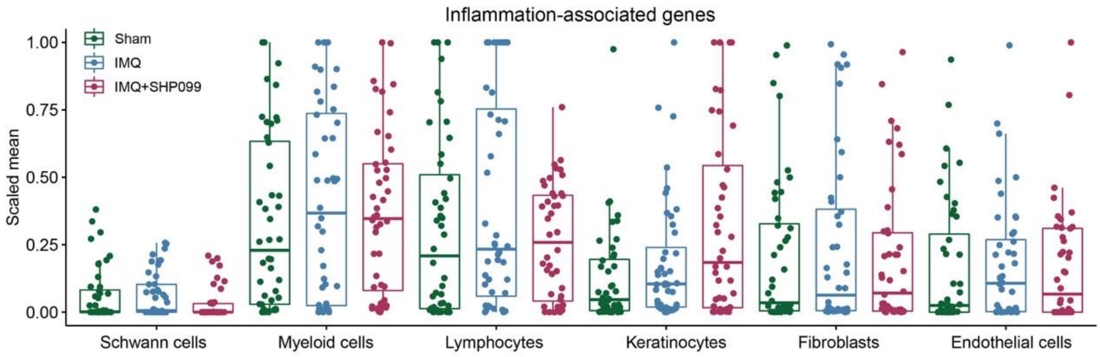


Response Figure 23. SHP099 treatment normalized Myeloid cells and Endothelial cells in psoriasis-like mouse skin lesions.

Boxplot showed the relative expression of 47 inflammation-associated genes based on sample cell clusters.

3) LysMcre mice may be useful for Cre-lox studies, but authors need to be careful with their language; LysMcre-lox will delete in all myeloid cell lineages, which include monocytes, mature macrophages and granulocytes. Therefore,

the authors cannot specifically assume that the effects of SHP2 in psoriasis stem strictly from macrophage expression.

Reply: We agree with the reviewer and have corrected our description. The development of psoriasis cannot occur without the involvement of multiple immune cells. scRNA-seq analysis revealed that top ten hub genes were mainly expressed in macrophages (Fig. 4 G), implying that macrophages may function as master regulators in a cell type-specific manner in psoriasis. In studying the molecular mechanisms by which SHP2 exacerbates the progression of psoriasis, we have conducted studies on macrophages and have also demonstrated that SHP2 exacerbates psoriasis by promoting TLR7 endosomal localization in macrophages.

Oxidative stress, granule composition and neutrophil extracellular traps of neutrophils are associated with the initial and maintenance phases of psoriasis (Chiang *et al*, 2019, PMID: 31649677; Jiang *et al*, 2019, PMID: 31539277; Lin *et al*, 2011, PMID: 21606249; Wang & Jin, 2020, PMID: 32587871; Katayama, 2018, PMID: 30019426; Wójcik *et al*, 2020, PMID: 32947961).

In fact, other colleagues in our laboratory are conducting a related study in which they found that neutrophil conditional knockout SHP2 mice have reduced psoriasis compared to wild-type mice. This part of the data has not been published and is only available to the editor and reviewers.

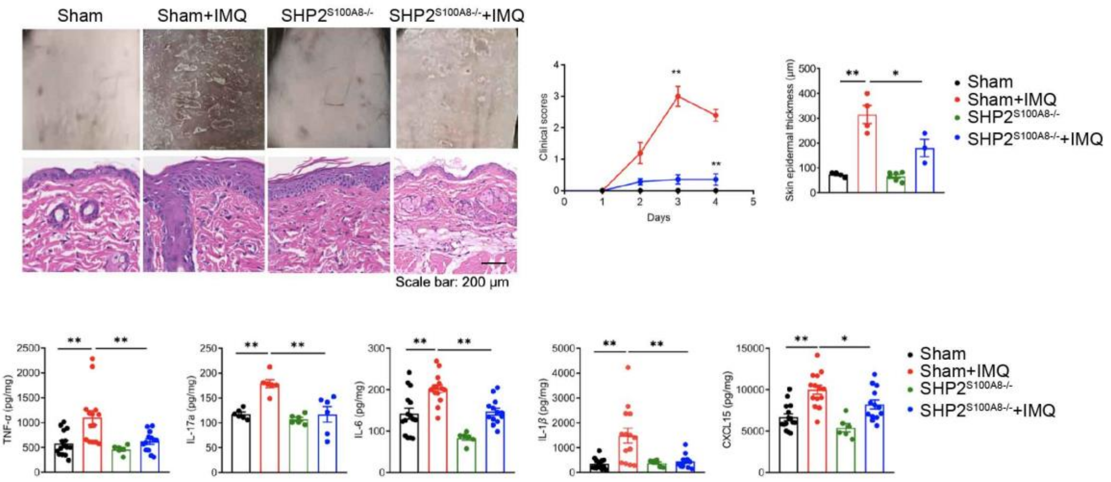


Response Figure 6. Neutrophil conditional knockout SHP2 mice with remission of psoriasis symptoms. (Unpublished observations).

4) Fig 5: It is clear that skin lesions are normalized in IMQ-treated SHP2-deleted monocytes; however, the data for IMQ-treated SHP2 deleted dendritic cells is missing and integral to show that the effects are predominantly affecting macrophage and not DCs.

Reply: Thank you for your suggestion. We have performed qPCR to analysis inflammation-related gene expression in the dorsal skin of DC-*Shp2*$^{-/-}$ mice and wild-type mice.

We have updated these data in **Figure 5D** in our revised manuscript.

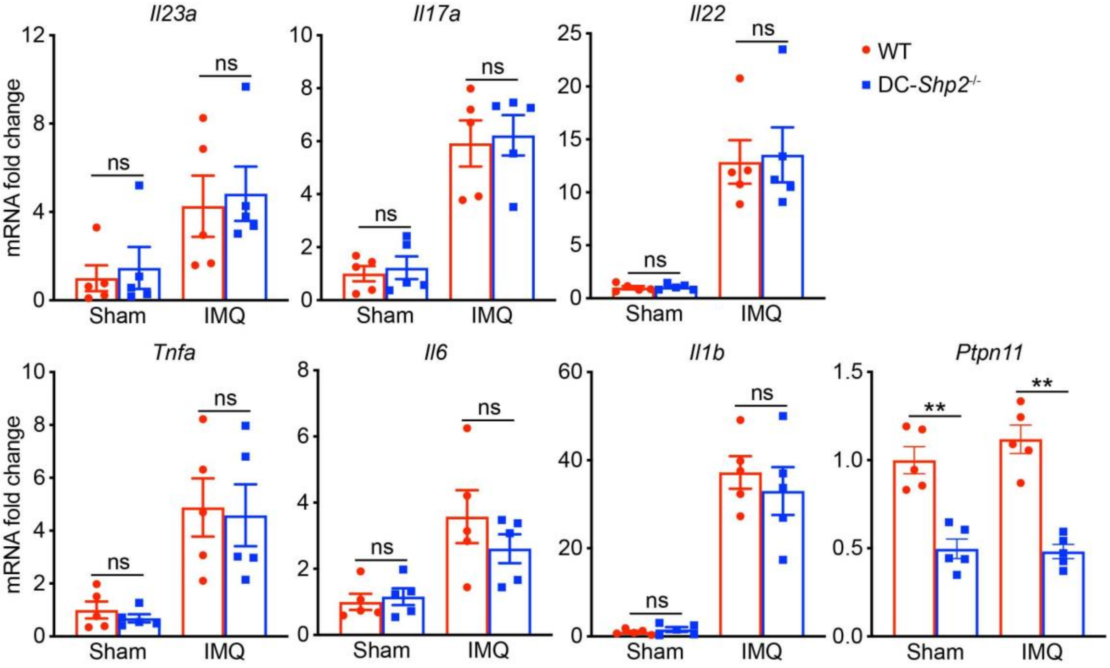


Response Figure 24. SHP2 deficiency in DCs did not affect the expression of inflammatory factors in IMQ-induced mouse skin.

Quantitative PCR analysis of mRNA levels in dorsal skin from wild-type and DC-*Shp2*$^{-/-}$ mice. Results were normalized to *Gapdh* expression.

Data information: Data are represented mean ± SEM. *P* values were calculated by Tukey multiple comparison test. ***P*<0.01, ns, not significant.

5) Fig 6: Baseline macrophage data are needed to compare and determine normalization of genes in macrophage in response to IMQ treatment in SHP2 deleted cells.

Reply: According to the reviewer's comment, we have repeated the experiment in Fig 6C and supplemented the baseline macrophage data.

We have updated these data in **Figure 6C** in our revised manuscript.

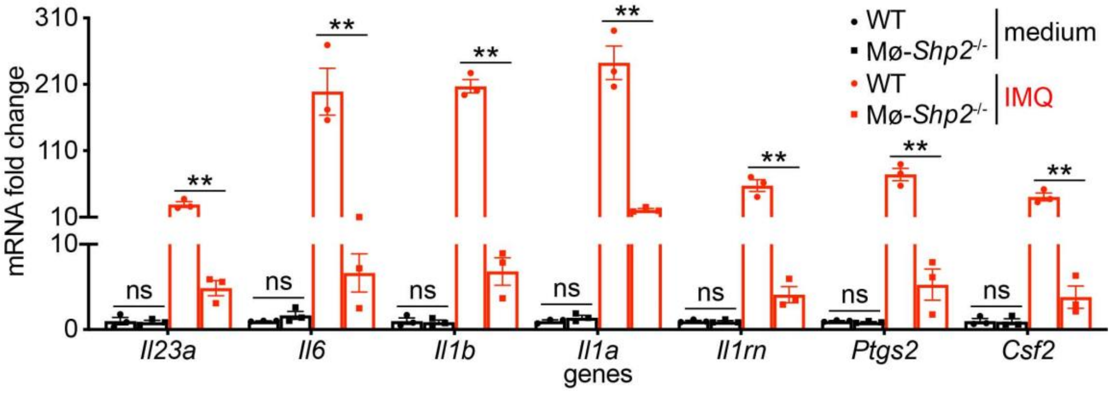


Figure 6C. Expression levels of representative psoriasis-related genes decreased in the IMQ model of Mø-*Shp2*$^{-/-}$ mice compared to wild-type mice.

Data information: Data are represented mean ± SEM. *P* values are determined by Tukey multiple comparison test. ***P*<0.01, ns, not significant.

6) Why did the authors overexpress SHP2 instead of measure its effects in SHP2 immunocomplexes endogenously? For example, in response to IMQ, an experiment to IP SHP2 blot pY and reprobe for TLR7 would show whether SHP2 binds to TLR7 and affects its phosphorylation directly. This should be demonstrated in macrophage and BMDCs.

Reply: Thank you for your suggestion. In the original manuscript, we gave IMQ stimulation to THP1 cells and then IP SHP2, blot TLR7, demonstrating endogenous binding of SHP2 to TLR7 (A). We have also performed an experiment according to this referee's suggestion. We gave IMQ stimulation to BMDMs and IP SHP2 blot p-Tyr and reprobe for TLR7, demonstrating that SHP2 binds to TLR7 and affects its phosphorylation (B).

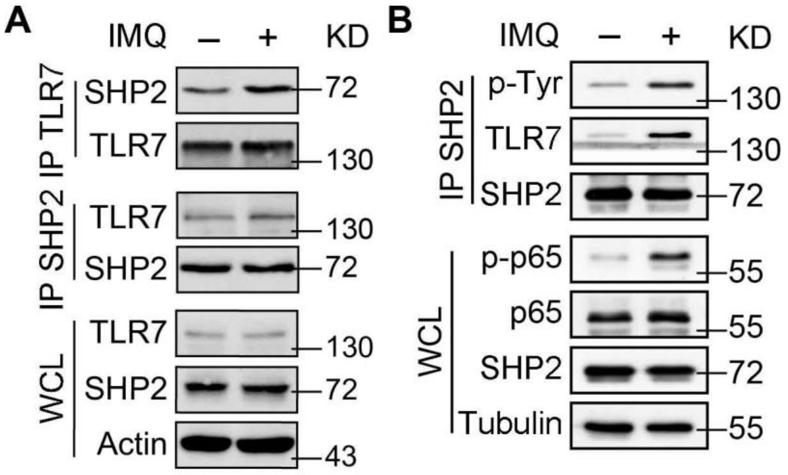


Response Figure 25. SHP2 binds to TLR7 and affects its phosphorylation.

(A) PMA-differentiated THP-1 cells were unstimulated or stimulated by IMQ (10 µg/ml) for 30 min. Cell lysates were immunoprecipitated by antiTLR7 or anti-SHP2 and probed by indicated antibodies.

(B) BMDMs derived from wild-type mice were stimulated by IMQ (10 µg/ml) for 30 min. Cell lysates were immunoprecipitated by anti-SHP2 and probed by indicated antibodies.

Since conditional knockdown of SHP2 in DCs did not affect the development of psoriasis in mice, we did not address DCs in the mechanism exploration section.

7) As well, the authors mention that they identified several SHP2-interacting proteins but only focused on TLR7. The authors need to identify these interacting proteins and then validate that it is indeed TLR7 that is integral to the SHP2-mediated effects in psoriasis.

Reply: This is a very valid concern. We demonstrated the binding of SHP2 to TLR7 by endogenous and exogenous IP experiments and constructed TLR7 point mutant mice to demonstrate the important role of TLR7 locus 1025 phosphorylation in murine psoriasis. Moreover, SHP099 provide no benefit in

the TLR7-Y1025D mice treated with IMQ. These data validate that TLR7 is integral to the SHP2-mediated effects in psoriasis.
We have updated these data in **Figure EV5** in our revised manuscript.

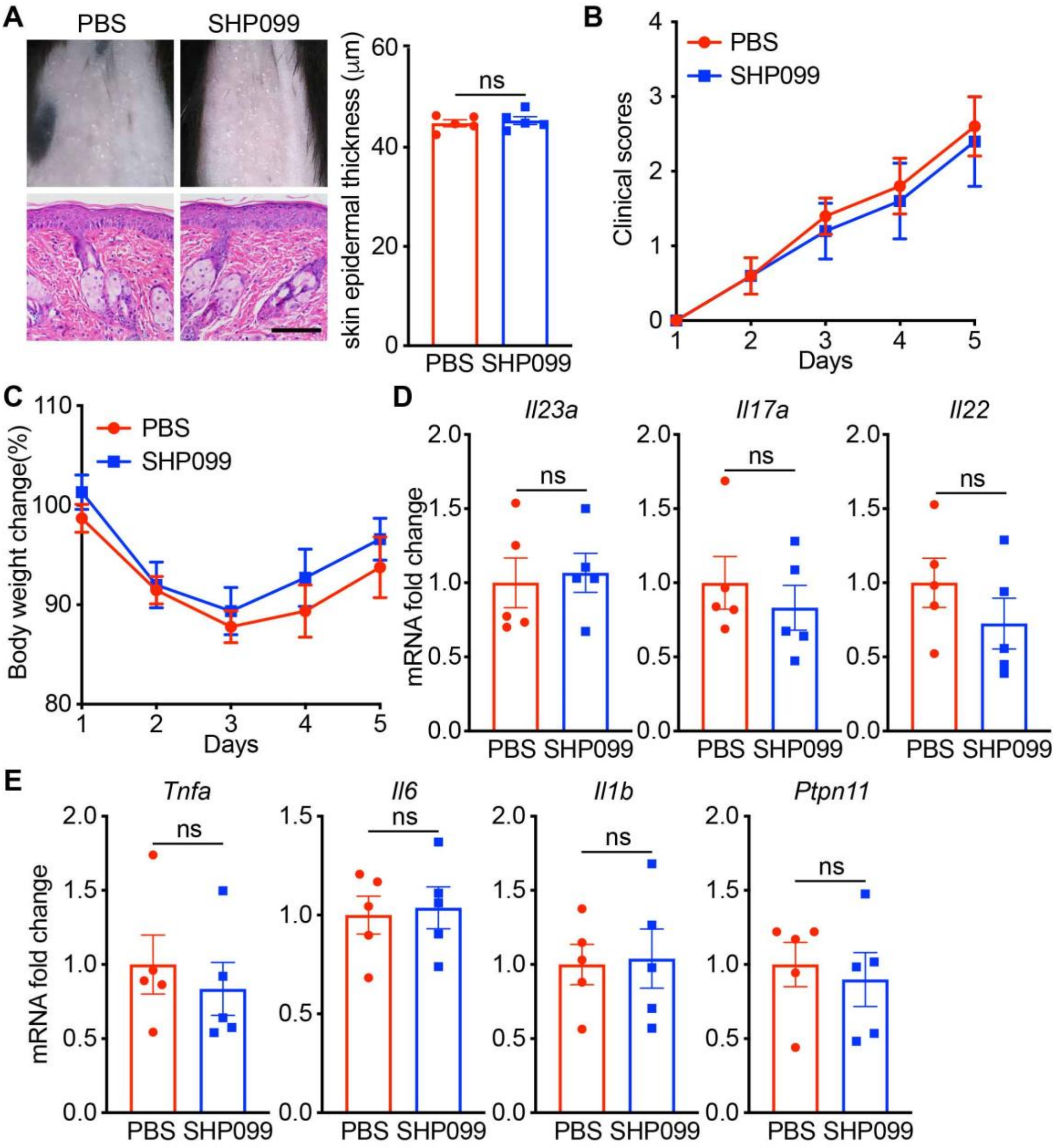


Response Figure 26. SHP099 provided no benefit in the *Tlr7*-Y1025D mice treated with IMQ.
*Tlr7*-Y1025D mutant *Tlr7*$^{ki}$ male mice (*n*=5/group) were treated with 10 mg/kg SHP099 or vehicle for 4 days.
(A) Phenotypic presentation (top) and H&E staining (bottom) of dorsal skin. Scale bar: 100 μm. Left: H&E staining data; right: statistical data (mean ± SEM).
(B) Clinical scores plotted with mean ± SEM.
(C) Body weight change plotted with mean ± SEM.

(D and E) Quantitative PCR analysis of mRNA encoding IL-23/IL-17A axis cytokines (D) and other psoriasis-related cytokines (E) in the dorsal skin. Results were normalized to *Gapdh* expression.

Data information: Data are represented mean ± SEM. *P* values are determined by two-tailed Student's *t* test (A, D, E). ns, no significant.

8) Authors also indicate that TLR7 binds SHP2 in the PTP domain; how is this possible, at what site/residue? Validation here is required as this is not the normal binding for SHP2 to proteins; almost all binding for SHP2 occurs through its SH2 domains to pY proteins (like TLR7) or through C-terminal tail binding at its own pY residues. The size of the PTP deletion mutant suggests that it is a truncated protein that may not express the c-terminus either?

Reply: As suggested by the reviewer, we have repeated the experiment. We constructed mutants of SHP2 with the deletion of various domains, SH2 and PTP domains of SHP2 were demonstrated to interact with TLR7.

We have updated these data in **Figure 7E** in our revised manuscript.

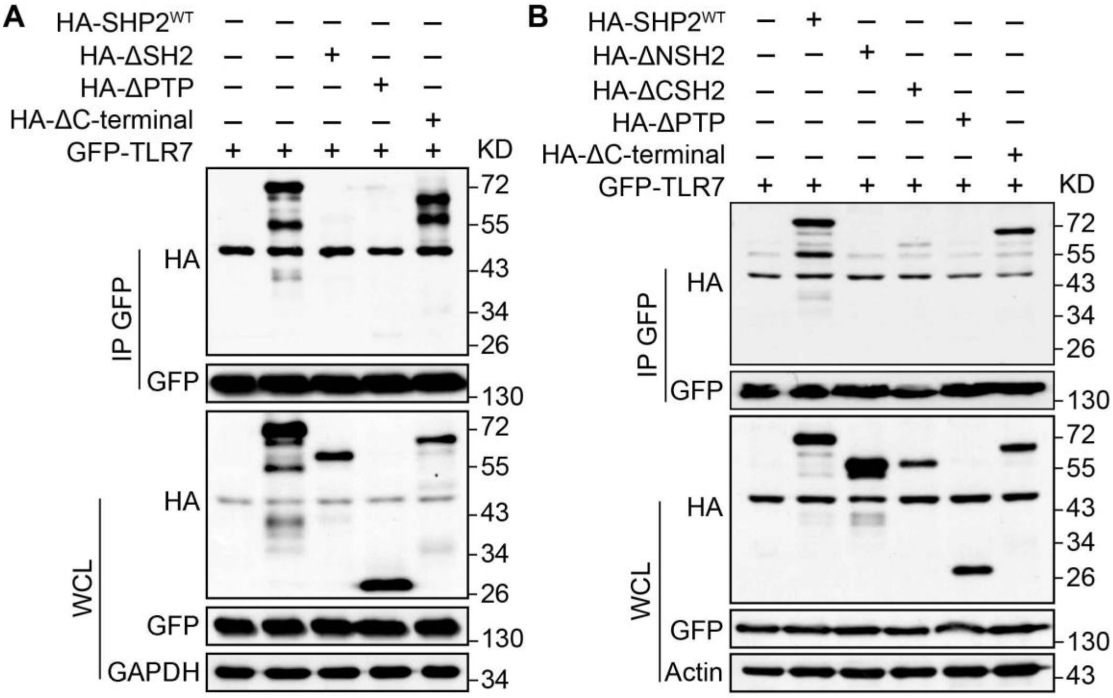


Response Figure 27. TLR7 binds SHP2 in the SH2 and PTP domain.

(A and B) Immunoblotting of HEK293T cells co-transfected for 48 h with GFP-TLR7, plus HA-SHP2, HA-SHP2 mutant vectors, followed by immunoprecipitation with anti-GFP beads.

9) Fig 8: The authors suggest SHP2 affects TLR phosphorylation, but this was never directly measures. Authors instead show that mutations at various tyr residues affects expression, but this is not the same as showing its phosphorylation.

Reply: We thank the reviewer for this insightful question. First, we demonstrated that SHP2 binds to TLR7 and affects its phosphorylation (A).

We then demonstrated that the enzymatic activity of SHP2 affects the phosphorylation of TLR7 (B). Finally, we constructed TLR7 point mutant mice to demonstrate the important role of TLR7 locus 1025 phosphorylation in murine psoriasis. In summary, we conclusively proved that SHP2 affects the phosphorylation of TLR7.

We have updated these data in **Figure 7C** and **8A** in our revised manuscript.

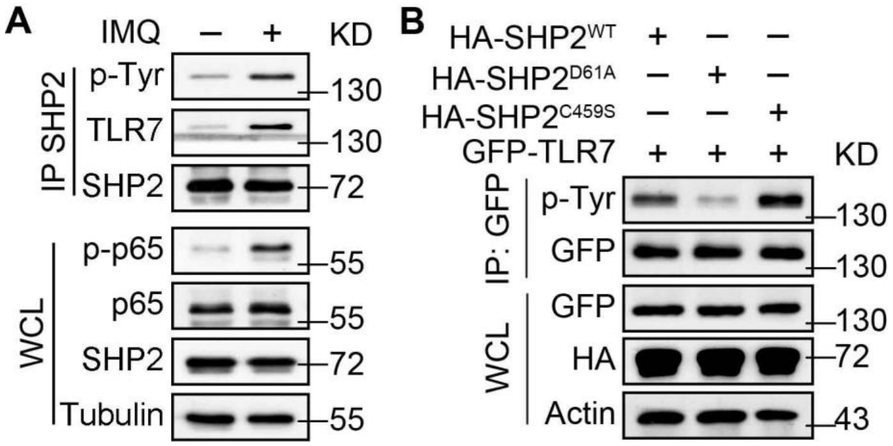


Response Figure 28. SHP2 affected TLR phosphorylation.

(A) BMDMs derived from wild-type mice were stimulated by IMQ (10 μg/ml) for 30 min. Cell lysates were immunoprecipitated by anti-SHP2 and probed by indicated antibodies.

(B) Immunoblotting of HEK293T cells co-transfected for 48 h with GFP-TLR7, plus HA-tagged SHP2 or SHP2 mutant vectors, followed by immunoprecipitation with anti-GFP.

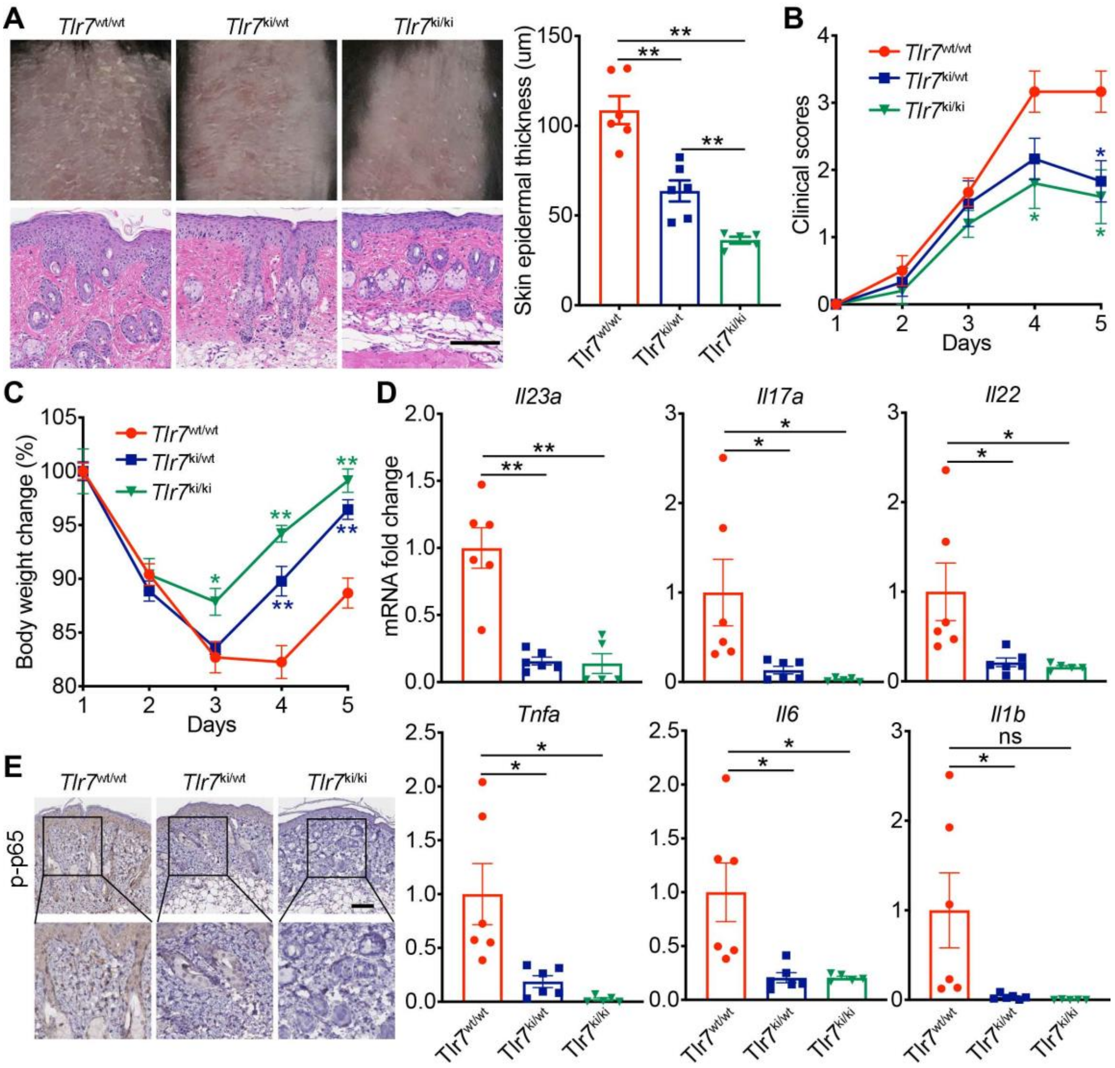


Figure 9. The *Tlr7*-Y1025D mutant alleviated the psoriasis-like phenotype in the IMQ-induced murine model.

*Tlr7*$^{wt/wt}$ female mice (*n*=6) and *Tlr7*-Y1025D mutant *Tlr7*$^{ki/wt}$ female mice (*n*=6) and *Tlr7*$^{ki/ki}$ female mice (*n*=5) were treated with indicated dose of IMQ for 4 days.

(A) Phenotypic presentation (*top*) and H&E staining (*bottom*) of dorsal skin. Scale bar: 100 μm. Left: H&E staining data; right: statistical data (mean ± SEM).

(B) Clinical scores plotted with mean ± SEM. *denotes statistical significance when compared with the *Tlr7*$^{wt/wt}$ group.

(C) Body weight change plotted with mean ± SEM. *denotes statistical significance when compared with the *Tlr7*$^{wt/wt}$ group.

(D) Quantitative PCR analysis of mRNA encoding IL-23/IL-17A axis cytokines and other psoriasis-related cytokines in the dorsal skin. Results were normalized to *Gapdh* expression.

(E) Representative p-p65 staining of the dorsal skin. Scale bars: 100 μm.

Data information: Data are represented mean ± SEM. *P* values are determined by two-tailed Mann-Whitney *U* test (A, D) or Tukey multiple comparison test (B, C). *$P<0.05$, **$P<0.01$.

10) Fig S16: Authors indicate that ubiquitination of TLR7 is increased when SHP2 is overexpressed; however, if the role of SHP2 is to increase TLR7 expression in general, wouldn't the increase in ub be an indirect consequence based on this fact? It seems therefore that the ub of TLR7 may be strictly a consequence of there being more of it in the cell, and not directly due to regulation by SHP2.

Reply: It is a very nice suggestion. We demonstrated by Western blots that SHP2 did not affect the expression of TLR7.

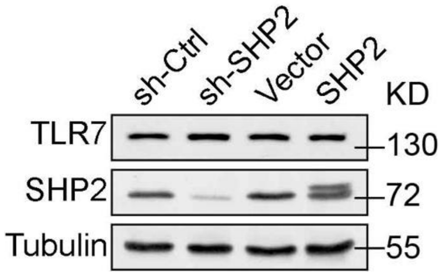


Response Figure 29. SHP2 unchanged protein level of TLR7.

Immunoblot analysis of PMA-differentiated THP-1 cells with shRNA-Control, shRNA-SHP2, vector or SHP2 lentivirus lysates.

11) What about other TLR proteins? Likely SHP2 regulates TLR 3, 8, 9, etc as well; authors should demonstrate the effects of SHP2 and its inhibition on these proteins as well.

Reply: It is a very nice suggestion, and we have found that SHP2 negatively regulated the production of inflammation-related factors in TLR4 and TLR3/8/9 signal transduction in macrophage. Small molecule CU-CPT17e is capable of activating TLRs 3, 8 and 9 simultaneously (Zhang *et al*, 2017, PMID: 28537961). In addition, Xuetao Cao and his colleagues documented that SHP2 negatively regulates TRIF-dependent type I interferon production in TLR4 and TLR3 signal transduction in macrophages (An et al, 2006, PMID: 17157040).

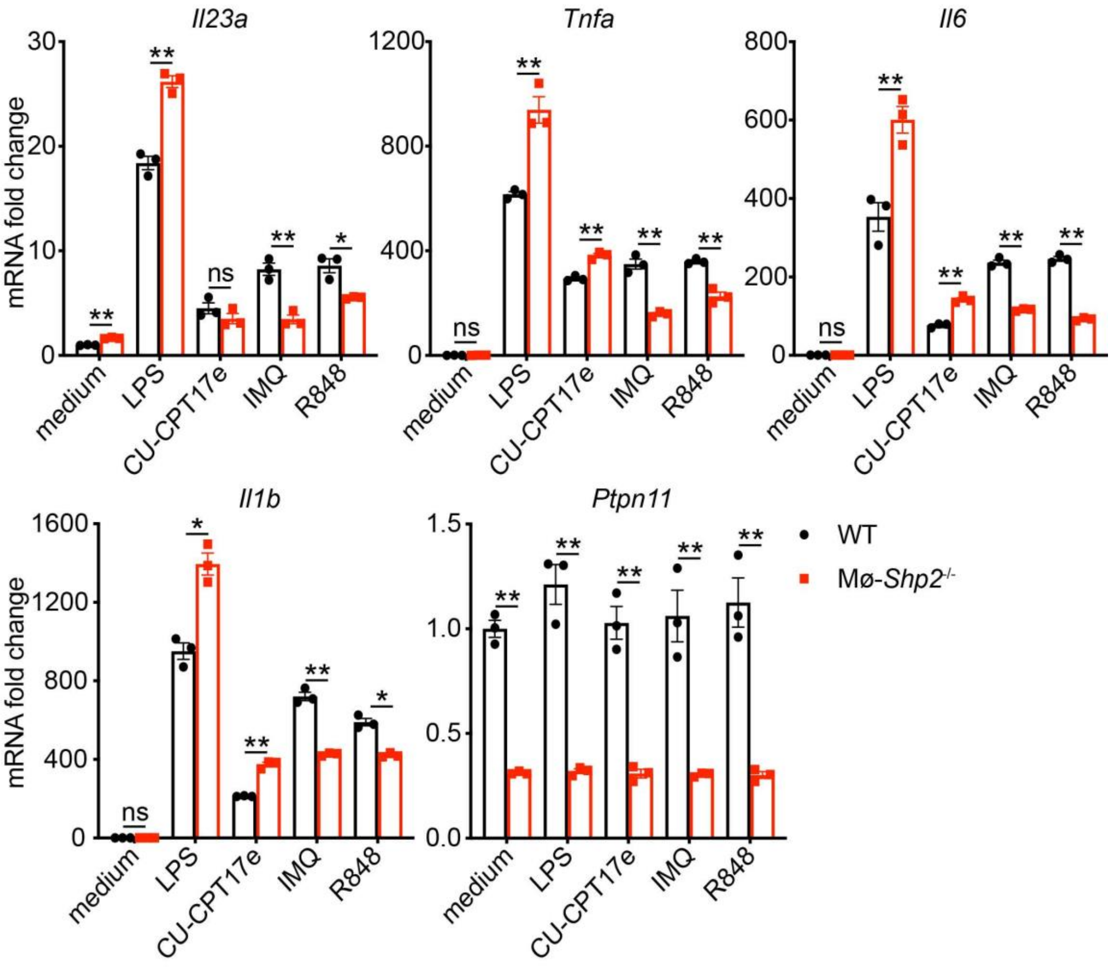


Response Figure 30. SHP2 negatively regulated the production of inflammation-related factors in TLR4 and TLR3/8/9 signal transduction in macrophage.

Expression levels of representative psoriasis-related genes of peritoneal macrophages derived from wild-type and Mø-*Shp2*$^{-/-}$ mice treated with or without LPS (100 ng/ml), CU-CPT17e (10 μM), IMQ (10 μg/ml) or R848(10 μg/ml) for 6 h.

Data information: Data are represented mean ± SEM. *P* values are determined by Tukey multiple comparison test. **P*<0.05, ***P*<0.01, ns, not significant.

In addition, effects on ACT1 should also be shown.

Reply: It has been reported in the literature that ACT1 cooperates with TRAF6 to cause NF-*κ*B activation in the IL-1/Toll signaling pathway (Huang *et al*, 2007, PMID: 17982039; Kanamori *et al*, 2002, PMID: 12459498). In our study, the protagonist was SHP2, and the interacting protein focused on TLR7, without examining the case of ACT1. Your advice about effects of SHP2 on ACT1 is a constructive suggestion. In view of the high competition in this research field, we hope that the current findings would be published as early as possible. Of course, we will absolutely adopt your suggestion in future research. Thank you again.

12) All western blot analyses should be quantified, with statistics, and represented by results from at least 3 independent experimental replicates.

Reply: We appreciate this constructive question raised by the reviewer. According to the reviewer's suggestion, we have displayed and grayed out the original strips of Western blots that appear in the manuscript.

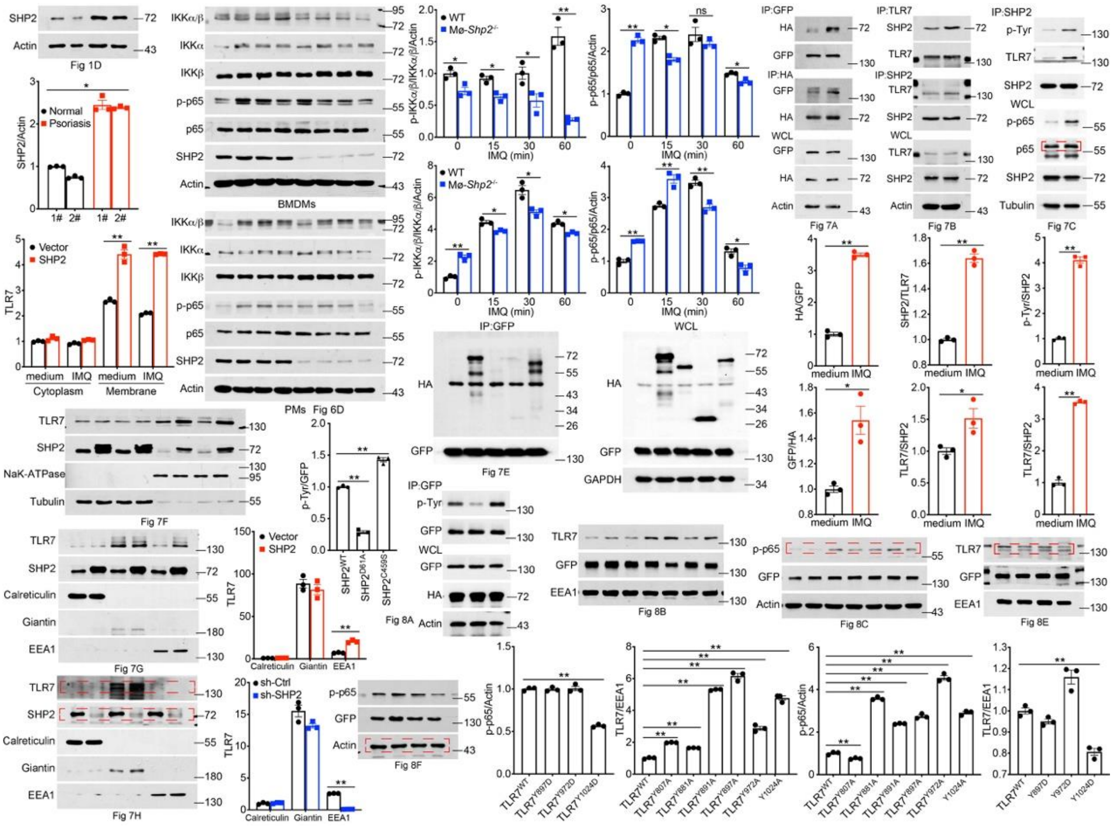


Response Figure 11. Raw data for Figure 1, Figure 6, Figure 7 and Figure 8. Panels corresponding to the figures in the manuscript are indicated.

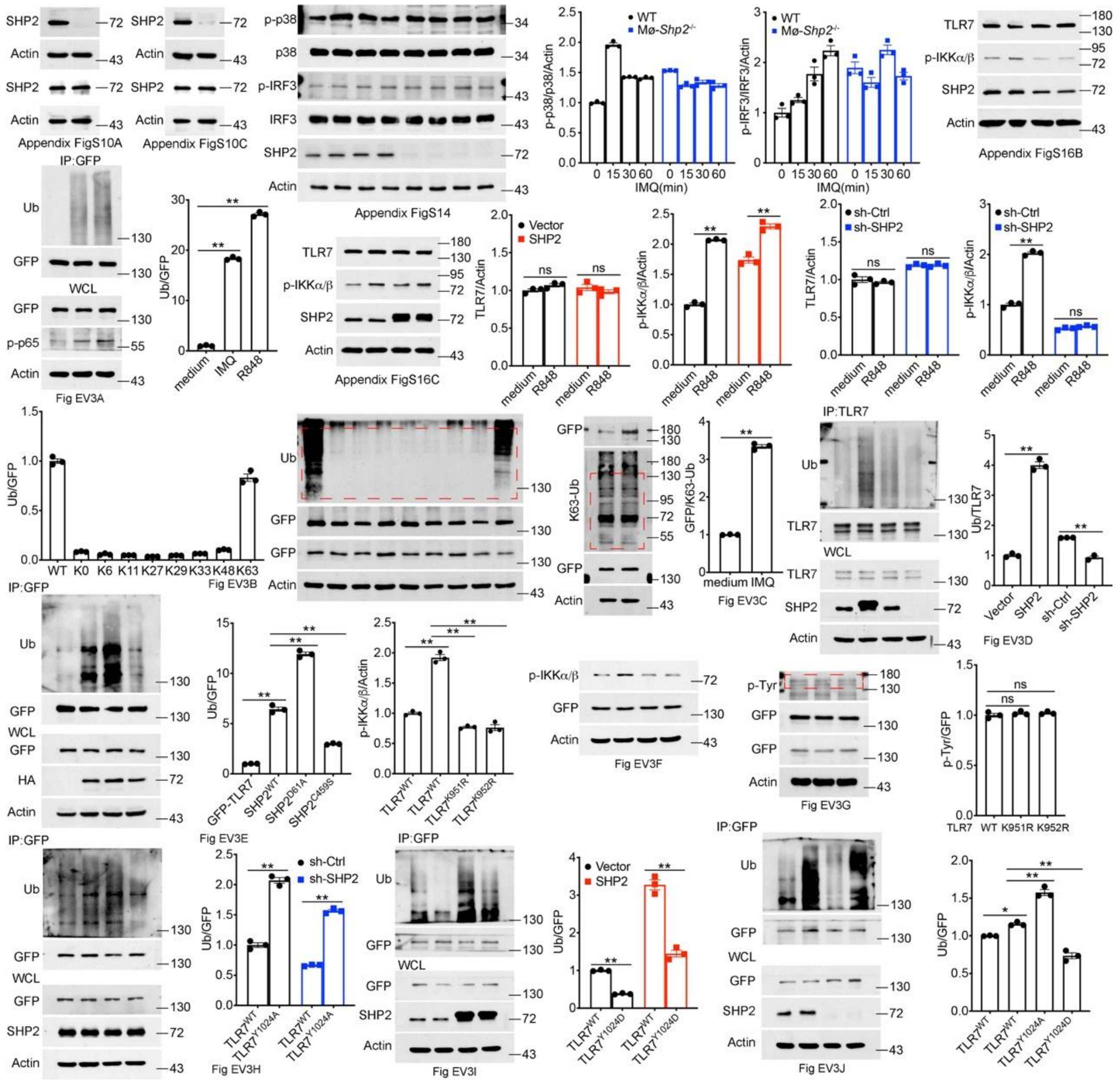


Response Figure 12. Raw data for Figure EV3, Appendix Fig S10, Appendix Fig S14 and Appendix Fig S16. Panels corresponding to the figures in the manuscript are indicated.

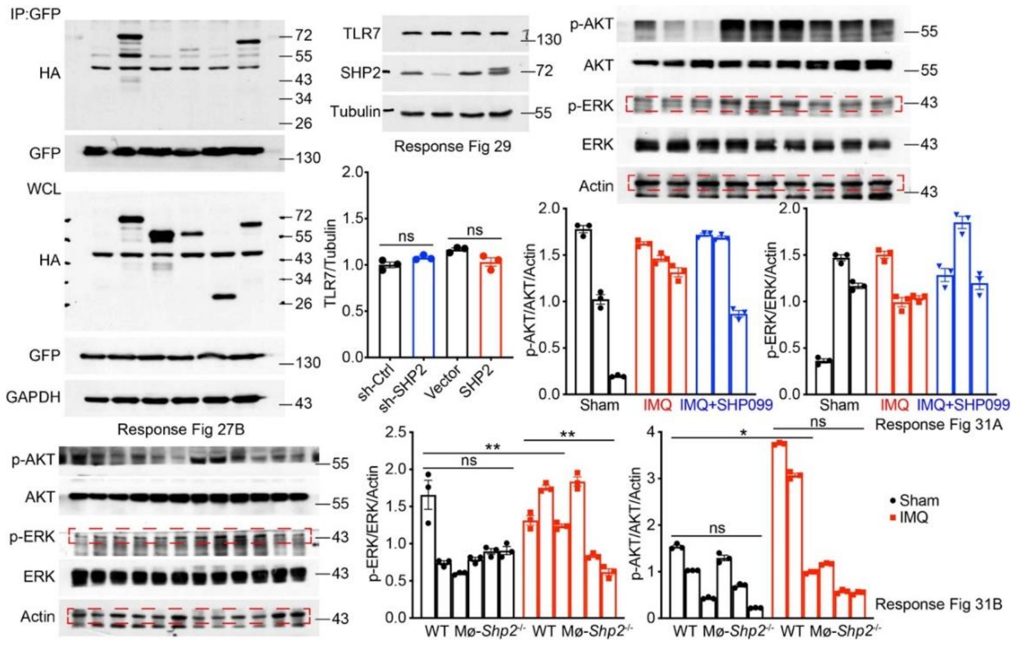

Response Figure 13. Raw data for Response Figures. Panels corresponding to the figures in the respond letter are indicated.

As well, downstream activities of SHP2 in response to IMQ or in psoriasis in the presence or absence of SHP099 should be shown by western blot (p-ERK and p-AKT).

Reply: We have now performed experiments according to the reviewer's suggestions and found that SHP099 did not affect p-AKT and p-ERK expression, whereas knockout of SHP2 in macrophages inhibited AKT and ERK phosphorylation levels.

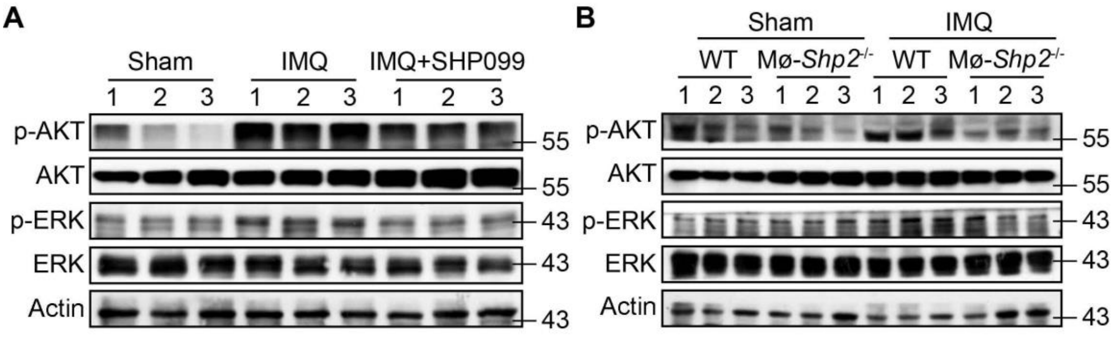


Response Figure 31. Knockout of SHP2 in macrophages inhibited AKT and ERK phosphorylation levels.

(A and B) Western blot analysis of Phospho- or total AKT and ERK in dorsal skin derived from SHP099 treated mice (A) or SHP2 knockout mice (B).

13) Number of mice used for omics data is not indicated.

Reply: We apologize for the lack of clarity in the original manuscript, which we have added to the revised manuscript.

In the RNA-seq experiment, each group included three mice. In single-cell transcriptome sequencing, each group consists of two samples, including two mice per sample in the sham group and three mice in the model and SHP099 administration groups.

Minor concerns:

1) Grammatical corrections are needed throughout.

Reply: We are grateful for pointing out this error and have corrected it.

2) Scale bars for most histological sections are missing.

Reply: We thank the referee for this comment and have added scale bars in the revised manuscript. We put only one scale bar in each panel.

3) Several figures have missing x or y axis labels.

Reply: We have checked through the manuscript and corrected them.

4) ELISA and qRT-PCR data should be measured for fold change (not absolute) and normalized to gapdh or other housekeeping genes.

Reply: Thank you for this comment and we have corrected them in revised manuscript.

## References


An H, Zhao W, Hou J, Zhang Y, Xie Y, Zheng Y, Xu H, Qian C, Zhou J, Yu Y, et al (2006) SHP-2 phosphatase negatively regulates the TRIF adaptor protein-dependent type I interferon and proinflammatory cytokine production. Immunity 25: 919–928

Cheng W-J, Korinek M, Lin C-Y & Hwang T-L (2019) Neutrophils in Psoriasis. Front Immunol 10: 2376

Chiang C-C, Cheng W-J, Korinek M, Lin C-Y & Hwang T-L (2019) Neutrophils in Psoriasis. Front Immunol 10: 2376

Huang F, Kao C-Y, Wachi S, Thai P, Ryu J & Wu R (2007) Requirement for both JAK-mediated PI3K signaling and ACT1/TRAF6/TAK1-dependent NF-kappaB activation by IL-17A in enhancing cytokine expression in human airway epithelial cells. J Immunol 179: 6504–6513

Jiang M, Fang H, Shao S, Dang E, Zhang J, Qiao P, Yang A & Wang G (2019) Keratinocyte exosomes activate neutrophils and enhance skin inflammation in psoriasis. FASEB J 33: 13241–13253

Kanamori M, Kai C, Hayashizaki Y & Suzuki H (2002) NF-κB activator Act1 associates with IL-1/Toll pathway adaptor molecule TRAF6. FEBS Letters 532: 241–246

Katayama H (2018) Development of psoriasis by continuous neutrophil infiltration into the epidermis. Exp Dermatol 27: 1084–1091

Lin AM, Rubin CJ, Khandpur R, Wang JY, Riblett M, Yalavarthi S, Villanueva EC, Shah P, Kaplan MJ & Bruce AT (2011) Mast cells and neutrophils release IL-17 through extracellular trap formation in psoriasis. J Immunol 187: 490–500

Wang W-M & Jin H-Z (2020) Role of Neutrophils in Psoriasis. J Immunol Res 2020: 3709749

Wójcik P, Garley M, Wroński A, Jabłońska E & Skrzydlewska E (2020) Cannabidiol Modifies the Formation of NETs in Neutrophils of Psoriatic Patients. Int J Mol Sci 21: E6795

Zhang L, Dewan V & Yin H (2017) Discovery of Small Molecules as Multi-Toll-like Receptor Agonists with Proinflammatory and Anticancer Activities. J Med Chem 60: 5029–5044


Thanks again for the Editor and Reviewers' precious comments and suggestions.

8th Oct 2021

Dear Prof. Sun,

Thank you for the submission of your revised manuscript to EMBO Molecular Medicine. We have now heard back from the two referees who we asked to re-evaluate your manuscript. As you will see from the reports below, while the referees #2 and #3 are supporting publication of your work, referee #1 raises serious concerns that should be addressed in an additional and final round of major revision. Particular attention should be given to the elucidation of the different SHP2 expressing cells and their contribution to the pathogenesis of psoriasis as well as the order of events in TLR7 signaling.

Further consideration of a revision that addresses reviewer's concerns in full will entail an additional round of review. Acceptance or rejection of the manuscript will depend on the completeness of your responses included in the next, final version of the manuscript. For this reason, and to save you from any frustrations in the end, I would strongly advise against returning an incomplete revision.

We would welcome the submission of a revised version within three months for further consideration. Please let us know if you require longer to complete the revision.

I look forward to seeing a revised form of your manuscript. Use this link to login to the manuscript system and submit your revision: https://embomolmed.msubmit.net/cgi-bin/main.plex

Yours sincerely,
Zeljko Durdevic

***** Reviewer's comments *****

Referee #1 (Comments on Novelty/Model System for Author):

The revision has not really significantly improved the study and it is even more confusing at the current stage

Referee #1 (Remarks for Author):

The authors provide new data to strengthen their hypotheses and answer the questions of the reviewers. However, a number of questions raised by the reviewer have not been fully addressed. The authors show convincing evidence that Shp2 plays a role in the pathogenesis of psoriasis in patients and in the IMQ model of psoriasis-like skin inflammation. There is solid data on the role of Shp2 in phosphorylating TLR7 and this has been mechanistically investigated in large detail. However, the series of events and the cell types involved remain a matter of debate and some of the additional data provided even highlight this criticism. Thus the authors should reformulate their hypothesis concerning the contribution of the different Shp2 expressing cell types (not exclusively macrophages) and adapt their model according to the new data provided.

1) The data provided regarding the expression of Ptpn11 by RNAseq analysis from IMQ treated mouse skin shows that macrophages are the cells with the lowest expression of this gene, whereas proliferating immune cells such as dendritic cells and NK cells as well as non-hematopoietic cells express high amounts of Ptpn11 RNA (Response Fig. 1). Also response Fig. 2 confirms LC, DC and NK cells as highest expressers, whereas Response Fig. 9 shows, that macrophages rather express other subtypes such as Ptpn1 and 6.
2) The high levels of ptpn11 in DC makes it important to confirm efficient deletion in the CD11c-Cre mouse model. As already mentioned in the initial review, deletion in BMDC is not sufficient to confirm efficient deletion in the inflamed skin. The authors cite data from the original publication of the CD11c-Cre mouse (Caton et al.). In this paper recombination efficiency for pDC, which are TLR7 positive, is only 86%. This may vary under inflammatory conditions and this also holds true for other DC subpopulations. Furthermore, dermal macrophages may also be CD11c positive. Thus, determining the exact cell type being depleted of Shp2 under inflammatory conditions either by immunofluorescence co-staining or RNA analysis from sorted cells is very important.
In support of their view in the response the authors write: "However, knockout of SHP2 in monocytes only improved psoriasis-like disease in mice, but not in DCs or $\gamma\delta$Th17 cells (Kadekar et al, 2020, PMID: 32092146), suggesting that SHP2 promotes psoriasis progression with monocyte specificity."
However, in this publication only the role of Shp2 in $\gamma\delta$ T-cells has been investigated, other citations are probably missing.

3) The phenotype observed in LysM-Cre /Sph2 fl/fl mice is not necessarily caused by deletion in macrophages since LysM Cre also deletes in other myeloid cells, most prominently in neutrophils, that may also express Shp2. Indeed the authors show in their response unpublished data from S100a8-Cre mice as a neutrophil conditional knockout (Response Fig. 6) that also show a reduced response to IMQ. Although these are data that will not be published in this paper it is still worth questioning, whether other myeloid cells that produce S100a8 under inflammatory conditions, like macrophages, do also show recombination?
4) The authors have now included the results of two new experiments where they have used Shp2 inhibitor in vivo. However, these experiments rather underscore the redundancy of Shp2 signalling in vivo. According to the model presented in the graphical abstract, Shp2 is active downstream of TLR7 in macrophages and is modifying TLR7 activity in these cells. In support of this hypothesis the authors show that IMQ induced IL-23 production depends on Shp2 in myeloid cells (Fig. 5).
However, the new data provided in Fig. EV1 show that systemic Shp2 inhibition rescues also from IL23 injection, indicating, that Shp2 acts also in other cells downstream of IL-23 produced by macrophages. The same holds true for the experiment presented in Appendix Fig. S20. IL-23 treatment in the phosphatase resistant TLRKI mice results in reduced ear inflammation compared to TLR7wt mice, indicating, that TLR7 signalling is downstream of IL-23. Thus the authors have to explain the sequence of events more precisely and specify which cells are target of Shp2 under which condition.
5) Fig. 6D: In the WB for pIKKa/b the difference especially in the peritoneal macrophages is hard to see. This should be quantified. The same applies to p-Tyr and p-p65 WBs. This analyses should be performed similar to the quantifications shown for e.g. GFP/HA.

minor points:
Appendix Fig. 15: This shows nicely nuclear p65 staining in CD68+ cells in human skin. Although there is a higher number of macrophages in psoriatic skin it appears that the percentage of p65+ within the CD68+ cells does not seem to be overtly different. This should be discussed.
Fig. 8I: In this figure staining of Y1024 on human TLR7 is shown. Interestingly in psoriatic skin also most of the epidermal cells are stained positive indicating that TLR7 is strongly expressed on keratinocytes. This is an interesting finding and should be discussed.
Response Fig. 2: here the sham population is not visible??

Fig. legend Fig.6B: The legend is unclear. It doesn´t say in which of the two conditions the genes are up or downregulated: "Volcano plot image of upregulated (in red) and down regulated (in green) genes in peritoneal macrophages from wild-type and Mø-Shp2-/- 288 mice after IMQ treatment (n=3/group)."

Appendix S8: the labelling of the X-axes in Figs A and C is not clear. Is this log transformed? Why are the levels negative?
Appendix Fig.12: To show that F4/80 + cells are indeed Shp2 positive higher magnifications need to be shown. The same holds true for Fig. S18 supposed to show subcellular localization of TLR7 in human psoriatic skin.
Furthermore, LAMP1 is not a good marker for early endosomes but rather for late endosomes / lysosomes. Thus TLR7 is not only found upregulated in early endosomes, this should be corrected in the text.
The references for Fig. EV3 are mislabeled as EV4.

Referee #2 (Remarks for Author):

Is suitable for publication.

Referee #3 (Remarks for Author):

Authors did extensive revisions that now satisfactorily address previous concerns.

Dear Editor and reviewers,
Thank you and reviewers for the valuable time and insightful comments. We appreciate the opportunity to revise and improve our manuscript. We have performed additional experiments and revised our manuscript according to the reviewers' comments and suggestions. All changes have been highlighted in **Red** in the revised files.
The following secure token has been created to allow review of record GSE165021 while it remains in private status: gvmxwmcuvhohnov.

***** Reviewer's comments *****

Referee #1 (Comments on Novelty/Model System for Author):

The revision has not really significantly improved the study and it is even more confusing at the current stage

Referee #1 (Remarks for Author):

The authors provide new data to strengthen their hypotheses and answer the questions of the reviewers. However, a number of questions raised by the reviewer have not been fully addressed. The authors show convincing evidence that Shp2 plays a role in the pathogenesis of psoriasis in patients and in the IMQ model of psoriasis-like skin inflammation. There is solid data on the role of Shp2 in phosphorylating TLR7 and this has been mechanistically investigated in large detail. However, the series of events and the cell types involved remain a matter of debate and some of the additional data provided even highlight this criticism. Thus the authors should reformulate their hypothesis concerning the contribution of the different Shp2 expressing cell types (not exclusively macrophages) and adapt their model according to the new data provided.
Reply: Thank you for your positive evaluation and encouragement. We have reformulated our hypothesis according to the new data provided.
1) The data provided regarding the expression of Ptpn11 by RNAseq analysis from IMQ treated mouse skin shows that macrophages are the cells with the lowest expression of this gene, whereas proliferating immune cells such as dendritic cells and NK cells as well as non-hematopoietic cells express high amounts of Ptpn11 RNA (Response Fig. 1). Also response Fig. 2 confirms LC, DC and NK cells as highest expressers, whereas Response Fig. 9 shows, that macrophages rather express other subtypes such as Ptpn1 and 6.
Reply: We thank the reviewer for this insightful question. Regarding the expression of *Ptpn11*, *Ptpn1*, and *Ptpn6* in immune cells, it was analyzed based on the sham, IMQ, IMQ+SHP099 three groups of integrated data. The expression of the genes is only one aspect, as validated by multiple conditional knockout mice, it was found that knockout of SHP2 in macrophages and neutrophils reduced psoriasis-like symptoms in mice. In contrast, knockdown of SHP2 in $\gamma\delta$Th17 cells and dendritic cells did not

affect the progression of psoriasis in mice. Thank you for your suggestion and we will investigate the role of *Ptpn1* and *Ptpn6* in macrophages in our follow-up experiments. To better answer this question, we have conducted the following experiments.

We download RNA-seq data from GSE54456 which sequences polyadenylated RNA-derived cDNAs from 92 psoriatic and 82 normal punch biopsies. CIBERSORT (Newman *et al*, 2015) was used to distinguish 22 human hematopoietic cell phenotypes, including seven T-cell types, naïve and memory B cells, plasma cells, natural killer (NK) cells, and myeloid subsets from the RNA-seq data. R script was downloaded from the website https://rdrr.io/github/singha53/amritr/src/R/supportFunc_cibersort.R and 22 cell gene sets was pulled from this article's Supplementary table 1 (Wang *et al*, 2021). The cell types were removed which were predicted in less than half of the samples. Thus, there were only 13 cell types manifested in the results. Obviously, the expression of multiple immune cells was elevated in the lesional psoriatic skin, including M1 macrophages, neutrophils, dendritic cells, T cells, and monocytes (Response Figure 1).

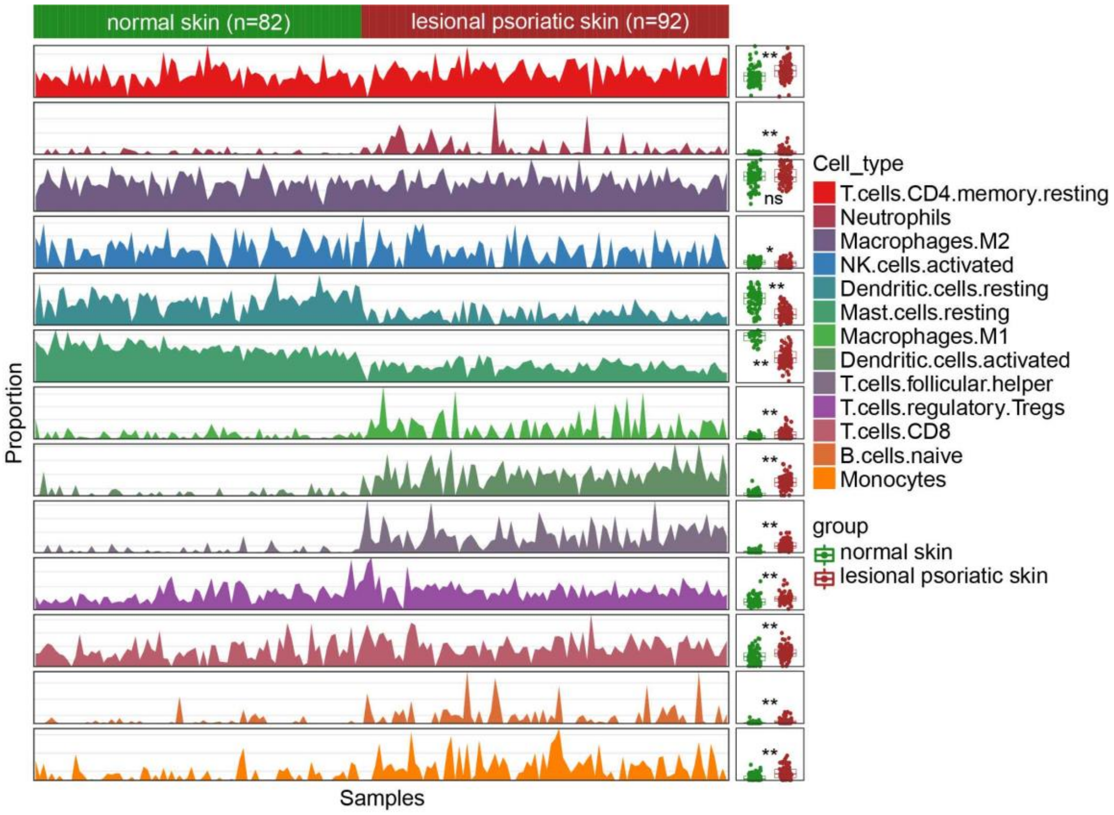


Response Figure 1. Multiple immune cells highly expressed in psoriatic lesions.

AreaPlot showing the estimated cell type proportion among 92 psoriatic and 82 normal skin samples sequenced by bulk RNA-seq (No. GSE54456). Boxplot depicting relative estimated cell percentage scores (*right*).

Data information: *P* values were calculated by Wilcox test. **P*<0.05, ***P*<0.01, ns, not significant.

We performed spatial transcriptome sequencing analysis in normal skin and psoriasis patient lesion skin using the section-specific barcodes. We obtained two skin sections from normal control and one lesion skin from psoriasis patient (Appendix Fig S14A and B), we focused on cluster 1 and cluster 3 located in the dermis (Appendix Fig S14C). *PTPN11* was most strongly correlated with macrophages in lesional psoriatic skin (Appendix Fig S14D). And the expression of CD68, which was co-expressed with *PTPN11*, was also upregulated in the skin lesions of psoriatic (Appendix Fig S14E and F). There are many kinds of cells inside each spot, and the differences between the cells in the spots in Cluster 1 and Cluster 3 of normal control and psoriatic patient are obvious. Normal control had only a few macrophages, whereas psoriatic patient had a large infiltration of macrophages (Appendix Fig S14G), which is consistent with the results of Appendix Fig S12A. Strong cellular interactions, especially between macrophages and fibroblasts, were present in Cluster 1 and Cluster 3 of psoriatic patient (Appendix Fig S14H). The above demonstrates that *PTPN11* in macrophages plays an important role in psoriasis progression.

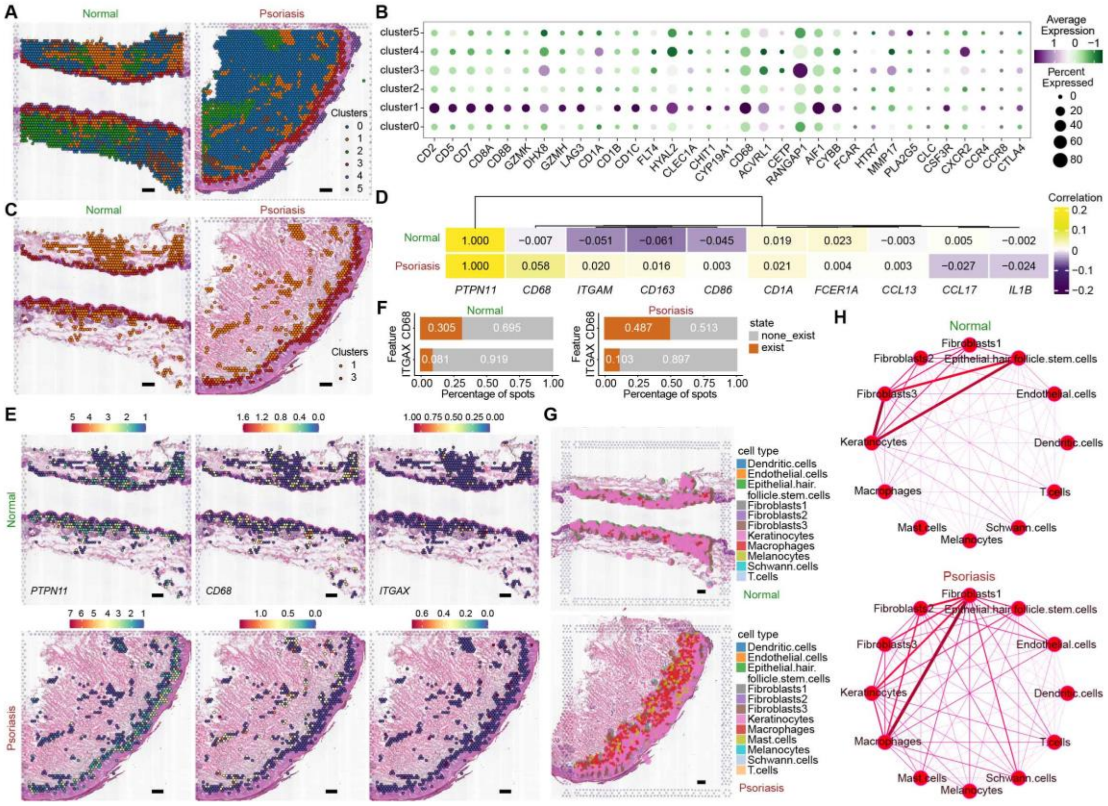


Appendix Fig S14. Spatial transcriptome sequencing analysis revealed that *PTPN11* was most strongly correlated with macrophages in lesional psoriatic skin.

A Six clusters reflecting six anatomical regions on the psoriasis slice and the normal slice. Scale bar: 500 µm.

B Dotplot showing some immune cell marker genes of all cell clusters in (A).

C Prominence of Cluster1 and Cluster3 in the dermis of psoriatic slice and normal slice. Scale bar: 500 µm.

D Heatmap showing the correlation coefficient between *PTPN11* and other genes in two groups respectively.

E Only spots expressing *PTPN11* shown in two slices (*top*) with the expression of *CD68* (*medium*) and *ITGAX* (*bottom*) on these spots. Scale bar: 500 μm.

F Barplot showing the percentage of the spots which expressing *CD68* and *ITGAX* in all spots expressing *PTPN11*, both shown in normal and psoriasis slices. Significant differences between the two groups were calculated by Fisher's exact test. The normal group was 7.623e-23, and the psoriasis was 4.542e-49.

G Spots in Cluster1 and Cluster3 showing the percentage of cell types by Scatterpieplot. Scale bar: 500 μm.

H The cell-cell interaction network between cell types shown in (G). The thickness of the connecting line indicates the strength of the interaction.

Furthermore, we performed scRNA-seq on unsorted cells from normal skin and lesional psoriatic skin. After performing unsupervised clustering and a UMAP plot analyses (Appendix Fig S17A), cluster identities were determined by the expression of established markers (Appendix Fig S17B), which indicated successful capture of major skin cell subsets. Heatmap showed that *PTPN11* was positively more correlated with the NF-kappa B pathway in macrophages than dendritic cells from psoriasis lesions (Appendix Fig S17C), suggesting that *PTPN11* exacerbates psoriasis mainly in macrophages by activating the NF-kappa B pathway.

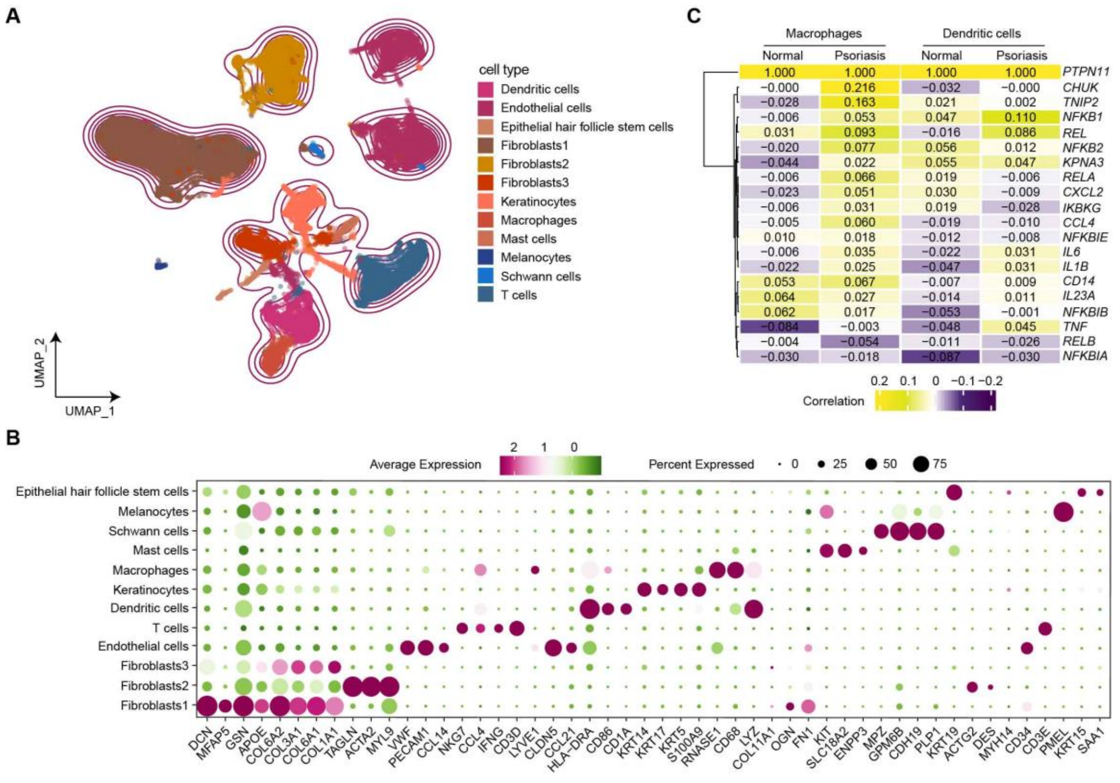


Appendix Fig S17. scRNA-seq analysis revealed that *PTPN11* positively correlated with the NF-kappa B pathway in macrophages of psoriasis lesions.

A Uniform Manifold Approximation (UMAP) plot of all cells colored by their cell type.

B Dotplot showing some marker genes of all cell types in (A).

C Heatmap showing the correlation coefficient between *PTPN11* and each gene from NF-kappa B signaling pathway in four groups (Macrophages in Normal, Macrophages in Psoriasis, Dendritic cells in Normal and Dendritic cells in Psoriasis) respectively.

Due to time and financial issues, and the fact that they are all clinical samples, we have a limited number of samples for testing, 2 per group for scRNA-seq and 1 per group for spatial transcriptomics.
We have updated these data in Appendix Figure S14 and Appendix Figure S17 in our revised manuscript.

2) The high levels of ptpn11 in DC makes it important to confirm efficient deletion in the CD11c-Cre mouse model. As already mentioned in the initial review, deletion in BMDC is not sufficient to confirm efficient deletion in the inflamed skin. The authors cite data from the original publication of the CD11c-Cre mouse (Caton et al.). In this paper recombination efficiency for pDC, which are TLR7 positive, is only 86%. This may vary under inflammatory conditions and this also holds true for other DC subpopulations. Furthermore, dermal macrophages may also be CD11c positive. Thus, determining the exact cell type being depleted of Shp2 under inflammatory conditions either by immunofluorescence co-staining or RNA analysis from sorted cells is very important.
Reply: We appreciate this constructive question raised by the reviewer. According to the reviewer's suggestion, we demonstrated that SHP2 was knocked down in dendritic cells of DC-*Shp2*$^{-/-}$ mice skin tissue in the disease state, but not in macrophages.

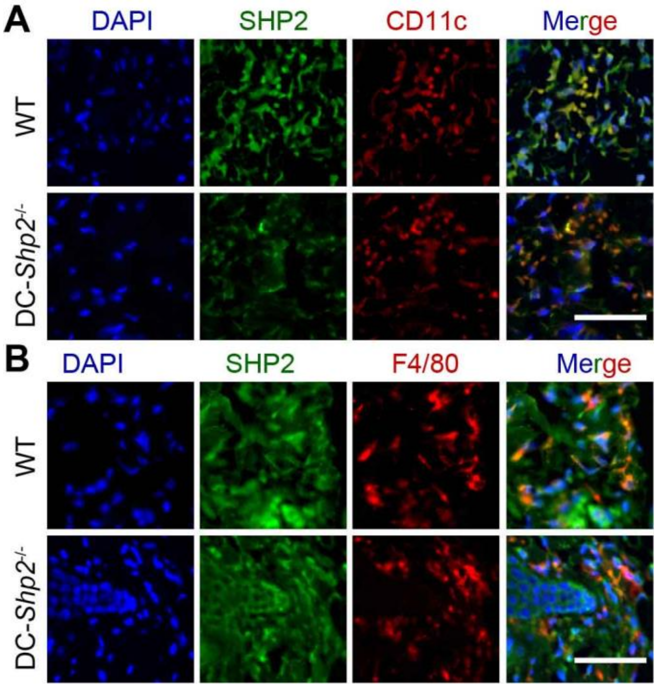


Response Figure 2. Deletion efficiency of SHP2 in DC-*Shp2*$^{-/-}$ mice.

(A and B) Immunofluorescence staining of skin sections from wild-type mice and DC-*Shp2*$^{-/-}$ mice with SHP2 and CD11c (A) or F4/80 (B), respectively showing SHP2 expression in dendritic cells or macrophages. Scale bar: 50 µm.

We fully affirm the key role of DCs in psoriasis. Our study found a more correlation between SHP2 and NF-kappa B in macrophages than in DCs (Appendix Fig S17C), which to some extent suggests that SHP2 plays different roles in macrophages and DCs.

In support of their view in the response the authors write: "However, knockout of SHP2 in monocytes only improved psoriasis-like disease in mice, but not in DCs or 훼훼 Th17 cells (Kadekar et al, 2020, PMID: 32092146), suggesting that SHP2 promotes psoriasis progression with monocyte specificity."

However, in this publication only the role of Shp2 in 훼훼 T-cells has been investigated, other citations are probably missing.

Reply: We are sorry for this confusion. The data for DC conditional knockout mice are the result of our experiments and therefore only $\gamma\delta$Th17 cells are cited in the literature.

3) The phenotype observed in LysM-Cre /Sph2 fl/fl mice is not necessarily caused by deletion in macrophages since LysM Cre also deletes in other myeloid cells, most prominently in neutrophils, that may also express Shp2. Indeed the authors show in their response unpublished data from S100a8-Cre mice as a neutrophil conditional knockout (Response Fig. 6) that also show a reduced response to IMQ. Although these are data that will not be published in this paper it is still worth questioning, whether other myeloid cells that produce S100a8 under inflammatory conditions, like macrophages, do also show recombination?

Reply: Thank you for this constructive advice. We demonstrated that SHP2 was knocked down in neutrophils of S100A8-cre:*Shp2*$^{fl/fl}$ mice skin tissue in the disease state, but not in macrophages.

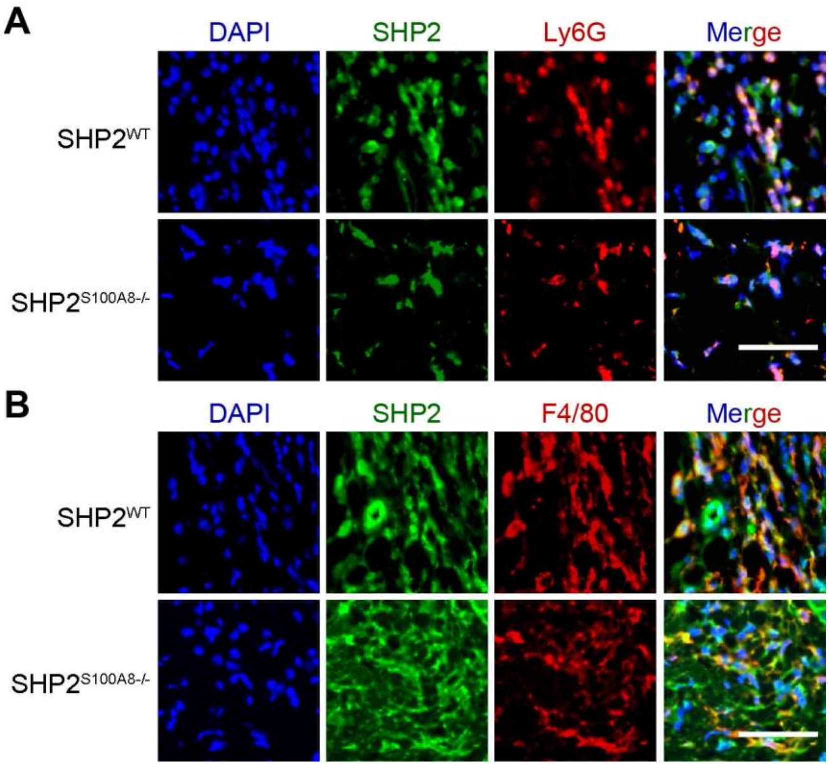


Response Figure 3. Deletion efficiency of SHP2 in SHP2$^{S100A8-/-}$ mice.

(A and B) Immunofluorescence staining of skin sections from wild-type mice and SHP2$^{S100A8-/-}$ mice with SHP2 and Ly6G (A) or F4/80 (B), respectively showing SHP2 expression in neutrophils or macrophages. Scale bar: 50 μm.

4) The authors have now included the results of two new experiments where they have used Shp2 inhibitor in vivo. However, these experiments rather underscore the redundancy of Shp2 signaling in vivo. According to the model presented in the graphical abstract, Shp2 is active downstream of TLR7 in macrophages and is modifying TLR7 activity in these cells. In support of this hypothesis the authors show that IMQ induced IL-23 production depends on Shp2 in myeloid cells (Fig. 5).
However, the new data provided in Fig. EV1 show that systemic Shp2 inhibition rescues also from IL23 injection, indicating, that Shp2 acts also in other cells downstream of IL-23 produced by macrophages. The same holds true for the experiment presented in Appendix Fig. S20. IL-23 treatment in the phosphatase resistant TLRKI mice results in reduced ear inflammation compared to TLR7wt mice, indicating, that TLR7 signaling is downstream of IL-23. Thus the authors have to explain the sequence of events more precisely and specify which cells are target of Shp2 under which condition.
Reply: Thank you for your question. TLR7 is the upstream of IL-23, but not the only one. When IL-23 cytokine is injected in the skin of mice, the inflammatory response is triggered and the inflammatory factors exert action on a variety of cells including macrophages. Macrophages are stimulated by TLR7 agonists and IL-23 expression is significantly upregulated. In contrast, IL-23 stimulation of macrophages did not affect TLR7 expression. This experiment demonstrates that TLR7 is the upstream of IL-23.

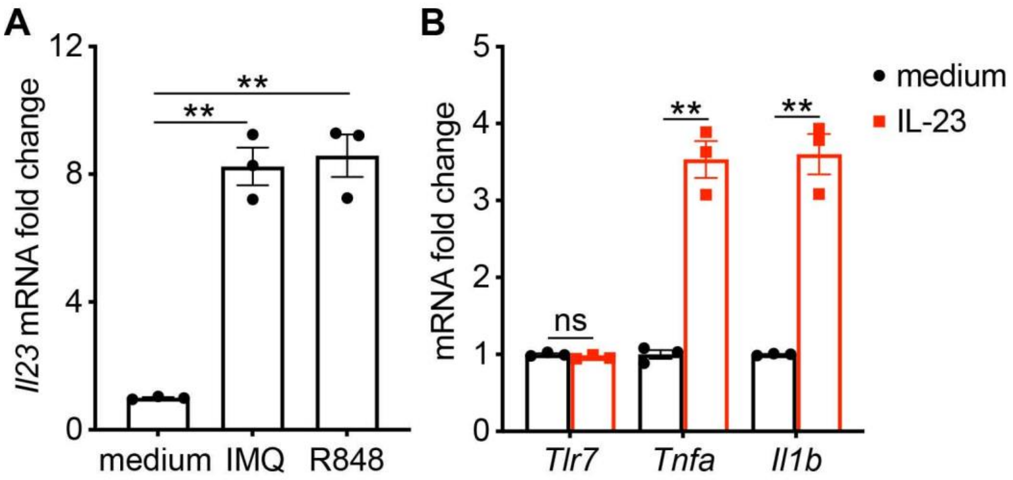


Response Figure 4. TLR7 is the upstream of IL-23.

Expression levels of indicated genes of peritoneal macrophages derived from C57BL/6 mice treated with or without IMQ (10 μg/ml) or R848(10 μg/ml) (A) or IL-23 (100 ng/ml) (B).

Data information: Data are represented mean ± SEM. *P* values are determined by Tukey multiple comparison test. ***P*<0.01, ns, not significant.

5) Fig. 6D: In the WB for pIKKa/b the difference especially in the peritoneal macrophages is hard to see. This should be quantified. The same applies to p-Tyr and p-p65 WBs. This analyses should be performed similar to the quantifications shown for e.g. GFP/HA.

Reply: It is a very nice suggestion. In the last version of the revised manuscript we have quantitatively the WB strips, and we are sorry about the wrong labeling in Figure 8C and Figure 8F, the y-axis is actually p-p65/GFP, and we have made a correction. Thanks again for your carefulness!

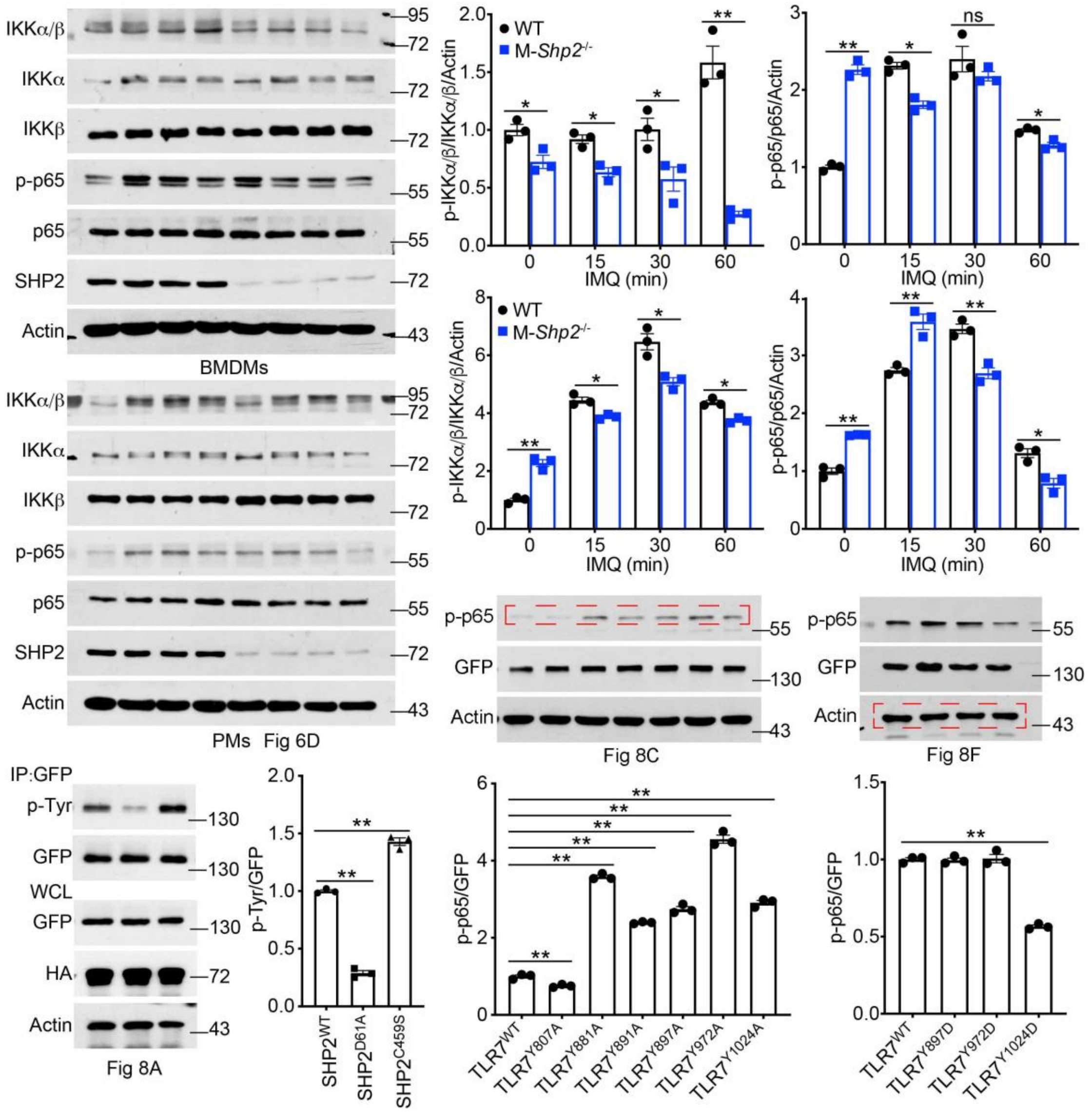


Response Figure 5. Raw data for Figure 6D, Figure 8A, Figure 8C, and Figure 8F. Panels corresponding to the figures in the manuscript are indicated.

minor points:

Appendix Fig. 15: This shows nicely nuclear p65 staining in CD68$^+$ cells in human skin. Although there is a higher number of macrophages in psoriatic skin it appears that the percentage of p65$^+$ within the CD68$^+$ cells does not seem to be overtly different. This should be discussed.

Reply: We thank the reviewer for this insightful question. Despite the small difference in proportion of p65$^+$ within the CD68$^+$ cells between the normal and psoriasis, both the infiltration of macrophages and the expression of p-p65 were significantly higher in the lesions of psoriasis patients than in healthy individuals, and this is important in the progression of psoriasis.

Fig. 8I: In this figure staining of Y1024 on human TLR7 is shown. Interestingly in psoriatic skin also most of the epidermal cells are stained positive indicating that

TLR7 is strongly expressed on keratinocytes. This is an interesting finding and should be discussed.
Reply: We thank the reviewer for this insightful question. Abnormal epidermal proliferation is a typical feature of psoriasis, where TLR7 may be involved in the expansion of inflammation in psoriasis, exacerbating psoriasis by activating the NF-kappa B pathway to upregulate the expression of multiple inflammatory factors. It may also bind to antimicrobial peptide LL37 and others to trigger the inflammatory response of immune cells. Or it may be involved in the initiation of psoriasis, which requires extensive experiments to confirm.

Response Fig. 2: here the sham population is not visible??
Reply: We apologize for the incorrect description. Response Fig. 2 is a mixture of boxplot and violinplot, indicating *Ptpn11* expression in mouse skin immune cells.

Fig. legend Fig. 6B: The legend is unclear. It doesn´t say in which of the two conditions the genes are up or downregulated: "Volcano plot image of upregulated (in red) and down regulated (in green) genes in peritoneal macrophages from wild-type and Mø-Shp2-/- 288 mice after IMQ treatment (n=3/group)."
Reply: We are grateful for pointing out this error and have corrected it.

Appendix S8: the labelling of the X-axes in Figs A and C is not clear. Is this log transformed? Why are the levels negative?
Reply: We are sorry for this confusion. As you said, it is indeed log transformed. We have corrected it in the revised manuscript.

Appendix Fig. 12: To show that F4/80$^{+}$ cells are indeed Shp2 positive higher magnifications need to be shown. The same holds true for Fig. S18 supposed to show subcellular localization of TLR7 in human psoriatic skin.
Reply: We are sorry for these poor IF images in the first revised manuscript and have updated the images.
Appendix Figure S18 in the first revised manuscript have updated to Appendix Figure S20 in this revised manuscript.

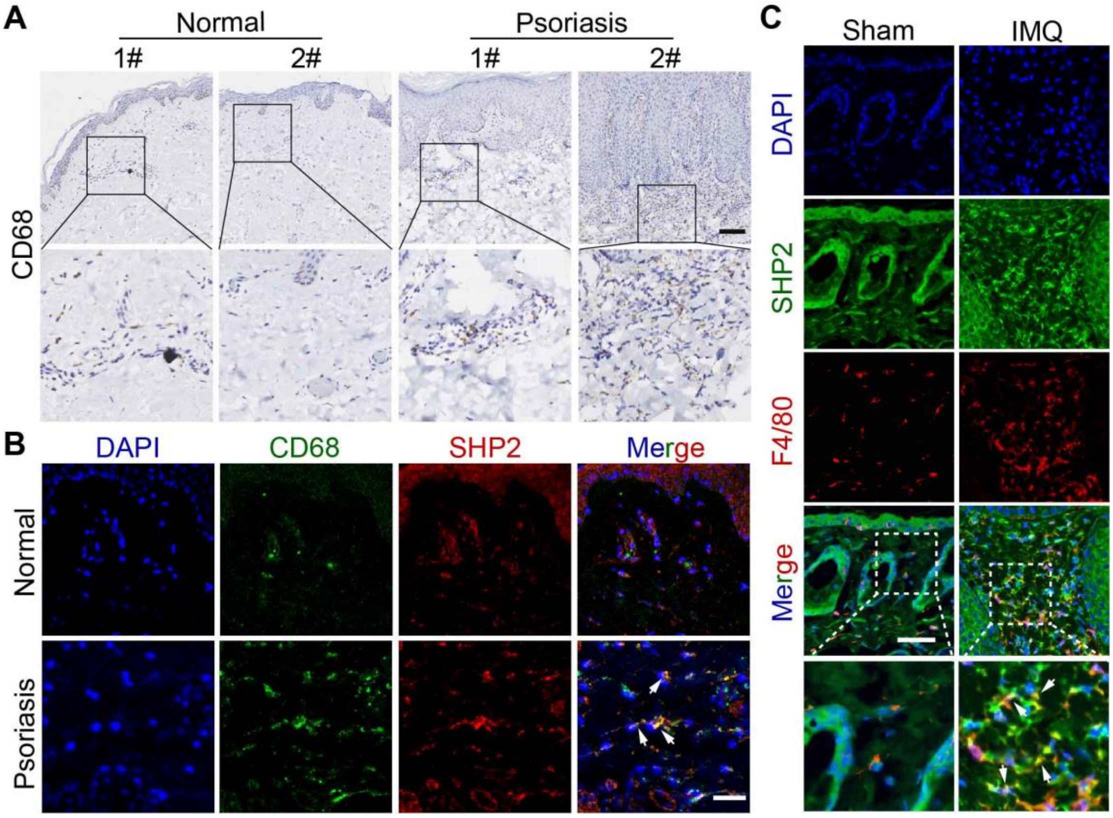


Appendix Fig S12. SHP2 highly expressed in the infiltrated macrophages in both psoriatic skin and IMQ-induced murine skin.

A Representative CD68 staining of skin sections from psoriatic patients (n=13) and normal controls (n=5). Scale bar: 200 μm.

B Immunofluorescence staining of skin sections from psoriatic patients and normal controls with SHP2 and CD68, showing SHP2 expression in macrophages (arrows). CD68, green; SHP2, red. Scale bar: 100 μm.

C Immunofluorescence staining of skin sections from IMQ-induced and sham mice with SHP2 and F4/80, showing SHP2 expression in macrophages (arrows). SHP2, green; F4/80, red. Scale bar: 50 μm.

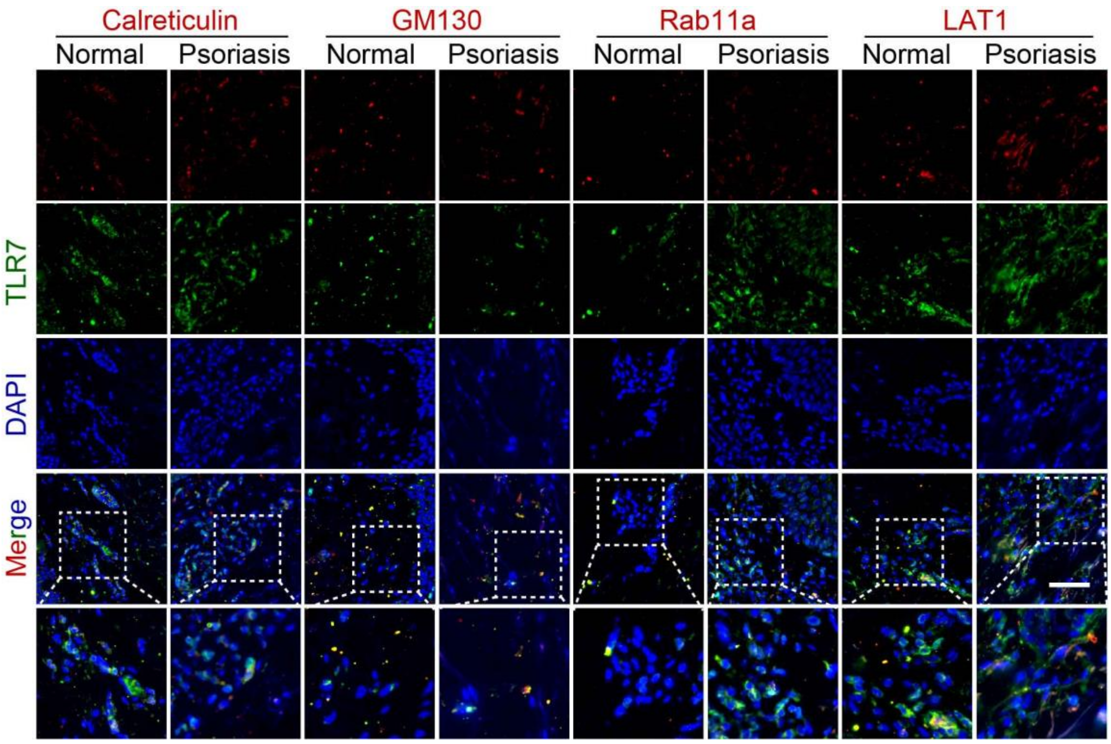

Appendix Fig S20. Cellular localization of TLR7 in psoriatic skin.

Skin sections from psoriasis patients and healthy controls were stained for TLR7 together with a marker of the ER (Calreticulin), Golgi (GM130) and recycling endosome (Rab11a) or cell membrane (LAT1) prior to analysis by confocal microscopy. Scale bar: 50 µm.

Furthermore, LAMP1 is not a good marker for early endosomes but rather for late endosomes / lysosomes. Thus TLR7 is not only found upregulated in early endosomes, this should be corrected in the text.

Reply: We completely agree with you. We have corrected the error in the revised manuscript.

The references for Fig. EV3 are mislabeled as EV4.

Reply: We thank the reviewer for reading our manuscript carefully and pointing out this mistake. We have corrected the error in the revised manuscript.

## References

Newman AM, Liu CL, Green MR, Gentles AJ, Feng W, Xu Y, Hoang CD, Diehn M & Alizadeh AA (2015) Robust enumeration of cell subsets from tissue expression profiles. *Nat Methods* 12: 453–457

Wang H, Chen W, He J, Xu W & Liu J (2021) Network analysis of potential risk genes for psoriasis. *Hereditas* 158: 21

Thanks again for the Editor and Reviewers' precious comments and suggestions.

1st Dec 2021

Dear Prof. Sun,

Thank you for the submission of your revised manuscript to EMBO Molecular Medicine. I am pleased to inform you that we will be able to accept your manuscript pending the following final amendments:

1) Please address all the concerns raised by the referee #1. Particular attention should be given to tuning down the conclusions on exclusive role of Shp2 in macrophages and broaden this to other cell types of the myeloid lineage.
2) In the main manuscript file, please do the following:
- Correct/answer the track changes suggested by our data editors by working from the attached document.
- Remove figures from the main manuscript file and compile figure legends at the end of the manuscript, following by EV figure legends.
- Please make sure that all special characters display well.
- Add callouts for Figure 9F.
- For author contributions please use initials.
- In M&M, include a statement that informed consent from patients was obtained and that the experiments conformed to the principles set out in the WMA Declaration of Helsinki and the Department of Health and Human Services Belmont Report.
- Add full URL to your deposited data and please be aware that all deposited data have to be freely available before publication of the manuscript. Please use the following format to report the accession number of your data:

The datasets produced in this study are available in the following databases:
[data type]: [full name of the resource] [accession number/identifier] ([doi or URL or identifiers.org/DATABASE:ACCESSION])

Please check "Author Guidelines" for more information.
https://www.embopress.org/page/journal/17574684/authorguide#availabilityofpublishedmaterial
- Correct the reference citation. In the reference list, citations should be listed in alphabetical order. Where there are more than 10 authors on a paper, 10 will be listed, followed by "et al.". Please check "Author Guidelines" for more information.
https://www.embopress.org/page/journal/17574684/authorguide#referencesformat
3) Synopsis: Please submit synopsis text as a separate .doc file and remove both synopsis text and image from the main manuscript file. Please check your synopsis text, revise it if necessary and submit its final versions with your revised manuscript. Please be aware that in the proof stage minor corrections only are allowed (e.g., typos).
4) Source data: Please zipp source data for all EV Figures as one file.
5) Funding: Mountain-Climbing Talents Project of Nanjing University is missing in our submission system. Please make sure that information about all sources of funding are complete in both our submission system and in the manuscript.
6) For more information: There is space at the end of each article to list relevant web links for further consultation by our readers. Could you identify some relevant ones and provide such information as well? Some examples are patient associations, relevant databases, OMIM/proteins/genes links, author's websites, etc...
7) As part of the EMBO Publications transparent editorial process initiative (see our Editorial at http://embomolmed.embopress.org/content/2/9/329), EMBO Molecular Medicine will publish online a Review Process File (RPF) to accompany accepted manuscripts. This file will be published in conjunction with your paper and will include the anonymous referee reports, your point-by-point response and all pertinent correspondence relating to the manuscript. Let us know whether you agree with the publication of the RPF and as here, if you want to remove or not any figures from it prior to publication. Please note that the Authors checklist will be published at the end of the RPF.
8) Please provide a point-by-point letter INCLUDING my comments as well as the reviewer's reports and your detailed responses (as Word file).

I look forward to reading a new revised version of your manuscript as soon as possible.

Yours sincerely,

Zeljko Durdevic

***** Reviewer's comments *****

Referee #1 (Remarks for Author):

Zhu et al. have made a tremendous effort in the revision of this manuscript. Overall, we think that the data provided are solid and in general support the hypothesis. Most of our criticism still applies on the authors insisting on an exclusive role of Shp2 in macrophages. On several occasions we have tried to convince the authors to weaken their point and discuss the potential role of Shp2 / ptpn11 also in other cell types. Rather than to accept this, the authors decided to produce and include more and more data into their manuscript, sometimes even data that did not support their hypothesis or raised even new questions.
This applies also to the present revision, which provides very interesting data but again raised more questions than it answered our points. Thus, we again suggest the authors should tune down their conclusion on insisting on the macrophages and broaden

this to other cell types of the myeloid lineage, which by no means would affect the interest and the impact of the paper....

In their reply to the reviewers general comment in the introduction the authors claim that they "reformulated their hypothesis accordingly" however, abstract and graphical abstract have not been changed at all.

previous point 1) New data presented in Appendix Fig. 14 and 17 are valuable, although they do not fully explain the points raised by the reviewer about expression of ptpn11 in other cell types, since the authors give mainly insight into macrophages and dendritic cells but no other cell types.
In App.Fig.14 some questions remain and there is need for more detailed explanations: It is difficult to see the colors of the clusters assigned in A. It is unclear why the authors focus on the two clusters 1 and 3. Whereas Cluster 1 seems to include a number of different immune cell types, the identity of cells contained in Cluster 3 cannot be easily determined from the data provided in panel B. In G the authors show a summary of the distribution of the different cell types as pie charts overlaying the section. However, there is discrepancy between this analysis and the sections shown in E. The keratinocyte contribution seems to be much higher in G (shown in pink). The distribution of CD68 shown in E is not reflected by the representation of macrophages in G. The authors should please check this and if this is correct the underlying possible mechanism for this difference should be explained / discussed.
In App.Fig 17 the authors claim that ptpn11 expression in macrophages but not so much in DC correlates with expression of genes of the NfKB pathway. Again, information on the other cell types would be important.
previous point 2) In response to point 2 the authors provide IF stainings to show deletion in DC or macrophages in DC-Shp2-/-

skin. We appreciate the effort of the authors, however, the quality of the IF stainings is poor due to the high background of the anti-Shp2 but also the anti-CD11c antibody. Therefore it is difficult to confirm the specific deletion in DC compared to macrophages. However, if the authors are convinced of these data, they should also include them into the manuscript since this is an important information for the readers.
The same applies to point 6. The authors provide quantifications of their WB in Response Fig. 5. however, these data are important also for the readers so they should be included in the manuscript!
previous point 4) There seems to be a misunderstanding. Thiis reviewer did not doubt that TLR7 is upstream of IL-23. However, data from Fig. EV1 and App.S20 indicated that Shp and TLR7 were also downstream of Il23 and this should be explained and discussed.
minor: In line 387 the authors included "lately" into the text of the figure legend, however, correct would be "late".

***** Reviewer's comments *****

Referee #1 (Remarks for Author):

Zhu et al. have made a tremendous effort in the revision of this manuscript. Overall, we think that the data provided are solid and in general support the hypothesis. Most of our criticism still applies on the authors insisting on an exclusive role of Shp2 in macrophages. On several occasions we have tried to convince the authors to weaken their point and discuss the potential role of Shp2 / ptpn11 also in other cell types. Rather than to accept this, the authors decided to produce and include more and more data into their manuscript, sometimes even data that did not support their hypothesis or raised even new questions.

Reply: We thank the reviewer for his/her confirmation of our work. We fully agree with the reviewer and have reformulated our hypothesis in this revised manuscript.

This applies also to the present revision, which provides very interesting data but again raised more questions than it answered our points. Thus, we again suggest the authors should tune down their conclusion on insisting on the macrophages and broaden this to other cell types of the myeloid lineage, which by no means would affect the interest and the impact of the paper....

Reply: Thanks for the reviewer's encouragement and suggestions, which we have corrected in the revised manuscript.

In their reply to the reviewers general comment in the introduction the authors claim that they "reformulated their hypothesis accordingly" however, abstract and graphical abstract have not been changed at all.

Reply: We are sorry for your trouble. In the previous version of the manuscript, we made changes in the text and in the images. As suggested by the reviewer, we have made additional changes in the abstract and graphic abstract in this revised manuscript.

previous point 1) New data presented in Appendix Fig. 14 and 17 are valuable, although they do not fully explain the points raised by the reviewer about expression of ptpn11 in other cell types, since the authors give mainly insight into macrophages and dendritic cells but no other cell types.

Reply: This is a very valid concern raised by the reviewer. We found by scRNA-seq of mouse skin that SHP099 inhibited the expression of inflammation-associated genes in myeloid cells, which have the highest number of macrophages, and that hub genes are mainly expressed in macrophages and Langerhans cells. We demonstrated the important role of SHP2 in myeloid cells in psoriasis with the help of myeloid cell lineages (monocyte-, mature macrophage- and granulocyte)-specific SHP2 conditional knockout mice, while the role of SHP2 in neutrophils in psoriasis was studied by colleagues. Considering that DCs play a crucial role in psoriasis development, we also constructed dendritic cells-specific SHP2 conditional knockout mice. Surprisingly, SHP2 in DCs did not affect psoriasis progression in mice. We fully agree with the important role of DCs in psoriasis; therefore, although we focused on macrophages in subsequent experiments, we also explored the role of *PTPN11* in DCs in Appendix Figure 14 and Appendix Figure 17. We added the correlation analysis of *PTPN11* with the gene expression of the NF-kappa B pathway in mast cells in Appendix Figure 17. SHP2, as a widely expressed protein, plays an important

role in a variety of cells, and it is difficult to study them thoroughly in one project. In this project, we focused on macrophages, and mechanistic studies were all carried out on macrophages; hence, this part of the conclusion is the role of SHP2 in macrophages. In contrast, the conclusions regarding myeloid cell-specific SHP2 conditional knockout mice emphasize SHP2 in myeloid cells.

In App.Fig.14 some questions remain and there is need for more detailed explanations: It is difficult to see the colors of the clusters assigned in A. It is unclear why the authors focus on the two clusters 1 and 3. Whereas Cluster 1 seems to include a number of different immune cell types, the identity of cells contained in Cluster 3 cannot be easily determined from the data provided in panel B. In G the authors show a summary of the distribution of the different cell types as pie charts overlaying the section. However, there is discrepancy between this analysis and the sections shown in E. The keratinocyte contribution seems to be much higher in G (shown in pink). The distribution of CD68 shown in E is not reflected by the representation of macrophages in G. The authors should please check this and if this is correct the underlying possible mechanism for this difference should be explained / discussed.

Reply: We apologize for the confusion and would like to provide the following explanation. In Appendix Figure 14A, we have enlarged the figure legend points, which seems to be the cause of mixing the six colors. We found that most of cluster 1 belonged to the papillary dermis, while cluster 3 was scattered, but part of it existed in the reticular dermis, and another part was scattered in the lower part of the reticular dermis near the subcutaneous area where T cells, lymphotic endothelial cells and mesenchymal stem cells existed. We thought they all belonged to dermis-existing cells involved in the response to psoriasis. E retained all spots expressing *PTPN11* in cluster 1 and cluster 3 and then checked the expression of *CD68* and *ITGAX* on these spots. G was not related to E. G extracted all spots in all cluster 1 and cluster 3 and calculated the cell proportion of each spot by using the spotlight algorithm. This algorithm was combined with the single-cell transcriptome data of Appendix Figure 17A. Thus, E shared the same cell types in Appendix Figure 17A.

We present our views from two perspectives:

In terms of expression, E shows the expression of *CD68* and *ITGAX* in spots that expressed *PTPN11*. In terms of cell proportion per spot, G shows the proportion of cells per spot in the dermis of normal slices more than in the psoriasis slice.

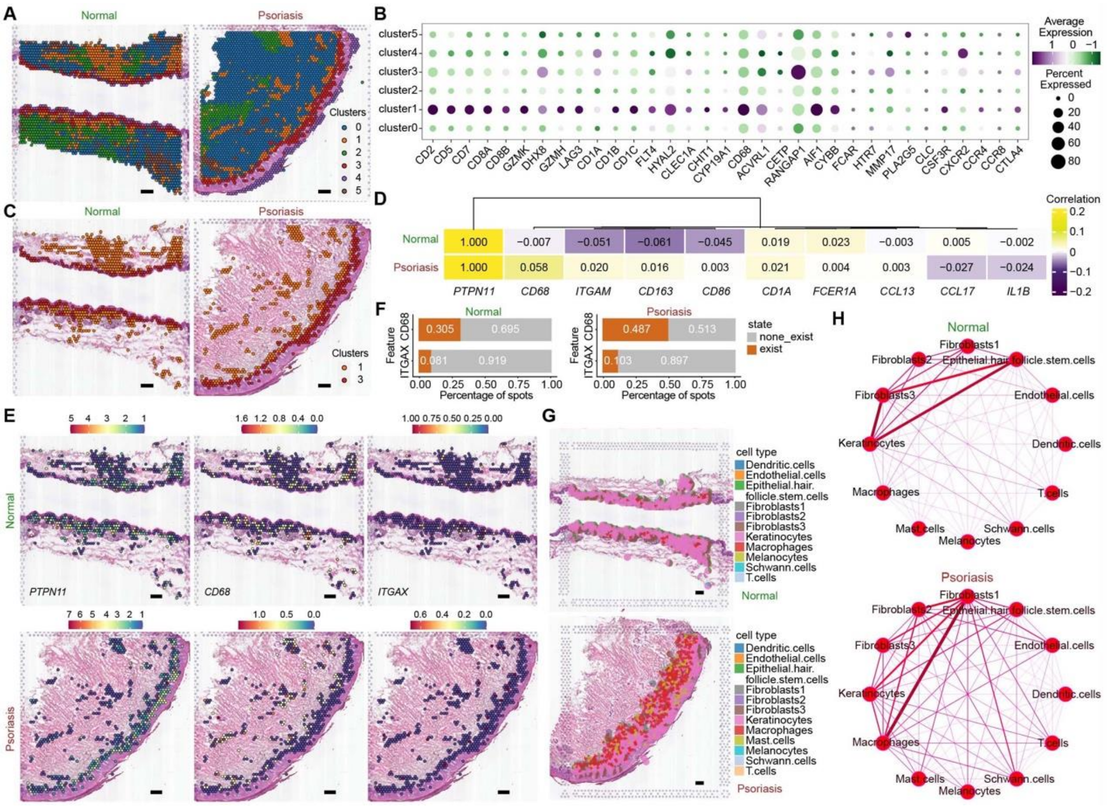

Appendix Fig S14. Spatial transcriptome sequencing analysis revealed that *PTPN11* was most strongly correlated with macrophages in lesional psoriatic skin.

A Six clusters reflecting six anatomical regions on the psoriasis slice and the normal slice. Scale bar: 500 µm.

B Dotplot showing some immune cell marker genes of all cell clusters in (A).

C Prominence of Cluster1 and Cluster3 in the dermis of psoriatic slice and normal slice. Scale bar: 500 µm.

D Heatmap showing the correlation coefficient between *PTPN11* and other genes in two groups respectively.

E Only spots expressing *PTPN11* shown in two slices (*top*) with the expression of *CD68* (*medium*) and *ITGAX* (*bottom*) on these spots. Scale bar: 500 µm.

F Barplot showing the percentage of the spots which expressing *CD68* and *ITGAX* in all spots expressing *PTPN11*, both shown in normal and psoriasis slices. Significant differences between the two groups were calculated by Fisher's exact test. The normal group was 7.623e-23, and the psoriasis was 4.542e-49.

G Spots in Cluster1 and Cluster3 showing the percentage of cell types by Scatterpieplot. Scale bar: 500 µm.

H The cell-cell interaction network between cell types shown in (G). The thickness of the connecting line indicates the strength of the interaction.

In App.Fig 17 the authors claim that ptpn11 expression in macrophages but not so much in DC correlates with expression of genes of the NfKB pathway. Again, information on the other cell types would be important.

Reply: We have followed the reviewer's helpful suggestion. We analyzed the correlation between the expression of *PTPN11* in myeloid cells including macrophages, dendritic cells, and mast cells and the gene expression of NF-kappa B pathway. We found that *PTPN11* was positively more correlated with the NF-kappa B pathway in macrophages than dendritic cells and mast cells from psoriasis lesions, suggesting that *PTPN11* exacerbates psoriasis mainly in macrophages by activating the NF-kappa B pathway.

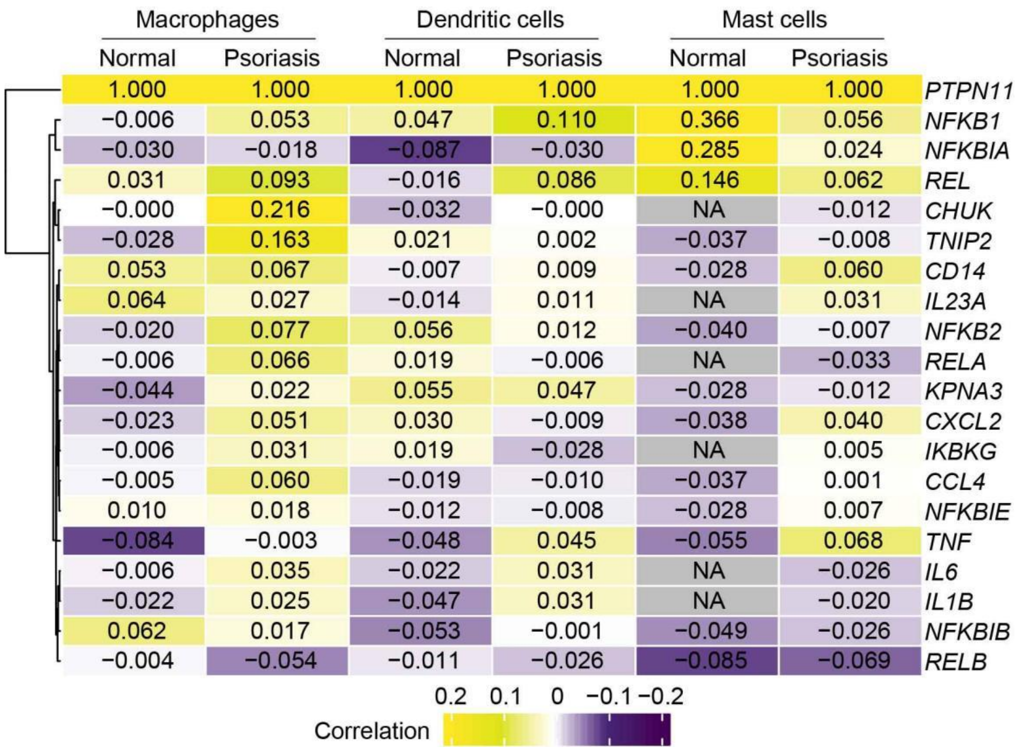


Appendix Fig S17C. Heatmap showing the correlation coefficient between *PTPN11* and each gene from NF-kappa B signaling pathway in six groups (Macrophages in Normal, Macrophages in Psoriasis, Dendritic cells in Normal, Dendritic cells in Psoriasis, Mast cells in Normal, and Mast cells in Psoriasis) respectively.

previous point 2) In response to point 2 the authors provide IF stainings to show deletion in DC or macrophages in DC-Shp2-/- skin. We appreciate the effort of the authors, however, the quality of the IF stainings is poor due to the high background of the anti-Shp2 but also the antiCD11c antibody. Therefore it is difficult to confirm the specific deletion in DC compared to macrophages. However, if the authors are convinced of these data, they should also include them into the manuscript since this is an important information for the readers.

Reply: Thanks to the understanding of the reviewer, we have added this part of the data in Appendix Figure S10.

The same applies to point 6. The authors provide quantifications of their WB in Response Fig. 5. however, these data are important also for the readers so they should be included in the manuscript!

Reply: Thanks to the reviewer's suggestion. As there is more data in the manuscript, the Raw WB data cannot be placed in the appendix file, although we have uploaded to the source data, which we once again place in the response letter for readers to review. The response letter is also available for the readers to read and download.

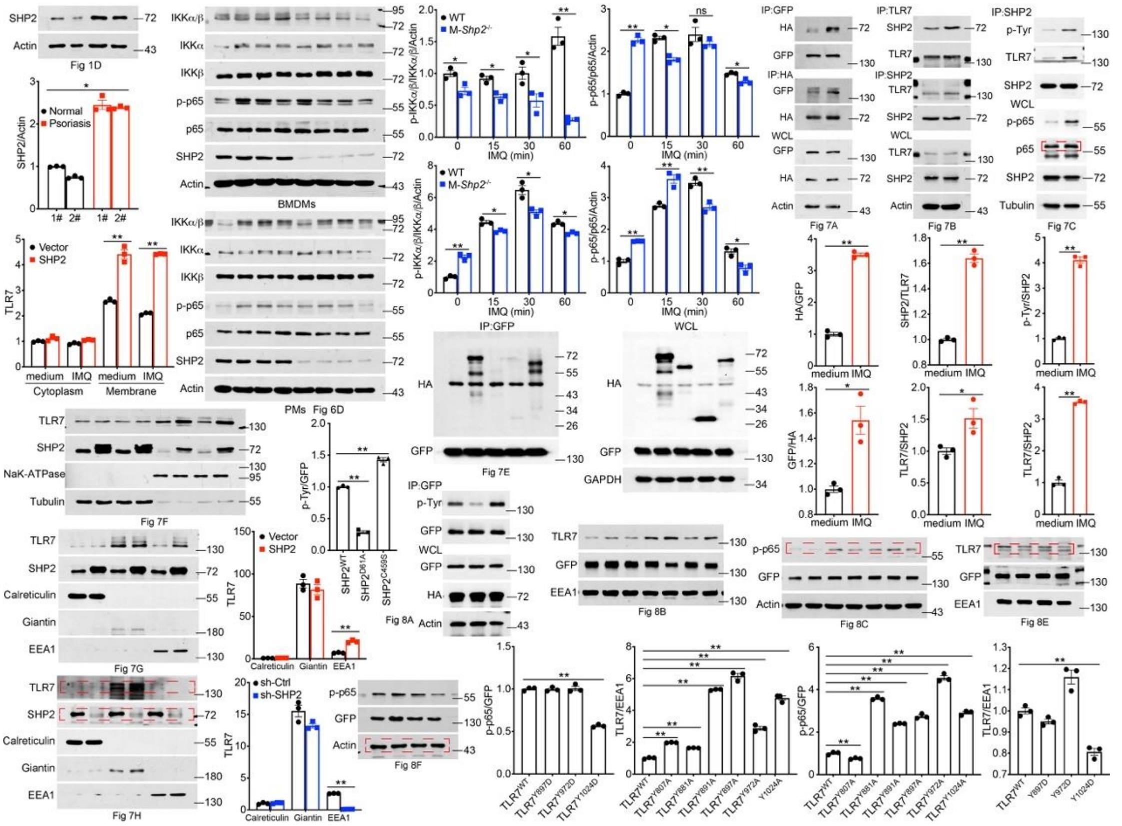


Raw Data and quantifications of Western blot in Figures.

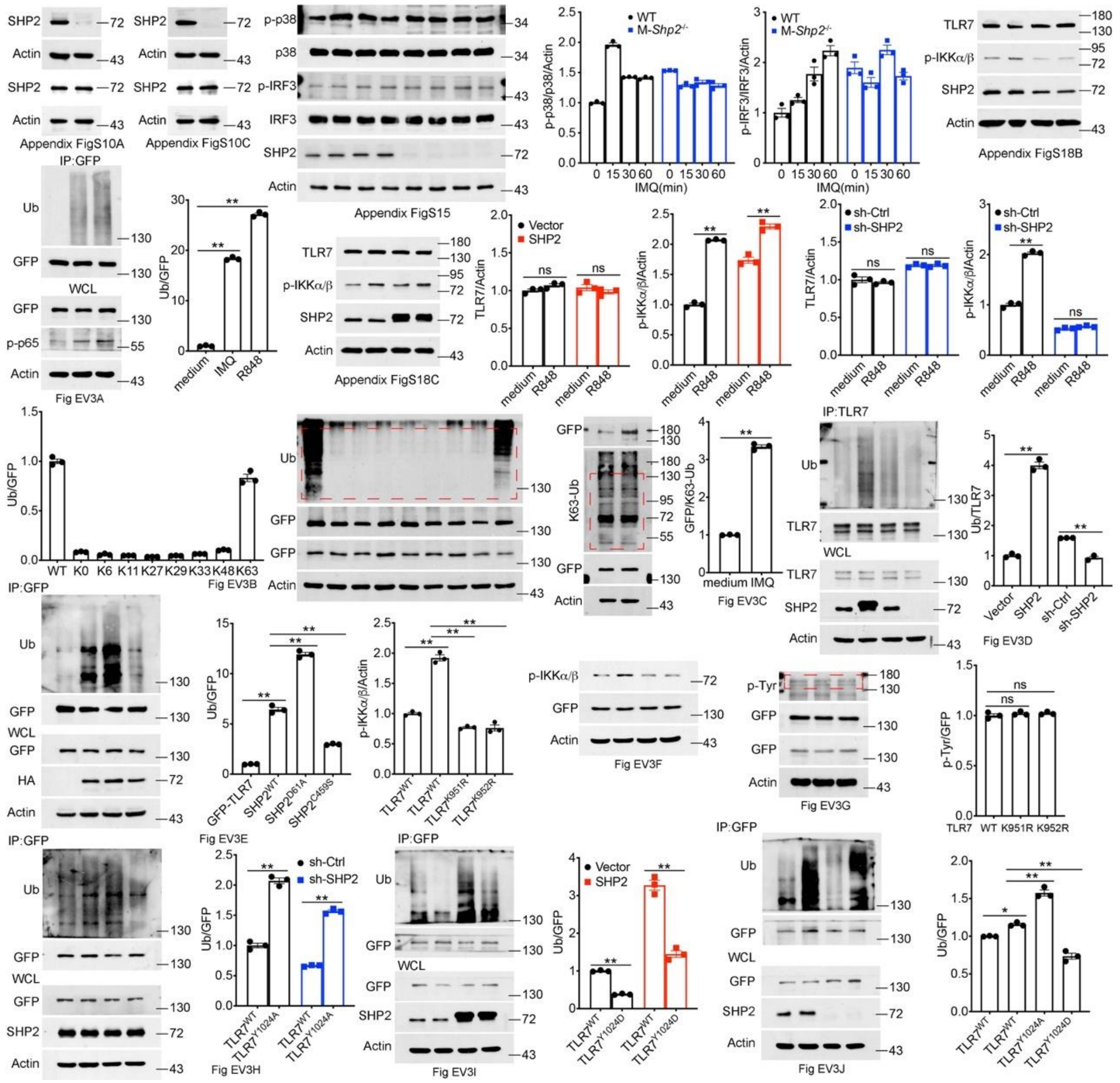


Raw Data and quantifications of Western blot in EV and Appendix Figures.

previous point 4) There seems to be a misunderstanding. This reviewer did not doubt that TLR7 is upstream of IL-23. However, data from Fig. EV1 and App.S20 indicated that Shp and TLR7 were also downstream of Il23 and this should be explained and discussed.

Reply: This is a very good question. In a complex disease state, a purely upstream-downstream relationship is not a good conclusion to draw. When IL-23 cytokine is injected in the skin of mice, an inflammatory response is triggered and the inflammatory factors produced act on a variety of cells involved in psoriasis, such as macrophages, and TLR7 is activated, causing downstream NF-κB activation. Thus, either administration of SHP2 inhibitor or mutation of TLR7 also ameliorates disease progression in mice.

minor: In line 387 the authors included "lately" into the text of the figure legend, however, correct would be "late".

Reply: We thank the reviewer for reading our manuscript carefully and pointing out this mistake. We have corrected the error in the revised manuscript.

Thanks again for the Editor and Reviewers' precious comments and suggestions.

We are pleased to inform you that your manuscript is accepted for publication and is now being sent to our publisher to be included in the next available issue of EMBO Molecular Medicine. Please be aware that all deposited data have to be freely available before publication of the manuscript.

EMBO PRESS

YOU MUST COMPLETE ALL CELLS WITH A PINK BACKGROUND

PLEASE NOTE THAT THIS CHECKLIST WILL BE PUBLISHED ALONGSIDE YOUR PAPER

Corresponding Author Name: Dijun Chen, Qiang Xu, Yang Sun
Journal Submitted to: EMBO Molecular Medicine
Manuscript Number: EMM-2021-14455

## Reporting Checklist For Life Sciences Articles (Rev. June 2017)

This checklist is used to ensure good reporting standards and to improve the reproducibility of published results. These guidelines are consistent with the Principles and Guidelines for Reporting Preclinical Research issued by the NIH in 2014. Please follow the journal's authorship guidelines in preparing your manuscript.

### A- Figures

#### 1. Data

#### The data shown in figures should satisfy the following conditions:

- the data were obtained and processed according to the field's best practice and are presented to reflect the results of the experiments in an accurate and unbiased manner.
- figure panels include only data points, measurements or observations that can be compared to each other in a scientifically meaningful way.
- graphs include clearly labeled error bars for independent experiments and sample sizes. Unless justified, error bars should not be shown for technical replicates.
- if n< 5, the individual data points from each experiment should be plotted and any statistical test employed should be justified
- Source Data should be included to report the data underlying graphs. Please follow the guidelines set out in the author ship guidelines on Data Presentation.

#### 2. Captions

#### Each figure caption should contain the following information, for each panel where they are relevant:

- a specification of the experimental system investigated (eg cell line, species name).
- the assay(s) and method(s) used to carry out the reported observations and measurements
- an explicit mention of the biological and chemical entity(ies) that are being measured.
- an explicit mention of the biological and chemical entity(ies) that are altered/varied/perturbed in a controlled manner.

- the exact sample size (n) for each experimental group/condition, given as a number, not a range;
- a description of the sample collection allowing the reader to understand whether the samples represent technical or biological replicates (including how many animals, litters, cultures, etc.).
- a statement of how many times the experiment shown was independently replicated in the laboratory.
- definitions of statistical methods and measures:
  - common tests, such as t-test (please specify whether paired vs. unpaired), simple χ2 tests, Wilcoxon and Mann-Whitney tests, can be unambiguously identified by name only, but more complex techniques should be described in the methods section;
  - are tests one-sided or two-sided?
  - are there adjustments for multiple comparisons?
  - exact statistical test results, e.g., P values = x but not P values < x;
  - definition of 'center values' as median or average;
  - definition of error bars as s.d. or s.e.m.

Any descriptions too long for the figure legend should be included in the methods section and/or with the source data.

**In the pink boxes below, please ensure that the answers to the following questions are reported in the manuscript itself. Every question should be answered. If the question is not relevant to your research, please write NA (non applicable). We encourage you to include a specific subsection in the methods section for statistics, reagents, animal models and human subjects.**

## USEFUL LINKS FOR COMPLETING THIS FORM

http://www.antibodypedia.com
http://1degreebio.org
http://www.equator-network.org/reporting-guidelines/improving-bioscience-research-repor
http://grants.nih.gov/grants/olaw/olaw.htm
http://www.mrc.ac.uk/Ourresearch/Ethicsresearchguidance/Useofanimals/index.htm
http://ClinicalTrials.gov
http://www.consort-statement.org
http://www.consort-statement.org/checklists/view/32-consort/66-title
http://www.equator-network.org/reporting-guidelines/reporting-recommendations-for-tumo
http://datadryad.org
http://figshare.com
http://www.ncbi.nlm.nih.gov/gap
http://www.ebi.ac.uk/ega
http://biomodels.net/
http://biomodels.net/miriam/
http://jjj.biochem.sun.ac.za
https://osp.od.nih.gov/biosafety-biosecurity-and-emerging-biotechnology/
http://www.selectagents.gov/

### B- Statistics and general methods

Please fill out these boxes (Do not worry if you cannot see all your text once you press return)

| | |
|---|---|
| 1.a. How was the sample size chosen to ensure adequate power to detect a pre-specified effect size? | The sample size was chosen based on our experience from previous studies. No statistical power analysis was conducted. |
| 1.b. For animal studies, include a statement about sample size estimate even if no statistical methods were used. | Statistical analysis was done on all experiment that we used mice. We have indicated the mouse numbers in Figure legends . |
| 2. Describe inclusion/exclusion criteria if samples or animals were excluded from the analysis. Were the criteria pre-established? | No samples were excluded from analysis. |
| 3. Were any steps taken to minimize the effects of subjective bias when allocating animals/samples to treatment (e.g. randomization procedure)? If yes, please describe. | The animals were randomized according to their body weight before starting therapy to avoid any bias. |
| For animal studies, include a statement about randomization even if no randomization was used. | The animals were randomized according to their body weight before starting therapy to avoid any bias. |
| 4.a. Were any steps taken to minimize the effects of subjective bias during group allocation or/and when assessing results (e.g. blinding of the investigator)? If yes please describe. | Measurement of skin thickness was performed blinded by an independent scientist. |
| 4.b. For animal studies, include a statement about blinding even if no blinding was done | No blinding of animals was possible. |
| 5. For every figure, are statistical tests justified as appropriate? | Yes, statistical tests were justified as appropriate. |
| Do the data meet the assumptions of the tests (e.g., normal distribution)? Describe any methods used to assess it. | Statistical analysis was performed using GraphPad Prism 9.0. Data are presented as the mean ± SEM. We assessed data for normal distribution and similar variance between groups. Unpaired two-tailed student's t-test, Mann-Whitney U test, one-way ANOVA with Tukey or Bonferroni multiple comparison test were used to assess statistical significance (*P < 0.05, **P < 0.01, ns, not significant). Error bars depict SEM. |
| Is there an estimate of variation within each group of data? | No, there was no estimate of variation within each group of data. |

| | |
|---|---|
| Is the variance similar between the groups that are being statistically compared? | All data in our experiments were statistically analyzed. |

## C- Reagents

| | |
|---|---|
| 6. To show that antibodies were profiled for use in the system under study (assay and species), provide a citation, catalog number and/or clone number, supplementary information or reference to an antibody validation profile. e.g., Antibodypedia (see link list at top right), 1DegreeBio (see link list at top right). | Antibodies used in our study have dilution information, source company informations, and catalog number in Materials and Methods. Pages 22, 23, 29, 20. |
| 7. Identify the source of cell lines and report if they were recently authenticated (e.g., by STR profiling) and tested for mycoplasma contamination. | HEK293T cell line and THP-1 cell line were purchased from ATCC. Human peripheral blood mononuclear cells, mouse bone marrow derived macrophages and dendritic cells were differenciated from fresh human peripheral blood and mouse bone marrow cells, respectively. All cells were negative for mycoplasma. |

* for all hyperlinks, please see the table at the top right of the document

## D- Animal Models

| | |
|---|---|
| 8. Report species, strain, gender, age of animals and genetic modification status where applicable. Please detail housing and husbandry conditions and the source of animals. | C57BL/6J mice were purchased from GemPharmatech Co. Ltd. The macrophage-specific SHP2 knockout mice (Mø-Shp2-/- mice) were generated by crossing Shp2 floxed mice with Lyz2-cre transgenic mice (Guo et al., 2017). The dendritic cell-specific SHP2 knockout mice (DC-Shp2-/- mice) were generated by crossing Shp2 floxed mice with Itgax-cre transgenic mice (Caton et al., 2007; Durai and Murphy, 2016), which were purchased from Jackson Laboratory, JAX: 008068. We purchased B6.129S1-Tlr7tm1Flv/J mice (Tlr7mut/wt mice, TLR7 knock-out mice) from Jackson Laboratory, JAX: 008380. We generated Tlr7-Y1025D point mutation mice via CRISPR/Cas9 system. All mice were on the C57BL/6 background. Age- and sex-matched mice 6-10 weeks of age were used. Mice had unlimited access to standard laboratory chow and water under a light dark cycle of 12 h in a room with a temperature of 22° C. |
| 9. For experiments involving live vertebrates, include a statement of compliance with ethical regulations and identify the committee(s) approving the experiments. | Animal welfare and experimental procedures were approved by the Institutional Animal Care and Use Committee at Nanjing University and were approved by the Experimental Animal Care and Use Committee of Nanjing University (IACUC-2006024) . All efforts were made to reduce the number of animals used and to minimize animal suffering. |
| 10. We recommend consulting the ARRIVE guidelines (see link list at top right) (PLoS Biol. 8(6), e1000412, 2010) to ensure that other relevant aspects of animal studies are adequately reported. See author guidelines, under 'Reporting Guidelines'. See also: NIH (see link list at top right) and MRC (see link list at top right) recommendations. Please confirm compliance. | We confirm compliance. |

## E- Human Subjects

| | |
|---|---|
| 11. Identify the committee(s) approving the study protocol. | The ethics committee of West China Hospital, Sichuan University. |
| 12. Include a statement confirming that informed consent was obtained from all subjects and that the experiments conformed to the principles set out in the WMA Declaration of Helsinki and the Department of Health and Human Services Belmont Report. | All human studies were approved by the ethics committee of West China Hospital, Sichuan University. Psoriatic patients were recruited by the Department of Dermatology and Venereology at West China Hospital under protocol 2019-R-513. Written informed consent was obtained from all subjects. |
| 13. For publication of patient photos, include a statement confirming that consent to publish was obtained. | NA |
| 14. Report any restrictions on the availability (and/or on the use) of human data or samples. | NA |
| 15. Report the clinical trial registration number (at ClinicalTrials.gov or equivalent), where applicable. | NA |
| 16. For phase II and III randomized controlled trials, please refer to the CONSORT flow diagram (see link list at top right) and submit the CONSORT checklist (see link list at top right) with your submission. See author guidelines, under 'Reporting Guidelines'. Please confirm you have submitted this list. | NA |
| 17. For tumor marker prognostic studies, we recommend that you follow the REMARK reporting guidelines (see link list at top right). See author guidelines, under 'Reporting Guidelines'. Please confirm you have followed these guidelines. | NA |

## F- Data Accessibility

| | |
|---|---|
| 18: Provide a "Data Availability" section at the end of the Materials & Methods, listing the accession codes for data generated in this study and deposited in a public database (e.g. RNA-Seq data: Gene Expression Omnibus GSE39462, Proteomics data: PRIDE PXD000208 etc.) Please refer to our author guidelines for 'Data Deposition'.<br><br>Data deposition in a public repository is mandatory for:<br>a. Protein, DNA and RNA sequences<br>b. Macromolecular structures<br>c. Crystallographic data for small molecules<br>d. Functional genomics data<br>e. Proteomics and molecular interactions | The authors declare that all data supporting the findings of this study are available within the paper. RNA-seq data is available from GEO under the ID GSE147535.scRNA-seq data have been deposited to the GEO database with accession number GSE165021. |
| 19. Deposition is strongly recommended for any datasets that are central and integral to the study; please consider the journal's data policy. If no structured public repository exists for a given data type, we encourage the provision of datasets in the manuscript as a Supplementary Document (see author guidelines under 'Expanded View' or in unstructured repositories such as Dryad (see link list at top right) or Figshare (see link list at top right). | RNA-seq and scRNA-seq data is available in GEO. |
| 20. Access to human clinical and genomic datasets should be provided with as few restrictions as possible while respecting ethical obligations to the patients and relevant medical and legal issues. If practically possible and compatible with the individual consent agreement used in the study, such data should be deposited in one of the major public access-controlled repositories such as dbGAP (see link list at top right) or EGA (see link list at top right). | NA |
| 21. Computational models that are central and integral to a study should be shared without restrictions and provided in a machine-readable form. The relevant accession numbers or links should be provided. When possible, standardized format (SBML, CellML) should be used instead of scripts (e.g. MATLAB). Authors are strongly encouraged to follow the MIRIAM guidelines (see link list at top right) and deposit their model in a public database such as Biomodels (see link list at top right) or JWS Online (see link list at top right). If computer source code is provided with the paper, it should be deposited in a public repository or included in supplementary information. | NA |

## G- Dual use research of concern

| | |
|---|---|
| 22. Could your study fall under dual use research restrictions? Please check biosecurity documents (see link list at top right) and list of select agents and toxins (APHIS/CDC) (see link list at top right). According to our biosecurity guidelines, provide a statement only if it could. | NA |
